# Supplementary material for: Pre-admission interventions (prehabilitation) to improve outcome after major elective surgery: a systematic review and meta-analysis
Source: BMJ Open. 2021 Sep 30;11(9):e050806. doi: 10.1136/bmjopen-2021-050806 (PMC8487197; doi:10.1136/bmjopen-2021-050806)
Supplement: Supplementary data [file bmjopen-2021-050806supp001.pdf]

Pre-admission interventions (prehabilitation) to improve outcome after major elective surgery: A systematic review and meta-analysis

Supplementary material

Table of Contents

Search strategy 9

Characteristics of studies and results 12

NUTRITIONAL INTERVENTIONS 12

Table 1. Characteristics of studies 12
Table 2. Results 33
Table 3. Risk of Bias 52
Table 4. Summary of findings (Immunonutrition) 56
Table 5. Summary of findings (Oral Nutritional Supplements) 57
Table 6. Summary of findings (Pre/Probiotics) 58
Table 7. Summary of findings (Weight Loss) 59

Meta analyses 60

IMMUNONUTRITION 60

Figure 1. Random effects meta-analysis of the risk ratio of mortality between immunonutrition (experimental) and usual care (control) 60

Figure 2. Random effects meta-analysis of the mean difference in length of hospital stay (LoS) between immunonutrition (experimental) and usual care (control) 60

Figure 3. Random effects meta-analysis of the risk ratio of total postoperative complications (Infective & non-infective) between immunonutrition (experimental) and usual care (control) 61

Figure 4. Random effects meta-analysis of the risk ratio of total infective complications between immunonutrition (experimental) and usual care (control). 61

Figure 5. Random effects meta-analysis of the risk ratio of pneumonia between immunonutrition (experimental) and usual care (control) 61

Figure 6. Random effects meta-analysis of the risk ratio of wound infection between immunonutrition (experimental) and usual care (control) 62

ORAL NUTRITIONAL SUPPLEMENTS (ONS) 62

Figure 7. Random effects meta-analysis of the risk ratio of mortality between ONS (experimental) and usual care (control). 62

Figure 8. Random effects meta-analysis of the mean difference in length of hospital stay (LoS) between oral nutritional supplements (experimental) and usual care (control). 62

WEIGHT LOSS INTERVENTIONS 63

Figure 9. Random effects meta-analysis of the mean difference in length of hospital stay (LoS) between weight loss interventions (experimental) and usual care (control). 63

PRE/PROBIOTICS 63

Perry R, *et al. BMJ Open* 2022; 11:e050806. doi: 10.1136/bmjopen-2021-050806

Figure 10. Random effects meta-analysis of the risk ratio of mortality between pre/probiotic interventions (experimental) and usual care (control). 63

Figure 11. Random effects meta-analysis of the risk ratio of total PO infective complications between pre/probiotic interventions (experimental) and usual care (control). 63

Sensitivity analyses (removing studies at high risk of bias)........ 64

IMMUNONUTRITION 64

Figure 12. Random effects meta-analysis of the risk ratio of mortality between immunonutrition (experimental) and usual care (control) with high risk of bias studies removed. 64

Figure 13. Random effects meta-analysis of the mean difference in length of stay between immunonutrition (experimental) and usual care (control) with high risk of bias studies removed. 64

Sensitivity analyses (removing studies with imputed results)........ 65

IMMUNONUTRITION 65

Figure 14. Random effects meta-analysis of the mean difference in length of stay between immunonutrition (experimental) and usual care (control) with imputed results removed. 65

ORAL NUTRITIONAL SUPPLEMENTS (ONS) 65

Figure 15. Random effects meta-analysis of the mean difference in length of stay between ONS (experimental) and usual care (control) with imputed results removed. 65

Subgroup analyses (type of surgery) 66

IMMUNONUTRITION (CANCER SURGERY) 66

Figure 16. Random effects meta-analysis of the risk ratio of mortality between immunonutrition (experimental) and usual care (control) for those undergoing cancer surgery. 66

Figure 17. Random effects meta-analysis of the mean difference in length of hospital stay (LoS) between immunonutrition (experimental) and usual care (control) for those undergoing cancer surgery. 66

ORAL NUTRITIONAL SUPPLEMENTS (ONS) (CANCER SURGERY) 66

Figure 18. Random effects meta-analysis of the risk ratio of mortality between ONS (experimental) and usual care (control) for those undergoing cancer surgery........ 66

Subgroup analysis (studies published before and after 2010)........ 67

IMMUNONUTRITION (STUDIES PUBLISHED AFTER 2010) 67

Figure 19. Random effects meta-analysis of the risk ratio of mortality between immunonutrition (experimental) and usual care (control) in studies published after 2010. 67

Figure 20. Random effects meta-analysis of the mean difference in length of hospital stay (LoS) between immunonutrition (experimental) and usual care (control) in studies published after 2010. 67

Figure 21. Random effects meta-analysis of the risk ratio of total postoperative complications between immunonutrition (experimental) and usual care (control) in studies published after 2010. 68

Figure 22. Random effects meta-analysis of the risk ratio of total infective complications between immunonutrition (experimental) and usual care (control) in studies published after 2010. 68

Figure 23. Random effects meta-analysis of the risk ratio of penumonia between immunonutrition (experimental) and usual care (control) in studies published after 2010. 68

Figure 24. Random effects meta-analysis of the risk ratio of wound infection between immunonutrition (experimental) and usual care (control) in studies published after 2010. 69

ORAL NUTRITIONAL SUPPLEMENTS (ONS) (STUDIES PUBLISHED AFTER 2010) 69

Figure 25. Random effects meta-analysis of the risk ratio of mortality between ONS (experimental) and usual care (control) in studies published after 2010. 69

Figure 26. Random effects meta-analysis of the mean difference in length of hospital stay (LoS) between ONS (experimental) and usual care (control) in studies published after 2010. 70

WEIGHT LOSS (STUDIES PUBLISHED AFTER 2010) 70

Figure 27. Random effects meta-analysis of the mean difference in length of hospital stay (LoS) between weight loss interventions (experimental) and usual care (control) in studies published after 2010. 70

PRE/PROBIOTICS (STUDIES PUBLISHED AFTER 2010) 70

Figure 28. Random effects meta-analysis of the risk ratio of mortality between pre/probiotics (experimental) and usual care (control) in studies published after 2010. 70

References 71

EXERCISE INTERVENTIONS 75

Table 8. Characteristics of studies 75

Table 9. Results 88

Table 10. Risk of bias 99

Table 11. Summary of findings 101

Meta analyses 102

Figure 29. Random effects meta-analysis of the risk ratio of mortality between exercise (experimental) and usual care (control). 102

Figure 30. Random effects meta-analysis of the mean difference in length of hospital stay (LoS) between exercise (experimental) and usual care (control). 102

Figure 31. Random effects meta-analysis of the risk ratio of total PO complications (infective & non-infective) between exercise interventions (experimental) and usual care (control). 103

Figure 32. Random effects meta-analysis of the risk ratio of pneumonia between exercise interventions (experimental) and usual care (control). 103

Figure 33. Random effects meta-analysis of the risk ratio of PPCs between exercise interventions (experimental) and usual care (control). 103

Sensitivity analyses (removing studies at high risk of bias) 104

Perry R, *et al. BMJ Open* 2022; 11:e050806. doi: 10.1136/bmjopen-2021-050806

Figure 34. Random effects meta-analysis of the mean difference in length of stay between exercise interventions (experimental) and usual care (control) with high risk of bias studies removed. 104

Sensitivity analyses (removing studies with imputed results) 104

Figure 35. Random effects meta-analysis of the mean difference in length of stay between exercise interventions (experimental) and usual care (control) with imputed results removed. done 104

Subgroup analyses (type of surgery) 105

Figure 36. Random effects meta-analysis of the mean difference in length of hospital stay (LoS) between exercise (experimental) and usual care (control) for patients undergoing cancer surgery. 105

Subgroup analyses (studies published before and after 2010) 105

Figure 37. Random effects meta-analysis of the mean difference in length of hospital stay (LoS) between immunonutrition (experimental) and usual care (control) in studies published after 2010. 105

References 106

RESPIRATORY INTERVENTIONS 108

Table 12. Characteristics of studies 108
Table 13. Results 119
Table 14. Risk of bias 129
Table 15. Summary of findings (inspiratory muscle training) 132
Table 16. Summary of findings (incentive spirometry) 133

Meta analyses 134

INSPIRATORY MUSCLE TRAINING 134

Figure 38. Random effects meta-analysis of the risk ratio of mortality between IMT (experimental) and usual care (control). 134

Figure 39. Random effects meta-analysis of the mean difference in length of hospital stay (LoS) between IMT (experimental) and usual care (control). 134

Figure 40. Random effects meta-analysis of the risk ratio of total PPCs between IMT (experimental) and usual care (control). 135

Figure 41. Random effects meta-analysis of the risk ratio of pneumonia between IMT (experimental) and usual care (control). 135

INCENTIVE SPIROMETRY 135

Figure 42. Random effects meta-analysis of the mean difference in length of hospital stay (LoS) between incentive spirometry (experimental) and usual care (control). 135

Figure 43. Random effects meta-analysis of the risk ratio of PPCs between incentive spirometry (experimental) and usual care (control). 136

Sensitivity analyses (removing studies at high risk of bias) 137

INSPIRATORY MUSCLE TRAINING 137

Sensitivity analyses (removing studies with imputed results) 137

INSPIRATORY MUSCLE TRAINING 137

Figure 44.Random effects meta-analysis of the mean difference in length of stay between IMT (experimental) and usual care (control) with imputed results removed. 137

Subgroup analyses (type of surgery) 137

INSPIRATORY MUSCLE TRAINING (CARDIAC SURGERY).. 137

Figure 45. Random effects meta-analysis of the mean difference in length of hospital stay (LoS) between IMT (experimental) and usual care (control) for those undergoing cardiac surgery. 137

Figure 46. Random effects meta-analysis of the risk ratio of pneumonia between IMT (experimental) and usual care (control) for those undergoing cardiac surgery................ 138

Figure 47. Random effects meta-analysis of the risk ratio of post-operative pulmonary complications between IMT (experimental) and usual care (control) for those undergoing cardiac surgery. 138

Subgroup analysis (studies published before and after 2010).. 139

INSPIRATORY MUSCLE TRAINING 139

Figure 48. Random effects meta-analysis of the risk ratio of mortality between inspiratory muscle training (experimental) and usual care (control) in studies published after 2010. 139

Figure 49. Random effects meta-analysis of the mean difference in length of hospital stay (LoS) between inspiratory muscle training (experimental) and usual care (control) in studies published after 2010. 139

Figure 50. Random effects meta-analysis of the mean difference in length of hospital stay (LoS) between inspiratory muscle training (experimental) and usual care (control) in studies published before 2010... 140

Figure 51. Random effects meta-analysis of the risk ratio of postoperative pulmonary complications (PPC) between inspiratory muscle training (experimental) and usual care (control) in studies published after 2010.. 140

Figure 52.Random effects meta-analysis of the risk ratio of penumonia between inspiratory muscle training (experimental) and usual care (control) in studies published after 2010. 140

Figure 53. Random effects meta-analysis of the risk ratio of penumonia between inspiratory muscle training (experimental) and usual care (control) in studies published before 2010. 141

References 141

MULTIMODAL INTERVENTIONS 144

Table 17. Characteristics of studies 144
Table 18. Results 155
Table 19. Risk of bias 167
Table 20. Summary of findings 170

Meta analyses 171

Figure 54. Random effects meta-analysis of the risk ratio of mortality between multimodal (experimental) and usual care (control). 171

Figure 55. Random effects meta-analysis of the mean difference in length of hospital stay (LoS) between multimodal intervention (experimental) and usual care (control)............. 171

Figure 56. Random effects meta-analysis of the risk ratio of pneumonia between multimodal interventions (experimental) and usual care (control). 172

Figure 57. Random effects meta-analysis of the risk ratio of total PO complications between multimodal interventions (experimental) and usual care (control). 172

Sensitivity analyses (removing studies at high risk of bias)....... 173

Figure 58. Random effects meta-analysis of the risk ratio of mortality between multimodal (experimental) and usual care (control) with high risk of bias studies removed. 173

Figure 59. Random effects meta-analysis of the mean difference in length of stay between multimodal interventions (experimental) and usual care (control) with high risk of bias studies removed. 173

Sensitivity analyses (removing studies with imputed results)....... 174

Figure 60. Random effects meta-analysis of the mean difference in length of stay between multimodal interventions (experimental) and usual care (control) with imputed results removed. 174

Subgroup analyses (type of surgery) 175

ORTHOPAEDIC SURGERY 175

Figure 61. Random effects meta-analysis of the mean difference in length of hospital stay (LoS) between multimodal (experimental) and usual care (control) for those undergoing orthopaedic surgery. 175

Subgroup analyses (Cancer surgery) 175

Figure 62. Random effects meta-analysis of the risk ratio of mortality between multimodal (experimental) and usual care (control) for those undergoing cancer surgery. 175

Figure 63. Random effects meta-analysis of the mean difference in length of hospital stay (LoS) between multimodal interventions (experimental) and usual care (control) for those undergoing cancer surgery. 176

Subgroup analysis (studies published before and after 2010) 176

Figure 64. Random effects meta-analysis of the risk ratio of mortality between multimodal (experimental) and usual care (control) in studies published after 2010. 176

Figure 65. Random effects meta-analysis of the mean difference in length of hospital stay (LoS) between multimodal (experimental) and usual care (control) in studies published after 2010. 177

Figure 66. Random effects meta-analysis of the mean difference in length of hospital stay (LoS) between inspiratory muscle training (experimental) and usual care (control) in studies published before 2010. 177

References 177

EDUCATIONAL INTERVENTIONS 180

Table 21. Characteristics of studies 180

Table 22. Results 188

Table 23. Risk of bias 195

Table 24. Summary of findings 196

Meta analyses 197

Figure 67. Random effects meta-analysis of the risk ratio of mortality between education (experimental) and usual care (control). 197

Figure 68. Random effects meta-analysis of the mean difference in length of hospital stay (LoS) between education interventions (experimental) and usual care (control)............. 197

Sensitivity analyses (removing studies at high risk of bias).. 198

Figure 69. Random effects meta-analysis of the mean difference in length of stay between education (experimental) and usual care (control) with high risk of bias studies removed. 198

Sensitivity analyses (removing studies with imputed results) 198

Figure 70. Random effects meta-analysis of the mean difference in length of stay between education interventions (experimental) and usual care (control) with imputed results removed. 198

Subgroup analysis (studies published before and after 2010).. 199

Figure 71. Random effects meta-analysis of the mean difference in length of hospital stay (LoS) between inspiratory muscle training (experimental) and usual care (control) in studies published after 2010. 199

References 199

PSYCHOLOGICAL INTERVENTIONS 201

Table 25. Characteristics of Studies 201

Table 26. Results 210

Table 27. Risk of bias.. 216

Table 28. Summary of findings 217

Meta analyses 218

Figure 72. Random effects meta-analysis of the mean difference in length of hospital stay (LoS) between psychological interventions (experimental) and usual care (control). 218

Sensitivity analyses (removing studies at high risk of bias).. 218

Figure 73. Random effects meta-analysis of the mean difference in length of stay between psychological interventions (experimental) and usual care (control) with high risk of bias studies removed. 218

Sensitivity analyses (removing studies with imputed results).. 218

Subgroup analysis (studies published before and after 2010).. 219

Figure 74. Random effects meta-analysis of the mean difference in length of hospital stay (LoS) between psychological interventions (experimental) and usual care (control) in studies published after 2010. 219

References 219

SMOKING/ALCOHOL CESSATION INTERVENTIONS 221

Table 29. Characteristics of studies 221

Table 30. Results 227

Table 31. Risk of bias.. 232

Table 32. Summary of findings 233

Meta analyses 234

Figure 75. Random effects meta-analysis of the risk ratio of wound infection between smoking cessation interventions (experimental) and usual care (control) 234

Sensitivity analyses (removing studies at high risk of bias).. 234

Sensitivity analyses (removing studies with imputed results).. 234

SMOKING CESSATION 234
ALCOHOL CESSATION 234
Subgroup Analyses 234
References 234
PHARMACOLOGICAL INTERVENTIONS 236
Figure 76. Characteristics of studies 236
Figure 77. Results 238
Figure 78. Risk of bias 240
Meta analyses 241
References 241
Funnel plots 242
Figure 79. Funnel Plot (Immunonutrition studies) 242
Figure 80. Funnel Plot (Exercise studies) 243
Figure 81. Funnel plot (Multimodal studies) 243
Figure 82. Funnel Plot (Education studies) 244
Figure 83. Figure Funnel Plot (IMT studies) 244
Table 33. Table of excluded studies 245
Table 34. Table of studies that fit inclusion criteria but have no usable data reported 252
Table 35. ClinicalTrials.gov: PROTOCOLS (searched 14/11/19) 266
Table 36. Protocols from searches 269
Table 37. Guidance for assessing Risk of Bias in the Prehabilitation systematic review (RCTs) . 270
Table 38. Sensitivity analyses: fixed versus random effects for all outcomes 277

Search strategy

Database: MEDLINE In-process - Current week, MEDLINE 1950 to present

1 (pre-hab$ or prehab$).ti,ab. (119)
2 ((presurg$ or preoperativ$ or pre-surg$ or pre-operativ$) adj3 (conditioning or optimis$ or optimiz$ or rehab$ or re-hab$ or care)).ti,ab. (1790)
3 (pre adj2 (postsurg$ or postoperativ$) adj3 (conditioning or optimis$ or optimiz$ or rehab$ or re-hab$ or care)).ti,ab. (406)
4 ((before or prior to) adj3 (CABG or surgery or surgical or procedure$ or arthroplast$ or hip replacement or knee replacement or joint replacement or total hip or total knee or total joint$ or operation) adj12 (conditioning or optimis$ or optimiz$ or rehab$ or re-hab$ or care)).ti,ab. (1137)
5 Preoperative Care/mt, rh [Methods, Rehabilitation] (9939)
6 (preoperative care/ or preoperative period/) and (conditioning or optimis$ or optimiz$ or rehab$ or re-hab$).ti,ab. (1435)
7 Postoperative Complications/pc and ((presurg$ or preoperativ$ or pre-surg$ or pre-operativ$) adj3 (assess$ or intervention$)).ti,ab. (349)
8 or/1-7 (14185)
9 preoperative care/ or preoperative period/ (54529)
10 (presurg$ or preoperativ$ or pre-surg$ or pre-operativ$).ti,ab. (224877)
11 (pre adj2 (postsurg$ or postoperativ$)).ti,ab. (12194)
12 ((before or prior to) adj3 (CABG or surgery or surgical or procedure$ or hip replacement or knee replacement or joint replacement or total hip or total knee or total joint$ or arthroplast$ or operation)).ti,ab. (83657)
13 or/9-12 (316706)
14 exp Exercise/ or exp Exercise Therapy/ (146992)
15 physical therapy modalities/ (28521)
16 physical fitness/ (22302)
17 (exercis$ or aerobic$ or swim$ or hydrotherapy or preconditioning or pre-conditioning or physical fitness or physical activit$ or physiotherap$ or physical therap$).ti,ab. (365238)
18 ((muscle or endurance or resistance or weight or strength) adj2 training).ti,ab. (13555)
19 ((function or functional capacity) adj2 (enhanc$ or improv$ or maximis$)).ti,ab. (39597)
20 Nutrition Therapy/ or exp diet therapy/ or exp diet/ or eating/ or nutritional physiological phenomena/ or elder nutritional physiological phenomena/ or nutritional requirements/ or nutritional status/ (288993)
21 exp Dietary Supplements/ or exp Food, Fortified/ (50721)
22 exp Malnutrition/dh, pc, rh, th [Diet Therapy, Prevention & Control, Rehabilitation, Therapy] (12552)
23 (diet$ or nutrition$ or malnutrition or underweight or low BMI or undernourish$ or undernutrition or malnourish$ or immunonutrition or macronutrient$ or micronutrient$ or immuno-nutrition or macro-nutrient$ or micro-nutrient$).ti,ab. (568387)
24 ((oral or food or multinutrient$ or multi-nutrient$ or multivitamin$ or iron or protein or folate or vitamin$) adj3 supplement$).ti,ab. (33003)
25 ((iron or protein or folate or vitamin$) adj3 (deficient or deficiency)).ti,ab. (47403)
26 ((fortif$ or enrich$) adj3 (food$ or feed$)).ti,ab. (3276)
27 (weight adj2 (loss or lose$ or lost or losing) adj2 (program$ or plan$)).ti,ab. (1818)
28 (fortisip or complan).ti,ab. (17)
29 Adaptation, Psychological/ (76095)
30 Cognitive Therapy/ or Psychotherapy/ or exp Mind-body therapies/ or behavior therapy/ or mindfulness/ (116265)
31 ((counselling or counseling) adj2 (session$ or therap$ or intervention$)).ti,ab. (3428)
32 mindfulness.ti,ab. (2121)
33 (CBT or ((cognitive or talking or mental health or behavio?ral) adj3 (intervention$ or therap$))).ti,ab. (27170)

34 ((education$ or psychoeducational or psychotherapeutic or psychological or psychosocial or behavio?ral or cognitive) adj3 (intervention$ or program$)).ti,ab. (67734)
35 ((anxiety or stress or fear) adj2 (manag$ or strateg$ or therap$ or reduc$)).ti,ab. (24159)
36 ((selfcare or self-care or self-help or coping) adj2 (mechanism$ or strateg$ or behavio?r$)).ti,ab. (13440)
37 Smoking Cessation/ (20803)
38 ((smoking or drug$) adj2 (cessation or stop$ or quit$ or giving up or give up)).ti,ab. (24592)
39 (nicotine replacement therapy or NRT).ti,ab. (2339)
40 (alcohol adj2 reduc$).ti,ab. (4053)
41 Blood Glucose Self-Monitoring/ (4398)
42 ((blood sugar or blood glucose or diabetes) adj2 (level or levels or control$ or monitor$)).ti,ab. (32523)
43 Anemia/dt, pc, th [Drug Therapy, Prevention & Control, Therapy] (10586)
44 ((an?emia or an?emic) adj3 (iron or prevent$ or treat$ or control$)).ti,ab. (16266)
45 ((blood pressure or BP or hypertens$) adj2 (control$ or manag$ or medication$)).ti,ab. (28359)
46 ((COPD or angina) adj2 (control$ or manag$ or medication$)).ti,ab. (2158)
47 Geriatric Assessment/ (19045)
48 ((geriatric or baseline status) adj2 assessment$).ti,ab. (2248)
49 ((optimiz$ or optimis$) adj2 medication$).ti,ab. (251)
50 (pre-existing adj2 (comorbidit$ or co-morbidit$ or chronic illness$ or chronic disease$ or chronic condition$)).ti,ab. (233)
51 or/14-50 (1543244)
52 8 or (13 and 51) (32833)
53 letter/ (867912)
54 editorial/ (371347)
55 news/ (167087)
56 exp historical article/ (328799)
57 Anecdotes as topic/ (4603)
58 comment/ (615244)
59 case report/ (1715831)
60 (letter or comment$).ti. (101137)
61 or/53-60 (3438065)
62 randomized controlled trial/ or Randomized Controlled Trials as Topic/ or random$.ti,ab. (896854)
63 61 not 62 (3406560)
64 animals/ not humans/ (3906384)
65 exp Animals, Laboratory/ (735772)
66 exp Animal Experimentation/ (6519)
67 exp Models, Animal/ (428521)
68 exp rodentia/ (2704363)
69 (rat or rats or mouse or mice or animal or animals).ti. (1219737)
70 or/63-69 (7953941)
71 52 not 70 (28469)
72 (exp child/ or exp infant/) not adult/ (1398640)
73 ((child$ or infant$ or newborn$ or neonat$) not adult$).ti. (826483)
74 72 or 73 (1610061)
75 71 not 74 (26156)
76 meta-analysis/ (53771)
77 meta-analysis as topic/ (14025)
78 (meta analy$ or metaanaly$ or metanaly$ or meta regression).ti,ab. (73844)
79 ((systematic$ or evidence$) adj2 (review$ or overview$)).ti,ab. or review.ti. (315502)
80 (reference list$ or bibliograph$ or hand search$ or manual search$ or relevant journals).ab. (27398)

81 (search strategy or search criteria or systematic search or study selection or data extraction).ab. (29241)
82 (search$ adj4 literature).ab. (31134)
83 (medline or pubmed or cochrane or embase or psychlit or psyclit or psychinfo or cinahl or science citation index or bids or cancerlit).ab. (96758)
84 cochrane.jw. (11169)
85 ((multiple treatment$ or indirect or mixed) adj2 comparison).ti,ab. (1006)
86 or/76-85 (435094)
87 randomized controlled trial.pt. or randomized controlled trial/ or Randomized Controlled Trials as Topic/ (477876)
88 controlled clinical trial.pt. (88869)
89 ((doubl$ or singl$ or trebl$ or tripl$) adj blind$).ti,ab. (130373)
90 random$.ti,ab. (749676)
91 clinical trials as topic.sh. (171370)
92 trial.ti. (134197)
93 (controlled adj clinical trial).ti,ab. (9292)
94 or/87-93 (1143831)
95 75 and (86 or 94) (4843)

Characteristics of studies and results

NUTRITIONAL INTERVENTIONS

Table 1. Characteristics of studies

ID	1st Author, year and country	Total *n* Intervention (I) Control (c)<br><br>Number analysed (An)if reported	Patient population, baseline clinical characteristics (mean (SD) or *n* (%) unless otherwise stated	Demographics (mean (SD) or *n* (%) unless otherwise stated)	Intervention	Comparator	Mode of delivery; place of delivery; training level of individuals who delivered the intervention; the number of contacts	Intervention fidelity; Compliance or adherence to intervention
**NUTRITIONAL/DIETARY INTERVENTION**								
**ONS**								
1	Burden et al., 2011 UK	125 ppts with colorectal cancer undergoing colorectal surgery.<br><br>I=59 (54An): C=66 (62An)	**BMI kg/m²**<br>I=25.0(4.8)<br>C=26.8(4.7)<br>**Severity of illness**<br>**TNM stage 1/2/3/4**<br>I=4/14/20/3<br>C=6/22/14/3	**Based on analysed ppts**<br>**Age yrs**<br>I=64.5(13.9)<br>C=65.3(2.7)<br>**Gender (%M)**<br>I=63:C=61<br>**Smokers**<br>I=10:C=13	ONS:<br>Milk-based supplement (630kJ and 6g protein/100mL) - Fortisip, or if not tolerated, fruit juice (630kJ and 4g protein/100mL) - Fortijuce 2 cartons (400mL) daily<br>Ppts started the supplements at time of enrolment up surgery	Dietary advice only	Self-delivery (oral), Dietician instructed;<br>Self-administered (at home);<br>Dietician;<br>NR	NR;<br>36 (72%) managed 100% of the supplement, 8 (16%) managed 50%, 6 (12%) managed <25%
2	Burden et al., 2017 UK	101 weight-losing ppts with colorectal cancer<br>I=55 (55ITT): C=46 (45ITT)	**BMI kg/m²**<br>I=25.5(4.6)<br>C=25.9.8(4.8)<br>Reported unintentional weight loss >1kg /3–6 mths<br>**% weight loss (prior to I)**<br>**Median (IQR)**<br>I=4.90(2.2–8.8)<br>C=6.8(3.4–12.1)<br>**Cancer stage (N) 1/2/3/4**<br>I=2/9/26/11<br>C=1/4/26/12<br>**No. of comorbidities**<br>0: I=15:C=6	**Age yrs**<br>I=70.5(11.66)<br>C=68.9(11.49)<br>**Gender (%M)**<br>I=64:C=70<br>**Smoking status (%)**<br>Never<br>I=49:C=26<br>Ex-smoker<br>I=33:C=45<br>Current<br>I=18:C=20<br>Missing data<br>I=0:C=9	ONS:<br>Oral feed: 2 cartons/day (250mL/day ONS: 10.1kJ and 0.096g protein/mL) + dietary advice (aimed at increasing energy and protein intake by increasing high-fat, sugar and protein rich foods and encouraging purchase of high-street dietary supplements) for a median of 8 days (IQR 5–15) before surgery.	Dietary advice (oral and written) only	Self-delivery (oral) (oral and written);<br>Self-administered (at home);<br>Nutritionist;<br>NR (advice was given at BL. Seen again for data collection 24-48hrs preop – but unclear if dietary advice given)	NR:<br>39 (74%) returned a diary detailing their adherence to the ONS.<br>29 (74%) ppts drank all the supplements (2 cartons daily), 2 (5%) ppts drank 1.5 cartons, 3 (8%) ppts drank 1 carton, 3 (8%) drank 0.5 carton, 2 (5%) ppts did not consume any of the ONS.<br>**Intolerance of ONS**<br>7=did not follow the ONS regimen;

ID	1st Author, year and country	Total *n* Intervention (I) Control (c)<br><br>Number analysed (An)if reported	Patient population, baseline clinical characteristics (mean (SD) or *n* (%) unless otherwise stated	Demographics (mean (SD) or *n* (%) unless otherwise stated)	Intervention	Comparator	Mode of delivery; place of delivery; training level of individuals who delivered the intervention; the number of contacts	Intervention fidelity; Compliance or adherence to intervention
**NUTRITIONAL/DIETARY INTERVENTION**								
			1: I=8:C=7<br>I=9:C=12<br>3: I=9:C=4<br>4+: I=8:C=8<br>Missing data<br>I=6:C=9					
3	Kikuchi et al., 2016 Japan	77 elective liver resections for hepatocellular carcinoma<br>I=39:C=38	**Hepatitis C**<br>I=8:C=14<br>**Hepatitis B**<br>I=18:C=11<br>**Non B/non C**<br>I=13:C=13<br>**Background liver fibrosis**<br>FO: I=3:C=2<br>F1-3: I=24:C=21<br>F4: I=12:C=15	**Age yrs**<br>I=69.4(7.5)<br>C=71.9(7.4)<br>**Gender (%M)**<br>I=79:C=16	Oral feed:<br>Livact: Preoperative oral supplementation with Branched-chain amino acid (BCAA) granules, 3 x day for up to 1 mth	Standard diet	Self-delivery (oral), NR how info was relayed;<br>Home;<br>Self-delivered;<br>Followed up regularly (not fully described)	NR;<br>NR
4	MacFie et al., 2000 UK	100 ppts undergoing elective major gastrointestinal surgery<br><br>Data included for group 2 & 4 only<br>(group 1 & 3 intervention continues PO)<br><br>I=24:C=25	**BMI kg/m^2 on entry into study**<br><19: I=3:C=0<br>19-25: I=16:C=18<br>≥ 26: I=5:C=7<br>**Preop BMI kgm^2 median (range)**<br>I=23(15-31)<br>C=25(21-35)<br>**POSSUM score mean (range)**<br>I=17.3(10-35)<br>C=17.6(11-27)<br>**Surgical procedures**<br>**Colorectal**<br>I=20:C=21<br>**GI**<br>I=3:C=3<br>**Hepatobiliary**<br>I=1:C=1	**Age yrs (range)**<br>I=68(23-84)<br>C=48(42-85)<br>**Gender (%M)**<br>I=44:C=48	ONS:<br>Min. of 2 cartons of oral dietary supplements daily (no duration stated)	No supplements	Self-delivery (oral), NR how info was relayed<br>Self-administered at home;<br>Experienced professional (specialist nutrition nurse);<br>None	NR – although range of 'feeding days' was large. Preoperatively 15 days (mean), range 5-59 days;<br>Preop supplements (kcal/day)<br>I=536 (22):C=0<br>Voluntary food intake (kcal/day)<br>I=1198 (range: 644-2022):C=1315 (range: 605-2215)<br>% energy from supplements<br>I=37:C=0

ID	1st Author, year and country	Total *n* Intervention (I) Control (c)<br><br>Number analysed (An)if reported	Patient population, baseline clinical characteristics (mean (SD) or *n* (%) unless otherwise stated	Demographics (mean (SD) or *n* (%) unless otherwise stated)	Intervention	Comparator	Mode of delivery; place of delivery; training level of individuals who delivered the intervention; the number of contacts	Intervention fidelity; Compliance or adherence to intervention
**NUTRITIONAL/DIETARY INTERVENTION**								
5	Nagata et al., 2013<br>Japan<br>Pilot Study	23 ppts undergoing hepatectomy<br>I=11:C=12	Living healthy liver donors	**Age yrs**<br>I=38.5(11.9)<br>C=21.4(7.9)<br>**Overall gender (%M)**<br>I=75:C=64	ONS:<br>Nutritional supplement:1400ml liquid diet/day, every day for 5 days preop	Standard food intake	Self-delivery (oral), NR how info was relayed;<br>Outpatients;<br>NR;<br>NR	NR;<br>Stated that all ppts adhered to protocol
6	Smedley et al., 2004<br>UK	98 ppts undergoing lower GI tract surgery<br>I (SC)=48 (41An):<br>C (CC)=50 (44An)<br>+2 groups not relevant to this review	**BMI kg/m^2**<br>I=26.9(4.9)<br>C=27.8(5.6)<br>**Diagnosis (N)**<br>**Colonic or rectal cancer**<br>I=31:C=35<br>**Colitis**<br>I=7:C=6<br>**Diverticulosis**<br>I=5:C=3<br>**Other**<br>I=5:C=6	**Age**<br>(N:I=48:C=50<br>I=61(23-84)<br>C=63(25-88)<br>**Gender (%M)**<br>I=69:C=56	ONS:<br>Fortisip drink containing 1·5 kcal and 0·05g protein/ml, to drinks *ad libitum* in small, frequent quantities between meals.<br>Min. 7 days (Mean=15 days)	No supplement	Self-delivery (oral), NR how info was relayed;<br>Self-administered at home and hospital;<br>NR;<br>NR	NR;<br>NR
7	Zhao et al., 2018<br>China	66 pts with Adenocarcinoma of esophagogastric junction<br>I= 33:C=33	**NR**	**Age, range**<br>26–74 yrs (average 62 yrs)	ONS:<br>7 days of Nutrison fibre (500ml) 500kcal, protein 20 g, fat 19.45 g, carbohydrate 61.5 g. in addition to routine preoperative diet.	Routine preoperative diet (35 kcal/kg/d) according to dietary guidance	Self-delivery (oral), NR how info was relayed;<br>NR<br>NR	NR;<br>NR
**IMMUNONUTRITION**								
8	Aida et al., 2014<br>Japan	50 ppts undergoing pancreatoduodenectomy or pylorus-preserving pancreatoduodenectomy<br><br>I=25:C=25	**ASA score 1/2/3/4/5**<br>I=10/8/7/0/0<br>C=11/10/4//0/0<br>**Diabetes**<br>I=8:C=5<br>**Jaundice**<br>I=14:C=15<br>**Diagnosis**<br>**Pancreatic carcinoma**:	**Age yrs**<br>I=66.4(1.5)<br>C=65.1(1.9)<br>**Gender (%M)**<br>I=80:C=64	Immunonutrition:<br>Oral nutrition formula containing arginine, omega-3 fatty acids, and RNA for 5 days before surgery.<br>+ 50% reduction in the amount of regular food (1,000 kcal/day).	No supplementation.<br>Allowed to consume regular food before surgery (2,000 kcal/day).	Self-delivery (oral), NR how info was relayed;<br>Self-administered at home;<br>NR;<br>None	NR;<br>All ppts were able to drink more than 90% of the indicated amount.

ID	1st Author, year and country	Total *n* Intervention (I) Control (c)<br><br>Number analysed (An)if reported	Patient population, baseline clinical characteristics (mean (SD) or *n* (%) unless otherwise stated	Demographics (mean (SD) or *n* (%) unless otherwise stated)	Intervention	Comparator	Mode of delivery; place of delivery; training level of individuals who delivered the intervention; the number of contacts	Intervention fidelity; Compliance or adherence to intervention
**NUTRITIONAL/DIETARY INTERVENTION**								
		N analysed not stated.	I=14:C=9<br>**Bile duct carcinoma**:<br>I=4:C=5<br>**Ampullary carcinoma:**<br>I=3:C=5<br>**Others**:<br>I=4:C=5<br>**Pathological tumour-node-metastasis stage 0/I/II/III/IV**<br>I=2/3/10/5/1<br>C=0/3/9/7/1<br>**Surgical procedure (Whipple /PpPD)**<br>I=20/5:C=19/6					
9	Barker et al., 2013<br>Australia	95 ppts undergoing major elective upper or lower GI surgery.<br>I=46:C=49	**Well nourished (SGA-A)**<br>I=35:C=40<br>**Malnourished (SGA-B/C)**<br>I=11:C=9	**Age yrs**<br>I=61(13)<br>C=65(15)<br>**Gender (%M)**<br>I=37:C=63	**I**mmunonutrition:<br>Impact Advanced Recovery (Nestle): to drink 3x 237ml packs/day for 5 days before surgery, or commencement of bowel preparation	No supplementation	Self-delivery (oral), NR how info was relayed;<br>Self-administered at home;<br>NR;<br>NR	NR;<br>Compliance was 91%: 32 ppts consumed all 3 supplements and 7 failed to consume 3 daily
10	Braga et al., 2002<br>Italy	200 ppts with colorectal cancer randomized into 4 groups.<br>Data extracted from<br>I=50:C=50	**ASA score**<br>I=1.9(1.1)<br>C=2.1(1.4)	**Age yrs**<br>I=63.0(8.1)<br>C=61.8(9.9)<br>**Gender (%M)**<br>I=60:C=62	Immunonutrition:<br>1L enriched formula Oral Impact: arginine (12.5g/L) omega-3 fatty acids (3.3 g/L)	Usual care	Self-delivery (oral), NR how info was relayed;<br>Home, hospital and 2 days out-patient therapy;<br>NR;<br>NR	NR;<br>Mean daily preoperative intake of the formula was 905 mL
11	Desai et al., 2016<br>India<br>Abstract only	80 ppts undergoing major cardiac surgery<br>I=40:C=40	NR	NR	Immunonutrtion<br>20g of oral L-Glutamine supplement<br>5 days preoperatively	Observation only	Oral<br>NR how info was relayed<br>NR<br>NR	NR;<br>NR

ID	1st Author, year and country	Total *n* Intervention (I) Control (c)<br><br>Number analysed (An)if reported	Patient population, baseline clinical characteristics (mean (SD) or *n* (%) unless otherwise stated	Demographics (mean (SD) or *n* (%) unless otherwise stated)	Intervention	Comparator	Mode of delivery; place of delivery; training level of individuals who delivered the intervention; the number of contacts	Intervention fidelity; Compliance or adherence to intervention
**NUTRITIONAL/DIETARY INTERVENTION**								
12	Fujitani et al., 2012 Japan	244 ppts with primary gastric cancer undergoing total gastrectomy<br>I=127 (120An):<br>C=117 (111 An)	**BMI kg/m^2 median (range)**<br>I=22·8(15·1–33·8)<br>C=22·6(17·8–33·1)<br>**Nutritional status (N)**<br>Well-nourished<br>I=123:C=116<br>Malnourished<br>I=4:C=1<br>**Tumour status**<br>T1: I=44:C=42<br>T2: I=36:C=37<br>T3: I=38:C=24<br>T4: I=5:C=8<br>Node status also reported	**Age yrs median (range)**<br>I=64(26–78)<br>C=65(30–79)<br>**Gender (%M)**<br>I=76:C=72	Immunonutrition:<br>1000 ml/day of preoperative oral supplementation: immunonutrient-enriched enteral feed (Impact) added to normal diet for 5 consecutive days before surgery (never through tube)	Regular diet without any nutritional supplementation	Self-delivery (oral), NR how info was relayed;<br>NR;<br>NR;<br>NR	NR;<br>Compliance with oral Impact was 91·7, 95·2, 96·6, 96·6 and 92·3% of planned volume over the 5 days before surgery, with an overall rate of adherence reported as 94·5%
13	Gade et al., 2016 Denmark	46 ppts undergoing surgery for pancreatic cancer<br>I=25 (19An):<br>C=21 (16An)<br><br>11 excluded at secondary assessment	**BMI kg/m^2 median (range)**<br>I=24.3(18.8-28.3)<br>C=23.8(18.1–30.8)<br>**Nutritional Risk Screening (%)**<br>I=32:C=50<br>**Comorbidities: Diabetes (%)**<br>I=32:C=19<br>**Cardiopulmonary** (%)<br>I=37:C=38<br>**Other** (%)(osteoporosis, previous cancer, stomach ulcers, benign polyps, metabolic disorder, fibromyalgia):	**Age yrs** median (range)<br>I=68(50-81)<br>C=69(53–79)<br>**Gender** (N:<br>I=19:C=16)<br>**(%M**)<br>I=63:C=37.5<br>**Smokers %**<br>I=32:C=25<br>**Smoking cessation <4 wks**<br>I=5:C=6	Immunonutrition:<br>7 days of preoperative oral immunonutrition (IN), Oral Impact Powder® (Nestle) as a supplement to normal diet to reach a total goal of 1.5 g protein/kg body weight. The ppts' habitual protein intake was estimated based on a Food Frequency questionnaire focusing on the past wk scheduled to end on the day before surgery.<br><br>The IN powder was diluted with 250 ml of water; each package contained 16.8 g protein. Ppts were offered three flavors (coffee, citrus, and tropical), and instructed to consume the product between meals	Received the standard of care in the department. This includes routine nutritional screening for disease-related malnutrition using NRS-2002, individual advice by nurses on intake of nutritional supplements in case of malnutrition and by a dietician prior to discharge.	Self-delivery (oral), NR how info was relayed;<br>NR;<br>NR;<br>NR	NR;<br>Ppts were requested to record their compliance with the prescribed dosage of IN using a provided diary.<br><br>Ppts were asked to hand in any excess product at admission to the hospital on the day before surgery. Compliance was defined as those who had consumed 75% of their provided dosage of IN. The overall compliance with the IN supplement ranged from 0 to 100% with a median compliance of 100%.<br><br>The reported median intake was 2 packages/day, and 13/

Perry R, *et al. BMJ Open* 2022; 11:e050806. doi: 10.1136/bmjopen-2021-050806

ID	1st Author, year and country	Total *n* Intervention (I) Control (c)<br><br>Number analysed (An)if reported	Patient population, baseline clinical characteristics (mean (SD) or *n* (%) unless otherwise stated	Demographics (mean (SD) or *n* (%) unless otherwise stated)	Intervention	Comparator	Mode of delivery; place of delivery; training level of individuals who delivered the intervention; the number of contacts	Intervention fidelity; Compliance or adherence to intervention
**NUTRITIONAL/DIETARY INTERVENTION**								
			I=16:C=31<br>None: I=16:C=13					19 ppts had an intake ≥75% of their calculated dosage.<br><br>The IN supplement was generally well accepted with only 1 diabetic ppt reporting an adverse event of fluctuating blood sugar levels.
14	Gianotti et al., 2002 Italy	305 ppts with GI cancer<br>I=102:C=102<br><br>(3rd arm, n=101 received perioperative nutrition; NR here)	Excluded ppts with weight loss ≥10% in past 6 mths, <18yrs, hepatic, respiratory. renal, and cardiac dysfunction, low performance status, pregnancy, ongoing infections and immune disorder<br>**BMI kg/m2**<br>I=24.5(4.9):C=23.8 (4.1)<br>**Hypertension**<br>I=16:C=22<br>**Diabetes**<br>I=6:C=5<br>**Arrhythmia**<br>I=7:C=4<br>**Liver disease**<br>I=3:C=5<br>**GI tract disease**<br>I=3:C=2<br>**Cardiac disease**<br>I=2:C=2<br>**Central nervous system disease**<br>I=1:C=2<br>**Miscellaneous**	**Age**<br>I=62.3(12.3)<br>C=63.4(11.9)<br>**Gender (%M)**<br>I=49:C=55	Immunonutrition:<br>Oral supplementation for 5 days before surgery with 1 L/day of Impact (with arginine, omega-3 fatty acids, and RNA), with no nutritional support given after surgery<br><br>Group 2 received enteral diet through a tube (NR to our review)	No artificial nutrition before and after surgery	Self-delivery (oral), NR how info was relayed;<br>Outpatients;<br>NR;<br>NR	NR;<br>NR

ID	1st Author, year and country	Total *n* Intervention (I) Control (c)<br><br>Number analysed (An)if reported	Patient population, baseline clinical characteristics (mean (SD) or *n* (%) unless otherwise stated	Demographics (mean (SD) or *n* (%) unless otherwise stated)	Intervention	Comparator	Mode of delivery; place of delivery; training level of individuals who delivered the intervention; the number of contacts	Intervention fidelity; Compliance or adherence to intervention
**NUTRITIONAL/DIETARY INTERVENTION**								
			I=7:C=3					
15	Gunerhan et al., 2009 Turkey	42 ppts undergoing surgery for gastrointestinal tumors<br>I=16<br>C(NN)=13<br>C(SE)=13<br><br>NB: 56 were recruited, 14 excluded and 42 completed	**BMI kg/m^2**<br>I=24.1(3.7)<br>C(NN)=23.0(5.7)<br>C(SE)=22.2(4.9)<br>**Malnutrition index**<br>I=8.0(1.4)<br>C(NN)=7.3(0.8)<br>C(SE)=8.4(1.7)<br>**Muscle circumference**<br>I=22.3(3.0)<br>C(NN)=21.1(4)<br>C(SE)=20.4(2.3)<br>**Hand grip strength**<br>I=0.5(0.2)<br>C(NN)=0.4(0.2)<br>C(SE)=0.4(0.2)<br>**Albumin**<br>I=3.6(0.6)<br>C(NN)=3.5(0.6)<br>C(SE)=3.3(0.5)<br>**Prealbumin**<br>I=13.64(8.83)<br>C(NN)=15.7(7.0)<br>C(SE)=17.7(8.5)<br>**No. of lymphocytes**<br>I=1454(462)<br>C(NN)=1281(780)<br>C(SE)=1277(546)<br>**Subjective global assessment**<br>**Moderate malnutrition**<br>I=9:C(NN)=7:<br>C(SE)=6	**Age yrs**<br>I=64.6(16.2)<br>C(NN)=64.4(11.7)<br>C(SE)=61.3(12.1)<br>**Gender (%M)**<br>I=50:C(NN)=30:<br>C(SE)=38	Immunonutrition:<br>Impact (with arginine, omega-3 fatty acids and RNA) for 7 days preop	NN (normal nutrition):<br>Normal feeding planned by a dietitian for 7 days preop (usual care)<br>**SE** (standard enteral nutrition): *Fresubin* for 7 days preop	Nutrition was administered to ppts;<br>General Surgery Unit;<br>NR;<br>NR	NR;<br>NR

ID	1st Author, year and country	Total *n* Intervention (I) Control (c)<br><br>Number analysed (An)if reported	Patient population, baseline clinical characteristics (mean (SD) or *n* (%) unless otherwise stated	Demographics (mean (SD) or *n* (%) unless otherwise stated)	Intervention	Comparator	Mode of delivery; place of delivery; training level of individuals who delivered the intervention; the number of contacts	Intervention fidelity; Compliance or adherence to intervention
**NUTRITIONAL/DIETARY INTERVENTION**								
			**Severe malnutrition**<br>I=7:C(NN)=6: C(SE)=7					
16	Kaya et al., 2016<br>Turkey	58 ppts undergoing lung cancer surgery<br>I=31:C=27	**BMI kg/m^2**<br>I=25.2(4.1)<br>C=26.7(3.5)<br>**COPD**<br>I=3:C=0<br>**hypertension**<br>I=5:C=4<br>**Coronary Artery Disease**<br>I=2:C=0<br>**Multi-nodal goiter**<br>I=1:C=2<br>**Congestive heart failure**<br>I=1:C=0<br>**Asthma**<br>I=1:C=0<br>**Ankylosing spondylitis**<br>I=0:C=1<br>**Parkinson's disease**<br>I=0:C=1<br>**Peripheral vascular disease**<br>I=0:C=1	**Age yrs**<br>I=57.8(9.7)<br>C=59.0(7.6)<br>**Gender (%M)**<br>I=94:C=93	Immunonutrition:<br>Immune-modulating formula enriched with arginine, omega 3 fatty acids and nucleotides for 10 days	Standard diet	Self-delivery (oral), NR how info was relayed;<br>NR;<br>Self-delivered;<br>NR	NR;<br>NR
17	Hossain et al., 2016<br>NR<br>Abstract only	80 ppts with gastrointestinal malignancies e.g. colon, stomach etc.<br>I= 40:C=40	**NR**	**NR**	Ppts were given nutritional plans that induced immunomodulatory activity (Impact)	Standard nutrition plans	NR;<br>NR;<br>NR;<br>NR	NR;<br>NR
18	Manzanares Campillo et al., 2017	84 ppts diagnosed with colorectal	At 1st preop assessment:	**Overall Age yrs**<br>69.9(11)	Immunonutrition:	Standard preoperative care (no immunonutrients) and normal diet.	Administration in hospital; immediately prior to surgery. Intervention delivered over 11 mths	NR;

ID	1st Author, year and country	Total *n* Intervention (I) Control (c)<br><br>Number analysed (An)if reported	Patient population, baseline clinical characteristics (mean (SD) or *n* (%) unless otherwise stated	Demographics (mean (SD) or *n* (%) unless otherwise stated)	Intervention	Comparator	Mode of delivery; place of delivery; training level of individuals who delivered the intervention; the number of contacts	Intervention fidelity; Compliance or adherence to intervention
**NUTRITIONAL/DIETARY INTERVENTION**								
	Spain (Translation)	cancer waiting surgery (intestinal resection).<br><br>I=42:C=42	**Protein malnutrition (alterations in at least 1 of: retinol-binding protein transferrin, albumin, prealbumin):** I=12 (29%)<br>C=14 (33%)<br>**Caloric malnutrition (alteration in at least 1 of: %weight loss, BMI, total cholesterol):** I=27 (64%)<br>C=24 (57%)<br>**Risk of malnutrition (scores>=3):** I=27 (64%)<br>C=27 (64)<br>**Index of nutritional risk(scores <=100):**<br>I=7(17%)<br>C=8(19%)	**Overall gender (%M)**<br>69	Preop immunonutrition and normal diet. Impact© Oral. 3 drinks of 237ml/day for 8 days.<br>Each bottle: 341kcal (18.1g proteins, 44.7g carbohydrates, 9.2g fats, 3.3g fibre, 0.43 nucleotide vitamins and oligo-elements.			Actual consumption of I drink: 7.3 (0.8) days. Mean volume consumed: 5180.1 (618.4)ml.
19	Martinez et al., 2020 Mexico	40 ppts with enterocutaneous fistula undergoing definitive surgery<br>I= 20, C=20	**ASA III or more**<br>I= 6: C= 10<br>**History of open abdomen**<br>I= 1: C=12<br>**Malnutrition**<br>I=12: C=13	**Age**<br>I= 54 (12.6):<br>C= 52 (13.5)<br>**Gender (%M)**<br>I=75:C=60	Immunonutrition:<br>Orally as a supplement, 4.5 g of arginine (Arginaid, Nestle, Sw) and 10 g of glutamine (Symptx, Merck, USA) diluted each in 125 ml of water/day.<br>& days preoperatively	Standard management	Oral, NR how info was relayed;<br>NR<br>NR<br>NR	NR;<br>NR
20	Mikagi et al., 2011	41 ppts undergoing	**BMI kg/m^2**<br>I=23.6(3.8)	**Age yrs**<br>I=67.5(11.3)	Immunonutrition:	Conventional hospital meals (1800 Kcals/day)	Self-delivery (oral), NR how info was relayed;	NR;

ID	1st Author, year and country	Total *n* Intervention (I) Control (c)<br><br>Number analysed (An)if reported	Patient population, baseline clinical characteristics (mean (SD) or *n* (%) unless otherwise stated	Demographics (mean (SD) or *n* (%) unless otherwise stated)	Intervention	Comparator	Mode of delivery; place of delivery; training level of individuals who delivered the intervention; the number of contacts	Intervention fidelity; Compliance or adherence to intervention
**NUTRITIONAL/DIETARY INTERVENTION**								
	Japan	segmentectomy or extensive hepatectomy<br><br>I=25 (13An):<br>C=16 (13An)	C=21.5(4.4)	C=61.5(10.2)<br>**Gender (%M)**<br>I=77:C=62	750 ml of IMPACT + half-size hospital meals (1000 kcal/day) from 5 days up to surgery (in hospital care)		In hospital;<br>NR;<br>NR	Compliance: 43% were lost from the study.
21	Nakamura et al., 2005<br>Japan	26 ppts undergoing major abdominal surgery<br>I=12:C=14	Ppts with BMI 16-30 kg/m^2 undergoing surgery for bile duct cancer (n=10), pancreatic cancer (n=7), gastric cancer (n=5), or oesophageal cancer (n=4). Ppts with severe comorbidities excluded.	**Age yrs**<br>I=64(10)<br>C=64(15)<br>**Gender (%M)**<br>I=92:C=79	Immunonutrition:<br>Preoperative oral nutritional supplement (Impact), containing (per 100mL): 1.28g of free L-arginine, 0.13g of RNA, 2.80g of total lipids, 0.40g of n-3 fatty acids, 0.33g of n-6 fatty acids, 1.10g of nitrogen , and 13.4g of carbohydrate, minerals, and vitamins). Asked to consume 1L/d for 5 days before surgery<br><br>After surgery, all ppts received total parenteral nutrition (TPN) and enteral nutrition (EN).	Normal diet without Impact<br><br>After surgery, all ppts received total parenteral nutrition (TPN) and enteral nutrition (EN).	Self-delivery (oral), NR how info was relayed;<br>Assumed hospital (due to compliance checking);<br>NR;<br>NR but assume no FU as intervention was short	NR;<br>Compliance during intervention period ensured and checked by doctors and nurses and recorded in food diaries
22	Pronio et al., 2008<br>Italy<br>(Translation)	153 ppts undergoing major abdominal surgery were screened for malnutrition. 43 ppts identified as having mild malnutrition were randomized<br>I=22:C=21	**Cardiovascular disease %**<br>I=23:C=5<br>**DM %**<br>I=5:C=5<br>**COPD %**<br>I=23:C=20<br>**Hypertension %**<br>I=14%:C=38%	**Age** Mean/SDs not given, but % people >75 yrs<br>I=14: C=19<br>**Gender (%M)**<br>I=64:C=48	Immunonutrition:<br>Impact Oral®: 3 packs of 80 gr/d dissolved in water for 7-10 days before surgery.	Usual care (no nutritional support)	NR but assume self-delivery at home.	NR;<br>NR.
23	Russell et al., 2019<br>New Zealand	34 ppts scheduled for non-laparoscopic elective hepatic resection for	**ASA (I/II/III)**<br>Intervention: 1/0/6<br>Control: 0/8/6<br><br>**SGA (A/B/C)**	**Age Median and range**<br>I= 61 (28-76)<br>C= 63 (31-79)<br>**Gender (%M)**<br>I=65:C=67	Immunonutrition:<br>In addition to their usual intake, IMN ppts were prescribed for each of the 5 consecutive days preceding surgery 3 x 237 mL tetra packs of IMPACT Advanced	STD group were advised to continue with their usual oral intake. Ppts in this group assessed as having malnutrition were provided with a standard nutritional	Oral, NR how info was relayed;<br>At home;<br>NR;<br>Phoned on pre-op day 5 to remind them to start taking the intervention	NR<br>Compliance with the full preoperative course of immunonutrition was 100% in 16/17 ppts with 1 ppt

ID	1st Author, year and country	Total *n* Intervention (I) Control (c)<br><br>Number analysed (An)if reported	Patient population, baseline clinical characteristics (mean (SD) or *n* (%) unless otherwise stated	Demographics (mean (SD) or *n* (%) unless otherwise stated)	Intervention	Comparator	Mode of delivery; place of delivery; training level of individuals who delivered the intervention; the number of contacts	Intervention fidelity; Compliance or adherence to intervention
**NUTRITIONAL/DIETARY INTERVENTION**								
		primary or secondary liver cancer<br>I= 17:C=17	Intervention: 15/1/1<br>Control: 13/2/0		Recovery® (Nestle) providing 1020 kcal energy, 54 g protein, 12.6 g arginine, 1.3 g nucleotides, and 3.3 g eicosapentaenoic acid (EPA) + docosahexaenoic acid (DHA) per day.	supplement (Fortisip®, Nutricia) twice daily (providing 600 kcal energy, 24 g protein), in addition to their usual intake, for the period preceding and including 5 days prior to surgery.		consuming 1 less tetra pack than prescribed.
24	Sufit et al., 2012<br>USA	14 ppts undergoing elective cardiac surgery requiring cardiopulmonary bypass<br>I=7 (4 completed):<br>C=7 (6 completed)	NR	**Age yrs**<br>I=65.3(9.0)<br>C=56.7(8.2)<br>**Gender (%M)**<br>(N=10)<br>I=100:C=67	Immunonutrition:<br>Oral alanyl-glutamine (GLN) group: 50g of oral alanyl-GLN dipeptide 25g 2x day.<br>3 days prior to surgery	Maltodextrin 25g 2x day	Self-delivery (oral),<br>Self-administered at home & hospital;<br>Study nurse;<br>NR	NR;<br>Compliance assessed by nurse calls, empty packet returns and self-reporting – noted as high
25	Tumas et al., 2020<br>Lithuania	92 ppts with suspected pancreatic cancer<br>I=40:C= 52	NR	**Ag, yrse**<br>I=62.6 (10.5)<br>C=63.0 (8.7)<br>**Gender (%M)**<br>I=57:C=50	Immunonutrition:<br>5 days of preoperative immunonutrition (L-arginine 6.04 g/day and polyunsaturated fat 4 g/day) in addition to the usual preoperative nutritional management.	Routine preoperative nutritional management only. Routine perioperative nutritional management included preoperative nutritional screening (NRS-2002) and supplementation with standard normocaloric formula for those at high nutritional risk (NRS ≥ 3) up to 5 days	Oral, NR how info was relayed;<br>NR;<br>NR;<br>NR	NR<br>NR
26	Yoshitomi et al., 2009<br>NR<br>Abstract only	14 ppts undergoing pancreatoduode nectomy or pancreatic body/tail resection<br>I= 6:C= 8	No difference in background variables (age, sex, operation time, blood loss volume).	NR	Impact (750 ml/day) for 5 preoperative days	NR<br>Assume standard care	Oral, NR how info was relayed;<br>NR;<br>NR;<br>NR	NR;<br>NR

ID	1st Author, year and country	Total *n* Intervention (I) Control (c)<br><br>Number analysed (An)if reported	Patient population, baseline clinical characteristics (mean (SD) or *n* (%) unless otherwise stated	Demographics (mean (SD) or *n* (%) unless otherwise stated)	Intervention	Comparator	Mode of delivery; place of delivery; training level of individuals who delivered the intervention; the number of contacts	Intervention fidelity; Compliance or adherence to intervention
**NUTRITIONAL/DIETARY INTERVENTION**								
**Weight loss intervention**								
27	Alami et al., 2007<br>USA	100 ppts undergoing bariatric surgery for weight loss.<br><br>I=50 (26An):<br>C=50 (35An)	**Initial BMI (kg/m^2)**<br>I=48.7(6.6)<br>C=49.3(6.4)<br>**Hypertension (%)**<br>I=66.7:C=57.1<br>**Diabetes (%)**<br>I=51.2:C=45.7<br>**Sleep apnea (%)**<br>I=37:C=40<br>**Previous surgery (%)**<br>I=59.3:C=54.3<br>**Average number of co-morbidities**<br>I=4.5(2.1)<br>C=4.5(2.2)	**Age yrs**<br>I=42.4(10.5)<br>C=44.9(7.8)<br>**Gender (%M)**<br>I=12:C=20	PPts had to demonstrate 10% weight loss before scheduled surgery. Encouraged to concentrate on diets that worked well in the past but allowed to use any means necessary.	Routine preoperative workup without need to demonstrate 10% weight loss.	Ppt-led weight loss;<br>Ppt-led at home;<br>Experienced professional (Ppts had access to nutritionist if required);<br>Ppts seen preoperatively at least 3x.	NR:<br>Weight loss achieved in I group averaged 8.2% loss (range 1.3 – 15.9);<br><br>Retention of 61%: only 61/100 ppts had surgery
28	Barth et al., 2019<br>USA	63 ppts undergoing partial hepatectomy<br>I=31 (30An):<br>C=32 (30An)	**BMI, kg/m^2**<br>I=32.9(6.3)<br>C=32.1(6.5)<br>**Diabetes M (%)**<br>I=17:C=17<br>**Cirrhosis (%)**<br>I=0:C=3	**Age yrs**<br>I=58(12)<br>C=54(12)<br>**Gender (%M)**<br>I=47:C=63	ONS:<br>5 units of *Optifast* 800 (Nestle) + unlimited calorie-free fluids/day. (800 kcal, 20g fat, 70g protein, 100g carbohydrates and meets/exceeds the Dietary Reference Intakes for all vitamins and minerals).<br>A wk supply of *Optifast* was provided. If could not comply, to take an alternative food diet equivalent.<br>For 1 wk before hepatic resection.	No specified diet	Self-delivery (oral);<br>Home based;<br>Study dietitian;<br>Study dietitian made 2+ phone calls	NR;<br>I= 28 of the 30 ppts (94%) were compliant. 1 ppt did not start the diet, and 1 ppt consumed substantially more carbohydrates and fat than specified by the diet. 26 ppts (93%) consumed solely the *Optifast* diet; 2 ppts took diet compliant portions of regular food.
29	Bottin et al., 2014<br>UK<br>Abstract only	21 ppts undergoing Roux-en-Y gastric bypass<br>I1=7:<br>I2=7:<br>C=7	**Overall BMI kg/m^2**<br>45.7(0.6)	**Overall Age yrs**<br>44(3)	ONS:<br>Liquid formula 800-1000 kcal/day low calorie diet for 2 wks	Standard 2 wk 1000kcal/day low carb, high protein diet	Self-delivery (oral), NR how info was relayed;<br>NR;<br>NR;<br>NR	NR;<br>NR

ID	1st Author, year and country	Total *n* Intervention (I) Control (c)<br><br>Number analysed (An)if reported	Patient population, baseline clinical characteristics (mean (SD) or *n* (%) unless otherwise stated	Demographics (mean (SD) or *n* (%) unless otherwise stated)	Intervention	Comparator	Mode of delivery; place of delivery; training level of individuals who delivered the intervention; the number of contacts	Intervention fidelity; Compliance or adherence to intervention
**NUTRITIONAL/DIETARY INTERVENTION**								
30	Chakravartty et al., 2019<br>UK	28 morbidly obese ppts undergoing laparoscopic Roux-en-Y gastric bypass<br>I=15 (10An):<br>C=13 (10An)	**BMI kg/m^2 (median/range)**<br>I=53.4(45.1–61.7)<br>C=52.8(42.1–63.0)<br>**ASA grade (median/range)**<br>I=2(1–3)<br>C=2(1–2)<br>**Co-morbidities**<br>**Hypertension**<br>**I**=3:C=1<br>**Asthma**<br>I=3:C=1<br>**Obstructive sleep apnea**<br>I=4:C=2<br>**Reflux**<br>I=3:C=1<br>**Polycystic ovarian disease**<br>I=3:C=2<br>**Hypothyroidism**<br>I=1:C=1<br>**Osteoarthritis**<br>I=3:C=3<br>**Depression**<br>I=4:C=1<br>**Hyperlipidaemia**<br>I=6:C=3	N: I=10:C=10<br>**Age yrs**<br>I=43.5(26-60)<br>C=38.5(24-66)<br>**Gender (%M)**<br>I=0:C=10	4-wk very low-calorie (800kcal) diet<br>This diet is based on the validated "Cambridge milk diet".	Normal diet	Self-delivery (oral), NR how info was relayed;<br>NR: presume home-based;<br>NR;<br>NR;	NR;<br>NR
31	Elrefai, 2017;Elrefai, 2019<br>Country NR (Abstract only)	40 ppts undergoing laparoscopic sleeve gastrectomy<br>I=20:C=20	**Overall BMI kg/m^2**<br>50.5(2.4)	NR	2-wk low caloric diet (LCD)	No dietary regimen	Self-delivery (oral), NR how info was relayed<br>Assumed home;<br>NR;<br>NR	NR;<br>NR
32	Faria et al., 2015<br>Brazil	142 obese ppts undergoing bariatric surgery<br>I=71:C=71	NR	**Age yrs**<br>36(10)<br>**Gender (%M)**<br>I=22:C=15	Very low-calorie liquid diet (controlled diet plan) for 14 days	Normal consistency diet (controlled diet plan) for 14 days	Detailed written instructions (3 sample menu plans) + verbal;<br>Home;<br>Dietician;	NR;<br>approximately25% of both groups presented signs of ketonuria. It was observed

ID	1st Author, year and country	Total *n* Intervention (I) Control (c)<br><br>Number analysed (An)if reported	Patient population, baseline clinical characteristics (mean (SD) or *n* (%) unless otherwise stated	Demographics (mean (SD) or *n* (%) unless otherwise stated)	Intervention	Comparator	Mode of delivery; place of delivery; training level of individuals who delivered the intervention; the number of contacts	Intervention fidelity; Compliance or adherence to intervention
**NUTRITIONAL/DIETARY INTERVENTION**								
					10-12 kcal/kg/day of energy and 1-1.2g/kg of protein were given using products with ideal weights		BL and 14th day	that 26% of the 104 patients had positive signs of ketonuria, with no significant difference between groups (P=.2958).
33	Grundmann et al., 2018 Germany	82 ppts scheduled for cardiac surgery involving cardiopulmonary bypass<br>I=41:C=41	**BMI (kg/m²) median (IQR)**<br>I=26.9(24.7–30.7)<br>C=26.7(25.2–30.3)<br>**Chronic kidney disease**<br>I=15(42):C=15(37.5)<br>**Peripheral arterial disease**<br>I=4(11):C=7(17.5)<br>**Congestive heart failure**<br>I=8(22):C=6(15)<br>**Previous heart surgery**<br>I=3(8):C=1(2.5)<br>**Coronary artery disease**<br>I=30(83):C=25(62.5)<br>**Left main stem disease**<br>I=6(17):C=4(10)<br>**COPD**<br>I=5(14):C=6(15)<br>**Hypertension**<br>I=33(92):C=33(82.5)<br>**Diabetes**<br>I=13(36):C=20(50)	**Age median (IQR)**<br>I=72(63–76)<br>C=75(70–77)<br>**Gender (%M)**<br>I=80.6:C=77.5	Calorie-restricted diet: *Fresubin* energy fiber drink to provide 60% of the daily energy expenditure (DEE) and individually assessed activity factors.<br><br>Ppts were instructed not to consume extra food or calorie-containing beverages like alcohol, fruit juices, or soft drinks.<br>The diet commenced on preop day -7 and was maintained until day -1. Median daily calorie intake was 1313 kcal (IQR: 1224–1412 kcal), equivalent to 60% (1.5%) of the calculated DEE.<br>On the day of surgery, ppts were maintained in a fasting state until surgery.	Ad libitum diet (usual diet, non-restricted calorie intake).	Self-delivery (oral), NR how info was relayed;<br>Home based;<br>NR;<br>Regular phone calls ascertained ppts' well-being and monitored adherence	NR;<br>Ppts in both groups were provided with diaries and reported their food consumption on a daily basis. All ppts in the C group reported they had not changed their eating habits and not completed a diet
34	Hollis et al., 2020 Australia	50 obese ppts awaiting general surgery	BMI **(kg/m²)**<br>I=40.3 (6.0)<br>C= 40.7 (5.9)	**Age, yrs**<br>I= 48.2 (13.3)<br>C= 51.8 (12.2)	8-wk VLCD program including advice to manage symptoms and promote adherence. The VLCD	Standard care consisting of generic weight loss tips and healthy information	Self delivered (oral);<br>At home;<br>Trained dietician;	NR;<br>NR

ID	1st Author, year and country	Total *n* Intervention (I) Control (c)<br><br>Number analysed (An)if reported	Patient population, baseline clinical characteristics (mean (SD) or *n* (%) unless otherwise stated	Demographics (mean (SD) or *n* (%) unless otherwise stated)	Intervention	Comparator	Mode of delivery; place of delivery; training level of individuals who delivered the intervention; the number of contacts	Intervention fidelity; Compliance or adherence to intervention
**NUTRITIONAL/DIETARY INTERVENTION**								
		I= 25:C=25		**Gender (%M)**<br>I=39:C=35	utilised Optifast (Nestle Health, Germany) meal replacement shakes to restrict intake to 700 to 800 calories/d intake with >0.75 g/kg adjusted body weight protein. The consumption of 3 to 4 Optifast shakes mixed on water + > 2 cups (non-starch) vegetable/ salad, at least 2L of energy free fluids, and one teaspoon of vegetable oil were recommended daily for 8 wks	sheets	NR	
35	Schouten et al., 2016<br>The Netherlands	212 morbidly obese ppts undergoing primary laproscopic with Roux-en-Y gastric bypass<br>I=105:C=107	**Preoperative BMI (kg/m²)**<br>I=42.8:C=41.1<br>Co-morbidities were present in many ppts and often in combinations:<br>**insulin dependent DM %**<br>I=11:C=5.2<br>**Non-insulin dependent diabetes m %**<br>I=27:C=12.8<br>**Hypertension %**<br>I=78:C=36.8<br>**Hyperlipidemia %**<br>I=55:C=25.9<br>**Obstructive sleep apnea syndrome %**<br>I=20:C=9.4<br>**Degenerative joint disease %**<br>I=13.7:C=67.0	**Age (yrs)**<br>I=40.2:C=41.7<br>**Gender (%M)**<br>**I**=19:C=23	Prodimed: Commercially available very low-calorie diet (protein shakes) 10 days before surgery<br><br>Total daily intake:<br>Kcal 650, Protein 101g, Carbohydrate 12g, Fat 16g	Standard diet (low on carbohydrates)<br><br>Total daily intake:<br>Kcal 647/657<br>Protein 81/86g<br>Carbohydrate 20g<br>Fat 21/25g<br>(Both diets were comparable in amounts of calories, proteins, carbohydrates, and fats)	Self-delivery (oral) & written;<br>Home;<br>NR;<br>NR	NR;<br>compliance, tolerance and acceptance of the diets measured by a self-designed questionnaire and a daily diet book filled out by the patient during 10 days.<br>patients in the Ct group were more tolerant of their diet compared to those in I group

ID	1st Author, year and country	Total *n* Intervention (I) Control (c)<br><br>Number analysed (An)if reported	Patient population, baseline clinical characteristics (mean (SD) or *n* (%) unless otherwise stated	Demographics (mean (SD) or *n* (%) unless otherwise stated)	Intervention	Comparator	Mode of delivery; place of delivery; training level of individuals who delivered the intervention; the number of contacts	Intervention fidelity; Compliance or adherence to intervention
**NUTRITIONAL/DIETARY INTERVENTION**								
36	van Ginhoven et al., 2011a,b The Netherlands	30 ppts undergoing laparoscopic nephrectomy (volunteers for live kidney donation). I=17:C=13	**BMI (kg/m^2)** I=25.0(4.0) C=26.7(3.4) **Blood glucose (mmol/L)** I=4.7(0.5) C=4.6(0.6)	**Age yrs** I=54(9) C=56(13.I) **Gender (%M)** I=46:C=35	Reduce calorie intake by 30% (relative to BL measurements) on day 4, 3 and 2 before operation.	Eat ad libitum and keep a food record form during the 4 preop days. Ppts instructed to record any changes from preop prescribed diet.	Self-delivery (oral); Ppt-led at home; Dietician – completed calorific assessment; 1x on day before surgery to obtain BL characteristics	NR; food record form: All ppts adhered to the study protocol without reporting adverse events.
37	Van Nieuwenhove et al., 2011; Dambrauskas et al., 2013 Sweden	294 ppts undergoing laparoscopic gastric bypass. I=149 (137An): C=145 (136An)	**BMI (kg/m^2)** I=43.4(10.0) C=43.3(8.2) **Co-morbidity Diabetes M %** I=14:C=14 **Arterial hypertension %** I=45:C=44 **Obstructive sleep apnea syndrome** I=15:C=11 **Pulmonary disease %** I=10:C=12 **CVD** I=6:C=10 **Gastroesophageal reflux disease** I=5:C=11 **Joint problems** I=20: C=17	**Based on N analysed** **Age yrs** I=39.7(9.5) C=40.3(9.7) **Gender (%M)** I=30.5:C=29	Very low-energy diet designed to replace 3 meals/day during a 14-day period. (5 shakes/day are consumed which provides 1906kJ plus recommended daily allowance of essential vitamins, minerals and trace elements).	Regular diet until day of procedure.	Self-delivery (oral), NR how info was relayed; Ppt-led at home; NR; 30 days PO	NR; 16 ppts(10.7%)could not complete the allocated regimen due to intolerance of the diet, lack of adherence to the diet regimen, or both.
**Pre/Probiotics**								
38	Anderson et al., 2004 UK	N=137 Elective laparotomy surgery 68% underwent colectomy I=72:C=65	Severity of illness reported: **GI malignancy N** I=43:C=43 **IBD** I=10:C=7	**Age median (IQR)** I=71(47-76) C=71(66-80) **Gender (%M)** I=53:C=65	Probiotic 1 capsule 3x/day + prebiotic (16g oligofructose dissolved in water) 2x/day	Placebo capsules and sucrose powder (identical quantity)	Self-delivery (oral), NR how info was relayed; Home; NR; NR	NR; Ppts were asked to bring unused capsules in. 1 ppt found capsules unpalatable

ID	1st Author, year and country	Total *n* Intervention (I) Control (c)<br><br>Number analysed (An)if reported	Patient population, baseline clinical characteristics (mean (SD) or *n* (%) unless otherwise stated	Demographics (mean (SD) or *n* (%) unless otherwise stated)	Intervention	Comparator	Mode of delivery; place of delivery; training level of individuals who delivered the intervention; the number of contacts	Intervention fidelity; Compliance or adherence to intervention
**NUTRITIONAL/DIETARY INTERVENTION**								
			**Diverticular disease**<br>I=10:C=7<br>**Aortic aneurism**<br>I=2:C=3<br>**Other**<br>I=11:C=5					
39	Consoli et al., 2016 Brazil	68 ppts undergoing colon resection<br>I=39 (15An):<br>C=29 (18An)	**BMI (kg/m^2)**<br>I=24.4:C=23.7<br>**Benign/malignant neoplasms**<br>I=1/9:C=0/16<br>**Other diseases**<br>I=5:C=2<br>**Subjective global assessment**<br>A: I=8:C=12<br>B: I=7:C=5<br>C: I=0:C=1	N: I=15:C=18<br>**Age yrs median (range)?**<br>I=51(28-76)<br>C=59(17-83)<br>**Gender (%M)**<br>I=33:C=56	1xdaily oral lyophilized yeast capsule with 100 mg (0.5 × 10^9 CFU/g of *S boulardi*. The treatment started at least 7 days before surgery	Usual care	Self-delivery (oral), NR how info was relayed;<br>Home;<br>NR;<br>The principle investigator called ppts daily reminding them to take the capsules (at least 7)	NR;<br>The probiotic therapy was well tolerated in all ppts in the probiotic group, and the mean intake time was 9 days
40	Grat et al., 2017 Poland	55 ppts undergoing liver transplantation<br>I=26 (21 An):<br>C=29 (23 An)<br><br>Transplant recipients<br>I=21:C=23	**Child-Turcotte-Pugh class at enrollment: Class A/B/C**<br>I=6/12/6<br>C=7/15/4<br>**Charlson comorbidity index median (IQR)**<br>I= 3 (3-4):<br>C= 3 (3-4)	**Age yrs Median (range?)**<br>N: I=24:C=26<br>I=52(43-58)<br>C=48(35-61)<br>**Overall Gender (%M)**<br>75	Probiotic capsules (ProBacti 4 Enteric®) containing 3 x 10^9 CFUs of Lactococcus lactis PB411 (50.0%), Lactobacillus casei PB121 (25.0%), Lactobacillus acidophilus PB111 (12.5%), and Bifidobacterium bifidum PB211 (12.5%) – 1x daily before breakfast until transplant	Placebo 1x daily before breakfast until transplant	Self-delivery (oral), NR how info was relayed;<br>Home based;<br>NR;<br>NR	NR;<br>Compliance not reported.
41	Krebs, 2016 Slovenia	73 ppts undergoing large bowel operation for colorectal cancer<br>54 completed<br>I(A)=18<br>I(B)=20	**ASA score median (range)**<br>I(A)=1.72(1–3)<br>I(B)=2.15(2–3)<br>C=2.06(1–3)	**Age yrs, median (range)**<br>I(A)=62(43-87)<br>I(B)=64(46-81)<br>C=67(52–78)<br>**Gender (%M)**<br>I(A)=61:I(B)=65<br>C=56	I(A): 2 sachets/day of Synbiotic 2000 FORTE, containing 4 different lactic acid bacteria and prebiotics (betaglucan, inulin, pectin and resistant starch), for 3 days before operation	Usual care (standard mechanic preop bowel preparation)	Self-delivery (oral), NR how info was relayed;<br>NR;<br>NR;<br>NR	NR;<br>Compliance/adherence: higher concentrations of lactobacilli in ppts who received synbiotics. Significant difference for all 4 lactic acid bacteria (P<0.0001).

ID	1st Author, year and country	Total *n* Intervention (I) Control (c)<br><br>Number analysed (An)if reported	Patient population, baseline clinical characteristics (mean (SD) or *n* (%) unless otherwise stated	Demographics (mean (SD) or *n* (%) unless otherwise stated)	Intervention	Comparator	Mode of delivery; place of delivery; training level of individuals who delivered the intervention; the number of contacts	Intervention fidelity; Compliance or adherence to intervention
**NUTRITIONAL/DIETARY INTERVENTION**								
		C=16			I(B): 2 sachets/day of prebiotics as above, for 3 days before operation			There was always a diff between I(A) and I(B) or C, however there was no diff between groups I(B) and C.
42	Polakowski et al., 2019<br>Brazil	73 ppts undergoing surgery for colorectal cancer<br>I=36<br>C=37	**Staging of disease**<br>**I/II/III**<br>I=22/61/17<br>C=24/57/19	**Age, yrs**<br>I=60.9 (6.7)<br>C=58.9 (6.3)	2x/day symbiotic diluted in 100mls of water<br><br>The synbiotic used was Simbioflora (Farmoquimica, Sao Paulo, Brazil), a dietary supplement comprising 6 g of fructooligosaccharide, and the probiotics Lactobacillus acidophilus NCFM, L. rhamnosus HN001, L. casei LPC-37, and Bifidobacterium lactis HN019 in the concentration of 109	2x/day for 7 days placebo diluted in 100mls water (same instructions as the I group)<br>The placebo was maltodextrin (obtained from cornstarch).	Self-delivery (oral);<br>Home;<br>Principal investigator;<br>Daily phone call for 7 days	NR;<br>NR
43	Zhang et al., 2012<br>China	60 ppts undergoing radical colorectal resection for CRC<br>I=30:C=30	**Cancer stage I/II/III/IV**<br>I=4/18/8/0<br>C=3/18/9/0<br>**BMI (kg/m^2)**<br>I=24.4 (2.1)<br>C=25.1(2.5)	**Age yrs median (range)**<br>I=67.5 (45.0–87.0)<br>C=61.5 (46.0–82.0)<br>**Gender (%M)**<br>I=33:C=44	3 oral bifid triple viable capsules, each contained 0.21 g (10^8 cfu/g) of B longum, L acidophilus and Enterococcus faecalis (Shanghai Sine Wangxiang Pharmaceutical Co., Shanghai, China), 3 x/day	3 placebo capsules containing maltodextrin 3 times/day	Oral;<br>NR;<br>NR;<br>NR	NR;<br>NR (although fecal bacteria counts were assessed which might indicate compliance)
**Nutritional optimization**								
44	Chuah et al., 2014<br>UK<br>Abstract only	41 obese type 2 diabetes ppts undergoing Roux-en-Y gastric bypass surgery<br>I=NR:C=NR	HbA1c > 8.5%	NR	Glucose optimization 3 mths before surgery. Details of intervention NR	NR (assume no glucose optimization)	Self-delivery (oral), NR how info was relayed;<br>Assumed home;<br>NR;<br>NR	NR;<br>NR
45	Flynn and Leightty, 1987<br>USA	36 malnourished ppts undergoing surgery for squamous cancer of the	**American Joint Committee on Cancer stages I & II**<br>I=6:C=11	**Age yrs (presume mean)**<br>I=59:C=68	Nutirional optimisation (dietary advice)<br>Between the 1st office visit and hospital admission (from 10 to 21 days) ppts were given nutritional	Nutritional counseling and suggestions on ways to cope with eating problems	Oral (assume written as well);<br>Assumed home;<br>Dietitian;<br>As necessary, with frequency determined by the dietitian	NR;<br>NR;<br>Ppts were encouraged to comply with the protocol when contacted by the dietitian

ID	1st Author, year and country	Total *n* Intervention (I) Control (c)<br><br>Number analysed (An)if reported	Patient population, baseline clinical characteristics (mean (SD) or *n* (%) unless otherwise stated	Demographics (mean (SD) or *n* (%) unless otherwise stated)	Intervention	Comparator	Mode of delivery; place of delivery; training level of individuals who delivered the intervention; the number of contacts	Intervention fidelity; Compliance or adherence to intervention
**NUTRITIONAL/DIETARY INTERVENTION**								
		upper aerodigestive tract.<br>I=19:C=17	**Stages III & IV**<br>I=13:C=6<br>**Previous irradiation:**<br>I=15:C=8<br>**Extent of procedure:**<br>Limited-intermediate<br>I=9:C=8<br>Major<br>I=5:C=9<br>Extended<br>I=5:C=0		counseling plus specific recommendations to meet individual nutrient requirements to fulfill intake needs			
46	Patel et al., 2004 (Retrospective analysis of Frost et al., 1996; Frost et al., 1998). (probably Indian Sub-continent)	35 ppt undergoing elective CABG<br>18 in low Glycemic Index diet<br>17 in high Glycemic index diet	NR	**Age**<br>C=61 (6)<br>I=59 (7)<br>**Gender (%M)**<br>Low=76<br>High= 89	Low glycemic index diet<br>4 weeks prior to surgery up to day before surgery<br>Ppts asked to swap carbohydrates they currently consumed for low glycemic index foods. E.g. replacing cornflakes with all bran, potatoes to pasta and wholemeal to granary bread. Emphasis on low GI foods (GI<80)	High glycemic index diet<br>Ppts asked to swap carbohydrates they currently consumed for high glycemic index foods. E.g. white bread, cornflakes, potatoes. Emphasis on high GI foods (GI>80)	Not stated but assume face to face for dietary advice;<br>NR;<br>NR;<br>Wk 1 and 4	NR;<br>Ppts completed 7 day unweighed diet diaries each week
**Other Nutritional interventions**								
47	Akbarzadeh et al., 2016 Iran (PhD thesis)	105 non-diabetic ppts scheduled for CABG randomized into 4 groups<br>1(SP)=27 (22An):<br>4(PP)=26 (23An)	**BMI kg/m^2**<br>SP=26.2(4.56)<br>PP=25.8(3.77)<br>**Hypertension**<br>SP=11:PP=16<br>**Hyperlipidemia**<br>SP=9:PP=11	**Age yrs**<br>SP=56.90(7.5)<br>PP=55.21(8.3)<br>**Gender (%M)**<br>SP=54.5:PP=73<br>**Previous smoker (N)**<br>SP=1:PP=3<br>**Current smoker (N)**<br>SP=12:PP=11	SP (supplement): the supplement was composed of glutamine (15g), L-carnitine (3g), vitamin C (750mg), vitamin E (250mg), and selenium (150 µg).<br>7 days before surgery<br>(also taken 30 days post-surgery)	PP (placebo):<br>Indistinguishable placebo for 7 days before + 30 days after surgery<br><br>(tested blindly in volunteers to confirm that they were not distinguishable).	Self-delivery (oral), NR how info was relayed;<br>Assumed home;<br>Self-delivered;<br>NR	NR;<br>NR

ID	1st Author, year and country	Total *n* Intervention (I) Control (c)<br><br>Number analysed (An)if reported	Patient population, baseline clinical characteristics (mean (SD) or *n* (%) unless otherwise stated	Demographics (mean (SD) or *n* (%) unless otherwise stated)	Intervention	Comparator	Mode of delivery; place of delivery; training level of individuals who delivered the intervention; the number of contacts	Intervention fidelity; Compliance or adherence to intervention
**NUTRITIONAL/DIETARY INTERVENTION**								
		(groups 2 & 3 not relevant to this review)						
48	Krasowska et al., 2019 Poland	39 ppts undergoing lumbar spine surgery utilizing static or dynamic implants (posterior lumbar interbody fusion, PLIF).<br>I=18:C=21	3/39 smoked and 0/39 drank alcohol while the project was running .<br>15 ppts noted to have hypertension<br>BMI :<br>I=29.15 (1.15)<br>C=28.14 (0.51)	**Age, yrs**<br>I=41.92 (2.97)<br>C=47.33 (2.15)<br>**Gender (%M)**<br>I=50:C=43	3200IU of vitaminD/day (Vigantol, Merck). Ppts took 5 drops of vitamin D 1x/day for 5 wks prior to operation.	Placebo of vegetable oil .<br>5 drops of placebo 1xday for 5 wks prior to operation .	Self administered (Oral);<br>NR;<br>NR;<br>NR	NR;<br>NR
49	Makhija et al., 2008 India	30 ppts undergoing CABG<br>I=15:C=15	**Comorbidities (N)**<br>**Diabetes M**<br>I=12:C=6<br>**Hypertension**<br>I=11:C=8<br>**MI**<br>I=1:C=0	**Age, yrs**<br>I=57.0(7.0)<br>C=58.4(5.4)<br>**Gender (%M)**<br>I=80:C=87<br>**Weight kg**<br>I=64.4(7.6)<br>C=67.8(11.7)	Co-enzyme Q10 150-180mg/day in 3 doses for 7 to 10 days preop until morning of surgery.<br>Dose depends on ppt weight.<br>All ppts advised to stop antiplatelet drugs 7 days before admission	No antioxidant or placebo<br><br>All ppts were advised to stop antiplatelet drugs 7 days before admission	Self-delivery (oral), NR how info was relayed;<br>Home;<br>NR;<br>NR	NR;<br>NR
50	Matzi et al., 2007 Austria	32 ppts with non-small cell lung cancer undergoing lung surgery<br>I=16:C=16	**COPD**<br>I=16:C=16<br>**Diabetes**<br>I=3:C=5<br>**CHD**<br>I=5:C=7<br>**MI**<br>I=2:C=2<br>**Hypertension**<br>I=8:C=7<br>**Tuberculosis**<br>I=3:C=4<br>**Renal Insufficiency**<br>I=0:C=1	**Age, yrs**<br>I=64.1(8.6)<br>C=62.4(10.2)<br>**Gender (%M)**<br>I=75:C=62.5	7.2 g a-ketoglutaric acid (a-KG) and 0.720 mg 5-hydroxymethylfurfural (5-HMF) /day subdivided into 3 doses were given as oral micronutrient supplementation. The single preparation of both substances was a drinking ampoule of 30 ml diluted either with cold pure water or orange juice, for 10 days	Normal nutritional guidance but no supplement	Self-delivery (oral), NR how info was relayed;<br>Unclear if hospital or home;<br>NR;<br>NR	NR;<br>NR
51	Metcalf et al., 2007 Australia	84 ppts undergoing on-pump cardiac	NR	**Age, yrs**<br>I1:7-day fish oil=63.9 (7.1)	5 active interventions:<br>I1:7 day fish oil<br>I2: 14 day fish oil	Usual care	Oral;<br>Assumed at home;<br>NR;	NR;<br>Compliance was assessed by interview and changes to

ID	1st Author, year and country	Total *n* Intervention (I) Control (c)<br><br>Number analysed (An)if reported	Patient population, baseline clinical characteristics (mean (SD) or *n* (%) unless otherwise stated	Demographics (mean (SD) or *n* (%) unless otherwise stated)	Intervention	Comparator	Mode of delivery; place of delivery; training level of individuals who delivered the intervention; the number of contacts	Intervention fidelity; Compliance or adherence to intervention
**NUTRITIONAL/DIETARY INTERVENTION**								
		surgery (CABG, valve repair or replacement, or both)<br>I1=15<br>I2=15<br>I3=13<br>I4=16<br>I5=16<br>C=10		I2: 14-day fish oil=65.2 (13.4)<br>I3:21-day fish oil= 63.5 (6.7)<br>I4:Flaxseed oil=64.3 (11.5)<br>I5:Olive oil=68.3 (6.1)<br>C= 60.7 (14.5)<br>**Gender (%M)**<br>I=100: I2=90<br>I3=80: I4=90<br>I5=90: C=90	I3: 21 day day fish oil<br>I4: Flaxseed oil<br>I5: Olive oil<br>The study tested the hypothesis that increased fish consumption is associated with reduced risk of cardiac mortality, therefore we have only considered I1-I3 (we combined groups).		NR	erythrocyte and plasm fatty acid composition

KEY: ASA=American Society of Anaesthesiologists; An=analysed; BL=baseline; BMI=body mass index; C=control; CABG=coronary artery bypass graft; CFU=colony-forming units; CHD=coronary heart disease; COPD=Chronic Obstructive Pulmonary Disease; CVD=cardiovascular disease; DEE=daily energy expenditure; diff=difference; DM=diabetes mellitus; g=gram; GI=gastrointestinal; HbA1c=Haemoglobin A1c; hr(s)=hour(s); I=intervention; IBD=inflammatory bowel disease; IN=immunonutrition; info=information; IQR=interquartile range; ITT=intention to treat; kcal=kilocalories; kg=kilogram; kJ=kilojoule; L=litre; LAB=lactic acid bacteria; M=male; MI=myocardial infarction; min=minute; ml=millilitres; mmol/L=millimoles per litre; mth(s)=month(s); N=number; NR=not reported; NYHA=New York Health Assessment; ONS=oral nutrition supplement; PO=postoperative/postoperatively; POSSUM=physiology and operative severity score; PpPD=pylorus-preserving pancreatoduodenectomy; ppt(s)=participant(s); preop=preoperative/preoperatively; RNA=ribonucleic acid; sd=standard deviation; SGA=Subjective Global Assessment; SGA-A=well nourished; SIRS=systemic inflammatory response syndrome; SGA-B/C=mildly or moderately malnourished/severely malnourished; TNM=tumour, nodal, metastatic; vs.=versus; wk(s)=week(s); x=times; yrs=years

NUTRITIONAL INTERVENTIONS

Table 2. Results

	Study	Total number of withdrawals	Clinical outcomes (mean (SD) or n unless otherwise stated)	Intervention-specific outcomes [(n or mean (SD) unless otherwise reported)] and economic evaluations
**Oral Nutritional Supplements**				
1	Burden et al., 2011	I=5 did not receive surgery<br>C=4 did not receive surgery	**Mortality** (30 d)<br>I=3/54:C=0/62<br>**LoS days median (IQR NR)**<br>I=13.5 (5-99): C=14<br>**Wound infections**<br>I=8/54:C=16/62, P=0.145<br>**Chest infections**<br>I=7/54:C=11/62, P=0.478<br>**UTI**<br>I=8/54:C=6/62, P=0.724<br>**Total PO infective complications**<br>I=20/54:C=20/62, P=0.589	**Outcomes reported: energy intake (Kcal/d)**<br>within group changes from BL to preoperatively only<br><br>**Other adverse events (related to intervention):**<br>Nausea and vomiting were reported by 4 ppts and exacerbation of diarrhea was reported in 2 ppts.
2	Burden et al 2017	Ineligible for surgery<br>I=2:C=2<br>Withdrawn<br>I=0:C=1	**Mortality (30 days)**<br>I=1/55:C=4/45, P>0.05<br>**LoS days (median/IQR) I=50:C=42**<br>I=7 (4.0–10.5): C=7 (4.0–10.0), P=0.630<br>*LoS days (mean/sd Quantile Estimation (QE) conversion method)*<br>*I=7.73 (4.81): C=7.00 (4.45)*<br>**Total complications**<br>I=23/55:C=25/45, P=0.114<br>**PO infective complications**<br>**Wound infection**<br>I=11/55: C=17/45, P=0.044<br>**Chest infection**<br>I=5/55:C=3/45, P=0.359<br>**UTI**<br>I=4/55:C=6/45, P=0.315	**Outcomes reported: % weight loss, Energy (kJ); Protein (g)**<br>Greater % weight loss in I vs. C (P=0.021)<br>Energy and protein:<br>BL – no difference between groups,<br>Preop: higher in I vs. C group (P<0.001, P<0.018)<br><br>**Adverse events of intervention (Intolerance of ONS)**<br>7=did not follow the ONS regimen; 4=nausea, 3=abdominal discomfort, 2=diarrhea
3	Kikutchi et al., 2016	None	**Mortality (<90 days)**<br>I=1/39: C=0/38, P>0.999<br>**LoS days I=39:C=38**<br>I=13.7 (11.2):C=12.7 (13.4), P=0.279<br>**Total PO infective complications:**<br>I=5/39: C=4/38, P>0.999<br>**Clavien-Dindo class I/II/IIIa/IVa/Ivb/V**<br>4/4/2/1/0/1	**Outcomes reported: Biomarkers (albumin (mg/dl)), Refractory ascites +/or pleural infusion, liver failure;**<br>No difference in the rate of refractory ascites in I vs. C (P=0.263). However, the occurrence of refractory ascites and/or pleural effusion was less frequent in the I vs. C (P=0.047).<br>With regard to liver-related complications, no significant difference in the incidence of liver failure and grade of liver failure was evident between groups.

	Study	Total number of withdrawals	Clinical outcomes (mean (SD) or n unless otherwise stated)	Intervention-specific outcomes [(n or mean (SD) unless otherwise reported)] and economic evaluations
			2/9/4/1/1/0, P=0.476<br>**Morbidity**<br>I=12/39: C=17/38, P=0.244	PO serum concentration of reduced state albumin was greater immediately after liver resection I vs. C P<0.05
4	MacFie et al., 2000	None	**All cause perioperative mortality 30 day**<br>I=1/24:C=1/25, NS<br>**LoS days (SD NR) I=24:C=25**<br>*Imputed SD (mean of all SD results)*<br>*I=12 (7.37):C=13 (7.63)*<br>**Septic complications**<br>I=6/24: C=2/25, NS<br>**HRQoL (HAD questionnaire)**<br>**Preop anxiety**<br>I=1/24:C=3/25, NS<br>**PO anxiety**<br>I=2/24:C=2/25, NS<br>**Preop depression**<br>I=1/24:C=0/25, NS<br>**PO depression**<br>I=2/24:C=1/25, NS	**Outcomes reported: Body weight (kg), mean serum albumin (mg/dl), mean mid-arm muscle circumference (cm), mean hand grip strength (pounds) reported at preop, preop, on discharge and PO.**<br>No difference in weight loss between groups, although both lost weight. No differences between groups in terms of the mid-arm muscle circumference or handgrip strength, or serum albumin levels.<br><br>**Adverse events**<br>No AEs in preop supplement group and control, however pre- and post-op supplements group and PO supplements group complained of nausea during PO period.
5	Nagata et al., 2013	None	**LoS days I=11:C=12**<br>I=12.9 (3.5): C=12.4 (4), P=0.759<br>**Duration of fever days I=11:C=12**<br>I=4.4 (3.7):C=7.4 (2.8), P=0.044<br>**PO Complications**<br>Results state none reported in either group except fever (briefer in supplement group)<br>**Other AEs**<br>Removal of abdominal drain (mean days)<br>I=2.9 (1.3): C=3.1 (1.4) p=0.373	**Outcomes reported: Nutritional parameters (transferrin), Immunological parameters (WBC, lymphocyte, and neutrophil, T-cell (CD4/CD8), Liver function (Lactate dehydrogenase (LDH)), Antioxidative capacity (from spectrophotometry)**<br>**Nutritional parameters** The level of transferrin was found to be significantly higher in the I group during the 7 days after surgery. However, no significant difference was found between the 2 groups regarding other nutritional parameters<br>**Immunological parameters**<br>The WBC, lymphocyte, and neutrophil counts of the I group at Days 3 and 7 were found to be lower than those of the CT group, but no difference was found in the T-cell (CD4/CD8) subpopulation count between the 2 groups (data not shown). Although the immunoglobulin level of the I group displayed a tendency to increase more rapidly, no significant differences were found in the overall immunoglobulin level between the 2 groups.<br>**Liver function**<br>A significant decrease in the LDH level of the I group was observed on day 7. However, no difference was found between the 2 groups regarding any other parameters.<br>**Antioxidative capacity (from spectrophotometry)**<br>the I group was found to maintain a higher level of antioxidative capacity than did the C group at each PO day of testing
6	Smedley et al., 2004	I=7<br>C=6	**LoS days I=41:C=44**<br>I=12.8 (4.5): C=14.1 (6.6)<br>**Total PO complications**	**Costing**

	Study	Total number of withdrawals	Clinical outcomes (mean (SD) or n unless otherwise stated)	Intervention-specific outcomes [(n or mean (SD) unless otherwise reported)] and economic evaluations
			I=20/41:C=34/44<br>**Minor complications**<br>I=17/41: C=30/44<br>**Major complications**<br>I=3:C=4<br>**QoL**<br>There were no differences between groups in fatigue and quality of life scores (data not shown).	The overall costs in all 3 supplemented groups were less than those in patients who received no ONS by approximately £300 or 15%/ patient episode,<br>although the difference was not significant
7	Zhao et al., 2018	NR	**LoS days I=33:C=33 Median and QR**<br>I=7.0 (1.0): C=8.0 (2.0)<br>*LoS days (mean/sd Quantile Estimation (QE) conversion method)*<br>*I=7.0 (1.48): C=8.0 (2.97)*<br>**Duration of using stomach tubes (days)**<br>I=2.00 (1.00); C=3.00 (1.00)<br>**Duration of using peritoneal cavity drainage tubes (days)**<br>I=6.00 (2.00) ; C=7.00 (1.50)<br>**Duration of the first postoperative flatus (days)**<br>I=3.00 (1.00): C=4.00 (1.50)<br>**Duration of the first postoperative defecation (days)**<br>I=4.00 (1.00): C=5.00 (2.00)	**Outcomes reported: nutritional status linked to BMI**<br>There were no difference between groups in the nutritional status within 48 hrs of admission and at the 1st day after neoadjuvant chemoradiotherapy (NCRT) (P>0.05).<br>However, the BMI and the concentrations of serum PA, TP, and ALB were higher in I group than in C group (P<0.05<br>The rate of malnutrition according to PG-SGA and nutritional risk according to NRS2002 became lower in the trial group at the 8th days after surgery (P<0.05, Fig. 1), though no difference was observed between the two groups within 48 h of hospitalization and on the 1st day after NCRT and surgery (P>0.05).<br><br>**Cost of hospitalization (10,000)**<br>lower costs in I vs C group<br>Z=2.398, P=0.016
**Immunonutirtion**				
8	Aida et al., 2014	5 intraoperatively excluded due to metastatic disease/ unresectable primary tumour	**All-cause mortality (30day)**<br>I=0/25:C=0/25<br>**LoS days I=25:C=25**<br>no difference in the duration of hospital stay<br>**Total PO infective complications**<br>I=7/25:C=15/25, P=0.023<br>**Pneumonia**<br>I=1/25:C=0/25, P=0.312<br>**Wound infection**<br>I=3/25: C=7/25, P=0.157<br>**Intra-abdominal abscess**<br>I=6/25:C=12/25, P=0.210<br>**Enteritis**<br>I=1/25:C=1/25, P<0.999<br>**Sepsis**<br>I=0/25:C=1/25, P=0.312<br>**Total PO non-infective complications**<br>I=15/25: C=17/25, P=0.556<br>**Pancreatic fistula**	**Outcomes reported: mRNA expression levels of T-bet and the T-bet/GATA-3 ratio on PO days -6, -1, 0, 1, 3, 7, 11.**<br>**Plasma concentration of IL-6, phytohemagglutinin (PHA)-stimulated lymphocyte proliferation, and Con A-stimulated lymphocyte proliferation**<br><br>mRNA expression levels of T-bet (a T-box transcription factor) were greater in the I vs. C group (P<0.05) on PO day 3.<br>Plasma concentration of IL-6 less in I than C group on PO day 0 (P<0.05)<br>PHA-stimulated lymphocyte proliferation, and Con A-stimulated lymphocyte proliferation days after surgery (shown graphically). Greater in I than C group on PO day 7 (P<0.05).

	Study	Total number of withdrawals	Clinical outcomes (mean (SD) or n unless otherwise stated)	Intervention-specific outcomes [(n or mean (SD) unless otherwise reported)] and economic evaluations
			I=5/25:C=7/25, P=0.508<br>**Delayed gastric emptying**<br>I=5/25:C=3/25, P=0.440<br>**Chylous ascites**<br>I=3/25:C=3/25, P<0.999<br>**Intra-abdominal bleeding**<br>I=1/25:C=1/25, P<0.999<br>**Others**<br>I=3/25:C=4/25, P=0.684<br>**Clavien-Dindo classification (Grade I/II/III/IV/V) I=25:C=25**<br>I=0/4/13/1/1:C=1/9/5/1/0, P=0.04<br>**Systemic inflammatory response syndrome (SIRS) days I=25:C=25**<br>I=1.4 (0.2):C=1.7 (0.2), P=0.228	
9	Barker et al., 2013	2<br>I=1 unable to contact<br>C=1 death	**Mortality (in hospital)**<br>I=0/46:C=1/49, P<0.999<br>**LoS I=46:C=49**<br>I=7.1 (4.1): C=8.8 (6.5), P=0.11<br>**Wound infection (30 day) and prescribed antibiotics**<br>I=5/46:C=10/49, P=0.26<br>**Prescribed antibiotics > 24 hrs**<br>I=7/46:C=14/49, P=0.14<br>**Unplanned ICU admission**<br>I=2/46:C=3/49, P<0.999<br>**Total no. of complications**<br>I=8/46:C=14/49, P=0.20	**Outcomes reported: Time to 1st flatus, Time to 1st open bowels, time to commencing fluids, time to commencing oral drink, amount of daily oral diet.**<br>There were no differences between groups on any outcome.<br><br>**Other adverse events relating to prehab**<br>2 (1 spike in blood sugar levels in a diabetic and 1 diarrhea-like symptoms)<br><br>**Economic effect**<br>Admission costs were also reduced in the I group with an average cost saving/ ppt of AUD1576 (P=0.37). Considering individual components of treatment cost, nursing cost showed the greatest reduction of AUD1000/ppt within the treatment group (P=0.08), and allied health costs (including dietitian intervention) were also reduced.
10	Braga et al., 2002	NR but 3 had reduced intake of supplement	**Mortality in hospital**<br>I=1/50:C=2/50<br>**LoS days I=50:C=50**<br>I=13.2 (3,5): C=15.3 (4.1) P<0.01<br>**Total No. of Ppts with complications**<br>I=14/50:C=21/50<br>**Total N of ppts with PO infective complications**<br>I=8/50:C=12/50, P<0.04<br>**Antibiotic therapy days I=50:C=50**<br>I=6.5(1.3):C=8.4(1.8), P<0.004<br>**Total N of ppts with PO Non-infective complications**<br>**Hospital readmission** I=0/50:C=1/50	**Outcomes reported: Time to first bowel movement, time to oral solid food intake after surgery /days, BMI:**<br>Bowel function<br>no difference between groups in bowel function or BMI weight loss
11	Desai et al., 2016	NR	**Mortality**<br>'There were no significant differences in the mortality rate of both the groups.'<br>**LoS (days) I=40:C=40**<br>**I**=8.27 (0.884):C=11.67 (2.690)	**Outcomes reported: Total leucocyte levels, Lymphocyte count, Serum protein, Serum albumin,CRP**<br>The increase in PO total leucocyte levels was lower in I (36%, SD 2287.44) tnan C (62% SD 4328) post operatively.<br>CRP increased by 35% in I and by 53% in C.

	Study	Total number of withdrawals	Clinical outcomes (mean (SD) or n unless otherwise stated)	Intervention-specific outcomes [(n or mean (SD) unless otherwise reported)] and economic evaluations
			**LoS ICU Hrs**<br>I=92.88 (12.38):C=19.858<br>**Pneumonia**<br>I=2/40: C=8/40<br>**Wound infection**<br>I=5/40:C=13/40<br>**UTI**<br>I=2/40:C=8/40	Lymphocyte increased by 28% in I and by 42% in C.<br>Platelet count dropped by –42% in I and –16% in C.<br>Reduction in total protein was is less in I than C<br>Serum albumin decreased by 28% in I and by 53% in C.<br>Average decrease in Hb in study group is 0.9 and experiment group is 1.2 g/dl. (there must be a mistake in reporting as study and experimental group are reported)
12	Fujitani et al., 2012	**Excluded from analysis (N)**<br>I=7: C=6<br>No gastrectomy I=4: C=6<br>>10% WL I=3:C=0	**Mortality (in hospital)**<br>I=0/120:C=0/111<br>**LoS days (median/range) I=120: C=111**<br>I=18 (9–85):C=17 (10–88), P=0·395<br>*LoS days (mean/sd Quantile Estimation (QE) conversion method)*<br>*I=24.01 (15.89):C=23.88 (16.84)*<br>**Wound infection**<br>I=27/120: C=23/111, P=0.268<br>**Infectious complication**<br>I=30/120:C=27/111,<br>**Any complication**<br>I=37/120:C=29/111, P=0.468<br>**Abdominal abscess**<br>I=11/120:C=7/111, P=0·469<br>**Pancreatic fistula**<br>I=8/120:C=7/111, P=1·000<br>**Anastomotic leakage**<br>I=3/120:C=3/111, P=1·000<br>**Wound dehiscence**<br>I=13/120:C=8/111, P=0·369<br>**Drain infection**<br>I=3/120:C=1/111, P=0·623<br>**Pneumonia**<br>I=5/120:C=0/111, P=0·061<br>**Venous catheter infection**<br>I=2/120:C=1/111, P=1·000<br>**Pleural effusion**<br>I=1/120:C=1/111, P=1·000<br>**PO bleeding**<br>I=3/120:C=0/111, P=0·248<br>**Ileus**<br>I=2/120:C=1/111, P=1·000<br>**SIRS**<br>I=46/120:C=34/111, P=0·268<br>**Reoperation**<br>I=0/120:C=0/111	NR

	Study	Total number of withdrawals	Clinical outcomes (mean (SD) or n unless otherwise stated)	Intervention-specific outcomes [(n or mean (SD) unless otherwise reported)] and economic evaluations
13	Gade et al., 2016		**Mortality 30 day**<br>0/19: C=1/19<br>**LoS days, median (range) I=19:C=19**<br>I=11 (6-30): C=16 (8-30)<br>***LoS days*** *(mean/sd Quantile Estimation (QE) conversion method)*<br>*I=14.02 (8.57):C=17.05 (7.09)*<br>**PO infective complications**<br>**Septic shock**<br>I=1/19:C=4/16<br>**Sepsis**<br>I=1/19:C=1/19<br>**SIRS**<br>I=0/19:C=1/19<br>**Anastomotic leak**<br>I=4/19:C=6/19<br>**Intra-abdominal abscess**<br>I=1/19:C=0/19<br>**Cholangitis**<br>I=0/19:C=1/19<br>**Pneumonia**<br>I=0/19:C=4/19<br>**Local wound infection**<br>I=3/19:C=8/19<br>**Fungal infection**<br>I=4/19:C=5/19<br>**Infectious diarrhea**<br>I=0/19:C=2.3/19<br>**PO non-infective complications**<br>**Vascular insufficiency**<br>I=0/19:C=4/19<br>**Cardiac insufficiency**<br>I=1/19:C=1/19<br>**Cardiac arrhythmia**<br>I=1/19:C=5/19<br>**Hypovolemia**<br>I=4/19:C=0/19<br>**Multiorgan dysfunctions syndrome**<br>I=0/19:C=1/19<br>**Renal insufficiency**<br>I=0/19:C=1/19<br>**Ileus**<br>I=1/19:C=0/19<br>**Chylous**<br>I=1/19:C=1/19	**Outcomes reported: functional capacity (questionnaire concerning everyday activities and quality of life issues) and body weight (kg):**<br>No changes in functional capacity and body weight between groups.

	Study	Total number of withdrawals	Clinical outcomes (mean (SD) or n unless otherwise stated)	Intervention-specific outcomes [(n or mean (SD) unless otherwise reported)] and economic evaluations
			**Fistula**<br>I=2/19:C=0/19<br>**Abdominal bleeding**<br>I=1/19:C=4/19<br>**Bleeding from cicatrice**<br>I=0/19:C=1/19<br>**Respiratory insufficiency**<br>I=3/19:C=219<br>**Atelectasis**<br>I=2/19:C=0/19<br>**Transient ischemic attack**<br>I=0/19:C=1/19<br>**Venous thrombosis**<br>I=1/19:C=0/19<br>**Non-infectious wound complication**<br>I=0/19:C=1/19<br>**Non- infectious diarrhea**<br>I=6/19:C=8/19<br>**Anaemia**<br>I=1/19:C=0/19<br>**Reoperation**<br>I=2/19:C=2.5/19<br>**Readmission to hospital**<br>I=4/19:C=1/19<br>**Admission to ICU**<br>I=2/19:C=6/19	
14	Gianotti et al 2002	NR<br>No drops out<br>ITT	**Mortality 30 day**<br>I=1/102:C=1/102<br>**LoS days I=102:C=102**<br>I=11.6 (4.7): C=14.0 (7.7), P=0.008<br>**Total PO infective complications**<br>I=14/102:C=31/102, P=0.006<br>**Total PO non-infective complications**<br>I=30/102:C=36/102<br>**Any complications**<br>I=36/102:C=49/102<br>**Wound infection**<br>I=7/102:C=11/102<br>**Abdominal abscess**<br>I=4/102:C=10/102<br>**Respiratory failure**<br>I=6/102:C=6/102<br>**Respiratory tract infection**<br>I=3/102:C=8/102	**Outcomes reported: Time to first flatus, time to first bowel movement (days), recovery of oral food intake PO (days), weight loss 8 days PO (%), PO abdominal cramping/bloating, vomiting, diarrhea**<br><br>**Time to first flatus**<br>I=3.5 (1.3):C=3.6(1.2)<br>**Time to first bowel movement**<br>I=5.4 (1.5):C=5.2 (1.7)<br>**Recovery of oral food intake after surgery**<br>I=6.7 (2.7):C=7.3 (3.8)<br>**Mean % weight loss 8 days after surgery**<br>I=5.2:C=4.8<br>**PO abdominal cramping/bloating, n**<br>I=16/102:C=14/102<br>**Diarrhoea**<br>I=3/102:C=3/102<br>**Vomiting**<br>I=1:C=2

	Study	Total number of withdrawals	Clinical outcomes (mean (SD) or n unless otherwise stated)	Intervention-specific outcomes [(n or mean (SD) unless otherwise reported)] and economic evaluations
			**Delayed gastric emptying**<br>I=6/102:C=5/102<br>**Pancreatic fistula**<br>I=5/102:C=5/102<br>**UTI**<br>I=4/102:C=5/102<br>**Circulatory insufficiency**<br>I=6/102:C=4/102<br>**Bleeding**<br>I=2/102:C=4/102<br>**Bacteremia**<br>I=0/102:C=5/102<br>**Wound dehiscence**<br>I=3/102:C=3/102<br>**Pleural effusion**<br>I=2/102:C=4/102<br>**Renal dysfunction**<br>I=1/102:C=2/102<br>**Sepsis**<br>I=0/102:C=2/102<br>**Intestinal obstruction**<br>I=1/102:C=1/102<br>**Pulmonary embolism**<br>I=0/102:C=0/102	
15	Gunerhan et al., 2009<br>Turkey	14 ppts (across groups) excluded due to: GI bleeding that started after the nutrition programme, emergency surgery to relieve obstruction, and uncontrolled blood sugar levels	**LoS days IM=13: SE=11: C=9**<br>IM=16.5 (14.8): SE=14.2 (9.1): C=12 (3.7), P>0.05<br>**Wound infection**<br>IM=5/13: SE=2/11: C=3/9, P>0.05<br>**Pneumonia**<br>IM=2/13: SE=4/11: C=1/9, P>0.05<br>**Urinary infection**<br>IM=0/13: SE=1/11: C=0/9, P>0.05<br>**Sepsis**<br>IM=0/13: SE=1/11: C=0/9, P>0.05<br>**Total PO noninfective complications**<br>IM=5/13: SE=2/11: C=3/9, P>0.05	**Outcomes reported: prealbumin (mg/dL), lymphocyte subpopulations (µL)**:<br>Prealbumin increased from BL in the I group (P=0.037) (no change in control group). No between group data.<br>No increase in lymphocyte sub-populations between groups.
16	Hossain et al., 2016	NR	**LoS days**<br>Reported reduced hospital stay and improve recorery<br>**PO complications**<br>Reduced postoperative infectious complications . lower rate of anastomosis dehiscence .	**NR**
17	Kaya et al., 2016	None	**PO infective complications**<br>**Pneumonia**<br>I=1/31:C=1/27	**Outcomes reported: Albumin (mg/dl), FEV-1:**<br>Albumin levels decreased more in C vs. I on PO day 3 (P<0.001).<br>No difference in FEV-1 between groups.

	Study	Total number of withdrawals	Clinical outcomes (mean (SD) or n unless otherwise stated)	Intervention-specific outcomes [(n or mean (SD) unless otherwise reported)] and economic evaluations
			**Non-infective complications**<br>**Prolonged air leak**<br>I=4/31:C=4/27<br>**Atelectasis requiring bronchoscopy**<br>**I=1/31:C=3/27**<br>**Cardiac arrythmia**<br>I=0/31: C=1/27<br>**Total overall complication rate**<br>I=6/31: C=12/27, P=0.049	
18	Manzanares Campillo et al., 2017	NR	**Hospital stay (days): I=42:C=42**<br>I=12.7 (8.3):C=13.1 (10.8), P=0.598<br>Total PO infective complications: I=14/42:C=17/42, P=0.49<br>Minor complications: I=10/42: C=17/42, P=0.1<br>Major complications: I=5/42: C=7/42, P=0.53	**Costs, Euros, mean (SD)**<br>I=Complications=12198(5431), No complications=5393(3461), P<0.001<br>C=Complications=12571(8243), No complications=4513 (1876.5), P<0.001
19	Martinez et al, 2020	NR<br>but 20 in each group analysed	**Mortality**<br>I=1/20:C=2/20<br>**LoS days I=20:C=30**<br>I=15.1 (6.88):C=90.9 (148.8)<br>**Total ppts with infectious complications**<br>I=0/20:C=6/20<br>**Number of infectious complications**<br>I=0/20:C=13/20<br>**Chest infections**<br>I=0/20:C=2/20<br>**Pneumonia**<br>I=0/20:C=3/20<br>**Bacteremia**<br>I=0/20:C=5/20<br>**Central line infection**<br>I=0/20:C=1/20<br>**Fistula recurrence**<br>I=2/20:C=9/20	**Outcomes reported: C reactive protein (CRP), interleukin 6 (IL-6)**<br>Serum CRP was lower in I than C on Day-1 (1.02 vs. 2.5 mg/dL; P < 0.05). No difference between groups were observed postoperatively.<br><br>Serum IL-6 was lower in I than C the day before surgery (7.1 vs. 21.1 pg/mL; P < 0.01), and on PO day 1 (113.8 vs. 260.7 pg/mL; P < 0.05). There was no difference on PO day 3 or PO day 7.
20	Mikagi et al., 2010	43% lost to study<br>I (52%)<br>Change of treatment=8<br>Side effects=3<br>Rejection=1<br><br>C (82%)<br>Change of treatment=2<br>Rejection=1	**LoS after operation (days, not clear whether mean or median)**<br>**I=13:C=13**<br>I=16.3: C=14.5, NS<br>**Total overall complications rate**<br>I=1/13: C=3/13, NS<br>**PO infective complications**<br>**UTI**<br>I=0/13:C=1/13, NS<br>**PO non-infective complications**<br>**Ileus**<br>I=1/13:C=2/13, NS<br>**Atelectsasis**	**Outcomes reported: EPA (mg), albumin (mg/dl), prealbumin (mg/dl), White cell count (mm3), IL-6 (units/ml), Liver function (AST and ALT).**<br>Increased EPA levels in I vs. C group immediately preop.(P<0.05)<br>No difference in albumin and prealbumin<br>White cell count lower in I vs. C group PO day 3 & 7 (P<0.05)<br>IL-6 significantly lower in I vs. C group immediate PO (P<0.05)<br>No difference in liver function

	Study	Total number of withdrawals	Clinical outcomes (mean (SD) or n unless otherwise stated)	Intervention-specific outcomes [(n or mean (SD) unless otherwise reported)] and economic evaluations
			I=0/13:C=2/13, NS	
21	Nakamura et al., 2005	NR	**Mortality (7 day)**<br>I=0/12:C=0/12<br>**LoS days I=12:C=14**<br>I=49.0 (18.3): C=46.1 (15.0), NS<br>**PO complications (cholangitis and bleeding)**<br>I=2/12: C= 2/14	**Outcomes reported: omega 3 fatty acids (mg), thromboxane 2, transferrin, retinol binding protein, inflammatory markers (C-reactive protein, PMN-elastase, a1-acid glycoprotein):**<br>Compared with BL, omega-3 fatty acids increased (P<0.001 vs. control), thromboxane 2 decreased (p<0.001) vs. control, transferrin, retinol binding protein increased (P<0.05 vs. control), and inflammatory markers decreased at the end of the intervention.
22	Pronio et al., 2008 Italy	All ppts randomized were analysed	**Mortality**<br>I=1/22:C=2/21<br>**LoS days I=22:C=21**<br>*Imputed SD (mean of all SD results)*<br>*I=12.3 (7.37):C=16.5 (7.5)*<br>**Total PO complications**<br>I=3/22:C=12/21	**Outcomes reported: Serum albumin and lymphocyte count**<br>Proportion of ppts with low serum albumin and low serum lymphocytes levels significantly lower in I vs C.
23	Russell et al., 2019	2 excluded after randomisation due to wrong surgery being performed	**LoS** days I=17:C=15 **Median and range I=17:C=15**<br>I=9 (4-49): C=8 (3-34)<br>*LoS days (mean/sd Quantile Estimation (QE) conversion method)*<br>*I=16.62 (22.72):C=12.98 (15.13)*<br>**Lower respiratory tract imfection**<br>I=3/17:C=3/15<br>**Wound infection**<br>I=2/17:C=2/15<br>**Gastrointestinal**<br>I=2/17:C=1/15<br>**Blood stream**<br>I=5/17:C=0/15<br>**UTI**<br>I=3/17:C=0/15<br>**Anastomotic leak**<br>I=1/17: C=0/15<br>**Wound dehiscence**<br>I=1/17:C=0/15<br>**AF/bradycardia/tachycardia**<br>I=3/17:C=2/15<br>**Acute kidney injury**<br>I=3/17:C=0/15<br>**Aspirational pneumonia**<br>I=1/17:C=0/15<br>**ARDS**<br>I=1/17:C=0/15<br>**Atelectasis**<br>I=7/17:C=6/17<br>**Bowel obstruction**	**Outcomes reported: Fatigue score (day -1, +7, +30)**<br>**Total lymphocyte count and white cell count (day -1, +1, +3, +5, +10, +30)**<br>**Fatigue score**<br>Changes over time did not differ between groups (P = 0.342)<br>**Performance status**<br>Changes over time did not differ between groups (P = 0.810)<br>**Grip strength**<br>Changes over time did not differ between groups (P = 0.849)<br>**Total lymphocytes (x10^9/L)**<br>Changes over time did not differ between groups (P = 0.575)<br>**White cell count (x10^9/L)**<br>Changes over time did not differ between groups (P = 0.201)<br><br>There were no differences between groups over time for changes in plasma concentrations of CRP, TNF-α, IL-8 and IL-10 to POD7. Circulating IL-6 concentrations were higher in I than C on POD7 (P = 0.017) and tended to be higher on POD1 (P = 0.087) and POD5 (P = 0.088). In both groups on POD7, IL-6 concentrations were elevated (P < 0.0001) compared to baseline, and group x time P = 0.034.

	Study	Total number of withdrawals	Clinical outcomes (mean (SD) or n unless otherwise stated)	Intervention-specific outcomes [(n or mean (SD) unless otherwise reported)] and economic evaluations
			I=1/17:C=3/15<br>**Diarrhoea**<br>I=/17:C= /15<br>**Electrolyte derangement**<br>I=4/17:C=3/15<br>**Encephalopathy**<br>I=1/17:C=0/15<br>**Hypotension**<br>I=2/17:C=0/15<br>**Ileus**<br>I=4/17:C=2/15<br>**Ischaemic optic neuropathy**<br>I=1/17:C=0/15<br>**Nausea and vomiting**<br>I=1/17:C=2/15<br>**Pleural effusion**<br>I=5/17:C=3/15<br>**Pain requiring epidural**<br>I=2/17:C=0/15<br>**Pneumothorax**<br>I=1/17:C=0/15<br>**Non-infected collection**<br>I=1/17:C=0/15<br>**Clavien-dindo grade 0**<br>I=5/17:C=4/15<br>**Clavien-Dindo Grade < III**<br>I=5/17:C=10/15<br>**Clavien-Dindo Grade ≥ III**<br>I=7/17:C=1/15	
24	Sufit et al., 2012	I=3 withdrawals<br>C=1 death	**Mortality (30 day)**<br>I=0/7:C=2/7 (1 inter-op and 1 PO)<br>**LoS days I=7:C=6**<br>I=6 (0.8):C=7 (3.2), P=0.48<br>**PO complications**<br>**vasopressor required PO:**<br>I=0/7:C=3/7<br>**atrial fibrillation**<br>I=1/7:C=2/7<br>**total arrhythmia**<br>I=1/7:C=3/7<br>1 ppt in C group had episode of ventricular tachycardia requiring treatment<br>**Complications/ppt: (mean/sd)**<br>I=0.25 (0.5): C=1.14 (0.90), P=0.03	**Outcomes reported: cardiac markers (TROP I, CK-MB, and MG) and glutamine levels (mmol/l):**<br>Significant differences in all cardiac markers in I vs. C group 24, 48, and 72 hours post-surgery (P=0.029, P=0.047, and P=0.040, respectively),<br>No between-group differences in glutamine levels.

	Study	Total number of withdrawals	Clinical outcomes (mean (SD) or n unless otherwise stated)	Intervention-specific outcomes [(n or mean (SD) unless otherwise reported)] and economic evaluations
25	Tumas et al., 2020	4 patients were excluded prior to surgery,<br>18 ppts were excluded during the surgery because of the change of scope or type of the procedure (these ppts underwent surgeries other than pancreatoduodenectomy)	**Mortality**<br>I=1/30:C=0/40<br>**Overall complication rate**<br>I=24/30:C=33/40<br>**Severe complication rate (CDC 3)**<br>I=4/30:C=9/40<br>**Mild complication rate**<br>I=20/30:C=24/40<br>**CDC 1**<br>I=10/30:C=15/40<br>**CDC 2**<br>I=10/30:C=9/40<br>**CDC 3a**<br>I=2/30:C=1/40<br>**CDC 3b**<br>I=1/30:C=7/40<br>**CDC 4a**<br>I=0/30:C=1/40<br>**CDC 5**<br>I=1/30:C=0/40<br>**CCI Mean**<br>I=18.39 (19.01):C=19.43 (14.88)<br>**CCI Median and IQR**<br>I=20.90 (12.20):C=19.43 (20.9)<br>**HRQoL**<br>In graphical format	**NR**
26	Yoshitomi et al., 2009	NR	**Duration of SIRS (days)**<br>I=0.83 (0.40), C=2.37 (0.37)	NR
**Weight loss interventions**				
27	Alami et al., 2007	61/100 ppts underwent surgery (39 ppts didn't)<br>3 ppts gained weight in I group,<br>13 were deemed ineligible after randomisation, and 23 were lost to FU.	**LoS days I=26:C=25**<br>I=3.4 (1.6):C=3.1(1.1)<br>**PO infective complications**<br>**UTI**<br>I=1/26:C=0/35<br>**PO non-infective complications**<br>**Anastomotic leak**<br>I=0/26:C=0/35<br>**Organ failure (Respiratory failure)**<br>I=0/26:C=1/35<br>**Pulmonary embolus**<br>I=0/26:C=1/35<br>**Bleeding**	**Outcomes reported: % excess weight loss and BMI (kg/m^2):at 2 wk, 6 wk, 3 mth, 6 mth FU**<br>% EWL =P<0.0001; BMI P=0.034 at 2 wks<br>%EWL= P=0.0017; BMI=NS at 6 wks<br>%EWL: P=0.0267 BMI NS at 3 mths<br>%EWL=NS; BM =NS at 4 mths

	Study	Total number of withdrawals	Clinical outcomes (mean (SD) or n unless otherwise stated)	Intervention-specific outcomes [(n or mean (SD) unless otherwise reported)] and economic evaluations
			I=4/26:C=2/35<br>**Re-exploration for tachycardia**<br>I=1/26:C=0/35<br>**Supraventricular tachycardia**<br>I=0/26:C=1/35<br>**Conversion**<br>I=0/26:C=0/35	
28	Barth et al., 2019	I=1 (did not receive surgery)<br>C=2 (did not receive surgery)	**Mortality (30 day)**<br>I=0/30; C=0/30<br>**LoS days Median (IQR) I=30:C=30**<br>I=5 (4-6):C=4 (4-5), NS<br>*LoS days (mean/sd Quantile Estimation (QE) conversion method)*<br>*I= 5.37 (0.77):C=4.38 (0.79)*	**Outcomes reported: diet parameters/day of Kcal, fat (g), protein (g)**<br>All consumed less in I vs. C p<0.001; p<0.001; P=0.015
29	Bottin et al, 2014<br>Abstract only	**NR**	**NR**	**Outcomes reported: weight (kg), waist circumference (cm), trunk of body (L) and liver fat (%) reported**<br>I was most effective in reducing trunk fat, waist circumference, weight and significant liver fat reduction at 2 wk with no further change by 6 wks.
30	Chakravartty et al., 2019	Did not want surgery<br>I=3:C=2<br>further investigation needed<br>I=2<br>Did not complete surgery C=1	**LoS days (median/range) I=10:C=10**<br>I=3 (2-3):C=3 (2-9), P=0.12<br>*LoS (mean/sd Quantile Estimation (QE) conversion method)*<br>*I=2.67 (0.40):C=4.56 (3.83)*<br>**PO complications: Clavien-Dindo<3**<br>I=1/10:C=2/10, P=1.00	**Outcomes reported: Expression of collagens (I and III), liver volume (unit), weight loss (kg), lean mass (kg):**<br>Expression of collagens I and III decreased more in the I vs. C group. Greater decrease in liver volume in I vs C (P=0.03) accompanied by greater weight loss p<0.001) mainly by losing lean mass
31	Elrefai et al., 2017;2019<br>Abstract only	NR	NR<br>Operative time was shorter in I group who underwent preoperative LCD than C (80 min vs 95 min respectively, P 0.03).<br>Intraoperative bleeding and morbidity were similar among both groups	**Outcomes reported: Expression of collagens (I and III), liver volume (unit), weight loss (kg), lean mass (kg):**<br>Expression of collagens I and III decreased more in the I vs. C group. Greater decrease in liver volume in I vs. C (P=0.03) accompanied by greater weight loss p<0.001) mainly by losing lean mass<br>Body weights, BMI, waist circumference, were lower at T1 and T2 in the 20 ppts who completed the preoperative LCD regimen
32	Faria et al., 2015		Relevant outcomes not reported.<br>There was no difference between groups in surgical time (P=0.45); intubation, P=1.0; ventilation, P=1.0; blood pressure, P=1.0; accessing the gastroesophageal junction, P=0.47, bleeding, P=0 .246; liver size, P= 0.79	**Outcomes reported: weight, visceral fat, ketonuria**<br>The liquid diet group lost significantly more weight (P=0.0290) and visceral fat (P=0 .0410) than the normal diet group.<br>There was an increase in ketonuria in both groups after 14 days of treatment, and no difference between the groups.
33	Grundmann et al., 2018	I=5 discontinued treatment:<br>1 withdrew consent,<br>4 rescheduled surgery<br>C=1 discontinued treatment (due to cancelled surgery)	**Mortality (30 day)**<br>I=2/36:C=2/40, P=091<br>**LoS Median (IQR)**<br>I=10 (9–11.5):C=10 (8–12), P=0.68<br>*LoS days (mean/sd Quantile Estimation (QE) conversion method)*<br>*I=10.29 (1.91): C=10 (2.97)*	**Outcomes reported: Creatinine (mg/dL), Urinary neutrophil gelatinase–associated lipocalin (NGAL; lmol/L) 8 hrs after cross-clamping and all other biochemical parameters (WBC count, creatinine kinase (U/L), troponin T (lmol/L), lactate (mmol/L), lactate dehydrogenase (ng/L), N-terminal pro–brain natriuretic peptide (ng/L), at 24 hrs after surgery**

	Study	Total number of withdrawals	Clinical outcomes (mean (SD) or n unless otherwise stated)	Intervention-specific outcomes [(n or mean (SD) unless otherwise reported)] and economic evaluations
			**Acute kidney injury**<br>I=15/36:C=19/40, P=0.60<br>**Renal replacement therapy**<br>I=2/36:C=0/40, P=0.13<br>**MI**<br>I=0/36:C=0/40 NA<br>**Stroke**<br>I=0/36:C=0/40, NA<br>**Atrial fibrillation**<br>I=3/36:C=0/40, P=0.06	No between-group difference in the primary end point of median serum creatinine increment, urinary NGAL and all other biochemical parameters at 24 hrs after surgery
34	Hollis et al., 2019	4 dropped from study<br>I=2:C=2<br>However<br>I=3 C=9 dropped from analysis	**LoS, days I=20:C=14**<br>I=1.2(0.5): C=1.36(0.7)<br>**Wound infection I=20:C=14**<br>I=0(0): C=2(14.2)<br>**QOL**<br>Difference between groups median (range)<br>17 (-14 to 41.4), P=0.009	**Outcomes reported: weight (kg), waist circumference (cm), fat mass (kg), muscle mass (kg)**<br>Difference between BL and post intervention between groups<br>Weight=6.6(1.2), P<0.001<br>Waist circumference=7.5(23), P=0.003<br>Muscle mass=1.43(1.1), P=0.213<br>Fat mass=-4.2(4.8), P=0.002
35	Schoulten et al., 2016	NR	**Total PO complications**<br>I=6/105:C=5/107	**Outcomes reported: Weight loss (kg), diet tolerance and satisfaction with diet**<br>Ppts lost weight with both preoperative diet regimens.<br>Weight loss after the diet period and 4 wks after the procedure were not different in I vs. C.<br>Tolerance: The parameters taste, variance, endurance (*meaning: to stick with the diet as prescribed*), tolerance (*meaning: of the diet ingredients*), and applicability (*meaning: easiness to apply the prescribed diet in daily life*) were all<br>significantly better appreciated by the C group as compared to I group (P=0.000 for all parameters). The parameters nausea and feelings of hunger during the diet period were not scored significantly different in the 2 groups.
36	van Ginhoven et al., 2011a, b	Surgery cancelled/ rescheduled<br>I=1:C=5 Withdrew informed consent<br>I=1:C=2	**PO infective complications**<br>**Epididymitis**<br>I=1/17:C=0/13<br>**PO non-infective complications**<br>**Acute tubular necrosis**<br>I=1/17:C=0/13<br>**Iatrogenic colon perforation**<br>I=0/17:C=1/13<br>**EuroQol questionnaire stated as outcome but not shown in results**	**Outcomes reported: insulin, appetite (VAS-levels), Leukocytes, B-lymphocytes, T-lymphocytes, CD4+ T-lymphocytes, CD8+ T-lymphocytes, natural killer cells, HLA expression shown in graphs.**<br>Calorie intake (kcal/day)<br>I=1322 (251): C=1853 (675)<br>Appetite: no significant differences PO.<br>PO there were no differences between groups in absolute lymphocyte numbers. In the I group a trend towards lower numbers of PO leucocytes. On PO day 1, the number of leukocytes in the C group was higher than the I group but not statistically different (P=0.098).<br>HLA-DR expression on T-lymphocytes was also comparable between both groups. The surgical procedure induced a significant decrease in absolute numbers of B- and T-lymphocytes. However, in the I group the number of

	Study	Total number of withdrawals	Clinical outcomes (mean (SD) or n unless otherwise stated)	Intervention-specific outcomes [(n or mean (SD) unless otherwise reported)] and economic evaluations
				natural killer cells decreased significantly postoperatively (P < 0.001), which was not observed in the control group (P=0.08).<br>CRP levels peaked on PO day 2 in both groups. No statistical differences in CRP levels were found at any time point.<br>Cytokine levels: No differences between both groups were found, although consistent trend to lower numbers in the I group,<br><br>**Adverse events**<br>None
37	van Nieuwenhove et al., 2011	I=16 did not finish diet, 12 lost to FU<br><br>C=2 did not receive surgery, 7 lost to FU	**All cause perioperative mortality**<br>I=0/37:C=0/36<br>**PO infective complications**<br>**Wound infection**<br>I=4/137:C=7/136<br>**UTI**<br>I=1/137:C=1/136<br>**Pulmonary infection**<br>I=1/137:C=2/136<br>**PO non-infective complications**<br>**Anastomotic leak**<br>I=0/137:C=1/136<br>**Wound dehiscence**<br>I=0/137:C=1/136<br>**Wound hemorrhage**<br>I=0/137:C=1/136<br>**Deep wound hemorrhage**<br>I=0/137:C=1/136<br>**GI tract hemorrhage**<br>I=1/137:C=1/136<br>**Pyrexia of unknown origin**<br>I=1/137:C=3/136	**Outcomes reported: Mean weight change (kg), Change in BMI (kg/m2), waist or hip circumference (cm) or waist-to-hip ratio**<br>The mean weight change during the 2 wks preop was significantly greater in I vs. C (P<0.001). The mean weight the day before surgery did not differ between groups. Similarly, BMI on the day before surgery was not different between groups, the reduction in BMI was higher in the I vs. C group P<0.001). No differences in mean waist or hip circumference or waist to hip ratio between groups on the day before surgery or in the difference compared with the corresponding values 2 weeks earlier (data not shown).
**Pre/Probiotics**				
38	Anderson et al., 2004	I=5 related to tolerance of prebiotic so continued with just probiotic capsule	**Mortality (30-day)**<br>I=9/72:C=5/65, P=0.354<br>**LoS days (median/ IQR NR) I=72:C=65**<br>I=8:C=8 reported in text<br>**PO complications**<br>**Total PO infective complications**<br>I=23/72:C=20/65	**Outcomes reported: Systemic inflammation (C reactive protein (CRP), interleukin 6 (IL-6), and antiendotoxin core antibody (IgM EndoCAb), bacterial translactation (defined as a positive mesenteric lymph node or serosal scraping),**<br>***Enteric (****Enterococcus; "Coliforms" (unspecified); Escherichia coli Proteae; Klebsiella; Citrobacter****); Non-enteric (****Staphylococcus aureus; Staphylococcus epidermidis; Pseudomonas; Diptheroids; Candida; Bacillus; Group B strep; Leuconostoc; Lactobacillus; Alpha-haemolytic staphylococci; Micrococcus***)**<br>There were no significant differences between groups in any of these outcomes.

Perry R, *et al. BMJ Open* 2022; 11:e050806. doi: 10.1136/bmjopen-2021-050806

	Study	Total number of withdrawals	Clinical outcomes (mean (SD) or n unless otherwise stated)	Intervention-specific outcomes [(n or mean (SD) unless otherwise reported)] and economic evaluations
				**Other adverse events relating to intervention**:<br>I=5 (4=diarrhea, 1 unpalatable):C=0
39	Consoli et al., 2016	Excluded ppts:<br>I=24<br>short time between first appointment and surgery=19<br>Discontinued probiotic use=5<br><br>C=11<br>Unresectable tumor=8<br>Unviability of the sample biopsy=3	**Mortality (in hospital)**<br>I=0/15: C=0/15, NS<br>**LoS (days) Median IQR NR**<br>I=10:C=11, NS<br>**Total PO infective complications**<br>I=2/15:C=7/18, NS<br>**Perineal infection/fistulae**<br>I=1/15: C=2/18, NS<br>**Intra-abdominal abscesses**<br>I=0/15:C=4/18, NS<br>**UTIs**<br>I=1/15:C=1/18, NS<br>The frequency of infectious complications did not differ according to nutrition status or tumor stage (5 ppts in the C group were in stage 1 or 2, while 5 in the probiotic group were in the same stage; the remaining ppts, in both groups, were in stage 3 or 4; P > .05).<br>**Total non-infective complications**<br>I=1/15 (abdominal hemorrhage that required relaparotomy):C=1/18 (intestinal obstruction), NS	**Outcomes reported: mucosal IL-1β, IL-10, and IL-23A mRNA levels:**<br>Ppts who received probiotics had lower mucosal IL-1β, IL-10, and IL-23A mRNA levels than the C group (P=0.001, P=0.04, and P=0.03, respectively).<br>mRNA expression of other cytokines did not differ between groups (P>0.05).
40	Grat et al., 2017	I=2:C=3 discontinued treatment and were excluded, leaving<br>I=24:C=26 for analysis of preoperative outcomes.<br><br>3 ppts in each treatment group were excluded from post-treatment outcome analyses:<br>I=21<br>C=23.<br><br>Results were based on PP approach.	**Mortality (90-day PO)**<br>I=0/21:C=1/23, p > 0.99<br>**30-day post-transplantation infection rate**<br>I=1/21:C:8/23, P=0.02<br>**90-day PO infection rate**<br>I=1/21:C=11/23, P=0.002<br>**PNF (primary graft non-function)**<br>I=0:C=1, P > 0.99<br>**EAD (early allograft dysfunction) rate:**<br>I=9/21): C=8/23, P=0.76<br>**Major post-transplant complications:**<br>I=5/21:C=7/23, P=0.74<br>No diff in PO hospitalization (P=0.89), ICU stay (P=0.32), and duration of additional antimicrobial therapy (P=0.17)<br>MELD (Model for End-Stage Liver Disease) score over the entire pre-transplant period:<br>I=no change (P=0.71): C=decreased from a median of 13 to 12.5 (P=0.04)<br>No difference between treatment groups for the change in MELD score (P=0.25)<br>No change in the CTP (Child-Turcotte-Pugh) class in either the probiotic (P=0.72) or placebo groups (P=0.51)	**Outcomes reported: Fecal microbiota after 10 wks of intervention:**<br>Increase in the no. of Bacteroides spp. in I vs. C (P=0.008).<br>Increase in no. of Enterococcus spp. (P=0.04) and a tendency towards increasing no. of Lactobacillus spp. (P=0.07) in I vs. C.<br>Bilirubin concentrations were lower and the decrease in aspartate and alanine aminotransferase activities were more rapid in I vs. C (Pp=0.02, P=0.03, and P=0.03, respectively).

	Study	Total number of withdrawals	Clinical outcomes (mean (SD) or n unless otherwise stated)	Intervention-specific outcomes [(n or mean (SD) unless otherwise reported)] and economic evaluations
			Pre-transplant rates of infection (P=0.74), hospitalization due to infection (P=0.23) or complications of cirrhosis (P=0.99), and adverse events (P=0.99) were similar in both groups.	
41	Krebs 2016 Slovenia	19 excluded (no. per group NR) for sign protocol violation or non-compliance, refusal to continue treatment, or decision of the investigator to withdraw ppt	**LoS days**<br>I(A)=10.16: I(B)=11.3: C=10.5, P=0.512 IQR NR<br>**PO Complications**<br>No major complications: overall 9 ppts had PO bowel paresis, 4 had wound infection and 2 pneumonia. No diffs between groups.	**Outcomes reported: systemic inflammatory response as measured by CRP (mg/dl), interleukin-6 (IL-6 units/ml), fibrinogen, leukocyte count and differential blood count prior to operation, on the 1st and 3rd PO day, bowel mucosa and days to:1st peristalsis (median, min-max), 1st solid oral food intake, passing of gasses, passing of stool**<br>No difference between groups in systemic inflammatory response.<br>There were considerably more lactic acid bacteria presented on the mucosa in the I group.<br>No difference between groups in days to:1st peristalsis (median, min-max), 1st solid oral food intake, passing of gasses, passing of stool.
42	Polakowski et al., 2019	During the intervention period, 4 ppts were excluded because they did not take the product according to the recommendation	**Mortality**<br>I=0/36:C=3/37<br>**LoS days median (range) I=36:C=37**<br>I=3 (3-5):C=4 (3-21)<br>*LoS days (mean/sd Quantile Estimation (QE) conversion method)*<br>*I=3.59 (0.57):C=6.48(5.69)*<br>**Infectious complications**<br>I=1/36:C=7/37<br>**Non-infectious complications**<br>I= 0/36:C=4/37<br>**Length of antibiotic use**<br>I=1.42 (0.5)<br>C=3.74 (4.3)	**Outcome reported: Mean I-6 CRP before and after the intervention**<br>There were no between-group differences in CRP and serum I at BL. Significant reduction in both iL-g and CRP in I group but no changes observed in C group PO.
43	Zhang et al., 2012 China	NR	**Mortality**<br>I=0/30:C=0/30<br>**LoS days I=30:C=30**<br>I=12(32):C=14(3), P=0.109<br>**PO infectious complications**<br>I=3.3% to 6.7%: C=3.3% to 30% (overall, 10.0% versus 33.3%, P<0.05)	**Outcomes reported: fecal bacterial colonies, bacterial translocation from the intestine, immune and inflammatory responses**<br><br>Little difference between groups in *E coli* after 2 days of treatment, but significantly higher counts in C than I at first post-operative defecation (<0.05). Higher *B longum* counts in I than C after 2 days of treatment and at 1st PO defecation (p<0.05). Higher ratio of *B longum:E coli* in I than C after 2 days of treatment and at first defecation (p<0.05).<br>I group also had lower levels of endotoxins, D-lactic acids, serum interleukin-6 and C-reactive protein but higher levels of serum IgG and sIgA (all p<0.05) than group B.
**Nutritional optimization**				
44	Chauh et al., 2014	36 analysed for HbA1c so assumed drop out from 41	**LoS**<br>There was no significant difference in hospital LoS between groups.	**Outcomes reported: HbA1c (mmol/mol)**<br>There was no difference in HbA1c changes between groups.
45	Flynn & Leighty 1987	None	**LoS: (days) mean (SD NR) N=19:17**<br>I=18: C=21<br>**Total PO complications**	**COST**

	Study	Total number of withdrawals	Clinical outcomes (mean (SD) or n unless otherwise stated)	Intervention-specific outcomes [(n or mean (SD) unless otherwise reported)] and economic evaluations
			I=6/19: C=10/17	A 3 day decrease in length of stay at the current average cost in Louisville hospitals represents a savings of $2,298/patient and a total cost savings of $43,662 for the entire group of19 ppts.
46	Patel et al., 2004	none	**LoS days mean/SEM)**<br>I=7.1(0.4):C=9.5 (1.4), P<0.05<br>**ICU days (mean/SEM)**<br>I=1.18 (0.13): C=1.38 (1.64), NS	**Outcomes reported: % glucose uptake in adipocytes, IAUC insulin (nmol/min), fasting glucose, insulin, cholesterol (total, LDL and HDL) and IAUC glucose and non-sterified free fatty acids (NEFA mmol/L)**<br>Greater % glucose uptake in adipocytes, and IAUC insulin levels in I vs. C (P<0.05).<br>No differences between fasting glucose, insulin, cholesterol (total, LDL and HDL) and IAUC glucose and NEFA in I vs. C.
**Other**				
47	Akbarzadeh et al., 2016	SP=5: PP=3<br>non-compliance<br>I=2:C=1<br>reoperation due to bleeding<br>I=3:C=1<br>surgery cancelled<br>I=0:C=1	**Mortality**<br>I=0: C=1<br>**LoS (median, IQR)**<br>I =3 (3 - 4): C=4 (3 - 4)<br>**ICU stay (hrs)**<br>I =43.0 (40.5 - 48.0): C= 43 (39 - 44)<br>**Infection, wound**<br>**deep sternal wound infection**<br>I=0/22: C=1/23<br>**CVA (cerebrovascular accident)**<br>I=1/22: C=1/23<br>**Pneumonia (not requiring ventilation)**<br>**I=**2/22: C=3/23<br>**Pneumonia (requiring ventilation)**<br>I =0/22: C=0/23<br>**Superficial wound infection**<br>I=2/22: C=6/23<br>**UTI**<br>I=2/22: C=1/23 P value reported between 4 gps<br>**Non-infective**<br>**Myocardial infarction**<br>I=0/22: C=1/23)<br>**Bleeding – surgical blood loss mL b** (median, IQR)<br>I= 815 (465-1185): C=770 (540-1650)<br>**Intra-aortic balloon pump** (0 in both groups)<br>**Dialysis** (0 in both groups)	**Outcomes reported: QUICKI index and Bennett's index**<br>**QUICK Iindex**<br>quantitative insulin sensitivity check index =<br>1 / log (insulin (µU/mL)) + Log (glucose (mg /dL))<br>No between group differnce P=0.184<br>**Bennttt's Index**<br>1/ log [insulin (U/mL)]×log [glucose (mmol /L)]<br>No between-gropu difference P=0.300
48	Krasowska et al., 2019	3 ppts resigned at the beginning of the study and were excluded from further analyses. NR which group	**PO pain: visual analogue scale (VAS)**<br>**Before surgery**<br>I=2.83 (0.51):C=3.29 (0.37)<br>**After surgery**<br>I=1.28 (0.29):C=2.62 (0.47)	**NR**

	Study	Total number of withdrawals	Clinical outcomes (mean (SD) or n unless otherwise stated)	Intervention-specific outcomes [(n or mean (SD) unless otherwise reported)] and economic evaluations
49	Makhiga et al., 2008	none	**Mortality (during hospital stay)**<br>I=0/15:C=0/15<br>**LoS days I=15:C=15**<br>I=7.1 (1.1): C=10.3 (7.8), P=0.02<br>**ICU LoS (days) I=15:C=15**<br>I=2.2 (0.4): C=2.4 (0.8), P=0.91<br>**Re-exploration and re-suturing**<br>I=0/15: C=4/15, P=0.1<br>1 ppt had atrial fibrillation requiring cardioversion in the C group, whereas in the I group 1 ppt had ventricular fibrillation requiring defibrillation.	**Outcomes reported: antioxidant level. blood glucose levels (mg/dL):**<br>There was no difference in the antioxidant level between groups at any timepoint.<br>BL blood glucose level was comparable between the 2 groups. The blood glucose level was higher in the C group at 30 mins (P=0.01) and 4 hrs after the release of aortic cross-clamp (Pp=0.02) in comparison to the I group<br><br>**Intervention-related-adverse events**<br>No serious side effects of CoQ10 administration were noticed
50	Matzi et al., 2007	NR	**Mortality (in hospital)**<br>I=0/16: C=016<br>**LoS hospital (days) I=16:C=16**<br>I=9.9 (3.6): C=16.2 (5.5), P=0.04<br>**LoS ICU (hrs)**<br>I=0.6 (0.5) C=2.6 (2.0), P=0.02<br>**PO complications**<br>Myocardial Ischemia:<br>I=0/16: C=1/16<br>Bleeding:<br>I=1/16: C=1/16<br>Minor air leak:<br>I=4/16: C=5/16<br>Prolonged air leak:<br>I=1/16:C=3/16<br>Pneumonia:<br>I=0/16: C=1/16<br>Wound healing:<br>I=0/16: C=1/16	**Outcomes reported: Spiroergometric evaluation (VO^2 max, O^2-pulse, max. Watts/kg)**, **Oxidative stress (carbonyl proteins, isoprostanes)**<br><br>**VO^2 max, O^2-pulse, max. Watts/kg**<br>on day 10 of supplementation:7.2 g α-KG and 720 mg 5-HMF showed a significant difference in favor of the study group at the level of maximum exercise capacity and aerobic/anaerobic level concerning VO2 max and Watts per kg compared to the C group<br><br>the study group showed a significant increase of 22.2% for the aerobic/anaerobic level concerning Watts/kg (P=0.0003), and also at the level of maximum exercise capacity for VO2 max of 14.7% (P=0.011) and Watts of 8.4% (P=0.012).<br>Oxidative stress parameters (carbonyl proteins and isoprostanes decreased for the study group compared to the control).
51	Metcalf et al 2007	I1FO7=5<br>I2FO14=5<br>I3FO21=3<br>I4flax=6<br>I5olive=6<br>C=0	**Mortality (in hospital)**<br>I=0/30: C=1/10<br>**LoS & ICU LoS**<br>No significant differences between groups (data not shown).<br>**MI**<br>I=0/30:C=0/10	**Outcomes reported: erythrocyte and atrial phospholipid (EPA/DHA) and serum levels of omega 3-fatty acids (EPA and DHA mg).**<br>No difference in erythrocyte and atrial phospholipid EPA/DHA in I vs C during first 10 days of intervention. No difference in erythrocyte EPA and DHA between groups.

KEY: AE(s)=adverse event(s); AST=aspartate transaminase; ALT=alanine aminotransferase; BL=baseline; BMI=body mass index; C=control; CoQ10=Coenzyme Q10; CK-MB=creatine kinase; cm=centimetre; Con A=Concanavalin A; CRP=C-reactive protein; DHA=docosahexaenoic acid; diff=difference; dL=decilitre; EPA= eicosapentaenoic acid; EQ-5D=EuroQuol-5D; EWL=excess weight loss; FEV=forced expiratory volume; FU=follow up; g=gram; GI=gastrointestinal; HAD=Hamilton anxiety and depression scale; HbA1c=Haemoglobin A1c; HDL=high density lipoprotein; HLA=Human Leukocyte Antigen; HLA-DR=Human Leukocyte Antigen–DR isotype; hr(s)=hour(s); HRQoL=Health related quality of life; I=intervention; IAUC=incremental area under the curve; ICU=intensive care unit; IL=interleukin; IQR=interquartile range; ITT=intention to treat; Kcal=kilocalories; kg=kilogram; kJ=kilojoule; L=litre; LDH=lactate dehydrogenase.

NUTRITIONAL INTERVENTIONS

Table 3. Risk of Bias

	Study	Selection bias		Performance bias		Detection bias						Attrition bias	Reporting bias
						Blinding of outcome assessment							
		Random sequence generation	Allocation concealment	Blinding of participants (all outcomes)	Blinding of personnel (all outcomes)	Perioperative mortality, hospital readmission	Postoperative complications	Length of stay	Patient reported outcomes (pain, HRQoL)	Intervention related outcomes	Intervention related adverse events	Incomplete outcome data	Selective reporting
**Nutritional/dietary interventions**													
**Oral Nutritional Supplements**													
1	Burden 2011								**NR**				
2	Burden 2017								**NR**		**NR**		
3	Kikutchi 2016								**NR**		**NR**		
4	Macfie 2000												
**5**	Nagata 2013					**NR**			**NR**				
**6**	Smedley 2004						**NR**					**NR**	
**7**	Zhao 2018					**NR**			**NR**		**NR**		
**Immunonutrition**													
8	Aida2014							**NR**	**NR**		**NR**		
9	Barker 2013								**NR**				
10	Braga 2002								**NR**		**NR**		
11	Desai 2016<br>Abstract and poster only								**NR**		**NR**		
12	Fujitani 2012								**NR**	**NR**	**NR**		
13	Gade 2016								**NR**		**NR**		

	Study	Selection bias		Performance bias		Detection bias						Attrition bias	Reporting bias
						Blinding of outcome assessment							
		Random sequence generation	Allocation concealment	Blinding of participants (all outcomes)	Blinding of personnel (all outcomes)	Perioperative mortality, hospital readmission	Postoperative complications	Length of stay	Patient reported outcomes (pain, HRQoL)	Intervention related outcomes	Intervention related adverse events	Incomplete outcome data	Selective reporting
**Nutritional/dietary interventions**													
14	Gianotti 2002								NR		NR		
15	Gunerhan 2009					NR			NR		NR		
16	Hossain 2016<br>Abstract Only					NR			NR	NR	NR		
17	Kaya, 2016					NR		NR	NR		NR		
18	Manzanares Campillo, 2017					NR	.		NR		NR		
19	Martinez, 2020								NR		NR		
**20**	Mikagi, 2010					NR			NR		NR		
**21**	Nakamura, 2005								NR		NR		
**22**	Pronio, 2008								NR		NR		
**23**	Russell, 2019					NR			NR		NR		
**24**	Sufit, 2012								NR		NR		
**25**	Tumas, 2020							NR	NR		NR		
**26**	Yoshitomi, 2009<br>Abstract only					NR	NR	NR	NR		NR		
**Weight loss intervention**													
**27**	Alami 2007								NR		NR		
28	Barth 2019								NR		NR		
29	Bottin 2014 (abstract)					NR	NR	NR	NR		NR		

	Study	Selection bias		Performance bias		Detection bias						Attrition bias	Reporting bias
						Blinding of outcome assessment							
		Random sequence generation	Allocation concealment	Blinding of participants (all outcomes)	Blinding of personnel (all outcomes)	Perioperative mortality, hospital readmission	Postoperative complications	Length of stay	Patient reported outcomes (pain, HRQoL)	Intervention related outcomes	Intervention related adverse events	Incomplete outcome data	Selective reporting
**Nutritional/dietary interventions**													
**30**	Chakravartty 2019					**NR**			**NR**		**NR**		
**31**	Elrefai, 2017 Abstract only					**NR**		NR	**NR**		**NR**		
32	Faria 2015					**NR**		**NR**	**NR**		**NR**		
33	Grundmann 2018								**NR**		**NR**		
34	Hollis 2019			from protocol	from protocol	**NR**					**NR**		
35	Scholuten 2016					**NR**		**NR**	**NR**		**NR**		
**36**	van Ginhoven, 2011a,b					**NR**		**NR**	**NR**		**NR**		
**37**	van Nieuwenhove, 2011							**NR**	**NR**		**NR**		
**Pre/Probiotics**													
**38**	Anderson 2004								**NR**				
**39**	Consoli 2016							**NR**	**NR**		**NR**		
40	Grat 2017							**NR**	**NR**		**NR**		
**41**	Krebs 2016					**NR**			**NR**		**NR**		
**42**	Polakowski 2019								**NR**		**NR**		
**43**	Zhang 2012								**NR**		**NR**		
**Nutritional optimization**													

	Study	Selection bias		Performance bias		Detection bias						Attrition bias	Reporting bias
						Blinding of outcome assessment							
		Random sequence generation	Allocation concealment	Blinding of participants (all outcomes)	Blinding of personnel (all outcomes)	Perioperative mortality, hospital readmission	Postoperative complications	Length of stay	Patient reported outcomes (pain, HRQoL)	Intervention related outcomes	Intervention related adverse events	Incomplete outcome data	Selective reporting
**Nutritional/dietary interventions**													
**44**	Chuah et al, 2014					**NR**	**NR**		**NR**		**NR**		
**45**	Flynn & Leighty (1987)					**NR**			**NR**	**NR**	**NR**		
**46**	Patel 2004					**NR**	**NR**		**NR**		**NR**		
**Other Nutritional interventions**													
**47**	Akbarzadeh 2017								**NR**		**NR**		
48	Krasowska 2019					**NR**	**NR**	**NR**		**NR**	**NR**		
49	Makhija, 2008								**NR**				
**50**	Matzi, 2007								**NR**		**NR**		
**51**	Metcalf 2007								**NR**		**NR**		

NR = not reported

NUTRITIONAL INTERVENTIONS

Table 4. Summary of findings (Immunonutrition)

Immunonutrition compared to usual care for any major surgery

Patient or population: any major surgery
Setting: hospital
Intervention: immunonutrition
Comparison: usual care

Outcomes	Anticipated absolute effects* (95% CI)		Relative effect (95% CI)	№ of participants (studies)	Certainty of the evidence (GRADE)	Comments
	Risk with usual care	Risk with immunonutrition				
mortality follow up: 30 days	20 per 1,000	**11 per 1,000** (4 to 28)	**RR 0.55** (0.21 to 1.42)	910 (11 RCTs)	⨁⨁◯◯ LOW [a,b]	
length of stay (LoS) follow up: 30 days		MD **2.11 lower** (3.07 lower to 1.15 lower)	-	1010 (13 RCTs)	⨁◯◯◯ VERY LOW [a,c,d]	
Pneumonia follow up: 30 days	36 per 1,000	**18 per 1,000** (6 to 51)	**RR 0.52** (0.18 to 1.44)	521 (7 RCTs)	⨁◯◯◯ VERY LOW [a,e,f]	
wound infection follow up: 30 days	168 per 1,000	**119 per 1,000** (85 to 166)	**RR 0.71** (0.51 to 0.99)	752 (8 RCTs)	⨁◯◯◯ VERY LOW [a,b,g]	
Total PO complications follow up: 30 days	328 per 1,000	**243 per 1,000** (177 to 334)	**RR 0.74** (0.54 to 1.02)	727 (7 RCTs)	⨁◯◯◯ VERY LOW [a,d,g]	
Total PO infective complications follow up: 30 days	373 per 1,000	**239 per 1,000** (149 to 377)	**RR 0.64** (0.40 to 1.01)	609 (6 RCTs)	⨁◯◯◯ VERY LOW [a,b,c]	

***The risk in the intervention group** (and its 95% confidence interval) is based on the assumed risk in the comparison group and the **relative effect** of the intervention (and its 95% CI).

CI: Confidence interval; **RR:** Risk ratio; **MD:** Mean difference

GRADE Working Group grades of evidence
High certainty: We are very confident that the true effect lies close to that of the estimate of the effect
Moderate certainty: We are moderately confident in the effect estimate: The true effect is likely to be close to the estimate of the effect, but there is a possibility that it is substantially different
Low certainty: Our confidence in the effect estimate is limited: The true effect may be substantially different from the estimate of the effect
Very low certainty: We have very little confidence in the effect estimate: The true effect is likely to be substantially different from the estimate of effect

Explanations

a. Downgrade for Risk of bias;
b. Downgrade for imprecision – includes null and appreciable benefit or harm, small sample size
c. Downgrade for inconsistency-different sizes of CI;
d. Downgrade for imprecision - small sample size
e. Downgrade for inconsistency: based on effects not in same direction, very large CIs different sizes of CI;
f. Downgrade for imprecision – includes null, small sample size
g. Downgrade Inconsistency: based on effects not in same direction

Table 5. Summary of findings (Oral Nutritional Supplements)

Oral nutritional supplements compared to usual care for any major surgery

Patient or population: any major surgery
Setting: hospital
Intervention: oral nutritional supplements
Comparison: usual care

Outcomes	Anticipated absolute effects* (95% CI)		Relative effect (95% CI)	№ of participants (studies)	Certainty of the evidence (GRADE)	Comments
	**Risk with usual care**	**Risk with oral nutritional supplements**				
mortality follow up: 30 days	0 per 1,000	**0 per 1,000** (0 to 0)	**RR 1.18** (0.23 to 6.11)	342 (4 RCTs)	⨁◯◯◯ VERY LOW [a,b,c]	
length of stay (LoS) Scale from: 7 to 14.1 follow up: mean 30 days		MD **0.54 days lower** (1.38 lower to 0.29 higher)	-	392 (6 RCTs)	⨁◯◯◯ VERY LOW [a,b,c]	

***The risk in the intervention group** (and its 95% confidence interval) is based on the assumed risk in the comparison group and the **relative effect** of the intervention (and its 95% CI).

CI: Confidence interval; **RR:** Risk ratio; **MD:** Mean difference

GRADE Working Group grades of evidence
High certainty: We are very confident that the true effect lies close to that of the estimate of the effect
Moderate certainty: We are moderately confident in the effect estimate: The true effect is likely to be close to the estimate of the effect, but there is a possibility that it is substantially different
Low certainty: Our confidence in the effect estimate is limited: The true effect may be substantially different from the estimate of the effect
Very low certainty: We have very little confidence in the effect estimate: The true effect is likely to be substantially different from the estimate of effect

Explanations

a. Downgrade for Risk of bias;
b. Downgrade for inconsistency: based on effects not in same direction different sizes of CI;
c. Downgrade for imprecision – includes null and appreciable benefit or harm, small sample size

Table 6. Summary of findings (Pre/Probiotics)

Pre/probiotics compared to usual care for any major surgery

Patient or population: any major surgery
Setting: hospital
Intervention: pre/probiotics
Comparison: usual care

Outcomes	Anticipated absolute effects* (95% CI)		Relative effect (95% CI)	№ of participants (studies)	Certainty of the evidence (GRADE)	Comments
	**Risk with usual care**	**Risk with pre/probiotics**				
Total PO infective complications follow up: 30 days	330 per 1,000	**158 per 1,000** (46 to 535)	**RR 0.48** (0.14 to 1.62)	214 (3 RCTs)	⨁⨁◯◯ LOW [a,b]	
mortality	0 per 1,000	**0 per 1,000** (0 to 0)	**RR 0.76** (0.17 to 3.42)	344 (5 RCTs)	⨁⨁◯◯ LOW [a,b]	

***The risk in the intervention group** (and its 95% confidence interval) is based on the assumed risk in the comparison group and the **relative effect** of the intervention (and its 95% CI).

CI: Confidence interval; **RR:** Risk ratio

GRADE Working Group grades of evidence
High certainty: We are very confident that the true effect lies close to that of the estimate of the effect
Moderate certainty: We are moderately confident in the effect estimate: The true effect is likely to be close to the estimate of the effect, but there is a possibility that it is substantially different
Low certainty: Our confidence in the effect estimate is limited: The true effect may be substantially different from the estimate of the effect
Very low certainty: We have very little confidence in the effect estimate: The true effect is likely to be substantially different from the estimate of effect

Explanations

a. Downgrade for inconsistency-different sizes of CI;
b. Downgrade for imprecision- includes null effect and appreciable benefit or harm, small sample size

Table 7. Summary of findings (Weight Loss)

Weight loss interventions compared to usual care for any major surgery

Patient or population: any major surgery
Setting:
Intervention: weight loss interventions
Comparison: usual care

Outcomes	Anticipated absolute effects* (95% CI)		Relative effect (95% CI)	№ of participants (studies)	Certainty of the evidence (GRADE)	Comments
	**Risk with usual care**	**Risk with weight loss interventions**				
Length of stay (LoS)		MD **0.22 higher** (0.46 lower to 0.91 higher)	-	241 (5 RCTs)	⨁◯◯◯ VERY LOW [a,b,c]	

***The risk in the intervention group** (and its 95% confidence interval) is based on the assumed risk in the comparison group and the **relative effect** of the intervention (and its 95% CI).

CI: Confidence interval; **MD:** Mean difference

GRADE Working Group grades of evidence
High certainty: We are very confident that the true effect lies close to that of the estimate of the effect
Moderate certainty: We are moderately confident in the effect estimate: The true effect is likely to be close to the estimate of the effect, but there is a possibility that it is substantially different
Low certainty: Our confidence in the effect estimate is limited: The true effect may be substantially different from the estimate of the effect
Very low certainty: We have very little confidence in the effect estimate: The true effect is likely to be substantially different from the estimate of effect

Explanations

a. downgrade for Risk of Bias
b. Inconsistency: variation in size, not in same direction
c. Imprecison: small sample size, wide confidence intervals

Meta analyses

IMMUNONUTRITION

Figure 1. Random effects meta-analysis of the risk ratio of mortality between immunonutrition (experimental) and usual care (control)

Study or Subgroup	Experimental Events	Experimental Total	Control Events	Control Total	Weight	Risk Ratio M-H, Random, 95% CI
Aida 2014	0	25	0	26		Not estimable
Barker 2013	0	46	1	49	9.0%	0.35 [0.01, 8.49]
Braga 2002	1	50	2	50	16.2%	0.50 [0.05, 5.34]
Fujitani 2012	0	120	0	111		Not estimable
Gade 2016	0	19	1	19	9.2%	0.33 [0.01, 7.70]
Gianotti 2002	1	102	1	102	11.9%	1.00 [0.06, 15.77]
Martinez 2020	1	20	2	20	16.9%	0.50 [0.05, 5.08]
Nakamura 2005	0	12	0	12		Not estimable
Pronio 2008	1	22	2	21	16.8%	0.48 [0.05, 4.88]
Sufit 2012	0	7	2	7	11.0%	0.20 [0.01, 3.54]
Tumas 2020	1	30	0	40	9.0%	3.97 [0.17, 94.13]
**Total (95% CI)**		**453**		**457**	**100.0%**	**0.55 [0.21, 1.42]**
Total events	5		11			

Heterogeneity: $Tau^2 = 0.00$; $Chi^2 = 2.35$, df = 7 (P = 0.94); $I^2 = 0\%$
Test for overall effect: Z = 1.23 (P = 0.22)

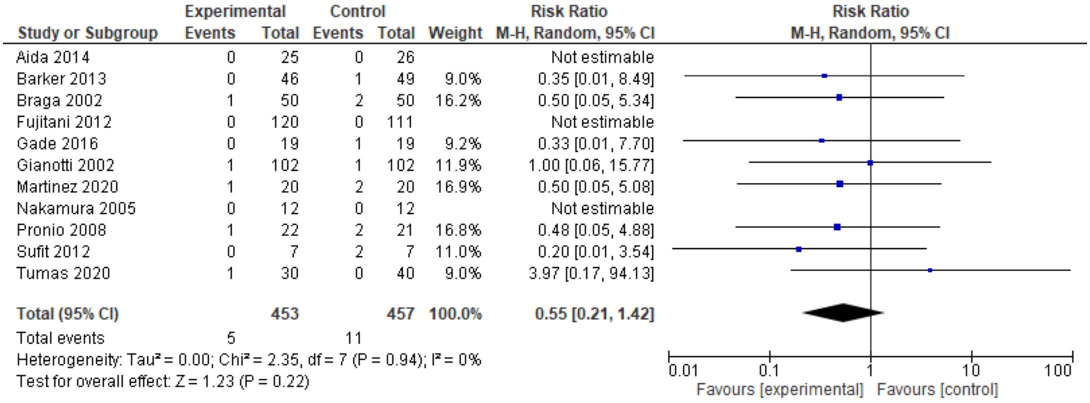

11/19 studies (910 participants).

Figure 2. Random effects meta-analysis of the mean difference in length of hospital stay (LoS) between immunonutrition (experimental) and usual care (control)

Study or Subgroup	Experimental Mean	Experimental SD	Experimental Total	Control Mean	Control SD	Control Total	Weight	Mean Difference IV, Random, 95% CI
Barker 2013	7.1	4.1	46	8.8	6.5	49	12.2%	-1.70 [-3.87, 0.47]
Braga 2002	13.2	3.5	50	15.3	4.1	50	18.2%	-2.10 [-3.59, -0.61]
Desai 2016	8.27	0.884	40	11.57	2.69	40	25.6%	-3.30 [-4.18, -2.42]
Fujitani 2012	24.01	15.89	120	23.88	16.84	111	4.5%	0.13 [-4.10, 4.36]
Gade 2016	14.02	8.57	19	17.05	7.09	19	3.3%	-3.03 [-8.03, 1.97]
Gianotti 2002	11.6	4.7	102	14	7.7	102	15.6%	-2.40 [-4.15, -0.65]
Gunerhan 2009	16.5	14.8	13	12	3.7	9	1.3%	4.50 [-3.90, 12.90]
Manzanares-Campillo 2017	12.7	8.3	42	13.1	10.8	42	4.7%	-0.40 [-4.52, 3.72]
Martinez 2020	15.1	6.88	20	90.9	148.8	20	0.0%	-75.80 [-141.08, -10.52]
Nakamura 2005	49	18.3	12	46.1	15	14	0.5%	2.90 [-10.10, 15.90]
Pronio 2008	12.3	7.37	22	16.5	7.5	21	4.1%	-4.20 [-8.65, 0.25]
Russell 2019	16.62	22.72	17	12.98	15.13	17	0.5%	3.64 [-9.34, 16.62]
Sufit 2012	6	0.8	7	7	3.2	6	9.5%	-1.00 [-3.63, 1.63]
**Total (95% CI)**			**510**			**500**	**100.0%**	**-2.11 [-3.07, -1.15]**

Heterogeneity: $Tau^2 = 0.74$; $Chi^2 = 17.35$, df = 12 (P = 0.14); $I^2 = 31\%$
Test for overall effect: Z = 4.31 (P < 0.0001)

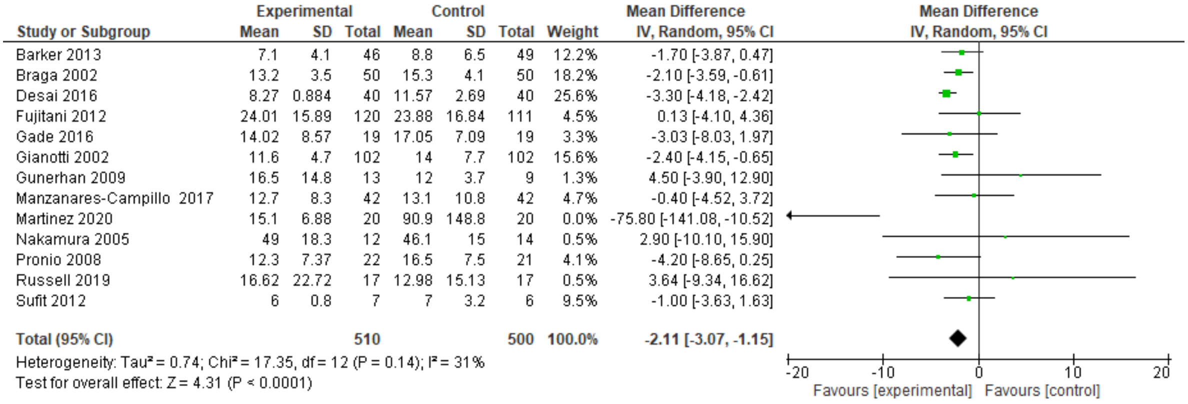

13/19 studies (1010 participants).1 abstract (Desai et al. 2016) included.

Figure 3. Random effects meta-analysis of the risk ratio of total postoperative complications (Infective & non-infective) between immunonutrition (experimental) and usual care (control)

Study or Subgroup	Experimental Events	Experimental Total	Control Events	Control Total	Weight	Risk Ratio M-H, Random, 95% CI
Barker 2013	8	46	14	49	11.1%	0.61 [0.28, 1.31]
Fujitani 2012	37	120	29	111	20.4%	1.18 [0.78, 1.78]
Gianotti 2002	36	102	49	102	23.2%	0.73 [0.53, 1.02]
Kaya 2016	6	31	12	27	10.0%	0.44 [0.19, 1.00]
Mikagi 2010	1	13	3	13	2.1%	0.33 [0.04, 2.80]
Pronio 2008	3	22	12	21	6.6%	0.24 [0.08, 0.73]
Tumas 2020	24	30	33	40	26.6%	0.97 [0.77, 1.22]
**Total (95% CI)**		**364**		**363**	**100.0%**	**0.74 [0.54, 1.02]**
Total events	115		152			

Heterogeneity: Tau² = 0.09; Chi² = 14.60, df = 6 (P = 0.02); I² = 59%
Test for overall effect: Z = 1.84 (P = 0.07)

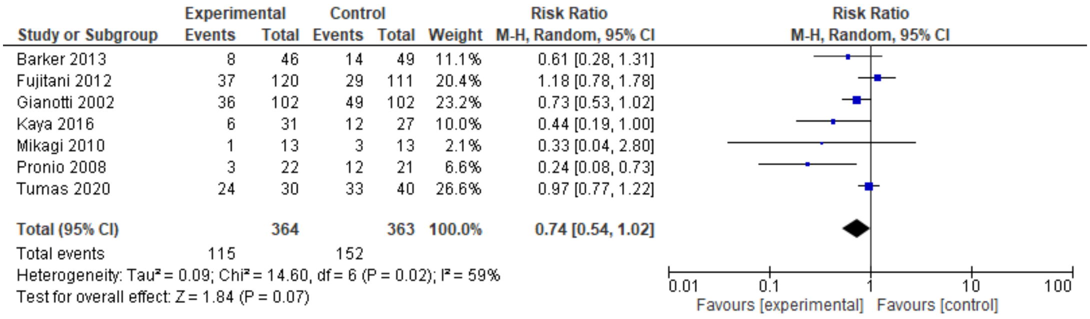

7/14 studies (727 participants).

Figure 4. Random effects meta-analysis of the risk ratio of total infective complications between immunonutrition (experimental) and usual care (control).

Study or Subgroup	Experimental Events	Experimental Total	Control Events	Control Total	Weight	Risk Ratio M-H, Random, 95% CI
Aida 2014	7	25	15	25	20.3%	0.47 [0.23, 0.94]
Braga 2002	8	50	12	50		Not estimable
Fujitani 2012	30	120	27	111	28.3%	1.03 [0.65, 1.61]
Gianotti 2002	14	102	31	102	24.4%	0.45 [0.26, 0.80]
Manzanares-Campillo 2017	14	42	17	42	24.5%	0.82 [0.47, 1.45]
Martinez 2020	0	20	6	20	2.5%	0.08 [0.00, 1.28]
**Total (95% CI)**		**309**		**300**	**100.0%**	**0.64 [0.40, 1.01]**
Total events	65		96			

Heterogeneity: Tau² = 0.14; Chi² = 9.18, df = 4 (P = 0.06); I² = 56%
Test for overall effect: Z = 1.94 (P = 0.05)

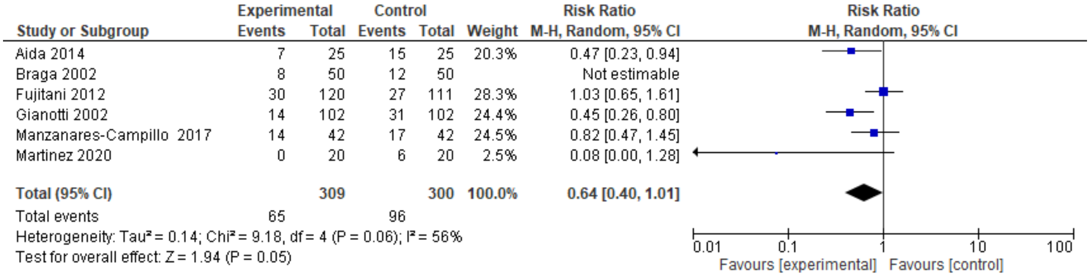

6/19 studies (609 participants).

Figure 5. Random effects meta-analysis of the risk ratio of pneumonia between immunonutrition (experimental) and usual care (control)

Study or Subgroup	Experimental Events	Experimental Total	Control Events	Control Total	Weight	Risk Ratio M-H, Random, 95% CI
Aida 2014	1	25	0	25	8.8%	3.00 [0.13, 70.30]
Desai 2016	2	40	8	40	24.8%	0.25 [0.06, 1.11]
Fujitani 2012	5	120	0	111	10.2%	10.18 [0.57, 182.04]
Gade 2016	0	19	4	19	10.4%	0.11 [0.01, 1.93]
Gunerhan 2009	2	13	4	11	24.6%	0.42 [0.09, 1.89]
Kaya 2016	1	31	1	27	11.2%	0.87 [0.06, 13.27]
Martinez 2020	0	20	3	20	10.1%	0.14 [0.01, 2.60]
**Total (95% CI)**		**268**		**253**	**100.0%**	**0.52 [0.18, 1.44]**
Total events	11		20			

Heterogeneity: Tau² = 0.54; Chi² = 8.43, df = 6 (P = 0.21); I² = 29%
Test for overall effect: Z = 1.26 (P = 0.21)

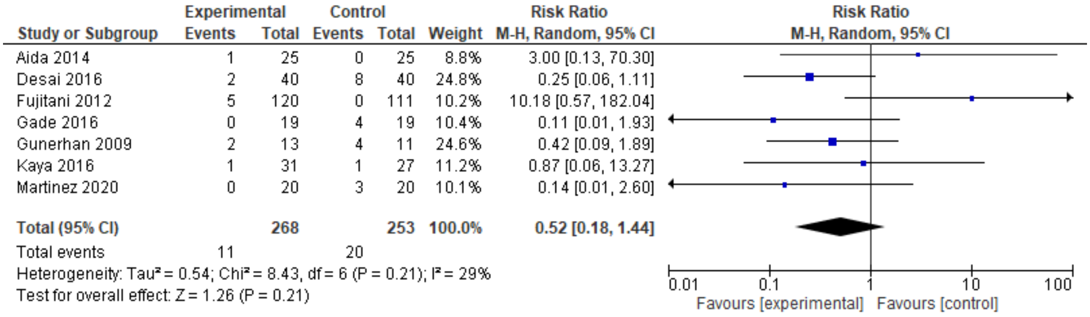

7/19 studies (521 participants).1 abstract (Desai et al., 2016) included.

Figure 6. Random effects meta-analysis of the risk ratio of wound infection between immunonutrition (experimental) and usual care (control)

Study or Subgroup	Experimental Events	Experimental Total	Control Events	Control Total	Weight	Risk Ratio M-H, Random, 95% CI
Aida 2014	3	25	7	25	7.2%	0.43 [0.12, 1.47]
Barker 2013	5	46	10	49	10.8%	0.53 [0.20, 1.44]
Desai 2016	5	40	13	40	12.2%	0.38 [0.15, 0.98]
Fujitani 2012	27	120	23	111	37.6%	1.09 [0.66, 1.78]
Gade 2016	3	19	8	19	8.0%	0.38 [0.12, 1.20]
Gianotti 2002	7	102	11	102	12.8%	0.64 [0.26, 1.58]
Gunerhan 2009	5	13	3	9	8.2%	1.15 [0.36, 3.65]
Russell 2019	2	17	2	15	3.3%	0.88 [0.14, 5.52]
**Total (95% CI)**		**382**		**370**	**100.0%**	**0.71 [0.51, 0.99]**
Total events	57		77			

Heterogeneity: Tau² = 0.02; Chi² = 7.44, df = 7 (P = 0.38); I² = 6%
Test for overall effect: Z = 2.00 (P = 0.05)

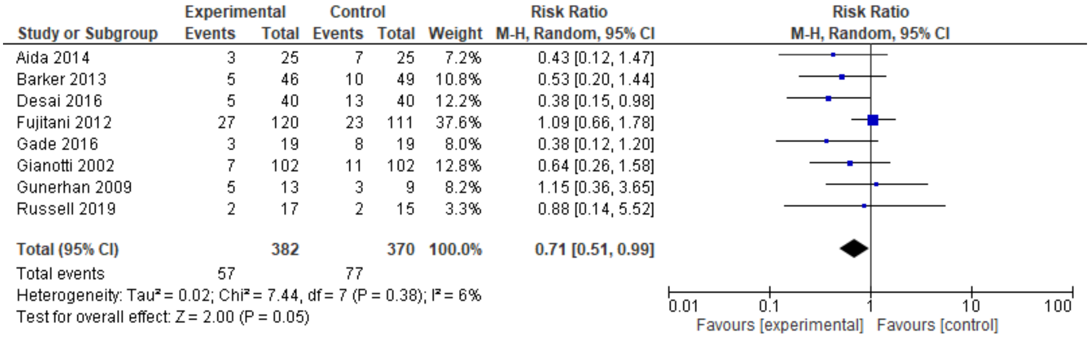

8/19 RCTs (752 participants); 1 abstract (Desai et al., 2016) included.

ORAL NUTRITIONAL SUPPLEMENTS (ONS)

Figure 7. Random effects meta-analysis of the risk ratio of mortality between ONS (experimental) and usual care (control).

Study or Subgroup	Experimental Events	Experimental Total	Control Events	Control Total	Weight	Risk Ratio M-H, Random, 95% CI
Burden 2011	3	54	0	62	22.2%	8.02 [0.42, 151.83]
Burden 2017	1	55	4	45	33.0%	0.20 [0.02, 1.77]
Kikutchi 2016	1	39	0	38	19.9%	2.92 [0.12, 69.64]
MacFie 2000	1	24	1	25	24.8%	1.04 [0.07, 15.73]
**Total (95% CI)**		**172**		**170**	**100.0%**	**1.18 [0.23, 6.11]**
Total events	6		5			

Heterogeneity: Tau² = 0.93; Chi² = 4.47, df = 3 (P = 0.22); I² = 33%
Test for overall effect: Z = 0.19 (P = 0.85)

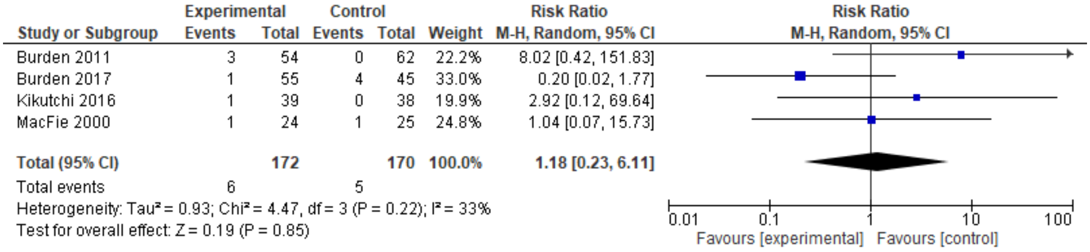

4/6 ONS studies (342 participants).

Figure 8. Random effects meta-analysis of the mean difference in length of hospital stay (LoS) between oral nutritional supplements (experimental) and usual care (control).

Study or Subgroup	Experimental Mean	Experimental SD	Experimental Total	Control Mean	Control SD	Control Total	Weight	Mean Difference IV, Random, 95% CI
Burden 2011	7.73	4.81	50	7	4.45	42	19.5%	0.73 [-1.16, 2.62]
Kikutchi 2016	13.7	11.2	39	12.7	13.4	38	2.3%	1.00 [-4.52, 6.52]
MacFie 2000	12	7.37	24	13	7.5	25	4.0%	-1.00 [-5.16, 3.16]
Nagata 2013	12.9	3.5	11	12.4	4	12	7.4%	0.50 [-2.57, 3.57]
Smedley 2004	12.8	4.5	41	14.1	6.6	44	12.3%	-1.30 [-3.69, 1.09]
Zhao 2018	7	1.48	33	8	2.97	33	54.5%	-1.00 [-2.13, 0.13]
**Total (95% CI)**			**198**			**194**	**100.0%**	**-0.54 [-1.38, 0.29]**

Heterogeneity: Tau² = 0.00; Chi² = 3.54, df = 5 (P = 0.62); I² = 0%
Test for overall effect: Z = 1.27 (P = 0.20)

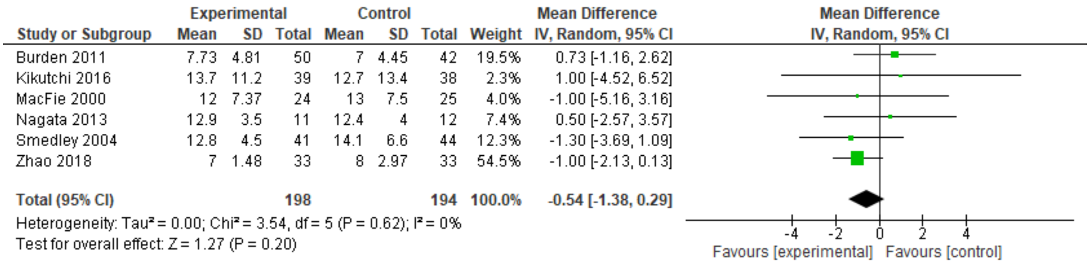

6/7studies (392 participants).

WEIGHT LOSS INTERVENTIONS

Although three weight loss intervention studies reported on mortality, two of these, Van Nieuwenhove et al and Barth et al.,2019,, could not be estimated as there were no deaths in either group. Thus, a meta-analysis was not possible.

Figure 9. Random effects meta-analysis of the mean difference in length of hospital stay (LoS) between weight loss interventions (experimental) and usual care (control).

	Experimental			Control				Mean Difference
Study or Subgroup	Mean	SD	Total	Mean	SD	Total	Weight	IV, Random, 95% CI
Alami 2007	3.4	1.6	26	3.1	1.1	25	22.2%	0.30 [-0.45, 1.05]
Barth 2019	5.37	0.77	30	4.38	0.79	30	27.5%	0.99 [0.60, 1.38]
Chakravartty 2019	2.67	0.4	10	4.56	3.83	10	6.5%	-1.89 [-4.28, 0.50]
Grundman 2018	10.29	1.91	36	10	2.97	40	16.8%	0.29 [-0.82, 1.40]
Hollis 2020	1.2	0.5	20	1.36	0.7	14	27.0%	-0.16 [-0.59, 0.27]
**Total (95% CI)**			**122**			**119**	**100.0%**	**0.22 [-0.46, 0.91]**

Heterogeneity: Tau² = 0.40; Chi² = 18.82, df = 4 (P = 0.0009); I² = 79%
Test for overall effect: Z = 0.63 (P = 0.53)

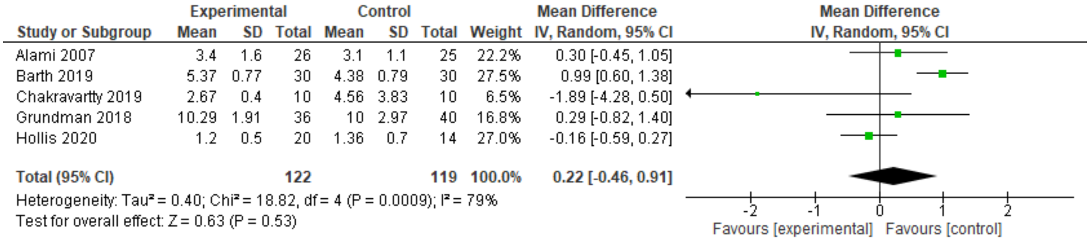

5/10 studies (241 participants).

PRE/PROBIOTICS

Figure 10. Random effects meta-analysis of the risk ratio of mortality between pre/probiotic interventions (experimental) and usual care (control).

	Experimental		Control			Risk Ratio
Study or Subgroup	Events	Total	Events	Total	Weight	M-H, Random, 95% CI
Anderson 2004	9	72	5	65	61.8%	1.63 [0.57, 4.60]
Consoli 2016	0	15	0	15		Not estimable
Grat 2018	0	21	1	23	18.0%	0.36 [0.02, 8.47]
Polakowski 2019	0	36	3	37	20.2%	0.15 [0.01, 2.74]
Zhang 2012	0	30	0	30		Not estimable
**Total (95% CI)**		**174**		**170**	**100.0%**	**0.76 [0.17, 3.42]**
Total events	9		9			

Heterogeneity: Tau² = 0.67; Chi² = 2.95, df = 2 (P = 0.23); I² = 32%
Test for overall effect: Z = 0.35 (P = 0.72)

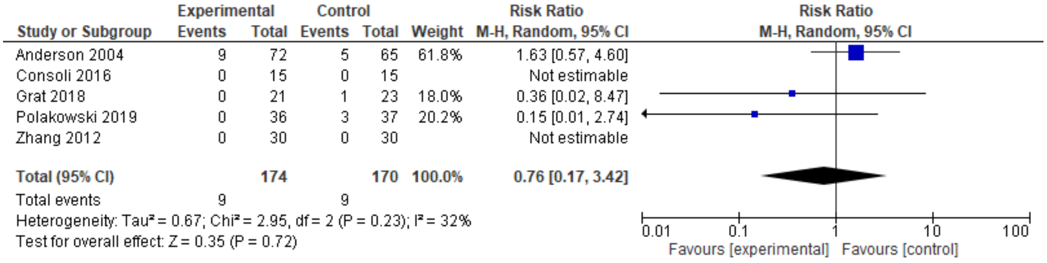

5/6 studies (244 participants).

Figure 11. Random effects meta-analysis of the risk ratio of total PO infective complications between pre/probiotic interventions (experimental) and usual care (control).

	Experimental		Control			Risk Ratio
Study or Subgroup	Events	Total	Events	Total	Weight	M-H, Random, 95% CI
Anderson 2004	23	72	20	65	47.6%	1.04 [0.63, 1.71]
Consoli 2016	2	15	7	18	30.6%	0.34 [0.08, 1.41]
Grat 2018	1	21	8	23	21.8%	0.14 [0.02, 1.00]
**Total (95% CI)**		**108**		**106**	**100.0%**	**0.48 [0.14, 1.62]**
Total events	26		35			

Heterogeneity: Tau² = 0.76; Chi² = 5.84, df = 2 (P = 0.05); I² = 66%
Test for overall effect: Z = 1.19 (P = 0.23)

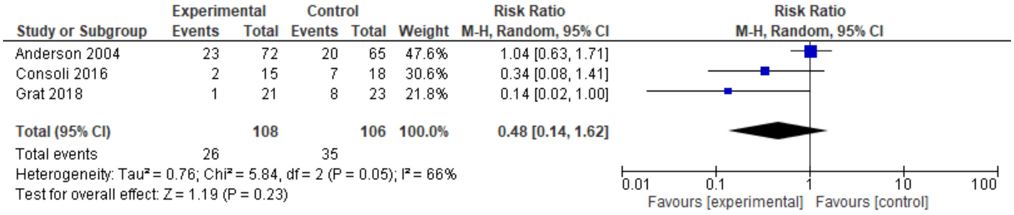

3/6 studies (214 participants).

Perry R, *et al. BMJ Open* 2022; 11:e050806. doi: 10.1136/bmjopen-2021-050806

Sensitivity analyses (removing studies at high risk of bias)

IMMUNONUTRITION

Figure 12. Random effects meta-analysis of the risk ratio of mortality between immunonutrition (experimental) and usual care (control) with high risk of bias studies removed.

Study or Subgroup	Experimental Events	Experimental Total	Control Events	Control Total	Weight	Risk Ratio M-H, Fixed, 95% CI
Barker 2013	0	46	1	45	37.8%	0.33 [0.01, 7.80]
Fujitani 2012	0	120	0	111		Not estimable
Sufit 2012	0	7	2	7	62.2%	0.20 [0.01, 3.54]
**Total (95% CI)**		**173**		**163**	**100.0%**	**0.25 [0.03, 2.05]**
Total events	0		3			

Heterogeneity: Chi² = 0.05, df = 1 (P = 0.82); I² = 0%
Test for overall effect: Z = 1.29 (P = 0.20)

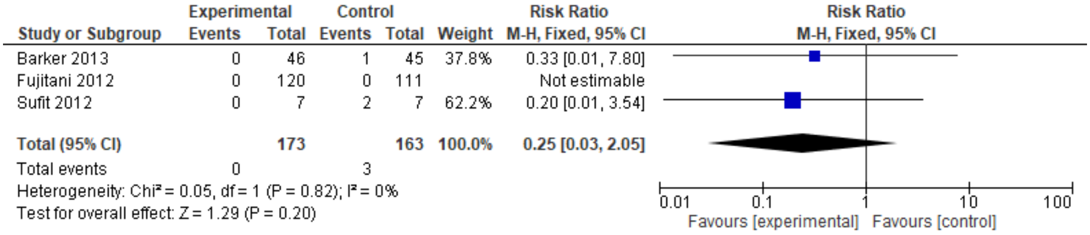

3 studies (790 participants) included and 8 studies excluded (Aida et al., 2014; Braga et al., 2002; Gade et al., 2016; Gianotti et al., 2002; Martinez et al., 2020; Nakamura et al., 2005; Pronio et al., 2008; Tumas et al., 2020).

Figure 13. Random effects meta-analysis of the mean difference in length of stay between immunonutrition (experimental) and usual care (control) with high risk of bias studies removed.

Study or Subgroup	Experimental Mean	Experimental SD	Experimental Total	Control Mean	Control SD	Control Total	Weight	Mean Difference IV, Random, 95% CI
Barker 2013	7.1	4.1	46	8.8	6.5	49	50.7%	-1.70 [-3.87, 0.47]
Fujitani 2012	24.01	15.89	120	23.88	16.84	111	13.4%	0.13 [-4.10, 4.36]
Russell 2019	16.62	22.72	17	12.98	15.13	15	1.4%	3.64 [-9.60, 16.88]
Sufit 2012	6	0.8	7	7	3.2	6	34.6%	-1.00 [-3.63, 1.63]
**Total (95% CI)**			**190**			**181**	**100.0%**	**-1.14 [-2.69, 0.41]**

Heterogeneity: Tau² = 0.00; Chi² = 1.11, df = 3 (P = 0.77); I² = 0%
Test for overall effect: Z = 1.45 (P = 0.15)

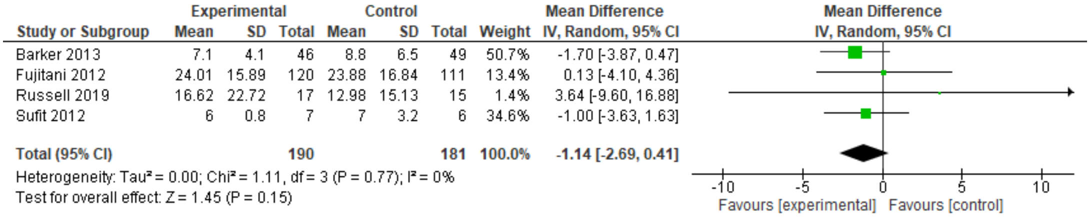

4 studies (371 participants) included and 8 studies excluded (Braga et al., 2002; Desai et al., 2016; Gade et al., 2016; Gianotti et al., 2002; Gunerhan et al., 2009; Marzanares-Campillo et al., 2017; Martinez et al., 2020; Nakamura et al., 2005; Pronio et al., 2008; Tumas et al., 2020).

Perry R, *et al. BMJ Open* 2022; 11:e050806. doi: 10.1136/bmjopen-2021-050806

Sensitivity analyses (removing studies with imputed results)

IMMUNONUTRITION

Figure 14. Random effects meta-analysis of the mean difference in length of stay between immunonutrition (experimental) and usual care (control) with imputed results removed.

	Experimental			Control				Mean Difference
Study or Subgroup	Mean	SD	Total	Mean	SD	Total	Weight	IV, Random, 95% CI
Barker 2013	7.1	4.1	46	8.8	6.5	49	17.5%	-1.70 [-3.87, 0.47]
Braga 2002	13.2	3.5	50	15.3	4.1	50	37.0%	-2.10 [-3.59, -0.61]
Gianotti 2002	11.6	4.7	102	14	7.7	102	27.0%	-2.40 [-4.15, -0.65]
Gunerhan 2009	16.5	14.8	13	12	3.7	9	1.2%	4.50 [-3.90, 12.90]
Manzanares-Campillo 2017	12.7	8.3	42	13.1	10.8	42	4.9%	-0.40 [-4.52, 3.72]
Nakamura 2005	49	18.3	12	46.1	15	14	0.5%	2.90 [-10.10, 15.90]
Sufit 2012	6	0.8	7	7	3.2	6	12.0%	-1.00 [-3.63, 1.63]
**Total (95% CI)**			**272**			**272**	**100.0%**	**-1.79 [-2.70, -0.89]**

Heterogeneity: Tau² = 0.00; Chi² = 4.08, df = 6 (P = 0.67); I² = 0%
Test for overall effect: Z = 3.87 (P = 0.0001)

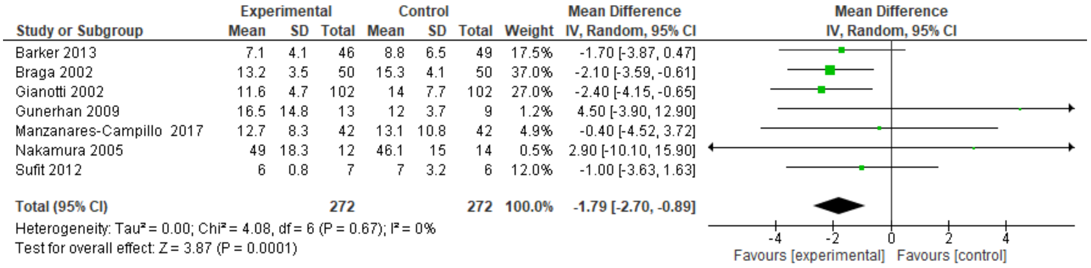

7 studies included (544 participants) and 4 studies excluded (Pronio et al., 2008, Fujitani et al., 2012, Gade et al.,2016, , Russell et al., 2019)

ORAL NUTRITIONAL SUPPLEMENTS (ONS)

Figure 15. Random effects meta-analysis of the mean difference in length of stay between ONS (experimental) and usual care (control) with imputed results removed.

	Experimental			Control				Mean Difference
Study or Subgroup	Mean	SD	Total	Mean	SD	Total	Weight	IV, Random, 95% CI
Kikutchi 2016	13.7	11.2	39	12.7	13.4	38	10.4%	1.00 [-4.52, 6.52]
Nagata 2013	12.9	3.5	11	12.4	4	12	33.8%	0.50 [-2.57, 3.57]
Smedley 2004	12.8	4.5	41	14.1	6.6	44	55.8%	-1.30 [-3.69, 1.09]
**Total (95% CI)**			**91**			**94**	**100.0%**	**-0.45 [-2.23, 1.33]**

Heterogeneity: Tau² = 0.00; Chi² = 1.12, df = 2 (P = 0.57); I² = 0%
Test for overall effect: Z = 0.50 (P = 0.62)

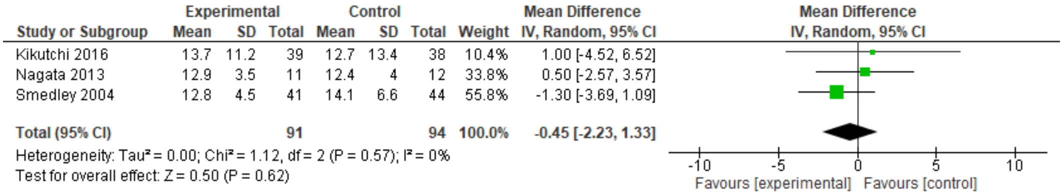

3 studies (185 participants) incuded and 3 studies excluded (Burden et al., 2017, MacFie et al., 2000, Zhao et al., 2018).

Subgroup analyses (type of surgery)

IMMUNONUTRITION (CANCER SURGERY)

Figure 16. Random effects meta-analysis of the risk ratio of mortality between immunonutrition (experimental) and usual care (control) for those undergoing cancer surgery.

Study or Subgroup	Experimental Events	Experimental Total	Control Events	Control Total	Weight	Risk Ratio M-H, Fixed, 95% CI
Braga 2002	1	50	2	50	40.6%	0.50 [0.05, 5.34]
Fujitani 2012	0	120	0	111		Not estimable
Gade 2016	0	19	1	19	30.4%	0.33 [0.01, 7.70]
Gianotti 2002	1	102	1	102	20.3%	1.00 [0.06, 15.77]
Tumas 2020	1	30	0	40	8.7%	3.97 [0.17, 94.13]
**Total (95% CI)**		**321**		**322**	**100.0%**	**0.85 [0.24, 3.05]**
Total events	3		4			

Heterogeneity: Chi² = 1.46, df = 3 (P = 0.69); I² = 0%
Test for overall effect: Z = 0.24 (P = 0.81)

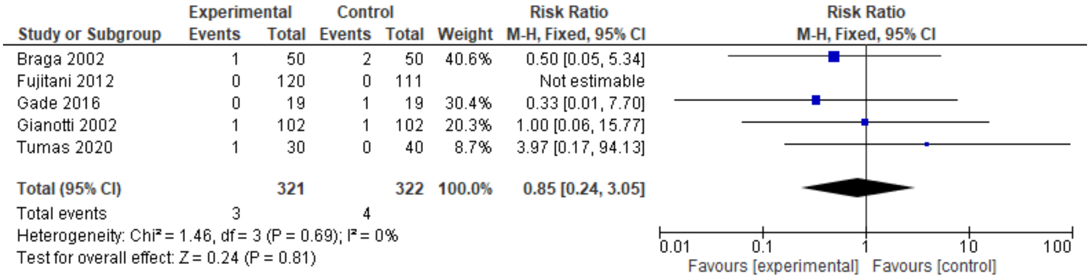

5 studies (643 participants) included and 6 studies excluded (Aida et al., 2014, Barker et al., 2013, Martinez et al., 2020, Nakamura et al., 2006, Pronio et al., 2008, Sufit et al., 2012).

Figure 17. Random effects meta-analysis of the mean difference in length of hospital stay (LoS) between immunonutrition (experimental) and usual care (control) for those undergoing cancer surgery.

Study or Subgroup	Experimental Mean	Experimental SD	Experimental Total	Control Mean	Control SD	Control Total	Weight	Mean Difference IV, Random, 95% CI
Braga 2002	13.2	3.5	50	15.3	4.1	50	47.2%	-2.10 [-3.59, -0.61]
Fujitani 2012	24.01	15.89	120	23.08	16.84	111	5.9%	0.93 [-3.30, 5.16]
Gade 2016	14.02	8.57	19	17.05	7.09	19	4.2%	-3.03 [-8.03, 1.97]
Gianotti 2002	11.6	4.7	102	14	7.7	102	34.4%	-2.40 [-4.15, -0.65]
Gunerhan 2009	16.5	14.8	13	12	3.7	9	1.5%	4.50 [-3.90, 12.90]
Manzanares-Campillo 2017	12.7	8.3	42	13.1	10.8	42	6.2%	-0.40 [-4.52, 3.72]
Russell 2019	16.62	22.72	17	12.98	15.13	15	0.6%	3.64 [-9.60, 16.88]
**Total (95% CI)**			**363**			**348**	**100.0%**	**-1.83 [-2.85, -0.80]**

Heterogeneity: Tau² = 0.00; Chi² = 5.69, df = 6 (P = 0.46); I² = 0%
Test for overall effect: Z = 3.48 (P = 0.0005)

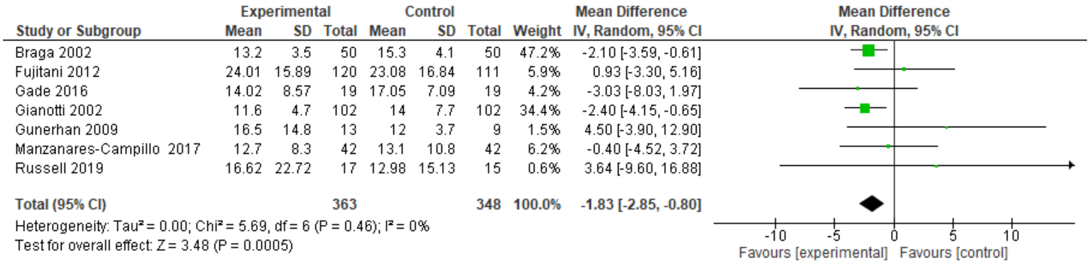

7 studies (711 participants) included and 6 studies excuded (Barker et al., 2013, Desai et al., 2016, Martinez et al., 2020, Nakamura et al., 2005, Pronio et al., 2019, Sufit et al., 2012).

ORAL NUTRITIONAL SUPPLEMENTS (ONS) (CANCER SURGERY)

Figure 18. Random effects meta-analysis of the risk ratio of mortality between ONS (experimental) and usual care (control) for those undergoing cancer surgery.

Study or Subgroup	Experimental Events	Experimental Total	Control Events	Control Total	Weight	Risk Ratio M-H, Random, 95% CI
Burden 2011	3	54	0	62	31.1%	8.02 [0.42, 151.83]
Burden 2017	1	55	4	45	40.1%	0.20 [0.02, 1.77]
Kikutchi 2016	1	39	0	39	28.8%	3.00 [0.13, 71.46]
**Total (95% CI)**		**148**		**146**	**100.0%**	**1.39 [0.13, 14.72]**
Total events	5		4			

Heterogeneity: Tau² = 2.41; Chi² = 4.49, df = 2 (P = 0.11); I² = 55%
Test for overall effect: Z = 0.27 (P = 0.78)

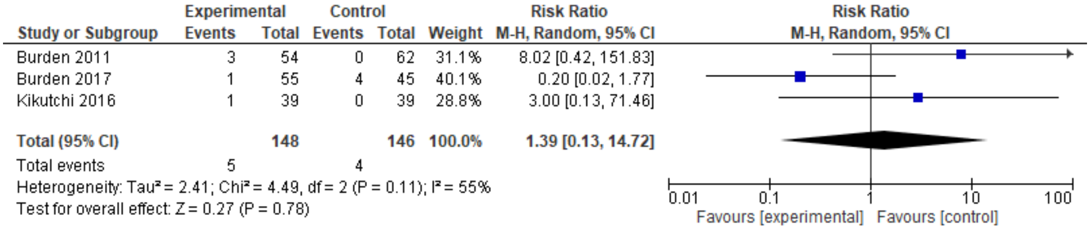

3 studies (294 participants) included and 1 studies excluded (Macfie et al., 2000).

Subgroup analysis (studies published before and after 2010)

There were less than 2 studies for each intervention and outcome that were published before 2010, therefore a meta analysis for this subgroup was not possible for any intervention or outcome.

IMMUNONUTRITION (STUDIES PUBLISHED AFTER 2010)

Figure 19. Random effects meta-analysis of the risk ratio of mortality between immunonutrition (experimental) and usual care (control) in studies published after 2010.

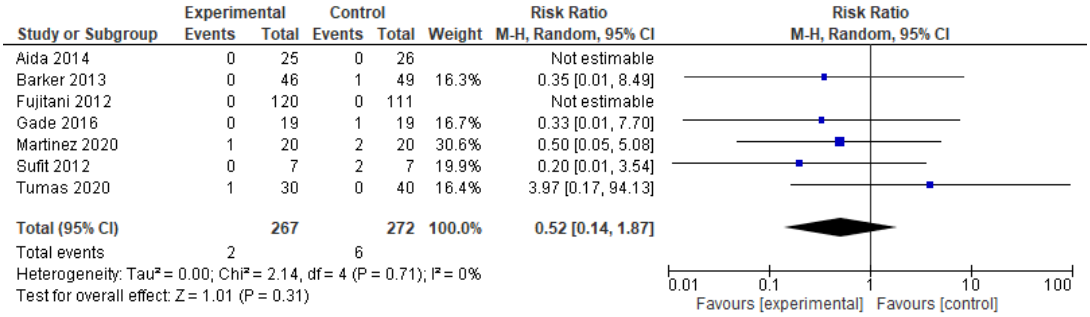

7 studies (539 participants) included and 4 studies excluded (Braga et al., 2002, Gianotti et al., 2002, Nakaumra et al., 2005, Ponio et al., 2008).

Figure 20. Random effects meta-analysis of the mean difference in length of hospital stay (LoS) between immunonutrition (experimental) and usual care (control) in studies published after 2010.

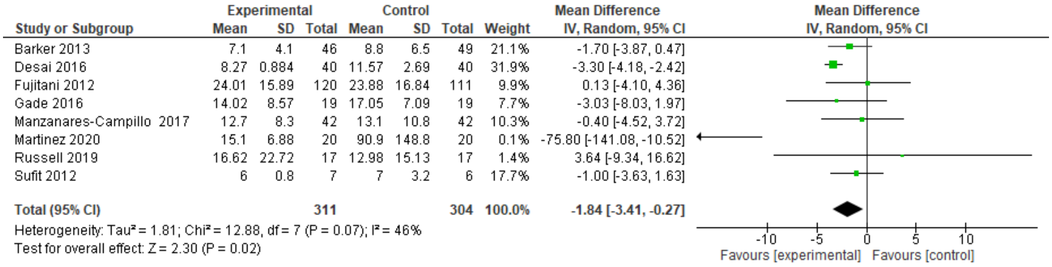

7 studies (615 participants) included and X studies included (Braga et al., 2002, Gianotti et al., 2002, Gunerhan et al., 2009, Nakamura et al., 2005, Pronio et al., 2008**)**.

Figure 21. Random effects meta-analysis of the risk ratio of total postoperative complications between immunonutrition (experimental) and usual care (control) in studies published after 2010.

Study or Subgroup	Experimental Events	Experimental Total	Control Events	Control Total	Weight	Risk Ratio M-H, Random, 95% CI
Barker 2013	8	46	14	49	13.9%	0.61 [0.28, 1.31]
Fujitani 2012	37	120	29	111	29.2%	1.18 [0.78, 1.78]
Kaya 2016	6	31	12	27	12.3%	0.44 [0.19, 1.00]
Mikagi 2010	1	13	3	13	2.3%	0.33 [0.04, 2.80]
Tumas 2020	24	30	33	40	42.3%	0.97 [0.77, 1.22]
**Total (95% CI)**		**240**		**240**	**100.0%**	**0.85 [0.61, 1.19]**
Total events	76		91			

Heterogeneity: Tau² = 0.05; Chi² = 6.97, df = 4 (P = 0.14); I² = 43%
Test for overall effect: Z = 0.95 (P = 0.34)

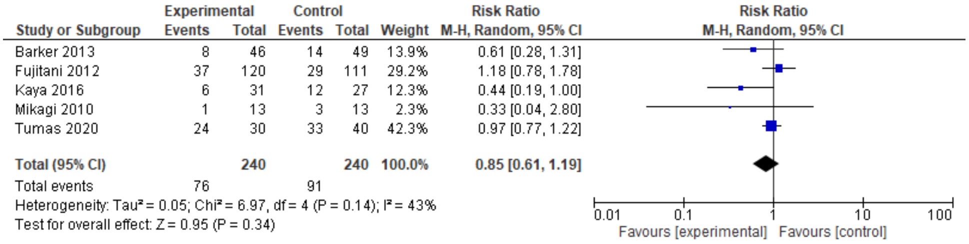

5 studies (480 participants) included and 2 studies included (Gianotti et al., 2002 and Pronio et al., 2008).

Figure 22. Random effects meta-analysis of the risk ratio of total infective complications between immunonutrition (experimental) and usual care (control) in studies published after 2010.

Study or Subgroup	Experimental Events	Experimental Total	Control Events	Control Total	Weight	Risk Ratio M-H, Random, 95% CI
Aida 2014	7	25	15	25	26.6%	0.47 [0.23, 0.94]
Fujitani 2012	30	120	27	111	37.8%	1.03 [0.65, 1.61]
Manzanares-Campillo 2017	14	42	17	42	32.5%	0.82 [0.47, 1.45]
Martinez 2020	0	20	6	20	3.1%	0.08 [0.00, 1.28]
**Total (95% CI)**		**207**		**198**	**100.0%**	**0.71 [0.43, 1.19]**
Total events	51		65			

Heterogeneity: Tau² = 0.13; Chi² = 6.20, df = 3 (P = 0.10); I² = 52%
Test for overall effect: Z = 1.28 (P = 0.20)

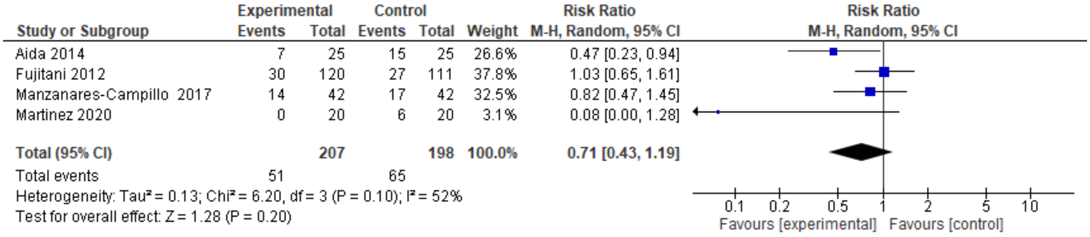

4 studies (405 participants) included and 2 studies excluded (Gianotti et al., 2002 and Braga et al., 2002).

Figure 23. Random effects meta-analysis of the risk ratio of penumonia between immunonutrition (experimental) and usual care (control) in studies published after 2010.

Study or Subgroup	Experimental Events	Experimental Total	Control Events	Control Total	Weight	Risk Ratio M-H, Random, 95% CI
Aida 2014	1	25	0	25	12.8%	3.00 [0.13, 70.30]
Desai 2016	2	40	8	40	24.7%	0.25 [0.06, 1.11]
Fujitani 2012	5	120	0	111	14.2%	10.18 [0.57, 182.04]
Gade 2016	0	19	4	19	14.4%	0.11 [0.01, 1.93]
Kaya 2016	1	31	4	11	19.7%	0.09 [0.01, 0.71]
Martinez 2020	0	20	3	20	14.2%	0.14 [0.01, 2.60]
**Total (95% CI)**		**255**		**226**	**100.0%**	**0.39 [0.09, 1.64]**
Total events	9		19			

Heterogeneity: Tau² = 1.59; Chi² = 10.35, df = 5 (P = 0.07); I² = 52%
Test for overall effect: Z = 1.28 (P = 0.20)

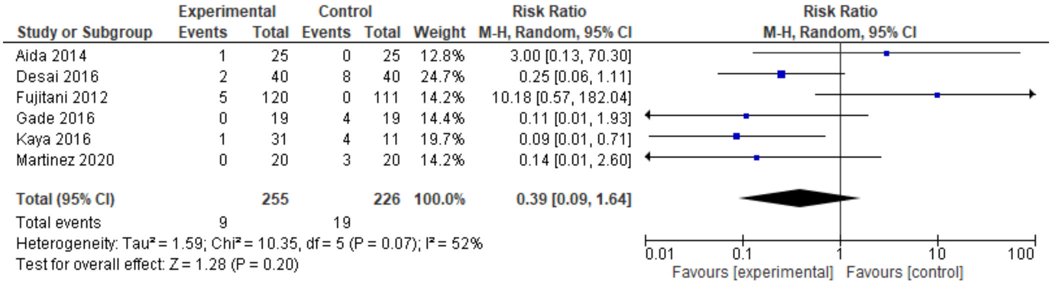

6 studies (481 participants) included ad 1 study excluded (Gunerhan et al., 2009). 1 abstract (Desai et al., 2016) included.

Figure 24. Random effects meta-analysis of the risk ratio of wound infection between immunonutrition (experimental) and usual care (control) in studies published after 2010.

Study or Subgroup	Experimental Events	Experimental Total	Control Events	Control Total	Weight	Risk Ratio M-H, Random, 95% CI
Aida 2014	1	25	0	25	12.8%	3.00 [0.13, 70.30]
Desai 2016	2	40	8	40	24.7%	0.25 [0.06, 1.11]
Fujitani 2012	5	120	0	111	14.2%	10.18 [0.57, 182.04]
Gade 2016	0	19	4	19	14.4%	0.11 [0.01, 1.93]
Kaya 2016	1	31	4	11	19.7%	0.09 [0.01, 0.71]
Martinez 2020	0	20	3	20	14.2%	0.14 [0.01, 2.60]
**Total (95% CI)**		**255**		**226**	**100.0%**	**0.39 [0.09, 1.64]**
Total events	9		19			

Heterogeneity: Tau² = 1.59; Chi² = 10.35, df = 5 (P = 0.07); I² = 52%
Test for overall effect: Z = 1.28 (P = 0.20)

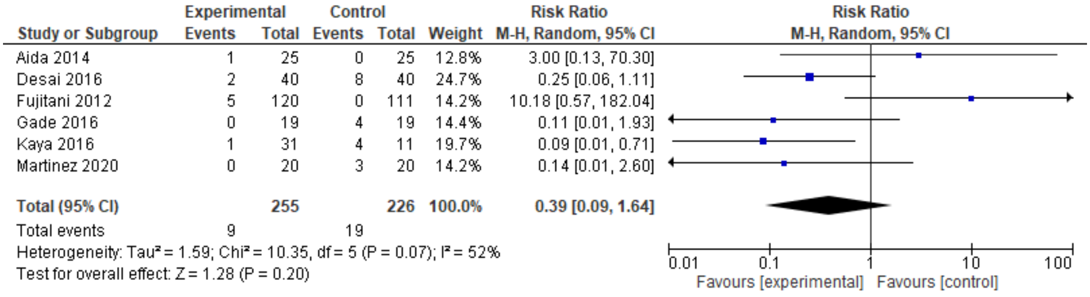

6 studies (481 participants) included and 2 studies excluded (Gunerhan et al., 2009 and Gianotti etal., 2002). 1 abstract (Desai et al., 2016) included.

ORAL NUTRITIONAL SUPPLEMENTS (ONS) (STUDIES PUBLISHED AFTER 2010)

Figure 25. Random effects meta-analysis of the risk ratio of mortality between ONS (experimental) and usual care (control) in studies published after 2010.

Study or Subgroup	Experimental Events	Experimental Total	Control Events	Control Total	Weight	Risk Ratio M-H, Random, 95% CI
Burden 2011	3	54	0	62	31.1%	8.02 [0.42, 151.83]
Burden 2017	1	55	4	45	40.1%	0.20 [0.02, 1.77]
Kikutchi 2016	1	39	0	38	28.8%	2.92 [0.12, 69.64]
**Total (95% CI)**		**148**		**145**	**100.0%**	**1.38 [0.13, 14.52]**
Total events	5		4			

Heterogeneity: Tau² = 2.39; Chi² = 4.47, df = 2 (P = 0.11); I² = 55%
Test for overall effect: Z = 0.27 (P = 0.79)

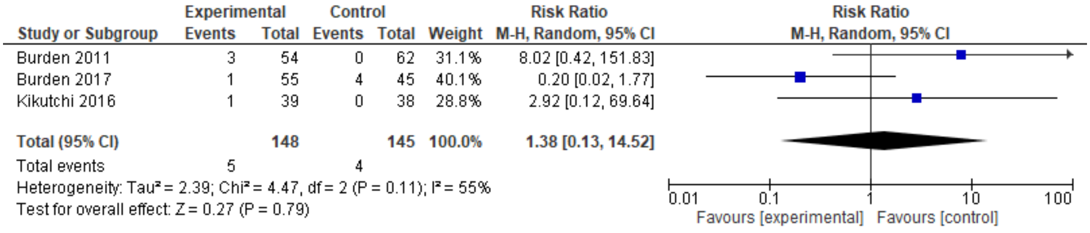

3 studies (293 participants) included and 1 studies excluded (Macfie et al., 2000).

Figure 26. Random effects meta-analysis of the mean difference in length of hospital stay (LoS) between ONS (experimental) and usual care (control) in studies published after 2010.

	Experimental			Control				Mean Difference
Study or Subgroup	Mean	SD	Total	Mean	SD	Total	Weight	IV, Random, 95% CI
Burden 2011	7.73	4.81	50	7	4.45	42	23.5%	0.73 [-1.16, 2.62]
Kikutchi 2016	13.7	11.2	39	12.7	13.4	38	2.8%	1.00 [-4.52, 6.52]
Nagata 2013	12.9	3.5	11	12.4	4	12	9.0%	0.50 [-2.57, 3.57]
Zhao 2018	7	1.48	33	8	2.97	33	64.6%	-1.00 [-2.13, 0.13]
**Total (95% CI)**			**133**			**125**	**100.0%**	**-0.40 [-1.33, 0.52]**

Heterogeneity: Tau² = 0.01; Chi² = 3.02, df = 3 (P = 0.39); I² = 1%
Test for overall effect: Z = 0.85 (P = 0.39)

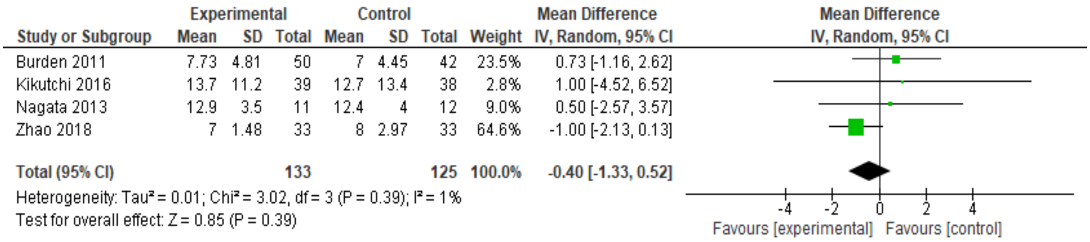

4 studies (258 participants) included and 2 studies excluded (Macfie et al., 2000, Smedley et al., 2004).

WEIGHT LOSS (STUDIES PUBLISHED AFTER 2010)

Figure 27. Random effects meta-analysis of the mean difference in length of hospital stay (LoS) between weight loss interventions (experimental) and usual care (control) in studies published after 2010.

	Experimental			Control				Mean Difference
Study or Subgroup	Mean	SD	Total	Mean	SD	Total	Weight	IV, Random, 95% CI
Barth 2019	5.37	0.77	30	4.38	0.79	30	33.7%	0.99 [0.60, 1.38]
Chakravartty 2019	2.67	0.4	10	4.56	3.83	10	9.9%	-1.89 [-4.28, 0.50]
Grundman 2018	10.29	1.91	36	10	2.97	40	23.0%	0.29 [-0.82, 1.40]
Hollis 2020	1.2	0.5	20	1.36	0.7	14	33.3%	-0.16 [-0.59, 0.27]
**Total (95% CI)**			**96**			**94**	**100.0%**	**0.16 [-0.72, 1.04]**

Heterogeneity: Tau² = 0.56; Chi² = 18.74, df = 3 (P = 0.0003); I² = 84%
Test for overall effect: Z = 0.35 (P = 0.72)

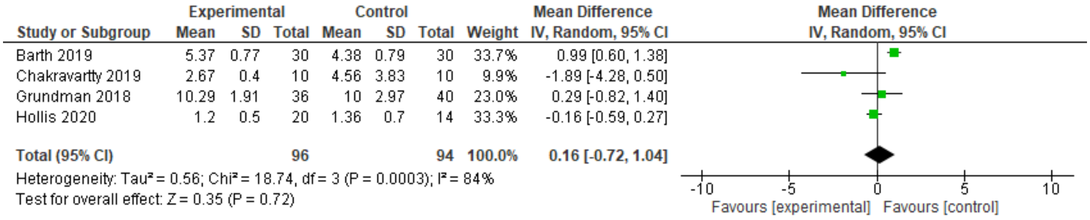

4 studies (190 participants) included and 1 study excluded (Alami et al., 2007).

PRE/PROBIOTICS (STUDIES PUBLISHED AFTER 2010)

Figure 28. Random effects meta-analysis of the risk ratio of mortality between pre/probiotics (experimental) and usual care (control) in studies published after 2010.

	Experimental		Control			Risk Ratio
Study or Subgroup	Events	Total	Events	Total	Weight	M-H, Random, 95% CI
Consoli 2016	0	15	0	15		Not estimable
Grat 2018	0	21	1	23	46.4%	0.36 [0.02, 8.47]
Polakowski 2019	0	36	3	37	53.6%	0.15 [0.01, 2.74]
Zhang 2012	0	30	0	30		Not estimable
**Total (95% CI)**		**102**		**105**	**100.0%**	**0.22 [0.03, 1.91]**
Total events	0		4			

Heterogeneity: Tau² = 0.00; Chi² = 0.17, df = 1 (P = 0.68); I² = 0%
Test for overall effect: Z = 1.37 (P = 0.17)

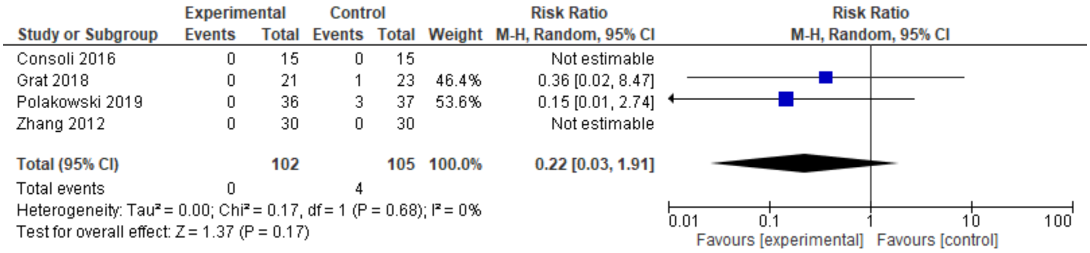

4 studies (207 participants) included and 1 study excluded (Anderson et al., 2004).

References

Aida, T., Furukawa, K., Suzuki, D., Shimizu, H., Yoshidome, H., Ohtsuka, M., . . . Miyazaki, M. 2014. Preoperative immunonutrition decreases postoperative complications by modulating prostaglandin E2 production and T-cell differentiation in patients undergoing pancreatoduodenectomy. *Surgery,* 155**,** 124-33.

Akbarzadeh, M., Eftekhari, M. H., Shafa, M., Alipour, S. & Hassanzadeh, J. 2016. Effects of a New Metabolic Conditioning Supplement on Perioperative Metabolic Stress and Clinical Outcomes: A Randomized, Placebo-Controlled Trial. *Iran Red Crescent Med J,* 18**,** e26207.

Alami, R. S., Morton, J. M., Schuster, R., Lie, J., Sanchez, B. R., Peters, A. & Curet, M. J. 2007. Is there a benefit to preoperative weight loss in gastric bypass patients? A prospective randomized trial. *Surg Obes Relat Dis,* 3**,** 141-5; discussion 145-6.

Anderson, A. D., Mcnaught, C. E., Jain, P. K. & Macfie, J. 2004. Randomised clinical trial of synbiotic therapy in elective surgical patients. *Gut,* 53**,** 241-5.

Barker, L. A., Gray, C., Wilson, L., Thomson, B. N., Shedda, S. & Crowe, T. C. 2013. Preoperative immunonutrition and its effect on postoperative outcomes in well-nourished and malnourished gastrointestinal surgery patients: a randomised controlled trial. *Eur J Clin Nutr,* 67**,** 802-7.

Barth, R. J., Jr., Mills, J. B., Suriawinata, A. A., Putra, J., Tosteson, T. D., Axelrod, D., . . . Kinlaw, W. B. 2019. Short-term Preoperative Diet Decreases Bleeding After Partial Hepatectomy: Results From a Multi-institutional Randomized Controlled Trial. *Ann Surg,* 269**,** 48-52.

Bottin, J., Balogun, B., Thomas, E., Fitzpatrick, J., Moorthy, K., Leeds, A., . . . Frost, G. 2014. Changes in body composition induced by pre-operative liquid low-calorie diet in morbid obese patients undergoing Roux-en-Y gastric bypass. *Obesity Reviews,* 15**,** 129-130.

Braga, M., Gianotti, L., Vignali, A. & Carlo, V. D. 2002. Preoperative oral arginine and n-3 fatty acid supplementation improves the immunometabolic host response and outcome after colorectal resection for cancer. *Surgery,* 132**,** 805-14.

Burden, S. T., Gibson, D. J., Lal, S., Hill, J., Pilling, M., Soop, M., . . . Todd, C. 2017. Pre-operative oral nutritional supplementation with dietary advice versus dietary advice alone in weight-losing patients with colorectal cancer: single-blind randomized controlled trial. *J Cachexia Sarcopenia Muscle,* 8**,** 437-446.

Burden, S. T., Hill, J., Shaffer, J. L., Campbell, M. & Todd, C. 2011. An unblinded randomised controlled trial of preoperative oral supplements in colorectal cancer patients. *J Hum Nutr Diet,* 24**,** 441-8.

Chakravartty, S., Vivian, G., Mullholland, N., Shaikh, H., Mcgrath, J., Sidhu, P. S., . . . Patel, A. G. 2019. Preoperative liver shrinking diet for bariatric surgery may impact wound healing: a randomized controlled trial. *Surg Obes Relat Dis,* 15**,** 117-125.

Chuah, L., Miras, A., Noon, J., Jackson, S., Olbers, T. & Le Roux, C. 2014. Does intensive preoperative and postoperative glucose management influence glycaemic outcome of Roux-en-Y gastric bypass surgery? *Obesity Reviews,* 15**,** 136.

Consoli, M. L., Da Silva, R. S., Nicoli, J. R., Bruña-Romero, O., Da Silva, R. G., De Vasconcelos Generoso, S. & Correia, M. I. 2016. Randomized Clinical Trial: Impact of Oral Administration of Saccharomyces boulardii on Gene Expression of Intestinal Cytokines in Patients Undergoing Colon Resection. *JPEN J Parenter Enteral Nutr,* 40**,** 1114-1121.

Dambrauskas, Z., Maleckas, A., Van Nieuwenhove, Y. G. E. U. & Thorell, A. 2013. The effects of short-term preoperative very low calorie diet (VLCD) on long-term outcomes after laparoscopic Roux-en-Y gastric bypass for morbid obesity. 2013.

Desai, S., Vijayashree, N., Merina, A., Benjamin, N. & Rajan, R. 2016. Effect of preoperative L-glutamine supplementation on primary outcomes in elective cardiac surgery. *European heart journal,* 37**,** 729-.

Elrefai, M. 2017. Value of low calorie diet before sleeve gastrectomy: Prospectieve randomised study. pre-operative management. *Obesity Surgery,* 27 (1 Supplement 1)**,** 813.

Elrefai, M. 2019. Value of preoperative diet before sleeve gastrectomy: A prospective randomized study. *Obesity Surgery,* 29 (5 Supplement)**,** 860.

Faria, S. L., Faria, O. P., De Almeida Cardeal, M. & Ito, M. K. 2015. Effects of a very low calorie diet in the preoperative stage of bariatric surgery: a randomized trial. *Surg Obes Relat Dis,* 11**,** 230-7.

Flynn, M. B. & Leightty, F. F. 1987. Preoperative outpatient nutritional support of patients with squamous cancer of the upper aerodigestive tract. *Am J Surg,* 154**,** 359-62.

Frost, G., Keogh, B., Smith, D., Akinsanya, K. & Leeds, A. 1996. The effect of low-glycemic carbohydrate on insulin and glucose response in vivo and in vitro in patients with coronary heart disease. *Metabolism,* 45**,** 669-72.

Frost, G. S., Keogh, B. E., Smith, D., Leeds, A. R. & Dornhorst, A. 1998. Reduced adipocyte insulin sensitivity in Caucasian and Asian subjects with coronary heart disease. *DIABETIC MEDICINE,* 15**,** 1003-1009.

Fujitani, K., Tsujinaka, T., Fujita, J., Miyashiro, I., Imamura, H., Kimura, Y., . . . Furukawa, H. 2012. Prospective randomized trial of preoperative enteral immunonutrition followed by elective total gastrectomy for gastric cancer. *Br J Surg,* 99**,** 621-9.

Gade, J., Levring, T., Hillingsø, J., Hansen, C. P. & Andersen, J. R. 2016. The Effect of Preoperative Oral Immunonutrition on Complications and Length of Hospital Stay After Elective Surgery for Pancreatic Cancer–A Randomized Controlled Trial. *Nutrition and Cancer,* 68**,** 225-233.

Gianotti, L., Braga, M., Nespoli, L., Radaelli, G., Beneduce, A. & Di Carlo, V. 2002. A randomized controlled trial of preoperative oral supplementation with a specialized diet in patients with gastrointestinal cancer. *Gastroenterology,* 122**,** 1763-70.

Grat, M., Wronka, K. M., Lewandowski, Z., Grat, K., Krasnodebski, M., Stypulkowski, J., . . . Krawczyk, M. 2017. Effects of continuous use of probiotics before liver transplantation: A randomized, double-blind, placebo-controlled trial. *Clin Nutr,* 36**,** 1530-1539.

Grundmann, F., Müller, R. U., Reppenhorst, A., Hülswitt, L., Späth, M. R., Kubacki, T., . . . Burst, V. 2018. Preoperative Short-Term Calorie Restriction for Prevention of Acute Kidney Injury After Cardiac Surgery: A Randomized, Controlled, Open-Label, Pilot Trial. *J Am Heart Assoc,* 7.

Gunerhan, Y., Koksal, N., Sahin, U. Y., Uzun, M. A. & Ekşioglu-Demiralp, E. 2009. Effect of preoperative immunonutrition and other nutrition models on cellular immune parameters. *World J Gastroenterol,* 15**,** 467-72.

Hollis, G., Franz, R., Bauer, J. & Bell, J. 2020. Implementation of a very low calorie diet program into the pre-operative model of care for obese general elective surgery patients: Outcomes of a feasibility randomised control trial. *Nutrition & dietetics: the journal of the Dietitians Association of Australia.,* 17.

Hossain, L. E., Gonzalez, F. G. & Manuel, R. R. 2016. Effect of "immunonutrition" compared to standard nutrition in gastrointestinal malignancies. *Surgical Infections,* 17 (Supplement 1)**,** S27.

Kaya, S. O., Akcam, T. I., Ceylan, K. C., Samancılar, O., Ozturk, O. & Usluer, O. 2016. Is preoperative protein-rich nutrition effective on postoperative outcome in non-small cell lung cancer surgery? A prospective randomized study. *J Cardiothorac Surg,* 11**,** 14.

Kikuchi, Y., Hiroshima, Y., Matsuo, K., Kawaguchi, D., Murakami, T., Yabushita, Y., . . . Tanaka, K. 2016. A Randomized Clinical Trial of Preoperative Administration of Branched-Chain Amino Acids to Prevent Postoperative Ascites in Patients with Liver Resection for Hepatocellular Carcinoma. *Ann Surg Oncol,* 23**,** 3727-3735.

Krasowska, K., Skrobot, W., Liedtke, E., Sawicki, P., Flis, D. J., Dzik, K. P., . . . Kaczor, J. J. 2019. The preoperative supplementation with Vitamin D attenuated pain intensity and reduced the level of pro-inflammatory markers in patients after posterior lumbar interbody fusion. *Frontiers in Pharmacology,* 10 (MAY) (no pagination).

Krebs, B. 2016. Prebiotic and Synbiotic Treatment before Colorectal Surgery--Randomised Double Blind Trial. *Coll Antropol,* 40**,** 35-40.

Macfie, J., Woodcock, N. P., Palmer, M. D., Walker, A., Townsend, S. & Mitchell, C. J. 2000. Oral dietary supplements in pre- and postoperative surgical patients: a prospective and randomized clinical trial. *Nutrition,* 16**,** 723-8.

Makhija, N., Sendasgupta, C., Kiran, U., Lakshmy, R., Hote, M. P., Choudhary, S. K., . . . Abraham, R. 2008. The role of oral coenzyme Q10 in patients undergoing coronary artery bypass graft surgery. *J Cardiothorac Vasc Anesth,* 22**,** 832-9.

Manzanares Campillo, M. D. C., Martín Fernández, J., Amo Salas, M. & Casanova Rituerto, D. 2017. [A randomized controlled trial of preoperative oral immunonutrition in patients undergoing surgery for colorectal cancer: hospital stay and health care costs]. *Cir Cir,* 85**,** 393-400.

Martinez, J. L., Bosco-Garate, I., Souza-Gallardo, L. M., Mendez, J. D., Juarez-Oropeza, M. A., Roman-Ramos, R. & Ferat-Osorio, E. 2020. Effect of Preoperative Administration of Oral Arginine and Glutamine in Patients with Enterocutaneous Fistula Submitted to Definitive Surgery: a Prospective Randomized Trial. *Journal of Gastrointestinal Surgery,* 24**,** 426-434.

Matzi, V., Lindenmann, J., Muench, A., Greilberger, J., Juan, H., Wintersteiger, R., . . . Smolle-Juettner, F. M. 2007. The impact of preoperative micronutrient supplementation in lung surgery. A prospective randomized trial of oral supplementation of combined alpha-ketoglutaric acid and 5-hydroxymethylfurfural. *Eur J Cardiothorac Surg,* 32**,** 776-82.

Metcalf, R. G., James, M. J., Gibson, R. A., Edwards, J. R., Stubberfield, J., Stuklis, R., . . . Cleland, L. G. 2007. Effects of fish-oil supplementation on myocardial fatty acids in humans. *Am J Clin Nutr,* 85**,** 1222-8.

Mikagi, K., Kawahara, R., Kinoshita, H. & Aoyagi, S. 2011. Effect of preoperative immunonutrition in patients undergoing hepatectomy; a randomized controlled trial. *Kurume Med J,* 58**,** 1-8.

Nagata, S., Shirabe, K., Sugimachi, K., Ikegami, T., Yoshizumi, T., Uchiyama, H., . . . Maehara, Y. 2013. Pilot study of preoperative immunonutrition with antioxidants in living donor liver transplantation donors. *Fukuoka Igaku Zasshi,* 104**,** 530-8.

Nakamura, K., Kariyazono, H., Komokata, T., Hamada, N., Sakata, R. & Yamada, K. 2005. Influence of preoperative administration of omega-3 fatty acid-enriched supplement on inflammatory and immune responses in patients undergoing major surgery for cancer. *Nutrition,* 21**,** 639-49.

Patel, V. C., Aldridge, R. D., Leeds, A., Dornhorst, A. & Frost, G. S. 2004. Retrospective analysis of the impact of a low glycaemic index diet on hospital stay following coronary artery bypass grafting: a hypothesis. *J Hum Nutr Diet,* 17**,** 241-7.

Polakowski, C. B., Kato, M., Preti, V. B., Schieferdecker, M. E. M. & Campos, A. C. L. 2019. Impact of the preoperative use of synbiotics in colorectal cancer patients: A prospective, randomized, double-blind, placebo-controlled study. *Nutrition,* 58**,** 40-46.

Pronio, A., Di Filippo, A., Aguzzi, D., Laviano, A., Narilli, P., Piroli, S., . . . Montesani, C. 2008. [Treatment of mild malnutrition and reduction of morbidity in major abdominal surgery: randomized trial on 153 patients]. *Clin Ter,* 159**,** 13-8.

Russell, K., Zhang, H. G., Gillanders, L. K., Bartlett, A., Fisk, H. L., Calder, P. C., . . . Plank, L. D. 2019. Preoperative immunonutrition in patients undergoing liver resection: A prospective randomized trial. *World Journal of Hepatology,* 11**,** 305-317.

Schouten, R., Van Der Kaaden, I., Van 'T Hof, G. & Feskens, P. G. 2016. Comparison of Preoperative Diets Before Bariatric Surgery: a Randomized, Single-Blinded, Non-inferiority Trial. *Obes Surg,* 26**,** 1743-9.

Smedley, F., Bowling, T., James, M., Stokes, E., Goodger, C., O'connor, O., . . . Silk, D. 2004. Randomized clinical trial of the effects of preoperative and postoperative oral nutritional supplements on clinical course and cost of care. *Br J Surg,* 91**,** 983-90.

Sufit, A., Weitzel, L. B., Hamiel, C., Queensland, K., Dauber, I., Rooyackers, O. & Wischmeyer, P. E. 2012. Pharmacologically dosed oral glutamine reduces myocardial injury in patients

undergoing cardiac surgery: a randomized pilot feasibility trial. *JPEN J Parenter Enteral Nutr,* 36**,** 556-61.

Tumas, J., Jasiunas, E., Strupas, K. & Sileikis, A. 2020. Effects of Immunonutrition on Comprehensive Complication Index in Patients Undergoing Pancreatoduodenectomy. *Medicina-Lithuania,* 56.

Van Ginhoven, T. M., De Bruin, R. W., Timmermans, M., Mitchell, J. R., Hoeijmakers, J. H. & Ijzermans, J. N. 2011. Pre-operative dietary restriction is feasible in live-kidney donors. *Clin Transplant,* 25**,** 486-94.

Van Nieuwenhove, Y., Dambrauskas, Z., Campillo-Soto, A., Van Dielen, F., Wiezer, R., Janssen, I., . . . Thorell, A. 2011. Preoperative very low-calorie diet and operative outcome after laparoscopic gastric bypass: a randomized multicenter study. *Arch Surg,* 146**,** 1300-5.

Yoshitomi, M., Kawahara, R., Akasu, G., Ishikawa, H. & Kinoshita, H. 2009. Preoperative immunonutrition for patients undergoing pancreatoduodenectomy (randomized controlled trial). *Pancreas.,* 38**,** 1066.

Zhang, J. W., Du, P., Gao, J., Yang, B. R., Fang, W. J. & Ying, C. M. 2012. Preoperative probiotics decrease postoperative infectious complications of colorectal cancer. *Am J Med Sci,* 343**,** 199-205.

Zhao, Q., Li, Y., Yu, B., Yang, P., Fan, L., Tan, B. & Tian, Y. 2018. Effects of Preoperative Enteral Nutrition on Postoperative Recent Nutritional Status in Patients with Siewert II and III Adenocarcinoma of Esophagogastric Junction after Neoadjuvant Chemoradiotherapy. *Nutrition and Cancer,* 70**,** 895-903.

EXERCISE INTERVENTIONS

Table 8. Characteristics of studies

ID	1st Author, year and country	Total n randomized Intervention (I):Control (C) (Number analysed) if reported	Patient population, baseline clinical characteristics (mean (SD) or *n (%)* unless otherwise stated	Demographics (mean (SD) or n unless otherwise stated)	Intervention	Comparator	Mode of delivery; place of delivery; training level of individuals who delivered the intervention; the number of contacts	Intervention fidelity; Compliance or adherence to intervention
**EXERCISE INTERVENTIONS**								
1	Argunova et al., 2018 Russia (Translation)	38 male ppts with stable coronary heart disease undergoing on-pump CABG I=20:C=18	**BMI kg/m2** I=28.1(3.99): C=28.5 (3.14) **Hypertension (n)** I=15:C=17 **History of MI (n)** I=10:C=9 **History of stroke (n)** I=1:C=1	**Age yrs** I=57.9 (7.2) C=60.4 (7.01) **Overall Gender (%M)** 100	High intensity exercise training Supervised treadmill exercises daily for 7 days	No exercise	Clinic based;<br>NR;<br>NR;<br>Daily measures (7 days)	NR;<br>NR
2	Banerjee et al., 2018, Banerjee et al., 2014 UK	60 ppts undergoing radical cystectomy I=30:C=30	**Hypertension %** I=57:C=57 **Ischaemic heart disease %** I=10:C=27 **Type 2 diabetes %** I=10:C13	**Age yrs** I=71.6(6.8):C=72.5(8.4) **Gender (%M)** I=90:C=87 **Current smokers %** I=13:C=13	2x/wk preop supervised vigorous intensity aerobic interval exercise + usual care (3-6 wks)	Usual care	Supervised exercise:<br>NR (assumed hospital);<br>NR;<br>NR	NR;<br>Median number of supervised exercise sessions attended 8 (range 1-10)
3	Barakat et al., 2016 UK	136 ppts undergoing open or endovascular abdominal aortic aneurysm (AAA) repair randomized; I=68(62An) C=68(62An)	Ppts 18+ yrs, with AAA ≥5.5cm in maximum diameter	**Age yrs** I=73.8(6.5):C=72.9(7.9) **Gender (%M)** I=90:C=89	Ppts provided with instructions and a timetable for hospital-based exercise classes, carried out 3x/wk, for 1-hr duration, in the physiotherapy gym for 6 wks	Usual care (no exercise classes)	1-to-1 (described as 'exercise classes' which may mean group sessions);<br>Physiotherapy gym in hospital;<br>Physiotherapists;<br>Supervised intervention thus continuous clinical supervision 3x/wk for 6 wks	NR;<br>32/62 ppts attended 13-18 classes; 19 attended 6-12 classes; 11 did not attend
4	Blackwell et al., 2020 UK	40 ppts with urological cancer	**Weight, kg** I=80.1 (10.4) C=79.6 (14.4)	**Age, yrs** I=71 (2) C=72 (4) **Gender (%M)**	HIIT 4 wk fully-supervised HIIT intervention.Ppts initially trained at 100–115% of maximum wattage achieved at BL CPET	Usual care	Face to face;<br>University exercise lab;<br>Qualified doctor;<br>Approx. 16	adherence (ten or more HIIT sessions) to the exercise training protocol was 84% (16/19).

ID	1st Author, year and country	Total n randomized Intervention (I):Control (C)<br><br>(Number analysed) if reported	Patient population, baseline clinical characteristics (mean (SD) or *n (%)* unless otherwise stated	Demographics (mean (SD) or n unless otherwise stated)	Intervention	Comparator	Mode of delivery; place of delivery; training level of individuals who delivered the intervention; the number of contacts	Intervention fidelity; Compliance or adherence to intervention
**EXERCISE INTERVENTIONS**								
		scheduled for major urological surgery<br><br>I=19 (18An)<br>C=21 (16An)	**Hypertension (medicated)**<br>I=8:C=7<br>**Diabetes**<br>I=0:c=2<br>**Musculoskeletal (osteoarthritis, rheumatoid arthritis or joint replacement)**<br>I=8:C=8<br>**None**<br>I=2:C=4	I=100:C=95	upto 12 HIIT session (3-4 wkly). Indiivdual sessions.			
5	Bridevaux et al., 2012 Switzerland preliminary data only	So far 65 ppts undergoing thoracic surgery with non-small cell lung cancer<br>I=31: C=34	NR	**Overall age, yrs**<br>63.4 [(0.5)	Short-term rehabilitation intensive, respiratory physiotherapist supervised interval training 3x/wk	Usual care	1-to-1 (described as 'exercise classes' which may mean group sessions);<br>Physiotherapy in hosptial;<br>Physiotherapists;<br>Supervised intervention thus continuous clinical supervision 3x/wk	NR:<br>NR
6	Carver et al., 2011<br>Abstract only (re-analysis of data arising from an RCT where ppts performed an 8-wk exercise program)	14 ppts undergoing bariatric surgery (obese ppts)	**Overall**<br>**BMI kg/m^2**<br>48(6)	**Overall**<br>**Age yrs**<br>40(10)<br>**Gender (%M)**<br>I=28.5:C=NR	Ppts performed an 8 wk exercise program consisting of cardiovascular (3/wk) and strength-training (1/wk) exercises.	NR	NR;<br>NR;<br>NR;<br>NR	NR;<br>NR

ID	1st Author, year and country	Total n randomized Intervention (I):Control (C)<br><br>(Number analysed) if reported	Patient population, baseline clinical characteristics (mean (SD) or *n (%)* unless otherwise stated	Demographics (mean (SD) or n unless otherwise stated)	Intervention	Comparator	Mode of delivery; place of delivery; training level of individuals who delivered the intervention; the number of contacts	Intervention fidelity; Compliance or adherence to intervention
**EXERCISE INTERVENTIONS**								
7	Cavill et al., 2016<br>Australia	64 ppts undergoing elective lower-limb arthroplasty<br><br>N=64<br>I=32 (THR=11/ TKR=21)<br>C=32 (THR=12/TK= 20)	Groups were comparable in demographics, joint range, physical function and QoL at time of recruitment	**Age yrs**<br>I=65.3(9.6): C=67.0(9.4)<br>**Gender (%M)**<br>I=50:C=47	Pre surgery: assessed at community rehab center by a physiotherapist prior to attending a 1hr group session of exercise and education.<br>2x/wk 1hr group session (circuit program of 6 stations, individualized dosage) min. 3 wks max. 4 wks. An individually tailored home exercise program was also provided, which included many of the same exercised done in the group. If surgery postponed or no surgery date – ppt instructed to continue with home exercise program until they presented for surgery.	Usual care	Verbal, face to face, group;<br>Community rehabilitation center + home exercise programme;<br>NR: possibly physiotherapist<br>Assessed initially by physiotherapist then ppts attended 2 x wkly, 1 hr sessions at the CRC for no less than 3 wks and max of 4 wks prior to surgery	NR;<br>NR
8	D'Lima et al., 1996<br>USA	30 ppts with arthritis or osteoarthritis undergoing elective primary unilateral TKR randomised to<br>I(EPT)=10<br>I(ECC)=10<br>C=10	Age >55 yrs with primary diagnosis of arthritis/ osteoarthritis	**Age yrs**<br>EPT=68.5(4.6)<br>ECC=71.6 (6.6)<br>C=69.5(6.5)<br>**Gender (%M)**<br>EPT=30:ECC= 80: Cl=50	**GROUP 1**<br>Experimental physical therapy (EPT): 1-on-1 Physical Therapy training program. 45 min./session, 3 sessions/wk for a total of 18 sessions (intervention designed to improve limb strength and knee range of motion, not meeting our definition of prehabilitation, so excluded from MA)<br>**GROUP 2**<br>Experimental cardiovascular conditioning (ECC):– ppts were tested by an exercise physiologist – a tailored CC program was designed for each ppt. 3 exercise sessions/wk, each lasting 45 mins, for a total of 18 sessions (12 sessions emphasised arm and cycle	Usual care: Ppts met physiotherapist. Printed material was provided. No recommendations for preop physical therapy or exercise were made.<br><br>The existing routine PO protocol for total knee replacements was used (quad and hamstring setting, straight leg raises, hamstring and heel cord stretches, knee strengthening, sitting and prone knee range of motion exercises, and routine precautions)	Verbal and written 1 to 1;<br>NR but assume in hospital;<br>Physiotherapists;<br>Supervised sessions, so follow up at each session	NR;<br>NR

ID	1st Author, year and country	Total n randomized Intervention (I):Control (C)<br><br>(Number analysed) if reported	Patient population, baseline clinical characteristics (mean (SD) or *n (%)* unless otherwise stated	Demographics (mean (SD) or n unless otherwise stated)	Intervention	Comparator	Mode of delivery; place of delivery; training level of individuals who delivered the intervention; the number of contacts	Intervention fidelity; Compliance or adherence to intervention
**EXERCISE INTERVENTIONS**								
					ergometry, 6 sessions consisted of aquatic exercises)			
9	Dunne et al., 2016<br>UK	N=38 undergoing elective liver resection for colorectal liver metastases<br>I=20<br>C=18 (17 + one withdrew, before knowing arm allocation)<br>N=35 completed both preop assessments and analysed<br>I=19:C=16	**BMI kg/m^2**<br>I=29.7(4.2):<br>C=29.3(4.2)<br>**Co-mobidity**<br>**Cardiovascular**<br>I=10:C=8<br>**Respiratory**<br>I=3:C=4<br>**Diabetes**<br>I=2:C=2<br>**Renal disease:**<br>I=1:C=0<br>**None:**<br>I=1:C=3	**Age median (IQR)**<br>I=61(56-66):<br>C=62(53-72)<br>**Gender (%M)**<br>I=65:C=76<br>**Smoking status (n)**<br>**Smoker:**<br>I=2:C=3<br>**Ex-Smoker:**<br>I=3:C=3<br>**Non-Smoker:**<br>I=15:C=11	4-wk (12 sessions) high-intensity cycle, interval training programme (personalised).<br>2 recovery exercise sessions were included at the end of the 1st and 4th wks (sessions 3 and 12). The interval sessions included a warm-up and warm-down, and 30min of interval training alternating between exercise of moderate (less than 60% *VO*^{2} at peak exercise) and vigorous (more than 90% *VO*^{2} at peak) intensity.	Usual care	Face-to-face;<br>NR but assume hospital;<br>NR;<br>12 sessions over 4 wks	NR:<br>I: 18/19 ppts analyzed completed 100% of exercise sessions. 1 patient missed 2 sessions.
10	García et al., 2016, Garcia et al., 2016, Garcia et al., 2017<br>Poster and paper<br>Spain	N=40 ppts with lung cancer undergoing video-assisted thoracotomy<br>I=20<br>C=20 (2 did not receive allocated intervention as they were referred to preop physio)<br>Analysed Pre-surgery<br>I=10	**BMI kg/m^2**<br>I=29.4(4.3)<br>C=25.7(2.8)<br>**Colinet Co-morbidity Score**<br>I=9.3(4.9)<br>C=8.7(4.2)<br>**Respiratory disease %**<br>I=70:C=33<br>**Cardiovascular disease %**<br>I=80:C=75<br>**Renal impairment %**<br>I=0:C=0<br>**Diabetes mellitus %**	**Age yrs**<br>I=70.9(6.1)<br>C=69.4(9.4)<br>**Gender (%M)**<br>I=90:C=92<br>**History of smoking (*n*, %)**<br>**None:**<br>I=2(20%):<br>C=2(16.7%)<br>**Former:**<br>I=8(80%):<br>C=7(58.3%)<br>**Current:**<br>I=0:C=3(25%)	Preop exercise-based intervention, 3-5 sessions/wk of interval endurance training (30 mins of interval training; 5 min warm up, 1 min at high intensity, plus 4 mins of 'active rest' and 4 min cool down), resistance training with elastic bands (6 exercises, 3 x 15 reps each with 45 secs between tests)<br>+<br>breathing exercises 2x daily at home using a spirometer.<br>The ppts must complete at least 16 sessions (according to protocol)	Standard care (no prehabilitation)	Face-to-face;<br>Rehabilitation room at University Hospital;<br>Supervised by highly specialized physiotherapist with an expertise in respiratory physiotherapy and pulmonary rehabilitation;<br>3-5 x/wk depending on surgery date	NR;<br>NR

ID	1st Author, year and country	Total n randomized Intervention (I):Control (C)<br><br>(Number analysed) if reported	Patient population, baseline clinical characteristics (mean (SD) or *n (%)* unless otherwise stated	Demographics (mean (SD) or n unless otherwise stated)	Intervention	Comparator	Mode of delivery; place of delivery; training level of individuals who delivered the intervention; the number of contacts	Intervention fidelity; Compliance or adherence to intervention
**EXERCISE INTERVENTIONS**								
		Analysed post-surgery<br>I=10:C=12	I=10:C=25<br>**Alcoholism %**<br>I=0:C=0<br>**Past history cancer %**<br>I=40:C=50<br>Ppts in the prehabilitation group tended to have a higher BMI, lower pulmonary function and lower peak exercise capacity.					
11	Hoogeboom et al., 2010<br>The Netherlands	N=21 ppts with hip osteoarthritis undergoing elective THA<br>I=10:C=11	Frail older adults with hip osteoarthritis	**Age yrs**<br>I=77(3):<br>C=75(5)<br>**Gender (%M)**<br>I=70:C=64	Therapeutic exercise program 3-6 wks. 60 min supervised sessions at least 2x/wk.<br>Supervised session had 4 phases:<br>(1) 5 min warm up walk;<br>(2) Leg press (sets of 10-20);<br>(3) Bicycle ergometer (20-30 mins);<br>(4) Tailor-made training integrating functional physical exercises into ppt's daily living.<br>Encouraged to exercise at home<br>Encouraged to use a pedometer on non-training days, provided feedback about intensity, include family in the exercise, progressed training parameters gradually	Usual care (in hospital) single group education session about early mobilisation, surgery and anaesthesia techniques, restricted movement, benefits of activity and proper use of crutches	Face to face then alone;<br>Outpatient dept of physiotherapy;<br>Physiotherapists (experience and trained);<br>NR	Tested feasibility of the intervention as this was a pilot study. All ppts received intended intervention<br><br>Adherence to treatment: all ppts showed good adherence: 91% participation in all sessions. Ppts valued the therapy and treatment
12	Kaibori et al., 2013<br>Japan	51 ppts with hepatocellular carcinoma who had chronic	**Diabetes Mellitus** (yes/no)<br>I=3/22:C=3/23<br>**Tumor stage (I or II/III or IV)**	**Age yrs**<br>I=68.0(9.1)<br>C=71.3(8.8)<br>**Gender (M%)**<br>I=65:C=76	Exercise at the anaerobic threshold of each ppt - started 1 mth preoperatively,<br>(resumed from 1 wk PO and continued for 6 mths).	Conventional perioperative care (+ diet therapy)<br>The dietitian provided instruction on the	NR<br>Hospital, not clear if home based for all sessions or not;<br>Exercise trainer – no mention of training level;	11 individuals maintained the exercise program (high frequency), while 14 did not.

ID	1st Author, year and country	Total n randomized Intervention (I):Control (C)<br><br>(Number analysed) if reported	Patient population, baseline clinical characteristics (mean (SD) or *n (%)* unless otherwise stated	Demographics (mean (SD) or n unless otherwise stated)	Intervention	Comparator	Mode of delivery; place of delivery; training level of individuals who delivered the intervention; the number of contacts	Intervention fidelity; Compliance or adherence to intervention
**EXERCISE INTERVENTIONS**								
		hepatitis or cirrhosis scheduled for liver resection<br><br>Exercise=25<br>Diet=26	I=19/6:C=21/5		The program consisted of 3x 60-min exercise sessions/wk. Each session included 5 min of stretching exercises, 30 mins of walking at an intensity based on the AT of each ppt, 20 mins of targeted stretching exercises, and 5 mins of cooling down with stretching<br><br>(+ diet therapy)	specific diet to both groups of ppts and checked ppt adherence preoperatively and at 1, 3, and 6 mths PO<br>For ppts with chronic hepatitis or liver cirrhosis, the daily energy intake was set at 25 to 30 kcal/kg body weight, with a daily protein intake of 1.0 to 1.2 g/kg body weight and a daily sodium chloride intake of 5 to 7 g/kg body weight during the 1-mth preoperative.<br>For ppts with diabetes or fatty livers, daily energy intake was set at 20 to 25 kcal/kg body weight, while the daily sodium chloride intake was set at 6 g/kg body weight for ppts with hypertension.	NR	Reported that some ppts did not complete the full exercise program as required - Some ppts failed to continue to exercise in the long term for various reasons (tumor recurrence, financial reasons, exacerbation of other diseases, etc)
13	Licker et al., 2017;<br>Karenovics et al., 2017<br>Switzerland	164 ppts with operable lung cancer undergoing lung surgery<br><br>I=81(77An)<br>C=83 (74An)	**COPD**<br>I=30(41):C=27(35)<br>**Hypertension**<br>I=33(45):C=32(42)<br>**Diabetes mellitus**<br>I=10(13.5):<br>C=11(14.3)<br>**Coronary artery disease**<br>I=10(13.5):<br>C=8(10.4)<br>**Heart failure**<br>I=8(10.8):	**Age yrs**<br>I=64(13)<br>C=64(10)<br>**Gender (%M)**<br>I=55:C=65<br>**Smoking**<br>**Current:**<br>I=28:C=39<br>**Past:**<br>I=43:C=31<br>**Non:**<br>I=4:C=7	HIIT programme<br><br>Exercised on a cycling ergometer in the outpatient clinic 2-3x/wk under supervision of physiotherapists.<br>2x 10 min series of 15 min sprint interval interspersed by 15-sec pauses and a 4-min rest between the two series.<br>Additional exercises, such as leg press, leg extension, back extension, seat row, biceps	Usual care	Verbal;<br>Outpatient clinic;<br>Physiotherapist;<br>Supervised throughout	NR;<br>NR

ID	1st Author, year and country	Total n randomized Intervention (I):Control (C)<br><br>(Number analysed) if reported	Patient population, baseline clinical characteristics (mean (SD) or *n (%)* unless otherwise stated	Demographics (mean (SD) or n unless otherwise stated)	Intervention	Comparator	Mode of delivery; place of delivery; training level of individuals who delivered the intervention; the number of contacts	Intervention fidelity; Compliance or adherence to intervention
**EXERCISE INTERVENTIONS**								
			C=8(10.4)<br>**Cardiac arrhythmias**<br>I=3(4.1):C=5(6.5)<br>**History of stroke**<br>I=6(8.1):C=1(1.3)<br>**Renal dysfunction**<br>I=4(5.4):C=6(7.8)<br>**Peripheral arterial disease**<br>I=16(21.6):C=13(16.9)<br>**Revised cardiac risk index**<br>I=1(1-2):C=1(1-2)<br>**Severity of illness ASA classes 3 & 4**<br>I=22:C=28		curls, or chest and shoulder press, were proposed on an individual basis.<br>+ advice + risk factor management			
14	Lindbäck et al., 2018<br>Fors et al., 2019<br>Sweden<br>“PREPARE”	197ppts undergoing spine surgery with degenerative lumbar spine disorder<br>I=99 (99An):<br>C=98 (98An)	**Diagnosis, n (%)**<br>**Spinal stenosis**<br>I=59/99:C=70/98<br>**Disc herniation**<br>I=23/99:C=17/98<br>**spondylolisthesis**<br>I=8/99:C=7/98<br>**Degenerative disc disease**<br>I=9/99:C=4/98	**Age, yrs**<br>I=58 (13.3)<br>C=61 (11.5)<br>**Gender**<br>I=46:C=48<br>**Cigarette smoker %**<br>I=99:C=98	Physiotherapy intervention<br>2x wk for 9 wks:<br>(1) physiotherapy according to a treatment-based classification<br>(i) specific exercises and mobilization,<br>(ii) motor control exercises, or<br>(iii)traction.<br>(2) tailor-made general supervised exercise program.<br>(3) behavioral approach to reduce fear avoidance and increase activity level.	Usual care (wait list control)	Face-to-face;<br>Supervised;<br>Physiotherapist;<br>2x wk for 9 wks	NR:<br>Adherence:<br>Received allocated intervention 1-18 sessions=85;<br>Did not receive allocated intervention, 0 session=14
15	Maguire et al., 2018<br>NR<br>Abstract only<br>Rex study	32 ppts with rectal cancer undergoing chemo-radiotherapy	**NR**	**NR**	telephone-guided walking intervention<br>(15-17 wks duration)	Usual cre	Telephone-bsed intervention;<br>Assumed home;<br>NR;<br>NR	NR;<br>NR

ID	1st Author, year and country	Total n randomized Intervention (I):Control (C)<br><br>(Number analysed) if reported	Patient population, baseline clinical characteristics (mean (SD) or *n (%)* unless otherwise stated	Demographics (mean (SD) or n unless otherwise stated)	Intervention	Comparator	Mode of delivery; place of delivery; training level of individuals who delivered the intervention; the number of contacts	Intervention fidelity; Compliance or adherence to intervention
**EXERCISE INTERVENTIONS**								
		I=NR:C=NR						
16	Mak et al., 2019<br>NR pilot study<br>Abstract only	22 ppts over 70 yrs of age undergoing colorectal cancer surgery	**NR**	**Overall Median age**<br>73.5 yrs<br>**Overall Gender (M%)**<br>55	Pre-operative exercise protocol involving daily 30 min home-based aerobic exercises before surgery.	NR	NR where the exercise were taught but they were performed at home	NR
17	Marchand et al., 2019<br>Canada	40 ppts awaiting decompression surgery for lumbar spinal stenosis<br>I=20:C=20	**Weight (kg)**<br>I=76.7 (16.4)<br>C=83.1 (14.8)	**Age, yrs**<br>I=66.7(11.6)<br>C=71.5 (7.3)<br>**Gender (%M)**<br>I=55:C=60	Exercise-based intervention 3x/wk for 6 wks prior to surgery. Sessions began with a 5-min warm-up which consisted of cycling (stationary) or walking (treadmill) based on ppts' preference, followed by 5 muscular exercises with concentric or isometric phases that aim to improve muscle and structures involved in walking capacities.<br>Each exercise intensity level was tailored to the participant's capacity and progressively modified to obtain increasing levels of difficulty in order to provide a safe, individualized and yet motivating training experience for each ppt.	Usual care<br>Including written information on how to keep a good back posture when getting in or out of bed and when sitting down.	Face-to-face;<br>Université du Québec à Trois-Rivières;<br>certified kinesiologist;<br>18	The process of defining the components of the training sessions was undertaken by three clinical experts familiar with the LSS population but working in different fields (kinesiology, chiropractic and neurosurgery).<br><br>A total of 8 ppts completed all 18 training sessions as planned (40% compliance) whereas 9 completed more than 50% of sessions (range: 11–17) and 3 less than 50% (range: 2–7).
18	McHugh, 2011<br>PhD Thesis<br>Ireland	21 ppts with advanced, predominantly unilateral knee osteoarthritis<br>I=10 (10An)<br>C=11 (10An)	**Weight (kg)**<br>I=85.9 (16.2)<br>C=84.4 (17.0)<br>**BMI (kg/m^2)**<br>I=30.7 (5.4)<br>C=32.1 (6.8)<br>**Kellgren Lawrence grade 3:4**<br>I=3:7: C=4:6	**Age, yrs**<br>I=63 (3.8)<br>C=61 (5.0)<br>**Gender (%M)**<br>I=17:C=17	6 wk home based pre-operative home workout programme with emphasis on building quadricep strength.<br>Strength exercises performed 3 days/wk and flexibility exercises to be performed 5 days/wk.	Usual care. Ppts were not discouraged from doing exercise, in order to represent the normal standard of care.	Face-to-face;<br>Home;<br>Physiotherpaist;<br>BL and prior to surgery	NR;<br>NR

Perry R, *et al. BMJ Open* 2022; 11:e050806. doi: 10.1136/bmjopen-2021-050806

ID	1st Author, year and country	Total n randomized Intervention (I):Control (C)<br><br>(Number analysed) if reported	Patient population, baseline clinical characteristics (mean (SD) or *n (%)* unless otherwise stated	Demographics (mean (SD) or n unless otherwise stated)	Intervention	Comparator	Mode of delivery; place of delivery; training level of individuals who delivered the intervention; the number of contacts	Intervention fidelity; Compliance or adherence to intervention
**EXERCISE INTERVENTIONS**								
19	Nolan et al., 2019<br>Conference abstract<br>UK	NR ppts undergoing cardiac or thoracic surgery ongoing study	**NR**	**NR**	instructions in the use of a patient diary, pedometer, incentive spirometer and a home-based exercise programme including walking and simple breathing exercises.	Usual care	Written instructions;<br>Home based;<br>NR;<br>NR	NR;<br>NR
20	Northgraves et al., 2020<br>UK	22 ppts with colorectal cancer or benign colorectal disease undergoing elective colorectal surgery<br>I=11:C=11	**BMI (kg/m^2)**<br>I=30.3 (4.3)<br>C=27.8 (5.7)<br>**Diagnosis:**<br>**Colon cancer**<br>I=3:C=4<br>**Rectal cancer**<br>I=6:C=6<br>**Diverticular disease**<br>I=1:C=1<br>**Neoadjuvant chemo-radiotherapy**<br>I=4:C=3	**Age, yrs**<br>I=64.1 (10.5)<br>C=63.5 (12.5)<br>**Gender (%M)**<br>I=40:C=64	2 wks exercise-resistance training and aerobic exercise. The prehabilitation programme consisted of 3 individualised 60-min exercise sessions/wk. The programme was tailored to the individual.<br><br>Progressions were applied every 2 to 3 sessions,dependent on the ppt's ability.	Standard care<br>No specific instructions or restrictions were placed on the physical activity	Fce-to-face;<br>University Sport Science Laboratory;<br>Trained instructor;<br>6	NR;<br>NR
21	Oosting et al., 2012<br>The Netherlands<br>Pilot study	30 ppts frail older adults undergoing elective THA with a min. waiting period of 3 wks<br>I=15 (14An)<br>C=15 (12An)	**Use of walking assistance %**<br>I=60:C=53<br>**BMI kg/m^2**<br>I=28.6(5.6)<br>C=27.8(4.2)	**Age yrs**<br>I=76.9(6.3):<br>C=75.0(6.3)<br>**Gender (%M)**<br>I=7:C=33	PT 3-6 wks. 30 min supervised sessions 2x/wk. Plus additional training 4x/wk alone or with help of friends/relatives. Tailored to ppt and home environment, intensity and reps gradually increase over time. Moderate intensity (55% to 75% of their maximal heart rate or to perceived exertion between 11 and 13 on the Borg scale)	Usual care (in hospital) single group session supervised by PT 3 wks pre-surgery: received info. on walking with crutches and exercise to be performed in PO phase	Face to face – supervised then alone;<br>Home;<br>Physical therapists;<br>2x wk (3-6 wks)	Tested feasibility of the intervention as this was a pilot study;<br>NR
22	Rooks et al., 2006<br>USA	108 ppts scheduled to undergo unilateral, primary THA or TKA for	**No. of comorbidities, median (range)**<br>**THA** I=1.0(0-7):C=1.0(0-6)	**Age yrs**<br>**THA**<br>I=65(11):<br>C=59(7)<br>**TKA**<br>I=65(8):	The exercise group performed water & land-based exercise 3x/wk over 6 wks. Each session lasted 30-60mins.	Control: used info. from the preop education booklet provided to all patients undergoing total joint arthroplasty	Assumed 1-to-1;<br>Community fitness facility;<br>Physical therapist (SAB);<br>3x/wk for 6 wks immediately prior to surgery;<br>18	2 main reasons for not participating: problems with transportation to the intervention site and the time commitment needed to participate.

ID	1st Author, year and country	Total n randomized Intervention (I):Control (C) (Number analysed) if reported	Patient population, baseline clinical characteristics (mean (SD) or *n (%)* unless otherwise stated	Demographics (mean (SD) or n unless otherwise stated)	Intervention	Comparator	Mode of delivery; place of delivery; training level of individuals who delivered the intervention; the number of contacts	Intervention fidelity; Compliance or adherence to intervention
**EXERCISE INTERVENTIONS**								
		advanced osteoarthritis<br><br>Assigned<br>THA I=32<br>THA C=31<br>TKA I=22<br>TKA C=23<br><br>Completed study<br>THA I=25<br>THA C=24<br>TKA I=14<br>TKA C=15	**TKA** I=2.0(0-8):C=1.0(0-6)<br>**BMI kg/m2**<br>**THA** I=28.4(5.3): C=30.3(9.1)<br>**TKA** I=35.7(9.2): C=33.9(6.5)<br>BL data of completers and dropouts within each of the 4 groups were similar. The only diff in BL measures was a higher WOMAC function score in the THA exercise dropout group.	C=69 (8)<br>**Gender (%M)**<br>**THA**<br>I=37:C=48<br>**TKA**<br>I=50:C=43	During the first 3 wks, ppts performed 1–2 sets of 8–12 reps of single-joint movements while standing in chest-deep, 93°F water. During wks 4–6, exercise sessions involved a total body fitness program of cardiovascular, strength, and flexibility training (using stationary bike and elliptical devic). Intervention was tailored to each person's fitness level and comfort with performing the movements.			Adherence to the exercise intervention was high; ppts attended an average of 16 (89%) of 18 class sessions.
23	Santa Mina et al., 2018 Canada	N=86 ppts undergoing radical prostatectomy<br>I=44:C=42<br><br>Preop<br>I=38:C=35<br><br>4 wk<br>I=37:C=34	**BMI kg/m²**<br>I=27.1(4.2)<br>C=27.1(4.4)<br>**WC mean (sd)**<br>I=98.8(10.4)<br>C=100.1(11.7)<br>I and C groups were similar in disease, treatment and outcome variables.	**Age yrs**<br>I=61.2(8.0):<br>C=62.2(6.9)	PREHAB ppts engaged in individualized, total-body exercise, plus daily pelvic floor muscle exercises. Individualized exercise prescriptions consisted of 60 min of unsupervised, homebased, moderate-intensity exercise 3-4 days/wk.<br>PREHAB ppts received an exercise manual and online videos, resistance bands, a stability ball, and a yoga mat to keep after the intervention phase. Ppts were given a heart rate monitor to comply with heart-rate based training zones.	In addition to the pelvic floor exercise regimen and lifestyle manual, usual care ppts received a ppt study manual detailing a pelvic floor training regimen with an accompanying completion log.	Exercise manual, online videos;<br>Home;<br>NR /"study team")<br>Weekly	The study team communicated with PREHAB and CON ppts wkly to facilitate and monitor program compliance and progression to their respective activities.<br><br>Adherence was measured through a log-book completed by the co-ordinator during wkly communication. Adherence to the homebased exercise program was defined as achieving the minimum of the prescribed exercise range for moderate intensity aerobic and resistance training.

ID	1st Author, year and country	Total n randomized Intervention (I):Control (C)<br><br>(Number analysed) if reported	Patient population, baseline clinical characteristics (mean (SD) or *n (%)* unless otherwise stated	Demographics (mean (SD) or n unless otherwise stated)	Intervention	Comparator	Mode of delivery; place of delivery; training level of individuals who delivered the intervention; the number of contacts	Intervention fidelity; Compliance or adherence to intervention
**EXERCISE INTERVENTIONS**								
					I group also received the same pelvic floor exercises as C group.			I: 27/38 (69.2%) met minimum requirements of their total exercise prescription.<br>36.8% and 38.9% of I and C achieved pelvic floor training prescriptions; no diff between groups.
24	Soares et al., 2013<br>Brazil	37 ppts undergoing abdominal surgery<br>I=18:C=19	**BMI, kg/m^2 (median/IQR)**<br>I=23.6 (120–26)<br>C=24.2 (21–28)<br>**ASA class (median)**<br>I=2.0 (1.8–2.0)<br>C=2.0 (2.0–2.0)<br>**Cancer diagnosis (y/n)**<br>I=12/4:C=13/3	**Age (yrs) median (IQR)**<br>I=58.5 (51–63.5):<br>C=55.0 (49–64)<br>**Gender (%M)**<br>I=50:C=78<br>**History of smoking (y/n)**<br>I=9/7:C=11/5	Physical therapy during the 2 to 3 wks preceding their surgical procedure, consisted of 2 supervised 50-minute physical therapy sessions per wk, including stretching, trunk rotation, deep breathing, respiratory muscle training, active upper and lower extremity exercise, walking and relaxation.<br><br>Respiratory muscle training (IMT 15 mins) and walking (≥10mins) both 4 x per week at home.<br>PO physical therapy consisted of a standardized protocol was applied to both groups until 7th PO day.	Usual care<br><br>PO physical therapy consisted of a standardized protocol was applied to both groups until 7th PO day.	Face to face;<br>Home (unclear where supervised activity took place);<br>Physical therapists;<br>2x/wk for 2-3 weeks.	NR;<br>All ppts reported full adherence with home-based activity.
25	Tenconi et al., 2017<br>Abstract only (preliminary data)	50/140 ppts with early stage lung cancer enrolled so far<br>I=25:C=25	The 2 groups are similar for demographics and functional parameters at BL	NR	pulmonary rehabilitation	Usual care	NR;<br>NR;<br>NR;<br>NR	NR;<br>NR
26	Tew et al., 2017,<br>UK<br>(feasibility study)	53 undergoing elective abdominal aortic aneurysm repair.	**BMI kg/m^2**<br>I=26.5(4.1):C=26.8(3.4)<br>**AAA diameter, mean (sd) cm**<br>I=6.0(0.4):C=5.8(0.4)	**Age yrs**<br>I=74.6(5.5):<br>C=74.9(6.4)<br>**Gender (%M)**<br>I=93:C=96	A preop exercise programme 3x/wk (on a cycle ergometer), for 4 consecutive wks (wks 1–4; main phase) immediately preceding their intended operation date (in week 5). [Ppts whose operation was delayed	Usual care (evidence based medical optimization)	Face-to-face, 1-to-1;<br>Hospital;<br>Research nurse & physiotherapist;<br>12<br><br>(NB - 15 ppts had a delayed operation, due to non-exercise related reasons,	Ppts defined as adherent completed at least 75% of the main-phase sessions (at least 9 of 12 sessions), plus all wkly maintenance sessions if surgery was delayed.

ID	1st Author, year and country	Total n randomized Intervention (I):Control (C)<br><br>(Number analysed) if reported	Patient population, baseline clinical characteristics (mean (SD) or *n (%)* unless otherwise stated	Demographics (mean (SD) or n unless otherwise stated)	Intervention	Comparator	Mode of delivery; place of delivery; training level of individuals who delivered the intervention; the number of contacts	Intervention fidelity; Compliance or adherence to intervention
**EXERCISE INTERVENTIONS**								
		I=27 (24 received at least one exercise session)<br>C=26	**Current or recent (within 6 mths) smoker**<br>I=8:C=2<br>**Co-morbidities:**<br>**Coronary artery disease**<br>I=11:C=14<br>**Cerebrovascular disease**<br>I=7:C=7<br>**Peripheral arterial disease**<br>I=0:C=2<br>**Diabetes mellitus**<br>I=4:C=2<br>**COPD**<br>I=6:C=7		beyond wk 5 (e.g., owing to lack of availability of a hospital bed) also received a maintenance phase of training (1 exercise session/wk)]<br><br>First 3 sessions: 10 min warm-up, 8x2 high intensity cycling interspersed with 2 min unloaded cycling, 5 min cool down. In all subsequent sessions, ppts had the choice of 8x 2-min or 4x 4-min 'work' intervals for the main body of the workout.		and had at least one maintenance session).	The trial protocol specified a success criterion of a lower limit of the 90% confidence interval of 67% for the proportion of the exercise group meeting the prespecified adherence rate. 17/27 exercise ppts (63%) were adherent, so this criterion was not met.
27	Wang et al., 2002<br>Australia	28 ppts undergoing THA<br>I=15:C=13	Ppts with end stage hip arthritis<br>Osteoarthritis=25<br>Inflammatory arthritis=1<br>Osteonecrosis of the hip=2	**Age yrs**<br>I=68.3(8.2):<br>C=65.7(8.4)<br>**Gender (%M)**<br>I=40:C=31	8-wk intervention:<br>2 x 1 hr long supervised clinic-based sessions/wk, including hydrotherapy, stationary bike riding and resistive training machines. Ppts determined the intensity of the exercises, but resistance initially set so that subject could move through pain-free range for 10 reps, once this achieved the weight was increased by 5kg increments.<br>2 home-based sessions with further muscle strengthening using ankle weights, dumbbells and a series of flexibility exercises.	No supervised or structured exercises beyond routine advice provided by hospital physiotherapist	Verbal;<br>Clinic and home-based;<br>NR;<br>16 face-to-face appointments	NR;<br>15 ppts attended 97.3% of scheduled pre-surgery appointments.

KEY: appt=appointment; ASA=American Society of Anaesthesiologists; AAA= abdominal aortic aneurysm; BMI=body mass index; C=control; CC=cardiovascular conditioning; CABG=coronary artery bypass graft;; CD=compact disc; CI=confidence interval; COPD= Chronic obstructive pulmonary

disease; dept=department; diff=difference; ECC=experimental cardiovascular conditioning; EPT= Experimental physical therapy; HIIT=high intensity interval training; hr(s)=hour(s); I=intervention; IMT=inspiratory muscle training; info.=information; IQR=inter-quartile range; LoS=length of stay; M=male; MI=myocardial infarction; min(s)=minute(s); mth(s)=month(s); 6MWT=6 minute walk test; NR=not reported; No.=Number; PO=post-operative; ppts=participants; preop=preoperative/preoperatively; PT=physical therapist; PTED=Preoperative Physical Therapy Education; QoL=quality of life; rehab=rehabilitation; reps=repetitions; RCT=randomised controlled trial; ROM=range of motion; sd=standard deviation; secs=seconds; THA/THR=total hip arthroplasty/total hip replacement; THEP=total hip endoprosthesis; TJA=total joint arthroplasty; TKA=total knee arthroplasty; VO2= oxygen uptake; WC=waist circumference; wk(s)=week(s); WOMAC=Western Ontario and McMaster Universities Osteoarthritis Index; yr(s)=year(s)

EXERCISE INTERVENTIONS

Table 9. Results

	Study	Total number of withdrawals	Clinical outcomes (mean (SD) or n unless otherwise stated)	Intervention-specific outcomes [(n or mean (SD) unless otherwise reported)] and economic evaluations
1	Argunova et al., 2018	None	**Time in ICU hr I=20:C=18**<br>I=25.0 (8.2):C=24.7 (13.3), P>0.05<br>**LoS (days) I=20:C=18**<br>I=11.1 (1.91):C=11.5 (2.03), P>0.05<br>**PO complications** (5-7 days)<br>Reported lower incidence of PO complications in I group (P=0.002), but no other data<br>**MI**<br>I=0/20:C=1/18, P>0.05<br>**Rhythm disturbances**<br>I=2/20:c=3/18, P>0.05<br>**Heart failure**<br>I=1/20:C=4/18, P>0.05<br>**Hydrothorax(a type of pleural effusion)**<br>I=0.20:C=2/18, P>0.05<br>**Hydropericardium**<br>I=0/20:C=0/18, P>0.05<br>**Mental health (SF-36 standard version)**<br>Better mental health in I group<br>I=48.9 (7.60): C=39.1 (6.80), P=0.03	**Outcomes reported: radio-pharmacological marker capture (5-7 days), V0^2 peak, Anaerobic Threshold, exercise tolerance, heart rate:**<br>radio-pharmacological marker capture higher levels in some basal (P=0.04), middle (P=0.03) and atypical (P=0.02) portions of myocardium, by SPECT in I vs C group.<br>V0^2 peak, Anaerobic Threshold, exercise tolerance, heart rate all NS
2	Banerjee et al.,2014; 2018	I=3:C=2	**LoS [median (range)] I=27:C=28**<br>**I=7 (4-78):C=7 (5-107), P=0.865**<br>*LoS days (mean/sd Quantile Estimation (QE) conversion method)*<br>*I=17.80 (37.45):C=22.31 (61.22)*<br>**LoS in high dependency unit [median(range)]**<br>**I=1 (1-10):C=1 (1-7), P = 0.938**<br>**High dependency unit Inotropic support**<br>I=2/27:C=7/28, P=0.078<br>**Surgical complications**<br>**Clavien-Dindo grade ≥1**<br>I=4/27:C=15/28, P=0.075,<br>**Clavien-Dindo grade ≥3**<br>I=1/27:C=4/28, P= 0.172<br>**PO ileus**<br>I=6/27:C=7, P=0.808<br>**Pneumonia**<br>I=3:C=2/28, P=0.609	**Outcomes measured: Cardio-pulmonary exercise test (CPET) parameters prior to surgery:**<br>Improvements in peak values of oxygen pulse (P = 0.001), ventilatory volume (P=0.002) and power output (P < 0.001) but not peak VO2 (P = 0.057) or Anaerobic threshold (P = 0.637) were observed at the FU CPET in the I group vs controls<br><br>**Adverse events**<br>None
3	Barakat et al., 2016	I=6:C=6	**Mortality 30 day**<br>I=2/62:C=2/62, P=1.00	**Outcomes reported: CPET parameters (VO^2 peak, anaerobic threshold, treadmill exercise time)**

	Study	Total number of withdrawals	Clinical outcomes (mean (SD) or n unless otherwise stated)	Intervention-specific outcomes [(n or mean (SD) unless otherwise reported)] and economic evaluations
			**LoS [days, median (IQR)] (I=62:C=62)**<br>I=7 (5.0-9.0):C=8.0 (6.0-12.0), P=0.025<br>*LoS days (mean/*sd Quantile Estimation (QE) conversion *method)*<br>I=7.00 (2.97):C=9.57 (5.50)<br>**ICU stay [days, median (IQR)] (I=62:C=62)**<br>I=1.0 (1.0-2.0):C=1.0 (1.0–1.0), P=0.845<br>**Total complications**<br>I=14/62:C=26/62,<br>**Cardiac complications**<br>I=5/62:C=14/62, P=0.025<br>**Pulmonary complications**<br>I=7/62:C=13/62, P=0.143<br>**Renal complications**<br>I=4/62:C=13/62, P=0.019<br>**Re-operation**<br>I=2/62:C=3/62, P=1.00<br>**Bleeding or transfusion > 4 units**<br>I=4/62:C=7/62, P=0.343<br>**Systemic inflammatory response syndrome (SIRS)**<br>I=50/62:C=51/62, P=0.817	All outcomes improved in the I vs C group (P<0.05).
4	Blackwell et al., 2020	I=1 (1 due to cardiac diagnosis)<br>C=2 did not have BL data<br>3(operation brought forward; 1 unable to contact; 1 DNA)	NR	**Outcomes reported: anaerobic threshold; Blood pressure, DXA**<br>Significant improvements in anaerobic threshold following HIIT.<br>Blood pressure (BP) significantly reduced in HIIT.<br>No significant changes in any DXA derived parameter of body composition total body fat percentage or total lean mass following HIIT<br><br>**Adverse events**<br>There were no adverse safety events reported throughout the study. Mild leg pain at the end of exercise and discomfort from the cycle ergometer seat were reported by 2 ppts, both of which were self-limiting and required no intervention.
5	Bridevaux et al., 2012	NR	NR	**Outcomes reported: FEV, DLCO Vo2 6MWT (preliminary results only)**<br>As reassessed immediately before surgery VO2 peak, P=0.04), watt, P=0.04), 6MWT P<0.01) improved in I but not in C group.
6	Carver et al, 2011		NR	**Outcomes reported: V0² peak; exercise capacity.**<br>No comparisons between I and C. Results reported only in ppts who completed prehabilitation. 5 (36%) improved their V0² peak, 7 (50%) stayed the same, and 2 (14%) declined before surgery. Among ppts who improved fitness during prehabilitation, comparisons in PO exercise capacity to baseline showed that 67% improved and 33% maintained. In ppts with no change in fitness during prehabilitation; 28% maintained and 72% declined.<br>Those who improved their exercise capacity during prehabilitation were better able to attenuate reductions in V0² peak after surgery (P<0.001).

	Study	Total number of withdrawals	Clinical outcomes (mean (SD) or n unless otherwise stated)	Intervention-specific outcomes [(n or mean (SD) unless otherwise reported)] and economic evaluations
7	Cavill et al, 2016	I=32:C=32<br>I=31 attended surgery;<br>29 8-wk outcome FU<br>C=31 attended surgery;<br>30 8-wk outcome FU	**LOS (days) N=29:C=30**<br>I=6.9 (2.4):C=6.9 (2.7), P=0.96<br>**Quality of Life**<br>**EQ-5D utility N=29:C=30**<br>I=0.64 (0.26):C=0.68 (0.17), P=0.50<br>**EQ-5D VAS N=29:C=30**<br>I=70.3 (22):C=70.5 (15.7), P=0.93)	**Outcomes reported: Knee flexion, knee extension, Patient specific functional scale, Timed up and go (TUG), Log (TUG time)**<br>Knee flexion demonstrated an improvement in I vs C (P=0.001)<br>Knee extension NS<br>Patient specific functional scale (P=0.32)<br>Timed up and go (TUG) (P=0.72)<br>Log (TUG time) (P=0.71)<br>No. requiring inpatient rehabilitation I=5:C=4
8	D'Lima et al, 1996	NR	**LoS (days) EPT=10:ECC=10:C=10**<br>EPT=6.29(1):ECC=6.1 (1.1):C=6.08 (1)<br>(EPT excluded from MA)<br>**PO complications**<br>**Wound infection**<br>EPT=0/10:ECC=0/10:C=1/10<br>**Intestinal pseudo-obstruction**<br>EPT=0/10:ECC=0/10:C=1/10<br>**Atrial fibrillation**<br>EPT=1/10:ECC=0/10:C=0/10<br>**Paroxysmal tachycardia**<br>EPT=1/10:ECC=0/10:C=0/10<br>**Sleep apnea**<br>EPT=0/10:ECC=1/10:C=0/10<br>**Restricted ROM**<br>EPT=0/10:ECC=1/10:C=0/10	**Outcomes reported: Hospital knee rating score (BL, post intervention and PO**<br>There was a decrease in function (assessed by knee rating score) in groups 1 and 2 vs C at all time points (data shown on graph).
9	Dunne et al., 2016	I= 1 did not complete exercise programme (concurrent oral cancer) but was included in analysis; 1 excluded from analysis (did not complete 2 CPETs):<br><br>C= 1 withdrew before knowing allocation; 1 withdrew as wanted exercise (Included in analysis); and 1 declined CPET due to arthritis; In total 2 excluded from analysis – did not complete 2 CPETs).<br><br>*Note: Fig 1 and text describing exclusions on bottom of 508-509 not congruent.*	**Duration of stay in critical care (days) median (IQR) (I=19:C=15)**<br>I=1(1–2):C=1·5(1–2)<br>**LoS days median (IQR) (I=19:C=15)**<br>I=5 (4-6):C=5 (4.5-7)<br>*LoS days (mean/sd Quantile Estimation (QE) conversion method)*<br>*I= 5.00 (1.48):C=5.74 (2.09)*<br>**Readmission**<br>I=4/19:C=0/15<br>**Total PO complications**<br>All grades: I=8/19:C=7/15<br>Grades 3 and 4 I=3/19:C=1/15<br>**QoL: SF-36 scores mean comparison scores I vs. C (95% CI.)**<br>**Overall physical health**<br>8 (−1, 16) P=0·102<br>**Overall mental health**<br>11 (1, 22) P=0·037<br>**Overall QoL**<br>11 (1, 21) P=0·028	**Outcomes reported: VO2 at anaerobic threshold, VO2 at peak, oxygen pulse at anaerobic threshold, oxygen pulse at peak, peak work rate, heart rate reserve**<br>Differences favouring I vs C group found for VO2 at anaerobic threshold; P=0·02, VO2 at peak = P=0.047, oxygen pulse at anaerobic threshold; P=0.05, Peak work rate P=0·005<br><br>**Adverse Events**<br>There were no reported adverse outcomes of the intervention.

	Study	Total number of withdrawals	Clinical outcomes (mean (SD) or n unless otherwise stated)	Intervention-specific outcomes [(n or mean (SD) unless otherwise reported)] and economic evaluations
10	Garcia et al., 2016a, 2016b; 2017	'Lost to FU'<br>I=0:C=2<br>Also 2 not evaluated and 3 excluded with no explanation. 3 others excluded for surgical/disease related reasons=10 not analyzed (making 22 ppts...)	**LoS days (median) (I=10:C=12)**<br>I=2:C=3, P=0.539 **no IQR**<br>**PO pulomnoary complications** Melbourne Group Scale **(I=10:C=12)**<br>I=5/10: C=8/12<br>scored positively in at least one item of the scale (P=0.361).<br>**Health-related QoL (SF-36)**<br>There were significant diffs in the physical component summary and bodily pain of the SF-36 (p<0.01)	**Outcomes reported (3 wks and 3 mth post surgery): Exercise capacity (endurance time); functional capacity (6MWT), muscle strength (Senior Fitness Test)**<br>No differences between groups at 3 wks in any of the main outcomes. At 3 mths, ppts in the I group had improved their endurance time (P-0.045), physical summary component (*p* = 0.001) and upper and lower body strength (*p* = 0.045 and *p* = 0.002) compared with C group.
11	Hoogeboom et al., 2010	C=1 but no reason given)	**LoS (median/range) (I=8:C=9)**<br>I=6 (5-22):C=6 (4-7), NS<br>*LoS days (mean/sd Quantile Estimation (QE) conversion method)*<br>*I=11.53 (13.08):C=5.67 (1.21)*<br>**Intraoperative complications**<br>I=2 (1 fractured femur during surgery, 1 had low saturation rate after surgery (89%) then diagnosed with intestinal obstruction)<br>HRQOL not assessed PO only compared preop to baseline	**Outcomes assessed: Chair rise test, timed up and go (TUG) test.**<br>No difference between groups.<br><br>**Adverse events**<br>During the intervention, no serious adverse events occurred
12	Kaibori et al., 2013	Lost to FU<br>I=14<br>C=10	**Mortality (in hospital)**<br>I=0/25:C=0/26<br>**LoS days I=25:C=26**<br>I=13.7 (4.0):C=17.5 (11.3), P=0.1200	
13	Licker et al, 2017; Karenovics et al, 2017	I=7:C=5<br><br>Withdrawal I=5:C=3<br>Operation cancelled I=2:C=2	**Mortality 30 day**<br>I=2/74:C=2/77, P=0.08<br>**LoS days median (IQR) I=74:C=77**<br>I=10 (8-12):C=9 (7-13), P=0.223<br>*LoS days (mean/sd Quantile Estimation (QE) conversion method)*<br>*I=10.00 (2.97):C=10.46 (5.29)*<br>**LoS in post anesthesia care unit (I=74:C=77)**<br>I=17 (7):C=25 (10), P<0.001<br>**Unplanned ICU admission (I=74:C=77)**<br>I=10 (13.5):C=14 (18.2), P=0.574<br>**Surgical complications**<br>**Reoperation**<br>I=8/74:C=2/77, P=0.089<br>**Bronchopleural fistula**<br>I=3/74:C=3/77, P=0.714<br>**Total Cardiovascular complications**<br>I=13/74:C=10/77, P=0.578<br>**Acute coronary syndrome:** I=2/74:C=1/77, P=0.972<br>**Acute heart failure:** I=2/74:C=0/77, P=0.460<br>**Pulmonary embolism**: I=2/74:C=1/77, P=0.972<br>**Stroke:** I= 1/74:C=0/77, P=0.984<br>**Arrhythmias:** I=11/74:C=8/77, P=0.560<br>**Wound infections**<br>I=3/74:C=4/77, P=0.957	**Outcomes reported: CPET parameters (FEV and FEC), 6MWT:**<br>Peak $V0^2$ at CPET increased significantly in I vs C (P<0.05)<br>mean distance in 6MWT increased in I vs C (P<0.05).<br>All other parameters NS

	Study	Total number of withdrawals	Clinical outcomes (mean (SD) or n unless otherwise stated)	Intervention-specific outcomes [(n or mean (SD) unless otherwise reported)] and economic evaluations
			**Renal dysfunction** I=2/74:C=4/77, P=0.731<br>**Total Respiratory complications**<br>I=17/74:C=33/77, P=0.009<br>**Acute Respiratory Distress Syndrome:**<br>I=2/74:C=1/77, P=0.972<br>**Ventilation (>6hr):** I=8/74:C=5/77, P=0.512<br>**Pneumonia:** I=8/74:C=15/77, P=2.09	
14	Lindback et al., 2018;<br>Fors et al., 2019	Administrative loss in part of the PROMs<br>I=9:C=5<br>But ITT analysis<br><br>For physical outcomes<br>I=80:C=91	The I group had better ODI, visual analog scale (VAS) back pain, EuroQol-5D (EQ-5D), EQ-VAS, Fear Avoidance Belief Questionnaire-Physical Activity (FABQPA), Self-Efficacy Scale (SES), and Hospital Anxiety and Depression Scale (HADS) depression scores and activity level compared with the waiting-list group after the pre-surgery intervention. However, these differences disappeared post-surgery.	**Outcomes reported: Activity levels**<br>higher activity level in the I group compared with the waiting-list group.<br><br>Ppts adhering,to ≥12 treatment sessions improved in all variables (P < 0.001 – P < 0.032) and those receiving 0-11 treatment session in only normal walking speed (P=0.035) but there were no differences when comparing dosages.
15	Maguire et al., 2018	NR	NR	**Outcomes reported: Muscle mass:Total psoas index (TPI) Priorto surgery**<br>There was a difference in TPI before and after the intervention on group comparison; P=0.021).<br>TPI declined in the C group (P=0.046) whilst the I group had a non-significant increase (P=0.292).<br>The intra- and inter-class correlation coefficient for TPI measurement variability were 0.985 and 0.957 respectively
16	Mak et al., 2019	NR	NR	**Outcomes reported: VO^2 max**<br>76% improved $V0^2$ max before surgery in I group.
17	Marchand et al., 2019	Preop assessment<br>I=2 due to early surgery.<br>C=n=3 (1 too stressed, 1 early surgery, 1 forgot appointment).<br><br>PO<br>I=1(due to personal reasons)<br>C=5 (due to 3 opted out of surgery, 1 health issues, 1 unknown)<br><br>3 mths PO<br>I=6 (1 did not perceive a change in their condition, 1 did not receive a questionnaire, 4 unknown).<br>C=5 (2 unknown, 3 opted out of surgery)	**LOS, days: I=20:C=17**<br>I=3.6(4):C=4.2(2.6), P=0.61<br>**Non-infective PO complications:**<br>**Blood loss (ml)**<br>I=120(141.8):C=220.6(269.2)<br>**Intraoperative complications (event/total no. ppts with data):**<br>I=0/20:C=1/17<br><br>**PO pain**<br>Numerical rating scale (NRS) back pain/10<br>Post-op:I= 2.7 (2.3):C=3.4 (2.8)<br>3mths PO= 1.8 (1.2):C=2.8 (2.4)<br>6mths PO= 2.9 (2.9):C=2.8 (3.2)<br>**NRS leg pain/10**<br>Post-op:I=2.9 (3.7):C=1.8 (2.7)<br>3mths PO=2.9 (3.1):C=1.8 (2.0)<br>6mths PO=3.5 (2.3):C=2.5 (1.9)	trunk muscle strength - flexion N.m<br>I=51.5(29.0):C=47.6(26.4)<br>trunk muscle strength - extension N.m<br>I=45.2(47.9):C=40.1(39.1)<br>lumbar active range of motion (degrees) - flexion<br>I=68(19):C=74(14)<br>lumbar active range of motion (degrees) - extension<br>I=18(7):C=15(6)<br>lumbar active range of motion (degrees) - left lateral flexion<br>I=14(5):C=14(5)<br>lumbar active range of motion (degrees) - right lateral flexion<br>I=14(6):C=17(6)<br>knee extensor strength (lbs)<br>I=63.3(39.7):C=58.4(30.4)<br>lumbar extensors endurance (secs)<br>I=45.5(43.6):C=43.7(55.2)<br>walking time to first symptoms (secs)<br>I=208.4(120.2):C=186.4(128.9)

Perry R, *et al. BMJ Open* 2022; 11:e050806. doi: 10.1136/bmjopen-2021-050806

	Study	Total number of withdrawals	Clinical outcomes (mean (SD) or n unless otherwise stated)	Intervention-specific outcomes [(n or mean (SD) unless otherwise reported)] and economic evaluations
		6 mths PO<br>I=6 (6 unknown)<br>C=5 (2 unknown, 3 opted out of surgery).	**HRQOL**<br>**Back pain disability /100**<br>Post-op:I=20.4 (15.4):C=15.5 (15.9)<br>3mths PO:I=20.3 (13.7):C=23.5 (13.5)<br>6mths PO:I=16.4 (19.0):C=22.9 (12.9)<br>**Kinesiophobia /68**<br>Post-op:I=39.8 (8.8):C=38.6 (9.6)<br>3mths PO=39.4 (7.9):C=41.1 (7.8)<br>6mths PO=37.5 (7.5):C=40.7 (10.5)<br>**Depression /63**<br>Post-op:I=2.7 (3.7):C=2.3 (3.6)<br>3mths PO=2.7 (2.6):C=4.2 (4.6)<br>6mths PO=2.1 (2.2):C=3.5 (3.5)	total ambulation time (secs)<br>I=259.6(72.7):C=274.1(53.7)<br>perceived change in global status<br>I=2.8(1.2):C=4.4(1.1)<br>% of group that reported improvements in global status<br>I=61:C=20<br>% of group that reported worsening in global status<br>I=5:C=33
18	McHugh 2011<br>PhD Thesis	1 ppt excluded	**LoS, days I=10:C=10**<br>Imputed SD (mean of all SD results)<br>I=5.4 (2.04):C=6.7 (2.49)<br>**HRQoL (SF-36**)<br>Physical Function<br>6wks preop<br>I=30.0(16.7):C=32.0(17.4)<br>Preop<br>I=37.0(17.7):C=26.4(16.0)<br>12wks PO<br>I=60.5(18.2):C=43.3(16.3)<br>**Mental Health**<br>6wks preop<br>I=78.8(18.4):C=78.0(17.5)<br>Preop<br>I=82.0(13.1):C=72.4(26.1)<br>12wks PO<br>I=83.2(15.8):C=77.2(17.1)<br>**Bodily pain**<br>6wks preop<br>I=40.5(17.3):C=33.7(25.4)<br>Preop<br>I=51.1(21.1):C=29.9(15.3)<br>12wks PO<br>I=73.6(16.7):C=68.4(18.6)	**Outcomes reported: Stair climb test, WOMAC**<br>6wks pre op<br>I=17.9(6.2):C=20.9(7.9)<br>Pre op<br>I=13.1(5.6):C=20.6(7.4)<br>12wks PO<br>15.8(7.0):C=15.8(3.8)<br><br>**WOMAC**<br>Pain<br>Lower in I group than C pre-operatively<br>Stiffness<br>Lower in I group than C P=0.023 12 wks PO
19	Nolan et al.,2019<br>Preliminary results conference abstract	NR	**LoS, days**<br>I =2.5 days decrease in Cardiac group vs 1 day decrease in Thoracic group) and physiotherapy length of stay (1.5 day decrease in both groups) in the prehabilitation groups.	All participants showed an increase in functional capacity from baseline to preop. As expected both groups had a drop in functional capacity in the postoperative in-patient phase, however, the prehab group had less of a decrease in both Cardiac and Thoracic groups
20	Northgraves et al., 2019	NR	**LoS [median and IQR]:I=10:C=11**<br>I= 10 (5-12):C=8(6-27)	**Outcomes assessed: Timed up and go (TUG), 6m walk test (6MWT)**

Perry R, *et al. BMJ Open* 2022; 11:e050806. doi: 10.1136/bmjopen-2021-050806

	Study	Total number of withdrawals	Clinical outcomes (mean (SD) or n unless otherwise stated)	Intervention-specific outcomes [(n or mean (SD) unless otherwise reported)] and economic evaluations
			LoS days (mean/sd Quantile Estimation (QE) conversion method) I=9.04 (2.62):C=12.97 (11.31)<br>**Total PO complications – infective**<br>I=3/10 C=4/11<br>**Total PO complications – non-infective**<br>related adverse events<br>I=0/10 C=0/11<br>unrelated adverse events<br>I= 1/10 C=1/11<br>**HRQoL[median(95%CI)]**<br>**physical functioning**<br>I= 93.3 (60; 100) C=100.0 (53.3; 100)<br>**role functioning**<br>I=100.0 (66.7; 100) C=100.0 (83.3; 100)<br>**emotional functioning**<br>I=75.0 (50; 100) C=100.0 (58.3; 100)<br>**cognitive functioning**<br>I=83.3 (83.3; 100) C=100.0 (83.3; 100)<br>**social functioning**<br>I=66.7 (16.7; 100) C=100.0 (100)<br>**global health**<br>I= 83.3 (33.3; 100) C= 83.3 (33.3; 100)<br>**anxiety**<br>I= 8.0(0; 11) C= 3.0 (0; 10)<br>**depression**<br>I= 2.0 (1; 9) C=1.0 (0; 4)	Timed up and go (TUG) difference baseline vs post op<br>I= 6.35(1.63):C=7.18(1.55)<br>5x sit to stand (FTSTS)<br>I=10.71(2.57):C=11.42(3.01)<br>Stair climb test (SCT) (in seconds)<br>I=2.47(0.46):C=3.02(0.72)<br>Handgrip dynamometry (HGD) right hand<br>I=28.8(11.6):C=28.6(7.3)<br>Handgrip dynamometry (HGD) left hand<br>I=26.1(10.1):C=27.3(7.0)<br>6m walk test (6MWT)<br>I=473.7(93):C=460.7(106)
21	Oosting et al., 2012	I=1 excluded at time point 3 (no surgery):C=2 excluded at timepoint 1 (1 lost to FU, 1 cancelled due to advanced surgery); 1 excluded at time point 3 (lost to FU)	**LoS days I=14:C=15**<br>I=5.1 (1.0):C=5.4 (2.1), NS<br>**Total PO Complications**<br>I=7/12:C=10/14<br>**Cardiac**<br>I=1/12:C=2/14<br>**Wound**<br>I=4/12:C=3/14<br>**Orthopedic**<br>I=1/12:C=1/14<br>**Loss of sensation**<br>I=1/12:C=0/14<br>**Shingles**<br>I=1/12:C=0/14<br>**Cognitive deficit/delirium**<br>I=0/12:C=2/14<br>**Renal**<br>I=0/12:C=1/14	**Outcomes assessed: Iowa Level of Assistance Scale, Longitudinal Aging Study Amsterdam Physical Activity Questionnaire, chair rise time, timed up and go, 6MWT, etc.).**<br>6MWT improved in I vs C (P<0.05) post intervention. All other outcomes NS. Chair rise time decreased significantly in I vs C (P<0.05) post-surgery. No difference between groups in all other parameters

	Study	Total number of withdrawals	Clinical outcomes (mean (SD) or n unless otherwise stated)	Intervention-specific outcomes [(n or mean (SD) unless otherwise reported)] and economic evaluations
			**Decubitus ulcers**<br>I=0/12:C=1/14<br>**Bowel obstruction**<br>I=0/12:C=1/14<br>**ILAS score <6 on day 4**<br>I=10/12:C=11/13<br>**Pain score (0-10) (I15:C=14)**<br>MD=0.0 (95% CI: -1.1 to -1.2), NS<br>**Health related QoL**<br>**HOOS** (5 subscales) – normalised scores of 0 to 100:<br>**Pain (I=14:C=12)**<br>MD=3.8 (95% CI: -3.1 to 10.7)<br>**Other symptoms (I=14:C=12)**<br>MD=-0.7 (95% CI: -10.7 to 9.3), NS<br>**Functioning in daily living (I=13:C=11)**<br>MD=4.4 (-7.2 to 16.0), NS<br>**Functioning in sports/recreation (I=8:C=10)**<br>MD=-4.2 (95% CI: -25.1 to 16.6), NS<br>**Hip-related QoL (I=14:C=12)**<br>MD=-9.4 (95% CI: -25.3 to 16.6), NS<br>P values all NR	
22	Rooks et al., 2006	30 ppts dropped out:<br>14 THA ppts (I=7:C=7)<br>16 TKA ppts (I=8:C=8)<br><br>Of which:<br>17 ppts dropped out prior to the end of intervention period (i.e., preop*);<br>I: 4 THA & 4 TKA<br>C: 5 THA & 4 TKA<br><br>*3 withdrew for medical issues unrelated to the study, 5 didn't like their group, 1 had bilateral hip arthroplasty, 2 couldn't commitment to exercise, and 6 cancelled surgeries.<br>13 ppts dropped out PO;<br>I: 3 THA & 3 TKA<br>C: 3 THA & 4 TKA<br>2 for medical issues unrelated to the surgery, 1 lost to FU by	**Discharged home**<br>I=24:C=20, P<0.05<br>THA I=14:C=14<br>TKA: I=10:C=6<br>**Discharged to inpatient rehabilitation facility**<br>I=12:C=23 NS<br>THA: I=6:C=12<br>TKA: I=6:C=11 NS<br>**Walk >50 feet at discharge**<br>I=27:C=21 P<0.05<br>**Walk < 50 feet at discharge**<br>I=9:C=22 NS	**Outcomes reported (pre- to post-intervention): WOMAC (function and pain), SF-36 (physical function, role functioning and pain); 1-repetition maximum, functional reach and timed up and go.**<br>In THA WOMAC physical function, SF-36 physical function and role functioning improved (P<0.05, P<0.01 and P<0.01, respectively) in I vs C,<br>In TKA SF-36 role functioning improved in I vs C (P<0.05).<br>No difference between I and C in all other outcomes, in THA or TKA.

	Study	Total number of withdrawals	Clinical outcomes (mean (SD) or n unless otherwise stated)	Intervention-specific outcomes [(n or mean (SD) unless otherwise reported)] and economic evaluations
		wk 8, and 10 lost to FU between wks 8 and 26.		
23	Santa Mina et al., 2018	Total from BL to 26 wks:<br>I=11:C=14<br><br>Preop<br>I=6:C=7<br>4 wks<br>I=1:C=1<br>12 wks<br>I=3:C=2<br>26 wks<br>I=1:C=4<br><br>IN THE TEXT: 9 and 21 ppts withdrew between BL and surgery and 26-wk FU, respectively; and 4 ppts did not undergo surgery (considered as dropouts, 2 at each center)	**LoS days I=38:C=35**<br>I=1.70 (0.9):C=1.76 (1.0), P=0.769<br>**PO complications: Frequency Grade 1/2/3/4**<br>I=8/9/1/0<br>C=7/6/0/1<br>**Total PO complications**<br>I=18/42:C=14/40, P=0.609<br>**No complications reported**<br>I=24/42:C=26/40<br>**Anxiety**<br>Anxiety lower in I at 26 wks (p=0.025).	**Outcomes reported at BL, pre-op, 4-wks, 12 wks and 26 wks post-op: 6MWT, upper-extremity strength, QoL, psychosocial wellbeing, urologic symptoms, and physical activity volume.**<br>Improvements in 6MWT (P=0.06) body fat percentage (p=0.001), erectile function (p=0.004), BMI (P=0.003), waist circumference (P=0.022) and grip strength at 22 wks (p=0.022) in I vs C.<br><br>**Adverse events**<br>total=5 (I=3:C=2), NS
24	Soares et al., 2013	Pre-surgery<br>Discontinued participation (surgery postponed/advanced)<br>I=2: C=3<br>Underwent surgery<br>I= 16:C=16<br><br>Excluded from analysis (died or surgery withheld /postponed/advanced)<br>I=3 :C=6	**Mortality 30 day**<br>I=1/16:C=3/16<br>**PO pulmonary complications**<br>I=5/15:C=11/13 (P = 0.03).<br>**atelectasis**<br>I=2/15: C=2/13<br>**atelectasis with pleural effusion**<br>I=0/15:C=2/13<br>**atelectasis with pneumonia**<br>I=0/15:C=1/13<br>**pneumonia**<br>I=1/15:C=0/13<br>**pneumonia with respiratory failure**<br>I=0/15:C=4/13<br>**pleural effusion with pneumothorax**<br>I=0/15:C=1/13<br>**pleural effusion**<br>I=1/15: C=1/13<br>**respiratory failure**<br>I=1/15:C=0/13	**Outcomes reported: Pulmonary function outcome variables (inspiratory and expiratory strength; respiratory muscle endurance and spirometry), and physical performance outcome variables were the functional independence measure and 6MWT** (in pre-operative period 13/16 ppts in I group increased walking distance vs 13/16 ppts in C group decreased walking distance; I=514.4 (460.8–557.5) vs C=441.5 (412.3–505.9))<br><br>In the preoperative period, ppts in the I group had higher inspiratory strength and respiratory muscle endurance than C group (88 (66–103) cmH2O versus 64 (40–84) cmH2O and 28 (22–32) cmH2O versus 23 (12–28) cmH2O, respectively; P < 0.05)
25	Tenconi et al 2017	12 ppts excluded after randmonisation:	**NR**	**Outcomes reported at interim analysis 6MWT, exercise capacity, FVC 6 MWT**<br>Improved in I vs C group (P=0.006)

	Study	Total number of withdrawals	Clinical outcomes (mean (SD) or n unless otherwise stated)	Intervention-specific outcomes [(n or mean (SD) unless otherwise reported)] and economic evaluations
		5 had unexpected lymph-nodal disease,<br>2 were unable to complete PO rehabilitation<br>5 withdrew their consent.		**Exercise capacity**<br>persists after surgery and comparison between the 2 groups shows a significant difference I vs C group; P=0.009<br>**forced vital capacity**<br>greater improvement in I vs C (P=0.003)
26	Tew et al., 2017	From text:<br>I=3:C=2<br>No longer undergoing surgery (I=1:C=1)<br>Withdrew after declining surgery (C=1)<br>Surgery expedited (I=1)<br>Did not have surgery (C=1)<br>.	**PO morbidity and mortality (N=48)**<br>I=2.3:C=2.1 (diff 0.2, 95% CI: -0.3 to 0.7)<br>There were no in-hospital or 30-day deaths in either group.<br>I=0/25:C=0/23<br>1 ppt in the I group died from a MI 12 wks after discharge from hospital.<br>**LoS, unadjusted median (IQR) days (I=25:C=23)**<br>I=7 (4.5-8.5):C=6 (4-8)<br>*LoS days (mean/sd Quantile Estimation (QE) conversion method)*<br>*I=6.67 (2.97):C=6.00 (2.97)*<br>The hazard ratio for discharge I vs. C groups was 0.96 (95% CI: 0.53 to 1.74).<br>**Health-related QoL**<br>**EQ-5D at 5 wks (I=25:C=24):**<br>I=0.864:C=0.796 (diff=0.068, 95% CI 0.002 to 0.135)<br>**EQ-VAS at 5 wks (I=25:C=24):**<br>I=81.9:C=75.8 (diff=6.1, 95% CI-0.3 to 12.6)<br>**EQ-5D at 12 wks (NI=21:C=22):**<br>I=0.837:C=0.760 (diff=0.077, 95% CI 0.005 to 0.148)<br>**EQ-VAS at 12 wks (I=21:C=22**<br>I=79.6:C=74.4 (diff=5.2, 95% CI -1.7 to 12.0)<br>**SF-36 PF at 5 wks (I=24:C=24):**<br>I=49.6:C=49.9 (diff=-0.3, 95% CI -2.7 to 2.1)<br>**SF-36 MH at 5 wks (I=25:C=24):**<br>I=54.6:C=55.1 (diff=-0.5, 95% CI -3.3 to 2.3)<br>**SF-36 PF at 12 wks (I=22:C=21):**<br>I=49.4:C=46.5 (diff=2.9, 95% CI 0.4 to 5.4)<br>**SF-36 MH at 12 wks (I=22:C=21):**<br>I=55.6:C=55.0 (diff=0.6, -2.4 to 3.6)<br>Adverse events: 1 non-serious (short-lived angina).	**Outcomes reported: cardiorespiratory fitness (anaerobic threshold and peak oxygen uptake) at wk 5 post surgery.**<br>No difference between groups.<br><br>*Other outcomes used to assess feasibility and acceptability were rates of: Screening, eligibility, recruitment, retention, outcome completion and adherence to exercise.*<br><br>**Costs**<br>**(means/sd)**<br>Cost of exercise programme = £176(0)<br>Total costs £12 519(3107): C=£12 009(3107)
27	Wang et al., 2002	None	No diff in the surgical complication rate between groups.<br>No wound infections, joint dislocations or complications requiring return to the operating room in either group.	**Outcomes reported: 6MWT; 25-m walk test (cadence, stride, length and gait velocity) measured at BL, 1-wk before and 3-, 12- and 24-wks post- surgery.**<br>There were no significant differences between the exercise and control groups for any of the gait variables before surgery.<br>The exercise group exhibited higher mean scores than the control group at every testing occasion and in every gait variable (P<0.05).

KEY: ARDS=Acute Respiratory Distress Syndrome; BL=baseline; C=control; CC= cardiovascular conditioning; CI=confidence interval; CPET= Cardiopulmonary exercise test; diff=difference; DVT=deep vein thrombosis; DXA=dual energy X-ray absorptiometry; ECC=experimental cardiovascular

conditioning; EPT= Experimental physical therapy; FEV=forced expiratory volume; FTSTS=five times sit to stand; FU=follow up; GI=gastrointestinal; group=group; HADS=Hospital anxiety and depression scale; HGD=handgrip dynamometry; HOOS=Hip disability and osteoarthritis outcome score; hr(s)=hour(s); HRQOL=health related quality of life; I=intervention; ICU=intensive care unit; ILAS score=Iowa Level of Assistance Score; IQR=interquartile range; ITT=intention to treat; L=litres; LAPAQ =Longitudinal aging study Amsterdam Physical Activity Questionnaire; LoS=length of stay; m=metre; mm=millimetre; MI=myocardial infarction; min(s)=minute(s); mth(s)=month(s); 6MWT=6-minute walk test; NR=not reported; NS=non-significant; PCA=patient controlled analgesia; PO=post-operative; ppt(s)=participant(s); preop=preoperative/preoperatively; PT=Physical Therapy; QoL=quality of life; ROM=range of motion; significant=significant; SCT=stair climb test; sd=standard deviation; SF-36=Social Function Health Assessment; SF36 PF=Social Function Health Assessment (physical function); SF 36 MH=Social Function Health Assessment (Mental Health); SIRS=Systemic inflammatory response syndrome; THA= Total Hip Arthroplasty; TKA=Total Knee Arthroplasty; TUG=timed up and go; VO2=Volume of oxygen; VAS=visual analogue scale; vs.=versus; WBC=whole blood count; wk=week; WOMAC= Western Ontario and McMaster Universities Osteoarthritis Index; yr(s)=year(s)

Perry R, *et al. BMJ Open* 2022; 11:e050806. doi: 10.1136/bmjopen-2021-050806

EXERCISE INTERVENTIONS

Table 10. Risk of bias

ID	Study	Selection bias: Random sequence generation	Selection bias: Allocation concealment	Performance bias: Blinding of participants (all outcomes)	Performance bias: Blinding of personnel(all outcomes)	Detection bias – Blinding of outcome assessment: Perioperative mortality, hospital readmission	Postoperative complications	Length of stay	Patient reported outcomes (pain, HRQoL)	Intervention related outcomes	Intervention related adverse events	Attrition bias: Incomplete outcome data	Reporting bias: Selective reporting
1	Argunova 2018<br>abstract					NR							
2	Banerjee 2014, 2018					NR	NR		NR		NR		
3	Barakat 2016												
4	Blackwell 2020					NR	NR	NR	NR				
5	Bridevaux. 2012<br>preliminary data -no ROB												
6	Carver 2011<br>abstract					NR	NR	NR	NR		NR		
7	Cavill 2016					NR	NR				NR		
8	D'Lima 1996												
9	Dunne, 2016					NR			NR				
10	Garcia 2016a,b; 2017					NR					NR		
11	Hoogeboom 2010					NR							
12	Kaibori 2013							NR	NR	NR	NR		
13	Licker 2017; Karenovics 2017								NR		NR		
14	Lindback 2018					NR	NR	NR			NR		
15	Maguire 2018<br>Abstract only					NR	NR	NR	NR		NR		

Perry R, *et al. BMJ Open* 2022; 11:e050806. doi: 10.1136/bmjopen-2021-050806

16	Mak 2019<br>Abstract only					**NR**			**NR**	**NR**	**NR**		
17	Marchand 2019					**NR**	from protocol				**NR**		
18	McHugh 2011			**NR**	**NR**	**NR**	**NR**			**NR**	**NR**		
19	Nolan 2019<br>preliminary data only: No ROB												
20	Northgraves 2020			from protocol		**NR**					**NR**		
21	Oosting 2012					**NR**	**NR**				**NR**		
22	Rooks 2006					**NR**	**NR**	**NR**			**NR**		
23	Santa Mina 2018					**NR**							
24	Soares 2013							**NR**	**NR**		**NR**		
25	Tenconi 2017<br>preliminary data only: No ROB												
26	Tew 2017												
27	Wang 2002					**NR**		**NR**	**NR**		**NR**		

NR = not reported

EXERCISE INTERVENTIONS

Table 11. Summary of findings

Exercise compared to usual care for any major surgery

Patient or population: any major surgery
Setting: hospital
Intervention: Exercise
Comparison: usual care

Outcomes	Anticipated absolute effects* (95% CI)		Relative effect (95% CI)	№ of participants (studies)	Certainty of the evidence (GRADE)	Comments
	Risk with usual care	Risk with Exercise				
mortality follow up: 30 days	34 per 1,000	**25 per 1,000** (8 to 81)	**RR 0.74** (0.23 to 2.35)	406 (5 RCTs)	⨁⨁◯◯ LOW [a,b]	
Length of Stay (LoS)		MD **0.38 days lower** (0.82 lower to 0.06 higher)	-	778 (15 RCTs)	⨁◯◯◯ VERY LOW [b,c,d]	
Total PO complications	401 per 1,000	**333 per 1,000** (245 to 450)	**RR 0.83** (0.61 to 1.12)	287 (5 RCTs)	⨁⨁◯◯ LOW [b,c]	
Post-operative Pulmonary Complications (PPCs)	396 per 1,000	**214 per 1,000** (155 to 297)	**RR 0.54** (0.39 to 0.75)	325 (4 RCTs)	⨁⨁◯◯ LOW [c,e]	
Pneumonia	144 per 1,000	**104 per 1,000** (50 to 207)	**RR 0.72** (0.35 to 1.44)	234 (3 RCTs)	⨁◯◯◯ VERY LOW [b,c,d]	

***The risk in the intervention group** (and its 95% confidence interval) is based on the assumed risk in the comparison group and the **relative effect** of the intervention (and its 95% CI).

CI: Confidence interval; **RR:** Risk ratio; **MD:** Mean difference

GRADE Working Group grades of evidence
High certainty: We are very confident that the true effect lies close to that of the estimate of the effect
Moderate certainty: We are moderately confident in the effect estimate: The true effect is likely to be close to the estimate of the effect, but there is a possibility that it is substantially different
Low certainty: Our confidence in the effect estimate is limited: The true effect may be substantially different from the estimate of the effect
Very low certainty: We have very little confidence in the effect estimate: The true effect is likely to be substantially different from the estimate of effect

Explanations

a. Downgrade inconsistency: based on effects not in same direction;
b. Downgrade for imprecision- includes null effect and appreciable benefit or harm, small sample size
c. Downgrade for Risk of bias,
d. Downgrade Inconsistency: based on effects not in same direction, different sizes of CI;
e. Downgrade for imprecision- small sample size

Meta analyses

Figure 29. Random effects meta-analysis of the risk ratio of mortality between exercise (experimental) and usual care (control).

Study or Subgroup	Experimental Events	Experimental Total	Control Events	Control Total	Weight	Risk Ratio M-H, Random, 95% CI
Barakat 2016	2	62	2	62	35.8%	1.00 [0.15, 6.88]
Kaibori 2013	0	25	0	26		Not estimable
Licker 2016:Karenovics 2017	2	74	2	77	35.6%	1.04 [0.15, 7.20]
Soares 2013	1	16	3	16	28.7%	0.33 [0.04, 2.87]
Tew 2017	0	25	0	23		Not estimable
**Total (95% CI)**		**202**		**204**	**100.0%**	**0.74 [0.23, 2.35]**
Total events	5		7			

Heterogeneity: Tau² = 0.00; Chi² = 0.74, df = 2 (P = 0.69); I² = 0%
Test for overall effect: Z = 0.51 (P = 0.61)

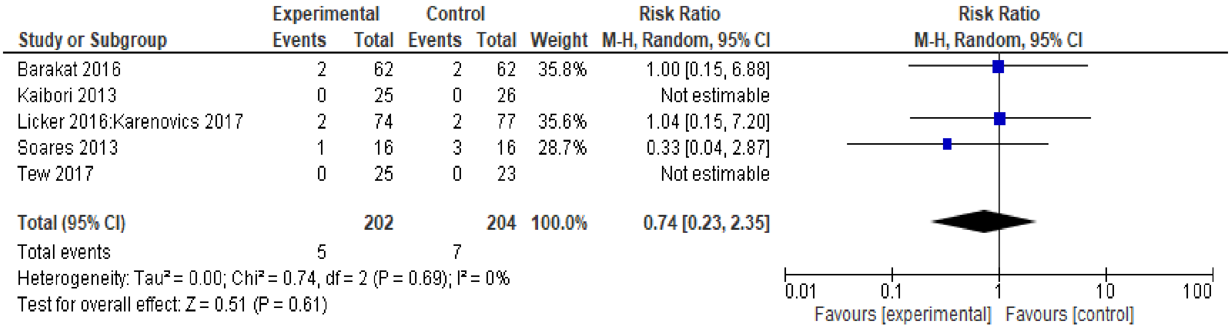

5/26 studies (406 participants).

Figure 30. Random effects meta-analysis of the mean difference in length of hospital stay (LoS) between exercise (experimental) and usual care (control).

Study or Subgroup	Experimental Mean	Experimental SD	Experimental Total	Control Mean	Control SD	Control Total	Weight	Mean Difference IV, Random, 95% CI
Argunova 2018	11.1	2.91	20	11.5	2.03	18	6.1%	-0.40 [-1.98, 1.18]
Banerjee 2018	17.8	37.45	27	22.31	61.22	28	0.0%	-4.51 [-31.23, 22.21]
Barakat 2016	7	2.97	62	9.57	5.5	62	6.3%	-2.57 [-4.13, -1.01]
Cavill 2016	6.9	2.4	29	6.9	2.7	30	8.3%	0.00 [-1.30, 1.30]
D'Lima 1996 ECC	6.3	1	10	6.1	1	10	14.0%	0.20 [-0.68, 1.08]
Dunne 2016	5	1.48	19	5.74	2.09	15	8.8%	-0.74 [-1.99, 0.51]
Hoogeboom 2010	11.53	13.08	8	5.67	1.21	9	0.2%	5.86 [-3.24, 14.96]
Kaibori 2013	13.7	4	25	17.5	11.3	26	0.9%	-3.80 [-8.42, 0.82]
Licker 2016:Karenovics 2017	10	2.97	74	10.46	5.29	77	7.8%	-0.46 [-1.82, 0.90]
Marchant 2019	3.6	4	20	4.2	2.6	17	3.7%	-0.60 [-2.74, 1.54]
McHugh 2011	5.4	2.04	10	6.7	2.49	10	4.2%	-1.30 [-3.30, 0.70]
Northgraves 2020	9.05	2.53	11	12.97	11.31	11	0.4%	-3.92 [-10.77, 2.93]
Oosting 2012	5.1	1	14	5.4	2.1	15	9.5%	-0.30 [-1.48, 0.88]
Santa Mina 2018	1.7	0.9	38	1.76	1	35	24.2%	-0.06 [-0.50, 0.38]
Tew 2017	6.67	2.97	25	6	2.97	23	5.6%	0.67 [-1.01, 2.35]
**Total (95% CI)**			**392**			**386**	**100.0%**	**-0.38 [-0.82, 0.06]**

Heterogeneity: Tau² = 0.16; Chi² = 18.66, df = 14 (P = 0.18); I² = 25%
Test for overall effect: Z = 1.70 (P = 0.09)

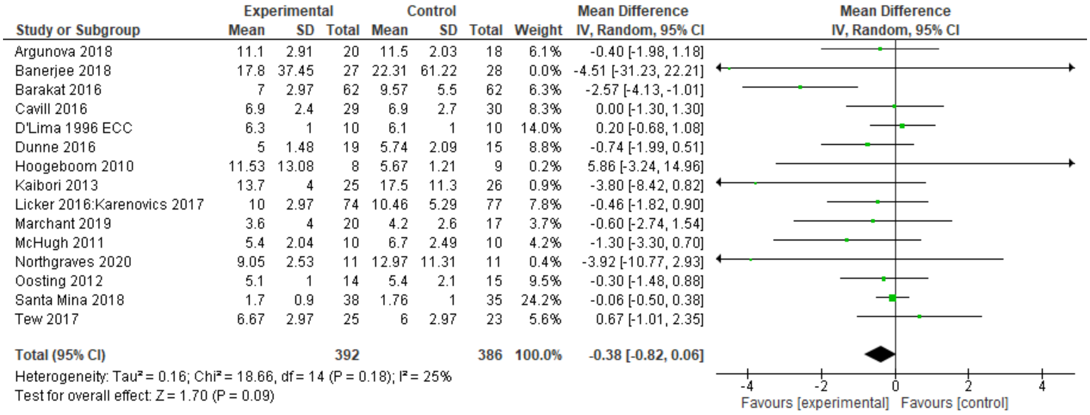

15/26 studies (778 participants). 1 abstract (McHugh et al., 2011) included.

Figure 31. Random effects meta-analysis of the risk ratio of total PO complications (infective & non-infective) between exercise interventions (experimental) and usual care (control).

	Experimental		Control			Risk Ratio
Study or Subgroup	Events	Total	Events	Total	Weight	M-H, Random, 95% CI
Barakat 2016	14	62	26	62	27.3%	0.54 [0.31, 0.93]
Dunne 2016	8	19	7	15	15.2%	0.90 [0.42, 1.92]
Northgraves 2020	3	10	4	11	6.0%	0.82 [0.24, 2.82]
Oosting 2012	7	12	10	14	24.4%	0.82 [0.46, 1.46]
Santa Mina 2018	18	42	14	40	27.1%	1.22 [0.71, 2.12]
**Total (95% CI)**		**145**		**142**	**100.0%**	**0.83 [0.61, 1.12]**
Total events	50		61			

Heterogeneity: Tau² = 0.01; Chi² = 4.42, df = 4 (P = 0.35); I² = 10%
Test for overall effect: Z = 1.22 (P = 0.22)

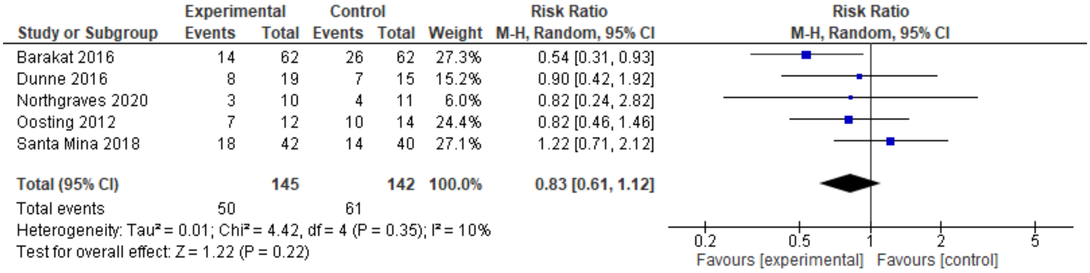

5/26 studies (287 participants).

Figure 32. Random effects meta-analysis of the risk ratio of pneumonia between exercise interventions (experimental) and usual care (control).

	Experimental		Control			Risk Ratio
Study or Subgroup	Events	Total	Events	Total	Weight	M-H, Random, 95% CI
Banerjee 2018	3	27	2	28	16.9%	1.56 [0.28, 8.59]
Licker 2016:Karenovics 2017	8	74	15	77	78.0%	0.55 [0.25, 1.23]
Soares 2013	1	15	0	13	5.1%	2.63 [0.12, 59.40]
**Total (95% CI)**		**116**		**118**	**100.0%**	**0.72 [0.35, 1.44]**
Total events	12		17			

Heterogeneity: Tau² = 0.00; Chi² = 1.86, df = 2 (P = 0.40); I² = 0%
Test for overall effect: Z = 0.93 (P = 0.35)

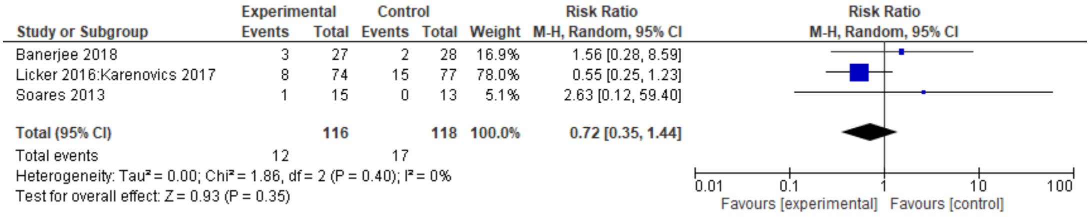

3/26 studies (234 participants).

Figure 33. Random effects meta-analysis of the risk ratio of PPCs between exercise interventions (experimental) and usual care (control).

	Experimental		Control			Risk Ratio
Study or Subgroup	Events	Total	Events	Total	Weight	M-H, Random, 95% CI
Barakat 2016	7	62	13	62	15.2%	0.54 [0.23, 1.26]
Garcia, 2016a,b;2017	5	10	8	12	20.1%	0.75 [0.36, 1.57]
Licker 2016:Karenovics 2017	17	74	33	77	45.4%	0.54 [0.33, 0.88]
Soares 2013	5	15	11	13	19.3%	0.39 [0.19, 0.84]
**Total (95% CI)**		**161**		**164**	**100.0%**	**0.54 [0.39, 0.75]**
Total events	34		65			

Heterogeneity: Tau² = 0.00; Chi² = 1.44, df = 3 (P = 0.70); I² = 0%
Test for overall effect: Z = 3.65 (P = 0.0003)

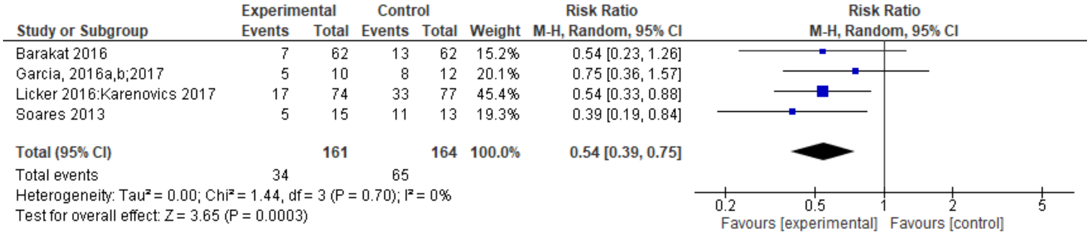

4/26 studies (325 participants).

Perry R, *et al. BMJ Open* 2022; 11:e050806. doi: 10.1136/bmjopen-2021-050806

Sensitivity analyses (removing studies at high risk of bias)

For mortality, 3 studies were removed due to high risk of bias (Kaibori et al., 2013, Licker et al., 2017, soares et al., 2013). Two studies (174 participants) remained but 1 study was not estimable (Tew et al., 2017), leaving just 1 study (Barakat et al., 2016), the RR=1.00 (95% CI, 0.15 to 6.88, P=1.00), demonstrating no effect of the intervention.

Figure 34. Random effects meta-analysis of the mean difference in length of stay between exercise interventions (experimental) and usual care (control) with high risk of bias studies removed.

	Experimental			Control				Mean Difference
Study or Subgroup	Mean	SD	Total	Mean	SD	Total	Weight	IV, Random, 95% CI
Banerjee 2018	17.8	3,745	27	22.31	61.22	28	0.0%	-4.51 [-1417.29, 1408.27]
Barakat 2016	7	2.97	62	9.57	5.5	62	17.3%	-2.57 [-4.13, -1.01]
Cavill 2016	6.9	2.4	29	6.9	2.7	30	20.2%	0.00 [-1.30, 1.30]
D'Lima 1996 ECC	6.3	1	10	6.1	1	10	25.8%	0.20 [-0.68, 1.08]
Dunne 2016	5	1.48	19	5.74	2.09	15	20.8%	-0.74 [-1.99, 0.51]
Tew 2017	6.67	2.97	25	6	2.97	23	16.0%	0.67 [-1.01, 2.35]
**Total (95% CI)**			**172**			**168**	**100.0%**	**-0.44 [-1.37, 0.49]**

Heterogeneity: Tau² = 0.67; Chi² = 11.38, df = 5 (P = 0.04); I² = 56%
Test for overall effect: Z = 0.93 (P = 0.35)

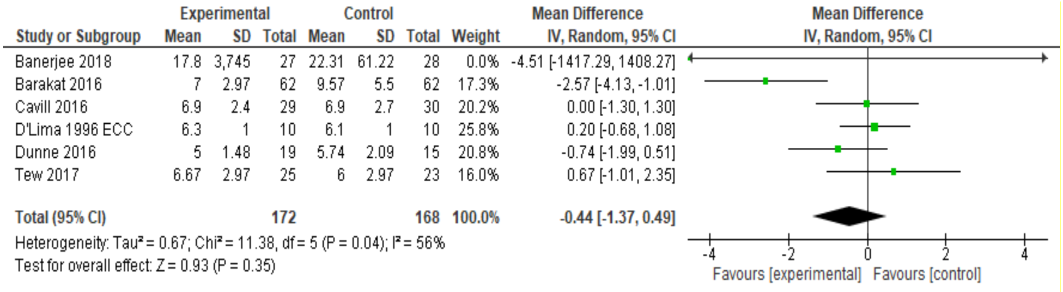

6 studies (340 participants) included and 6 studies excluded (Argunova et al., 2018, Hoogeboom et al., 2010, Kaibori et al., 2013, Licker et al., 2017, Oosting et al., 2012, Santa Mina et al., 2018).

Sensitivity analyses (removing studies with imputed results)

Figure 35. Random effects meta-analysis of the mean difference in length of stay between exercise interventions (experimental) and usual care (control) with imputed results removed. done

	Experimental			Control				Mean Difference
Study or Subgroup	Mean	SD	Total	Mean	SD	Total	Weight	IV, Random, 95% CI
Argunova 2018	11.1	1.91	20	11.5	2.03	18	7.3%	-0.40 [-1.66, 0.86]
Cavill 2016	6.9	2.4	29	6.9	2.9	30	6.3%	0.00 [-1.36, 1.36]
D'Lima 1996 ECC	6.3	1	10	6.1	1	10	15.0%	0.20 [-0.68, 1.08]
Kaibori 2013	13.7	4	25	17.5	11.3	26	0.5%	-3.80 [-8.42, 0.82]
Marchant 2019	3.6	4	20	4.2	2.6	17	2.5%	-0.60 [-2.74, 1.54]
Oosting 2012	5.1	1	14	5.4	2.1	15	8.2%	-0.30 [-1.48, 0.88]
Santa Mina 2018	1.7	0.9	38	1.76	1	35	60.2%	-0.06 [-0.50, 0.38]
**Total (95% CI)**			**156**			**151**	**100.0%**	**-0.10 [-0.44, 0.24]**

Heterogeneity: Tau² = 0.00; Chi² = 3.51, df = 6 (P = 0.74); I² = 0%
Test for overall effect: Z = 0.55 (P = 0.58)

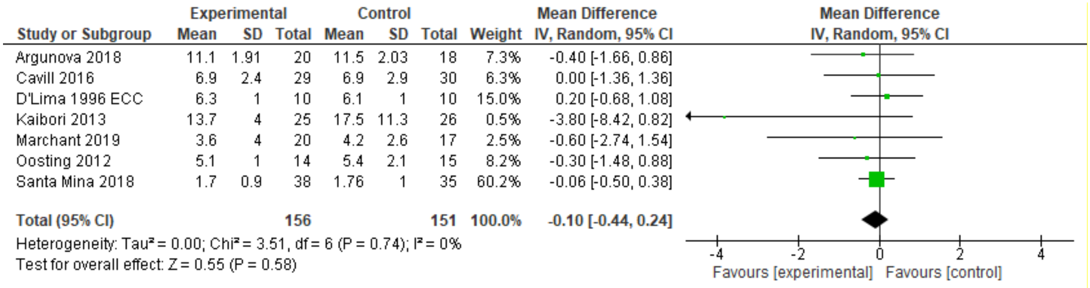

6 studies (270 participants) included and 8 studies excluded (Banerjee et al 2018, Barakat et al., 2016, Dunne et al., 2016, Hoogeboom et al., 2010, Kaibori et al., 2013, Twe et al., 2017, Licker et al., 2016, Northgraves et al., 2020, McHugh 2011)

Subgroup analyses (type of surgery)

Figure 36. Random effects meta-analysis of the mean difference in length of hospital stay (LoS) between exercise (experimental) and usual care (control) for patients undergoing cancer surgery.

	Experimental			Control				Mean Difference
Study or Subgroup	Mean	SD	Total	Mean	SD	Total	Weight	IV, Random, 95% CI
Banerjee 2018	17.8	37.45	27	23.31	61.22	28	0.0%	-5.51 [-32.23, 21.21]
Kaibori 2013	13.7	4	25	17.5	11.3	26	1.0%	-3.80 [-8.42, 0.82]
Licker 2016:Karenovics 2017	10	2.97	74	10.46	5.29	77	11.4%	-0.46 [-1.82, 0.90]
Northgraves 2019	9.04	2.62	10	12.97	11.31	11	0.5%	-3.93 [-10.81, 2.95]
Santa Mina 2018	1.7	0.8	38	1.76	1	35	87.1%	-0.06 [-0.48, 0.36]
**Total (95% CI)**			**174**			**177**	**100.0%**	**-0.16 [-0.63, 0.31]**

Heterogeneity: Tau² = 0.02; Chi² = 4.09, df = 4 (P = 0.39); I² = 2%
Test for overall effect: Z = 0.68 (P = 0.49)

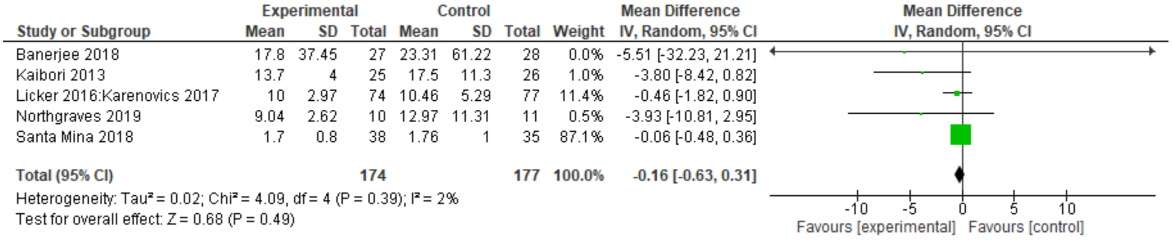

5 studies (351 participants) included and 10 studies excluded (Argunova et al., 2018, Barakat et al., 2016, Cavill et al., 2016, D'Lima et al., 1996, Dunne et al., 2016, Hoogeboom et al., 2010, Marchant et al., 2019, McHugh et al., 2011, Oosting et al., 2012, Santa Mina et al., 2018, Tew et al., 2017).

Subgroup analyses (studies published before and after 2010)

All studies that reported the outcomes of mortality, total postoperative complications and pneumonia were published after 2010, therefore a subgroup analysis was not necessary.

Figure 37. Random effects meta-analysis of the mean difference in length of hospital stay (LoS) between immunonutrition (experimental) and usual care (control) in studies published after 2010.

	Experimental			Control				Mean Difference
Study or Subgroup	Mean	SD	Total	Mean	SD	Total	Weight	IV, Random, 95% CI
Argunova 2018	11.1	2.91	20	11.52	2.03	18	7.4%	-0.42 [-2.00, 1.16]
Banerjee 2018	17.8	37.45	27	22.31	61.22	28	0.0%	-4.51 [-31.23, 22.21]
Barakat 2016	7	2.97	62	9.57	5.5	62	7.6%	-2.57 [-4.13, -1.01]
Cavill 2016	6.9	2.4	29	6.9	2.7	30	9.8%	0.00 [-1.30, 1.30]
Dunne 2016	5	1.48	19	5.74	2.09	15	10.4%	-0.74 [-1.99, 0.51]
Hoogeboom 2010	11.53	13.08	8	5.67	1.21	9	0.3%	5.86 [-3.24, 14.96]
Kaibori 2013	13.7	4	25	17.5	11.3	26	1.1%	-3.80 [-8.42, 0.82]
Licker 2016:Karenovics 2017	10.2	2.94	74	10.46	5.29	77	9.3%	-0.26 [-1.62, 1.10]
Marchant 2019	3.6	4	20	4.2	2.6	17	4.5%	-0.60 [-2.74, 1.54]
McHugh 2011	5.4	2.04	10	6.7	2.49	10	5.1%	-1.30 [-3.30, 0.70]
Northgraves 2020	9.05	2.53	11	12.97	11.31	11	0.5%	-3.92 [-10.77, 2.93]
Oosting 2012	5.1	1	14	5.4	2.1	15	11.2%	-0.30 [-1.48, 0.88]
Santa Mina 2018	1.7	0.9	38	1.76	1	35	26.2%	-0.06 [-0.50, 0.38]
Tew 2017	6.67	2.97	25	6	2.97	23	6.7%	0.67 [-1.01, 2.35]
**Total (95% CI)**			**382**			**376**	**100.0%**	**-0.47 [-0.96, 0.02]**

Heterogeneity: Tau² = 0.19; Chi² = 17.49, df = 13 (P = 0.18); I² = 26%
Test for overall effect: Z = 1.89 (P = 0.06)

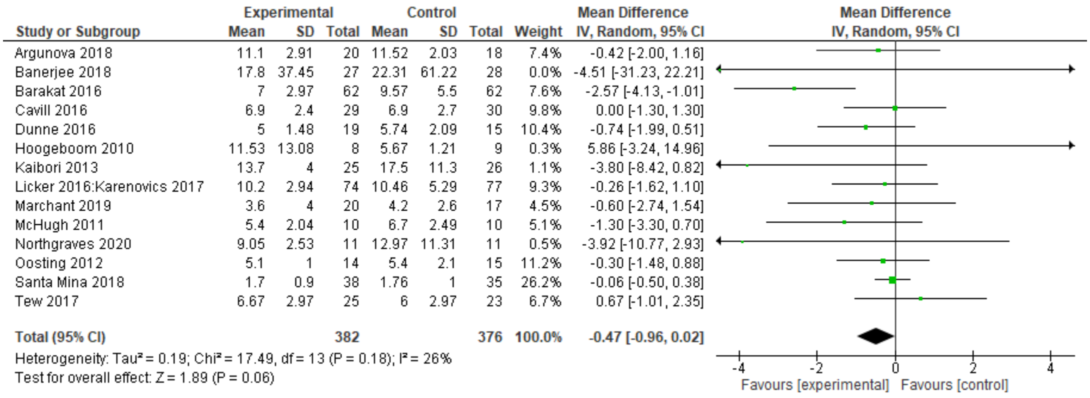

14 studies (768 participants) included and 1 study excluded (D'lima et al., 1996). 1 abstract (Mchugh et al., 2011) included.

References

Argunova, Y., Korotkevich, A., Pomeshkina, S., Kokov, A., Inozemtseva, A. & Barbarash, O. 2018. Efficacy of physical trainings as cardioprotection method for coronary bypass surgery. *Russian Journal of Cardiology,* 23**,** 159-165.

Banerjee, S., Manley, K., Shaw, B., Lewis, L., Cucato, G., Mills, R., . . . Saxton, J. M. 2018. Vigorous intensity aerobic interval exercise in bladder cancer patients prior to radical cystectomy: a feasibility randomised controlled trial. *Support Care Cancer,* 26**,** 1515-1523.

Banerjee, S., Manley, K., Shaw, B., Lewis, L., Rochester, M., Saxton, J. & Mills, R. 2014. *Is preoperative exercise to improve fitness before cystectomy feasible? Results from a randomised controlled study*.

Barakat, H. M., Shahin, Y., Khan, J. A., Mccollum, P. T. & Chetter, I. C. 2016. Preoperative Supervised Exercise Improves Outcomes After Elective Abdominal Aortic Aneurysm Repair: A Randomized Controlled Trial. *Ann Surg,* 264**,** 47-53.

Blackwell, J. E. M., Doleman, B., Boereboom, C. L., Morton, A., Williams, S., Atherton, P., . . . Lund, J. N. 2020. High-intensity interval training produces a significant improvement in fitness in less than 31 days before surgery for urological cancer: a randomised control trial. *Prostate Cancer and Prostatic Diseases.*

Bridevaux, P.-O., Tschopp, J.-M., Bhatia, C., Frésard, I., Triponez, F., Schnyder, J.-M., . . . Licker, M. 2012. Effect of pre-operative short-term rehabilitation on peak VO2 in patients with NSCLC. *European Respiratory Journal,* 40**,** 3306.

Carver, T. E., Mayo, N., Andersen, R. E. & Zavorsky, G. S. 2011. Pilot investigation to evaluate changes in exercise capacity following a prehabilitation intervention among seriously obese patients awaiting bariatric surgery. *Canadian Journal of Diabetes,* 35**,** 149.

Cavill, S., Mckenzie, K., Munro, A., Mckeever, J., Whelan, L., Biggs, L., . . . Haines, T. P. 2016. The effect of prehabilitation on the range of motion and functional outcomes in patients following the total knee or hip arthroplasty: A pilot randomized trial. *Physiother Theory Pract,* 32**,** 262-70.

D'lima, D. D., Colwell, C. W., Jr., Morris, B. A., Hardwick, M. E. & Kozin, F. 1996. The effect of preoperative exercise on total knee replacement outcomes. *Clin Orthop Relat Res***,** 174-82.

Dunne, D. F., Jack, S., Jones, R. P., Jones, L., Lythgoe, D. T., Malik, H. Z., . . . Fenwick, S. W. 2016. Randomized clinical trial of prehabilitation before planned liver resection. *Br J Surg,* 103**,** 504-12.

Fors, M., Enthoven, P., Abbott, A. & Oberg, B. 2019. Effects of pre-surgery physiotherapy on walking ability and lower extremity strength in patients with degenerative lumbar spine disorder: Secondary outcomes of the PREPARE randomised controlled trial. *BMC Musculoskeletal Disorders,* 20 (1) (no pagination).

García, R. S., Lista, P. A., Mi, Y.-B., E, G.-M., Salorio, R. M. & Borro, M. J. 2016. Preliminary efficacy of preoperative exercise training in patients with lung malignancies undergoing Video-Assisted Thoracic Surgery. *European Respiratory Journal,* 48**,** PA541.

Garcia, R. S., Paz, A. L., Brage, M. I. Y., Moolhuyzen, E. G., Rioboo, M. S. & Mate, J. M. B. 2016. Does preoperative exercise training prevent functional decline after video-assisted thoracic surgery? *European Respiratory Journal,* 48**,** PA538.

Garcia, R. S., Yanez-Brage, M. I., Moolhuyzen, E. G., Riobo, M. S., Paz, A. L. & Mate, J. M. B. 2017. Preoperative exercise training prevents functional decline after lung resection surgery: a randomized, single-blind controlled trial. *Clin Rehabil,* 31**,** 1057-1067.

Hoogeboom, T. J., Dronkers, J. J., Van Den Ende, C. H., Oosting, E. & Van Meeteren, N. L. 2010. Preoperative therapeutic exercise in frail elderly scheduled for total hip replacement: a randomized pilot trial. *Clin Rehabil,* 24**,** 901-10.

Kaibori, M., Ishizaki, M., Matsui, K., Nakatake, R., Yoshiuchi, S., Kimura, Y. & Kwon, A. H. 2013. Perioperative exercise for chronic liver injury patients with hepatocellular carcinoma undergoing hepatectomy. *Am J Surg,* 206**,** 202-9.

Karenovics, W., Licker, M., Ellenberger, C., Christodoulou, M., Diaper, J., Bhatia, C., . . . Triponez, F. 2017. Short-term preoperative exercise therapy does not improve long-term outcome after lung cancer surgery: a randomized controlled study. *Eur J Cardiothorac Surg,* 52**,** 47-54.

Licker, M., Karenovics, W., Diaper, J., Frésard, I., Triponez, F., Ellenberger, C., . . . Bridevaux, P. O. 2017. Short-Term Preoperative High-Intensity Interval Training in Patients Awaiting Lung Cancer Surgery: A Randomized Controlled Trial. *J Thorac Oncol,* 12**,** 323-333.

Lindbäck, Y., Tropp, H., Enthoven, P., Abbott, A. & Öberg, B. 2018. PREPARE: presurgery physiotherapy for patients with degenerative lumbar spine disorder: a randomized controlled trial. *Spine J,* 18**,** 1347-1355.

Maguire, S., Kinsella, J., Steele, R., Mutrie, N., Anderson, A. & Moug, S. 2018. Does prehabilitation modify muscle wasting in patients with rectal cancer undergoing neoadjuvant therapy? *Colorectal Disease,* 20 (Supplement 7)**,** 9-10.

Mak, T., Futaba, K., Leung, W. W., Ma, B., Lau, V. & Ng, S. 2019. Multidisciplinary prehabilitation programme on patients undergoing colorectal cancer surgery: A single centre randomised controlled trial. *Colorectal Disease,* 21 (Supplement 3), 16.

Marchand, A. A., Suitner, M., O'shaughnessy, J., Chatillon, C. E., Cantin, V. & Descarreaux, M. 2019. Feasibility of conducting an active exercise prehabilitation program in patients awaiting spinal stenosis surgery: a randomized pilot study. *Scientific Reports,* 9**,** 12257.

Mchugh, G. 2011. The role of perhabilitation on the outcome of total knee arthroplasty: a randomized control trial. *Role of perhabilitation on the outcome of total knee arthroplasty: a randomized control trial***,** N.PAG p.

Nolan, F., Lyon, K. & Lambie, N. 2019. Initial results: The effect of a physiotherapy prehabilitation programme on postoperative outcomes in patients undergoing cardiac or thoracic surgery. *Clinical Nutrition ESPEN,* 31**,** 111.

Northgraves, M. J., Arunachalam, L., Madden, L. A., Marshall, P., Hartley, J. E., Macfie, J. & Vince, R. V. 2020. Feasibility of a novel exercise prehabilitation programme in patients scheduled for elective colorectal surgery: a feasibility randomised controlled trial. *Support Care Cancer,* 28**,** 3197-3206.

Oosting, E., Jans, M. P., Dronkers, J. J., Naber, R. H., Dronkers-Landman, C. M., Appelman-De Vries, S. M. & Van Meeteren, N. L. 2012. Preoperative home-based physical therapy versus usual care to improve functional health of frail older adults scheduled for elective total hip arthroplasty: a pilot randomized controlled trial. *Arch Phys Med Rehabil,* 93**,** 610-6.

Rooks, D. S., Huang, J., Bierbaum, B. E., Bolus, S. A., Rubano, J., Connolly, C. E., . . . Katz, J. N. 2006. Effect of preoperative exercise on measures of functional status in men and women undergoing total hip and knee arthroplasty. *Arthritis Rheum,* 55**,** 700-8.

Santa Mina, D., Hilton, W. J., Matthew, A. G., Awasthi, R., Bousquet-Dion, G., Alibhai, S. M. H., . . . Carli, F. 2018. Prehabilitation for radical prostatectomy: A multicentre randomized controlled trial. *Surg Oncol,* 27**,** 289-298.

Soares, S. M., Nucci, L. B., Da Silva, M. M. & Campacci, T. C. 2013. Pulmonary function and physical performance outcomes with preoperative physical therapy in upper abdominal surgery: a randomized controlled trial. *Clin Rehabil,* 27**,** 616-27.

Tenconi, S., Galeone, C., Fugazzaro, S., Rapicetta, C., Piro, R. & Formisano, D. 2017. Perioperative and long-term effects of comprehnsive pulmonary rehabilitation on exercise capacity, postoperative outcome and quality of life in patients undergoing lung resection: A randomised controlled trial. *Interactive CardioVascular and Thoracic Surgery,* 25.

Tew, G. A., Batterham, A. M., Colling, K., Gray, J., Kerr, K., Kothmann, E., . . . Danjoux, G. 2017. Randomized feasibility trial of high-intensity interval training before elective abdominal aortic aneurysm repair. *Br J Surg,* 104**,** 1791-1801.

Wang, A. W., Gilbey, H. J. & Ackland, T. R. 2002. Perioperative exercise programs improve early return of ambulatory function after total hip arthroplasty: a randomized, controlled trial. *Am J Phys Med Rehabil,* 81**,** 801-6.

RESPIRATORY INTERVENTIONS

Table 12. Characteristics of studies

ID	1st Author, year and country	Total *n* Intervention (I) Control (c)<br><br>Number analysed (An)if reported	Patient population, baseline clinical characteristics (mean (SD) or *n (%)* unless otherwise stated	Demographics (mean (SD) or *n* unless otherwise stated)	Intervention	Comparator	Mode of delivery; place of delivery; training level of individuals who delivered the intervention; the number of contacts	Intervention fidelity; Compliance or adherence to intervention
**Inspiratory muscle training (IMT)**								
1	Barbalho-Moulim et al., 2011<br>Brazil	32 obese ppts undergoing Roux-en-Y gastric bypass.<br>I=15:C=17	**BMI kg/m^2**<br>I=41.4 (4.7)<br>C= 42.1 (3.0)<br>**W/H ratio**<br>I= 0.96 (0.09)<br>C=0.89 (0.07)<br>**Hypertension**<br>I=9:C=3<br>**Diabetes M**<br>I=3:C=3<br>**Dyslipidemia**<br>I=2:C=2	**Age yrs**<br>I=36.1 (8.1)<br>C=34.8 (9.5)	Use of threshold IMT<br>1 daily session lasting 15 mins<br>6x/wk for 2-4 wks before surgery	Usual Care	Ppts were instructed by staff on how to use the IMT device and record their data;<br>Self-administered at home;<br>Physiotherapist;<br>2-3 days before surgery	NR;<br>NR
2	Carvalho et al., 2011<br>Abstract only	32 ppts undergoing CABG with high risk of pulmonary complications	NR	**Age yrs**<br>I=62 (9.9)<br>C=62 (10.9)<br>**Gender (%M)**<br>I=62.5 :C=69	IMT workload of 30% maximal inspiratory pressure, 7 days a wk, 2x/day, 3 sets of 10 repetitions<br>2 wks prior to surgery	NR	NR;<br>Self-administered at home;<br>NR;<br>Were visited 4x in 2 wks+ 1 day before surgery	NR;<br>NR
3	Chen and Guo, 2018, Chen et al., 2019<br>China	200 ppts scheduled for cardiac surgery aged >50<br>I=100:C=100	**Smoking %**<br>I=45:C=37<br>**Diabetes Mellitus %**<br>I=25.5:C=27.3<br>**Hypertension%**<br>I=56:C=67.7<br>Hyperipidemia%<br>I=5.1:C=3	**Age, yrs**<br>I=61.7 (7.7)<br>C=61.7 (8.1)<br>**Gender (%M)**<br>I=74.5:C=68.7	IMT using the threshold Inspiratory muscle trainer (threshold IMT) device at 30% of MIP for 20 mins 2x/day for 5 days before surgery with supervision by a physical therapist .	Trained using the same protocol as intervention group for the same number of repetitions, frequency, duration and supervision but the intensity was fixed at the minimum load of the device (9cm H20) throughout the training period.	Verbal;<br>Hospital;<br>physical therapist;<br>daily IMT progression	NR;<br>NR
4	Da Cunha et al., 2013	16 ppts undergoing	**Esophageal cancer**	**Age, yrs**<br>I=55 (15)	Respiratory muscle training	Usual care	NR;<br>Assumed home;	NR;<br>NR

ID	1st Author, year and country	Total *n* Intervention (I) Control (c)<br><br>Number analysed (An)if reported	Patient population, baseline clinical characteristics (mean (SD) or *n (%)* unless otherwise stated	Demographics (mean (SD) or *n* unless otherwise stated)	Intervention	Comparator	Mode of delivery; place of delivery; training level of individuals who delivered the intervention; the number of contacts	Intervention fidelity; Compliance or adherence to intervention
	Brazil<br>Poster only	esophagectomy for megaesophagus or esophageal cancer<br>I=7:C=9	I=2/7:C=4/9<br>**Megaesophageal cancer**<br>I=5/7:C=5/9	C=54 (10)<br>**Gender (%M)**<br>I=75:C=50	3x12 repetitions using IMT and PEP devices. Resistance =60% of MIP or MEP<br>5x wk for at least 2 wks.		NR;<br>NR	
5	Dronkers et al., 2008<br>The Netherlands	20 ppts undergoing elective abdominal aortic aneurysm Surgery<br>I=10:C=10	**Smokers (N)**<br>I=6:C=6<br>**COPD (N)**<br>I=1:C=1<br>**BMI (kg/m^2)**<br>I=26:C=25	**Age, yrs**<br>I=70(6)*:<br>C=59 (6)<br>*significant difference<br>**Gender (%M)**<br>I=20:C=30	Training programme designed to increase the strength and endurance of the inspiratory muscles. IMT, 20% MIP delivered at home (6 sessions over 6 days). Min. 2 wks before surgery	Usual care consisting of instruction in (a) diaphragmatic breathing,24–26 (b) deep inspirations with the aid of incentive spirometer, and<br>(c) coughing and 'forced expiration techniques' (FET)	Face-to-face (supervised by physiotherapist) and self-delivery;<br>Home and hospital;<br>Physiotherapist;<br>Wkly supervised session	NR;<br>Patient questionnaire showed that acceptance of and compliance with IMT were high
6	Dronkers et al., 2010<br>The Netherlands	42 ppts undergoing elective surgery for colorectal cancer<br>I=22:C=20	Ppts had adequate cognitive functioning and age>60 yrs.15% had COPD. Excluded conditions that impeded exercise	**Age (yrs)**<br>I=71.1 (6.3)<br>C=68.8 (6.4)<br>**Gender (%M)**<br>I=68:C=80	Training programme:<br>resistance training of the lower limb extensors;<br>IMT, 10–60% MIP;<br>aerobic training:<br>training functional activities according to capabilities and interest.<br>2x60 min sessions/wk for 2-4 wks	Usual care (home-based exercise advice)	One-to-one and self-delivery;<br>Outpatient department and home;<br>Physical therapist;<br>One-to-one sessions 2x/wk	NR;<br>Adherence calculated as the percentage of scheduled visits to the outpatient department (97% in the I gp)
7	Ferreira et al., 2009<br>Brazil	30 ppts undergoing cardiac surgery<br>I=15:C=15	**BMI kg/m^2**<br>I=26.60 (5.17)<br>C=28.34 (3.16)<br>**NY Heart association classification**<br>class 1/2/3/4<br>I=7/5/2/1<br>C=10/3/2/0<br>**Ejection fraction < 35**<br>I=2:C=0	**Age (yrs)**<br>I= 62.5 (6.1)<br>C=63 (7.9)<br>**Gender (%M)**<br>I=60:C=87<br>**Ethnicity**<br>No race discrimination (but % NR)<br>**Smoker**<br>I=3(20%)<br>C=1(6.7%)<br>**Ex-smoker**	IMT using a device with a load corresponding to 40% MIP.<br><br>5 series of 10 calm and deep inspirations with 1+ minute intervals without feeling sick or tired.<br>3x day while waiting for surgery<br><br>Mean time between inclusion in programme and surgery was 154 (87.4) days	Usual care (general advice and no training general information (e,g, damaging effects of tobacco, encouraged not to smoke before surgery, deep - inspiration exercises without special equipment and daily walks up to their own limits)	Self-administered (following instructions);<br>Home based;<br>NR;<br>Assessed BL and surgery day	NR;<br>NR.

ID	1st Author, year and country	Total *n* Intervention (I) Control (c)<br><br>Number analysed (An)if reported	Patient population, baseline clinical characteristics (mean (SD) or *n (%)* unless otherwise stated	Demographics (mean (SD) or *n* unless otherwise stated)	Intervention	Comparator	Mode of delivery; place of delivery; training level of individuals who delivered the intervention; the number of contacts	Intervention fidelity; Compliance or adherence to intervention
			**Chronic atrial fibrillation**<br>I=3:C=3<br>**Heart failure**<br>I=1:C=0<br>**Previous MI**<br>I=4:C=7<br>**Arterial hypertension**<br>I=10:C=10<br>**Diabetes M**<br>I=6:C=6<br>**Carotid artery obstruction**<br>I=1:C=0<br>**Obstructive pulmonary disease**<br>I=1:C=1<br>**Pulmonary systolic pressure > 40 mmHg**<br>I=3:C=0<br>**Restrictive pulmonary disease**<br>**Mild**<br>I=1:C=0<br>**Moderate**<br>I=1:C=0<br>**Cerebral vascular accident**<br>I=2:C=0<br>**Peripheral vascular disease**<br>I=1:C=0	I=4/15 (27%)<br>C=4/15 (27%)				

ID	1st Author, year and country	Total *n* Intervention (I) Control (c)<br><br>Number analysed (An)if reported	Patient population, baseline clinical characteristics (mean (SD) or *n (%)* unless otherwise stated	Demographics (mean (SD) or *n* unless otherwise stated)	Intervention	Comparator	Mode of delivery; place of delivery; training level of individuals who delivered the intervention; the number of contacts	Intervention fidelity; Compliance or adherence to intervention
8	Huang et al., 2017<br>STUDY 1<br>China	60 ppts awaiting a lobectomy<br>I=30:C=30	**BMI % >30 kg/m^2**<br>I=13.3:C=16.7<br>**COPD%**<br>I=13.3:C=20.0<br>**Smoking%**<br>I=20.0:C=23.3<br>**Previous Thoracic surger %**<br>I=0:C=0<br>**Severity of illness%**<br>**ASA >III**<br>I=10:C=6.7	**Age, yrs**<br>I=64.1(5.3)<br>C=63.6(6.5)<br>**Gender (%M)**<br>I=70:C=70	Conventional single-mode IMT. IMT involved abdominal and thoracic breathing traing.<br>Abdomnial exercises: performed at least 43 x/day for 15–20 mins/session.<br>Thoracic breathing training: used a simple respiratory training device (Voldyne 5000, USA). Ppts performed these exercises for 20 mins at least 4x daily. NR how many wks before surgery.	Usual care: routine preoperative preparation, including preoperative education for in hospital	NR;<br>Hospital;<br>trained nurses/physiotherapist;<br>IMT exercise sessions supervised daily	NR;<br>92% in the IMT Group completed the 7-day rehabilitation period.<br>2 in the IMT Group dropped out due to loss of motivation.
9	Hulzebos et al., 2006b<br>The Netherlands<br>Feasibility study	26 ppts undergoing elective CABG<br>I=14:C=12	Ppts at high risk of developing PO pulmonary complications based in risk score	**Age yrs**<br>I=70.14 (9.86)<br>C=70.5 (10.10)<br>**Gender (%M)**<br>I=50:C=50<br>**Smoking %**<br>I=29:C=25	IMT, starting at 30% MIP, no trained daily at home, 7x/wk week, for at least 2 wks before surgery. Each training session consisted of 20 min of IMT and increased incrementally	Usual care: patient education re: early mobilisation and coughing with wound support received day before surgery	Face to face;<br>Home;<br>Physical therapist;<br>Wkly supervision.	NR;<br>Compliance: All ppts completed the intervention and reported high scores for motivation.<br>Everybody returned self-completion questionnaire score for satisfaction and motivation (out of 10) 7.9 (0.7) and 8.2 (1.0) respectively for intervention.
10	Hulzebos et al., 2006a<br>The Netherlands	279 ppts undergoing elective CABG<br>I=140:C= 139	Ppts at high risk of developing PO pulmonary complications based in risk score<br>**BMI kg/m^2**<br>**I=**28.3 (5.5)<br>C= 28.1 (3.2)<br>History of MI %<br>I=32.5:C=35.0<br>Hypertension %<br>I=57:C=44.5	Based on 139:137<br>**Age yrs**<br>I=66.5 (9.0):<br>C= 67.3 (9.2)<br>**Gender %M**<br>I=78:C=78<br>**NYHA classI/II/III/IV %**<br>I=19.4/15.1/63.3/2.3<br>C=3.6/15.1/76.5/2.2	IMT with a supportive education component (preoperative instruction in the active cycle of breathing techniques).<br>education in active cycle of breathing techniques; and forced expiration techniques trained daily, 7x/wk for at least 2 wks before surgery. Each session consisted of 20 mins of IMT, performed 6x/wk without and 1x/wk with supervision by a	Usual care: patient education re: early mobilisation and coughing with wound support received day before surgery	Face to face;<br>Home;<br>Physical therapist;<br>Wkly supervision.	NR:<br>The ppts were instructed to record daily IMT progression, complaints, and adverse events in a diary

ID	1st Author, year and country	Total *n* Intervention (I) Control (c)<br><br>Number analysed (An)if reported	Patient population, baseline clinical characteristics (mean (SD) or *n (%)* unless otherwise stated	Demographics (mean (SD) or *n* unless otherwise stated)	Intervention	Comparator	Mode of delivery; place of delivery; training level of individuals who delivered the intervention; the number of contacts	Intervention fidelity; Compliance or adherence to intervention
			Hypercholesterolemia %<br>I=25.9:C=26.3<br>**Type of surgery**<br>**On-pump CABG**<br>I=80.6:C=83.2<br>**Off-pump CABG**<br>I=19.4:C=16.8		physical therapist who measured the strength and endurance of the inspiratory muscles each wk.PPts were trained to breathe with an inspiratory threshold-loading device (threshold IMT). The ppts started breathing at a resistance equal to 30% of their max. inspiratory mouth pressure (Pi-max), measured at BL, for 20 mins. and increased incrementally, based on the rate of perceived exertion scored on the Borg scale.			
11	Jarosz et al., 2014<br>Poland<br>Abstract	100 ppts with non-small cell lung cancer undergoing lung resection.<br>No. randomized/ analysed not stated in abstract	NR	NR	2-wks intensive preoperative IMT	No IMT	NR – assumed under professional supervision;<br>Home;<br>NR;<br>NR	NR;<br>NR
12	Kulkarni et al., 2010<br>UK	80 ppts awaiting major abdominal or urological surgery, ASA grades I-IV.<br><br>I1 Deep breathing exercises=20 (An17)<br>I2 IS=20 (An15)<br>I3 IMT=20 (An17)<br>C=20 (An17)	NR	NR	I1: deep breathing exercises<br>I2 (IS): the training load was set depending on age and gender.<br>I3 (IMT): training load was set at 20-30% of baseline MIP.<br><br>For I1-I3 ppts were instructed to train 2x/d, each session lasting 15 mins for a minimum 2 wks before surgery.	Usual care	Verbal (ppts were instructed by the researcher on how to perform exercises);<br>Home;<br>“Researchers” instructed ppts, but their training level not stated;<br>NR	NR;<br>Ppts in I1 trained for 14 days (median; range, 6–35 days); ppts in I2 trained for 14 days (median; range, 10–50 days); ppts in I3 trained for 14 days (median; range, 8–28 days).

ID	1st Author, year and country	Total *n* Intervention (I) Control (c)<br><br>Number analysed (An)if reported	Patient population, baseline clinical characteristics (mean (SD) or *n (%)* unless otherwise stated	Demographics (mean (SD) or *n* unless otherwise stated)	Intervention	Comparator	Mode of delivery; place of delivery; training level of individuals who delivered the intervention; the number of contacts	Intervention fidelity; Compliance or adherence to intervention
13	Laurent et al., 2020 France	28 ppts who were eligible for NSCLC resection: lobectomy or pneumonectomy I =14:C=14	**BMI mean(SD) kg/m^3**<br>I= 25.8(5.9)<br>C=25.8(6.2)<br>20 ppts were ex-smoker and 2 were current smokers.<br>**Oncologic stage**<br>I=14:C=12<br>A-2<br>I=0%:C=18%	**Age, yrs**<br>I=64(7)<br>C=62(9)<br>**Gender (%M)**<br>I=64:C=75	12x 30min sessions of preoperative respiratory muscle endurance training (RMET) performed over 3 wks, with 2 consecutive days of RMET and 1 rest day, consisting of Isocapnic hyperpnoea added to usual chest physical therapy, the training started at 30% of maximal voluntary ventilation (MVV). The respiratory rate was increased every session by 1 cycle/min if the previous session lasted 30 mins.	12 usual preoperative chest physical therapy consisting of 30-min sessions performed for 3 wks.	Assume verbal training and used a sprotiger device for exercises;<br>3 learning sessions in hospital and then home;<br>physical therapist;<br>RMET was supervised 1x/wk	NR;<br>Adherence was considered when 9/12 sessions (75%) were completed.<br>The adherence to the RMET program was (86%)<br>Reasons for not reaching the minimal number of required sessions were paraneoplastic syndrome and tiredness.
14	Ma and Bao, 2009 China	32 ppts undergoing total hip replacement surgery<br>I=16 (15An)<br>C=16 (16An)	**COPD**<br>I=3<br>C=4	**Age, yrs**<br>I=66.4 (7.5)<br>C: 65.1 (8.2)<br>**Gender (%M)**<br>I=36:C=23<br>**Smoking**<br>I=2:C=2	IMT, 3 sessions/day using for about 4 days before surgery. Each session 20 mins of training and 5 mins rest. 1 session supervised other 2 were not. Starting threshold NR	Conventional treatment: diaphragmatic breathing, deep inspirations, coughing and “forced expiration techniques”	Supervised by experienced anaesthesiologist for first session;<br>NR but assumed hospital;<br>Experienced professional;<br>1 supervised session	NR;<br>NR
15	Sobrinho et al., 2014 Brazil	70 ppts undergoing cardiac surgery with cardiopulmonary bypass<br>I=35:C=35	**BMI, kg/m2**<br>**N=10:10**<br>I=27.1(4.0)<br>C=26.0(3.9)	**Age, yrs**<br>**N=10:10**<br>I:58.9(9.5)<br>C:61.4(8.4)<br>**Gender (%M)**<br>I=66:C=83<br>**Smokers (%)**<br>I=31:C=17	Written protocol guidance, breathing exercises and IMT Breathing exercises: breathing in time, deep breathing followed by prolonged expiration, sustained maximal inspiration with apnea of 6 seconds, and diaphragmatic breathing associated with the mobilization of the upper limbs). IMT: with threshold trainer starting resistance<br><br>For both breathing exercises and IMT 3 sets of 10 repetitions with an interval of 2 min between each repetition	Usual care (only orientation on the ward on the day of surgery)	Verbal and written;<br>Not clear, but indication that intervention was conducted in hospital;<br>Physiotherapist;<br>NR	NR;<br>NR but assume high as intervention appears to have been conducted in hospital
16	Valkenet et al., 2018	270 ppts with oesophageal	**BMI kg/m^2**<br>I=26.7 (4.8)	**Age yrs**<br>I=63.7 (7.5)	The intervention entailed the use of a tapered flow resistive	Usual care was not standardized, Thus, all	face-to-face instruction;<br>outpatient clinic;	NR;

ID	1st Author, year and country	Total *n* Intervention (I) Control (c)<br><br>Number analysed (An)if reported	Patient population, baseline clinical characteristics (mean (SD) or *n* *(%)* unless otherwise stated	Demographics (mean (SD) or *n* unless otherwise stated)	Intervention	Comparator	Mode of delivery; place of delivery; training level of individuals who delivered the intervention; the number of contacts	Intervention fidelity; Compliance or adherence to intervention
	Multicentred: 6 in the Netherlands, 1 in Belgium, 1 in Ireland 1 in Finland.	cancer undergoing oesophagectom y<br>I=132 (120An)<br>C=138 (121An)	C=26.5 (5.2)<br>**COPD**<br>I=19 (15·8): C=14 (11·6)<br>**Pneumonia in past 8 wks**<br>I=4·3: C=2·5<br>**Productive cough in past 5 days %**<br>I=16·2:C=21·7<br>**Cardiac history I=21·0:C=29·4**<br>**Diabetes mellitus**<br>I=14 (11·7)<br>C=13 (10·7)<br>**ASA 0-1/2/3-4%**<br>I=9.8/58.9/31.3<br>C=9.4/61.5/29.1	C=62.7 (8.9)<br>**Gender (%M)**<br>I=74:C=80<br>**Current smoker %**<br>I=15·8:C=16·7	inspiratory loading device. Starting inspiratory load was aimed at 60% of the baseline maximum inspiratory pressure, and was tailored on an individual basis during training	ppts received usual care according to local policies. IMT could not be part of usual care procedures during the trial.	physiotherapist;<br>BL + wkly telephone FU	adherence to the training and training progress was evaluated by wkly scheduled telephone interviews.<br>Ppts in the I group trained for a median 21 (0–74) days and 35 (0–130) sessions
17	van den Buijs et al., 2004<br>The Netherlands (Translation)	40 ppts undergoing CABG<br>I=21(20An)<br>C=19<br>1 ppt was not operated on for being too overweight (BMI=38)	**Diabetes melitus**<br>I=3(14) C=4(21)<br>**BMI, > 29 kg/m²**<br>I=7(33):C=9(47)<br>**COPD%**<br>I = 7 (33):C=3 (16)<br>**Cough %**<br>I=4 (19):C=3 (16)<br>**NYHA-class**<br>I=2-4:C=2-4	**Age, yrs**<br>I=58.9(9.5)<br>C=61.4(8.4)<br>**Overal Gender (%M)**<br>58<br>**Smoking n(%)**<br>I=5 (25):<br>C=5 (26)	IMT Threshold loading device 2 – 4 wks before surgery 7 x /wk 1xday total duration of 20 mins. Restistence was set at 30% of the Pimax and increased by 5% if able to breathe for 30 mins with this resisttance.	Usual Care	Face-to-face;<br>Home;<br>Physiotherapist;<br>1x/wk (2 –4)	NR;<br>Diaries were kept.<br>Compliance evaluated using a self-completed questionnaire, ppts reported being motivated to start and continue training.<br>18 (90%) ppt were able to train with increasing resistance, with a constant Borg score.
18	Weiner et al., 1998<br>Israel	84 ppts undergoing CABG<br>I=42:C=42	NR	**Age, yrs**<br>I=59.2 (3.8)<br>C=63.8 (3.1)<br>**Overall Gender (%M)**<br>69	IMT, resistance set at 15% MIP and increased incrementally (5% each session up to 60% MIP)<br>Trained 6x wk for 30 mins	Sham training<br>Breathed through the same muscle trainer but with no resistance	NR, but likely face-to-face<br>Hospital;<br>High;<br>NR	Weiner et al., 1998<br>Israel

ID	1st Author, year and country	Total *n* Intervention (I) Control (c)<br><br>Number analysed (An)if reported	Patient population, baseline clinical characteristics (mean (SD) or *n (%)* unless otherwise stated	Demographics (mean (SD) or *n* unless otherwise stated)	Intervention	Comparator	Mode of delivery; place of delivery; training level of individuals who delivered the intervention; the number of contacts	Intervention fidelity; Compliance or adherence to intervention
**Incentive Spirometry (IS)**								
19	Bergin et al., 2014<br>USA	104 ppts undergoing total hip replacement<br>I=72:C=69	**BMI (kg/m^2)**<br>I=30.52 (5.6)<br>C=30.57 (5.7)<br>**Asthma**<br>I=7(14):C=5(27)<br>**COPD**<br>I=2 (4.0%); C=3 (5.4%);<br>**Bronchitis**<br>I=4:C=6<br>**Pneumonia**<br>I=5: C=3<br>**Cardiovascular**<br>I=29:C=36	**Age**<br>I=61.6 (8.3)<br>C=65.7 (8.7)<br>**Gender (%M)**<br>43<br>**Ethnicity (% Caucasian)**<br>93<br>**Smokers**<br>I=5 (10)<br>C=6 (10.7)	Preoperative incentive spirometry education (*POISE*)<br>7 days preop:<br>Researchers instructed ppts with IS volumes below the predicted volume to use the IS 10x every 2 hrs while awake for 1 wk before surgery.<br>Ppts with IS volumes above the predictive volume were instructed to use the IS 10x every 6 hrs while awake.	Usual care	Written and verbal;<br>In hospital (bed community);<br>Researchers;<br>NR	NR;<br>Compliance for Group 1 patients regarding the preoperative and PO instructions for IS use was not measured"
20	Celli et al., 1984<br>Country NR	172 ppts undergoing abdominal surgery<br>IPPB=45<br>IS=42<br>DBE=41<br>C=44	NR	NR	I1: Intermittent positive pressure breathing (IPPB): 15 min 4x/day<br>I2: Incentive spirometry (IS): 4x/day<br>I3: Deep breathing exercises (DBE): 15 min, 4x/day.<br>Duration NR	Control (not stated, but assume usual care)	NR;<br>NR;<br>NR;<br>NR	NR;<br>NR
21	Cattano et al., 2010<br>USA	41 ppts undergoing laparoscopic bariatric surgery (including gastric bypass, gastric sleeve and gastric lap band)<br>I=20 (18An)<br>C=21 (19An)	Morbidly obese BMI ≥40kg/m^2<br>**BMI kg/m^2**<br>I=48.3 (6.9)<br>C=48.9 (5.7)	**Age yrs**<br>I=42.5 (12)<br>C=45 (12)<br>**Gender (%M)**<br>I=10:C=19	Use of incentive spirometer: 10 breaths, ≥5 attempts/day (as they were instructed to repeat 5 cycles of 10 attempts/day)<br>until the day of surgery (av. 6 days)<br>I=6.4 (3.3) days	Use of incentive spirometer (sham): 3 breaths 1x/day until the day of surgery<br>C=7.2 (4.9) days	Ppts were instructed by staff on how to use the spirometer/ record their data;<br>Self-administered at home;<br>NR but assume staff were trained in how to use spirometer;<br>None	NR<br>NR
	Kulkarni et al., 2010<br>UK	Study included IMT and IS interventions. Details provided under IMT section (No.12)						

ID	1st Author, year and country	Total *n* Intervention (I) Control (c)<br><br>Number analysed (An)if reported	Patient population, baseline clinical characteristics (mean (SD) or *n (%)* unless otherwise stated	Demographics (mean (SD) or *n* unless otherwise stated)	Intervention	Comparator	Mode of delivery; place of delivery; training level of individuals who delivered the intervention; the number of contacts	Intervention fidelity; Compliance or adherence to intervention
22	Leguisamo et al., 2005 Brazil	N=86 undergoing elective CABG surgery I=42:C=44	**Obesity (%)** I=26.2:C=20.5 **Advanced age (%) age NR** I=28.6:C=38.6 **COPD (%)** I=11.9:C=27.3	**Age yrs** I=59.3(8.4): C=60.6(10.9) **Gender (%M)** I=74:C=77 **Smokers (%)** I=28.6:C=20.5 **Ex-smokers (%)** I=62:C=77	Pulmonary function evaluation. Incentive spirometry 2x/day [3 (1) Diaphragmatic ventilatory pattern, (2) Ventilatory pattern with inspiration split in two parts (3) Ventilatory pattern with inspiration split in 3 parts, performed in two series of 10 repetitions of each type of exercise giving a total of 60 ventilatory exercises per series] until hospital admittance 24hrs before surgery at least 15 days before surgery. Education about coronary artery bypass surgery	Individual wkly appointment & verbal guidance of ventilatory exercises (no written materials given)	Face to face, written; Outpatients' clinic and home; Physiotherapists; At least 3 at the clinic (1initial appointment, at least 2 FUs)	NR; NR
**Combined Respiratory Interventions**								
23	Benzo et al., 2011 USA STUDY 1	9 undergoing lung cancer resection I=5:C=4	moderate-severe COPD	NR	Preoperative pulmonary rehabilitation for exercise prescription for 4 wks	Usual care	Face to face; Hospital (assumed); Physiotherapist; NR	NR; All 5 pps successfully completed 4 wks
24	Devecel and Senturan, 2018 Turkey	48 ppts undergoing open heart surgery (CABG, mitral value replacement, other) I=24:C=24	**Diabetes Mellitus %** I= 33:C=41 **Hypertension%** I=16:C=45 **Smoking** All ppts smoked until 8 wks before surgery	**Age, yrs** I=58.0(8.7) C=59.7(7.77) **Gender (%M)** I=54:C=70	Breathing exercises at least 7 day prior to operation. Education on the exercises given in a 15-min theoretical and practical training using a 'patient education booklet'. NR how often exercises done in 7 day period	Usual care.	Verbal and written; Hospital; NR; ppts were visited every 2 days	NR; NR
25	Lloréns et al., 2015 Spain	47 morbidly obese ppts undergoing laparoscopic bariatric surgery. 3 excluded after randomisation,	BMI >=40 kg/m^2 without COPD, bronchial asthma, or cardiac disease resulting in dysponea.	**Age, yrs** I:43.7(9.1) C:43.2(10.9) **Gender (%M)** I=48:C=57 **Smokers (%)** I=30:C=29	IMT and IS for 30 consecutive days before surgery + usual care (including postoperative IS)<br><br>Each preoperative daily session consisted of 20 min each of IMT	Usual care (including postoperative IS)	NR; NR; assume ppts were trained how to do the intervention in hospital and conducted daily sessions at home; Intervention delivered by trained physiotherapists; Wkly contacts with ppts.	NR; NR The physiotherapist evaluated the adherence of patients to the IMT programme weekly (assume ppts came to hospital for this).

Perry R, *et al. BMJ Open* 2022; 11:e050806. doi: 10.1136/bmjopen-2021-050806

ID	1st Author, year and country	Total *n* Intervention (I) Control (c)<br><br>Number analysed (An)if reported	Patient population, baseline clinical characteristics (mean (SD) or *n (%)* unless otherwise stated	Demographics (mean (SD) or *n* unless otherwise stated)	Intervention	Comparator	Mode of delivery; place of delivery; training level of individuals who delivered the intervention; the number of contacts	Intervention fidelity; Compliance or adherence to intervention
		I=24 (23)<br>C=23 (21)	**BMI, kg/m^2**<br>I=47.5(4.3)<br>C=51.6(6.9)		and IS. Starting load for IMT was 30% MIP			
26	Ortega et al., 2013<br>Spain<br>Abstract only	N=47 undergoing laproscopic bariatric surgery<br>I=24:C=23	Morbidly obese	NR	Repiratory physiotherapy program IMT and lung re-expansion exercises. 30 days presurgery 20 mins/day	No physiotherapy	NR;<br>NR;<br>NR;<br>NR	NR:<br>NR
27	Rajendran et al., 1998<br>Not stated (probably Indian subcontinent)<br>Abstract only	45 COPD ppts undergoing CABG surgery	Both groups evenly matched for duration and severity of COPD and coronary artery disease	"ppts of both groups were evenly matched with respect to age, sex, body surface area"	Pre-op short-term pulmonary rehab	No pulmonary rehab	NR;<br>NR;<br>NR;<br>NR	NR;<br>NR
28	Rovira Soriano et al., 2012<br>Moreno et al., 2012<br>(Abstracts)<br>Spain	29 morbidly obese ppts undergoing laparoscoic bariatric surgery<br>I=19:C=10	**Overall BMI (kg/m^2)**<br>46.1 (6.1)<br>Decreased lung volumes, chest wall compliance, raised intra-abdominal pressure, muscle inefficiency & decreased muscle endurance	NR	Respiratory physiotherapy program which included lung re-expansion (Voldyne5000) and inspiratory muscle training (threshold IMT) 20 mins a day, for 30 consecutive days before surgery.	Usual care	NR<br>NR<br>NR (probably physiotherapists);<br>NR	NR;<br>NR
29	Yamana et al., 2015<br>Abstract only	60 ppt undergoing oesophag-ectomy.<br>I=30:C=30	NR	NR	Intensive preoperative respiratory rehabilitation for 7+ days<br>All ppts underwent PO rehabilitation from the 1st PO day	Usual care<br><br>All ppts underwent PO rehabilitation from the 1st PO day	NR;<br>NR;<br>NR;<br>NR	NR;<br>NR

KEY: An=analysed; ASA=American Society of Anesthesiologists; BL=baseline; BMI=body mass index; C=control; CABG=coronary artery bypass graft; COPD=chronic obstructive pulmonary disease; DBE=deep breathing exercises; FU=follow up; gp=group; GP1/2=group1, group 2; I=intervention; IMT=inspiratory muscle training; IPPB=intermittent positive pressure breathing; IS=incentive spirometry; M=male; MEP=maximal expiratory pressure; min(s)=minute(s); MI=myocardial infarction; MIP=maximal inspiratory pressure; n or No.=number; NSCLC=Non-small-cell lung carcinoma; NR=not reported; NYHA=New York Heart Association; PEP=Positive Expiratory Pressure; Pimax=maximum inspiratory pressure; ppts=participants; PO=post-operative; rehab=rehabilliation; sd=standard deviation; W/H=waist to hip; wk(s)=week(s); yr(s)=year(s)

Perry R, *et al. BMJ Open* 2022; 11:e050806. doi: 10.1136/bmjopen-2021-050806

RESPIRATORY INTERVENTIONS

Table 13. Results

ID	Study	Total number of withdrawals	Clinical outcomes [n (%) or mean (SD) unless otherwise stated]	Intervention-specific outcomes [(n or mean (SD) unless otherwise reported)] and economic evaluations
**IMT**				
1	Barbalho-Moulim et al., 2011	0	**LoS days I=15:C=17**<br>I=2 (0.0): C=2.11 (0.33), P=0.571<br>**PO pulmonary complications (PPC)**<br>I=0/15:C=0/17<br>**Pain VAS I=15:C=17**<br>I =4.46 (1.30):C=4.35 (1.62)	**Outcomes reported: Respiratory muscle strength (MEP/MIP)**<br>**Training period**<br>**MEP**<br>The decrease in MEP from BL to end of training was similar between gps thus training did not influence the MEP.<br>**MIP**<br>There was an increase in the MIP only in the I group.<br>No change in lung volume (VC, VT, IRV, ERV, FVC, FEV1, and MVV) during training period.<br><br>**PO period**<br>there was a decrease in MIP and MEP in both groups, but a greater decrease in C vs I (47%vs 28%) implying that preoperative IMT attenuates the negative effects of open bariatric surgery in the inspiratory muscle strength (MIP) However, IMT did not appear to influence the lung volume and diaphragmatic excursion as there was a decrease in lung volume in both groups. The expiratory reserve volume (ERV) remained unchanged in I group only.
2	Carvalho et al., 2011 (Abstract/poster only)	NR	**Pneumonia**<br>I=0.85/16: C= 1.9/16, P=0.04<br>I=1/16:C=2/16<br>**Non-infective complications**<br>Lung collapse (atelectasis)<br>I=3.0/16: C=6.9/16, P=0.02	**Outcomes reported: MIP/MEP functional class (FC), PO (at 3- or 7-days PO), 6MWT**<br>Preoperative period<br>MIP values and FC showed less decrease and better recovery in I gp than C. In relation to MEP no difference between groups (p=0.28).<br>**PO**<br>PO MIP values and FC showed less decrease and better recovery in I group<br>**6MWT 7th day**<br>A difference (P<0.01) favouring the I vs. C
3	Chen et al., 2018;2019	I=2 (1 due to loss of motivation and 1 disharge from hospital)<br>C=1 (due to loss of motivation)	**LoS days I=98:C=99**<br>I=7.51(2.83):C=9.38(3.10)<br>**ICU hours (median (range))**<br>I=42.93(40.11-44.90):C=42.93(39.52-44.61)<br>*ICU Hours (mean/Sd Quantile Estimation (QE) conversion method)*<br>*I=43.05(0.99):C=42.79 (1.07)*	**Outcomes reported: Pulmonary function; Pi-max, FEV1 (% predicted), FVC (% predicted) , FEV1/FVC (% predicted), VC (% predicted), MVV (% predicted)**<br>Pulmonary Function compared to the control group, there were significant increase in FEV in the first second predicted, FVC

ID	Study	Total number of withdrawals	Clinical outcomes [n (%) or mean (SD) unless otherwise stated]	Intervention-specific outcomes [(n or mean (SD) unless otherwise reported)] and economic evaluations
			**Pneumonia**<br>I=3/98:C=7/99<br>**PO pulmonary complications (PPC)**<br>I=10/100:C=27/100	predicted and MVV predicted in the intervention group, respectively .<br>**FVC**<br>I= 88.49(16.41) to 92.06(16.24)<br>C=89.70(14.97) to 89.11(12.64<br>**FEV1**<br>I= 88.04(11.85) to 91.14(15.10)<br>C= 87.93(16.21) to 87.28(14.87)<br>**VC**<br>I= 87.55(21.92) to 91.37(23.76)<br>C= 90.43(14.26) to 89.66(13.95)<br>**Pi-max**<br>I= 86.93(23.03) to 100.08(23.36)<br>C=90.06(22.55) to 93.22(23.12)<br>**MVV**<br>I = 82.94(26.12 to 94.30(24.37)<br>C= 85.73(21.74) to 90.65(25.02)
4	Da Cunha et al., 2013	NR	NR	**Outcomes reported: MIP, MEP pre-operative period and PO; Lung function (2 and 30-day PO); 6 MWT.**<br>MIP and MEP increased in the preoperative in the RMT (although only MIP increment was significant), but not in the control group. In the postoperative period MIP and MEP had a similar behavior in both groups.<br>Lung function was decreased in the 2-day PO and returned to BL values in the 30-day PO in both groups.<br>No difference in 6MWT in both groups in the pre and PO periods.
5	Dronkers et al., 2008	None;<br>(2 ppts in the I gp could not be followed up for 7days after surgery because or re-operation; 2 ppts in the C gp FU was not possible)	**Perioperative mortality PO day 35**<br>I=1/10: C=0/8<br>**Infective complications (sepsis) PO day 35**<br>I=1/10: C=0/8 (reported in text)<br>**Atelectasis**<br>I=3/10:C=0/8, P=0.07<br>**Reoperation for blood vessel occlusion**<br>I=2/10: C=0/8 (reported in text)	**Outcomes reported: Inspiratory muscle endurance, MIP, Inspiratory muscle function, inspiratory vital capacity (IVC)**<br>**MIP** There was no difference between groups: ANCOVA analysis showed that the length of the training did not affect the change in MIP (P=0.97) and **inspiratory muscle endurance** (P=0.76).<br>**Inspiratory muscle function** recovered faster in the ppts in the I group; however, this improvement appeared not to affect the PO **inspiratory vital capacity**<br><br>**Adverse events**<br>No adverse events were reported
6	Dronkers et al, 2010	I=4/22	**LoS days (assumed)**<br>I=16.2 (11.5): C=21.6 (23.7)<br>**PO pulmonary complications (PPC)**<br>I=5/21: C=5/20, P=0.93<br>**Pneumonia**	**Outcomes reported: MIP, time-up-and-go, chair rise time, physical activity questionnaire, physical work capacity, abbreviated fatigue questionnaire, Respiratory muscle endurance:**

ID	Study	Total number of withdrawals	Clinical outcomes [n (%) or mean (SD) unless otherwise stated]	Intervention-specific outcomes [(n or mean (SD) unless otherwise reported)] and economic evaluations
			I=1/21:C=3/20, P=0.27<br>**Other PO complications**<br>I=9/21 C=8/20, P=0.65<br>**Quality of Life**<br>**EORTC QLQ-C30 BL and Post intervention [mean (SD)]**<br>**Global Health Status**<br>I=70(23) and 72(19): C=71(20) and 68(18), P=0.88<br>**Functional Scale**<br>I=408(67) and 413(64), C=247(53) and 425(67), P=0.72<br>**Symptoms Scale**<br>I=154(122) and 119(98): C=130(90) and 155(117), P=0.20	No diffs between groups in any of the above outcomes (p>0.05 or all) except for respiratory muscle endurance which improved in the I vs C group (P<0.01)
7	Ferreira et al., 2009	NR	**All-cause mortality 30 days**<br>I=3/15: C=1/15<br>**PO complications**<br>**Pneumonia** I =1/15: C=0/15,<br>**Renal failure** I=3/15: C=0/15,<br>**Heart failure** I= 1/15: C=1/15,<br>**Prolonged ventilation** I=1/15: C=0/15	**Outcomes reported: Manovacuometry, Temporal evolution of arterial PO2**<br>**Manovacuometry**<br>There was no significant difference between groups concerning (MIP) and Max EP (MEP).<br>**Temporal evolution of arterial PO^2**<br>No difference between groups<br><br>**Adverse events**<br>None of the ppts had to leave the program due to adverse events.
8	Huang 2017<br>STUDY 1	I=2 (loss of motivation)	**Mortality**<br>I=0/30: C=1/30<br>**LoS days I=30: C=30**<br>I=15.7(3.0): C=17.3(4.3)<br>**Pneumonia**<br>I=5/30:C=7/30<br>**Non-infective Complications**<br>**Anastomotic Leak(air leak >7 days)**<br>I=1/30: C=2/30<br>**Thromboembolism**<br>I= 1/30: C=1/30<br>**Pleural effusion needing tube**<br>I=2/30: C=2/30<br>**Respiratory failure**<br>I=0/30: C=1/30<br>**Atelectasis needing toilet bronchoscope**<br>I=1/30: C=1/30<br>**Empyema**<br>I=1/30: C=1/30<br>**Mechanical ventilation > 48h**<br>I=1/30: C=2/30<br>**Bronchopleural fistula**	**Outcomes reported: mean 6-MWD, Fatigue score, dyspnea score, PEF**<br><br>No differences were found in mean Fatigue score (Borg) and mean Dyspnea score (Borg) between the I group and C Group, No difference in mean PEF (P=0.127) and mean 6-MWD (P=0.740) was found between the Single IMT Group and Control Group.

ID	Study	Total number of withdrawals	Clinical outcomes [n (%) or mean (SD) unless otherwise stated]	Intervention-specific outcomes [(n or mean (SD) unless otherwise reported)] and economic evaluations
			I=0/30: C=1/30<br>**Chylothroax**<br>I=1/30: C=1/30<br>**Hospital Readmission**<br>I=1/30: C=1/30<br>**HRQoL(scored with EORTC QLQ-C30&LC13_CN (version 3))**<br>I=70.0(13.9): C=67.5(11.9)<br>**PO complications**<br>**Clavien-dindo Classification(CDC)**<br>**Grade I/II/III/IV/V**<br>14/5/3/1/0<br>16/8/3/2/1	
9	Hulzebos et al., 2006a	None	**LoS I=14:C=12**<br>I=7.93 (1.94): C=9.92 (5.78)<br>**PO complications**<br>Bronchitis – data NR<br>Pneumonia I=1/14: C=2/12, NS<br>X ray images: alterations in 19/26 cases:<br>I=6: C=5<br>Segmental Atelectasis I=2: C=6, P=0.05	**Outcomes reported: Mean increase in MIP (cmH20)**<br>I=36% (from 64.6 (15.8) to 87.6 (29.1))<br>C=15% (from 66.8 (26.3) to 76.8 (27.9))<br>Lung function<br>Predicted values improvements Pulmonary functions at BL and 1 day before surgery<br>**FVC**<br>I=88.6 (16) to 87.3 (18.1)<br>C=84.0 (18.6) to 87.4 (17.8)<br>**FEV1**<br>I=81.9 (20.0) to 80.7 (20.6)<br>C=80.8 (20.2) to 80.9 (20.3)<br>**IVC**<br>I =88.6 (16.0) to 87.3 (18.1)<br>C=84.00 (18.6) to 87.4 (17.8)<br>**$P_I$max**<br>I=64.60 (15.79): C= 66.80 (26.31)<br>No adverse events were reported
10	Hulzebos et al., 2006b		**LoS days I=139 C=137**<br>I=7.89 (2.17):C=9.94 (8.27)<br>**Level of PPC**<br>Grade 1 114 (82.0) 89 (65.0) P=0.02<br>Grade 2 14 (10.1) 18 (13.1).02<br>Grade 3 10 (7.2) 24 (17.5) 0.01<br>Grade 4 1 (0.7) 6 (4.4) 0.20 .09<br>PPC grade >2 25/139:C=48/137P=0.02<br>Pneumonia 9/139:C= 22/137 P=0 .01	
11	Jarosz et al., 2014 Poland (Abstract)	NR	**PO complications (non-infective)**<br>**I:** Lower frequency of PO complications. 2x fewer number of ppts required elongated pleural drainage/mechanical ventilation during PO cardiovascular and respiratory failure (P=0.0015)	**Outcomes reported: FVC/FEV1; 6MWT distance; Post-exercise saturation days**<br>**FVC/FEV1**<br>higher in I gp than C gp but NS<br>**6MWT distance**

ID	Study	Total number of withdrawals	Clinical outcomes [n (%) or mean (SD) unless otherwise stated]	Intervention-specific outcomes [(n or mean (SD) unless otherwise reported)] and economic evaluations
			C: higher rates of dyspnoea (P = 0.049) and weariness (P < 0001) after exercise during the PO period<br>**PO pain**<br>A test for homogeneity (P < 0.014) revealed less intensive postoperative pain in Days 1 and 4-9 in I Group.	longer in I gp than C gp especially on days 4-9/ 21-23<br>**Post-exercise saturation days**<br>I gp higher than C gp (4-9/ 21-23) after surgery<br>I gp had lower rates of dyspnea (P=0.049) and weariness (P < 0001) than C gp
12	Kulkarni et al., 2010	I1=3<br>I2=5<br>I3=3<br>C=3	**Pneumonia**<br>I1 (Deep breathing exercises) =1/17<br>**I2 (IS)=0/15**<br>I3 (IMT)=0/17<br>C=2/17	**Outcomes reported: MIP, MEP, VC, FVC, FEV1**<br>MIP increased by 33% from BL (P<0.01) at the preoperative time point in I3 (IMT); MIP decreased in all other groups from BL. Change from BL significantly different between I3 vs all other groups (P<0.01).<br><br>No difference in change from BL (preoperatively or postoperatively) in MEP, VC, FVC and FEV1 between groups.
13	Laurent et al., 2020	C=2	**Mortality**<br>I=0/14: C=1/12<br>**LoS days I=14:C=12**<br>I=7.6(3.3): C=8.5(4.7)<br>**ICU LoS hours**<br>I=62.4(57.6): C=112.8 (91.2)<br>**Chest tube duration days**<br>I=5.2(2.8): C=4.9(3.9)<br>**PO complications (Infective)**<br>I=0/14: C=0/12<br>**PO Complications (non-infective)**<br>**Thromboembolism**<br>I=0/14: C=1/12<br>**Pneumopathy**<br>I=1/14: C=4/12<br>**Prolonged Chest tube duration**<br>I=1/14: C=2/12<br>**Rhythm Disorder**<br>I=1/14: C=0/12<br>**Haemothorax**<br>I=2/14: C=0/12<br>**Recurrent paralysis**<br>I=0/14: C=2/12<br>**Chest wall hematoma**<br>I=0/14: C=1/12<br>**Empyema**<br>I=0/14: C=1/12<br>**Arterial hypertension**<br>I=0/14: C=1/12<br>**Respiratoy failure**<br>I=0/14: C=1/12	**Outcomes reported: results of isocapnic hyperpnoea endurance test, pulmonary function and exercise capacity.**<br>IHET (VE/ET) the groups did not significantly differ in VE and ET before RMET. VE and ET increased significantly after RMET in only the T group.<br>Pulmonary function test Before RMET, the I and C group did not differ in all PFT parameters, except MEP which was lower in the I group than C group. After RMET, the groups did not differ in any parameter<br>Exercise capacity Before RMET the I and C group did not differ in exercise capacity. After RMET the groups also did not differ.

ID	Study	Total number of withdrawals	Clinical outcomes [n (%) or mean (SD) unless otherwise stated]	Intervention-specific outcomes [(n or mean (SD) unless otherwise reported)] and economic evaluations
			**Distented bladder**<br>I=1/14: C=0/12<br>**Ventilatory Disorder requiring bronchoscopy**<br>I=0/14: C=1/12	
14	Ma & Bao 2009		**All cause perioperative mortality (30d)**<br>I =2/16: C=1/16<br>**LoS days [median, (range)] I=16:C=16**<br>I= 13 days (10-17): C=16 days (11-23)<br>LoS days (mean/sd Quantile Estimation (QE) conversion method)<br>*I=13.22 (2.31):C=16.41 (3.99)*<br>* 2 ppts followed up in first 7 days in each group received different treatment from different departments, ∴ not included in outcome<br>**PO complications (infective)**<br>**Pneumonia** I =1/15 : C= 0/16<br>**PO complications (non-infective)**<br>**Respiratory failure** I =1/15: C=1/16	**Outcomes reported: FVC (% predicted), IVC (litres), MIP (cmH20), FEV1 (% predicted) Pao2 (mmHg)**<br><br>**Changes from BL to post-intervention**<br>No between-group difference in mean MIP (P=0.11), IVC (P=0.23), Fev1 (P=0.23), PaO2 (P=0.06), FVC (P=0.13)<br>**PO day 1-7**<br>MIP & IVC higher in I vs C group from day2-7 (P<0.05)
15	Sobrinho et al., 2014	NR	**ICU LoS, days [median (range)] I=35:C=35**<br>I=1.8 (1.5-2): C=1.8 (0.6-1.9), NS<br>**LoS, days [median (range)]**<br>I=5.9 (4.7-7):C=6.9 (4.7-13.4), P<0.001<br>*LoS days (mean/sd Quantile Estimation (QE) conversion method)*<br>*I=5.87 (0.60):C= 7.41 (2.51)*	**Outcomes reported: MIP; MEP; Minute Volume (MV); Tidal Volume (TV); Respiratory Rate (RR)**<br>Changes in MIP, MEP and RR from BL not different between groups, either pre- or postoperatively.<br><br>Changes in MV and TV from BL different between groups at the preoperative timepoint (P=0.001 and P=0.03, respectively) but not postoperatively.
16	Valkenet et al., 2018	I: Did not receive allocated intervention=1<br>Ineligible, hearing impaired=1<br>Lost to FU=11<br>Discontinued intervention= 25<br>C Lost to FU=17	**Mortality (in hospital)**<br>I=5/120:C=3/121, P=0.48<br>**LoS days (mean/sd) I=120:C=121**<br>I=18·4(18·0): C=20·5(20·9), P=0.23<br>**ICU LoS days (mean/sd)**<br>I=3·3(7·5): C=3·1(6·6)<br>**Readmission ICU**<br>I=12 (10·0):C=20 (16·5)<br>**Retubation**<br>I=15/114:C=17/117<br>**Pneumonia**<br>I=47/120:C=43/121, P=0.56<br>**Antibiotics for suspected pneumonia**<br>I=47/120:C=40/120, P=0.33<br>**Pulmonary, other**<br>I=41/118:C=40/120, P=0.82<br>**Cardiac**<br>I=23/118:C=27/120, P=0.57<br>**Complications (other)**<br>I=17/120:C=26/118, =0.12	**Outcomes reported: respiratory muscle function (inspiratory muscle strength; preoperative inspiratory muscle endurance) and lung function measurements were performed by the blinded physiotherapist at 5 consecutive time points: BL (T0), before surgery(T1), and during hospital stay on PO days 3 (T2), 6 (T3) and 9 (T4)**<br>Maximal inspiratory muscle strength increased from in both groups. Preoperative inspiratory muscle endurance increased in both group increases were highest in the intervention group (P <0·050). Fatigue and physical activity level measures 4 weeks after surgery showed no differences between the groups<br><br>**Adverse events**<br>No serious adverse events were reported during the trial.

ID	Study	Total number of withdrawals	Clinical outcomes [n (%) or mean (SD) unless otherwise stated]	Intervention-specific outcomes [(n or mean (SD) unless otherwise reported)] and economic evaluations
			**Infection (other)**<br>I=10118:C=9/119<P=0.80<br>**Wound Infection**<br>I=8/117:C=6/119, P=0.38<br>**Sepsis**<br>I=1/118:C=6/119, P=0.78<br>**Positive sputum culture**<br>I=12/120:C=15/121, P=0.56<br>**Chyle leak**<br>I=4/118:C=10/118, P=0.64<br>**Vocal cord paresis**<br>I=4/118:C=10/118, P =0.11<br>**Anastomatic leak**<br>I=18/118:C=17/119, P=0.83<br>**Thromboembolism**<br>I=2.118:C=1/119, P=0.57<br>**Bleeding**<br>I=3/118:C=1/120, P=0.33<br>**Neuroligical**<br>I=6/116:C=4/120, P=0.49<br>**Delirium/confusion**<br>I=9/120:C=3/121, P=0.09<br>**Quality of Life (4 wks PO)**<br>Quality of life, fatigue and physical activity level measures showed no differences between the I and C group	
17	Van der Buis et al., 2004	1 dropped	NR (LoS could not be reported since ppt were sometimes transferred to other hospitals)	**Outcomes reported: Pi-Max**<br>PO day 1: PiMax decreased by more than 50% in both groups PO, but in the I group PiMax decreased less than in the C group (35% vs 29% of predicted respectively).<br><br>The recovery curve of the I group during the first 4 days of the PO period was clearly above that of the C group. Both groups followed the same line from day 5.
18	Weiner et al., 1998	NR	**PO pulmonary complications (PPC)**<br>I=8/42: C=9/42<br>**Pneumonia** I=1/42:C=3/42<br>**Pleural effusion** I=5: C=3<br>**Hemidiaphragmatic paralysis** I=2: C=3<br>11 (26%) ppts in the C group but only 2 (5%) in the I group needed postsurgical mechanical ventilation longer than 24 hrs.	Outcomes reported: FEV1<br>after the operation there was a significant decrease in the FEV1 values, and in the FVC values, from in the C gp but not in the I gp. (between group data NR)<br>**PO**<br>There were differences in both the MIP and endurance between the 2 groups (P=0.001) favouring IMT for a period of 2 to 4 wks before surgery resulted in a significant increase in MIP and endurance before the surgery and led to significantly better blood gases and pulmonary function after the operation.

ID	Study	Total number of withdrawals	Clinical outcomes [n (%) or mean (SD) unless otherwise stated]	Intervention-specific outcomes [(n or mean (SD) unless otherwise reported)] and economic evaluations
				There was a sign decrease in respiratory muscle function, PFTs, and gas exchange in the C gp following CABG, whereas these parameters remained similar to those before entering the study in the training group. The diff between groups were statistically different
**Incentive Spirometry**				
19	Bergin et al., 2014	34/140 enrolled did not complete the study for the following reasons:<br>7 no longer eligibility (I=4; C=3)<br>11 had a cancelled surgery (I=7; C=4)<br>9 had missing IS data (I=6; C=3)<br>7 withdrew (I=4; C=3)	**LoS I=50:C=56**<br>I=2.5 (0.6): C=2.7 (0.6), P 0.226<br>**PO pulmonary complications (PPC)**<br>I=1/50: C=3/56, P=0.62<br>**Non-pulmonary complications**<br>I=26/50: C=34/56, P=0.43<br>**Pain**<br>I For average pain at return to IS BL volume, there was a trend for lower scores in the I group both overall (2.72 vs 3.17, P=5.55) and for knees (2.90 vs 3.65, P=5.50), but neither trend was statistically significant.	**Economic Effect**<br>Daily mean hospital charges from Day 1 through discharge were lower for Group 1 ($1,175, SD=$185; average total per patient charge= $2,782, SD= $986) than for Group 2 ($1,285, SD=$214; average total per patient charge=$3,451, SD=$1,003)."
20	Cattano et al., 2010	4<br>I=1:C=3	**LoS**<br>no ppt required a hospital stay longer than 3 days<br>**PO pulmonary complications (PPC)**<br>I=0/18: C=0/19<br>**PO pain**<br>I=2/18 moderate/severe pain<br>C=2/19 moderate/severe pain	**Outcomes reported: Inspiratory capacity (IC) volume (cm^3)**<br>Preoperative (day of surgery):<br>IC volumes measured preoperatively (day of surgery) and postoperatively in ppts by group. Both the I and C groups showed a significant difference in IC lung volume. The % loss for the C group was 31%, and for the experimental group, it was 36%.<br>No differences were found between the I and C when comparing day of surgery and PO day 1<br>**Preoperative and PO IC volumes.**<br>The groups were divided further by BMI. The I group with BMI >50 showed an improvement (31% vs 44%) of IC lung loss.
21	Celli et al, 1983	0	**LoS, days I=42:C=44**<br>IS=8.6 (3.0): C=13.0 (5.0)<br>**PO pulmonary complications (PPC)**<br>Intermittent positive pressure breathing (IPPB)=10/45<br>IS=9/42<br>Deep breathing exercises (DBE)=9/41<br>C=21/44	NR
22	Kulkarni et al., 2010	I1=3<br>I2=5<br>I3=3<br>C=3	**Pneumonia**<br>I1 (Deep breathing exercises) =1/17<br>**I2 (IS)=0/15**<br>I3 (IMT)=0/17<br>C=2/17	**Outcomes reported: MIP, MEP, VC, FVC, FEV1**<br>MIP increased by 33% from BL (P<0.01) at the preoperative time point in I3 (IMT); MIP decreased in all other groups from BL. Change from BL significantly different between I3 vs all other groups (P<0.01).

ID	Study	Total number of withdrawals	Clinical outcomes [n (%) or mean (SD) unless otherwise stated]	Intervention-specific outcomes [(n or mean (SD) unless otherwise reported)] and economic evaluations
				No difference in change from BL (preoperatively or postoperatively) in MEP, VC, FVC and FEV1 between groups.
23	Leguisamo et al., 2005	Mortality prior to surgery<br>I=0/42:C=1/44<br>chose not to participate<br>I=2:C=0<br>Did not undergo surgery<br>I=1:C=0	LoS days I=42:C=44<br>I=11.77 (6.26):C=14.65 (6.61), P<0.005<br>LoS days (median IQR)<br>I=9.0 (8.0-12.8):C=12.0 (9.0-19.0)<br>**PO pulmonary complications (PPC)**<br>**PO day 1**<br>non-ventilated areas I=13/42:C=22/44<br>non-ventilated áreas and pleural effusion I=7/42:C=3/44<br>pleural effusion I=3/42:C=4/44<br>without complications I=19/42:C=15/44<br>**PO day 6**<br>non-ventilated areas I=0/42:C=5/44<br>non-ventilated áreas and pleural effusion I=16/42:C=15/44<br>pleural effusion I=19/42:C=12/44<br>without complications I=7/42:C=12/44<br>**total I=35/42 :C=32/44**	**Outcomes reported: FVC; FEV_1; Pulmonary volumes (peak inspiring pressure).**<br>**Change from baseline:**<br>No difference between gps.
**Combined Respiratory Interventions**				
23	Benzo et al., 2011 STUDY 1	NR	**LoS, PO complications (non-infective), adverse events:**<br>No diffs found on any outcome but no actual results reported	**NR**
24	Devecel et al., 2018	NR	**LoS, days**<br>I=12.04(1.83): C=12.92(2.04)<br>**ICU hrs**<br>I=3.29(0.46): C=3.67(0.63)<br>**Duration of extubation hours**<br>I=8.27(1.58) :C=9.17(1.90)<br>**Non-infective complications**<br>Lung collapse (atelectasis)<br>0 Day PO: I= 17(71%), P=0.003: C= 22 (92%), P=0.001<br>1st day PO: I=13(54%) :C=17 (71%)<br>2nd Day PO: I=7(29%) :C=10(42%)	**NR**
25	Llorens et al., 2015	3 ppts excluded after randomisation (I=1:C=2) due to violations of study protocol	**PO pulmonary complications**<br>I=0/23:C=0/21<br>**Adverse events**<br>I=0/23:C=1/21 (suture dehisence requiring re-operation)	**Outcomes reported: FVC, FEV1, MIP and MEP.**<br>**FVC**, **FEV1** and **MEP**: no difference between I vs C (BL, post-intervention and 12 hr PO).<br>**MIP**: increased by 18.7% from BL to post-intervention time point.
26	Ortega et al., 2013		**NR**	**Outcomes reported: FVC, FEV1, PiMAX, PeMAX pre and post training.**

ID	Study	Total number of withdrawals	Clinical outcomes [n (%) or mean (SD) unless otherwise stated]	Intervention-specific outcomes [(n or mean (SD) unless otherwise reported)] and economic evaluations
				PaO2/FiO2 ratio measured a) 5m after induction of anaesthetic b) 15 min after pneumoperitoneum, withdrawal c) 1h post-surgery 4) 24h post-surgery.<br>**PiMAX and PeMAX**<br>Only results reported – sig. increases in PiMAX and PeMAX (P=0.01) and PO2/FiO2 ratio higher for intervention group (P=0.04 at time 1 and P=0.002 at time 3 – not reported at time 2 and 4)
27	Rajendran et al., 1998	NR	**PO ventilation time (hrs) N=NR**<br>I=24.5 (6.00):C=35.2 (22.3)<br>**PO complications**<br>I=4:C=11 (N NR)<br>**LoS days N NR**<br>I=12.4 (3.6):C=18.8 (6.6)<br>Diffs described as 'significant'	**Outcomes reported: Pre-op and PO; peak expiratory flow rate and inspiratory capacity;**<br>both improved significantly in I vs C but P values not reported
28	Roviro et al., 2012; Moreno et al, 2012	0	An increase in P02/Fi02 at all time points (5 mins after induction; 15mins after pneumoperitoneum withdrawal; 1 hr after surgery; 24 hrs after surgery).<br>**24hrs surgery P02/Fi02**<br>The data shown an increase in the P02/Fi02 ratio at all time points for the study group respect control group, although results were only statistically significant (P=0,021) for T4.	**Outcomes reported: Respiratory muscle strength:**<br>Respiratory muscle strength parameters were higher in the IMT gp at all time-points though no statistical significance was reached.
29	Yamana et al., 2015	NR	**PO pulmonary complications**<br>**Clavien-Dindo classification (CDC)**<br>The CDC grade in the I gp was lower than that in the C group (P=0.014)<br>**Utrecht Pneumonia Scoring System (UPSS)**<br>The UPSS score in the I gp was lower than that in the C group at PO day 1 (P=0.031)<br>In the multivariate analysis, C group was an independent risk factor for PO pulmonary complications greater than CDC grade II (OR: 3.99, 95% CI: 1.28-12.4, P=0.017).	NR

KEY: 6MWT=6 minute walk test; arterial PO2= artierial pressure; BL=baseline; C=control; DBE=deep breathing exercises; diffs=differences; EORTC QLQ-C30= European Organization for Research and Treatment of Cancer quality of life questionnaire; ERV=Expiratory reserve volume; FC=functional class; FEV=Forced expiratory volume; FVC=Forced vital capacity; FU=follow up; gp(s)=group(s); I= intervention; ICU=intensive care unit; IPPB= intermittent positive pressure breathing; IRV=inspiratory reserve volume; IS=incentive spirometry; IVC=Inspiratory vital capacity; LoS = length of stay; MEP=maximal expiratory pressure; MIP=maximal inspiratory pressure; MVV=maximal voluntary ventilation; n=number; P02/Fi02= arterial oxygen partial pressure to fractional inspired oxygen ratio; PCC=post-operative pulmonary complications; PeMax=Maximum expiratory pressure; PiMax=Maximum inspiratory pressure; PFT=pulmonary function test; PO=post operative; ppts=participants; Pre-op=pre operative; sd=standard deviation; VAS=visual analogue scale; VC=vital capacity; VT=tidal volume

RESPIRATORY INTERVENTIONS

Table 14. Risk of bias

	Study	Selection bias		Performance bias	Performance bias	Detection bias						Attrition bias	Reporting bias
						Blinding of outcome assessment							
		Random sequence generation	Allocation concealment	Blinding of participants (all outcomes)	Blinding of personnel (all outcomes)	Perioperative mortality, hospital readmission	Postoperative complications	Length of stay	Patient reported outcomes (pain, HRQoL)	Intervention related outcomes	Intervention related adverse events	Incomplete outcome data	Selective reporting
**RESPIRATORY INTERVENTIONS**													
**Inspiratory muscle training (IMT)**													
1	Barbalho-Moulim 2011					**NR**					**NR**		
2	Carvalho 2011 Abstract					**NR**		**NR**	**NR**		**NR**		
3	Chen 2018; 2019					**NR**			**NR**		**NR**		
4	Da Cunha 2013 Preliminary data No ROB												
5	Dronkers 2008								**NR**		**NR**		
6	Dronkers 2010					**NR**					**NR**		
7	Ferreira 2009								**NR**				
8	Huang 2017 STUDY1										**NR**		
9	Hulzabos 2006a					**NR**			**NR**				
10	Hulzabos 2006b					**NR**			**NR**	**NR**	**NR**		
11	Jarosz 2014 Abstract					**NR**		**NR**	**NR**		**NR**		
12	Kulkarni 2010					**NR**					**NR**		

	Study	Selection bias		Performance bias	Performance bias	Detection bias						Attrition bias	Reporting bias
						Blinding of outcome assessment							
		**Random sequence generation**	**Allocation concealment**	**Blinding of participants (all outcomes)**	**Blinding of personnel (all outcomes)**	Perioperative mortality, hospital readmission	Postoperative complications	Length of stay	Patient reported outcomes (pain, HRQoL)	Intervention related outcomes	Intervention related adverse events	**Incomplete outcome data**	**Selective reporting**
**RESPIRATORY INTERVENTIONS**													
13	Laurent 2020								**NR**		**NR**		
14	Ma & Bao 2009								**NR**		**NR**		
15	Sobrihno 2014					**NR**			**NR**		**NR**		
16	Valkenet 2018												
17	Van de buis 2004 (translation)					**NR**			**NR**				
18	Weiner 1998					**NR**		**NR**	**NR**	**NR**	**NR**	**NR**	
**Inspiratory Spirometry (IS)**													
19	Bergin 2014					**NR**					**NR**		
20	Cattano 2010					**NR**				**NR**	**NR**		
21	Celli 1983 Abstract only					**NR**			**NR**	**NR**	**NR**		
see 13	Kulkarni 2010					**NR**					**NR**		
22	Leguisamo 2005							**NR**	**NR**		**NR**		
**Combined Respiratory Interventions**													
23	Benzo 2011 STUDY 1					**NR**			**NR**	**NR**			

	Study	Selection bias		Performance bias	Performance bias	Detection bias						Attrition bias	Reporting bias
						Blinding of outcome assessment							
		**Random sequence generation**	**Allocation concealment**	**Blinding of participants (all outcomes)**	**Blinding of personnel (all outcomes)**	Perioperative mortality, hospital readmission	Postoperative complications	Length of stay	Patient reported outcomes (pain, HRQoL)	Intervention related outcomes	Intervention related adverse events	**Incomplete outcome data**	**Selective reporting**
**RESPIRATORY INTERVENTIONS**													
24	Devecel 2018					**NR**			**NR**		**NR**		
25	LLorens 2015					**NR**			**NR**		**NR**		
26	Ortega 2013 Abstract only					**NR**	**NR**	**NR**	**NR**		**NR**		
27	Rajendran 1998 Abstract					**NR**			**NR**		**NR**		
28	Roviro 2012 Abstract only					**NR**	**NR**	**NR**	**NR**		**NR**		
29	Yamana 2015 Abstract only					**NR**		**NR**	**NR**	**NR**	**NR**		

NR = not reported

Perry R, et al. BMJ Open 2022; 11:e050806. doi: 10.1136/bmjopen-2021-050806

RESPIRATORY INTERVENTIONS

Table 15. Summary of findings (inspiratory muscle training)

Inspiratory muscle training (IMT) compared to usual care for any major surgery

Patient or population: any major surgery
Setting: hospital
Intervention: Inspiratory muscle training (IMT)
Comparison: usual care

Outcomes	Anticipated absolute effects* (95% CI)		Relative effect (95% CI)	№ of participants (studies)	Certainty of the evidence (GRADE)	Comments
	Risk with usual care	Risk with Inspiratory muscle training (IMT)				
Mortality follow up: 30 days	25 per 1,000	**37 per 1,000** (15 to 91)	**RR 1.49** (0.60 to 3.69)	407 (6 RCTs)	⨁⨁◯◯ LOW [a,b]	
Length of Stay (LoS)		MD **1.81 days lower** (2.31 lower to 1.32 lower)	-	1003 (10 RCTs)	⨁⨁⨁◯ MODERATE [a]	
Post-operative Pulmonary Complications (PPCs)	0 per 1,000	**0 per 1,000** (0 to 0)	**RR 0.55** (0.38 to 0.80)	633 (5 RCTs)	⨁⨁◯◯ LOW [a,c]	
Pneumonia	147 per 1,000	**101 per 1,000** (72 to 154)	**RR 0.69** (0.49 to 1.05)	1052 (11 RCTs)	⨁◯◯◯ VERY LOW [a,d,e]	

***The risk in the intervention group** (and its 95% confidence interval) is based on the assumed risk in the comparison group and the **relative effect** of the intervention (and its 95% CI).

CI: Confidence interval; **RR:** Risk ratio; **MD:** Mean difference

GRADE Working Group grades of evidence
High certainty: We are very confident that the true effect lies close to that of the estimate of the effect
Moderate certainty: We are moderately confident in the effect estimate: The true effect is likely to be close to the estimate of the effect, but there is a possibility that it is substantially different
Low certainty: Our confidence in the effect estimate is limited: The true effect may be substantially different from the estimate of the effect
Very low certainty: We have very little confidence in the effect estimate: The true effect is likely to be substantially different from the estimate of effect

Explanations

a. Downgrade for Risk of bias,
b. Downgrade for imprecision- includes null effect and appreciable benefit or harm, small sample size
c. Downgrade for imprecision- small sample
d. Downgrade Inconsistency: variation in CIs, not the same direction of effect across all studies
e. Downgrade for imprecision- includes null effect and appreciable benefit or harm, small sample size

Table 16. Summary of findings (incentive spirometry)

Incentive spirometry compared to usual care for any major surgery

Patient or population: any major surgery
Setting: hospital
Intervention: Incentive spirometry
Comparison: usual care

Outcomes	Anticipated absolute effects* (95% CI)		Relative effect (95% CI)	№ of participants (studies)	Certainty of the evidence (GRADE)	Comments
	Risk with usual care	Risk with Incentive spirometry				
Length of stay (LoS)		MD **2.39 days lower** (5.5 lower to 0.72 higher)	-	278 (3 RCTs)	⨁◯◯◯ VERY LOW [a,b,c]	
Postoperative pulmonary complications (PPCs)	344 per 1,000	**234 per 1,000** (86 to 622)	**RR 0.68** (0.25 to 1.81)	315 (4 RCTs)	⨁◯◯◯ VERY LOW [a,d,e]	

***The risk in the intervention group** (and its 95% confidence interval) is based on the assumed risk in the comparison group and the **relative effect** of the intervention (and its 95% CI).

CI: Confidence interval; **MD:** Mean difference; **RR:** Risk ratio

GRADE Working Group grades of evidence
High certainty: We are very confident that the true effect lies close to that of the estimate of the effect
Moderate certainty: We are moderately confident in the effect estimate: The true effect is likely to be close to the estimate of the effect, but there is a possibility that it is substantially different
Low certainty: Our confidence in the effect estimate is limited: The true effect may be substantially different from the estimate of the effect
Very low certainty: We have very little confidence in the effect estimate: The true effect is likely to be substantially different from the estimate of effect

Explanations

a. Downgrade for risk of bias
b. Downgrade inconsistency: variation in CIs, high heterogeneity
c. Downgrade for imprecision: includes null effect and appreciable benefit or harm, small sample size
d. Downgrade inconsistency: variation in CIs, not the same direction of effect across all studies, no overlap of CIs, high heterogeneity
e. Downgrade for imprecision: includes null effect and appreciable benefit or harm, small sample size

Meta analyses

INSPIRATORY MUSCLE TRAINING

Figure 38. Random effects meta-analysis of the risk ratio of mortality between IMT (experimental) and usual care (control).

Study or Subgroup	Experimental Events	Experimental Total	Control Events	Control Total	Weight	Risk Ratio M-H, Random, 95% CI
Laurent 2020	0	14	1	12	8.5%	0.29 [0.01, 6.50]
Huang 2017 STUDY 1	0	30	1	30	8.2%	0.33 [0.01, 7.87]
Valkenet 2018	5	120	3	121	41.3%	1.68 [0.41, 6.88]
Ma & Bao 2009	2	16	1	16	15.5%	2.00 [0.20, 19.91]
Dronkers 2010	1	10	0	8	8.7%	2.45 [0.11, 53.25]
Ferreira 2009	3	15	1	15	17.8%	3.00 [0.35, 25.68]
**Total (95% CI)**		**205**		**202**	**100.0%**	**1.49 [0.60, 3.69]**
Total events	11		7			

Heterogeneity: Tau² = 0.00; Chi² = 2.54, df = 5 (P = 0.77); I² = 0%
Test for overall effect: Z = 0.87 (P = 0.39)

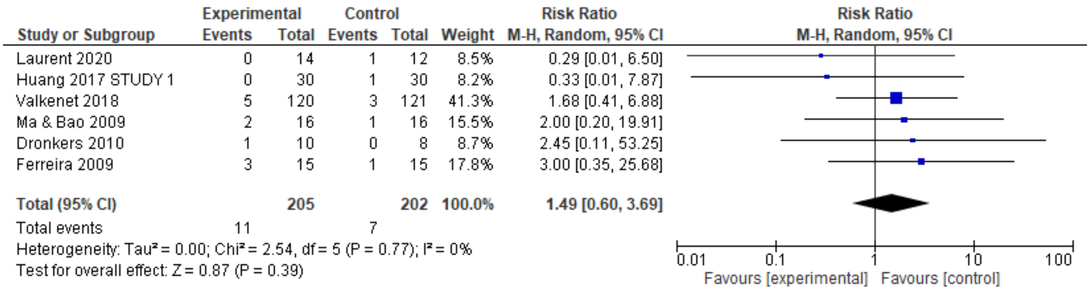

6/18 studies (407 participants).

Figure 39. Random effects meta-analysis of the mean difference in length of hospital stay (LoS) between IMT (experimental) and usual care (control).

Study or Subgroup	Experimental Mean	Experimental SD	Experimental Total	Control Mean	Control SD	Control Total	Weight	Mean Difference IV, Random, 95% CI
Barbalho-Moulim 2011	2	0	15	2.11	0.33	17		Not estimable
Chen 2018;2019	7.51	2.83	100	9.38	3.1	100	36.5%	-1.87 [-2.69, -1.05]
Dronkers 2010	16.2	11.5	20	21.6	23.7	20	0.2%	-5.40 [-16.95, 6.15]
Huang 2017 STUDY 1	15.7	3	30	17.3	4.3	30	7.0%	-1.60 [-3.48, 0.28]
Hulzabos 2006b	7.89	2.17	139	9.94	8.27	137	12.1%	-2.05 [-3.48, -0.62]
Hulzebos 2006a	7.93	1.94	14	9.92	5.78	12	2.1%	-1.99 [-5.41, 1.43]
Laurent 2020	7.6	3.3	14	8.5	4.7	12	2.5%	-0.90 [-4.07, 2.27]
Ma & Bao 2009	13.22	2.31	16	16.41	3.99	16	4.8%	-3.19 [-5.45, -0.93]
Sobrinho 2014	5.87	0.6	35	7.41	2.51	35	33.8%	-1.54 [-2.39, -0.69]
Valkenet 2018	18.4	18	120	20.5	20.9	121	1.0%	-2.10 [-7.02, 2.82]
**Total (95% CI)**			**503**			**500**	**100.0%**	**-1.81 [-2.31, -1.32]**

Heterogeneity: Tau² = 0.00; Chi² = 2.70, df = 8 (P = 0.95); I² = 0%
Test for overall effect: Z = 7.15 (P < 0.00001)

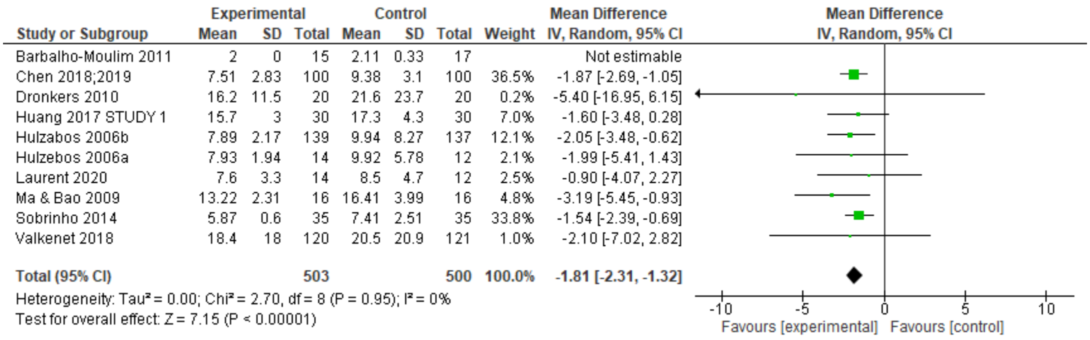

10/18 studies (1003 participants).

Figure 40. Random effects meta-analysis of the risk ratio of total PPCs between IMT (experimental) and usual care (control).

Study or Subgroup	Experimental Events	Experimental Total	Control Events	Control Total	Weight	Risk Ratio M-H, Random, 95% CI
Barbalho-Moulim 2011	0	15	0	17		Not estimable
Chen 2018;2019	10	100	27	100	24.6%	0.37 [0.19, 0.72]
Dronkers 2010	5	21	5	20	10.8%	0.95 [0.32, 2.80]
Hulzabos 2006b	25	139	48	137	48.1%	0.51 [0.34, 0.78]
Weiner 1998	8	42	9	42	16.5%	0.89 [0.38, 2.08]
**Total (95% CI)**		**317**		**316**	**100.0%**	**0.55 [0.38, 0.80]**
Total events	48		89			

Heterogeneity: Tau² = 0.03; Chi² = 3.66, df = 3 (P = 0.30); I² = 18%
Test for overall effect: Z = 3.12 (P = 0.002)

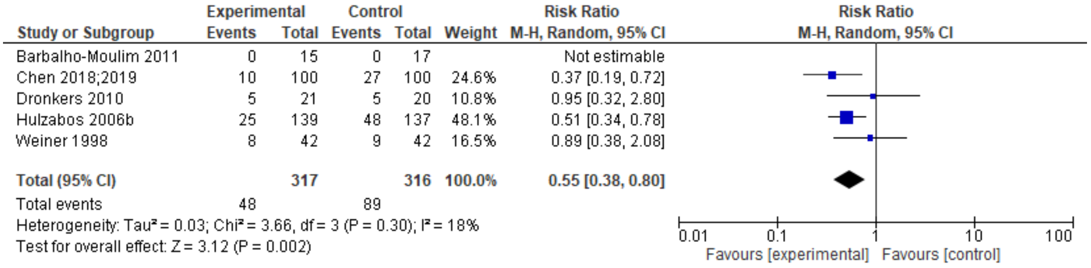

5/18 studies (633 participants)

Figure 41. Random effects meta-analysis of the risk ratio of pneumonia between IMT (experimental) and usual care (control).

Study or Subgroup	Experimental Events	Experimental Total	Control Events	Control Total	Weight	Risk Ratio M-H, Random, 95% CI
Carvalho 2011 Poster	1	16	2	16	3.0%	0.50 [0.05, 4.98]
Chen 2018;2019	3	98	7	99	8.2%	0.43 [0.12, 1.63]
Dronkers 2010	1	21	3	20	3.4%	0.32 [0.04, 2.80]
Ferreira 2009	1	15	0	15	1.7%	3.00 [0.13, 68.26]
Huang 2017 STUDY 1	5	30	7	30	12.4%	0.71 [0.25, 2.00]
Hulzabos 2006b	9	139	22	137	20.0%	0.40 [0.19, 0.84]
Hulzebos 2006a	1	14	2	12	3.1%	0.43 [0.04, 4.16]
Kulkami 2010	0	17	2	17	1.9%	0.20 [0.01, 3.88]
Ma & Bao 2009	1	15	0	16	1.7%	3.19 [0.14, 72.69]
Valkenet 2018	47	120	43	121	41.4%	1.10 [0.79, 1.53]
Weiner 1998	1	42	3	42	3.2%	0.33 [0.04, 3.08]
**Total (95% CI)**		**527**		**525**	**100.0%**	**0.69 [0.46, 1.05]**
Total events	70		91			

Heterogeneity: Tau² = 0.08; Chi² = 12.15, df = 10 (P = 0.28); I² = 18%
Test for overall effect: Z = 1.74 (P = 0.08)

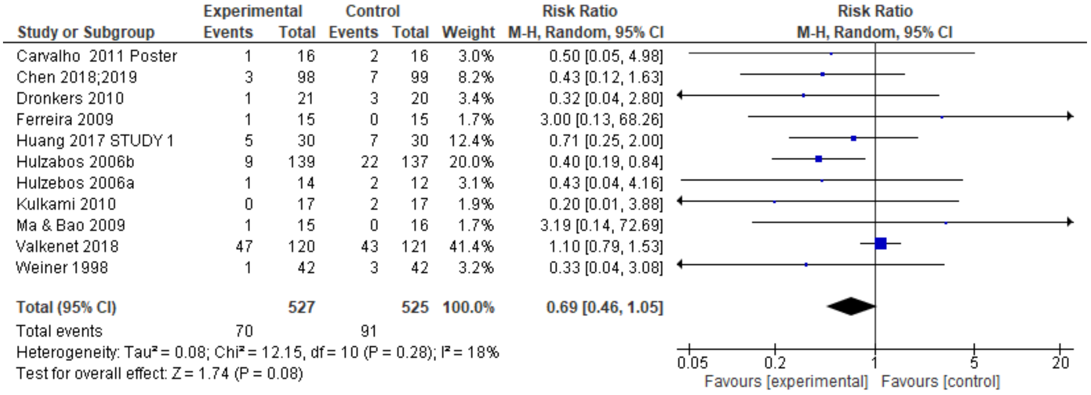

11/18 studies (1052 participants). 1 abstract poster (Carvalho et al., 2011) included.

INCENTIVE SPIROMETRY

Figure 42. Random effects meta-analysis of the mean difference in length of hospital stay (LoS) between incentive spirometry (experimental) and usual care (control).

Study or Subgroup	Experimental Mean	Experimental SD	Experimental Total	Control Mean	Control SD	Control Total	Weight	Mean Difference IV, Random, 95% CI
Bergin 2014	2.5	0.6	50	2.7	0.6	56	37.4%	-0.20 [-0.43, 0.03]
Celli 1983	8.6	3	42	13	5	44	33.5%	-4.40 [-6.13, -2.67]
Leguisamo 2005	11.77	6.26	42	14.65	6.61	44	29.1%	-2.88 [-5.60, -0.16]
**Total (95% CI)**			**134**			**144**	**100.0%**	**-2.39 [-5.50, 0.72]**

Heterogeneity: Tau² = 6.71; Chi² = 25.67, df = 2 (P < 0.00001); I² = 92%
Test for overall effect: Z = 1.51 (P = 0.13)

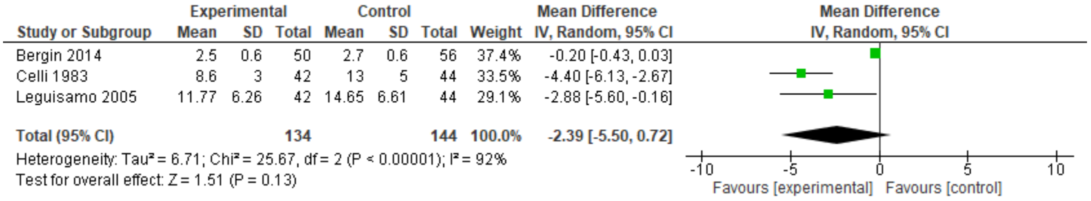

3/5 studies (278 participants).

Perry R, et al. BMJ Open 2022; 11:e050806. doi: 10.1136/bmjopen-2021-050806

Figure 43. Random effects meta-analysis of the risk ratio of PPCs between incentive spirometry (experimental) and usual care (control).

Study or Subgroup	Experimental Events	Experimental Total	Control Events	Control Total	Weight	Risk Ratio M-H, Random, 95% CI
Bergin 2014	1	50	3	56	13.8%	0.37 [0.04, 3.47]
Cattano 2010	0	18	0	19		Not estimable
Celli 1983	9	42	21	44	39.5%	0.45 [0.23, 0.87]
Leguisamo 2005	35	42	32	44	46.7%	1.15 [0.91, 1.44]
**Total (95% CI)**		**152**		**163**	**100.0%**	**0.68 [0.25, 1.81]**
Total events	45		56			

Heterogeneity: Tau² = 0.53; Chi² = 11.10, df = 2 (P = 0.004); I² = 82%
Test for overall effect: Z = 0.77 (P = 0.44)

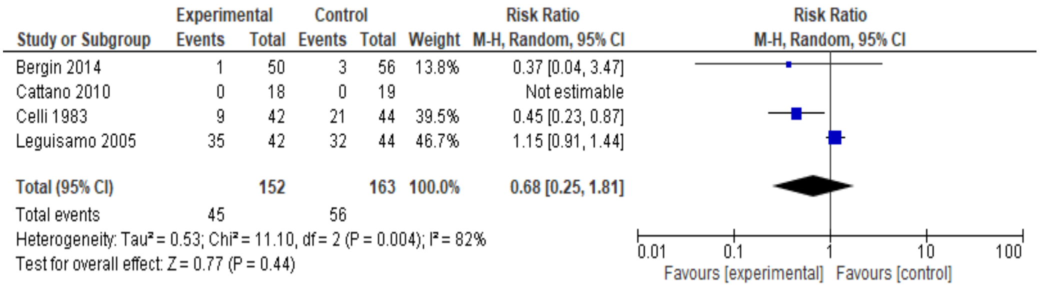

4/5 studies (315 participants).

Sensitivity analyses (removing studies at high risk of bias)

INSPIRATORY MUSCLE TRAINING

This sensitivity analysis could not be conducted as there were not enough studies available.

Sensitivity analyses (removing studies with imputed results)

INSPIRATORY MUSCLE TRAINING

Figure 44.Random effects meta-analysis of the mean difference in length of stay between IMT (experimental) and usual care (control) with imputed results removed.

	Experimental			Control				Mean Difference
Study or Subgroup	Mean	SD	Total	Mean	SD	Total	Weight	IV, Random, 95% CI
Barbalho-Moulim 2011	2	0	15	2.11	0.33	17		Not estimable
Chen 2018;2019	7.51	2.83	98	9.38	3.1	100	70.2%	-1.87 [-2.70, -1.04]
Dronkers 2010	16.2	11.5	20	21.6	23.7	20	0.4%	-5.40 [-16.95, 6.15]
Hulzabos 2006b	7.89	2.17	139	9.94	8.27	137	23.4%	-2.05 [-3.48, -0.62]
Hulzebos 2006a	7.93	1.94	14	9.92	5.78	12	4.1%	-1.99 [-5.41, 1.43]
Valkenet 2018	18.4	18	120	20.5	20.9	121	2.0%	-2.10 [-7.02, 2.82]
**Total (95% CI)**			**406**			**407**	**100.0%**	**-1.93 [-2.63, -1.24]**

Heterogeneity: Tau² = 0.00; Chi² = 0.40, df = 4 (P = 0.98); I² = 0%
Test for overall effect: Z = 5.48 (P < 0.00001)

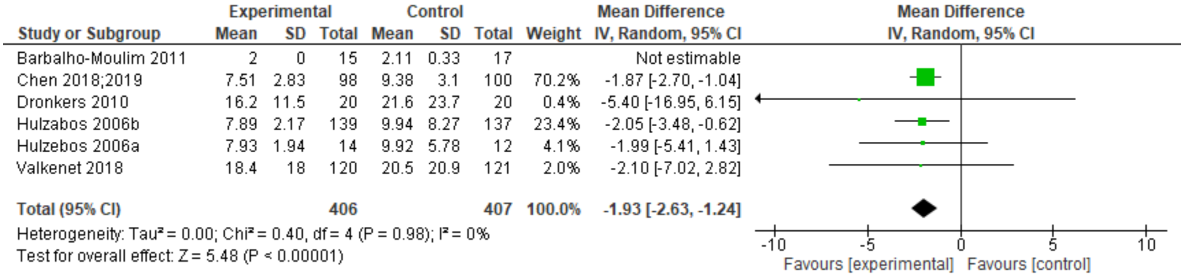

6 studies (815 participants) included and 3 studies excluded (Ma and Bao 2009, Sobrinho et al., 2014; Barbalho-Moulim et al., 2011).

Subgroup analyses (type of surgery)

INSPIRATORY MUSCLE TRAINING (CARDIAC SURGERY)

Figure 45. Random effects meta-analysis of the mean difference in length of hospital stay (LoS) between IMT (experimental) and usual care (control) for those undergoing cardiac surgery.

	Experimental			Control				Mean Difference
Study or Subgroup	Mean	SD	Total	Mean	SD	Total	Weight	IV, Random, 95% CI
Chen 2018	7.5	2.8	100	9.4	8.1	100	15.4%	-1.90 [-3.58, -0.22]
Dronkers 2010	16.2	11.5	20	21.6	23.7	20	0.3%	-5.40 [-16.95, 6.15]
Hulzabos 2006b	7.89	2.17	139	9.94	8.27	137	21.2%	-2.05 [-3.48, -0.62]
Hulzebos 2006a	7.93	1.94	14	9.92	5.78	12	3.7%	-1.99 [-5.41, 1.43]
Sobrinho 2014	5.87	0.6	35	7.41	2.51	35	59.4%	-1.54 [-2.39, -0.69]
**Total (95% CI)**			**308**			**304**	**100.0%**	**-1.73 [-2.39, -1.07]**

Heterogeneity: Tau² = 0.00; Chi² = 0.83, df = 4 (P = 0.93); I² = 0%
Test for overall effect: Z = 5.15 (P < 0.00001)

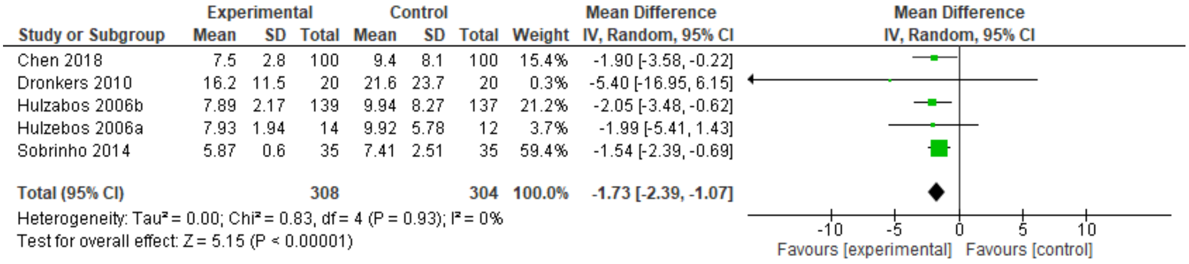

5 studies (612 participants) included and 5 studies excluded (Barbalho-Moulim et al., 2011, Huang et al., 2017, Laurent et al., 2002, Ma & Bao 2009, Valkenet et al., 2018).

Perry R, *et al. BMJ Open* 2022; 11:e050806. doi: 10.1136/bmjopen-2021-050806

Figure 46. Random effects meta-analysis of the risk ratio of pneumonia between IMT (experimental) and usual care (control) for those undergoing cardiac surgery.

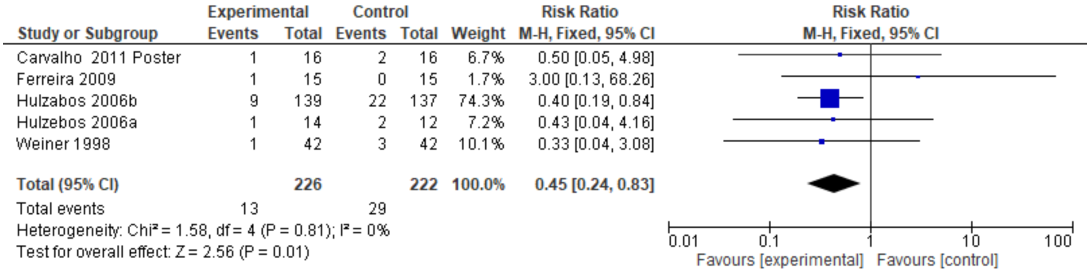

5 studies (448 participants) included and 6 studies excluded (Chen et al., 2019, Dronkers et al., 2010, Huang et al., 2017, Kulkami et al., 2010, Ma & Bao 2009, Valkenet et al., 2018). 1 abstract poster (Carvalho et al., 2011) included.

Figure 47. Random effects meta-analysis of the risk ratio of post-operative pulmonary complications between IMT (experimental) and usual care (control) for those undergoing cardiac surgery.

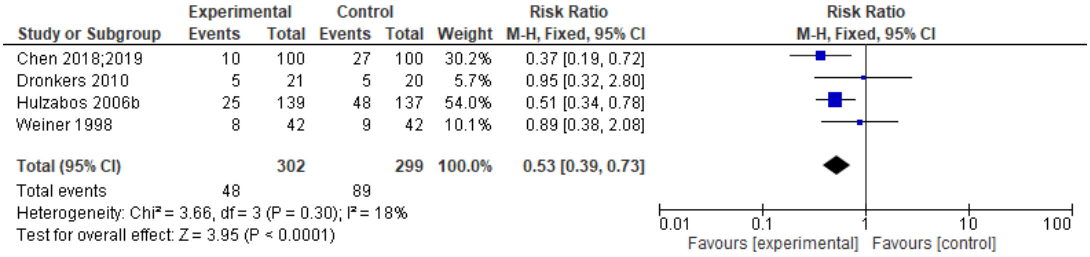

4 studies (601 participants) included and 1 studies excluded (Barbalho-Moulim et al., 2011).

Perry R, *et al. BMJ Open* 2022; 11:e050806. doi: 10.1136/bmjopen-2021-050806

Subgroup analysis (studies published before and after 2010)

This subgroup analysis could not be performed for incentive spirometry as not enough studies were available.

INSPIRATORY MUSCLE TRAINING

Figure 48. Random effects meta-analysis of the risk ratio of mortality between inspiratory muscle training (experimental) and usual care (control) in studies published after 2010.

Study or Subgroup	Experimental Events	Experimental Total	Control Events	Control Total	Weight	Risk Ratio M-H, Random, 95% CI
Dronkers 2010	1	10	0	8	13.0%	2.45 [0.11, 53.25]
Huang 2017 STUDY 1	0	30	1	30	12.3%	0.33 [0.01, 7.87]
Laurent 2020	0	14	1	12	12.7%	0.29 [0.01, 6.50]
Valkenet 2018	5	120	3	121	62.0%	1.68 [0.41, 6.88]
**Total (95% CI)**		**174**		**171**	**100.0%**	**1.16 [0.38, 3.51]**
Total events	6		5			

Heterogeneity: Tau² = 0.00; Chi² = 1.86, df = 3 (P = 0.60); I² = 0%
Test for overall effect: Z = 0.26 (P = 0.80)

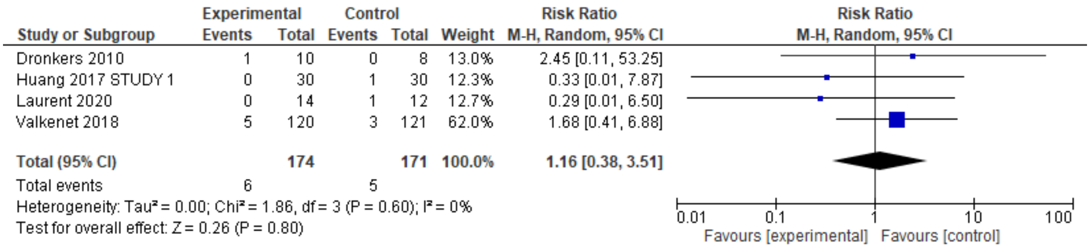

4 studies (345 participants) included and 2 studies were excluded (Ma and Bao 2009 and Ferreira et al., 2009).

Figure 49. Random effects meta-analysis of the mean difference in length of hospital stay (LoS) between inspiratory muscle training (experimental) and usual care (control) in studies published after 2010.

Study or Subgroup	Experimental Mean	Experimental SD	Experimental Total	Control Mean	Control SD	Control Total	Weight	Mean Difference IV, Random, 95% CI
Barbalho-Moulim 2011	2	0	15	2.11	0.33	17		Not estimable
Chen 2018;2019	7.51	2.83	100	9.38	3.1	100	45.1%	-1.87 [-2.69, -1.05]
Dronkers 2010	16.2	11.5	20	21.6	23.7	20	0.2%	-5.40 [-16.95, 6.15]
Huang 2017 STUDY 1	15.7	3	30	17.3	4.3	30	8.7%	-1.60 [-3.48, 0.28]
Laurent 2020	7.6	3.3	14	8.5	4.7	12	3.0%	-0.90 [-4.07, 2.27]
Sobrinho 2014	5.87	0.6	35	7.41	2.51	35	41.7%	-1.54 [-2.39, -0.69]
Valkenet 2018	18.4	18	120	20.5	20.9	121	1.3%	-2.10 [-7.02, 2.82]
**Total (95% CI)**			**334**			**335**	**100.0%**	**-1.69 [-2.24, -1.14]**

Heterogeneity: Tau² = 0.00; Chi² = 0.97, df = 5 (P = 0.96); I² = 0%
Test for overall effect: Z = 6.00 (P < 0.00001)

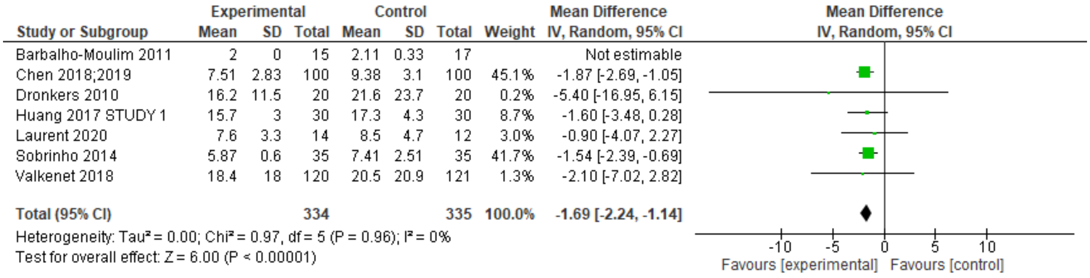

7 studies (669 participants) included and 3 studies excluded (Hulzabos et al., 2006a and b, Ma & Bao, 2009).

Figure 50. Random effects meta-analysis of the mean difference in length of hospital stay (LoS) between inspiratory muscle training (experimental) and usual care (control) in studies published before 2010.

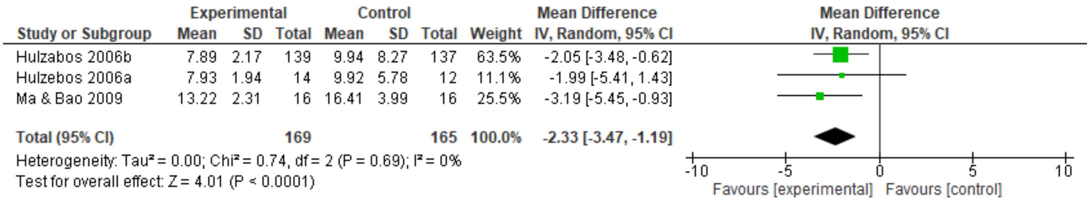

Study or Subgroup	Experimental Mean	Experimental SD	Experimental Total	Control Mean	Control SD	Control Total	Weight	Mean Difference IV, Random, 95% CI
Hulzabos 2006b	7.89	2.17	139	9.94	8.27	137	63.5%	-2.05 [-3.48, -0.62]
Hulzebos 2006a	7.93	1.94	14	9.92	5.78	12	11.1%	-1.99 [-5.41, 1.43]
Ma & Bao 2009	13.22	2.31	16	16.41	3.99	16	25.5%	-3.19 [-5.45, -0.93]
**Total (95% CI)**			**169**			**165**	**100.0%**	**-2.33 [-3.47, -1.19]**

Heterogeneity: Tau² = 0.00; Chi² = 0.74, df = 2 (P = 0.69); I² = 0%
Test for overall effect: Z = 4.01 (P < 0.0001)

3 studies (334 participants) included and 7 studies excluded (Barbalho-Moulim et al., 2011; Chen 2019; Dronkers et al., 2010; Huang et al., 2017; Laurent et al., 2020; Sobrinho et al., 2014; Valkenet et al., 2018).

Figure 51. Random effects meta-analysis of the risk ratio of postoperative pulmonary complications (PPC) between inspiratory muscle training (experimental) and usual care (control) in studies published after 2010.

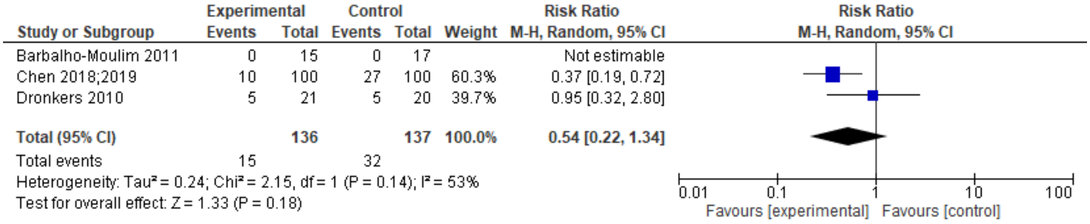

Study or Subgroup	Experimental Events	Experimental Total	Control Events	Control Total	Weight	Risk Ratio M-H, Random, 95% CI
Barbalho-Moulim 2011	0	15	0	17		Not estimable
Chen 2018;2019	10	100	27	100	60.3%	0.37 [0.19, 0.72]
Dronkers 2010	5	21	5	20	39.7%	0.95 [0.32, 2.80]
**Total (95% CI)**		**136**		**137**	**100.0%**	**0.54 [0.22, 1.34]**
Total events	15		32			

Heterogeneity: Tau² = 0.24; Chi² = 2.15, df = 1 (P = 0.14); I² = 53%
Test for overall effect: Z = 1.33 (P = 0.18)

3 studies (273 participants) included and 3 studies exlcuded (Hulzabos et al., 2006b, Ma & Bao, 2009; Weiner et al., 1998).

Figure 52.Random effects meta-analysis of the risk ratio of penumonia between inspiratory muscle training (experimental) and usual care (control) in studies published after 2010.

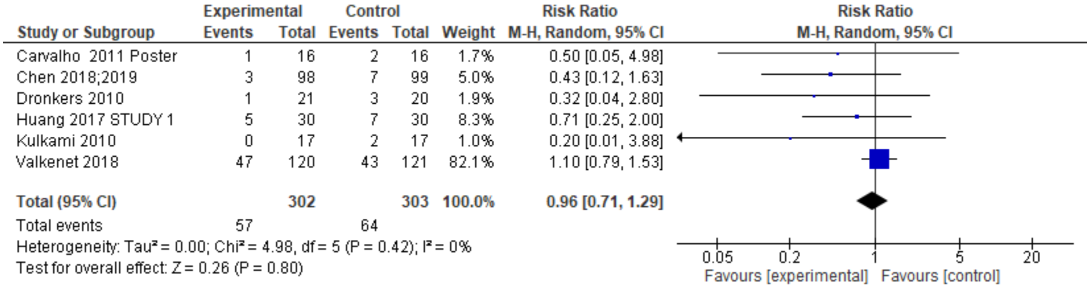

Study or Subgroup	Experimental Events	Experimental Total	Control Events	Control Total	Weight	Risk Ratio M-H, Random, 95% CI
Carvalho 2011 Poster	1	16	2	16	1.7%	0.50 [0.05, 4.98]
Chen 2018;2019	3	98	7	99	5.0%	0.43 [0.12, 1.63]
Dronkers 2010	1	21	3	20	1.9%	0.32 [0.04, 2.80]
Huang 2017 STUDY 1	5	30	7	30	8.3%	0.71 [0.25, 2.00]
Kulkami 2010	0	17	2	17	1.0%	0.20 [0.01, 3.88]
Valkenet 2018	47	120	43	121	82.1%	1.10 [0.79, 1.53]
**Total (95% CI)**		**302**		**303**	**100.0%**	**0.96 [0.71, 1.29]**
Total events	57		64			

Heterogeneity: Tau² = 0.00; Chi² = 4.98, df = 5 (P = 0.42); I² = 0%
Test for overall effect: Z = 0.26 (P = 0.80)

6 studies (605 participants) included and 5 studies excluded (Ferreira et al., 2009; Hulzebos et al., 2006a and b; Ma & Bao, 2009; Weiner et al., 1998).

Figure 53. Random effects meta-analysis of the risk ratio of penumonia between inspiratory muscle training (experimental) and usual care (control) in studies published before 2010.

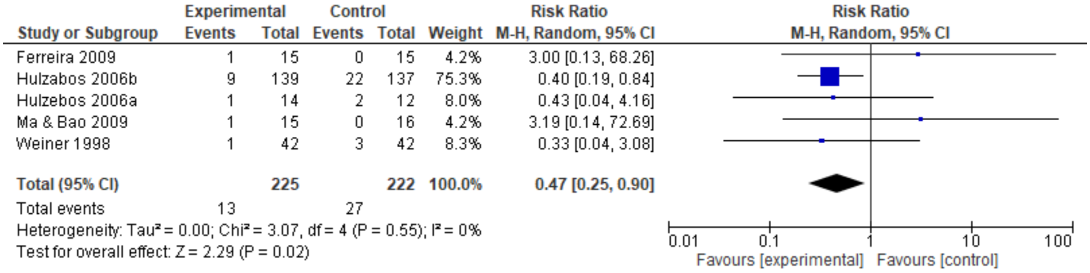

5 studies (447 participants) included and 6 studies excluded (Carvalho et al., 2011; Chen etal., 2019; Dronkers et al., 2010; Huang et al., 2017; Kulkami et al., 2010 Valkenet et al., 2018).

References

Barbalho-Moulim, M. C., Miguel, G. P., Forti, E. M., Campos Fdo, A. & Costa, D. 2011. Effects of preoperative inspiratory muscle training in obese women undergoing open bariatric surgery: respiratory muscle strength, lung volumes, and diaphragmatic excursion. *Clinics (Sao Paulo),* 66**,** 1721-7.

Benzo, R., Wigle, D., Novotny, P., Wetzstein, M., Nichols, F., Shen, R. K., . . . Deschamps, C. 2011. Preoperative pulmonary rehabilitation before lung cancer resection: results from two randomized studies. *Lung Cancer,* 74**,** 441-5.

Bergin, C., Speroni, K. G., Travis, T., Bergin, J., Sheridan, M. J., Kelly, K. & Daniel, M. G. 2014. Effect of preoperative incentive spirometry patient education on patient outcomes in the knee and hip joint replacement population. *J Perianesth Nurs,* 29**,** 20-7.

Carvalho, T., Bonorino, K. C. & Panigas, T. F. 2011. Preoperative respiratory muscle training reduces complications in coronary artery bypass surgery [abstract]. *Eur Heart J,* 32.

Cattano, D., Altamirano, A., Vannucci, A., Melnikov, V., Cone, C. & Hagberg, C. A. 2010. Preoperative use of incentive spirometry does not affect postoperative lung function in bariatric surgery. *Translational Research: The Journal Of Laboratory & Clinical Medicine,* 156**,** 265-72.

Celli, B. R., Rodriguez, K. S. & Snider, G. L. 1984. A controlled trial of intermittent positive pressure breathing, incentive spirometry, and deep breathing exercises in preventing pulmonary complications after abdominal surgery. *Am Rev Respir Dis,* 130**,** 12-5.

Chen, X. & Guo, Q. 2018. Short-term intensive preoperative inspiratory muscle training to prevent postoperative pulmonary complications in patients undergoing cardiac surgery: A randomized controlled trial. *Journal of the American College of Cardiology,* 72**,** C240.

Chen, X., Hou, L., Zhang, Y., Liu, X., Shao, B., Yuan, B., . . . Guo, Q. 2019. The effects of five days of intensive preoperative inspiratory muscle training on postoperative complications and outcome in patients having cardiac surgery: a randomized controlled trial. *Clinical Rehabilitation,* 33**,** 913-922.

Da Cunha, F., Ruas, G., Fanan, J., Crema, E. & Volpe, M. 2013. Effects of preoperative respiratory muscle training on early and late postoperative outcome of patients undergoing esophageal surgery. *Intensive Care Medicine* 39**,** S369.

Devecel, G. & Senturan, L. 2018. The Effect of Breathing Exercises on Preventing Postoperative Atelectasis: A Randomized Controlled Trial. *Florence Nightingale Journal of Nursing-Florence Nightingale Hemsirelik Dergisi,* 26**,** 159-167.

Dronkers, J., Veldman, A., Hoberg, E., Van Der Waal, C. & Van Meeteren, N. 2008. Prevention of pulmonary complications after upper abdominal surgery by preoperative intensive inspiratory muscle training: a randomized controlled pilot study. *Clin Rehabil,* 22**,** 134-42.

Dronkers, J. J., Lamberts, H., Reutelingsperger, I. M., Naber, R. H., Dronkers-Landman, C. M., Veldman, A. & Van Meeteren, N. L. 2010. Preoperative therapeutic programme for elderly patients scheduled for elective abdominal oncological surgery: a randomized controlled pilot study. *Clin Rehabil,* 24**,** 614-22.

Ferreira, P. E., Rodrigues, A. J. & Evora, P. R. 2009. Effects of an inspiratory muscle rehabilitation program in the postoperative period of cardiac surgery. *Arq Bras Cardiol,* 92**,** 275-82.

Huang, J., Lai, Y., Zhou, X., Li, S., Su, J., Yang, M. & Che, G. 2017. Short-term high-intensity rehabilitation in radically treated lung cancer: a three-armed randomized controlled trial. *J Thorac Dis,* 9**,** 1919-1929.

Hulzebos, E. H., Helders, P. J., Favié, N. J., De Bie, R. A., Brutel De La Riviere, A. & Van Meeteren, N. L. 2006a. Preoperative intensive inspiratory muscle training to prevent postoperative pulmonary complications in high-risk patients undergoing CABG surgery: a randomized clinical trial. *Jama,* 296**,** 1851-7.

Hulzebos, E. H., Van Meeteren, N. L., Van Den Buijs, B. J., De Bie, R. A., Brutel De La Rivière, A. & Helders, P. J. 2006b. Feasibility of preoperative inspiratory muscle training in patients undergoing coronary artery bypass surgery with a high risk of postoperative pulmonary complications: a randomized controlled pilot study. *Clin Rehabil,* 20**,** 949-59.

Jarosz, A., Szlubowski, A., Grochowski, Z., Janczura, M., Ładyńska, M., Pominkiewicz, Ł., . . . Kużdżał, J. 2014. P-120 The Evaluation of utility of preoperative systematized pulmonological physiotherapy amongh non-small-cell lung cancer patients undergoing anatomical lung resection. *Interactive CardioVascular and Thoracic Surgery,* 18**,** S32-S32.

Kulkarni, S. R., Fletcher, E., Mcconnell, A. K., Poskitt, K. R. & Whyman, M. R. 2010. Pre-operative inspiratory muscle training preserves postoperative inspiratory muscle strength following major abdominal surgery - a randomised pilot study. *Ann R Coll Surg Engl,* 92**,** 700-7.

Laurent, H., Aubreton, S., Galvaing, G., Pereira, B., Merle, P., Richard, R., . . . Filaire, M. 2020. Preoperative respiratory muscle endurance training improves ventilatory capacity and prevents pulmonary postoperative complications after lung surgery. *European journal of physical and rehabilitation medicine,* 56**,** 73-81.

Leguisamo, C. P., Kalil, R. a. K. & Furlani, A. P. 2005. A efetividade de uma proposta fisioterapêutica pré-operatória para cirurgia de revascularização do miocárdio. *Brazilian Journal of Cardiovascular Surgery,* 20**,** 134-141.

Lloréns, J., Rovira, L., Ballester, M., Moreno, J., Hernández-Laforet, J., Santonja, F. J., . . . Ortega, J. 2015. Preoperative inspiratory muscular training to prevent postoperative hypoxemia in morbidly obese patients undergoing laparoscopic bariatric surgery. A randomized clinical trial. *Obes Surg,* 25**,** 1003-9.

Ma, B. & Bao, H. 2009. Reduction in pulmonary complications in high risk patients undergoing surgery for total hip replacement under general anesthesia by preoperative intensive inspiratory muscle training : A randomized controlled clinical trial. *Journal of Nanjing Medical University,* 23**,** 328-334.

Moreno, J., Rovira, L., Hernandez, J., Ballester, M., Belda, J. & Llorens, J. 2012. Effect of preoperatory chest physiotherapy program on the oxigenation, after laparoscopic bariatric surgery: 5AP3-8. *European Journal of Anaesthesiology (EJA),* 29**,** 88.

Ortega, J., Cassinello, N., Rovira, L., Mayte, B. & Julio, L. 2013. *Preoperative Respiratory Physiotherapy Can Improve Oxygenation During Bariatric Surgery*.

Rajendran, A. J., Pandurangi, U. M., Murali, R., Gomathi, S., Vijayan, V. K. & Cherian, K. M. 1998. Pre-operative short-term pulmonary rehabilitation for patients of chronic obstructive pulmonary disease undergoing coronary artery bypass graft surgery. *Indian Heart J,* 50**,** 531-4.

Rovira Soriano, L., Moreno Pachón, J., Hernández Laforet, J., Ballester Luján, M., Belda Nácher, F. J. & Lloréns Herrerias, J. 2012. Effects of respiratory preoperative physiotherapy on the intraoperative respiratory mechanics and oxygenation in patients undergoing laparoscopic bariatric surgery: 5AP3-9. *European Journal of Anaesthesiology | EJA,* 29**,** 88.

Sobrinho, M. T., Guirado, G. N. & Silva, M. a. D. M. 2014. Preoperative therapy restores ventilatory parameters and reduces length of stay in patients undergoing myocardial revascularization. *Revista brasileira de cirurgia cardiovascular : orgao oficial da Sociedade Brasileira de Cirurgia Cardiovascular,* 29**,** 221-228.

Valkenet, K., Trappenburg, J. C. A., Ruurda, J. P., Guinan, E. M., Reynolds, J. V., Nafteux, P., . . . Backx, F. J. G. 2018. Multicentre randomized clinical trial of inspiratory muscle training versus usual care before surgery for oesophageal cancer. *British Journal of Survery,* 105**,** 502-511.

Van Den Buijs, B. J. W., Hulzebos, H. J., Bie, R., Riviere, A. B., Helders, P. J. M. & Meeteren, N. L. U. 2004. Preoperative inspiratory muscle training in patients due to undergo open-heart surgery: a pilot study. *Dutch J Phys Ther,* 114**,** 104-109.

Weiner, P., Zeidan, F., Zamir, D., Pelled, B., Waizman, J., Beckerman, M. & Weiner, M. 1998. Prophylactic inspiratory muscle training in patients undergoing coronary artery bypass graft. *World J Surg,* 22**,** 427-31.

Yamana, I., Takeno, S., Hashimoto, T., Maki, K., Shibata, R., Shiwaku, H., . . . Yamashita, Y. 2015. Randomized Controlled Study to Evaluate the Efficacy of a Preoperative Respiratory Rehabilitation Program to Prevent Postoperative Pulmonary Complications after Esophagectomy. *Dig Surg,* 32**,** 331-7.

MULTIMODAL INTERVENTIONS

Table 17. Characteristics of studies

ID	1st Author, year and country	Total *n* Intervention (I) Control (C)<br><br>Number analysed (An if reported)	Patient population, baseline clinical characteristics (mean (SD) or *n (%)* unless otherwise stated	Demographics (mean (SD) or *n* unless otherwise stated)	Intervention	Comparator	Mode of delivery; place of delivery; training level of individuals who delivered the intervention; the number of contacts	Intervention fidelity; Compliance or adherence to intervention
**MULTIMODAL INTERVENTIONS**								
1	Abdelaal et al., 2017<br>Egypt	50 ppts undergoing elective upper abdominal surgery (laparoscopic biliary surgery, hiatus hernia repair, gastrectomy, splenectomy)<br><br>I=26:C=24	No past surgery in upper abdomen; BMI>30; 'in good general condition'<br>**ASA classification I/2/3**<br>I=17/6/3<br>C=16/5/3	**Age yrs [median (range)]**<br>I=55.5 (49-67)<br>C=52 (47-56)<br>**Gender (M%)**<br>I=86:C=71<br>**Smoker (%)**<br>I=23:C=16	Pre-operative physical and respiratory therapy for 2 wks before surgery.<br>2x 40 min physical therapy sessions/wk (stretching, upper/lower extremity, walking and relaxation).<br>Respiratory: Instructions to inflate a balloon for 15mins and diaphragmatic breathing 2x/day 4x/wk<br>Walking: Instructions to walk faster than normal for 10mins on flat ground 2x/day 4x/wk<br><br>Both arms received a PO physical therapy program	No pre-op physical or respiratory therapy	Unclear if physical therapy sessions were 1:1 or group based;<br>Clinic (physical therapy) plus home-based;<br>Physiotherapist;<br>4 sessions	NR;<br>NR
2	Allen et al., 2019<br>NR<br>Abstract only	53 ppts undergoing resection for oesophagogastric (OG) cancer<br>I=25 (22An)<br>C=28(23An)	NR	NR	15-wk multi-modal prehabilitation programme<br>Prehab comprised 2x/wk supervised and 3x/wk home exercise sessions, medical coaching, and tailored dietetic input.	Usual care	Face-to-face; written;<br>Centre & Home-based;<br>NR;<br>30 sessions supervised	NR;<br>% compliance was I=76:C=65

ID	1st Author, year and country	Total *n* Intervention (I) Control (C)<br><br>Number analysed (An if reported)	Patient population, baseline clinical characteristics (mean (SD) or *n (%)* unless otherwise stated	Demographics (mean (SD) or *n* unless otherwise stated)	Intervention	Comparator	Mode of delivery; place of delivery; training level of individuals who delivered the intervention; the number of contacts	Intervention fidelity; Compliance or adherence to intervention
**MULTIMODAL INTERVENTIONS**								
3	Ausania et al., 2019<br>Spain	40 pancreaticoduodenectomy ppts with pancreatic or periampullary tumors<br>I=18:C=22	**ASA 2/3**<br>I=7/11:C=9/13<br>**BMI kg/m²) (median)**<br>I=24.8:C=26.5	**Age yrs [median (range)]**<br>I=66.1 (38-80)<br>C=65.7 (38-81)<br>**Gender (M%)**<br>I=59:C=50	Personalized prehab program:<br>Nutrition support liquid oral nutrition supplements + vitamins<br>Control of diabetes and exocrine pancreatic insufficiency<br>Physical and respiratory training<br>5 sessions: 1 hr each/day Then home training.<br>Median duration of prehab 12.6 days	Usual care	Oral, written;<br>NR but "supervised"+ home;<br>NR;<br>5 supervised sessions	NR;<br>NR
4	Arthur et al., 2000<br>Canada	Cardiovascular surgery CABG<br>N=249 randomised (3 withdrew)<br>I=123:C=123		**Age**<br>I=61.8 (8.4)<br>C=63.8 (7.8)<br>**Gender (M%)**<br>I=88:C=83<br>**Smoker %**<br>I=20.3:C=13	Multi modal intervention: exercise education and reinforcement<br>Exercise=2x/wk for 8 wks<br>Education BL and 1 wk before surgery	Usual care	Face to face;<br>Supervised hospital environment;<br>Kinesiologists and exercise specialists;<br>NR	NR;<br>I=mean 14 sessions (1-57) over 8.3 wks
5	Barberan-Garcia et al., 2018<br>Spain	144 ppts undergoing elective major abdominal surgery<br><br>I=73 (62 An)<br>C=71 (63 An)	**ASA index 2/3/4**<br>I=30/68/2%<br>C=38/56/6%<br>**Charlson index**<br>I=7(9):C=7(8)<br>**BMI (kg/m²)**<br>I=21(7):C=22(7)	**Age yrs**<br>I=71 (11)<br>C=71 (10)<br>**Gender (% M)**<br>I=68:C=80<br>**Current smoker %**<br>I=32:C=39	Personalized prehabilitation Program: i) motivational interview to assess ppt's adherence profile and to codesign the characteristics of the physical activity program with the ppt; ii) personalized, unsupervised home program to promote daily physical activity; and iii) supervised high-intensity endurance exercise training program.<br>At least 4 wks (mean duration 6 (2) wks)	Usual care	Face-to-face;<br>Home and outpatient's clinic;<br>Delivered by physiotherapist;<br>1-3 supervised sessions/wk, mean no. of sessions=12 (5)	NR:<br>NR
6	Benzo et al., 2011<br>STUDY 2	19 undergoing lung cancer resection<br>I=10:C=9	**Current Smoker**<br>I=1 (10%)<br>C=2 (22%)<br>**Comorbidities**	**Age** yrs (N=19)<br>I=70.2 (8.61)<br>C=72.0 (6.69)<br>**Gender (%M)**<br>I=50:C=44	Preoperative pulmonary rehabilitation training,<br>Lower extremity (LE) endurance training; Strengthening exercises with Thera-band; Inspiratory muscle training (IMT);	Usual care	Face to face;<br>Hospital?<br>Physiotherapist;<br>NR	NR;<br>All 10 pps successfully completed 10 face to face sessions

ID	1st Author, year and country	Total *n* Intervention (I) Control (C)<br><br>Number analysed (An if reported)	Patient population, baseline clinical characteristics (mean (SD) or *n* *(%)* unless otherwise stated	Demographics (mean (SD) or *n* unless otherwise stated)	Intervention	Comparator	Mode of delivery; place of delivery; training level of individuals who delivered the intervention; the number of contacts	Intervention fidelity; Compliance or adherence to intervention
**MULTIMODAL INTERVENTIONS**								
			**Coronary artery disease**<br>I =3 (30%):<br>C =1 (11%)<br>**Heart failure**<br>I =0%:C 0%<br>**Diabetes M**<br>I=3 (30%)<br>C= 3 (33%)		The Practice of slow breathing (prolonging expiratory time and thereby decreasing respiratory rate to less that 10 breaths per minute)			
7	Beaupre et al., 2004<br>Canada	131 ppts with diagnosis of non-inflammatory arthritis booked for a primary TKA (115 underwent surgery).<br><br>I=66 (55)<br>C=65 (60)	**Diagnosis of osteoarthritis** n (%)<br>I=63(96):C=64(97)<br>**> 1 joint with osteoarthritis**<br>I=33(51):C=38(58)<br>**No. of comorbid condition**<br>I=28(70):C=22(55)	**Age yrs**<br>I=67(7):C=67(6)<br>**Gender (%M)**<br>I=40:C=50	**Exercise program**: simple exercises similar to postsurgical exercise routine.<br>3x/ wk for 4 wks for a total of 12 treatment sessions.<br>**+ Education program:**<br>(1) crutch walking on level ground & stairs,<br>(2) bed mobility & transfers,<br>3) Post-operative range of motion for routine PO care.	Continued with regular activities until surgery	Verbal, face to face;<br>Community PT physical therapy clinic;<br>NR;<br>None	NR:<br>All but one ppt completed the 12 treatment sessions prior to surgery, based upon review of the logbooks.
8	Bousquet-Dion et al., 2018<br>Canada	80 ppts with colorectal cancer undergoing surgery<br><br>I=41:C=39	**Diabetes %**<br>I=27:C=15<br>**COPD**<br>I=5:C=15<br>**Coronary artery disease**<br>I=5:C=15<br>**Peripheral vascular disease**<br>I=5:C=4<br>**Atrial fibrillation**<br>I=11:C=4	**Age yrs (median, IQR)**<br>I=74(67.5-78)<br>C=71(54.5-74.5)<br>**Gender (%M)**<br>I=81:C=62	Multimodal: i) home-based exercise program, consisting of 30mins moderate to vigorous physical activity + 8 resistance training exercises both performed 3-4 days/wk; 1x/wk supervised session 30mins aerobic exercise + resistance exercises for 25mins;<br>ii) nutritional assessment, counselling and supplementation (if necessary);<br>iii) 60 min session with psychologist who provided	REHAB: identical to PREHAB programme for 8 wks PO	Face to face, written;<br>Home and hospital exercise lab;<br>Kinesiologist (and dietitian and psychologist);<br>Up to 4 supervised sessions	NR;<br>Compliance (defined as attendance to the preoperative supervised exercise sessions and recorded) was 98%.

ID	1st Author, year and country	Total *n* Intervention (I) Control (C)<br><br>Number analysed (An if reported)	Patient population, baseline clinical characteristics (mean (SD) or *n (%)* unless otherwise stated	Demographics (mean (SD) or *n* unless otherwise stated)	Intervention	Comparator	Mode of delivery; place of delivery; training level of individuals who delivered the intervention; the number of contacts	Intervention fidelity; Compliance or adherence to intervention
**MULTIMODAL INTERVENTIONS**								
					personalized techniques to manage anxiety and depressive symptoms + CD with instructions to perform techniques at home.			
9	Crowe and Henderson, 2003<br>Canada	133 ppts undergoing knee or hip arthroplasty<br>I=65:C=68	Ppts with limited physical function and limited social support and/or co-morbidities	**Age yrs**<br>I=66.9(11.9)<br>C=70.7(10.7)<br>**Gender (%M)**<br>I=28:C=24	Individually tailored comprising education package (video, booklet, information on what happens during hospital stay, respite care and diet); optional tour of hospital unit; individualized counselling sessions; optional physical activity programme to improve strength and endurance; telephone support (all in addition to usual care).<br><br>Varied depending on individual needs (administered between 1 and 24 wks prior to surgery, most common time 6 wks)	Usual care. 1 standard preoperative clinic visit (lasting about 7 hrs) approx. 1 to 2 wks prior to surgery	Verbal, written, audiovisual;<br>Hospital and home;<br>Occupational therapist, physiotherapist or nurse;<br>Depending on package (minimum.1, maximum. 2-3 sessions wkly)	NR:<br>NR
10	Demark-Wahnefried et al., 2016<br>USA	40 overweight or obese undergoing prostatectomy<br>I=20 (16An):<br>C=20 (18An)	NR for individual treatment groups<br>**Overall BMI kg/m^2**<br>31.6(4.4)	NR for individual treatment groups<br>**Overall Age yrs**<br>59.9(6.5)<br>**Ethnicity (%)**<br>African-American=30<br>White=70	Advice to:<br>reduce calorie intake by 500-1000 kcal/day 2x/wk contact<br>+<br>Daily activity plan to increase energy expenditure to achieve an additional 250 to 500 kcal/day deficit, 2xwk contact	Wait-list control arm: assigned to receive the intervention post-surgery<br><br>NB – both groups had counseling session to correct nutrient deficiencies and improve overall dietary composition	Diet counseling provided face-to-face, telephone or e-mail;<br>Exercise at University (UAB) and at home;<br>Dietitian and exercise physiologist, 2x/wk	Diet: log-book for counting calories and mins of exercise. Ppts were instructed to weigh themselves daily. In each counseling session, dietary intakes were reviewed, reinforcement provided, and challenges of the upcoming week discussed.<br>Exercise: Heartrate monitors were used to assess adherence. Adherence reported as 95% (not stated how this was calculated)
11	Huang et al., 2012	243 ppts	Advanced osteoarthritis	**Overall Age yrs**<br>70.2 (7.3)	Education program started 2-4 wks pre-op	Nothing pre-op – only “usual leisure activities	Face to face;<br>Clinic;	NR:<br>NR

ID	1st Author, year and country	Total *n* Intervention (I) Control (C) Number analysed (An if reported)	Patient population, baseline clinical characteristics (mean (SD) or *n (%)* unless otherwise stated	Demographics (mean (SD) or *n* unless otherwise stated)	Intervention	Comparator	Mode of delivery; place of delivery; training level of individuals who delivered the intervention; the number of contacts	Intervention fidelity; Compliance or adherence to intervention
**MULTIMODAL INTERVENTIONS**								
	Taiwan	undergoing total knee arthroplasty I=126:C=117	**Severity of illness** Moderate **(mean Ahlback score** of 3.04 (0.82) and **ASA** of 2.56 (0.50))	**Overal Gender (M%)** 28	40 min initial session with experienced physio for pre-op education program Taught exercises (straight leg raises, knee setting, ankle pumping, hip abduction) + protocol for TKA hospitalization/discharge program, rehabilitation program, fall prevention etc) 3 sets of repetitions done Unclear how often	and exercise were not prohibited"	Experienced physiotherapist; Phone call from physio one wk prior to admission	
12	Huang et al., 2017 STUDY 2 China	90 ppts with lung cancer awaiting lobectomy<br><br>Conventional resistance training (CRT) + IMT=30 Control=30<br><br>A 3rd arm is dscribed above (see Huang 2017 STUDY 1 IMT=30)	**Currently smoking %** I= 23.3:C=23.3 **ASA > III %** I=10:C=6.7 **COPD %** I=16.7:C=20 **BMI>30 (kg/m^2)** I=0:C=16.7 **VATS (video assisted thoracic surgery) %** I=56.7:63.3 **Open thoracotomy%** I=43.3:C=36.7	**Age, yrs** I=63.0 (8.7) C=63.6 (6.5) **Gender (%M)** I=66.7:C=70	combined PR group 1 wk with high-intensity preoperative PR using IMT and aerobic endurance exercise. It was a primarily a physical-based intervention that focused on exercise endurance and resistance training or a combination of methods, such as inspiratory muscle training (IMT), and CRT, coupled with psychological-educational guidance to cope with pre-operative anxiety and depression, perioperative activities. 9x 20 min training sessions/day. NR how many wks before surgery.	Usual care: preoperative preparation and essential encouragement or psychological caring.	Face-to-face; written; Patient ward; under the supervision and guidance of trained nurses; daily (at least 7 sessions)	NR: I=90% completed the 7 day prehabilitation period
13	Kassouf et al., 2018 Canada (Preliminary results only)	64 ppts undergoing cystectomy I=32:C=32	**Overall BMI kg/m2** 27 (5) Hb 118 (21) Pre-Alb 118 (21)	**Overall Age yrs** 66 (11)	Moderate aerobic and resistance exercises, nutritional counseling with protein supplementation, and relaxation exercises initiated 4 wks before surgery	Moderate aerobic and resistance exercises, nutritional counseling with protein supplementation, and relaxation exercises 4 wks postoperatively	Home-based; NR; NR; NR	NR; NR

ID	1st Author, year and country	Total *n* Intervention (I) Control (C)<br><br>Number analysed (An if reported)	Patient population, baseline clinical characteristics (mean (SD) or *n (%)* unless otherwise stated	Demographics (mean (SD) or *n* unless otherwise stated)	Intervention	Comparator	Mode of delivery; place of delivery; training level of individuals who delivered the intervention; the number of contacts	Intervention fidelity; Compliance or adherence to intervention
**MULTIMODAL INTERVENTIONS**								
			**BL distance walked in six mins (6MWD) m**<br>I=399.4 (150)<br>C=421.8 (131)					
14	Kasvis et al., 2018<br>Poster<br>Preliminary results only	Ppts with hepatopancreato- biliary cancer (HPBC)<br><br>14 ppts recruited to date:<br>I=7:C=7	**NR**	**Age, yrs**<br>I=54.4 (17.4):<br>C=60.0 (8.0)	Exercise by kinesiologist: Aerobic, resistance and stretching exercises<br>Nutrition by dietician: 1.5kg/day of protein intake<br>Relaxation by psychologist: deep abdominal breathing, meditation, and muscle relaxation techniques + CD for home use for 4 wks	Rehabilitation (intervention delivered PO)	Face to face;<br>Supervised by kinesiologist, dietician, psychologist;<br>Experienced;<br>Daily;<br>NR but assumed at least wkly	NR;<br>NR
15.	Lai et al., 2017, Lai et al., 2016<br>China	60 elderly population (>70 yrs) with lung cancer undergoing lung resection<br>I=30 (30An)<br>C=30 (30An)	**BMI >30 kg/m^2**<br>I=3(10.0):<br>C=2(6.7)<br>**Comorbidities**<br>**COPD n(%)**<br>I=5(17):C=4(13)<br>**Severity of illness**<br>**ASA score >3**<br>I=2(7):C=3(10)<br>**Clinical stage**<br>**Stage I**<br>I=16(53):C=18(60)<br>**Stage II**<br>I=10(33):C=10(33)<br>**Stage III**<br>I=3(10):C=2(7)<br>**Stage IV**<br>I=1(3):C=0(0)	**Age yrs**<br>I=72.5(3.4):<br>C=71.6(1.9)<br>**Overall gender (%M)**<br>53<br>**Smoking %**<br>I=20.0:C=16.7	The program was primarily a physical intervention focusing on exercise endurance training and inspiratory muscle training (IMT).<br>7-day intensive course<br>In hospital	Usual care	Verbal;<br>Hospital: IMT (performed in the ward) and aerobic endurance training (performed in the rehabilitation training center);<br>Physiotherapist;<br>Daily	NR;<br>4 ppts in the I group suspended the training because they could not endure the highly intensive regimen.

ID	1st Author, year and country	Total *n* Intervention (I) Control (C)<br><br>Number analysed (An if reported)	Patient population, baseline clinical characteristics (mean (SD) or *n (%)* unless otherwise stated	Demographics (mean (SD) or *n* unless otherwise stated)	Intervention	Comparator	Mode of delivery; place of delivery; training level of individuals who delivered the intervention; the number of contacts	Intervention fidelity; Compliance or adherence to intervention
**MULTIMODAL INTERVENTIONS**								
16	Lai et al., 2019 China	68 ppts with non small cell lung cancer undergoing video-assisted thoracic surgery (VATS) lung cancer lobectomy<br>I=34 (34An)<br>C=34 (34An)	**Hypertension%**<br>I=23.5:C=8.82<br>**COPD%**<br>I=I33.3:C=26.5<br>**Diabetes mellitus%**<br>I=8.82:C=2.94	**Age, yrs**<br>I=64.2 (6.8)<br>C=63.4 (8.2)<br>**Gender (%M)**<br>I=53:C=50	Multimodal intervention including aerobic exercise and breathing exercises for 1 wk	Usual care	Face -to-face;<br>Assumed in hospital;<br>Specialised nurses and physical therapists;<br>4 supervised sessions/day for 7 days	NR;<br>32/34 complete the planned intervention.
17	Liu et al., 2019 China	85 ppts <70 years old with newly suspected or confirmed non small cell lung cancer (NSCLC) clinical stage I-III, scheduled for a VAT lobectomy.<br>I=43 (37An)<br>C=42 (36An)	**ASA I /II/III %**<br>I=54/44/35<br>C=44/11/11<br>**Hypertension %**<br>I=22:C=31<br>**Diabetes %**<br>I=11:C=14<br>**Ischemic heart disease %**<br>I=8:C=6<br>**Cardiac arrhythmia %**<br>I=11:C=14<br>**Cerebral infarction %**<br>I=5:C=8<br>**COPD**<br>I=0:C=3<br>**Asthma**<br>I=14:C=6	**Age, yrs**<br>I=56.2 (10.3)<br>C=56.2 (8.7)<br>**Gender (%M)**<br>I=32:C=31<br>**Smoked %**<br>**Never**<br>I=89:C=94<br>**Current**<br>I=8:C=6<br>**Former**<br>I=3:C=0	Multimodal prehabilitation interventions, including aerobic and resistance exercises, respiratory training, nutritional counseling with whey protein supplementation, psychological adjustment and conventional guidance for approx. 2 wks<br><br>A booklet to record activity<br>wkly telephone calls with the same standardized question<br>30 mins of home-based exercise 3x/wk<br>Resistance exercise performed 2 x/wk<br>Respiratory training over 3 sessions<br>A 3-day recall food questionnaire + Advice following 1.5 g/kg/d.1 of whey protein<br>basic mental relaxation skills<br>relaxing music every day before sleeping	Usual care	Booklet; verbal;<br>Hospital & home;<br>Doctor of physical therapy;<br>Wkly calls	NR;<br>Diaries and phone calls were used to calculate compliance (defined as <70% execution of the plan). No ppts were excluded for lack of compliance

ID	1st Author, year and country	Total *n* Intervention (I) Control (C)<br><br>Number analysed (An if reported)	Patient population, baseline clinical characteristics (mean (SD) or *n (%)* unless otherwise stated	Demographics (mean (SD) or *n* unless otherwise stated)	Intervention	Comparator	Mode of delivery; place of delivery; training level of individuals who delivered the intervention; the number of contacts	Intervention fidelity; Compliance or adherence to intervention
**MULTIMODAL INTERVENTIONS**								
18	Minnella et al., 2019, Minnella et al., 2018, Minnella et al., 2016, Canada	68 adults with non-metastatic esophagogastric cancer awaiting elective esophagogastric resection<br>I=32 (+2 who withdrew consent)<br>C=30 (+ 4 who withdrew consent)<br>51 analysed<br>I=26:C=25		**Age**<br>I=67.3(7.4): C=68(11.6)<br>**Gender (M%)**<br>I=69:C=80	Pre-operative exercise and nutrition optimization in addition to standard perioperative care:<br><br>Exercise: Assessment of BL fitness; Individualised exercise program consisting of 30 mins aerobic exercise, 3 days/wk and 30 mins resistance training, 1 day/wk<br>Nutrition: Assessment of BL dietary habits. Food-based dietary advice and daily whey protein supplement to guarantee a daily protein intake of 1.2 to 1.5g/kg (using ideal body weight) or approximately 20% of total energy requirements<br><br>Median length of prehabilitation programme: 36 days (IQR 17 – 73)	**Active Comparator:** Exercise<br>Ppts in this group will follow standard McGill University Health Centre (MUHC) protocol of nutritional counseling and supplementation as needed in order to maintain caloric and protein requirements in the preoperative period. Additionally, these ppts will be given a specific physical exercise program before and after surgery by kinesiologist.<br><br>**Control**<br>standard MUHC protocol of nutritional counseling and supplementation as needed in order to maintain caloric and protein requirements in the preoperative period. + general instructions on exercises (breathing, ankle rotation) to be done during hospital stay by kinesiologist. Standardised perioperative care according to ERAS Society Guideline protocol.	Face to face;<br>MUHC, Home-based program;<br>Kinesiologist /dietitian;<br>1 phone call each from kinesiologist and dietitian/wk	NR;<br>63% compliance

ID	1st Author, year and country	Total *n* Intervention (I) Control (C)<br><br>Number analysed (An if reported)	Patient population, baseline clinical characteristics (mean (SD) or *n (%)* unless otherwise stated	Demographics (mean (SD) or *n* unless otherwise stated)	Intervention	Comparator	Mode of delivery; place of delivery; training level of individuals who delivered the intervention; the number of contacts	Intervention fidelity; Compliance or adherence to intervention
**MULTIMODAL INTERVENTIONS**								
19	Morano et al., 2013<br>Brazil	24 ppts with non-small cell lung cancer scheduled for resection<br>I=12 (12An)<br>C=12(9An)	Excluded ppts with previous pulmonary disease, interstitial lung disease, or obstructive airway disease, or impaired respiratory function by spirometry.<br>**Co-morbidity (%)**<br>I=58:C=33	**Age, yrs**<br>I=64.8 (8.0)<br>C=68.8 (7.3)<br>**Gender (%M)**<br>I=33:C=42<br>**Smoker (%)**<br>I=83:C=75	4 wks (5 sessions/wk) pulmonary rehabilitation (strength and endurance training: upper limb and lower limb exercises involving weights and a treadmill) plus IMT (10 to 30 mins daily, training load set at 20% of MIP for 1st wk and increased by 5% to 10% each session, to reach a 60% of MIP).	Chest physical therapy (consisted of a set of instructions about techniques for lung expansion, i.e. various breathing exercises).<br><br>Ppts in both groups participated in classes about the importance of preoperative and PO care and knowledge of the surgical process. Assume this was usual care, but not explicitly stated.	Not stated but likely supervised intervention at the hospital (use of gym equipment); likely training level of those who delivered the intervention high; number of contacts 5 weekly for 4 weeks before surgery.	NR;<br>NR; but assume high as intervention likely supervised and conducted in hospital
20	Ommundsen et al., 2018<br>Norway	122 ppts scheduled for resection of colorectal cancer<br><br>I=57 (53An)<br>C=65 (63An)	> 65 years with frailty<br>**Malnourished (%)**<br>I=23:C=35	**Age yrs**<br>I=78.2(7.4)<br>C=78.8(7.8)<br>**Gender (%M)**<br>I=42:C=59<br>**Severe comorbidity(%)**<br>I=59:C=49	Preoperative geriatric assessment and tailored intervention performed during one session, occurring as soon as possible after diagnosis confirmed and surgery planned. Intervention was treatment of comorbidities, optimization of drugs e.g. beta-blockers, statins and antiplatelet drugs; chest physiotherapy in COPD, dietary advice and prescription of oral nutritional supplements inc. micronutrients for ppts with malnutrition, etc. Encouraged to maintain physical activity.	Usual care	Face-to-face;<br>In hospital,<br>Medical doctor;<br>Delivered during one session	NR,<br>NR, although 9 ppts in I group had no problem areas that needed intervention. The remaining 44 went through intervention
21	Pehlivan et al., 2011<br>Turkey	60 ppts undergoing lung cancer resection (lobectomy or	**Severity of illness**<br>NR (stated in methods that each ppt was	**Age yrs**<br>I=54.1(8.5):<br>C=54.8(8.45)<br>**Smoking**<br>(Pack/yr)	Physical therapy 1 wk prior to surgery<br>Walking exercise on treadmill 3x/day	No intense physical therapy and no exercise. Routine physical therapy performed until discharge in both groups	Verbal (exercise) and direct (PT);<br>Hospital (surgical unit);<br>Physical therapist;<br>3 x/day for exercise; 2x/ day for PT	NR;<br>NR

ID	1st Author, year and country	Total *n* Intervention (I) Control (C)<br><br>Number analysed (An if reported)	Patient population, baseline clinical characteristics (mean (SD) or *n (%)* unless otherwise stated	Demographics (mean (SD) or *n* unless otherwise stated)	Intervention	Comparator	Mode of delivery; place of delivery; training level of individuals who delivered the intervention; the number of contacts	Intervention fidelity; Compliance or adherence to intervention
**MULTIMODAL INTERVENTIONS**								
		pneumonectomy)<br>I=30:C=30	classified according to severity)	I=46.8(23.6): C=50.8(26.6)	Chest physiotherapy applied 2x/day<br><br>Suggests that they stayed in the surgical unit for the wk prior to surgery (as "in the control group…ppts were discharged… and recalled the night before the panned surgery").			
22	Rosenfeldt et al., 2011 Australia	117 ppts undergoing elective CABG and/or valve surgery<br>I=60:C=57	**Co-morbidities**<br>Diabetes (%)<br>I=20:C=29	**Age yrs**<br>Median (IQR)<br>I=62.5(59-68.5): C=68(58-77)<br>**Gender (%M)**<br>I=78:C=70	Holistic therapy – physiotherapy & stress reduction exercises for the first 2 wks after joining waiting list<br><br>Physiotherapy: 2x 60min exercise prog./wk. Encouraged to do additional 30min exercise at least 2x/wk. After 2wk period encouraged to continue at least 4 30m sessions/wk.<br>Mental stress reduction: 4x 60 min sessions (family members encouraged to attend) with homework and handouts. Encouraged to practice relaxation techniques daily using CD or relaxing music for 20 min every day.	Usual care	Face to face then at home;<br>Outpatients then home;<br>Physiotherapist and occupational therapist;<br>NR	NR;<br>NR
23	Sawatzky et al., 2014	17 ppts undergoing elective CABG<br>I=8:C=9	Pts with a min. estimated 4 wk wait time, no history of unstable angina, recent MI (within last wk), or dementia, ejection fraction	**Age yrs**<br>I=64 (7): C=63 (9)<br>**Gender (%M)**<br>I=75:C=86	Exercise (aerobic and resistance) and education classes for 60 mins/day, 2x wkly for at least 4 wks. 12 class-based education sessions concerning medication use, exercise, stress, diet, and cardiovascular risk factor management.	Usual care (no prehab)	1-to-1, then group;<br>Medical fitness facility;<br>Physiotherapists (plus cardiologists and nurses for monitoring ppts);<br>Supervised intervention so FU at every session	NR;<br>Ppts attended a mean of 19 (sd 7) exercise sessions over a mean exposure time of 8.2 (sd 2.2) wks

ID	1st Author, year and country	Total *n* Intervention (I) Control (C)<br><br>Number analysed (An if reported)	Patient population, baseline clinical characteristics (mean (SD) or *n (%)* unless otherwise stated	Demographics (mean (SD) or *n* unless otherwise stated)	Intervention	Comparator	Mode of delivery; place of delivery; training level of individuals who delivered the intervention; the number of contacts	Intervention fidelity; Compliance or adherence to intervention
**MULTIMODAL INTERVENTIONS**								
			>30% and who were sedentary.		Min 4 wks.			
24	Vagvolgyi et al., 2018., Hungary	238 ppts with COPD undergoing thoracic surgery for primary lung cancer, lung metastases, benign disease, infection and other causes.<br>I1=72<br>I2= 80<br>I3=86	**BMI (kg/m²)**<br>I1=27 (5)<br>I2=25 (5)<br>I3=27 (6)<br>**Hypertension%**<br>I1=56<br>I2=53<br>I3=52<br>**Diabetes%**<br>I1=31<br>I2=28<br>I3=27<br>**Atherosclerosis%**<br>I=28<br>I2=26<br>I3=28<br>**Pulmonary hypertension%**<br>I1=13<br>I2=11<br>I3=9	**Age yrs**<br>I=65 (7)<br>I2=61 (10)<br>I3=65 (6)<br>**Gender (%M)**<br>I1=65<br>I2=54<br>I3=49	I1: preoperative pulmonary rehabilitation (3 wks).<br>I2= postoperative pulmonary rehabilitation (3 wks).<br>I3=Pre and postoperative pulmonary rehabilitation (6 wks).<br><br>Rehabilitation programme consisted of respiratory training (10-30 min cycle and/or treadmill, 2-3x/d at a level of 60-80% maximal intensity), chest wallmobilization, learning controlled breathing techniques, inhalation, expectoration, psychological support, smoking cessation and a session of further personalized training for each ppt.<br><br>For this review I3 was the intervention of interest.	Ppts undergoing thoracic surgery receive pulmonary rehabilitation as part of usual care, therefore for the purpose of our review I2 was considered the control group	Assume verbal;<br>assumed in hospital;<br>trained indiviiduals;.<br>3xday for 3 wks	NR;<br>NR.
25	Wang et al., 2017<br>Country NR (Abstract only Preliminary data)	90 ppts<br>I (combined pulmonary rehabilitation, PR)=30<br>I=30:C=30	Lung cancer undergoing surgery	NR	Combined PR: inspiratory muscle training (IMT) and aerobic endurance training<br>Single IMT<br><br>Duration of interventions not reported. Intervention started 1 wk preoperatively.	Usual care (routine preoperative preparation)	NR;<br>NR;<br>NR;<br>NR	NR;<br>NR

KEY: ASA= American Society of Anaesthesiologists physical status classification; An=analysed; BL=baseline; BMI=body mass index; C=control; CABG=coronary artery bypass graft; CD=compact disc; COPD=Chronic Obstructive Pulmonary Disease; hr=hour; I=intervention; IMT=Inspiratory muscle training; IQR=interquartile range; kg=kilogram; M=male; min=minute; mth=month; no.=number; NR=not reported; ppts=participants; pre-op=preoperative; prehab=prehabilitation; SD=standard deviation; TKA=total knee arthroplasty; wk=week; yrs=years

MULTIMODAL INTERVENTIONS

Table 18. Results

	Study	Total number of withdrawals	Clinical outcomes (mean (SD) or n unless otherwise stated)	Intervention-specific outcomes [(n or mean (SD) unless otherwise reported)] and economic evaluations
1	Abdelaal et al., 2017	0	**LoS days, median (range) I=26:C=24**<br>I=6 days (2-10):C=9 days (4-14) P NR<br>LoS days *mean/sd Quantile Estimation (QE) conversion method)*<br>*I= 6.00 (2.26): C= 9.00 (2.89)*<br>**Total PO Complications (up to 5th PO day) I=26:C=24**<br>I=7/26:C=15/24 had PO pulmonary complications (P=0.034)<br>**Atelectasis**<br>I=3/26:C=7/24<br>**Atelectasis with pneumonia**<br>I=0/26:C=5/24<br>**Pneumonia with respiratory failure**<br>I=0/26:C=3/24<br>**Pneumonia**<br>I=2/26:C=0/24<br>**Pleural effusion**<br>I=2/26:C=0/24	**Outcomes reported: pulmonary functions (MIP, MEP, SVC, IC, 6 MWT)**<br>Ppts in the I group had improved pulmonary functions compared to C group immediately PO, 2 days, 5 days (except SVC) and 1 mth (only IC and 6 MWT measured) after surgery.
2	Allen et al., 2019	I=3<br>C=5	**LoS, days (median), range NR**<br>I=11:C=16, P=0.155<br>**PO complication rates**<br>I=11/22:Control=17/23,P=0.89<br>**Depression Scores (BDI)**<br>I group had significant improvement in depression scores compared to C group<br>I=-2.71:C= +0.57; P=0.003	**Outcomes reported: VO^2 (CPET); dose reduction; chemotherapy - related toxicity; skeletal mass end of intervention**<br>**VO^2**<br>I group had an improvement in peak VO^2 following NAC (P=0.004) with a trend towards lesser reduction in anaerobic threshold (P=0.342).<br>**Dose reducation**<br>A higher proportion of controls required NAC deferral or dose reduction (P=0.041), with 72% vs 46% completing all cycles at full dose (P=0.076). There was no difference in chemotherapy-related toxicity.<br>**Skeletal mass**<br>I group had less skeletal muscle loss following NAC (P=0.049). Controls showed a trend towards more sarcopenia development after NAC I:Pre=37%/post=54% vs C:Pre=32%/post=64% (P=0.404).<br><br>**Adverse events**<br>no adverse events reported
3	Ausania et al., 2019	0 (ITT analysis)	**Mortality (in hospital)**<br>I=0/18:C=0/22<br>(There were no postoperative deaths)<br>**LoS (days) median (range) I=18:C=22**<br>I=11.4 (7-46):C=13.2 (7-60), P=0.449<br>LoS days *mean/sd Quantile Estimation (QE) conversion method)*<br>I=17.92 (16.35):C= 20.85 (20.56)	**Outcomes reported: FEV 1, FVC, dynamometer strength test, 10M walk test.**<br>Only within-group change scores reported. All improved after the intervention.

	Study	Total number of withdrawals	Clinical outcomes (mean (SD) or n unless otherwise stated)	Intervention-specific outcomes [(n or mean (SD) unless otherwise reported)] and economic evaluations
			**Readmission**<br>I=1/18:C=2/22, P=0.673<br>**PO complications**<br>I=6/18:C=12/22, P=0.18<br>**Major complications (types III-IV)**<br>I=4/18:C=4/22, P=0.751<br>**Pancreatic leak**<br>I=2/18:C=6/22, P=0.204<br>**Delayed gastric emptying**<br>I=1/18:C=9/22, P=0.01	
4	Arthur et al., 2000	29 in total<br>I=10<br>C=16<br>+ 3 withdrew initially	**Mortality (6-8wk FU)**<br>1 man died of noncardiac causes between the 6- to 8-wk PO FU (group NR)<br>**LoS after surgery (days: median (IQR)) I=113:C=107**<br>I=5(5-6):C=6(5-7)<br>Estimated diffs in median 95% CI: 1.0 (0.98 to 1.0), P=0.001<br>LoS days *mean/sd Quantile Estimation (QE) conversion method)*<br>*I=5.37(0.77); C=6.00(1.48)*<br>**Actual time in ICU, hr**<br>I=24.67 (21.68–41.85):C=26.71 (22.76–46.50**)** Estimated diffs in median 95% CI: 2.0 (21.2 to 16.0), P=0.038<br>**Total time in hospital, days**<br>I=6 (5–7):C=7 (6–8) Estimated diffs in median 95% CI: 1.0 (0.0 to 1.0) P=0.002<br>The 2 groups did not differ in certain markers of PO complications, such as episodes of atrial fibrillation (chi-square test, 1.02; P>0.2)<br>**QoL SF-36 (I=111: C=109) during the waiting period**<br>Physical role I=9.46 (34.39):C=22.06 (33.70), P=0.01<br>Physical functioning I=-1.17 (18.46):C=-6.56 (20.12), P=0.04<br>General health I=8.22 918.20): C=4.14 (18.78), P=0.10<br>Bodily pain I=3.58 (22.24): C=4.11 (20.54), P=>0.02<br>Physical Composite Summary Score I=1.55 (7.48):C=21.46 (7.81), P= 0.04<br>Vitality -0.95 (18.46):C=-1.19 (15.48), P=>0.02<br>Social functioning 4.50 (24.70):C=0.92 (24.10), P>0.02<br>Emotional role 7.51 (45.32):C=16.82 (44.82), P=0.13<br>Mental health 2.05 (18.52): C=0.77 (17.11), P=>0.2<br>Mental Composite Summary Score I=1.54 (10.55):C=2.93 (9.15), P=>0.2	**Outcomes reported: Adverse events during waiting period while doing the intervention (unlikely to be related to the intervention)**<br>Worsened cardiac status: I=4:C=8<br>Unstable angina: I=1:C=5<br>MI: I=1:C=2<br>Surgery brought forward due to worsening condition: I=2:C=1
5	Barberan-Garcia et al., 2018	19 lost to FU<br>I=8<br>C=11	**Mortality (in hospital)**<br>I=1/62:C=1/62<br>**LoS I=62: C=63**<br>I=8 (8):C=13 (20), P=0.078<br>**ICU LoS [mean (SD)]**<br>I=1 (2):C=4 (13), P=0.078	**Outcomes reported: Aerobic capacity (Endurance time), 6MWT (m)**:<br>Endurance time: slightly different between groups, P<0.001)<br>6MWT (no difference)

	Study	Total number of withdrawals	Clinical outcomes (mean (SD) or n unless otherwise stated)	Intervention-specific outcomes [(n or mean (SD) unless otherwise reported)] and economic evaluations
			**Surgical reintervention**<br>I=2/62:C=6/63<br>**PO complications (total ppts) I=62: C=63**<br>**Medical I=62: C=63**<br>**Cardiovascula**r I=1/62:C=8/63<br>**Respiratory** I=4/62:C=10/63<br>**Neurological** I=2/62:C=5/63<br>**AKI** I=0/62:C=4/63<br>**Nausea/vomiting** I=3/62:C=6/63<br>**DVT** I=0/62:C=1/63<br>**UTI** I=3/62:C=4/63<br>**Bloodstream infection (lab confirmed)** I=1/62:C=4/63<br>**Infection of uncertain source** I=0/62:C=7/63<br>**Others** I=6/62:C=13/63<br>**Surgical**<br>**PO hemorrhage** I=4/62:C=6/63<br>**Anastomotic breakdown** I=3/62:C=3/63<br>**Paralytic ileus** I=0/62:C=10/63<br>**Surgical site infection (superficial and deep)** I=1/62:C=1/63<br>**Surgical site infection (organ and space)** I=1/62:C=1/63<br>**Mechanical ileus** I=1/62:C=0/63<br>**QoL SF36 I=54:C=56**<br>**Physical Component Summary**<br>No between group results<br>**Mental Component Summary**<br>No between group results<br>**HAD total score (psychological) I=54:C=56**<br>No between group results<br>**Yale physical activity survey I=54:C=56**<br>No between group results	
6	Benzo et al., 2011 STUDY 2	2 ppts (1 from each arm) were excluded from LoS analysis	**LoS days I=9:C=8**<br>I= 6.3 (3.0):C=11 (6.3). P=0.058 (Diff. figures in table and text)<br>**ICU hrs I=9:C=8**<br>I=14.9 (44.7): C=40.5 (75.2), P=0.39<br>**PO Pulmonary complications (PCCs):**<br>I=3/9:C=5/8, P=0.23<br>**Pneumonia**<br>I=1/9: C=2/8, P=0.45<br>**Respiratory failure**<br>I=1/9:C=2/8, P=0.45<br>**Average number of days with chest tubes**<br>I= 4.3 (2.1):C=8.8 (5.3), P=0.04<br>**No. of ppts that had prolonged Chest Tube (>7days)**<br>I=1/9:C= 5/8, P=0.03	**Outcomes reported: shuttle walk test (m):**<br>Ppts did not improve the shuttle walk test (BL test was 31 shuttles or 310 metres) after the short term intervention (p=NS).<br><br>**Adverse events**<br>None reported

	Study	Total number of withdrawals	Clinical outcomes (mean (SD) or n unless otherwise stated)	Intervention-specific outcomes [(n or mean (SD) unless otherwise reported)] and economic evaluations
			**No. of ppts requiring bronchoscopy for atelectasis**<br>I= 1/9:C= 2/8, P=0.45	
7	Beaupre et al., 2004	Cancelled surgeries (thus no FU)<br>I=10:C=6<br><br>Died before 3 mth assessment<br>I=2 (1 unrelated to surgery)<br><br>Withdrew<br>I=2:C=2<br>But included in ITT analysis<br><br>18 ppts missed 1 of the PO visits:<br>9 missed the 3-mth (I=2:C=7)<br>9 missed the 6-mth I=7:C=2<br>No further withdrawals at 1-yr assessment'.	**Mortality (before 3 mth FU)**<br>I=2/65:C=0/66<br>**Acute care LoS I=55:C=60)**<br>I=6.7 (2.2):C=7.3 (2.5), P=0.14<br>**Transfer LoS (I=23:C=31)**<br>I=7.7 (2.0):C=7.7 (2.8), P=0.66<br>**Readmission LoS (I=5:C=6)**<br>I=3.4 (0.55):C=3.8 (2.0), P=0.95<br>**Total LoS days (I=55:C=60)**<br>I=10.2 (4.5):C=11.7 (5.2), P=0.1<br>**Readmission in first year, total N=11**<br>I=5 (1 due to poor ROM):C=6 (2 due to poor ROM)<br>**Infection**:<br>Superficial infection<br>I=2/55: C=3/60, P 1.0<br>**People without any complications during hospital stay**<br>84/115 (similar between groups)<br>**Non-infection**<br>**Pulmonary embolism**<br>I=2/55: C=2/60, P=1.00<br>**Thromboembolism**<br>I=2/55: C=3/60, P=1.0<br>**DVT**<br>I=3/55: C=6/60, P=0.49<br>**QoL**<br>Aside from the dimension of vitality (P=0.04) of the SF-36 questionnaire, no differences were seen between groups (p > 0.05) No significant interaction occurred between time and group in any clinical measure, indicating that the 2 groups followed a similar pattern of recovery throughout the study period.	**Outcomes reported: WOMAC pain, stiffness, function, Knee ROM (degrees), Quadriceps strength, Hamstring strength.**<br>There was no difference between groups on all outcomes at any time point<br><br>**Health utilization Costs:**<br>Institutional costs: I= 878 (1233):C=1090 (1316) P=0.38<br>Homecare costs I=127 (177):C= 117 (159) P=0.76<br>Community rehabilitation I=125 (226) C=159 (251) P=0.45<br>costs<br>Total costs I= 1369 (1274):C= 1366 (1415), P=0.99
8	Bousquet-Dion et al., 2018	7 excluded/withdrew<br>I=0<br>C=7	**LoS days [median (IQR)] (PP analysis: I=37:C=26)**<br>I=3 (3-4):C=3 (2-4), P=0.122<br>LoS days *mean/sd Quantile Estimation (QE) conversion method)*<br>*I=3.40(0.81); C=3.00(1.48)*<br>**30d Hospital readmission**<br>I=5:C=2, P=0.051<br>**30d emergency department visits**<br>I=7:C=7, P=0.975<br>**LoS [median (IQR)] (ITT analysis: I=41:C=31)**<br>I=3 (3-5):C=3 (2-4), P=0.111<br>**30 d Hospital readmission**<br>I=5:C=2, P=0.415	**Outcomes reported: change in 6MWT (m), BMI (kg/m^2), glycated haemoglobin (HbA1C):**<br>no difference between groups.<br>Self-reported physical activity higher in I vs C group (P=0.02)

	Study	Total number of withdrawals	Clinical outcomes (mean (SD) or n unless otherwise stated)	Intervention-specific outcomes [(n or mean (SD) unless otherwise reported)] and economic evaluations
			**30d emergency department visits**<br>I=7:C=7, P=0.559<br>**Ppts with at least one 30-day complication**<br>I=14/37: C=8/26, P=0.562<br>**Individual complications**<br>**Ileus** I=4/37:C=1/26, P=0.802<br>**Wound infection**<br>I=5/37:C=3/26<br>**Anemia** I=2/37:C=1/26<br>**Coronary heart failure** I=2/37:C=1/26<br>**Pain** I=2/37:C=0/26<br>**Bleeding** I=1/37:C=1/26<br>**Bowel obstruction** I=1/37:C=1/26<br>**AKI** I=1/37:C=0/26<br>**Anastomotic leak** I=2/37:C=0/26<br>**Grade of most severe complication (Clavien-Dindo):**<br>I I=9:C=4 P=0.269<br>II I=3:C=4<br>III I=2:C=0	
9	Crowe et al., 2003	None.<br>1 in I group did not undergo surgery.	**LoS days I=65:C=68**<br>I=6.6 (4.2):C=10.5 (14.2), P=0.032<br>**Total PO infective complications**<br>I=2/64:C=5/68<br>**Cardiac complications**<br>I=1/64:C=3/68<br>**Confusion**<br>I=3/64:C=5/68<br>**Hip dislocation**<br>I=0/64:C=2/58<br>**Other**<br>I=1/64:C=7/58<br>**No PO complications**<br>I=57/64:C=46/58, P=0.007	**Outcomes reported: get out of bed timings, joint flexion (ROM = 40° active hip flexion for hip arthroplasty clients, or 50° active knee flexion for knee arthroplasty clients), equipment/meal planning:**<br>Shorter time to Independently get out of bed (P=0.05) and joint flexion (P=0.08), Discharge equipment planning (P<0.0000) and Discharge meal planning (P<0.0000) in I vs C group.<br>No between-group difference in time to walk 30 m or climb stairs.
10	Demark-Wahnefried et al., 2016<br>USA	I: n=4 (2 cancelled prostatectomy<br>1 diagnosed with metastatic disease<br>1 withdrew consent)<br>C: n=2 (cancelled prostatectomy)	NR. Only feasibility parameters (adherence and retention) were reported	**Adverse events**<br>No adverse events observed or reported during the intervention.
11	Huang et al., 2012	none	**LoS days I=126:C=117**<br>I=7(2):C=8 (1), P=0.027<br>CI for diff=-0.795 to -0.044<br>**Total PO infective complications**	**Outcomes reported: Range of motion:**<br>No difference in range of motion day 1 or day 5 PO

	Study	Total number of withdrawals	Clinical outcomes (mean (SD) or n unless otherwise stated)	Intervention-specific outcomes [(n or mean (SD) unless otherwise reported)] and economic evaluations
			I=2/126:C=1/117, P=0.605<br>**DVT**<br>I=5/126:C=2.6 3/117<br>**Blood transfusion**<br>I=15/126:C=10/117<br>**PO pain VAS mean (SD) (I=126: C=117)**<br>**1-day PO**<br>I=4.5 (1.3):C=4.4 (1.2), P=0.431<br>**5-days PO**<br>I=2.4 (0.7):C=2.5 (0.6), P=0.686	
12	Huang et al., 2017<br>STUDY 2	I=3 (had acute COPD exacerbation; 2 had new worsening of knee pain)<br>ITT<br>C=0	**Mortality**<br>I=0/30:C=1/30<br>**LoS, days I=30:C=30**<br>I=14.1 (2.7):C=17.3 (4.3)<br>**Readmission rate**<br>I=0/30:C=1/30<br>**Pneumonia**<br>I=4/30:C=7/30<br>**Anastomotic leak (air leak >7 days)**<br>I=2/30:C=2/30<br>**Pulmonary embolus**<br>I=0/30:C=1/30<br>**Pleural effusion needing tube**<br>I=0/30:C=2/30<br>**Respiratory failure**<br>I=0/30:C=1/30<br>**Atelectasis needing toilet bronchoscope**<br>I=2/30:C=1/30<br>**Empyema**<br>I=1/30:C=1/30<br>**Mechanical ventilation >48h**<br>I=1/30:C=2/30<br>**Bronchopleural fistula**<br>I=0/30:C=1/30<br>**Chylothorax**<br>I=0/30:C=1/30<br>**Grade I/II/III/IV/V complications**<br>14/4/3/1/0<br>16/8/3/2/1<br>**HRQol**<br>**EORTC QLQ-C30&LC13_CN (version 3)**<br>I=74.2 (12.1):C=67.5 (11.9)	**NR**
13	Kassouf et al., 2018	40 ppts analyzed<br>Lost to FU:	NR	**Outcomes reported: self-reported physical fitness:**

	Study	Total number of withdrawals	Clinical outcomes (mean (SD) or n unless otherwise stated)	Intervention-specific outcomes [(n or mean (SD) unless otherwise reported)] and economic evaluations
		I=14<br>C=10		Proportion of ppts experiencing a significant preoperative improvement in physical fitness higher in I vs C group (P<0.024)
14	Kasvis et al., 2018	NR	NR	**Outcomes reported: 6MWTD 4, 8 wks PO**<br>4 wks PO<br>the average decrease in 6MWTD in the rehab group was more than doubled compared to prehab and statistically different from BL (p=0.047).<br>At 8 wks, the 6MWTD of the prehab group exceeded BL whereas, the rehab group failed to return to BL (p>0.05).
15	Lai et al., 2016;<br>Lai et al., 2017	None	**Mortality (in hospital)**<br>I=0/30:C=1/30<br>**LoS days (I=30:C=30)**<br>I=16.0 (4.5):C=19.7 (6.5), P=0.012<br>**Preop days**<br>I=9.0 (1.1):C=9.0 (1.6), P=1.000<br>**PO days**<br>I=6.9 (4.4):C=10.7 (6.4), P=0.010<br>**Complications**<br>**Grade I Total**<br>I=15/30:C=16/30, P=0.796<br>**New-onset purulent sputum**<br>I=10/30:C=12/30<br>**Fever >38C, no focus outside the lungs**<br>I=9/30:C=8/30<br>**New rise in C-reactive protein or WBC count**<br>I=7/30:C=9/30<br>**Positive blood cultures**<br>I=4/30:C=5/30<br>**Atelectasis**<br>I=6/30:C=5/30<br>**Pleural effusion**<br>I=4/30:C=6/30<br>**Grade II Total**<br>I=4/30:C=8/30, P=0.197<br>**Pneumonia**<br>I=4/30:C=7/30<br>**Mechanical ventilation <48 hr**<br>I=2/30:C=1/30<br>**Pleural effusion needing tube**<br>I=1/30:C=2/30<br>**Atelectasis needing toilet bronchoscopy**<br>I=2:C=1/30<br>**Grade III Total**<br>I=2/30:C=4/30, P= 0.389<br>**Empyema**	**Outcomes reported: 6MWD (m), pulmonary function test (FEV1, FVC, D1Co, PEF), change from BL to PO**<br>A significant improvement was found for 6MWD (P=0.029) and PEF (P<0.001) in I vs C group.<br>There was no difference in the remaining pulmonary function markers (FEV1, FVC, DlCo)<br><br>**Adverse events due to intervention**<br>4 ppts in the I group suspended the training because they could not endure the highly intensive regimen.

	Study	Total number of withdrawals	Clinical outcomes (mean (SD) or n unless otherwise stated)	Intervention-specific outcomes [(n or mean (SD) unless otherwise reported)] and economic evaluations
			I=1/30:C=1/30<br>**Mechanical ventilation >48 hr**<br>I=1/30:C=2/30<br>**Bronchopleural fistula**<br>I=0/30:C=1/30<br>**Chylothorax**<br>I=0/30:C=1/30<br>**Grade IV**<br>I=0/30:C=1/30, P=1.000<br>**Return to ICU**<br>I=0/30:C=1/30<br>**Pulmonary embolism**<br>I=0/30:C=1/30<br>**ARDS or respiratory failure**<br>I=0/30:C=1/30<br>**QoL between group diffs (95% CI) I=30:C=30**<br>Global QoL -0.5 (-4.6 - 3.5), P=0.785<br>Physical function: -0.67 (-4.0 - 2.7), P=0.691<br>Emotional function: -2.2 (-5.7 - 1.3), P=0.206<br>Dyspnea score: 0.37 (-2.7 - 3.4), P=0.808	
16	Lai et al., 2019	I=2 (could not endure the training intensity<br>C=0	**Mortality**<br>I=0/34:C=0/34<br>**LoS, days median, IQR I=34:C=34**<br>I=5.0 (4.0-7.0):C=8.0(7.0-10.0)<br>*LoS days mean/sd Quantile Estimation (QE) conversion method)*<br>*I=5.69 (2.57):C=8.55 (2.37)*<br>**Return to ICU**<br>I=0/34:C=1/34<br>**Pneumonia or wound infection**<br>I=3/34:C=6/34<br>**Dyspnea**<br>I=8/34:C=9/34<br>**Cough**<br>I=6/34:C=9/34<br>**Microatelectasis, microaerothorax or air leakage**<br>I=6/34:C=6/34<br>**Mechanical ventilation <48h**<br>I=0/34:C=3/34<br>**Pleural effusion needing tube relocation or thoracentesis**<br>I=2/34:C=2/34<br>**Atelectasis needing bronchoscope and aspirations**<br>I=1/34:C=3/34<br>**Dyspnea pharmacological intervention or ventilator support**<br>I=2/34:C=4/34	**Outcomes reported: 6 MWT (m); Lung function (FEV1, FVC, DLco, PEF index of fatigue, index of dyspnea during 6MWT)**<br>**Blood gas analysis, EORTC QLQ-C30**<br><br>**6MWT**<br>Increase in 6MWT in I vs. C group, P=0.004.<br>**Lung function**<br>No difference between groups was found in lung function including FEV1, FVC, DLco, PEF, index of fatigue, index of dyspnea during 6-MWT, blood gas analysis, or EORTC QLQ-C30 scores except for emotional function (Z=3.363, P=0.001)<br><br>**Costs**<br>**Prehab - median (IQR)**<br>- Total – 48,588.7 (44,999.1–52,693.3)<br>- Material cost – 23,350.8 (18,300.6–26,421.9)<br>- Drug cost – 7,230.0 (6,661.9–8,347.4)<br><br>**Control – median (IQR)**<br>- Total – 52,445.3 (49,002.9–61,994.0)<br>- Material cost – 25,730.0 (21,328.7–29,250.2)<br>- Drug cost - 11,388.6 (7,963.0–16,314.3)

	Study	Total number of withdrawals	Clinical outcomes (mean (SD) or n unless otherwise stated)	Intervention-specific outcomes [(n or mean (SD) unless otherwise reported)] and economic evaluations
			**Air leak**<br>I=3/34:C=4/34<br>**Empyema**<br>I=0/34:C=1/34<br>**Mechanical ventilation >48h**<br>I=0/34:C=1/34<br>**Bronchopleural fistula**<br>I=0/34:C=0/34<br>**Chylothorax**<br>I=0/34:C=1/34<br>**Pulmonary embolus**<br>I=0/34:C=0/34<br>**ARDS**<br>I=0/34:C=0/34<br>**Single or multiorgan failure**<br>I=0/34:C=0/34<br><br>**HQol**<br>**Global QOL Median (IQR):**<br>I=70.8 (58.3-83.3):C=70.8 (58.3–83.3)<br>**Physical function**<br>I=86.7 (86.7–93.3):C=86.7 (86.7-93.3)<br>**Emotional function**<br>I=83.3 (83.3–91.7):C=83.3 (83.3–91.7)	
17	Liu et al., 2019	12 excluded:<br>Did not receive surgery<br>I=4:C=2<br>Lost to FU<br>I=2:C=3	**Mortality**<br>I=0/37:C=0/36<br>**LoS, days (median, IQR) I=37:C=36**<br>I=8 [6–10]:C=8 [7–11] P=0.57<br>*LoS days mean/sd Quantile Estimation (QE) conversion method)*<br>*I=8.00 (2.97):C=9.05 (3.33)*<br>**Pneumonia**<br>I=0/37:C=1/36<br>**Wound infection**<br>I=1/37:C=0/36<br>**Atelectasis**<br>I=0/37:C=1/36<br>**Cardiac complications**<br>I=3/37:C=2/36<br>**Chest tube duration**<br>I=0/26:C=1/35<br>**Conversion to open procedure**<br>I=0/26:C=0/35	**Outcomes reported: L6MWD (m); FEV1 (L); FVC (L);PEF (L/min); WHODAS2, (WHO disability assessment schedule)**<br>6MWD (m) P<.001<br>FEV1 (L) P=0 .240<br>FVC (L) P=0.021<br>PEF (L/min) P=0.339<br>WHODAS P=0 .152

	Study	Total number of withdrawals	Clinical outcomes (mean (SD) or n unless otherwise stated)	Intervention-specific outcomes [(n or mean (SD) unless otherwise reported)] and economic evaluations
			**Depression/anxiety**<br>HADS-Anxiety P=0.799<br>HADS-Depression P=0.277	
18	Minnella et al., 2016,2018, 2019	17 not analysed<br>Reasons NR<br><br>Missed second assessment<br>I=5:C=3<br>Died<br>I=1:C=2	**Mortality (in hospital)**<br>I=0/24:C=2/25<br>**LoS days (median/IQR) I=24:C=25**<br>I=8.0 (5.75-11.75):C=7.0 (5.5-12.5), P=0.44<br>LoS days *mean/sd Quantile Estimation (QE) conversion method)*<br>*I= 9.41 (5.47):C= 9.77 (7.48)*<br>**Complications**<br>Ppts with no complications I=10/24:C=7/25, P=0.24 (14/24:18/25)<br>**Comprehensive complication index (median, IQR)**<br>I=14.8 (0.0-28.8):C=20.9 (20.9-36.2), P=0.17<br>Clavien-Dindo classification of complication severity, in-hospital mortality, emergency department visit and readmission rate also reported, no differences between groups.	**Outcomes reported: walking distance (BL to preoperative assessment and 4 wks PO), 6MWD (m) over time**:<br>Walking distance change improved in I vs C, P<0.001:<br>A difference was also seen in improved functional capacity measured as change in 6MWD over time.
19	Morano et al., 2013	3 ppts in C group did not have surgery because of inoperable cancer.	**LoS, days I=12:C=9**<br>I=7.8(4.8):C=12.2(3.6)<br>**ICU stay (d) Median (IQR)**<br>I=2(2-3):C=2(2-4.5)<br>**PPCs**<br>I=2/12:C=7/9<br>**Pneumonia**<br>I=0/12:C=2/9<br>**Ventilation >48h**<br>I=1/12:C=3/9<br>**Bronchopleural fistula**<br>I=2/12:C=7/9<br>**Atelectasis**<br>I=0/12:C=3/9<br>**Bronchospasm**<br>I=0/12:C=6/9<br>**N days with chest tube**<br>I=4.5(2.9):C=7.4(2.6)	**Outcomes reported: FVC (% predicted), IVC (litres), MIP and MEP (cmH$_2$0), PaO$_2$ and PaCO$_2$ (mm Hg); FEV1 and FVC (l and %); 6MWT (m)**<br>During phase 1 evaluation, most functional parameters in the PR group improved from baseline to 1 month:<br>forced vital capacity (FVC; P<0.02); FVC%: P<0.05); 6-minute walk test; P<0.05); MIP P0<.05); and MEP; P0<.05)
20	Ommundsen et al., 2018	**9 lost to FU**<br>**I=4**<br>**C=3**<br><br>2 excluded from each group after randomization.<br>At 30 days:<br>I: 2 lost, 1 withdrew but included with last observation carried forward; C: 1 lost but	**30d mortality**<br>I=2/53:C=3/53, P=0.79<br>**LoS [days, median] I=53:C=53 IQR NR**<br>I=8:C=8, P=0.63<br>**Hospital readmission I=53:C=53**<br>I=8:C=4, P=0.12<br>**Reoperation I=53:C=63**<br>I=10:C=7, P=0.24<br>**PO complications (n) I=53:C=63**<br>**Grade I-V**	NR

Perry R, et al. BMJ Open 2022; 11:e050806. doi: 10.1136/bmjopen-2021-050806

	Study	Total number of withdrawals	Clinical outcomes (mean (SD) or n unless otherwise stated)	Intervention-specific outcomes [(n or mean (SD) unless otherwise reported)] and economic evaluations
		included with last observation carried forward.<br>Analyzed I=53:C=63)	I=40:C=55, P=0.10<br>After adjusting for prognostic factors, OR 0.33 (0.11-0.95)<br>**Grade II-V**<br>I=36:C=47, P=0.43<br>After adjusting for prognostic factors, the diff between groups was non-7sig<br>**No complications**<br>I=13/53:C=8/63, P=0.14	
21	Pehlivan et al., 2011	None reported	**Mortality (in hospital)**<br>I=0/30:C=0/30<br>**LoS days (I=30:C=30)**<br>I=5.4 (2.67):C=9.66 (3.09)<br>**PO Complications**<br>**Pneumonia**: I=0/30:C=0/30<br>**Atelectasis**: I=1/30:C=0/30<br>**Fever**: I=2/30:C=1/30<br>**Dyspnea**: I=1/30:C=0/30<br>**Haemorrhagic drainage**: I=1/30:C=0/30	**Outcomes reported: markers of pulmonary function e.g. FVC (L), FEV1 (L/sec), PaO2 (mmHg), etc. and arterial blood gases.**<br>Some within group changes in pulmonary function and arterial blood gases before and after preoperative IPT.<br>Differences between groups not reported.
22	Rosenfeldt et al., 2011	None	**LoS days (Median; IQR) (I=60:C=57)**<br>I=6(5-8):C=6 (5-8), P=0.54<br>LoS days *mean/sd Quantile Estimation (QE) conversion method)*<br>*I= 6.63 (2.47): C= 6.63 (2.47)*<br>**PO complications (non-infective)**<br>**PO atrial fibrillation**<br>I=22/60:C=19/57, P=0.71 (chi^2 test)<br>**SF-36 (I=60:C=57)**<br>**QoL**<br>**Mental QoL scale, post therapy**<br>I=45.4 (1.2):C=45.2 (1.6), P=0.91<br>**Physical QoL scale post therapy**<br>I=44.1 (1.0):C=42.8 (1.3), P=0.45	**Outcomes reported: Troponin [(µg/L) Protein found in muscles] at 24 hrs**<br>No between-group difference in Troponin (µg/L) P=0.91
23	Sawatzky et al., 2014	I=0<br>C=2 (unable to contact)	**LoS days (I=8:C=7)**<br>I=5.1 (1.4):C=5.3 (1.0)<br>**ICU LoS hrs (I=8:C=7)**<br>I=24 (12):C=25 (7)<br>**Non-infective complications**<br>**Atelectasis:**<br>I=2/8:C=0/7, P=0.47<br>**Atrial fibrillation:**<br>I=2/8:C=4/7, P=0.31<br>**Quality of life (SF-36)**<br>depression (Patient Health Questionnaire), self-efficacy (Cardiac Exercise Self Efficacy Index) between groups No difference between groups.	**Outcomes reported: 6MWT (m), 5-metre gait speed, physical activity parameters (BL to end of intervention**<br>No difference between groups (P>0.05).<br><br>**Adverse events**<br>No adverse events occurred during participation in the prehab programme (data not shown).

	Study	Total number of withdrawals	Clinical outcomes (mean (SD) or n unless otherwise stated)	Intervention-specific outcomes [(n or mean (SD) unless otherwise reported)] and economic evaluations
24	Vagvolgy et al., 2019	None reported	PO complications (classed as severe and not severe) but there was no comparison between groups.	**Outcomes reported:** pulmonary function (forced expiratory volume in 1 second, FEV1, and forced vital capacity, FVC); 6 minute walk distance (6MWD, M); dyspnea; breath holding time (s); grip strength (kg); cycle ergometry (time, min; power, watt; distance, km).<br><br>Reported all outcomes within groups (before intervention and after) but no between group comparisons.
25	Wang et al., 2017 (abstract only)	NR (although analysis was ITT)	**LoS**<br>Reduced by 3.6 days (P=0.001) in combined PR group vs. single IMT and usual care<br>**PO complications**<br>No diff in PO pulmonary complications between groups (data NR)	**Outcomes reported: 6MWD (m), Peak expiratory flow:**<br>In combined PR group vs C group, there was an increase in 6MWD (by 32.6m, P=0.002) and PEF (by 14.3 l/min, P=0.001)

KEY: AKI=acute kidney injury; BL=baseline; BDI=Becks Depression Inventory; C=control; CPET= cardiopulmonary exercise test; CI=confidence interval; Dlco=diffusion capacity of the lung for carbon monoxide; DVT=deep vein thrombosis; FEV1=forced expiratory volume in 1second; FVC=forced vital capacity; FU=follow up; HbA1C =Haemoglobin A1C; HAD=Hamilton anxiety depression scale; I=intervention; ICU=intensive care unit; ITT=intention to treat; IQR=interquartile range; LoS=length of stay; m=metre; min=minute; MI=myocardial infarction; maximum inspiratory and expiratory pressures; NAC= neoadjuvant chemotherapy; PEF=peak expiratory flow; PO=postoperative; PP=per protocol; ppts=participants; PR=pulmonary rehabilitation;; ROM=Range of movement; SVC=slow vital capacity; SD=standard deviation; VAS=visual analogue scale; WHODAS=World Health Organization disability assessment schedule; 6 MWT=6 minute walk test or 6MWD=6 minute walk distance

MULTIMODAL INTERVENTIONS

Table 19. Risk of bias

	Study	Selection bias		Performance bias		Detection bias						Attrition bias	Reporting bias
						Blinding of outcome assessment							
		Random sequence generation	Allocation concealment	Blinding of participants (all outcomes)	Blinding of personnel (all outcomes)	Perioperative mortality, hospital readmission	Postoperative complications	Length of stay	Patient reported outcomes (pain, HRQoL)	Intervention related outcomes	Intervention related adverse events	Incomplete outcome data	Selective reporting
1	Abdelaal 2017					NR			NR		NR		
2	Ausania 2019								NR		NR		
3	Allen 2019<br>Abstract only					NR			NR				
4	Arthur 2000						NR		NR	NR			
5	Barberan-Garcia 2018										NR		
6	Benzo 2011<br>STUDY 2					NR			NR				
7	Beupre. 2004										NR		
8	Bousquet-Dion 2018					NR			NR		NR		
9	Crowe 2003					NR			NR		NR		
10	Demark-Wahnefried, 2016					NR	NR	NR	NR	NR			
11	Huang 2012					NR					NR		
12	Huang 2017 STUDY 2										NR		
13	Kassouf 2018<br>Preliminary results only												
14	Kasvis 2018<br>Preliminary results only												

Perry R, *et al. BMJ Open* 2022; 11:e050806. doi: 10.1136/bmjopen-2021-050806

	Study	Selection bias		Performance bias		Detection bias						Attrition bias	Reporting bias
						Blinding of outcome assessment							
		Random sequence generation	Allocation concealment	Blinding of participants (all outcomes)	Blinding of personnel (all outcomes)	Perioperative mortality, hospital readmission	Postoperative complications	Length of stay	Patient reported outcomes (pain, HRQoL)	Intervention related outcomes	Intervention related adverse events	Incomplete outcome data	Selective reporting
15	Lai 2016; 2017												
16	Lai 2019										NR		
17	Liu 2019										NR		
18	Minella 2016, 2018 , 2019								NR		NR		
19	Morano 2013					NR			NR		NR		
20	Ommundsen 2018								NR	NR	NR		
21	Pehlivan 2011					NR					NR		
22	Rosenfeldt 2011												
23	Sawatzky 2014					NR					NR		
24	Vagolgy 2019					NR		NR	NR		NR		
25	Wang 2017<br>Abstract only<br>Preliminary results												

NR = not reported

MULTIMODAL INTERVENTIONS

Table 20. Summary of findings

Multimodal interventions compared to usual care for any major surgery

Patient or population: any major surgery
Setting: hospital
Intervention: multimodal interventions
Comparison: usual care

Outcomes	Anticipated absolute effects* (95% CI)		Relative effect (95% CI)	№ of participants (studies)	Certainty of the evidence (GRADE)	Comments
	Risk with usual care	Risk with multimodal interventions				
mortality follow up: 30 days	18 per 1,000	**12 per 1,000** (4 to 35)	**RR 0.67** (0.23 to 1.95)	771 (10 RCTs)	⨁⨁◯◯ LOW [a,b]	
Length of Stay (LoS)		MD **1.67 days lower** (2.31 lower to 1.03 lower)	-	1529 (18 RCTs)	⨁⨁⨁◯ MODERATE [c]	
pneumonia	66 per 1,000	**37 per 1,000** (18 to 74)	**RR 0.56** (0.28 to 1.12)	341 (5 RCTs)	⨁◯◯◯ VERY LOW [b,d,e]	
Total PO complications	465 per 1,000	**391 per 1,000** (335 to 451)	**RR 0.84** (0.72 to 0.97)	313 (5 RCTs)	⨁◯◯◯ VERY LOW [b,d,e]	

***The risk in the intervention group** (and its 95% confidence interval) is based on the assumed risk in the comparison group and the **relative effect** of the intervention (and its 95% CI).

CI: Confidence interval; **RR:** Risk ratio; **MD:** Mean difference

GRADE Working Group grades of evidence
High certainty: We are very confident that the true effect lies close to that of the estimate of the effect
Moderate certainty: We are moderately confident in the effect estimate: The true effect is likely to be close to the estimate of the effect, but there is a possibility that it is substantially different
Low certainty: Our confidence in the effect estimate is limited: The true effect may be substantially different from the estimate of the effect
Very low certainty: We have very little confidence in the effect estimate: The true effect is likely to be substantially different from the estimate of effect

Explanations

a. Downgrade Inconsistency: based on effects not in same direction;
b. Downgrade for imprecision- includes null effect and appreciable benefit or harm, small sample size
c. Downgrade Inconsistency: based on effects not in same direction, different sizes of CI, no overlap in CIs
d. Downgrade for Risk of bias
e. Downgrade Inconsistency: based on effects not in same direction, different sizes of CI;

Meta analyses

Figure 54. Random effects meta-analysis of the risk ratio of mortality between multimodal (experimental) and usual care (control).

Study or Subgroup	Experimental Events	Experimental Total	Control Events	Control Total	Weight	Risk Ratio M-H, Random, 95% CI
Ausania 2019	0	18	0	22		Not estimable
Barberan-Garcia 2018	1	62	1	62	15.0%	1.00 [0.06, 15.63]
Beupre 2004	2	65	0	66	12.5%	5.08 [0.25, 103.73]
Huang 2017 STUDY 2	0	30	1	30	11.3%	0.33 [0.01, 7.87]
Lai 2016; 2017	0	30	1	30	11.3%	0.33 [0.01, 7.87]
Lai 2019	0	34	0	34		Not estimable
Liu 2019	0	37	0	36		Not estimable
Minella 2016; 2018; 2019	0	24	2	25	12.7%	0.21 [0.01, 4.12]
Ommundsen 2018	2	53	3	53	37.1%	0.67 [0.12, 3.83]
Pehlivan 2011	0	30	0	30		Not estimable
**Total (95% CI)**		**383**		**388**	**100.0%**	**0.67 [0.23, 1.95]**
Total events	5		8			

Heterogeneity: Tau² = 0.00; Chi² = 2.78, df = 5 (P = 0.73); I² = 0%
Test for overall effect: Z = 0.73 (P = 0.46)

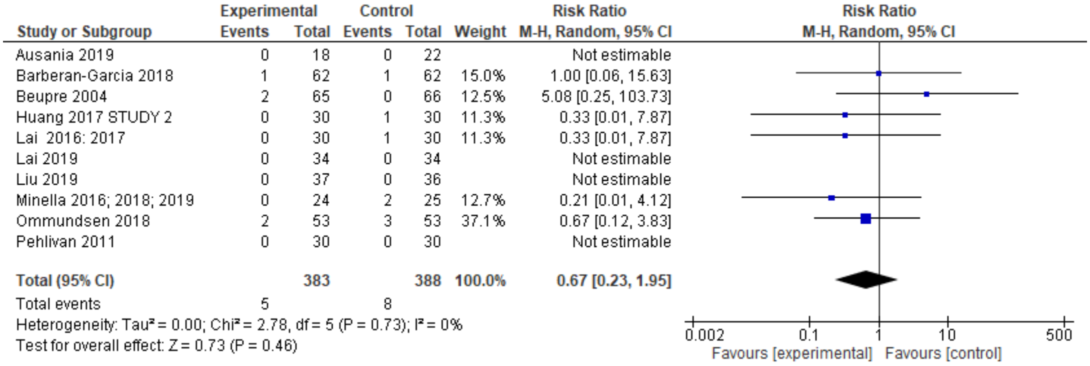

10/25 studies (771 participants).

Figure 55. Random effects meta-analysis of the mean difference in length of hospital stay (LoS) between multimodal intervention (experimental) and usual care (control).

Study or Subgroup	Experimental Mean	Experimental SD	Experimental Total	Control Mean	Control SD	Control Total	Weight	Mean Difference IV, Random, 95% CI
Abdelaal 2017	6	2.26	26	9	2.89	24	6.9%	-3.00 [-4.45, -1.55]
Arthur 2000	5.37	0.77	113	6	1.48	107	10.3%	-0.63 [-0.94, -0.32]
Ausania 2019	17.92	16.35	18	20.85	20.56	22	0.3%	-2.93 [-14.37, 8.51]
Barberan-Garcia 2018	8	8	62	13	20	63	1.3%	-5.00 [-10.32, 0.32]
Benzo 2011 STUDY 2	6.3	3	9	11	6.3	8	1.5%	-4.70 [-9.49, 0.09]
Beupre 2004	10.2	4.5	55	11.7	5.2	60	5.8%	-1.50 [-3.27, 0.27]
Bousquet-Dion 2018	3.4	0.81	37	3	1.48	26	9.6%	0.40 [-0.23, 1.03]
Crowe 2003	6.6	4.2	65	10.5	14.2	68	2.5%	-3.90 [-7.43, -0.37]
Huang 2012	7	2	126	8	1	117	10.1%	-1.00 [-1.39, -0.61]
Huang 2017 STUDY 2	14.1	2.7	30	17.3	4.3	30	5.7%	-3.20 [-5.02, -1.38]
Lai 2016; 2017	16	4.5	30	19.7	6.5	30	3.5%	-3.70 [-6.53, -0.87]
Lai 2019	5.69	2.57	34	8.55	2.37	34	7.8%	-2.86 [-4.04, -1.68]
Liu 2019	8	2.97	37	9.05	3.33	36	6.8%	-1.05 [-2.50, 0.40]
Minella 2016; 2018; 2019	9.41	5.47	24	9.77	7.48	25	2.4%	-0.36 [-4.02, 3.30]
Morano 2013	7.8	4.8	12	12.2	3.6	9	2.4%	-4.40 [-7.99, -0.81]
Pehlivan 2011	5.4	2.67	30	9.66	3.09	30	6.8%	-4.26 [-5.72, -2.80]
Rosenfeldt 2011	6.64	2.53	60	6.64	2.53	57	8.7%	0.00 [-0.92, 0.92]
Sawatzky 2014	5.4	1.4	8	5.3	1	7	7.6%	0.10 [-1.12, 1.32]
**Total (95% CI)**			**776**			**753**	**100.0%**	**-1.67 [-2.31, -1.03]**

Heterogeneity: Tau² = 1.02; Chi² = 86.59, df = 17 (P < 0.00001); I² = 80%
Test for overall effect: Z = 5.10 (P < 0.00001)

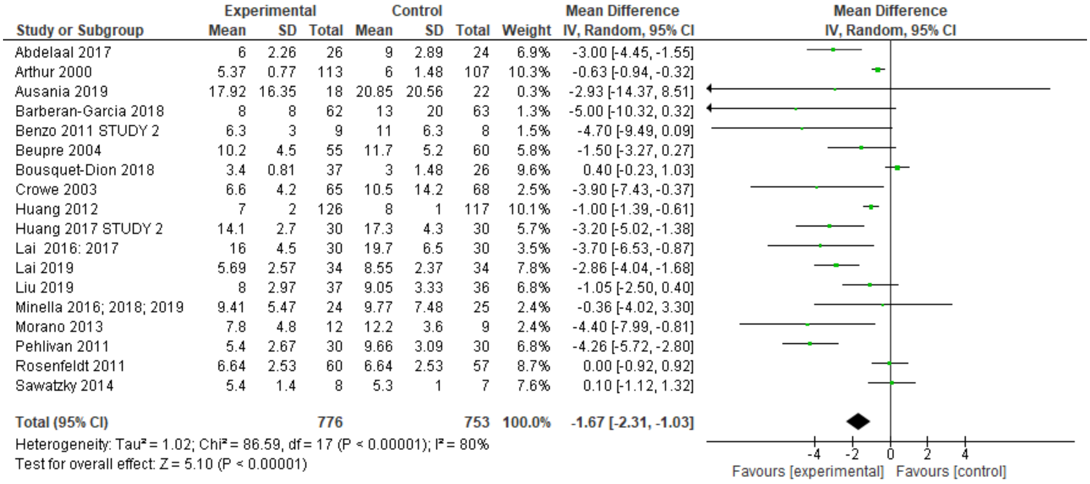

18/25 RCTs (1529 participants).

Figure 56. Random effects meta-analysis of the risk ratio of pneumonia between multimodal interventions (experimental) and usual care (control).

Study or Subgroup	Experimental Events	Experimental Total	Control Events	Control Total	Weight	Risk Ratio M-H, Random, 95% CI
Abdelaal 2017	2	26	0	24	5.3%	4.63 [0.23, 91.81]
Benzo 2011 STUDY 2	1	9	2	8	9.7%	0.44 [0.05, 4.02]
Huang 2017 STUDY 2	4	30	7	30	37.5%	0.57 [0.19, 1.75]
Lai 2016; 2017	4	30	7	30	37.5%	0.57 [0.19, 1.75]
Liu 2019	0	37	1	36	4.7%	0.32 [0.01, 7.71]
Morano 2013	0	12	2	9	5.5%	0.15 [0.01, 2.86]
Pehlivan 2011	0	30	0	30		Not estimable
**Total (95% CI)**		**174**		**167**	**100.0%**	**0.56 [0.28, 1.12]**
Total events	11		19			

Heterogeneity: Tau² = 0.00; Chi² = 2.84, df = 5 (P = 0.72); I² = 0%
Test for overall effect: Z = 1.64 (P = 0.10)

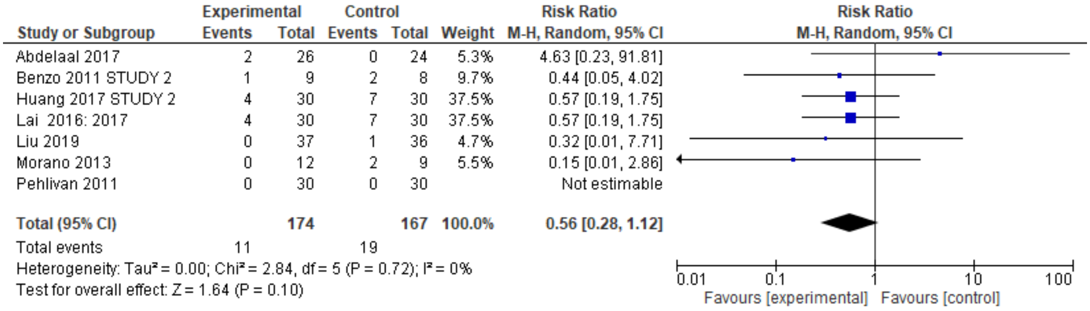

7/25 studies (341 participants).

Figure 57. Random effects meta-analysis of the risk ratio of total PO complications between multimodal interventions (experimental) and usual care (control).

Study or Subgroup	Experimental Events	Experimental Total	Control Events	Control Total	Weight	Risk Ratio M-H, Random, 95% CI
Allen 2019	11	22	17	23	9.6%	0.68 [0.42, 1.10]
Ausania 2019	6	18	12	22	3.9%	0.61 [0.29, 1.30]
Bousquet-Dion 2018	14	37	8	26	4.5%	1.23 [0.61, 2.50]
Minella 2018	14	24	18	25	12.9%	0.81 [0.53, 1.23]
Ommundsen 2018	40	53	55	63	69.1%	0.86 [0.72, 1.04]
**Total (95% CI)**		**154**		**159**	**100.0%**	**0.84 [0.72, 0.97]**
Total events	85		110			

Heterogeneity: Tau² = 0.00; Chi² = 2.70, df = 4 (P = 0.61); I² = 0%
Test for overall effect: Z = 2.30 (P = 0.02)

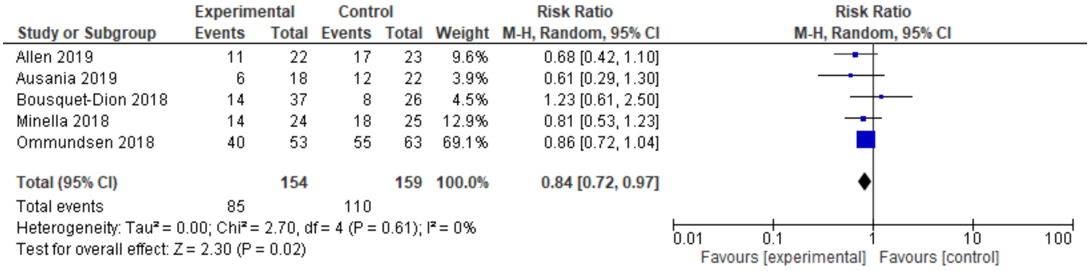

5/20 studies (313 participants). 1 abstract (Allen et al., 2019) included.

Sensitivity analyses (removing studies at high risk of bias)

Figure 58. Random effects meta-analysis of the risk ratio of mortality between multimodal (experimental) and usual care (control) with high risk of bias studies removed.

	Experimental		Control			Risk Ratio
Study or Subgroup	Events	Total	Events	Total	Weight	M-H, Random, 95% CI
Barberan-Garcia 2018	1	62	1	62	19.4%	1.00 [0.06, 15.63]
Beupre 2004	2	65	0	66	16.1%	5.08 [0.25, 103.73]
Minella 2018	0	24	2	25	16.5%	0.21 [0.01, 4.12]
Ommundsen 2018	2	53	3	53	48.0%	0.67 [0.12, 3.83]
**Total (95% CI)**		**204**		**206**	**100.0%**	**0.83 [0.25, 2.77]**
Total events	5		6			

Heterogeneity: Tau² = 0.00; Chi² = 2.29, df = 3 (P = 0.51); I² = 0%
Test for overall effect: Z = 0.31 (P = 0.76)

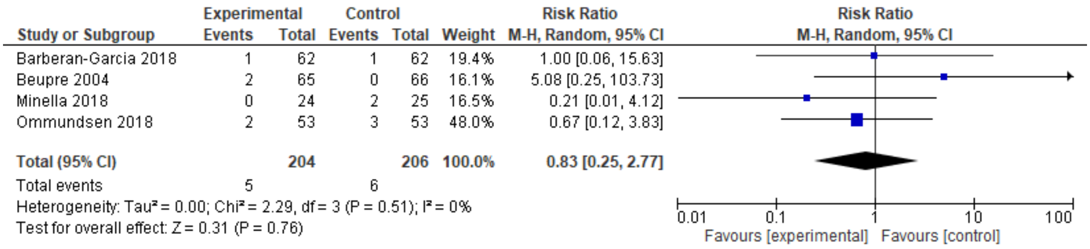

4 studies (410 participants) and 6 studies excluded (Ausania et al., 2019, Lai et al., 2016, Pehlivan et al., 2011,, Huang et al., 2017; Lai et al., 2019; Liu et al., 2019)

Figure 59. Random effects meta-analysis of the mean difference in length of stay between multimodal interventions (experimental) and usual care (control) with high risk of bias studies removed.

	Experimental			Control				Mean Difference
Study or Subgroup	Mean	SD	Total	Mean	SD	Total	Weight	IV, Random, 95% CI
Arthur 2000	5.37	0.77	113	6	1.48	107	47.2%	-0.63 [-0.94, -0.32]
Barberan-Garcia 2018	8	8	62	13	20	63	5.6%	-5.00 [-10.32, 0.32]
Beupre 2004	10.2	4.5	55	11.7	5.2	60	26.1%	-1.50 [-3.27, 0.27]
Minella 2016; 2018; 2019	9.41	5.47	24	9.77	7.48	25	10.4%	-0.36 [-4.02, 3.30]
Morano 2013	7.8	4.8	12	12.2	3.6	9	10.7%	-4.40 [-7.99, -0.81]
**Total (95% CI)**			**266**			**264**	**100.0%**	**-1.48 [-2.81, -0.14]**

Heterogeneity: Tau² = 0.96; Chi² = 7.58, df = 4 (P = 0.11); I² = 47%
Test for overall effect: Z = 2.17 (P = 0.03)

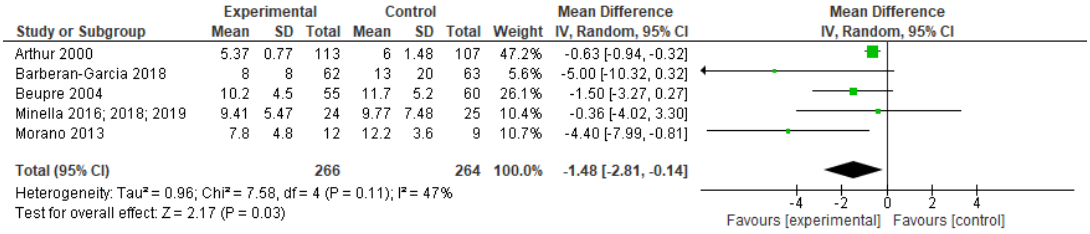

5 studies (530 partcipants) and 14 studies study excluded (Abdelaal et al., 2017, Ausania et al., 2019, Benzo et al., 2011, Huagne et al., 2012, Lai et al., 2017, Pehlivan et al., 2011,; Rosenfelt et al., 2011; Sawatzky et al., 2014; Lai et al., 2019; Liu et al., 2019; Morano et al., 2013).

Perry R, *et al. BMJ Open* 2022; 11:e050806. doi: 10.1136/bmjopen-2021-050806

Sensitivity analyses (removing studies with imputed results)

Figure 60. Random effects meta-analysis of the mean difference in length of stay between multimodal interventions (experimental) and usual care (control) with imputed results removed.

	Experimental			Control				Mean Difference
Study or Subgroup	Mean	SD	Total	Mean	SD	Total	Weight	IV, Random, 95% CI
Barberan-Garcia 2018	8	8	62	13	20	63	3.9%	-5.00 [-10.32, 0.32]
Benzo 2011 STUDY 2	6.3	3	9	11	6.3	8	4.5%	-4.70 [-9.49, 0.09]
Beupre 2004	10.2	4.5	55	11.7	5.2	60	12.4%	-1.50 [-3.27, 0.27]
Crowe 2003	6.6	4.2	65	10.5	14.2	68	6.8%	-3.90 [-7.43, -0.37]
Huang 2012	7	2	126	8	1	117	16.8%	-1.00 [-1.39, -0.61]
Huang 2017 STUDY 2	14.1	2.7	30	17.3	4.3	30	12.2%	-3.20 [-5.02, -1.38]
Lai 2016: 2017	16	4.5	30	19.7	6.5	30	8.7%	-3.70 [-6.53, -0.87]
Morano 2013	7.8	4.8	12	12.2	3.6	9	6.7%	-4.40 [-7.99, -0.81]
Pehlivan 2011	5.4	2.67	30	9.66	3.09	30	13.6%	-4.26 [-5.72, -2.80]
Sawatzky 2014	5.4	1.4	8	5.3	1	7	14.5%	0.10 [-1.12, 1.32]
**Total (95% CI)**			**427**			**422**	**100.0%**	**-2.59 [-3.78, -1.41]**

Heterogeneity: Tau² = 2.15; Chi² = 38.79, df = 9 (P < 0.0001); I² = 77%
Test for overall effect: Z = 4.28 (P < 0.0001)

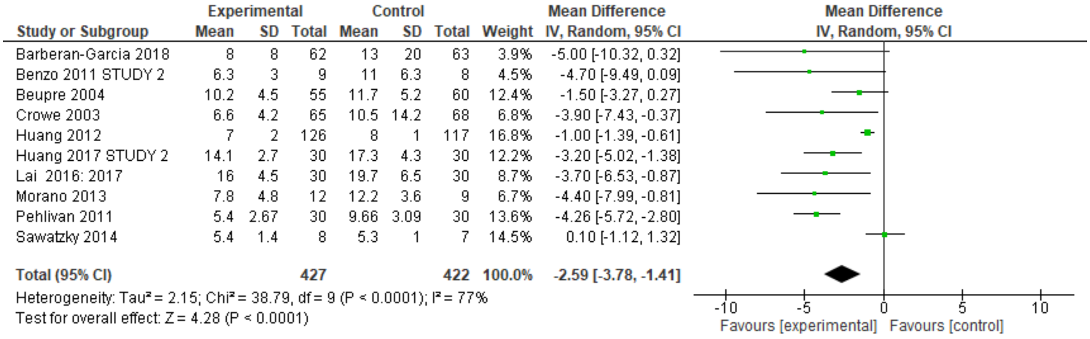

10 studies (849 participants) included and 8 studies excluded (Abdelaal et al., 2017, Arthur et al., 2000, Ausania et al., 2019, Bousquet-Dion et al., 2018, Minnella et al., 2018, Rosenfeldt et al., 2011).

Subgroup analyses (type of surgery)

ORTHOPAEDIC SURGERY

Figure 61. Random effects meta-analysis of the mean difference in length of hospital stay (LoS) between multimodal (experimental) and usual care (control) for those undergoing orthopaedic surgery.

	Experimental			Control				Mean Difference
Study or Subgroup	Mean	SD	Total	Mean	SD	Total	Weight	IV, Random, 95% CI
Beupre 2004	10.2	4.5	55	11.7	6.3	8	8.7%	-1.50 [-6.02, 3.02]
Crowe 2003	6.6	4.2	65	10.5	14.2	68	13.4%	-3.90 [-7.43, -0.37]
Huang 2012	7	2	126	8	1	117	78.0%	-1.00 [-1.39, -0.61]
**Total (95% CI)**			**246**			**193**	**100.0%**	**-1.43 [-2.84, -0.02]**
Heterogeneity: Tau² = 0.62; Chi² = 2.61, df = 2 (P = 0.27); I² = 23%								
Test for overall effect: Z = 1.99 (P = 0.05)								

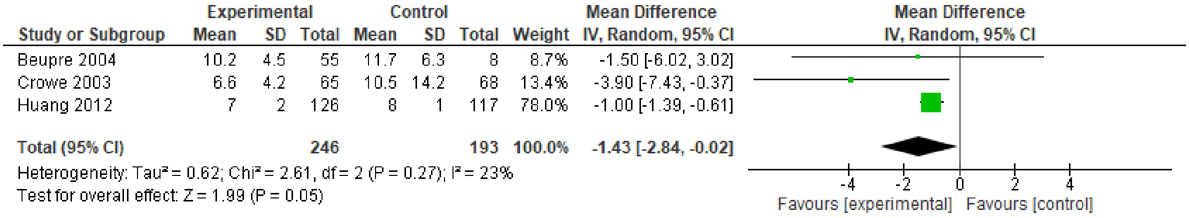

3 studies (439 participants) included and 15 studies excluded (Abdelaal et al., 2017, Arthur et al., 2000, Ausania et al., 2019, Barberan-Garcia et al., 2019, Benzo et al., 2011, Bousquet-Dion et al., 2018, Huang et al., 2017, Lai et al., 2017, Lai et al., 2019, Liu et al., 2019, Minella et al., 2019, Morano et al., 2013, Pehlivan et al., 2011, Rosnfeldt et al., 2011, Sawatzky et al., 2014).

Subgroup analyses (Cancer surgery)

Figure 62. Random effects meta-analysis of the risk ratio of mortality between multimodal (experimental) and usual care (control) for those undergoing cancer surgery.

	Experimental		Control			Risk Ratio
Study or Subgroup	Events	Total	Events	Total	Weight	M-H, Fixed, 95% CI
Ausania 2019	0	18	0	22		Not estimable
Huang 2017 STUDY 2	0	30	1	30	17.7%	0.33 [0.01, 7.87]
Lai 2016; 2017	0	30	1	30	17.7%	0.33 [0.01, 7.87]
Lai 2019	0	34	0	34		Not estimable
Liu 2019	0	37	0	36		Not estimable
Minella 2016; 2018; 2019	0	24	2	25	29.0%	0.21 [0.01, 4.12]
Ommundsen 2018	2	53	3	53	35.5%	0.67 [0.12, 3.83]
Pehlivan 2011	0	30	0	30		Not estimable
**Total (95% CI)**		**256**		**260**	**100.0%**	**0.42 [0.12, 1.41]**
Total events	2		7			
Heterogeneity: Chi² = 0.52, df = 3 (P = 0.91); I² = 0%						
Test for overall effect: Z = 1.41 (P = 0.16)						

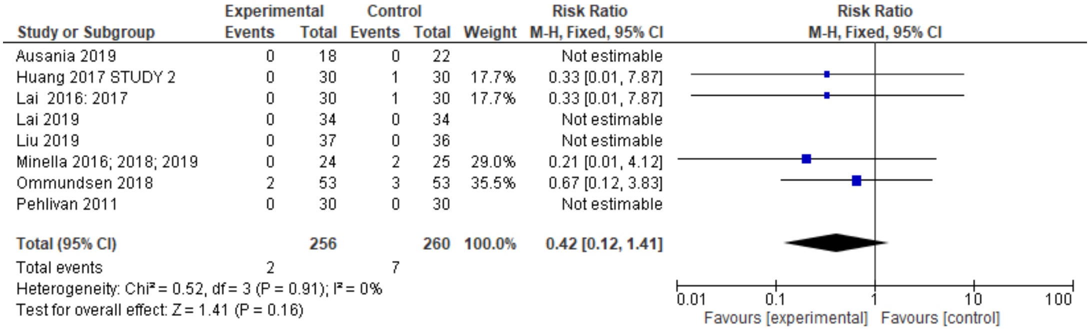

8 studies (516 participants) included and 10 studies excluded (Abdelaal et al., 2017, Arthur et al., 2000, Barberan-Garcia et al., 2019, Benzo et al., 2011, Bousquet-Dion et al., 2018, Crowe et al., 2003, Huang et al., 2012, Lai et al., 2017 Morano et al., 2013, Rosenfeldt et al., 2011, Sawatzky et al., 2014).

.

Figure 63. Random effects meta-analysis of the mean difference in length of hospital stay (LoS) between multimodal interventions (experimental) and usual care (control) for those undergoing cancer surgery.

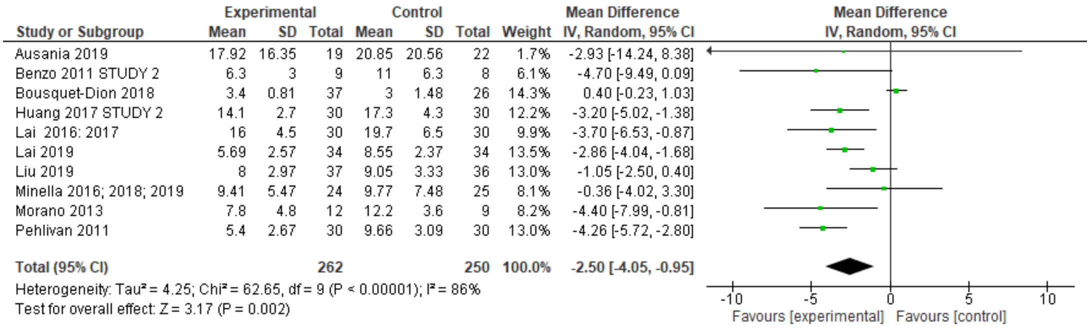

10 studies (512 participants) included and 8 studies excluded (Abdelaal et al., 2017; Arthur et al., 2000; Barberan-Garcia et al., 2019; Crowe et al., 2003; Huang et al., 2012; Morano et al., 2013; Rosenfeldt et al., 2011; Sawatzky et al., 2014).

Subgroup analysis (studies published before and after 2010)

A subgroup analysis for pneumonia and total postoperative complications could not be performed as the studies reporting these outcomes were all conducted after 2010.

Figure 64. Random effects meta-analysis of the risk ratio of mortality between multimodal (experimental) and usual care (control) in studies published after 2010.

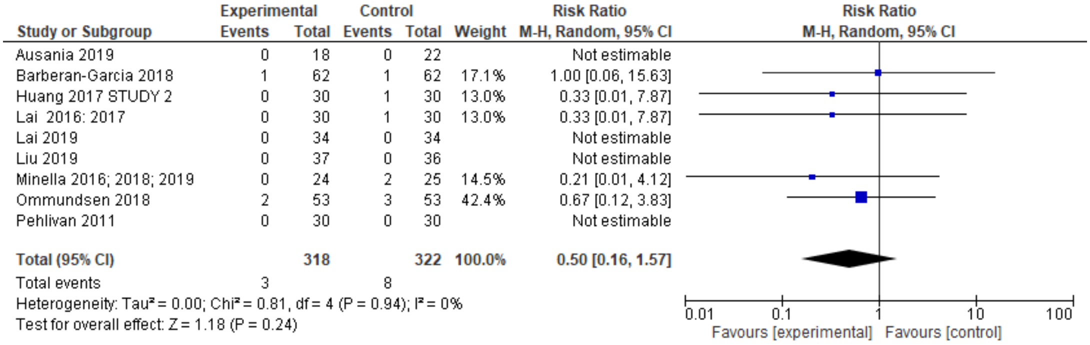

9 studies (640 participants) incuded and 1 study was excluded (Beupre et al., 2004).

Figure 65. Random effects meta-analysis of the mean difference in length of hospital stay (LoS) between multimodal (experimental) and usual care (control) in studies published after 2010.

	Experimental			Control				Mean Difference
Study or Subgroup	Mean	SD	Total	Mean	SD	Total	Weight	IV, Random, 95% CI
Abdelaal 2017	6	2.26	26	9	2.89	24	8.5%	-3.00 [-4.45, -1.55]
Ausania 2019	17.92	16.35	18	20.85	20.56	22	0.5%	-2.93 [-14.37, 8.51]
Barberan-Garcia 2018	8	8	62	13	20	63	2.1%	-5.00 [-10.32, 0.32]
Benzo 2011 STUDY 2	6.3	3	9	11	6.3	8	2.5%	-4.70 [-9.49, 0.09]
Bousquet-Dion 2018	3.4	0.81	37	3	1.48	26	10.6%	0.40 [-0.23, 1.03]
Huang 2012	7	2	126	8	1	117	10.9%	-1.00 [-1.39, -0.61]
Huang 2017 STUDY 2	14.1	2.7	30	17.3	4.3	30	7.4%	-3.20 [-5.02, -1.38]
Lai 2016; 2017	16	4.5	30	19.7	6.5	30	5.0%	-3.70 [-6.53, -0.87]
Lai 2019	5.89	2.57	34	8.55	2.37	34	9.2%	-2.66 [-3.84, -1.48]
Liu 2019	8	2.97	37	9.05	3.33	36	8.5%	-1.05 [-2.50, 0.40]
Minella 2016; 2018; 2019	9.41	5.47	24	9.77	7.48	25	3.7%	-0.36 [-4.02, 3.30]
Morano 2013	7.8	4.8	12	12.2	3.6	9	3.7%	-4.40 [-7.99, -0.81]
Pehlivan 2011	5.4	2.67	30	9.66	3.09	30	8.4%	-4.26 [-5.72, -2.80]
Rosenfeldt 2011	6.64	2.53	60	6.64	2.53	57	9.9%	0.00 [-0.92, 0.92]
Sawatzky 2014	5.4	1.4	8	5.3	1	7	9.1%	0.10 [-1.12, 1.32]
**Total (95% CI)**			**543**			**518**	**100.0%**	**-1.84 [-2.69, -0.99]**

Heterogeneity: Tau² = 1.69; Chi² = 78.29, df = 14 (P < 0.00001); I² = 82%
Test for overall effect: Z = 4.24 (P < 0.0001)

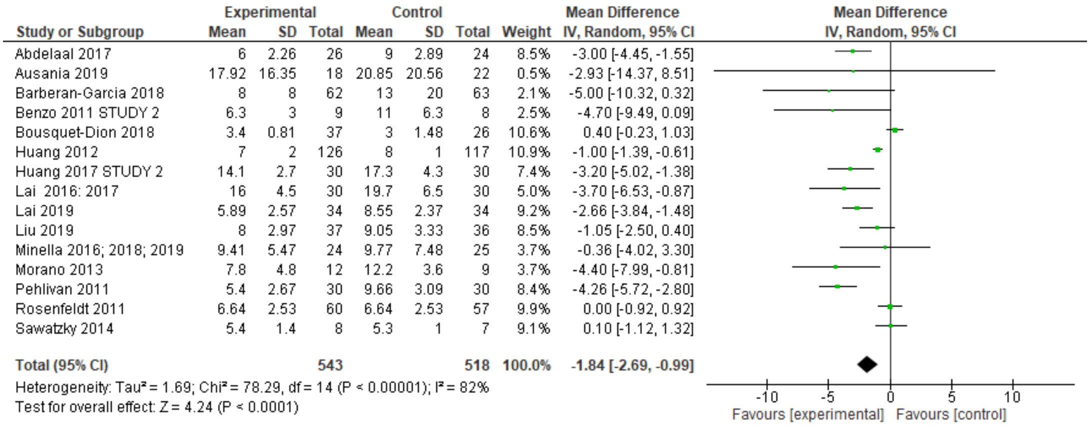

15 studies (1061 participants) included and 3 studies excluded (Beupre et al., 2004; Crowe et al., 2003; Arthur et al, 2000).

Figure 66. Random effects meta-analysis of the mean difference in length of hospital stay (LoS) between inspiratory muscle training (experimental) and usual care (control) in studies published before 2010.

	Experimental			Control				Mean Difference
Study or Subgroup	Mean	SD	Total	Mean	SD	Total	Weight	IV, Random, 95% CI
Arthur 2000	5.37	0.77	113	6	1.48	107	59.8%	-0.63 [-0.94, -0.32]
Beupre 2004	10.2	4.5	55	11.7	5.2	60	28.9%	-1.50 [-3.27, 0.27]
Crowe 2003	6.6	4.2	65	10.5	14.2	68	11.2%	-3.90 [-7.43, -0.37]
**Total (95% CI)**			**233**			**235**	**100.0%**	**-1.25 [-2.55, 0.06]**

Heterogeneity: Tau² = 0.72; Chi² = 4.12, df = 2 (P = 0.13); I² = 51%
Test for overall effect: Z = 1.87 (P = 0.06)

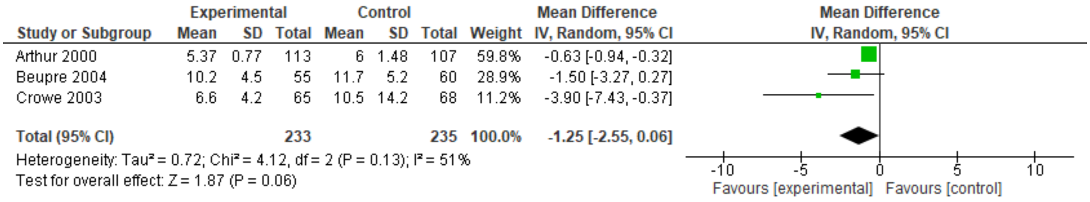

3 studies (468 participants) included and 15 studies excluded (Abdelaal et al., 2017; Ausania et al., 2019; Barberan-Garcia et al., 2018; Benzo et al., 2011; Bousquet-Dion et al., 2018; Huang et al., 2012; Huang et al 2017, Lai et al 2017, Lai 201; Liu et al 2019. Minella et al., 2016; Morano et al., 2013; Pehlivan et al., 2011; Rosenfeldt et al., 2011;Sawatzky et al., 2014).

References

Abdelaal, G., Eldahdouh, S., Abdelsamie, M. & Labeeb, A. 2017. Effect of preoperative physical and respiratory therapy on postoperative pulmonary functions and complications after laparoscopic upper abdominal surgery in obese patients. *Egyptian Journal of Chest Diseases and Tuberculosis,* 66.

Allen, S. K., Brown, V., White, D., King, D., Hunt, J., Prabhu, P., . . . Sultan, J. 2019. P185 MULTI-MODAL PREHABILITATION DURING NEOADJUVANT THERAPY PRIOR TO RESECTION FOR OESOPHAGOGASTRIC CANCER: A PILOT RANDOMISED CONTROLLED TRIAL. *Diseases of the Esophagus,* 32.

Arthur, H. M., Daniels, C., Mckelvie, R., Hirsh, J. & Rush, B. 2000. Effect of a preoperative intervention on preoperative and postoperative outcomes in low-risk patients awaiting elective coronary artery bypass graft surgery. A randomized, controlled trial. *Ann Intern Med,* 133, 253-62.

Ausania, F., Senra, P., Meléndez, R., Caballeiro, R., Ouviña, R. & Casal-Núñez, E. 2019. Prehabilitation in patients undergoing pancreaticoduodenectomy: a randomized controlled trial. *Rev Esp Enferm Dig,* 111, 603-608.

Barberan-Garcia, A., Ubré, M., Roca, J., Lacy, A. M., Burgos, F., Risco, R., . . . Martínez-Pallí, G. 2018. Personalised Prehabilitation in High-risk Patients Undergoing Elective Major Abdominal Surgery: A Randomized Blinded Controlled Trial. *Ann Surg,* 267, 50-56.

Beaupre, L. A., Lier, D., Davies, D. M. & Johnston, D. B. 2004. The effect of a preoperative exercise and education program on functional recovery, health related quality of life, and health service utilization following primary total knee arthroplasty. *J Rheumatol,* 31, 1166-73.

Benzo, R., Wigle, D., Novotny, P., Wetzstein, M., Nichols, F., Shen, R. K., . . . Deschamps, C. 2011. Preoperative pulmonary rehabilitation before lung cancer resection: results from two randomized studies. *Lung Cancer,* 74, 441-5.

Bousquet-Dion, G., Awasthi, R., Loiselle, S., Minnella, E. M., Agnihotram, R. V., Bergdahl, A., . . . Scheede-Bergdahl, C. 2018. Evaluation of supervised multimodal prehabilitation programme in cancer patients undergoing colorectal resection: a randomized control trial. *Acta Oncol,* 57, 849-859.

Crowe, J. & Henderson, J. 2003. Pre-arthroplasty rehabilitation is effective in reducing hospital stay. *Can J Occup Ther,* 70, 88-96.

Demark-Wahnefried, W., Nix, J. W., Hunter, G. R., Rais-Bahrami, S., Desmond, R. A., Chacko, B., . . . Grizzle, W. E. 2016. Feasibility outcomes of a presurgical randomized controlled trial exploring the impact of caloric restriction and increased physical activity versus a wait-list control on tumor characteristics and circulating biomarkers in men electing prostatectomy for prostate cancer. *BMC Cancer,* 16, 61.

Huang, J., Lai, Y., Zhou, X., Li, S., Su, J., Yang, M. & Che, G. 2017. Short-term high-intensity rehabilitation in radically treated lung cancer: a three-armed randomized controlled trial. *J Thorac Dis,* 9, 1919-1929.

Huang, S. W., Chen, P. H. & Chou, Y. H. 2012. Effects of a preoperative simplified home rehabilitation education program on length of stay of total knee arthroplasty patients. *Orthop Traumatol Surg Res,* 98, 259-64.

Kassouf, W., Minnella, E., Awasthi, R., Ferreira, V., Aprikian, A., Tanguay, S. & Carli, F. 2018. Prehabilitation for patients undergoing cystectomy: Preliminary analysis of a single-center, randomized controlled trial. *Journal of Urology,* 199 (4 Supplement 1), e622.

Kasvis, P., Bui, T., Kilgour, R., Carli, F. & Vignali, A. 2018. A multimodal prehabilitation program in hepato-pancreato-biliary cancer patients awaiting surgery: preliminary results. *MASCC/ISOO Annual Meeting on Supportive Care in Cancer.*

Lai, Y., Huang, J., Yang, M., Su, J., Liu, J. & Che, G. 2017. Seven-day intensive preoperative rehabilitation for elderly patients with lung cancer: a randomized controlled trial. *J Surg Res,* 209, 30-36.

Lai, Y., Su, J., Yang, M., Zhou, K. & Che, G. 2016. Impact and Effect of Preoperative Short-term Pulmonary Rehabilitation Training on Lung Cancer Patients with Mild to Moderate Chronic Obstructive Pulmonary Disease: A Randomized Trial]. *Zhongguo Fei Ai Za Zhi,* 19, 746-753.

Lai, Y., Wang, X., Zhou, K., Su, J. & Che, G. 2019. Impact of one-week preoperative physical training on clinical outcomes of surgical lung cancer patients with limited lung function: A randomized trial. *Annals of Translational Medicine,* 7 (20) (no pagination).

Liu, Z., Qiu, T., Pei, L., Zhang, Y., Xu, L., Cui, Y., . . . Huang, Y. 2019. Two-Week Multimodal Prehabilitation Program Improves Perioperative Functional Capability in Patients Undergoing

Thoracoscopic Lobectomy for Lung Cancer: A Randomized Controlled Trial. *Anesthesia and analgesia.,* 23.

Minnella, E. M., Awasthi, R., Bousquet-Dion, G., Ferreira, V., Austin, B., Audi, C., . . . Kassouf, W. 2019. Multimodal Prehabilitation to Enhance Functional Capacity Following Radical Cystectomy: A Randomized Controlled Trial. *Eur Urol Focus*.

Minnella, E. M., Awasthi, R., Gillis, C., Fiore, J. F., Jr., Liberman, A. S., Charlebois, P., . . . Carli, F. 2016. Patients with poor baseline walking capacity are most likely to improve their functional status with multimodal prehabilitation. *Surgery,* 160**,** 1070-1079.

Minnella, E. M., Awasthi, R., Loiselle, S. E., Agnihotram, R. V., Ferri, L. E. & Carli, F. 2018. Effect of Exercise and Nutrition Prehabilitation on Functional Capacity in Esophagogastric Cancer Surgery: A Randomized Clinical Trial. *JAMA Surg,* 153**,** 1081-1089.

Morano, M. T., Araujo, A. S., Nascimento, F. B., Da Silva, G. F., Mesquita, R., Pinto, J. S., . . . Pereira, E. D. 2013. Preoperative pulmonary rehabilitation versus chest physical therapy in patients undergoing lung cancer resection: a pilot randomized controlled trial. *Arch Phys Med Rehabil,* 94**,** 53-8.

Ommundsen, N., Wyller, T. B., Nesbakken, A., Bakka, A. O., Jordhøy, M. S., Skovlund, E. & Rostoft, S. 2018. Preoperative geriatric assessment and tailored interventions in frail older patients with colorectal cancer: a randomized controlled trial. *Colorectal Dis,* 20**,** 16-25.

Pehlivan, E., Turna, A., Gurses, A. & Gurses, H. N. 2011. The effects of preoperative short-term intense physical therapy in lung cancer patients: a randomized controlled trial. *Ann Thorac Cardiovasc Surg,* 17**,** 461-8.

Rosenfeldt, F., Braun, L., Spitzer, O., Bradley, S., Shepherd, J., Bailey, M., . . . Esmore, D. 2011. Physical conditioning and mental stress reduction--a randomised trial in patients undergoing cardiac surgery. *BMC Complement Altern Med,* 11**,** 20.

Sawatzky, J. A., Kehler, D. S., Ready, A. E., Lerner, N., Boreskie, S., Lamont, D., . . . Duhamel, T. A. 2014. Prehabilitation program for elective coronary artery bypass graft surgery patients: a pilot randomized controlled study. *Clin Rehabil,* 28**,** 648-57.

Vagvolgyi, A., Rozgonyi, Z., Kerti, M., Agathou, G., Vadasz, P. & Varga, J. 2018. Effectiveness of pulmonary rehabilitation and correlations in between functional parameters, extent of thoracic surgery and severity of post-operative complications: randomized clinical trial. *Journal of Thoracic Disease,* 10**,** 3519-+.

Wang, X., Che, G. & Liu, L. 2017. A short-term high-intensive pattern of preoperative rehabilitation better suits surgical lung cancer patients. *Interactive Cardiovascular and Thoracic Surgery,* 25 (Supplement 1)**,** i11.

EDUCATIONAL INTERVENTIONS

Table 21. Characteristics of studies

	1st Author, year and country	Total *n* randomized Intervention (I):Control (C)<br><br>Number analysed (An)if reported	Patient population, baseline clinical characteristics (mean (SD) or *n* unless otherwise stated	Demographics (mean (SD) or *n* unless otherwise stated)	Intervention	Comparator	Mode of delivery; place of delivery; training level of individuals who delivered the intervention; the number of contacts	Intervention fidelity; Compliance or adherence to intervention
**EDUCATIONAL INTERVENTIONS**								
1	Boden et al., 2018<br>Australia	441 ppts scheduled for abdominal surgery<br>I=219:C=222<br><br>432 completed trial	**Functional comorbidity index [median (IQR)]**<br>I=2 (1-5)<br>C=2 (1-4)<br>**ASA physical health status**<br>**1-2**<br>I=69%:C=58%<br>**3-4**<br>I=31%:C=42%	**Age [median (IQR)]**<br>I=63.4 (51.5-71.9)<br>C= 67.5 (56.3-75.3)<br>**Gender (% M)**<br>I=61:C=61	Standardised physiotherapy assessment (physical and subjective) and booklet with information about PPCs and post-op breathing exercises plus additional single 30 min education and breathing exercise coaching session with physiotherapist to reinforce the booklet.	Standardised physiotherapy assessment and booklet only	Face to face;<br>Hospital;<br>Physiotherapist;<br>1 x 30 mins.	Intervention consisted of a single training session delivered in hospital.
2	Butler et al., 1996<br>Canada	123 ppts randomised but data only used for 80 who had NOT undergone at least 1 THR surgery (43 were excluded due to this)<br>I=32:C=48	NR	**Age yrs**<br>I=63.86 (13.08)<br>C=61.83 (12.86)<br>**Gender (%M)**<br>I=44:C=54	Total hip replacement: a patient's guidebook added to preadmission information pack 4-6 wks prior to surgery. Patients were not told to study the booklet and did not know it was being evaluated.	Usual care; After admission (typically the day before surgery) both groups received education that paralleled the information in the booklet so by discharge all had been exposed to the same information	Self-delivery;<br>Home;<br>None;<br>None	NR;<br>26% reported reading booklet once, 39% read it 2-3 times, 35% 4+ times.

Perry R, *et al. BMJ Open* 2022; 11:e050806. doi: 10.1136/bmjopen-2021-050806

	1st Author, year and country	Total *n* randomized Intervention (I):Control (C)<br><br>Number analysed (An)if reported	Patient population, baseline clinical characteristics (mean (SD) or *n* unless otherwise stated	Demographics (mean (SD) or *n* unless otherwise stated)	Intervention	Comparator	Mode of delivery; place of delivery; training level of individuals who delivered the intervention; the number of contacts	Intervention fidelity; Compliance or adherence to intervention
**EDUCATIONAL INTERVENTIONS**								
3	Cooke et al., 2016<br>Australia<br>Pilot study	91 ppts undergoing hip or knee replacement surgery (THA or TKA)<br>I=45 (40An)<br>C=46 (42An)		**Overall Age yrs**<br>Median (range)<br>67 (36-86)<br>**Overall Gender (%M)**<br>37<br>I:C "profile similar"	Self-efficacy-based education sessions of 20-30 mins delivered via DVD.<br>Ppts were asked to review the DVD within 72hrs of receiving it at the pre-op session and work through activities at home 4x before admission.<br>A research nurse phoned ppts 72 hrs after pre-op session and again in 2 to 3 wks.	Usual care	Self-delivery;<br>Home;<br>Registered nurse;<br>2	NR;<br>Self reported: 6.3% (writing goals down) to 78.1% (setting goals before surgery) engaged in some goal setting and 9.7% (meditation) to 68.8% (listening to music) with suggested relaxation strategies. More criteria reported in paper.
4	Eschalier et al., 2017<br>USA	42 ppts undergoing TKA<br>I=22:C=20	**Preoperative PA** (yes/no)<br>I=9/12<br>C=14/6<br>**BMI Kg/m^2**<br>I=31.2 (5.1)<br>C=31.6 (5.4)<br>**Function (WOMAC score)**<br>I=41.9 (13.7)<br>C=40.7 (17.9)	**Age yrs**<br>I=68.1 (4.7)<br>C=66.8 (5.8)<br>**Gender (%M)**<br>I=54.5:C=50	Standard information delivered orally by the surgeon plus standardized information booklet given just after preanesthesia visit, i.e., 4±6 wks before TKA. Ppts asked to read the booklet carefully several times.	Standard information delivered orally by the surgeon.	Written;<br>Outpatient clinic;<br>Nurse;<br>One-off intervention	NR;<br>NR
5	Giraudet-Le Quintrec et al., 2003<br>France	100 ppts undergoing THA<br>I=48:C=52	**Height cm**<br>I=165.7 (17.4)<br>C=166.8 (10.0)<br>**Weight kg**<br>I=73.5 (13.7)<br>C=71.7 (14.1)	**Age yrs**<br>I=62.7 (8.8)<br>C=64.3 (9.5)<br>**Gender (%M)**<br>I=50:C=38	Usual verbal information and standard information leaflet plus standardised group education session with 3 to 6 ppts, 2-6 wks before surgery describing the surgery, what to expect and rehabilitation procedure. Half a day delivered by one surgeon and one anesthetist.	Usual verbal information from surgeon and anesthetist and the standard information leaflet.	Face-to-face, written;<br>Hospital;<br>Surgeon and anesthetists;<br>One-off intervention	NR;<br>NR
6	Johansson et al., 2007<br>Finland	123 ppts undergoing elective hip arthroplasty<br>I=62:C=61	**Previous surgery Revision operation n (%)**<br>53 (43%)<br>(I=38%:C=49%)	**Age yrs**<br>I=59.7:C=65.2<br>**Gender (%M)**<br>I=49:C=49	Usual written educational materials plus education using the concept map method (ppt and the nurse sit side by side and discuss ppt care-related issues identified by the patient	Usual written educational materials	Face to face, written;<br>NR;<br>Nurse (Specialized in surgical nursing + extensive experience in orthopaedic nursing. Nurses received special training	NR;<br>NR

	1st Author, year and country	Total *n* randomized Intervention (I):Control (C)<br><br>Number analysed (An)if reported	Patient population, baseline clinical characteristics (mean (SD) or *n* unless otherwise stated	Demographics (mean (SD) or *n* unless otherwise stated)	Intervention	Comparator	Mode of delivery; place of delivery; training level of individuals who delivered the intervention; the number of contacts	Intervention fidelity; Compliance or adherence to intervention
**EDUCATIONAL INTERVENTIONS**								
					and deemed to be relevant). In addition, the nurses used checklists to ensure all the relevant aspects were covered in the preadmission education.<br>2 wks prior to admission<br>1 session 30mins to 1 hr		1 x 30mins-1hr	
7	Kesänen et al., 2017<br>Finland	100 ppts wth spine stenosis udergoing surgery (decompression, fusion or both)<br>I=50:C=50	**Chronic diseases**<br>I=37:C=38<br>**Mental health problems**<br>I=1:C=2<br>**Anxiety (BL)**<br>I=44.0:C=41.9<br>**Previous spine surgery (yes/no)**<br>I=17/33: C=16/32<br>**Previous other surgery (yes/no)**<br>I=39/10: C=45/4	**Age yrs**<br>I=62 (12.5)<br>C=63 (12)<br>**Gender (%M)**<br>I=34:C=38	Knowledge Test Feedback Intervention (KTFI) in addition to usual care. KTFI consists of an educational telephone discourse that aims to support the patient´s cognitive empowerment through strengthening his/her knowledge of surgery-related issues. The telephone discourse consisted of three phases: i) creating an open atmosphere with small talk; ii) discourse session utilising open-ended questions and active listening; iii) summary of session. The mean duration of discourse was 21 min (range 8–65).	Routine preoperative education. The surgeon informed the ppt about the disease, differenceerent treatment options, the surgery, possible complications, and expected outcomes. A staff nurse gave instructions on how to prepare for surgery, and dealt with any possible concerns the ppt may have had. At admission to the hospital, the ppt met an anaesthesiologist and a physiotherapist.	Phone;<br>NR- assumed home;<br>Nurse;<br>NR	NR;<br>NR
8	Koyuncu et al., 2013<br>Turkey<br>(Abstract only)	37 ppts undergoing arthroscopic rotator cuff repair<br>I=17:C=20	NR	**Age (yrs) median, range)**<br>I=59 (43-76)<br>C=58.5 (46-78)	Preoperative education session on relaxation techniques, sling wear practice, postural exercises	No preoperative education	Self-delivery (assumed)<br>NR;<br>NR;<br>NR;	NR;<br>NR
9	Magdaleno et al., 2018<br>Spain	62 ppts undergoing	**ASA i/ii (%)**<br>I=52/55<br>C=55/45<br>**BMI kg/m^2**	**Age yrs**<br>I=44.5 (12)<br>C=49 (11)<br>**Gender (%M)**	Scheduled office visits to surgeon where surgical intervention was explained followed by preoperative	Office visits to surgeon where surgical intervention was explained.	Oral, written information;<br>Hospital outpatient setting;<br>Specialized nurse;	

	1st Author, year and country	Total *n* randomized Intervention (I):Control (C)<br><br>Number analysed (An)if reported	Patient population, baseline clinical characteristics (mean (SD) or *n* unless otherwise stated	Demographics (mean (SD) or *n* unless otherwise stated)	Intervention	Comparator	Mode of delivery; place of delivery; training level of individuals who delivered the intervention; the number of contacts	Intervention fidelity; Compliance or adherence to intervention
**EDUCATIONAL INTERVENTIONS**								
		major outpatient surgery (laproscopic cholecystectomy)<br>I=31:C=31	I=29.6 (5.5)<br>C=25.2 (3)	I=32:C=26	education - personalized oral and written information (informative brochure) of the entire surgical and anesthetic process from a specialized nurse.<br>15-30 days prior to surgery.		1 contact with nurse plus "visits to surgeon" for routine information	
10	McGregor et al., 2004<br>UK	35 ppts undergoing TKA<br>(All ppts received the same cemented prosthesis)<br><br>I=19:C=20	Exclusions for previous hip surgery	**Age yrs**<br>I=70.8(9.3):<br>C=72.8(10.1)<br>**Overall gender (%M)**<br>29	Attendance at a class 2-4 wks before surgery + info. booklet.<br><br>Info. booklet documented info. on the surgery and all preop and PO stages, rehabilitation stages including exercise regimes, and a series of answers to commonly asked questions regarding TKA. The preop class enforced this booklet and ensured that all ppts could do the exercises and understood how to use walking aids postoperatively. The class also ensured that ppts understood and could make provision for any adaptations required to homes for the immediate NRRPO recovery period.	Standard pathway of care:<br>this included a description of the surgery and its risks and approximations on LoS	Verbal, written;<br>Hospital setting + home;<br>NR;<br>NR	NR;<br>NR
11	Pazar and Iyigun, 2020<br>Turkey	205 ppts undergoing cardiac surgery<br><br>I=102 (100)<br>C=103 (100)	**Hypertension**<br>I=25(25%)<br>C=41(41%)<br>**COPD**<br>I=5(5%)<br>C=2(2%)<br>**Diabetes**<br>I=28(28%)<br>C=32(32%)<br>**BMI kg/m^2**	**Age yrs**<br>I=55.5 (17.3)<br>C=60.6 (15.1)<br>**Gender (%M)**<br>I=77:C=72	Preoperative education on mechanical ventilation (which was developed using data from semi-structured interview forms, opinions of medical doctors/nurses in ICU, and the literature). Plus a communication panel for patients to use for communicating with health	Usual care	Booklet (which was read and explained by the researchers);<br>Within the ppts' room in hospital;<br>Researchers;<br>1 contact (20-30 mins) the day before surgery	NR;<br>NR

	1st Author, year and country	Total *n* randomized Intervention (I):Control (C)<br><br>Number analysed (An)if reported	Patient population, baseline clinical characteristics (mean (SD) or *n* unless otherwise stated	Demographics (mean (SD) or *n* unless otherwise stated)	Intervention	Comparator	Mode of delivery; place of delivery; training level of individuals who delivered the intervention; the number of contacts	Intervention fidelity; Compliance or adherence to intervention
**EDUCATIONAL INTERVENTIONS**								
			**[median(IQR)]**<br>I=27.7(19.0–19.3)<br>C=27.2(18.0–18.3)		professionals whilst under mechanical ventilation			
12	Shuldham et al., 2002<br>UK	356 ppts undergoing CABG<br><br>I=188 (173 An)<br>C=168 (156 An)	**Urgency of illness:**<br>Very urgent<br>I=43(25%):C=32(20%)<br>Urgent<br>I=26(15%):C=15(10%)<br>Not urgent<br>I=103(59%):C=107(69%)<br>Not categorized<br>I=1(1%):C=2 (1%)	**Age yrs**<br>I=62.7(7.46)<br>C=62.3(8.46)<br>**Gender (M%)**<br>I= 90:C=85<br>**Ethnicity**<br>(White/Other/Not classified%)<br>I=84/13/3:<br>C=92/6/2	Each ppt attended the hospital once for approx. 4 h in a group of between 10 and 15 early in the waiting period. Relatives could choose to attend.<br>The education programme provided information on pre-operative events and likely progress. Factors personal to the ppt such as recovery and stay in ICU/recovery, pain and analgesia were discussed. Videos were used and a package of written information given to everyone. An optional visit to the wards and ICU was arranged.	The usual education provided, informally on a one-to-one basis by staff as part of the normal care which C and I groups received, started at the time of admission, anything from a few days to the day before surgery. Nurse, doctor, physiotherapist, occupational therapist, pharmacist and dietician all participated.<br>This individual teaching was supplemented by a regular series of sessions on the wards to which ppts were invited.	Verbal: videos and written materials;<br>Hospital;<br>Independent team of staff;<br>None	NR:<br>Adherence:<br>I=124 followed protocol<br>C=145 followed protocol
13<br>.	Soeters et al., 2018<br>USA	N=126<br>Unilateral TJA (THA or TKA)<br>I=63:C=63	**Operation**<br>THA; I=31:C=32<br>TKA: I=32:C=31<br>**BMI kg/m^2**<br>I=29(6):<br>C=29(6.1)<br>More men [(48%)] were randomized into the I group than into the C group [(19%)], P=0.013.	**Age yrs**<br>I=61(9):<br>C=62(8)<br>**Gender (%M)**<br>I=44:C=29<br><br>.	Group education class and a surgery-specific info. booklet (same as C group, delivered before randomisation) plus Preoperative Physical Therapy Education (PreopPTEd): a one-time, 1-on-1 session with a Physical Therapist (PT) to learn and practice PO precautions, exercises, bed mobility and ambulation with negotiation of stairs. Main diff between group class and this session was the opportunity to practice mobility and assistive devices before	Group education class and a surgery-specific info. booklet.	Face-to-face, 1-to1 plus access to online education;<br>Hospital;<br>Physical Therapist;<br>1 contact	NR;<br>NR<br><br>"All ppts reported utilizing the microsite at one point in the study period with 96%reportedly utilizing it preop and 76% reportedly using it postoperatively"

	1st Author, year and country	Total *n* randomized Intervention (I):Control (C)<br><br>Number analysed (An)if reported	Patient population, baseline clinical characteristics (mean (SD) or *n* unless otherwise stated	Demographics (mean (SD) or *n* unless otherwise stated)	Intervention	Comparator	Mode of delivery; place of delivery; training level of individuals who delivered the intervention; the number of contacts	Intervention fidelity; Compliance or adherence to intervention
**EDUCATIONAL INTERVENTIONS**								
					surgery. After this session, ppts given access to a lateralized, joint-specific microsite that provided detailed info. regarding exercises, transfers, ambulation, and Activities of daily living through videos, pictures and text.<br>PreopPTEd session took place approx. 2 wks before surgery and lasted 20-30 mins.			
14	Vukomanović et al., 2008 Serbia	45 ppts undergoing primary THR<br>I=23:C=22	**Yrs since 1st disorder [mean(sd); median(range)]**<br>I=8.05(5.9);6(1-25):<br>C=6.33(7.5);4(1-27)<br>**Mths with intensive pain [mean(sd); median(range)]**<br>I=20.1(14.3);12(6-60):<br>C=16.35(4.3);12(6-60)<br>**Use of analgesics (n, %)**<br>**Does not use**<br>I=6(30):C=4(20)<br>**Occasionally**<br>I=9(45):C=7(35)<br>**Permanently**<br>I=5(25):C=9(45)<br>**No. of other medications**	**Age [mean (sd); median, (range)]**<br>I=60.05(11.0);62.5 (30-70):<br>C=56.2(18.5);66.5 (19-70)<br>**Gender (%M)**<br>I=30:C=20	Usual rehab. care plus short-term intensive preop preparation through education about the procedure and instruction in basic activities from the PO rehab program e.g getting in and out of bed. Ppts were educated verbally and with a brochure. Unspecified duration	Usual rehab. care	Verbal;<br>NR but assumed hospital;<br>Experienced professionals;<br>1 appointment with the psychiatrist and 2 practical classes with a physiotherapist	NR;<br>NR

	**1st Author, year and country**	**Total *n* randomized Intervention (I):Control (C)<br><br>Number analysed (An)if reported**	**Patient population, baseline clinical characteristics (mean (SD) or *n* unless otherwise stated**	**Demographics (mean (SD) or *n* unless otherwise stated)**	**Intervention**	**Comparator**	**Mode of delivery; place of delivery; training level of individuals who delivered the intervention; the number of contacts**	**Intervention fidelity; Compliance or adherence to intervention**
**EDUCATIONAL INTERVENTIONS**								
			**[mean(sd); median(range)]**<br>I=2.6(2.4);2.5(0-6):<br>C=2.3(2);2.5(0-6)					
15	Watt-Watson et al., 2000 Canada	47 ppts undergoing elective first CABG surgery attending pre-admission clinic<br>I1=15<br>I2=16<br>C=16<br>Data on 45 ppts only as 2 in I1 were too ill to participate post surgery	NR	**Age yrs**<br>I1=64.2 (7)<br>I2=57.1 (9)<br>C=60.1 (11)<br>**Gender (%M)**<br>I1: 87: I2: 87.5<br>C :94	I1: study-specific booklet (*Pain relief after surgery),* instructions to read it before surgery and bring it to hospital + usual care<br><br>I2: As above + an interview with a research nurse who discussed points from the booklet and answered questions	Usual care (generic booklet + video 2-7 days before surgery)	I1: Written only<br>I2: Face to face, written;<br>NR- likely to be hospital clinic;<br>Research nurse;<br>1 contact, 2 – 7 days before surgery	NR;<br>NR
16	Watt-Watson et al., 2004 Canada	406 ppts undergoing elective first CABG surgery attending pre-admission education session<br>I=202 (198)<br>C=204 (192)	NR	**Age yrs**<br>I=61.7 (9)<br>C=61.9 (9)<br>**Gender (%M)**<br>I=87:C=83	Usual care + a study specific booklet (*Pain relief after surgery),* instructions to read it before surgery and bring it to hospital + an interview with a research nurse who discussed points from the booklet and answered questions	Usual care (generic booklet + video 2-7 days before surgery)	Face-to-face and written;<br>NR- likely to be hospital clinic;<br>Research nurse;<br>1 contact, 2–7 days before surgery	20% of I group said they hadn't received the booklet. Of the remainder, 88% said they had read all or most of it.

Perry R, *et al. BMJ Open* 2022; 11:e050806. doi: 10.1136/bmjopen-2021-050806

	1st Author, year and country	Total *n* randomized Intervention (I):Control (C)<br><br>Number analysed (An)if reported	Patient population, baseline clinical characteristics (mean (SD) or *n* unless otherwise stated	Demographics (mean (SD) or *n* unless otherwise stated)	Intervention	Comparator	Mode of delivery; place of delivery; training level of individuals who delivered the intervention; the number of contacts	Intervention fidelity; Compliance or adherence to intervention
**EDUCATIONAL INTERVENTIONS**								
17	Wilson, 2011; (Phd); 2016 Canada	143 ppts undergoing elective unilateral primary TKA<br>I=73:C=70<br><br>ITT on:<br>I=72:C=68	**Hypertension**<br>I=29(40%)<br>C=31(44%)<br>**Osteoarthritis**<br>I=70(96%)<br>C=67(96%)<br>**Rheumatoid arthritis**<br>I=3(4%)<br>C=3(4%)	**Age yrs**<br>I=66 (8):C=67 (8)<br>**Gender (%M)**<br>I=37:C=39	Usual care teaching plus: booklet specific to symptom management after TKA; individual teaching session during preadmission testing visit; telephone follow-up support call during the wk before surgery	Usual care teaching: educational session by physiotherapist; 30 min video explaining surgery and PO routines; brief review of patient-controlled anaesthesia. Written teaching material: pain after surgery; warfarin; plan of care for elective TKA	Face-to-face, written material, telephone call;<br>Clinic appointment and home (telephone call);<br>PhD student (an advanced practice nurse);<br>2 (face-to-face within 4 wk of surgery, telephone call during the wk before surgery)	NR;<br><br>Clinic visit (individual teaching session): 100% attendance<br><br>Booklet: 100% indicated they had read the booklet<br><br>Telephone call: 65/72 (90%) [surgery time change (n=4), unable to be reached (n=3)]

KEY: An=analysed; ASA=American Society of Anaesthesiologists; BL=baseline; C=control; CABG=coronary artery bypass graft; hr(s)=hour(s); I=intervention; ICU=Intensive care unit; LoS=length of stay; M=male; PhD=doctorate; min(s)=minutes; n or No.=number; NR=not reported; NRS=numerical rating scale; PA=physical activity; ppt=participants; PO=postoperative; pre-op=preoperative; PROMIS=Patient-Reported Outcomes Measurement Information System; THR=total hip replacement; TJA=total joint replacement; TKA=total knee arthroplasty; wk(s)=week(s); WOMAC=Western Ontario and McMaster Universities Osteoarthritis Index; yrs=years

Perry R, *et al. BMJ Open* 2022; 11:e050806. doi: 10.1136/bmjopen-2021-050806

EDUCATIONAL INTERVENTIONS

Table 22. Results

ID	Study	Total number of withdrawals (n)	Clinical outcomes(mean (SD) or n unless otherwise stated)	Intervention-specific outcomes [(n or mean (SD) unless otherwise reported)] and economic evaluations
1	Boden et al, 2018	9 withdrawn from trial<br>(I=5, ITT=218<br>C=4, ITT=214)	**Mortality In hospital**<br>I=3/218: C=3/214<br>**At 6wks**<br>I=4/218: C=3/214<br>**ICU LoS [days, median (IQR)]**<br>I= 1.3 (2-9): C=1.5 (2-7)<br>**LoS [days, median (IQR)] I=218:C=214**<br>I=8 (6-11): C=9 (7-13)<br>LoS days (mea/sd Quantile Estimation (QE) conversion method)<br>*I=8.95 (4.28):C=10.46 (5.29)*<br>**Hospital readmissions at 6 wks**<br>I=36/197: C=33/199<br>**All ppt reporting complications at 6 wks**<br>I=74/192: C=79/197<br>**Total Pulmonary complications (I=218: C=214)**<br>I=28/218: C=60/214, P<0.001<br>**Pneumonia**<br>I=18/218: C=42/214<br>**Pneumothorax**<br>I=11/218:C=8/214<br>**Pleural effusion**<br>I=10/218: C=11/214<br>**Pulmonary embolism**<br>I=3/218:C=4/214<br>**Acute respiratory failure**<br>I=5/218: C=12/214<br>**Infections**<br>**Wound**<br>I=22/218: C=25/214<br>**UTI**<br>I=12/218: C=18/214<br>**Sepsis**<br>I=8/218: C=14/214<br>**All others**<br>I=16/218: C=20/214<br>**Surgical complications (in hospital)**<br>**Surgical lacerations**<br>I=22/218: C=12/214	**Adverse events**<br>No adverse events were attributable to the preoperative physiotherapy education sessions or to the assisted ambulation protocol

ID	Study	Total number of withdrawals (n)	Clinical outcomes(mean (SD) or n unless otherwise stated)	Intervention-specific outcomes [(n or mean (SD) unless otherwise reported)] and economic evaluations
			**Haemorrhage**<br>I=16/218: C=13/214<br>**Wound dehiscence**<br>I=5/218: C=8/214<br>**Anastomosis leaks**<br>I=3/218: C=2/214<br>**Other events**<br>**Delirium**<br>I=17/218: C=22/214<br>**Re-intubation**<br>I=8/218: C=11/214<br>**Cardiac event**<br>I=11/218: C=7/214	
2	Butler et al., 1996	43 ppts were excluded after it was discovered they had had previous THR.	**Mortality I=30:C=40**<br>1 ppt died during study but unclear which group<br>**LoS days I=30:C=40**<br>I=10.3 (4.7): C=10.4 (5.5), NS<br>**QoL**<br>**Anxiety from admission to 1 day prior to discharge**<br>Less anxiety in booklet group: F (1.68)=7.81, P=0.007	**Outcomes measured: preparedness for returning home and practice of exercises**<br>No difference between groups in how they rated their overall preparedness for returning home.<br>Compared with C, ppts in the I group were significantly more likely to practice: breathing and coughing exercises (55% vs.15%, P=0.001); log rolling (39% vs. 6%); leg exercises (65% vs. 24%, P=0.001)<br>No difference between groups on satisfaction ratings
3	Cooke et al., 2016	9 (I=5:C=4) excluded after initial randomization (2 recruited in error, 5 had surgery cancelled, 1 withdrew from surgery, 1 withdrew from trial)<br><br>16 (8 in each arm) lost to FU and 1 in I didn't receive the DVD.	**LoS [Median (IQR)] I=40:C=42**<br>In hospital ranged from 3 to 27 days, the median was 5 days (IQR 4–6). NR whether I or C<br>**Pain NRS 0 (no pain) to 10 (worst pain possible) I=40:C=42**<br>**2 days PO**<br>I=4.2 (3.0): C=3.5 (2.3)<br>**10-14 days post discharge**<br>I=2.05 (2): C=2.8 (2.2)<br>**Health service use**<br>"Levels of health service use at 6 wks post discharge were similar for both groups"	**Outcomes reported: Self-efficacy (General Self Efficacy Scale), Anxiety (State-Trait Anxiety Inventory)**<br>Self-efficacy (General Self Efficacy Scale): increased for both groups from pre-hospital to 6 wks post discharge with no evidence for a difference between groups at any timepoint<br>Anxiety (State-Trait Anxiety Inventory): No evidence for a difference between groups at any timepoint
4	Eschalier et al., 2017	None	**LoS (days) I=22:C=20**<br>I=9.6 (2.8): C=8.8 (2.0), NS	**Outcomes reported: knowledge score post intervention, satisfaction with information received**<br>No difference between groups in knowledge score or satisfaction with the information received (both groups reported high satisfaction)
5	Giraudet-Le Quintrec et al, 2003	I=0<br>C=1 (refused to complete the post-operative STAI).	**LoS days I=48:C=52**<br>I=8.1 (2.5): C=7.9 (2.4), P=0.71<br>**Pain I=48:C=52**<br>**Before surgery**<br>I=24 (21):C=35 (29), P=0.04	**Outcomes reported: Anxiety (assessed using the State Trait Anxiety Inventory) Time to standing (days), Patient satisfaction**<br>No difference between groups

ID	Study	Total number of withdrawals (n)	Clinical outcomes(mean (SD) or n unless otherwise stated)	Intervention-specific outcomes [(n or mean (SD) unless otherwise reported)] and economic evaluations
			**After surgery**<br>I=21 (18): C=28 (22), P=0.07<br>**Complications (n)**<br>I=9/48: C=6/52, P=0.40	
6	Johansson et al., 2007	12 ppts at admission and 5 ppts dropped out at discharge	**LoS days (mean only) I=55:C=51**<br>I=6.8: C=8.2 (NS)<br>*Imputed SD (mean of all SD results)*<br>*I= 6.8 (28.94): C=8.2 (24.48)*<br>**Less need of further care**<br>I=7.7/55 (14%): C=11.7/51 (22.9%) (NS)	**Outcomes reported: Length of admission discussion (mins), Orthopedic Patient Knowledge Questionnaire (OPKQ -39 item score), Empowerment assessed using the Modified Empowerment Questionnaire (MEQ)**<br>**Length of admission discussion (mins)**<br>I=13: C=33, P<0.0001<br>**Orthopedic Patient Knowledge Questionnaire (OPKQ -39 item score)**<br>significantly higher from baseline in I vs C, at admission and at discharge, p=0.021 and p=0.022, respectively.<br><br>Empowerment (MEQ) was only assessed at admission. In all 10 items (measured in terms of total mean scores), the preadmission education was statistically significantly more empowering for patients in I group than for those in C group (P < 0.001, Cohen's d = 1.34)
7	Kesanen et al., 2017	I=0<br>C=3<br>(surgery cancelled=2 died=1)	**Mortality in hospital**<br>I=0/50: C=1/50<br>**Mortality at 3 mth FU**<br>I=0/50:C=1/47<br>No between group stats reported<br>**LoS days I=50:C=47**<br>I=7.1 (2.4): C=7.5 (2.6), P=0.446<br>**Health related QoL 0 to 100 with higher scores indicating better I=45:C=39**<br>Although no significant differences between groups emerged, some subscales of RAND-36 showed a trend in favor of the I group (social functioning, vitality, and emotional role functioning) within the first 3 mths<br>**PO pain (VAS scores)** No sig between-group differences were detected (back:P=0.78, leg:P=0.98) during first 3 mths	**Outcomes reported: anxiety** (STAI)**; disability (Oswestry Disability Index):**<br>No difference between groups in anxiety (assessed using) or disability (assessed using the
8	Koyuncu et al., 2013	NR	**PO pain (VAS) I=17:C=20 (assumed)**<br>Both resting and motion VAS scores of the I group were lower in I vs C on PO day 1, 2, 3 (P<0.01) but data not available (abstract only)	NR
9	Magdaleno et al., 2018	Lost to FU<br>I=1<br>C=1<br><br>Interruption of intervention (hosptialisation)	**Hospital readmission**<br>I=2/31: C=3/31, P=0.194<br>**Adverse events (Clavian Dindo classification)**<br>**Nausea & vomiting**<br>I=1/31*: C=1/31*, P=0.432<br>**Unexpected hosptialisations**	**Outcomes reported: Satisfaction survey**<br>No difference between I and C<br>A total of 98% considered they had their surgery explained thoroughly

ID	Study	Total number of withdrawals (n)	Clinical outcomes(mean (SD) or n unless otherwise stated)	Intervention-specific outcomes [(n or mean (SD) unless otherwise reported)] and economic evaluations
		I=1<br>C=3	I=2/31 (acute cholecystitis*): C=3/31 (2 empyema of gallbladder*), P=0.194<br>**Re-admissions**<br>I=0/31: C=0/31, P=0.362<br>**PO complications**<br>I=1/31: C=1/31, P=0.872<br>**Placement of additional trocar**<br>I=1/31: C=1/31, P=0.836<br>**Conversion to open surgery**<br>I=0/31: C=0/31, P=0.568<br>*required hospital admission<br>**Outpatient management rate** was 92% (57 ppts)<br>**Pain VAS I=29:C=28**<br>**24 hrs abdominal pain**<br>I=2.9: C=2.7, P=0.782<br>**24 hrs subscapular pain**<br>I=2.3: C=2.4, P=0.927<br>**7 days abdominal pain**<br>I=1.1: C=0.9, P=0.837<br>**7 days subscapular pain**<br>I=0: C=1, P=0.193<br>30-day pain also reported<br>**Quality of life**<br>No difference between I and C in pre-post results of the SF12	
10	McGregor et al., 2004	I=4	**LoS days**<br>"I group reduced LoS by 3 days"<br>**PO complications**<br>Minor PO complications occurred, but these seemed to show a similar incidence in each group.<br>**Pain** WOMAC (pain, stiffness, function)<br>Pain VASs showed significant improvements with time (P=0.001) independent of study group. No between group stats reported.<br>**HrQoL (assessed by EQ5D)**<br>No difference between groups at admission, discharge and 3 mth.<br>**PO Satisfaction with Surgery**<br>Ppts in I reported higher levels of satisfaction than ppts in C (P<0.01)	**Outcomes reported (at discharge and 3 mo PO): WOMAC (pain, stiffness, function); The Barthel Activities of Daily Living Index; Positive Affect Negative Affect Scale; The helplessness short subscale of the Rheumatology Attitudes Index; Cantril Life Satisfaction Ladder**.<br>No between group comparison for any of these outcomes.<br><br>**Economic analysis**<br>There was no difference in QoL (EQ5D) between groups, but cost was lower in I vs C (£2,842 vs £3,429).
11	Pazar and Iyigun 2020<br>Turkey	I=2 (long-term stay on ICU)<br>C=3 (2 long-term stay on ICU, 1 described as 'Ex patients')	Differences in haemodynamic measurements in favour of the I group after patients woke up in the ICU were observed for:<br>breathing rate (p<0.001), systolic blood pressure (p=0.006), oxygen saturation (p=0.012), heart rate (p=0.045), body temperature (p=0.014).<br>Differences in measurements taken in the ICU during mechanical ventilation and before extubation in favour of the I group were observed for: breathing rate (p=0.001), systolic blood pressure (p<0.001), diastolic	**Outcomes reported: patient comfort and anxiety, and patient-ventilator synchrony**<br><br>Median (IQR) scores in the I and C groups, respectively, for the Perianaesthesia Comfort Questionnaire were 5.7(5.4-5.7) and 4.1(2.4-3.4) (p<0.001) and for the Tension-Anxiety subscale of the Profile of Mood Scale were 2.0 (0-0) and 24.0 (2-2) (p<0.001).

ID	Study	Total number of withdrawals (n)	Clinical outcomes(mean (SD) or n unless otherwise stated)	Intervention-specific outcomes [(n or mean (SD) unless otherwise reported)] and economic evaluations
			blood pressure (p<0.001), oxygen saturation (p=0.002), $PaCO_2$ (p=0.015), heart rate (p<0.001), body temperature (p=0.001).	Fewer people in the I than C group experienced:<br>Agitation, fear, panic and unrest; Lifting the Tube; Biting the Tube; Use of Accessory Respiratory Muscles; Struggle with Mechanical Ventilator; Sedation after Waking up; Negative Reaction to Aspiration. More people in the I than C group showed: Adequate Communication; Cooperation; Harmony between the Thorax Movement and Ventilation.
12	Shuldham et al., 2002	**Did not follow protocol (n) I=49:C=11**<br>[NB – included in intention to treat (ITT) analyses]<br>**Withdrawn**<br>I=15:C=12	**LoS days (I=162:C=152)**<br>I=10.07 (5.04):C=9.15 (4.38), P=0.01<br>**Pain (VAS) median (range)I=173:C=156 BL to 6 mths**<br>I=40 (0–98):C=37 (0–94), P=0.93<br>**Psychological well-being questionnaires (HAD) 6 mths**<br>**Anxiety Median (range)I=169:C=151**<br>I=2 (-9 to +13):C=2 (-8 to +13), P=0.09<br>**Depression Median (range) I=166:C=148**<br>I=2 (-7 to +14):C=2 (-8 to +12), P=0.62<br>**General Wellbeing Questionnaire:**<br>**Worn out Median (range) I=160:C=143**<br>I=2 (-19 to +20):C=3 (-14 to +30), P=0.11<br>**Tense and uptight Median (range) I=162:C=145**<br>I= 3 (-13 to +21):C=3 (-13 to +34), P=0.29<br>**QoL**<br>SF36 (data on mental health and physical health only reported at BL)	NR
13	Soeters et al., 2018 USA	NR	**LoS days (I=63:C=63)**<br>I=2.4 (0.9):C=2.6(0.8), P=0.077	**Outcomes reported: Time to discharge by physiotherapist, WOMAC (pain stiffness, function)**<br>Time to discharge by physiotherapist (proxy for PO function) significantly shorter in I vs C, P<0.001);<br>WOMAC<br>significant improvement in stiffness in I vs C (P=0.038) but no other parameters.
14	Vukomanovic et al., 2008	Exclusion because of intraoperative and PO complication<br>I=3:C=2<br>Drop-out<br>I=2:C=2	**LoS days I=20:C=20**<br>I=9.8 (2.4):C=10.2 (1.7)<br>**PO non-infective complications**<br>**Fracture of proximal femur**<br>I=0/20:C=1/20<br>**Fracture of acetabulum cavity**<br>I=0/20:C=1/20<br>**Hip dislocation**<br>I=1/20:C=0/20<br>**Seizure of epilepsy**<br>I=1/20:C=0/20	**Outcomes reported: flexion of the hip with flexed knee, flexion of the hip with extended knee, abduction, Harris hip score, Japanese Orthopedic Association hip score, Oxford hip score**<br><br>The only difference in baseline measures was higher Oxford hip score in the study group (higher score means worse functional status).<br><br>There were no differences between the groups on the discharge according to pain, range of motion, Harris hip score and JOA hip

ID	Study	Total number of withdrawals (n)	Clinical outcomes(mean (SD) or n unless otherwise stated)	Intervention-specific outcomes [(n or mean (SD) unless otherwise reported)] and economic evaluations
			**GI disorder**<br>I=1/20:C=0/20<br>**PO pain**<br>**Pain in rest (VAS) (mm) 15 mths after operation**<br>I=3.95 (13.08):C+6.2(14.95)<br>**Pain while move (VAS) (mm) 15 mths after operation**<br>I=10.25 (17.33):C=11.5 (17.33)	score. Oxford hip score did not differ between the groups 15 months after the operation<br><br>Performance of normal daily activities (e.g. walking, using toilet, use of chair, walking up and down stairs, etc.) significant improvements in I vs C group differences in ability to perform activities on day 3 PO and at discharge
15	Watt-Watson et al., 2000	I1=2<br>I2=0<br>C=0	**LoS , days I1=15, I2=16, C=16**<br>I1=5.0 (0.9)<br>I2=6.1 (1.4)<br>C=5.1 (1.0) NS<br>**Pain**<br>**Day 3 post-surgery**<br>I1=2.14 (0.86)<br>I2=2.75 (1.13)<br>C=2.35 (1.15)<br>**Day 5 post-surgery**<br>I1=2.15 (0.99)<br>I2=2.25 (0.77)<br>C=2.44 (1.36)<br>No difference between groups and no difference. between days 3 and 5	**Outcome reported: Concern about seeking help (the Barriers Questionnaire). Pain treatment satisfaction, Interference in activities because of pain**<br><br>**Concern about:**<br>**seeking help and taking analgesia**<br>Lower for ppts in the I groups at day 3 (, P<0.07) and day 5 (P<0.02)<br>**Addiction**<br>Lower for ppts in the I groups (P<0.003)<br>**Pain treatment satisfaction**<br>Ppts in the I groups tended to be more satisfied with their pain treatment (P<0.06)<br>At days 3 and 5, I1 and I2 ppts received 46% and 33% more analgesia in previous 24hrs compared to C ppts<br>**Interference in activities because of pain**<br>No difference between groups but within-intervention group changes were seen, with a decrease in pain-related interference in activities between day 3 and 5 (P<0.01)
16	Watt-Watson, et al., 2004	Those with complete data<br>I=6<br>C=12<br><br>Lost to FU: 2007<br>I=6<br>C=10<br>(surgery date changed:<br>I=2:C=5<br>too ill: I=3:C=4<br>died: I=1:C=1)	**Mortality (in hospital)**<br>I=1/202: C=1/204<br>**LoS (days) I=202:C=204**<br>I=6.8 (5.9): C=6.6 (3.1), NS<br>**Pain (measured by McGill Pain Questionnaire Short Form)**<br>No difference between groups	**Outcome reported: Concern about seeking help (the Barriers Questionnaire), patient satisfaction, analgesia prescribed, or pain related interference in usual activities**<br><br>**Concerns about:**<br>Asking for help with pain medication (P>0.05)<br>Taking analgesics (P<0.005)<br>Addiction on days 3 and 5 respectively (P<0.04).<br><br>No difference in patient satisfaction, analgesia prescribed, or pain related interference in usual activities between groups
17	Wilson et al., 2011; 2016	I=1 (procedure cancelled)<br>C=2 (1 not eligible (change in surgery type), 1 procedure cancelled)	**LoS >4 days I=72:C=68**<br>I=29 (40%): C=23 (34%), P=0.22<br>*Imputed SD (mean of all SD results)*<br>*I= 29 (28.94): C=23 (24.48)*<br>**Pain** (assessed using the Brief Pain Inventory Interference subscale) on PO day 3:	**Adverse events**<br>Reported in text that no adverse effects occurred that were related to the intervention.

ID	Study	Total number of withdrawals (n)	Clinical outcomes(mean (SD) or n unless otherwise stated)	Intervention-specific outcomes [(n or mean (SD) unless otherwise reported)] and economic evaluations
			**Total (score 0-60) I=70:C=65**<br>I=24.4 (14.4): C=22.4 (15.1), P=0.45<br>**Pain (Short Form McGill Pain Questionnaire) I=62:C=55**<br>NS group differences on any of the 3 PO days in either pain right now at rest (P=0.70), pain now with movement (P=0.20) or worst pain in last 24 hours (P=0.87)	

KEY: BL=baseline; C=control; FU=follow up; HrQoL=Health related quality of life; hrs=hours; I=intervention; IQR interquartile range; LoS=length of stay; n=number; NS=non-significant; NRS=numerical rating scale; PO=post operative; PROMIS=Patient-Reported Outcomes Measurement Information System; QoL=Quality of Life; SD=standard deviation; STAI=State-Trait Anxiety Inventory; THR=Total hip replacement; VAS=visual analogue scale; wks=weeks; WOMAC=Western Ontario and McMaster Universities Osteoarthritis Index

Perry R, *et al. BMJ Open* 2022; 11:e050806. doi: 10.1136/bmjopen-2021-050806

EDUCATIONAL INTERVENTIONS

Table 23. Risk of bias

		Selection bias		Performance bias	Performance bias	Detection bias						Attrition bias	Reporting bias
						Blinding of outcome assessment							
		Random sequence generation	Allocation concealment	Blinding of participants (all outcomes)	Blinding of personnel (all outcomes)	Perioperative mortality, hospital readmission	Postoperative complications	Length of stay	Patient reported outcomes (pain, HRQoL)	Intervention related outcomes	Intervention related adverse events	Incomplete outcome data	Selective reporting
1	Boden 2018								**NR**	**NR**	**NR**		
2	Butler 1996						**NR**				**NR**		
3	Cooke 2016					**NR**	**NR**				**NR**		
4	Eschalier 2017					**NR**	**NR**			**NR**	**NR**		
5	Giraudet-Le Quintrec 2003					**NR**					**NR**		
6	Johansson 2007					**NR**	**NR**		**NR**				
7	Kesanen. 2017									**NR**	**NR**		
8	Koyuncu 2013<br>Abstract only					**NR**	**NR**	**NR**		**NR**	**NR**		
9	Magdaleno. 2018					**NR**		**NR**			**NR**		
10	McGregor 2004					**NR**					**NR**		
11	Pazar & Iyigun 2020					**NR**	**NR**	**NR**			**NR**		
12	Shuldham 2002					**NR**	**NR**			**NR**	**NR**		
13	Soeters 2018												
14	Vukomanovic 2008					**NR**					**NR**		
15	Watt-Watson 2000					**NR**	**NR**				**NR**		
16	Watt-Watson 2004						**NR**				**NR**		
17	Wilson, 2011;2016					**NR**	**NR**			**NR**			

EDUCATIONAL INTERVENTIONS

Table 24. Summary of findings

Education interventions compared to usual care for any major surgery

Patient or population: any major surgery

Setting: hospital

Intervention: education interventions

Comparison: usual care

Outcomes	Anticipated absolute effects* (95% CI)		Relative effect (95% CI)	№ of participants (studies)	Certainty of the evidence (GRADE)	Comments
	**Risk with usual care**	**Risk with education interventions**				
mortality follow up: 30 days	11 per 1,000	**9 per 1,000** (3 to 32)	**RR 0.83** (0.24 to 2.95)	938 (3 RCTs)	⨁⨁◯◯ LOW [a,b]	
Length of Stay (LoS)		MD **0** (0.4 lower to 0.4 higher)	-	1920 (13 RCTs)	⨁◯◯◯ VERY LOW [b,c,d,e]	

***The risk in the intervention group** (and its 95% confidence interval) is based on the assumed risk in the comparison group and the **relative effect** of the intervention (and its 95% CI).

CI: Confidence interval; **RR:** Risk ratio; **MD:** Mean difference

GRADE Working Group grades of evidence
High certainty: We are very confident that the true effect lies close to that of the estimate of the effect
Moderate certainty: We are moderately confident in the effect estimate: The true effect is likely to be close to the estimate of the effect, but there is a possibility that it is substantially different
Low certainty: Our confidence in the effect estimate is limited: The true effect may be substantially different from the estimate of the effect
Very low certainty: We have very little confidence in the effect estimate: The true effect is likely to be substantially different from the estimate of effect

Explanations

a. Different surgical populations/different interventions
b. Downgrade for Imprecision- includes null effect and appreciable benefit or harm; small number of events
c. Downgrade for risk of bias
d. Downgrade for inconsistency: variation in CI, not in same direction
e. Downgrade for Imprecision- includes null effect and appreciable benefit or harm

Meta analyses

Figure 67. Random effects meta-analysis of the risk ratio of mortality between education (experimental) and usual care (control).

Study or Subgroup	Experimental Events	Experimental Total	Control Events	Control Total	Weight	Risk Ratio M-H, Random, 95% CI
Boden 2017	3	218	3	214	63.3%	0.98 [0.20, 4.81]
Kesanen 2017	0	50	1	50	15.8%	0.33 [0.01, 7.99]
Watt-Watson 2004	1	202	1	204	20.9%	1.01 [0.06, 16.04]
**Total (95% CI)**		**470**		**468**	**100.0%**	**0.83 [0.24, 2.95]**
Total events	4		5			

Heterogeneity: Tau² = 0.00; Chi² = 0.38, df = 2 (P = 0.83); I² = 0%
Test for overall effect: Z = 0.28 (P = 0.78)

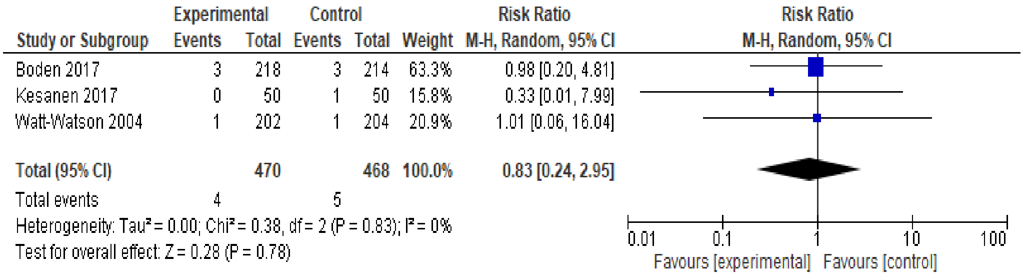

3 studies (938 participants). 1 study (Butler et al., 1996) did not identify in which group the death occurred so was not included in the meta-analysis.

Figure 68. Random effects meta-analysis of the mean difference in length of hospital stay (LoS) between education interventions (experimental) and usual care (control).

Study or Subgroup	Experimental Mean	Experimental SD	Experimental Total	Control Mean	Control SD	Control Total	Weight	Mean Difference IV, Random, 95% CI
Boden 2017	8.95	4.28	218	10.46	5.29	214	10.0%	-1.51 [-2.42, -0.60]
Butler 1996	10.3	4.7	30	10.4	5.5	40	2.5%	-0.10 [-2.49, 2.29]
Eschalier 2017	9.6	2.8	22	8.8	2	20	5.5%	0.80 [-0.66, 2.26]
Giraudet-Le Quintrec 2003	8.1	2.5	48	7.9	2.4	52	9.4%	0.20 [-0.76, 1.16]
Johansson 2007	6.8	28.94	55	8.2	24.48	51	0.2%	-1.40 [-11.58, 8.78]
Kesanen 2017	7.1	2.4	50	7.5	2.6	47	9.0%	-0.40 [-1.40, 0.60]
Shuldham 2002	10.07	5.04	162	9.15	4.38	152	8.6%	0.92 [-0.12, 1.96]
Soeters 2018	2.4	0.9	63	2.6	0.8	63	18.3%	-0.20 [-0.50, 0.10]
Vukomanovic 2008	9.8	2.4	20	10.2	1.7	20	6.6%	-0.40 [-1.69, 0.89]
Watt-Watson 2000 Booklet only	5	0.9	15	5.1	1	8	10.9%	-0.10 [-0.93, 0.73]
Watt-Watson 2000 Interview	6.1	1.4	16	5.1	1	8	9.2%	1.00 [0.02, 1.98]
Watt-Watson 2004	6.8	5.9	202	6.6	3.1	204	9.8%	0.20 [-0.72, 1.12]
Wilson 2011; 2016	29	28.94	72	23	24.48	68	0.2%	6.00 [-2.86, 14.86]
**Total (95% CI)**			**973**			**947**	**100.0%**	**0.00 [-0.40, 0.40]**

Heterogeneity: Tau² = 0.21; Chi² = 22.93, df = 12 (P = 0.03); I² = 48%
Test for overall effect: Z = 0.01 (P = 1.00)

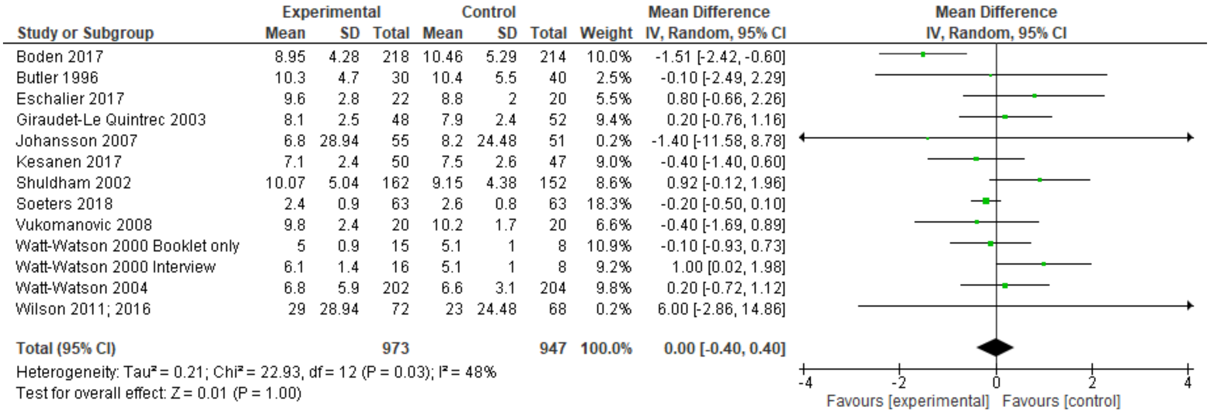

13/17 studies (1920 participants).

Perry R, et al. BMJ Open 2022; 11:e050806. doi: 10.1136/bmjopen-2021-050806

Sensitivity analyses (removing studies at high risk of bias)

Figure 69. Random effects meta-analysis of the mean difference in length of stay between education (experimental) and usual care (control) with high risk of bias studies removed.

Study or Subgroup	Experimental Mean	Experimental SD	Experimental Total	Control Mean	Control SD	Control Total	Weight	Mean Difference IV, Random, 95% CI
Boden 2017	8.95	4.28	218	10.26	5.29	214	20.1%	-1.31 [-2.22, -0.40]
Eschalier 2017	9.6	2.8	22	8.8	2	20	13.4%	0.80 [-0.66, 2.26]
Kesanen 2017	7.1	2.4	50	7.5	2.6	47	18.9%	-0.40 [-1.40, 0.60]
Shuldham 2002	10.07	5.04	162	9.15	4.38	152	18.3%	0.92 [-0.12, 1.96]
Soeters 2018	2.4	0.9	63	2.6	0.8	63	28.0%	-0.20 [-0.50, 0.10]
Wilson 2011;2016	29	8.94	72	23	24.48	68	1.3%	6.00 [-0.17, 12.17]
**Total (95% CI)**			**587**			**564**	**100.0%**	**-0.04 [-0.77, 0.68]**

Heterogeneity: Tau² = 0.47; Chi² = 15.99, df = 5 (P = 0.007); I² = 69%
Test for overall effect: Z = 0.11 (P = 0.91)

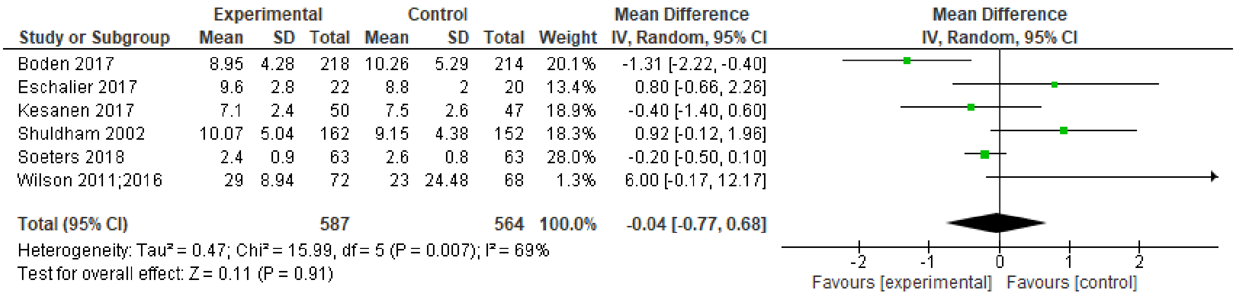

6 studies (1151 participants) included and 6 studies excluded (Butler et al., 1996, Giraudet-Le Quintrec et al., 2003, Johansson et al., 2007, Watt-Watson et al., 2004, Watt-Watsone et al., 2004, ; Watt-Watson et al., 2000; Vukomanovic et al., 2008).

Sensitivity analyses (removing studies with imputed results)

Figure 70. Random effects meta-analysis of the mean difference in length of stay between education interventions (experimental) and usual care (control) with imputed results removed.

Study or Subgroup	Experimental Mean	Experimental SD	Experimental Total	Control Mean	Control SD	Control Total	Weight	Mean Difference IV, Random, 95% CI
Butler 1996	10.3	4.7	30	10.4	5.5	40	1.7%	-0.10 [-2.49, 2.29]
Eschalier 2017	9.6	2.8	22	8.8	2	40	5.1%	0.80 [-0.52, 2.12]
Giraudet-Le Quintrec 2003	8.1	2.5	48	7.9	2.4	52	8.8%	0.20 [-0.76, 1.16]
Kesanen 2017	7.1	2.4	50	7.5	2.6	47	8.3%	-0.40 [-1.40, 0.60]
Shuldham 2002	10.07	5.04	162	9.15	4.38	152	7.7%	0.92 [-0.12, 1.96]
Soeters 2018	2.4	0.9	63	2.6	0.8	63	33.7%	-0.20 [-0.50, 0.10]
Vukomanovic 2008	9.8	2.4	20	10.2	1.7	20	5.3%	-0.40 [-1.69, 0.89]
Watt-Watson 2000 Booklet only	5	0.9	15	5.1	1	8	11.2%	-0.10 [-0.93, 0.73]
Watt-Watson 2000 Interview	6.1	1.4	16	5.1	1	8	8.6%	1.00 [0.02, 1.98]
Watt-Watson 2004	6.8	5.9	202	6.6	3.1	204	9.5%	0.20 [-0.72, 1.12]
**Total (95% CI)**			**628**			**634**	**100.0%**	**0.10 [-0.22, 0.42]**

Heterogeneity: Tau² = 0.05; Chi² = 11.56, df = 9 (P = 0.24); I² = 22%
Test for overall effect: Z = 0.62 (P = 0.54)

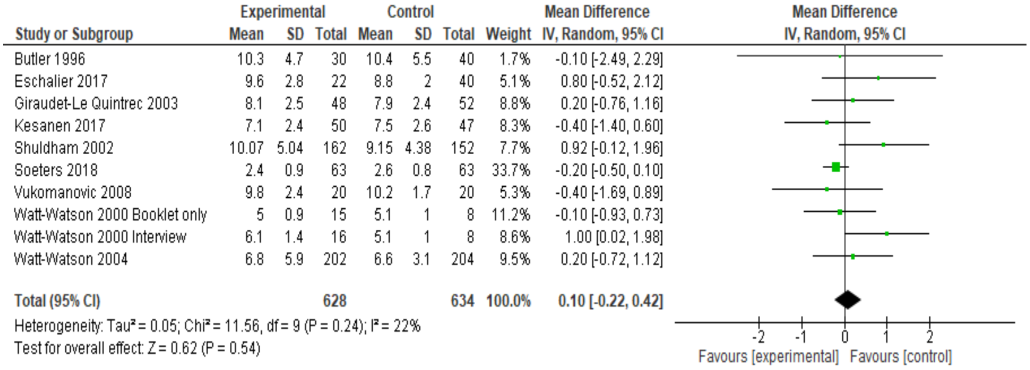

9 studies (1269 participants) included and 3 studies excluded (Johansson et al., 2007, Boden et al., 2018, Wilson, 2011).

Subgroup analysis (studies published before and after 2010)

A subgroup analysis for mortality could not be performed because there were insufficient studies available.

Figure 71. Random effects meta-analysis of the mean difference in length of hospital stay (LoS) between inspiratory muscle training (experimental) and usual care (control) in studies published after 2010.

	Experimental			Control				Mean Difference
Study or Subgroup	Mean	SD	Total	Mean	SD	Total	Weight	IV, Random, 95% CI
Eschalier 2017	9.6	2.8	22	8.8	2	40	8.2%	0.80 [-0.52, 2.12]
Kesanen 2017	7.1	2.4	50	7.5	2.6	47	13.8%	-0.40 [-1.40, 0.60]
Soeters 2018	2.4	0.9	63	2.6	0.8	63	78.0%	-0.20 [-0.50, 0.10]
**Total (95% CI)**			**135**			**150**	**100.0%**	**-0.15 [-0.54, 0.24]**

Heterogeneity: Tau² = 0.03; Chi² = 2.30, df = 2 (P = 0.32); I² = 13%
Test for overall effect: Z = 0.73 (P = 0.46)

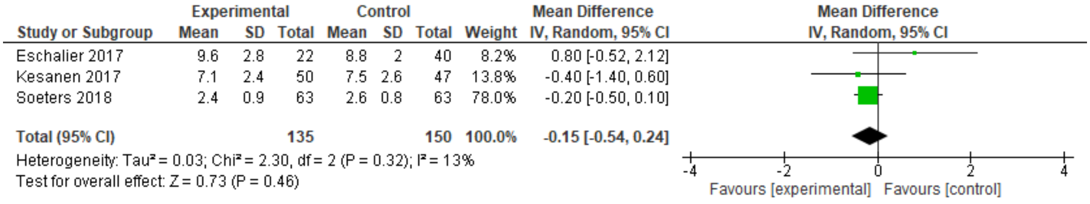

3 studies (285 participants) included and 6 stfuies excluded (Butler et al, 1996; Giraudet-Le Quintrec et al., 2003; Shuldham et a;., 2002; Vukomanovic et al., 2008; Watt-Watson et al., 2000; Watt-Watson et al., 2004).

References

Boden, I., Skinner, E. H., Browning, L., Reeve, J., Anderson, L., Hill, C., . . . Denehy, L. 2018. Preoperative physiotherapy for the prevention of respiratory complications after upper abdominal surgery: pragmatic, double blinded, multicentre randomised controlled trial. *Bmj,* 360**,** j5916.

Butler, G. S., Hurley, C. A., Buchanan, K. L. & Smith-Vanhorne, J. 1996. Prehospital education: effectiveness with total hip replacement surgery patients. *Patient Educ Couns,* 29**,** 189-97.

Cooke, M., Walker, R., Aitken, L. M., Freeman, A., Pavey, S. & Cantrill, R. 2016. Pre-operative self-efficacy education vs. usual care for patients undergoing joint replacement surgery: a pilot randomised controlled trial. *Scand J Caring Sci,* 30**,** 74-82.

Eschalier, B., Descamps, S., Pereira, B., Vaillant-Roussel, H., Girard, G., Boisgard, S. & Coudeyre, E. 2017. Randomized blinded trial of standardized written patient information before total knee arthroplasty. *PLoS One,* 12**,** e0178358.

Giraudet-Le Quintrec, J. S., Coste, J., Vastel, L., Pacault, V., Jeanne, L., Lamas, J. P., . . . Courpied, J. P. 2003. Positive effect of patient education for hip surgery: a randomized trial. *Clin Orthop Relat Res***,** 112-20.

Johansson, K., Salanterä, S. & Katajisto, J. 2007. Empowering orthopaedic patients through preadmission education: results from a clinical study. *Patient Educ Couns,* 66**,** 84-91.

Kesänen, J., Leino-Kilpi, H., Lund, T., Montin, L., Puukka, P. & Valkeapää, K. 2017. Increased preoperative knowledge reduces surgery-related anxiety: a randomised clinical trial in 100 spinal stenosis patients. *Eur Spine J,* 26**,** 2520-2528.

Koyuncu, O., Canbulat, N., Seyahi, A., Uzun, A., Guzelocak, E. & Demirhan, M. 2013. Does preoperative patient education have an effect on early postoperative pain levels in patients having arthroscopic rotator cuff surgery?, Artroskopik Rotator Manset Cerrahisi Oncesi Verilen Hasta Etitiminin Cerrahi Sonrasi Erken Donemde Atri Seviyesi Uzerine Etkisi Var midir?. [Turkish, English]. *Turkiye Fiziksel Tip ve Rehabilitasyon Dergisi,* 59**,** 432.

Magdaleno, H., Tarragó, A., Casas, C., Padillo, A., Chacón, M., Bargalló, J., . . . Martín, R. 2018. Evaluation of the impact of preoperative education in ambulatory laparoscopic cholecystectomy. A prospective, double-blind randomized trial. *Cir Esp,* 96**,** 88-95.

Mcgregor, A. H., Rylands, H., Owen, A., Doré, C. J. & Hughes, S. P. 2004. Does preoperative hip rehabilitation advice improve recovery and patient satisfaction? *J Arthroplasty,* 19**,** 464-8.

Pazar, B. & Iyigun, E. 2020. The effects of preoperative education of cardiac patients on haemodynamic parameters, comfort, anxiety and patient-ventilator synchrony: A randomised, controlled trial. *Intensive and Critical Care Nursing,* 58.

Shuldham, C. M., Fleming, S. & Goodman, H. 2002. The impact of pre-operative education on recovery following coronary artery bypass surgery. A randomized controlled clinical trial. *Eur Heart J,* 23**,** 666-74.

Soeters, R., White, P. B., Murray-Weir, M., Koltsov, J. C. B., Alexiades, M. M. & Ranawat, A. S. 2018. Preoperative Physical Therapy Education Reduces Time to Meet Functional Milestones After Total Joint Arthroplasty. *Clin Orthop Relat Res,* 476**,** 40-48.

Vukomanović, A., Popović, Z., Durović, A. & Krstić, L. 2008. The effects of short-term preoperative physical therapy and education on early functional recovery of patients younger than 70 undergoing total hip arthroplasty. *Vojnosanit Pregl,* 65**,** 291-7.

Watt-Watson, J., Stevens, B., Costello, J., Katz, J. & Reid, G. 2000. Impact of preoperative education on pain management outcomes after coronary artery bypass graft surgery: a pilot. *Can J Nurs Res,* 31**,** 41-56.

Watt-Watson, J., Stevens, B., Katz, J., Costello, J., Reid, G. J. & David, T. 2004. Impact of preoperative education on pain outcomes after coronary artery bypass graft surgery. *Pain,* 109**,** 73-85.

Wilson, R. 2011. *A Randomized Controlled Trial of an Individualized Preoperative Education Intervention for Symptom Management Following Total Knee Arthroplasty.* PhD, University of Toronto

PSYCHOLOGICAL INTERVENTIONS

Table 25. Characteristics of Studies

ID	1st Author, year and country	Total *n* Intervention (I) Control (c)<br><br>Number analysed (An if reported)	Patient population, baseline clinical characteristics (mean (SD) or *n (%)* unless otherwise stated	Demographics (mean (SD) or *n (%)* unless otherwise stated)	Intervention	Comparator	Mode of delivery; place of delivery; training level of individuals who delivered the intervention; the number of contacts	Intervention fidelity; Compliance or adherence to intervention
**PSYCHOLOGICAL INTERVENTIONS**								
1	Auer et al., 2017;<br>Salzmann et al., 2017;<br>Rief et al., 2017<br>Germany<br>*The PSY-HEART* trial	124 ppts undergoing elective CABG or CABG/valve<br><br>I1=41 Supportive information (SUPPORT)<br>I2=39 Expectation manipulation intervention (EXPECT)<br>C=44 standard medical care (SMC)	**NYHA class 3-4 %**<br>I1=65<br>I2=63<br>C=74<br>**NYHA class (n) I/2/3/4**<br>I1=1/15/20/2<br>I2=0/16/19/3<br>C=1/11/31/1<br>**LVEF <=49% (%)**<br>I1=44.5<br>I2=16.7<br>C=37.5<br>**LVEF (≥50%:40-30%:<30%)**<br>I1=19/14/2<br>I2=30/6/0<br>C=25/13/2<br>**EuroSCORE [mean (sd)]**<br>I1=2.24(4.52)<br>I2=1.25(0.79)<br>C=1.54(0.84)	**Age (yrs)**<br>I1=64.6(8.1)<br>I2=65.8(7.8)<br>C=67.1(8.9)<br>**Gender (%M)**<br>I1=81:I2=87<br>C=88<br>**Smokers (%)**<br>I1=6:I2=16<br>C=15	**I1: (SUPPORT)**: An expectation manipulation intervention to optimize patients' expectations about course and outcomes or supportive therapy, containing the same amount of therapeutic attention, but without specifically focusing on expectations.<br>**I2 (EXPECT)**: An expectation manipulation intervention involving two individual sessions (50 min) plus two phone calls (20 min) between baseline assessment (approx. 10 days before surgery) and surgery	Standard medical care	Verbal;<br>Not stated but assume in clinic (& 2 phone calls);<br>Clinical psychologists with advanced CBT training;<br>4 contacts (2 face-to-face, 2 phone calls)	Treatment fidelity for both treatments reported as high;<br>NR
2	Cohen et al., 2011<br>USA	159 men undergoing radical prostatectomy randomized into:	>70% white ethnicity;<br>>80% married. Clinical characteristics NR.	**Age (yrs)**<br>SM=59.8(6.9)<br>SA=60.7(7.2)<br>C=60.9(5.9)	**Stress management:** 2x cognitive-behavioral sessions with psychologist (including breathing, diaphragmatic breathing and guided imagery).<br>**Supportive Attention:** 2x 'supportive' sessions with	Usual care	Verbal by clinical psychologist + written (SM only);<br>Not stated but assume in hospital;<br>NR but intervention delivered by clinical psychologists, therefore assume appropriately trained;<br>NR	No monitoring of intervention delivery but the same psychologists delivered both SM and SA sessions;<br><br>NR whether ppts performed any of the techniques at home

ID	1st Author, year and country	Total *n* Intervention (I) Control (c)<br><br>Number analysed (An if reported)	Patient population, baseline clinical characteristics (mean (SD) or *n* (%) unless otherwise stated	Demographics (mean (SD) or *n* (%) unless otherwise stated)	Intervention	Comparator	Mode of delivery; place of delivery; training level of individuals who delivered the intervention; the number of contacts	Intervention fidelity; Compliance or adherence to intervention
**PSYCHOLOGICAL INTERVENTIONS**								
		I stress management (SM)=53<br>I supportive attention (SA)=54<br>C=52			psychologist (listening and empathy).<br>Both interventions lasted 60 to 90 mins, 1-2 wks before surgery. Both groups received additional brief booster sessions on day of surgery and 48hrs after.			
3	Furze et al., 2009<br>UK	204 ppts undergoing elective CABG<br><br>I=100:C=104	**Severity of illness NYHA class**<br>I=2.01(0.83)<br>C=2.15(0.83)<br>**Canadian angina class**<br>I=2.26(0.68)<br>C=2.17(0.80)	**Age (yrs)**<br>I=64.3(8.8)<br>C=65.3(8.5)<br>**Gender (%M)**<br>I=85:C=76<br>**Smoking (%)**<br>I=10:C=8	Initial interview (45-60 min).<br>A 2-part patient-held booklet (the HeartOp Plan) covering cardiac myths and misconceptions. Reducing risk factors for secondary prevention, what to expect during hospital stay and subsequent recovery period. Relaxation programme on tape/CD - diary to record activity & risk factor reduction goals. The facilitator initially aims to dispel specific cardiac misconceptions and work with the patient to agree and set goals to reduce CV risk and increase activity levels. Several FU phone calls	Same initial interview (45-60 mins). Verbal advice on risk factors and description of operation and aftercare plus written information from British Heart Foundation.<br>No attempt to elicit specific misconceptions but if asked about specific misconception these were dispelled. General advice given (no formal goal setting). FU phone calls.	Verbal, written, audiovisual aids, phone calls;<br>Hospital plus home (via phone);<br>Nurse facilitator;<br>Phone call at wk 1,3,6 (+/- 1 wk), and then every mth until admission.	NR;<br>NR
4	Gade et al., 2014, Gade et al., 2017 (abstract) Hjelmesæth et al., 2019<br>Norway	102 morbidly obese ppts admitted for bariatric surgery<br>I=50:C=52	**BMI (kg/m^2)**<br>I=43.6 (5.1)<br>C=43.5 (4.7)<br>**Weight (kg)**<br>I=129.1 (18.0)<br>C=126.9 (20.1)	**Age (yrs)**<br>I=44.1 (9.8)<br>C=41.2 (9.6)<br>**Gender (%M)**<br>I=38:C=27	10 sessions based on theoretical principles from CBT, to recognize triggers of DE, identifying associated cognitions and emotions, initiating plans for change, and use of home-work task in between the sessions.<br>Sessions 1-2 included strategies to enhance intrinsic motivation	Usual care:<br>consisting of up to 3 voluntary sessions with nutritionist and physiotherapist before bariatric surgery and received detailed information about the mandatory low calorie diet the last 3 wks	Expert-led;<br>5 sessions at treatment center, and 6 as scheduled telephone calls;<br>?<br>11	NR;<br>NR

ID	1st Author, year and country	Total *n* Intervention (I) Control (c)<br><br>Number analysed (An if reported)	Patient population, baseline clinical characteristics (mean (SD) or *n (%)* unless otherwise stated	Demographics (mean (SD) or *n (%)* unless otherwise stated)	Intervention	Comparator	Mode of delivery; place of delivery; training level of individuals who delivered the intervention; the number of contacts	Intervention fidelity; Compliance or adherence to intervention
**PSYCHOLOGICAL INTERVENTIONS**								
					and addressed resistance to change<br><br>Sessions 2–11 were based on CBT-principles.<br><br>During the 4 mths prior to surgery, received up to 3 voluntary sessions with nutritionist and physiotherapist before bariatric surgery and received detailed information about the mandatory low calorie diet the last 3 wks			
5	Goodman et al., 2008 UK	188 ppts listed for coronary artery bypass surgery with at least 1 poorly controlled risk factor<br><br>I=94:C=94	**BMI ≥28 kg/m^2**<br>I=63:C=53	**Age (yrs)**<br>I=63.7:C=65.9<br>**Gender (%M)**<br>I=77:C=86<br>**Smokers (n)**<br>I=6:C=6	The Fit For Surgery Programme<br><br>Each mthly pre-op appointment with the cardiac homecare nurse had 3 key aims;<br>1) Ask questions and voice concerns about individual needs and the operation and be counselled regarding their anxieties.<br>2) Undergo a cardiac risk assessment to assist them to make lifestyle changes. (including blood pressure, BMI, total and HDL cholesterol).<br>3) Counselling regarding lifestyle changes in response to the risk factor assessment and according to motivational interviewing techniques	Received standard care<br><br>Hospital helpline numbers plus a pre-surgery information day in addition to a baseline assessment.<br>They were visited at home at the measurement points by a research assistant (independent of the study and homecare teams) to take blood samples and administer the questionnaires (which were sent to the I group with pre-paid envelopes).	Face to face, written;<br>Home;<br>Cardiac nurse;<br>Up to 5 x	NR;<br>NR

Perry R, *et al. BMJ Open* 2022; 11:e050806. doi: 10.1136/bmjopen-2021-050806

ID	1st Author, year and country	Total *n* Intervention (I) Control (c)<br><br>Number analysed (An if reported)	Patient population, baseline clinical characteristics (mean (SD) or *n (%)* unless otherwise stated	Demographics (mean (SD) or *n (%)* unless otherwise stated)	Intervention	Comparator	Mode of delivery; place of delivery; training level of individuals who delivered the intervention; the number of contacts	Intervention fidelity; Compliance or adherence to intervention
**PSYCHOLOGICAL INTERVENTIONS**								
					Plus copy of the manual - nurses guided them through the sections covering risk factors, preparation for surgery and what to do if they encounter chest pain.<br>Plus can telephone the team between visits<br><br>The median wait was 5.5 mths (range 1.5–8.9)	The median wait was 5.4 mths (range 1.6–11.5)		
6	Loi et al., 2016 Abstract only, Country NR	40 ppts with colorectal cancer<br>I=20:C=20		**Overall Age (yrs, range)**<br>21-80	A psychoeducation intervention over 6 wks involving a presurgery patient education session, educational booklets and 2 FU phone calls	Usual care	Written and oral;<br>Home based;<br>NR;<br>NR	NR;<br>NR
7	Lotzke et al., 2019<br>Sweden	118 ppts undergoing lumbar fusion surgery<br>I=59:C=59	**Smoking**<br>I=3(5.1)<br>C=5(8.5)<br>**Comorbidity**<br>I=6(10.2)<br>C=7(11.9)<br>**Previous lumbar spine surgery**<br>**None %**<br>I=93.2:C=89.8<br>**1 surgery %**<br>I=6.8:C=5.1<br>**2 surgeries %**<br>I=0.0:C=5.1	**Age (yrs)**<br>I=44.8(8.2)<br>C=46.7(8.5)<br>**Gender(%M)**<br>I=44:C=49<br>**BMI kg/m^2**<br>I=26.3(3.9)<br>C=26.4(3.4)	Physiotherapeutic prehabilitation program based on a cognitive behavioural (fear-avoidance) approach.<br><br>Session 1: person-centered analysis of functioning<br>Session 2: Educational session<br>Session 3: Cognitive behavioural experiment<br>Session 4: Goal setting after surgery<br>Session 5: Booster session (2wks after surgery)<br><br>8-12 wks before surgery:4 x 1hr sessions before surgery. 1 half-hour telephone session 2 wks after surgery.	Usual care	Face-to-face and phone call;<br>Spine clinic and continuation at home;<br>Physical therapist had >10 yrs of clinical experience of ppts with LBP and 1.5 yrs education and training, with a Graduate Diploma in Cognitive and Behavioral Psychotherapy;<br>8-12 wks before surgery:4 x 1hr sessions before surgery. 1 half-hour telephone session 2 wks after surgery.	The treatment “dose” in the active intervention was controlled for by using the same pattern of sessions (number, frequency, and length of contact) as in the previously published study protocol.<br>NR

ID	1st Author, year and country	Total *n* Intervention (I) Control (c)<br><br>Number analysed (An if reported)	Patient population, baseline clinical characteristics (mean (SD) or *n* *(%)* unless otherwise stated	Demographics (mean (SD) or *n* *(%)* unless otherwise stated)	Intervention	Comparator	Mode of delivery; place of delivery; training level of individuals who delivered the intervention; the number of contacts	Intervention fidelity; Compliance or adherence to intervention
**PSYCHOLOGICAL INTERVENTIONS**								
8	Ma et al., 1996 China	51 ppts with abdominal surgery I=25:C=26	**State anxiety scores**<br>No data reported but significant difference between two groups (P<0.05) on the operation day and 3rd day before operation<br>**Physical symptoms of anxiety, mean (sd)**<br>3 days before operation:<br>I=14.84(2.8)<br>C=15.62(3.01)<br>On the operation day:<br>I=15.92(4.05)<br>C=16.5(3.37)<br>**Systolic pressure (kPa) mean (sd)**<br>3 days before operation:<br>I=15.98(2.41)<br>C=15.15(2.82)<br>On the operation day:<br>I=-1.23(2.01)<br>C=0.05(2.35)<br>**Diastolic pressure (kPa) mean (sd)**	**Overall Gender (%M)**<br>70.5	Standard hospital preparatory instructions. Relaxation training start from 4 days before ppts' abdominal surgery: lay down with eyes closed and relax every single part of muscle in turn, do it 30 mins 3x/day.	Standard hospital preparatory instructions	NR (presume face to face);<br>NR (presume hospital);<br>NR;<br>NR	NR;<br>NR

ID	1st Author, year and country	Total *n* Intervention (I) Control (c) Number analysed (An if reported)	Patient population, baseline clinical characteristics (mean (SD) or *n (%)* unless otherwise stated	Demographics (mean (SD) or *n (%)* unless otherwise stated)	Intervention	Comparator	Mode of delivery; place of delivery; training level of individuals who delivered the intervention; the number of contacts	Intervention fidelity; Compliance or adherence to intervention
**PSYCHOLOGICAL INTERVENTIONS**								
			3 days before operation:<br>I=9.77(1.53)<br>C=9.44(1.58)<br>On the operation day:<br>I=-0.30(1.25)<br>C=-0.07(1.73)					
9<br>G<br>H	Marinelli et al., 2020<br>Italy	400 ppts undergoing pancreatic surgery<br>I=200 (49An)<br>C=200 (65An)	**Citizenship n(%)**<br>Italian, 395(99)<br>**Smoker n(%)**<br>No, 334(84)<br>**Alcohol n(%)**<br>No, 396(99)	**Overall Age (yrs, mean)**<br>62<br>**Overall Gender (%M)**<br>53	1 hr session where ppt disclosed concerns and worries about surgery and then the "Four Elements" protocol (chosen because it's easy to remember and refers to mindfulness techniques and guided imagery) for stress management was applied to reduce anxiety and foster abilities to cope with stress.	Usual care	Clinical psychologist and then patient-led;<br>at home;<br>Clinical psychologist;<br>1 to complete intervention	NR;<br>NR
10	Medina-Garzon, 2019<br>Columbia	56 ppts sceduled for knee replacement surgery<br>I=28 (28An)<br>C=28 (27An)	**ASA Classification; 0-1 (n)**<br>I=6:C=9<br>**2-4**<br>I=22:C=19<br>**Prior surgeries Yes n(%)**<br>I=21 (75):<br>C=15 (55.5)<br>**Anxiolytics Yes n(%)**<br>I=0 (0):C=1 (3.7)	**Age, yrs**<br>I=76.32 (16.1)<br>C=73.7 (16.6)<br>**Gender (%M)**<br>I=50:C=44	Motivational interview based on ppts establishing their own goals to change their lifestyles, by exploring their level of anxiety and the triggering factors during the 8 days prior to the interview.<br>3 sessions (of approx. 40 mins) were conducted within a 20-day period plus FU at 4 wks PO<br><br>Plus usual care	Usual care	Face to face;<br>specialized clinic;<br>trained nurse;<br>3 x	NR;<br>NR
11	Miró and Raich, 1999<br>Spain	90 ppts undergoing cholecystecto my	NR	**Overall Age yrs (mean [range])**<br>44[33-62]<br>**Gender (%M)**	I1 Information provision (sensory and procedural information)<br>I2 Relaxation training	Attention control group (neutral topic)	Face to face;<br>NR;<br>Clinical psychologist;<br>1 to complete intervention	NR:<br>Reported that most pts in I1 had engaged with the information received

ID	1st Author, year and country	Total *n* Intervention (I) Control (c)<br><br>Number analysed (An if reported)	Patient population, baseline clinical characteristics (mean (SD) or *n (%)* unless otherwise stated	Demographics (mean (SD) or *n (%)* unless otherwise stated)	Intervention	Comparator	Mode of delivery; place of delivery; training level of individuals who delivered the intervention; the number of contacts	Intervention fidelity; Compliance or adherence to intervention
**PSYCHOLOGICAL INTERVENTIONS**								
		I1=30<br>I2=30<br>C=30		47	1 session, 1 wk before surgery	1 session 1 wk before surgery		(answered between 90-95% of the questions correctly on a quiz designed to test their understanding of the information provided).<br><br>Similarly, all ppts in I2 reported to have practised relaxation at home.
12	Osinowo et al., 2003<br>Nigeria	33 ppts awaiting elective surgery<br>I1=11<br>I2=11<br>C=11	8 underwent gynaecological procedures, 25 had general surgical. Clinical characteristics NR.	**Overall Age (yrs)**<br>32.72(15.83)<br>**Gender (%M)**<br>54.5	Cognitive Behavioural Therapies<br>I1=Rational emotive therapy (RET)<br>I2=Self-instructional training (SIT)	No treatment	Face to face;<br>Carried out in patient's day room in hospital;<br>NR;<br>NR	NR;<br>NR
13	Samnani et al., 2014<br>Pakistan	232 ppts undergoing abdominal surgery<br>I=116(113An)<br>C=116(111An)	**ASA score Normal**<br>I=66:C=72<br>**Mild systemic disease**<br>I=47:C=39<br>**Emergency (either class I or II)**<br>I=62:C=55<br>**VAS pain (mean/sd)**<br>I=3.80(0.77)<br>C=3.98(0.92)	**Age (yrs)**<br>I=36.7(8.7)<br>C=37.0(8.4)<br>**Gender(%M)**<br>I=43:C=34	Information regarding surgical procedure as well as counseling related to early PO mobilization and its impact on surgical outcome.	Information regarding surgical procedure only.	NR (presume face-to-face);<br>NR (presume hospital);<br>Senior residents who were not part of the study conducted the counseling sessions;<br>NR	NR;<br>NR
14	Strom et al., 2019<br>Denmark	224 ppts awaiting first-time elective 1-3 level lumbar spine	**Indication for fusion, n (%)**<br>Spondylolisthesis	**Age [mean, (range)]**<br>I=53(29-77)<br>C=55(30-79)<br>**Gender(%M)**	Standard joint session plus access to the 'Web-based Spine Platform featuring Interaction and Information by Animation' (W-SPINA) consisting of on-line	Standard 2-hr joint information session 1-5 wks before surgery with ppts and their support person.	15 min face-to-face introduction to the site, then web-based;<br>At home;<br>NR;	Tracked from BL to 6 bmths PO.<br>All ppts accessed W-SPINA more than once.

ID	1st Author, year and country	Total *n* Intervention (I) Control (c) Number analysed (An if reported)	Patient population, baseline clinical characteristics (mean (SD) or *n (%)* unless otherwise stated	Demographics (mean (SD) or *n (%)* unless otherwise stated)	Intervention	Comparator	Mode of delivery; place of delivery; training level of individuals who delivered the intervention; the number of contacts	Intervention fidelity; Compliance or adherence to intervention
**PSYCHOLOGICAL INTERVENTIONS**								
		fusion (instrumented posterolateral fusion or transforaminal interbody fusion)<br><br>I=57 (48An)<br>C=57 (51An)	I=16(33):C=19(37)<br>Degenerative disease<br>I=64(65):C=32(66)<br>**HADS-A score [median (IQR)]**<br>I=6(4-8):C=6(4-9)<br>**HADS-D score [median (IQR)]**<br>I=5.5(3-8.5):C=5(2-7)<br>**Disability (ODI) [median (IQR)]**<br>I=48(30-54):C=49(32-60)	I=46:C=25	animated information, using CBT principals, an internet support group and a pain and physical activity diary. Animations comprised 17 sequences of 16-min animations explaining treatment and including non-catastrophic images, to modify ppts beliefs, feelings and behaviour.		Access on demand (17x 16min animations available, plus daily diary and internet support group).	90% accessed the I group, of which 48% were active users.<br>Animated information viewed 656 times<br>Diary usage = 293 times<br>I group events = 3357<br>Total number of interactions = 4306
15	Vlisides et al., 2019<br>NR<br>Abstract only	Total: 61 gerenal surgery<br>I=30 (23An)<br>C=31 (29An)	**coronary artery disease%**<br>I=14:C=16<br>**cerebrovascular disease%**<br>I= 4.3:C=3.4<br>**hypertension,**<br>I=57:C=55<br>**COPD%**<br>I=8.7:C=14<br>**chronic kidney disease**<br>I= 4.3:C= 6.9<br>**Diabetes Mellitus %**<br>I=13:C=17	**Age, yrs**<br>I=66 (4.9)<br>C=68 (5.4)<br>**Gender (%M)**<br>I=43:C=52	Cognitive training (CBT) 20 mins per day 1 session/day for 7 days<br><br>This study utilized an adaptive, computer-based cognitive training battery that specifically targets executive function, attention, working memory, and visuospatial processing (BrainHQ, Posit Science Corporation, San Francisco, CA USA),	usual care?	Computer programme;<br>Home;<br>NR;<br>NR	NR;<br>only 5/29 (17%) ppts were able to complete the prescribed 7 days of training, and 14/29 (48%) opted out of training once home.

ID	1st Author, year and country	Total *n* Intervention (I) Control (c)<br><br>Number analysed (An if reported)	Patient population, baseline clinical characteristics (mean (SD) or *n (%)* unless otherwise stated	Demographics (mean (SD) or *n (%)* unless otherwise stated)	Intervention	Comparator	Mode of delivery; place of delivery; training level of individuals who delivered the intervention; the number of contacts	Intervention fidelity; Compliance or adherence to intervention
**PSYCHOLOGICAL INTERVENTIONS**								
16	Wells et al., 1986<br>USA	24 ppts (NR by group) undergoing operations including debridement of decubitus' ulcer/skin graft, abdominal hysterectomy, cholecystostomy, fasciectomy and evacuation of hematoma, resection of cavernous hemangioma, and repair of arterial venous fistula	NR	**Age [yrs, median (range)]**<br>41 (14-62)	Conceptualisation phase of stress inoculation training (asserting that hospitalisation/ surgery is stressful), skills acquisition phase of training (e.g. deep breathing and induction of pleasant images), and application phase of training (rehearsing the skills which stressed).<br>Approx. 1 wk + practice each day.	Standard hospital preparatory instructions	Patient-led;<br>At hospital to meet with the psychologist, and then patient-led at home;<br>PhD-level;<br>Day before surgery for pre-surgical post-inoculation testing	NR;<br>NR

KEY: An=analysed; ASA=American Society of Anaesthesiologists physical status classification system; BMI=body mass index; C=control; CABG=coronary artery bypass graft; CBT=Cognitive Behavioural Therapy; CV=cardiovascular; DE=dysfunctional eating: F=female; FU=follow up; HDL=high density lipids; HM=High monitoring; hrs=hours; I=intervention; kPa=kilopascal; LM=Low monitoring; LVEF=Left ventricular ejection fraction; M=male; min=minute; mth=month; n=number; NR=not reported; NYHA=New York Heart Association; PhD=doctorate level; PO=post-operative; ppts=participants; pre-op=pre-operative; RET=Rational emotive therapy; SA=supportive attention; sd=standard deviation; SIT=Self-instructional training; SM=stress management; SMC=standard medical care; VAS=visual analogue scale; wk=week; x=times; yrs=years

PSYCHOLOGICAL INTERVENTIONS

Table 26. Results

	Study	Total number of withdrawals	Clinical outcomes (mean (SD) or n unless otherwise stated)	Intervention-specific outcomes [(n or mean (SD) unless otherwise reported)] and economic evaluations
1	Auer et al., 2017;<br>Salzmann et al., 2017;<br>Reif et al., 2017<br>I1=41<br>Supportive information (SUPPORT)<br>I2=39<br>Expectation manipulation intervention (EXPECT)<br>C=44<br>standard medical care (SMC)	I1=4<br>I2=2<br>C=3<br><br>3 ppts were excluded from final statistical analysis:<br>2 ppts were lost to BL assessment in the SUPPORT group (1 did not require surgery and 1 resigned from study before BL assessment).<br>1 ppt excluded from analysis because of violation of design requirements (>4 wks between intervention and surgery).	**LoS [days, mean (SEM)] I1=37: I2 =37: C=44**<br>*Imputed SD from mean of SDs*<br>I1 (SUPPORT) =*14.1(3.8) (SD=23.1)*<br>I2 (EXPECT) =*12.6(5.2) (SD=31.6)*<br>C=*17.3(11.1), (SD=73.6), P=0.028*<br>**ICU LoS [hours, mean (SEM)]**<br>I1=103.1(52.0)<br>I2=103.8(86.3)<br>C=158.5(180.5), P=0.066<br>Both I1 and I2 reduced hrs in ICU (P=0.029).<br>No difference between I1 and I2 (P=0.787).<br><br>**HRQOL**<br>Only reported in Reif et al., 2017<br>6-mth data only so NR here	**Outcomes reported**: **Noradrenaline (pg/mL)** and **cortisol (nmol/L)** levels did not differ significantly between treatment groups
2	Cohen et al., 2011	No withdrawals after randomization	There were no group differences in pre-, peri-, or PO complications, blood loss, length of surgery, anesthesia, and hospitalization time (data not shown).	**Outcomes reported: Immune markers:**<br>3/7 (2 interleukins and 1 tumour necrosis factor) significantly higher in I group vs. C group.
3	Furze et al., 2009	I=11 lost to FU (9 no reason, 2 had MI),<br>3 not operated on.<br><br>C=11 lost to FU (10 no reason, 1 MI), 3 not operated on.	**All-cause mortality (8 wk FU)**<br>I=1/100 (infected heart valve): C=1/104 (cerebrovascular accident)<br>**LoS (days) I=100:C=104**<br>I=7.61(2.69): C=8.28(4.96), NS<br>(when controlling for NYHA, CCSAC, BMI, systolic BP, smoking status, step-test time and Time 1 dependent variable);<br>hazard ratio=0.98, (95% CI=0.72-1.34, P=0.90)<br>**Non-fatal cardiac event**<br>I=1/100: C=2/104 no analysis<br><br>**QOL (EQ5D)**<br>I=0.109(0.003) (n=88)<br>C=0.103(0.003) (n=94)	**Outcomes reported: Cardiac beliefs/cardiac misconceptions, anxiety, depression, physical function (CLASP)**:<br>There were significant differences for cardiac depression (P=0.008), physical functioning on CLASP mobility scale (P=0.001) and cardiac beliefs (P<0.001) favouring the I vs C group<br><br>**Anxiety**<br>no differences in anxiety (mean difference when controlling for all of the covariates=1.07, (P=0.49)<br>**Anxiety and depression scores**<br>reduced in both groups at 6 months PO compared to T2<br><br>**Economic effect (8 wk FU)**<br>The HeartOp Programme had an Incremental Cost Effectiveness Ratio (ICER) of £288.83 per QALY, although there was uncertainty around this estimate.<br>Mean QALYs at 8 wks were similar for both groups (0.103 vs. 0.109), with a differential mean QALY favouring the I group of 0.006 (95% credibility intervals=−0.002 to 0.015)

	Study	Total number of withdrawals	Clinical outcomes (mean (SD) or n unless otherwise stated)	Intervention-specific outcomes [(n or mean (SD) unless otherwise reported)] and economic evaluations
4	Gade et al., 2014;2017; Hjelmesæth et al., 2019 (4 yr FU)	Discontinued due to trial fatigue<br>I=2:C=2	**Outcomes reported: Emotional eating & uncontrolled eating (TFEQ-R21); Anxiety and Depression (HADS),** DE, BMI.<br>**Anxiety & Depression:**<br>The I group showed less DE and affective symptoms (all P<0.05)<br>**BMI**<br>The I group showed a larger weight loss at follow-up (P=0.004)<br><br>**4 yr FU: additional reporting: HRQoL (weight-related)**<br>The I group did not surpass the C group in any of the outcome variables. On the contrary, ppts in the C group displayed a higher mean improvement in scores of depression (62% vs 33%, respectively, P=0.023).<br>**HRQoL**<br>did not differ significantly between groups	**NR**
5	Goodman et al., 2008	Removed from list<br>I=4:C=1<br>Died<br>I=2:C=4<br>(ITT analysis)	**Mortality**<br>Deaths prior to discharge<br>I=1/90:C=2/93<br>Total deaths<br>I=2/90:C=4/93<br>**LoS days [median(range)], I=91:C=90**<br>I=8.5(4-50): C=9(2-170), P=0.29<br>*LoS days (mean/sd Quantile Estimation (QE) conversion method)*<br>*I= 12.21 (10.27): C=20.72 (42.00)*<br>**PO complications**<br>There were no statistically significant differences in the number of complications suffered by patients in each group during the intra-hospital stay (data not shown).<br>**At 3 mths**<br>There was a reduction in risk factors in both groups but the difference between groups were not significant with wide CIs (BP, cholesterol, BMI, HAD anxiety & depression)	**Economic effect**<br>Total estimated costs for the trial period were £1,817 lower in the I arm [£10,954(3,660) vs. £12,771(5,801), 95%CI −3224 to −409, P=0.01]. The most significant difference was for in-patient episodes [£9,092(3,578) vs. £11,047(5,118), 95%CI −3251 to −658, P=0.003] suggesting that the intervention may have had an impact on cardiac healthcare utilization in relation to admissions to hospital
6	Loi et al., 2016	NR	There was significant improvement in global QoL, self-regulation and anxiety in I vs. C (P<0.01).	NR
7	Lotzke et al., 2019	ITT analysis on data not lost to FU<br>Lost to FU 1 wk preop<br>I=8:C=6<br>Lost to FU 3 wks PO<br>I=9:c=9<br><br>18 no FU data at 8 wk<br>14 no FU data at 3 mths<br>17 no FU data at 6 mths	**Between group effect size (ANOVA group x time)**<br>**Pain intensity in back (VAS)**<br>Post intervention: 0.21 (−0.18 to 0.59)<br>3 wks PO: −0.22 (−0.62 to 0.17), P=0.16<br>**Pain intensity in leg (VAS)**<br>Post intervention:I=0.21 (−0.18 to 0.59)<br>3 wks PO: −0.22 (−0.62 to 0.17), P=0.90<br>**Pain catastrophizing (PCS)**<br>Post intervention: −0.09 (−0.47 to 0.30)<br>3 wks PO: 0.00 (−0.39 to 0.40), P=0.20<br>**Oswestry Disability Index 2.0**	**Outcomes reported: Patient reported functioning (PSFS); Fear of movement (TSK); Self-efficacy for exercise (SEES); Steps**<br>**Time spent in MVPA, min/d (total accumulated);Time spent in light physical activity, min/d;Time spent sedentary, min/d; 5 min walk; 15 m walk**<br>**Timed “Up & Go” test; 1-min stair climb; 1-leg stand test: eyes open and closed**<br><br>No difference between groups over time in any between group x time interaction.

	Study	Total number of withdrawals	Clinical outcomes (mean (SD) or n unless otherwise stated)	Intervention-specific outcomes [(n or mean (SD) unless otherwise reported)] and economic evaluations
			Post intervention: 0.13 (−0.26 to 0.51)<br>3 wks PO: −0.08 (−0.47 to 0.31), P=0.19<br>**Anxiety (HADS)**<br>Post-interventtion:0.16 (−0.23 to 0.56)<br>3 wks PO: 0.11 (−0.30 to 0.51), P=0.85<br>**Depressed mood (HADS)**<br>Post intervention: 0.25 (−0.14 to 0.65)<br>3 wks PO: 0.00 (−0.40 to 0.40), P=0.34<br>**Health-related quality of life (EQ-5D Index)**<br>Post intervention:0.57 (0.16–0.98)<br>3 wks PO: −0.18 (−0.57 to 0.22), P=0.01<br>**Health-related quality of life (EQ-5DVAS)**<br>Post intervention:0.02 (−0.37 to 0.41)<br>3 wks PO: −0.18 (−0.58 to 0.22), P=0.53	**Adverse events**<br>I=0:C=0
8	Ma et al., 1996	None	**Severity of pain**<br>Significantly lower on 1st day PO but no difference on 4th day PO<br>**Usage of pain killer**<br>No difference in Dihydroetorphine hydrochloride tablets usage but the C group needed more Diazepam injection P<0.05	**Outcomes reported: Heart rate, BP, serum cortisol level (mmol/L),**<br>**Heart rate:**<br>No data reported but P<0.05 between I and C group 1 day after operation.<br>**BP:**<br>No difference between groups in Systolic & Diastolic pressure at PO day 1 & 4<br>**Serum cortisol level (mmol/L),**<br>PO day 1:<br>I=lower in I vs C group P<0.01<br><br>**State anxiety scores; 1st and 4th day PO**<br>significant differences between groups (P<0.05)<br>**Physical symptoms of anxiety, on the 1st and 4th day PO**<br>Significantly lower in I vs C group (P<0.05)
9	Marinelli et al., 2020<br>Italy	Drop outs<br>I=134(47.3%):<br>C=149 (52.7%)	**LoS, days I=134:C=149**<br>I=12.5(12.0):C=13.6(14.1), P=0.62<br>**PO Complications**<br>I=47.7%:C=55.9%, P=0.48<br>**BPI-physical pain**<br>I=4.3(1.6):C=4.8(2.3), P=0.33<br>**BPI-emotional**<br>I=2.5(1.8):C=3.9(2.4), P=0.02<br>**BPI-operative**<br>I=4.5(2.3):C=5.3(2.5), P=0.22<br>**PHQ-9**<br>I:4.6 (4.2):C=4.2 (3.6), P=0.56<br>**GSES**<br>I:35.0 (5.0):C=34.5 (5.4), P=0.66<br>**MSPSS**<br>I:6.5 (0.9):C=6.6 (0.7), P=0.37	**NR**

	Study	Total number of withdrawals	Clinical outcomes (mean (SD) or n unless otherwise stated)	Intervention-specific outcomes [(n or mean (SD) unless otherwise reported)] and economic evaluations
			**FACIT-F**<br>I:2.7 (10.5):C=43.6 (9.9), P=0.65<br>**FACT-G**<br>I:55.5 (10.0):C= 56.7 (7.0), P=0.47<br>**Brief COPE**<br>I:67.1 (9.7):C= 66.9 (8.7), P=0.94<br>**STAI-Y2**<br>I:31.3 (8.4):C=32.1 (10.6), P=0.62<br>**APAIS**<br>I:14.9 (6.2):C= 15.3 (6.9), P=0.90<br>**STAI-Y1**<br>I:43.1 (13.7):C= 43.4 (12.1), P=0.70<br>**EFFICACY**<br>**I:6.9 (1.7):C=7.1 (2.1), P=0.57**	
10	Medina-Garzon et al., 2019	C=1 (discontinued)	NR	**Outcomes reported: Amsterdam Preoperative Anxiety and Information Scale (APAIS), administered prior to surgery at end of intervention**<br>**APAIS Mean (SD)**<br>although both groups diminished the anxiety score over time, in the I group the difference between both assessment moments is of 4.2 points, while in the C group it is of 1.73 points.<br>The mean score of preoperative anxiety in the post-intervention evaluation was 5 points lower in the I group, compared with the C group, with this difference being statistically significant.
11	Miro et al., 1999	NR	Ppts in both groups were divided into sub-groups (high and low monitoring) based on “coping style” (identified through the Miller Behavioural Style Scale questionnaire, which assesses the information-seeking style of individuals of coping with stressful and threatening events)<br><br>**Pain at 24hrs & 72hrs (standing, walking, moving in bed)**<br>Low monitoring ppts trained in relaxation experienced less pain and performed at a higher level when compared to low monitoring individuals exposed to an attention control intervention.<br>Low monitoring ppts trained in relaxation experienced less pain at 24hrs after surgery (while walking [F(1,84)=59.36, P<0.001] and moving in bed [F(1,84)=35.99, P<0.001], 72hrs after surgery [under all 3 circumstances or conditions: standing up (F(1,84)=44.02, P<0.001), walking (F(1,84)=9.82, P<0.005) and moving in bed (F(1,84)=8.47, P<0.005)]<br>**Pain at FU**<br>3 wks after surgery [F(1,84)=73.40, P<0.0001];<br>Ppts also reported performing a higher activity level both at home [F(1,84)=4.29, P<0.05] and during leisure time [F(1,84)=10.64, P<0.05].	**Outcomes reported: Return to normal daily activities:**<br>There appeared to be a difference between low monitoring and high monitoring ppts, but no formal comparisons between groups was carried out.

	Study	Total number of withdrawals	Clinical outcomes (mean (SD) or n unless otherwise stated)	Intervention-specific outcomes [(n or mean (SD) unless otherwise reported)] and economic evaluations
12	Osinowo et al., 2003	NR	NR	**Outcomes reported: anxiety and depression:**<br>**Anxiety and Depression (HADS)**<br>Significant decrease in anxiety in I1 and I2 vs C (P<0.01)<br>No difference in depression between groups.<br><br>**STAI & BDI Pre-Post surgery (within group data)**<br>No between group differences analysis reported
13	Samnani et al., 2014	Excluded<br>I=2:C=5<br>(poor surgical outcomes)<br><br>Declined<br>I=1:C=0<br>(low pain threshold)	**PO pulmonary complications (PCC)**<br>I=8/113:C=33/111<br>**Abnormal breath sound**<br>I=9/113:C=32/111<br>**Fever for consecutive days**<br>I=9/113:C=8/111<br>**Presence of cough**<br>I=15/113:C=38/111<br>**White colored sputum**<br>I=8/113:C=4/111<br>**Yellow or green colored sputum**<br>I=3/113:C=34/111<br>**Chest X-ray showing collapse or consolidation**<br>I=5/113:C=30/111<br>**Unexplained rise on WBC**<br>I=2/113:C=5/111<br>**P<0.001 for all**	**Outcomes reported: Mobilization**<br>Bed to chair (minutes) & upright mobilization > 10 mins: both quicker in I vs C group (P<0.001)
14	Strøm et al, 2019	Excluded<br>I=9: C=6<br>2 mth PO<br><br>(12 moved, cancelled or changed surgery; 3 withdrew from intervention before surgery due to loss of motivation)	**Los (days [mean, range]) 1:51:C=48**<br>*Imputed SD (mean of all SD results)*<br>*I=4.9 (16.9): C=5.3 (48.54)*<br>**Low Back Pain Rating Scale**<br>No differences were found between the groups in the overall changes at any point after surgery, except on “leg pain right now”, which was better in the control group than the intervention group on day 2 after surgery (p=0.01).	**HADS: 1 day before and 2 days after surgery (median [IQR])**<br>**HADS-A**<br>I=7(4-10): C=6(4-8)<br>I=7(4-10): C=6(3-9)<br>**HADS-D**<br>I= 6(3 to 9):C= 6(3 to 8)<br>I= 8(5 to 12):C= 6 (3 to 9)<br>**ODI (2 days after surgery)**<br>I= 80 (63 to 87): C=76 (59 to 87)<br>**EQ5D (2 days after surgery)**<br>I= 0.5 (0.37 to 0.58): C=0.5 (0.37 to 0.62)<br><br>Results also available for 3 and 6 mths after surgery for all outcomes
15	Vlisides et al, 2019	I= 7 (5 withdrew and 2 had surgery cancelled)<br><br>C=: 2 (ineligible)	**LoS I=23:C=29**<br>*Imputed SD (mean of all SD results)*<br>*I=6.8 (16.9):C=6.4 (48.54)*	PO delirium incidence<br>I= 6 23:C=5 29<br>perioperative cognitive function*<br>There was no significant difference in estimated mean score between groups for any of the tests.
16	Wells et al., 1986	NR	**LoS (days)**<br>I=7.75(5.15):C=11.25(5.77)	**Outcomes reported: Presurgical anxiety (State and Trait anxiety), hospital anxiety and Depression Scale (HADs) PO day 3**

	Study	Total number of withdrawals	Clinical outcomes (mean (SD) or n unless otherwise stated)	Intervention-specific outcomes [(n or mean (SD) unless otherwise reported)] and economic evaluations
			**PO pain**<br>I=2.67(2.22):C=7.17(3.66)<br>**Nurse rating of adjustment**<br>I=10.33 (1.07):C=8.58 (2.54), P<0.05<br>**Analgesic use [units, NR how measured]**<br>I=1.0(0.73):C=1.75(0.96), P=0.08	**Anxiety**<br>significant treatment effect (P<0.002) for state anxiety (I group reported less anxiety) but no between-group difference for trait anxiety<br>**HADS**<br>**I=6.58(4.50):C=24.83(11.40), P<0.0001**<br><br>**Economic effect**<br>Net savings in hospital bills to treat ppts was estimated at $12,600. The intervention cost was estimated to be $1,200.

KEY:BDI=Becks Depression Inventory; BL=baseline; BMI=body mass index; BP=blood pressure; C=control; CCSAC=Canadian Cardiovascular Society Angina Class; CI=confidence interval; CLASP=Cardiovascular Limitations and Symptoms Profile; difference=difference; EQ5D=EuroQol-5D; FU=follow up; HADS=Hamilton anxiety/depression scale; HRQOL=health related quality of life; hrs=hours; I=intervention; ICU=intensive care unit; IQR=interquartile range; ITT=intention to treat; L=litre; kPa=kilopascal; LoS=length of stay; MI=myocardial infarction; mL=millilitre; mmol=millimole; mths=months; n=number; nmol=nanomole; NR=not reported; NYHA=New York Heart Association; pg=picogram; PO=postoperative; ppts=participants; QALY=quality-adjusted life years; QoL=quality of life; sd=standard deviation; SEM=standard error of the mean; STAI=State-Trait anxiety Scale; T1=Time 1; vs.=versus; WBC=whole blood count; wks=weeks

PSYCHOLOGICAL INTERVENTIONS

Table 27. Risk of bias

	Study	Selection bias		Performance bias		Detection bias						Attrition bias	Reporting bias
						Blinding of outcome assessment							
		Random sequence generation	Allocation concealment	Blinding of participants (all outcomes)	Blinding of personnel (all outcomes)	Perioperative mortality, hospital readmission	Postoperative complications	Length of stay	Patient reported outcomes (pain, HRQoL)	Intervention related outcomes	Intervention related adverse events	Incomplete outcome data	Selective reporting
1	Auer 2017; Salzmann 2017 Reif 2017					NR	NR				NR		
2	Cohen 2011					NR			NR		NR		
3	Furze 2009										NR		
4	Gade 2014,2017; Hjelmesæth et al., 2019				protocol	NR	NR	NR		NR	NR		
5	Goodman 2008								NR	NR	NR		
6	Loi 2016 Abstract only					NR	NR	NR		NR	NR		
7	Lotzke 2016					NR	NR	NR					
8	Ma 1996												
9	Marinelli 2020					NR					NR		
10	Medina-Garzon 2019					NR	NR	NR	NR		NR		
11	Miro 1999					NR	NR	NR		NR	NR		
12	Osinowo, 2003					NR	NR	NR	NR		NR		
13	Samnani 2014					NR		NR	NR		NR		
14	Strom 2019					NR				NR	NR		
15	Vlisides 2019					NR	NR	blinding broken	NR	NR	NR		
16	Wells 1986					NR	NR				NR		

NR = not reported

PSYCHOLOGICAL INTERVENTIONS

Table 28. Summary of findings

Psychological interventions compared to usual care for any major surgery

Patient or population: any major surgery
Setting: hospital
Intervention: psychological interventions
Comparison: usual care

Outcomes	Anticipated absolute effects* (95% CI)		Relative effect (95% CI)	№ of participants (studies)	Certainty of the evidence (GRADE)	Comments
	Risk with usual care	Risk with psychological interventions				
Length of Stay (LoS) follow up: 30 days		MD **0.82 days lower** (1.83 lower to 0.19 higher)	-	937 (7 RCTs)	⨁◯◯◯ VERY LOW [a,b,c,d]	

***The risk in the intervention group** (and its 95% confidence interval) is based on the assumed risk in the comparison group and the **relative effect** of the intervention (and its 95% CI).

CI: Confidence interval; **MD:** Mean difference

GRADE Working Group grades of evidence
High certainty: We are very confident that the true effect lies close to that of the estimate of the effect
Moderate certainty: We are moderately confident in the effect estimate: The true effect is likely to be close to the estimate of the effect, but there is a possibility that it is substantially different
Low certainty: Our confidence in the effect estimate is limited: The true effect may be substantially different from the estimate of the effect
Very low certainty: We have very little confidence in the effect estimate: The true effect is likely to be substantially different from the estimate of effect

Explanations

a. Downgrade for risk of bias
b. Downgrade inconsistency: different sizes of CI
c. Downgrade for indirectness: varied interventions
d. Downgrade for imprecision: includes null effect and appreciable benefit or harm, small sample size

Meta analyses

Figure 72. Random effects meta-analysis of the mean difference in length of hospital stay (LoS) between psychological interventions (experimental) and usual care (control).

Study or Subgroup	Experimental Mean	Experimental SD	Experimental Total	Control Mean	Control SD	Control Total	Weight	Mean Difference IV, Random, 95% CI
Auer 2017 EXPECT	12.6	31.6	37	17.3	73.6	22	0.1%	-4.70 [-37.10, 27.70]
Auer 2017; SUPPORT	14.1	23.1	37	17.1	73.6	22	0.1%	-3.00 [-34.64, 28.64]
Furze 2009	7.61	2.69	100	8.28	4.96	104	86.6%	-0.67 [-1.76, 0.42]
Goodman 2008	12.21	10.27	91	20.72	42	90	1.3%	-8.51 [-17.44, 0.42]
Marinelli 2020	12.5	12	134	13.6	14.1	149	11.1%	-1.10 [-4.14, 1.94]
Strom 2019	4.9	16.9	51	5.3	48.54	48	0.5%	-0.40 [-14.89, 14.09]
Vlisides 2019	6.8	16.9	23	6.4	48.54	29	0.3%	0.40 [-18.57, 19.37]
**Total (95% CI)**			**473**			**464**	**100.0%**	**-0.82 [-1.83, 0.19]**

Heterogeneity: Tau² = 0.00; Chi² = 3.05, df = 6 (P = 0.80); I² = 0%
Test for overall effect: Z = 1.59 (P = 0.11)

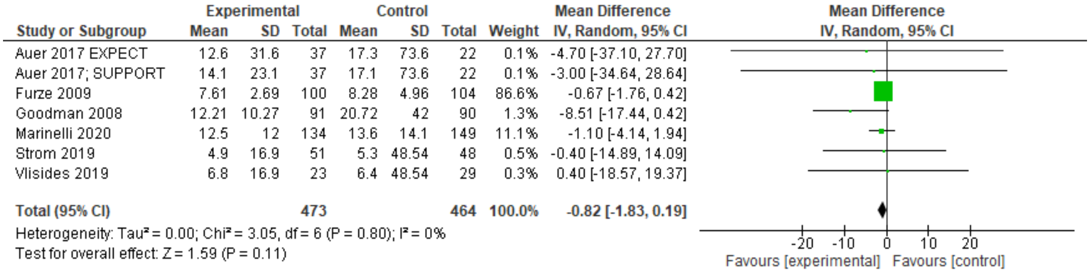

7/15 studies (937 participants). 1 abstract (Vlisides et al., 2019) included.

Sensitivity analyses (removing studies at high risk of bias)

Figure 73. Random effects meta-analysis of the mean difference in length of stay between psychological interventions (experimental) and usual care (control) with high risk of bias studies removed.

Study or Subgroup	Experimental Mean	Experimental SD	Experimental Total	Control Mean	Control SD	Control Total	Weight	Mean Difference IV, Random, 95% CI
Furze 2009	7.61	2.69	100	8.28	4.96	104	87.2%	-0.67 [-1.76, 0.42]
Goodman 2008	12.21	10.27	91	20.72	42	90	1.3%	-8.51 [-17.44, 0.42]
Marinelli 2020	12.5	12	134	13.6	14.1	149	11.2%	-1.10 [-4.14, 1.94]
Vlisides 2019	6.8	16.9	23	6.4	48.54	29	0.3%	0.40 [-18.57, 19.37]
**Total (95% CI)**			**348**			**372**	**100.0%**	**-0.82 [-1.83, 0.20]**

Heterogeneity: Tau² = 0.00; Chi² = 2.97, df = 3 (P = 0.40); I² = 0%
Test for overall effect: Z = 1.57 (P = 0.12)

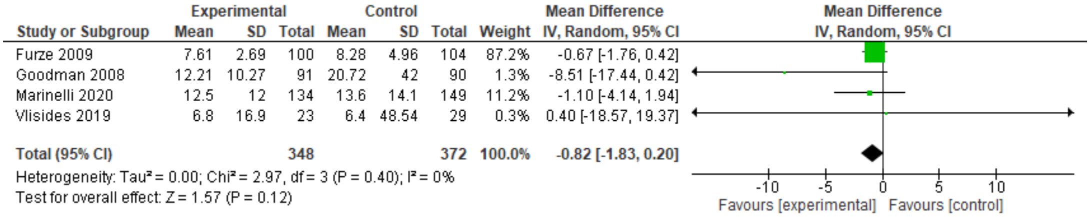

4 studies (720 participants) included and 3 studies excluded (Auer et al., 2017a; Auer et al., 2017b; Strom et al., 2019). 1 abstract (Vlisides et al., 2019) included.

Sensitivity analyses (removing studies with imputed results)

Results were estimated or imputed for LoS in 4 studies (Auer et al., 2017, Goodmand et al., 2008, ; Vlisides et al., 2019; Strom et al., 2019). After excluding results from these studies from the meta-analysis, only 2 studies remained (Furze et al., 2009 & Marinelli et al., 2020).

Subgroup analysis (studies published before and after 2010)

Figure 74. Random effects meta-analysis of the mean difference in length of hospital stay (LoS) between psychological interventions (experimental) and usual care (control) in studies published after 2010.

	Experimental			Control				Mean Difference
Study or Subgroup	Mean	SD	Total	Mean	SD	Total	Weight	IV, Random, 95% CI
Auer 2017 EXPECT	12.6	31.6	37	17.3	73.6	22	0.8%	-4.70 [-37.10, 27.70]
Auer 2017; SUPPORT	14.1	23.1	37	17.1	73.6	22	0.8%	-3.00 [-34.64, 28.64]
Marinelli 2020	12.5	12	134	13.6	14.1	149	91.9%	-1.10 [-4.14, 1.94]
Strom 2019	4.9	16.9	51	5.3	48.54	48	4.0%	-0.40 [-14.89, 14.09]
Vlisides 2019	6.8	16.9	23	6.4	48.54	29	2.4%	0.40 [-18.57, 19.37]
**Total (95% CI)**			**282**			**270**	**100.0%**	**-1.08 [-4.00, 1.84]**

Heterogeneity: Tau² = 0.00; Chi² = 0.09, df = 4 (P = 1.00); I² = 0%
Test for overall effect: Z = 0.73 (P = 0.47)

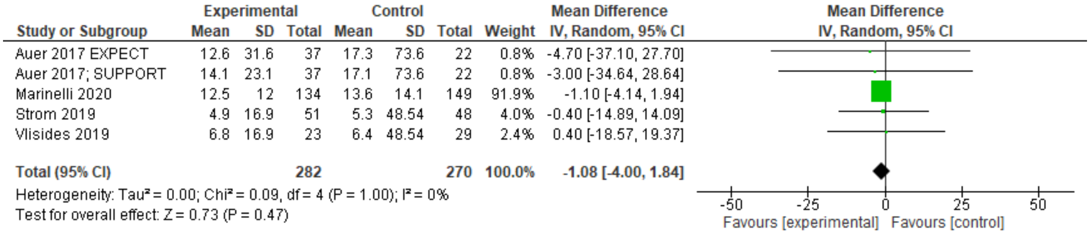

5 studies (552 participants) included and 2 studies excluded (Furze et al., 2009; Goodman et al., 2008). 1 abstract (Vlisides et al., 2019) included.

References

Auer, C. J., Laferton, J. a. C., Shedden-Mora, M. C., Salzmann, S., Moosdorf, R. & Rief, W. 2017. Optimizing preoperative expectations leads to a shorter length of hospital stay in CABG patients: Further results of the randomized controlled PSY-HEART trial. J Psychosom Res, 97, 82-89.

Cohen, L., Parker, P. A., Vence, L., Savary, C., Kentor, D., Pettaway, C., . . . Radvanyi, L. 2011. Presurgical stress management improves postoperative immune function in men with prostate cancer undergoing radical prostatectomy. Psychosom Med, 73, 218-25.

Furze, G., Dumville, J. C., Miles, J. N., Irvine, K., Thompson, D. R. & Lewin, R. J. 2009. "Prehabilitation" prior to CABG surgery improves physical functioning and depression. Int J Cardiol, 132, 51-8.

Gade, H., Friborg, O., Sandbu, R., Rosenvinge, J. & Hjelmesaeth, J. 2017. Long-term follow-up (4 years) of patients receiving cognitive behavioural therapy (CBT) or usual care before bariatric surgery: a randomized controlled trial. Obesity facts, 10, 38-.

Gade, H., Hjelmesæth, J., Rosenvinge, J. H. & Friborg, O. 2014. Effectiveness of a Cognitive Behavioral Therapy for Dysfunctional Eating among Patients Admitted for Bariatric Surgery: A Randomized Controlled Trial. Journal of Obesity, 2014, 127936.

Goodman, H., Parsons, A., Davison, J., Preedy, M., Peters, E., Shuldham, C., . . . Cowie, M. R. 2008. A randomised controlled trial to evaluate a nurse-led programme of support and lifestyle management for patients awaiting cardiac surgery 'Fit for surgery: Fit for life' study. Eur J Cardiovasc Nurs, 7, 189-95.

Hjelmesæth, J., Rosenvinge, J. H., Gade, H. & Friborg, O. 2019. Effects of Cognitive Behavioral Therapy on Eating Behaviors, Affective Symptoms, and Weight Loss After Bariatric Surgery: a Randomized Clinical Trial. Obes Surg, 29, 61-69.

Loi, C. T. T., Ong, C. E., Tang, C. L., Wong, R. K., Chan, S. W. C. & He, H. G. 2016. Effects of a psychoeducation intervention on improving outcomes of patients with colorectal cancer: A pilot randomised controlled trial. Psycho-Oncology, 25, 30-31.

Lotzke, H., Brisby, H., Gutke, A., Hagg, O., Jakobsson, M., Smeets, R. & Lundberg, M. 2019. A Person-Centered Prehabilitation Program Based on Cognitive-Behavioral Physical Therapy for

Patients Scheduled for Lumbar Fusion Surgery - A Randomized Controlled Trial. Physical therapy, 99, 1069-1088.

Ma, Y. L., Qin, L. J. & Han, Z. F. 1996. [Relaxation training on stress response to abdominal surgery]. Zhonghua Hu Li Za Zhi, 31, 377-80.

Marinelli, V., Danzi, O. P., Mazzi, M. A., Secchettin, E., Tuveri, M., Bonamini, D., . . . Del Piccolo, L. 2020. PREPARE: PreoPerative Anxiety REduction. One-Year Feasibility RCT on a Brief Psychological Intervention for Pancreatic Cancer Patients Prior to Major Surgery. Frontiers in Psychology, 11, 362.

Medina-Garzon, M. 2019. Effectiveness of a Nursing Intervention to Diminish Preoperative Anxiety in Patients Programmed for Knee Replacement Surgery: Preventive Controlled and Randomized Clinical Trial. Investigacion y Educacion en Enfermeria, 37.

Miró, J. & Raich, R. M. 1999. Preoperative preparation for surgery: an analysis of the effects of relaxation and information provision. Clinical Psychology & Psychotherapy, 6, 202-209.

Osinowo, H. O., Olley, B. O. & Adejumo, A. O. 2003. Evaluation of the effect of cognitive therapy on perioperative anxiety and depression among Nigerian surgical patients. West Afr J Med, 22, 338-42.

Rief, W., Shedden-Mora, M. C., Laferton, J. A., Auer, C., Petrie, K. J., Salzmann, S., . . . Moosdorf, R. 2017. Preoperative optimization of patient expectations improves long-term outcome in heart surgery patients: results of the randomized controlled PSY-HEART trial. BMC Med, 15, 4.

Salzmann, S., Euteneuer, F., Laferton, J. a. C., Auer, C. J., Shedden-Mora, M. C., Schedlowski, M., . . . Rief, W. 2017. Effects of Preoperative Psychological Interventions on Catecholamine and Cortisol Levels After Surgery in Coronary Artery Bypass Graft Patients: The Randomized Controlled PSY-HEART Trial. Psychosom Med, 79, 806-814.

Samnani, S. S., Umer, M. F., Mehdi, S. H. & Farid, F. N. 2014. Impact of Preoperative Counselling on Early Postoperative Mobilization and Its Role in Smooth Recovery. International scholarly research notices, 2014, 250536-250536.

Strom, J., Nielsen, C. V., Jorgensen, L. B., Andersen, N. T. & Laursen, M. 2019. A web-based platform to accommodate symptoms of anxiety and depression by featuring social interaction and animated information in patients undergoing lumbar spine fusion: a randomized clinical trial. Spine Journal, 19, 827-839.

Vlisides, P. E., Das, A. R., Thompson, A. M., Kunkler, B., Zierau, M., Cantley, M. J., . . . Giordani, B. 2019. Home-based Cognitive Prehabilitation in Older Surgical Patients: A Feasibility Study. Journal of Neurosurgical Anesthesiology, 31, 212-217.

Wells, J. K., Howard, G. S., Nowlin, W. F. & Vargas, M. J. 1986. Presurgical anxiety and postsurgical pain and adjustment: effects of a stress inoculation procedure. J Consult Clin Psychol, 54, 831-5.

SMOKING/ALCOHOL CESSATION INTERVENTIONS

Table 29. Characteristics of studies

ID	1st Author, year and country	Total *n* Intervention (I) Control (c) Number analysed (An if reported)	Patient population, baseline clinical characteristics (mean (SD) or *n* (%) unless otherwise stated	Demographics (mean (SD) or *n* (%) unless otherwise stated)	Intervention	Comparator	Mode of delivery; place of delivery; training level of individuals who delivered the intervention; the number of contacts	Intervention fidelity; Compliance or adherence to intervention
**SMOKING/ALCOHOL CESSATION INTERVENTIONS**								
**SMOKING CESSATION**								
1	Andrews et al., 2006 UK	102, ppts undergoing elective surgery(type of surgery not known) I=51 C=51	NR	NR	Clear advice to stop smoking from a consultant surgeon. The advice was given by letter + standard booklet Approx. 4 wks before surgery	Standard booklet only	Letter; Home; Nurse/surgeon; Wkly for 6-8 wks prior to surgery	NR; NR
2	Lee et al., 2013 USA & Canada	N=168 undergoing any type of elective surgery (including day surgery) I=84 (PO=78 An, 1 mth=73) C=84 (PO=79 An, 1 mth=77)	**Type of surgery N(%)** **Dental** I=3(4):C=1(1) **Head and neck** I=7(8):C=12(14) **General surgery** I=18(21):C=13(15) **Gynecologic** I=11(13):C=12(14) **Ophthalmologic** I=6 (7):C=5 (6) **Plastic** I=4(5):C=5 (6) **Urologic** I=11(13):C=16(19) **Orthopedic, including hand & upper limb** I=24(29):C=20(24)	**Age yrs** I=48(13.2):C=47 (12.3) **Gender (%M)** I=42:C=49 **Cigarettes/day before trial enrollment** I=15(7.5):C=16(10) **No. of yrs smoking before trial enrollment** I=27(14):C=27(1 3) **Fagerström score(out of 10)** I=3.9(2.1):C=4.3 (2.3) **Exhaled CO level (ppm) before**	6-wk supply of free transdermal nicotine replacement therapy (Nicoderm®) along with written medication information + brief (5 min) intervention by a preadmission nurse, brochures, and referral to the Smokers' Helpline which initiated contact (up to 4 attempts) with the ppt, and subsequent counselling was as agreed on by the ppt, but generally aimed to have at least 4 contacts.	Perioperative smoking cessation advice from nurses, surgeons, or anesthesiologists, but no further study-specific smoking cessation intervention. Ppts were not discouraged from using perioperative smoking cessation aids and could still obtain brochures on request.	Face to face + written; Hospital clinic then home; Trained preadmission nurse; 1x nurse + up to 4 contacts from smokers' helpline	NR; NR for compliance/adherence .

ID	1st Author, year and country	Total *n* Intervention (I) Control (c)<br><br>Number analysed (An if reported)	Patient population, baseline clinical characteristics (mean (SD) or *n* (%) unless otherwise stated	Demographics (mean (SD) or *n* (%) unless otherwise stated)	Intervention	Comparator	Mode of delivery; place of delivery; training level of individuals who delivered the intervention; the number of contacts	Intervention fidelity; Compliance or adherence to intervention
**SMOKING/ALCOHOL CESSATION INTERVENTIONS**								
			**Weight (kg)**<br>I=79(16.9):C=77(18.1)<br>**BMI (kg/m²)**<br>I=28.0(4.6):C=27.0(6.2)<br>**Current disease Diabetes N(%)**<br>I=15(18):C=7(8)<br>**Hypertension**<br>I=30(36):C=16(19)<br>**Heart disease**<br>I= 5(6):C=0<br>**COPD or asthma**<br>I=14(17):C=18(21)	**randomization**<br>I=23.1(11.6):C= 21.9 (12.5)				
3	Myles et al., 2004 Australia	N=47<br>I=24 (11 underwent surgery)<br>C=23 (9 underwent surgery)	**Weight (kg)**<br>I=79(21):C=82(19)<br>**Type of surgery General**<br>I=6(25):C=7(30)<br>**Orthopaedic**<br>I=8(33):C=6(26)<br>**Urological**<br>I=2(8):C=0<br>**ENT / faciomaxillary**<br>I=3(13):C=3(13)<br>**Plastics**<br>I=1(4):C=1(4)<br>**Neurosurgery**<br>I=1(4):C=2(9)<br>**Other**<br>I=(13):C=4(17)	**Age yrs**<br>I=45(11):C=40(10)<br>**Gender (%M)**<br>I=62.5:C=70<br>**Smoking behaviour Years smoked**<br>I=28(14):C=22(11)<br>**Daily cigarette consumption N 10–15**<br>I=6:C=4<br>**16–20**<br>I=6:C=4<br>**21–30**<br>I=11:C=12<br>**31–40**<br>I=1:C=1<br>**41–50**<br>I=0:C=2	Bupropion: 150mg single dose daily for 3 days<br>Then 150 mg 2x daily for 7 wks<br><br>Plus booklet 'Smoking and Anaesthesia'	Identical placebo<br><br>Plus booklet 'Smoking and Anaesthesia'	Face to face, self-delivery, written;<br>Hospital then home;<br>Healthcare professional (administered dose);<br>Ppts were given a 3-wk supply of study medication at the first visit, and a further 4-wk supply at visit on day 21	NR:<br>Study drug compliance was higher in the I group, 92 vs. 61%, RR 2.10 (CI=1.28–3.45), p=0.032.

ID	1st Author, year and country	Total *n* Intervention (I) Control (c)<br><br>Number analysed (An if reported)	Patient population, baseline clinical characteristics (mean (SD) or *n* (%) unless otherwise stated	Demographics (mean (SD) or *n* (%) unless otherwise stated)	Intervention	Comparator	Mode of delivery; place of delivery; training level of individuals who delivered the intervention; the number of contacts	Intervention fidelity; Compliance or adherence to intervention
**SMOKING/ALCOHOL CESSATION INTERVENTIONS**								
				**Another smoker in household N**<br>I=14:C=13<br>**Cigar or pipe smoking**<br>I=3:C=4<br>**Previous quit attempts**<br>I=19:C=20<br>**No. of quit attempts**<br>I=3(1–6):C=2(2–3)<br>**Fagerstro¨m score**<br>I=5.6(1.5):C=5.6 (2.0)<br>Median (IQR)<br>I=6(5–7):C=6(5–7)				
4	Nåsell et al., 2010<br>Sweden	N=105 ppts with either lower or upper fracture needing acute surgical treatment<br><br>I=50 (completers=49)<br>C=55 (completers=55)	**Current illness**<br>**Heart disease**<br>I=3(6):C=2(4)<br>**Lung disease**:<br>I=7(14):C=8(15)<br>**Diabetes mellitus**<br>I=3(6):C=2(4)<br>**Depression**:<br>I=11(22):C=10(19)<br>**High BP**<br>I=8(16):C=5(9)<br>**No other disease**<br>I=23(50):<br>C=32(60)	**Age yrs**<br>I=55(2):C=51.5(2)<br>**Gender (%M)**<br>I=26:C=30<br>**Smoking Pack-years**<br>I=21.5(11.8):C=21.5(16.2)<br>**Cigarettes/day**<br>I=12.8(5.7):C=13.2(6.3)	1-2 meetings<br>+ wkly telephone for 6 wks with a nurse	Advice to stop smoking	Letter;<br>Home;<br>Nurse/surgeon;<br>Wkly for 6-8 wks prior to surgery	NR;<br>NR

ID	1st Author, year and country	Total *n* Intervention (I) Control (c)<br><br>Number analysed (An if reported)	Patient population, baseline clinical characteristics (mean (SD) or *n* (%) unless otherwise stated	Demographics (mean (SD) or *n* (%) unless otherwise stated)	Intervention	Comparator	Mode of delivery; place of delivery; training level of individuals who delivered the intervention; the number of contacts	Intervention fidelity; Compliance or adherence to intervention
**SMOKING/ALCOHOL CESSATION INTERVENTIONS**								
5	Pei et al., 2014<br>China<br>(Translation)	N=120<br>Ppts ready for total hip replacement<br>I=60 (56 completers)<br>C=60 (57 completers)	Smoke 20+ cigarettes/day for more than 1 yr<br>Smoking status before trial enrollment (median)<br>I=15 cigarettes/ day with 35-year smoking history<br>C=15 cigarettes/ day with 37-year smoking history<br>**Weight (kg)**<br>I=21(2):C=21(2)<br>**ASA 1/2/3**<br>I=23/31/2:C=22/33/2<br>**Hemachrome (g/L)**<br>I=117(17):C=105(17)<br>**Serum creatinine (µmol/L)**<br>I=97(24):C=91(27)<br>**FEV_1/FVC (%)**<br>I=79(2):C=80(3)	**Overall Age yrs**<br>I=67(8):C=65(9)<br>**Overall Gender (%M)**<br>100	Ppts took the Fagerström test 4 wks before surgery to assess their degree of nicotine addiction. Ppts were provided health education and health theory guide based on the result of Fagerström test. Ppts who scored 6+ in the test were provided Nicorette/Nicotine Patches.	Ppts received usual care (seldom or no provision of any smoking risk information or smoking cessation counselling).	NR;<br>NR;<br>1 Anesthetist;<br>4 wks prior to surgery in I group	NR;<br>Compliance was reported as high in the I group.
**ALCOHOL CESSATION**								
6	Tønnesen and Kehlet, 1999<br>Denmark	42 alcoholic ppts (without liver disease) admitted for elective colorectal surgery	**BMI (kg/m²)**<br>I=24(18-30):C=28(19-32)<br>**Operative procedure: Resection of transverse colon**<br>I=1:C=1	**Age (yrs) median (range)**<br>I=58(37-75):C=61(50-76)<br>**Gender (%M)**<br>I=100:C=84<br>**No of non-smokers**	1 mth of preoperative withdrawal from alcohol and treatment with disulfiram (800 mg disulfiram taken during controlled supervision 2x wkly until wk before surgery).	Allowed continuous drinking + usual care	Verbal & oral;<br>Home (hospital for supervision);<br>Patient-led;<br>2 wkly for 1 mth prior to surgery in I group	NR:<br>All I group ppts completed the withdrawal programme of total abstinence from alcohol, including the 2 ppts who did not require surgery.

ID	1st Author, year and country	Total *n* Intervention (I) Control (c)<br><br>Number analysed (An if reported)	Patient population, baseline clinical characteristics (mean (SD) or *n* (%) unless otherwise stated	Demographics (mean (SD) or *n* (%) unless otherwise stated)	Intervention	Comparator	Mode of delivery; place of delivery; training level of individuals who delivered the intervention; the number of contacts	Intervention fidelity; Compliance or adherence to intervention
**SMOKING/ALCOHOL CESSATION INTERVENTIONS**								
		I=20 (16An)<br>C=22 (19An)<br><br>(did not receive procedure C=1)	**Hemicolectomy**<br>I=2:C=3<br>**Sigmoid resection**<br>I=3:C=4<br>**Low anterior resection**<br>I=4:C=5<br>**Rectal amputation**<br>I=1:C=1<br>**Closure of stoma after Hartmann's resection**<br>I=5:C=5<br>**Diagnosis benign/malignant**<br>I=8/8:C=9/10<br>**Dukes' type A**<br>I=1:C=0<br>**Dukes' type B**<br>I=2:C=8<br>**Dukes' type C**<br>I=5:C=2<br>**Prognostic nutrition index (%)**<br>I=51(14-63):C=30(12-52)<br>**Cardiac risk index (points)**<br>I=3(3-15):C=3(3-10)<br>**Total no treated for chronic diseases:**<br>I=5:C=6	I=7:C=8<br>**Smokers alcohol intake (g/day)**<br>I=20(5-40):C=20(2-30)				

ID	1st Author, year and country	Total *n* Intervention (I) Control (c)<br><br>Number analysed (An if reported)	Patient population, baseline clinical characteristics (mean (SD) or *n* (%) unless otherwise stated	Demographics (mean (SD) or *n* (%) unless otherwise stated)	Intervention	Comparator	Mode of delivery; place of delivery; training level of individuals who delivered the intervention; the number of contacts	Intervention fidelity; Compliance or adherence to intervention
**SMOKING/ALCOHOL CESSATION INTERVENTIONS**								
			**Heart failure**<br>I=2:C=1<br>**Angina pectoris**<br>I=0:C=0<br>**Hypertension**<br>I=1:C=4<br>**Chronic bronchitis**<br>I=1:C=2<br>**Asthma**<br>I=1:C=0<br>**IDDM/NIDDM**<br>I=0:C=0					
7	Tonnesen et al., 2002 Unpublished data (taken from Oppedal et al., 2012 Cochrane review) Denmark	N=28 undergoing elective hip arthroplasty<br>I=15<br>C=13	Eligibility criterion was alcohol consumption exceeding 60 g/day or 420 g/wk. Preoperative alcohol consumption at inclusion was:<br>I=72g (60 to 156g)/day<br>C=72g (60 to 96 g)/day	**Overall**<br>**Age yrs range**<br>39-75<br>**Gender (%M)**<br>100	Aimed at 3 mths of preoperative withdrawal from alcohol, supported by disulfiram 800mg/wk, 400mg taken under supervision, and 400mg taken without supervision. Chlordiazepoxide was offered for withdrawal symptoms.<br>The intervention included motivational counselling together with a brief interview (all together ~30 mins) every wk. Project staff were available for ppts by phone in the daytime. All ppts received B-vitamins	Usual care	Face to face (group) & oral;<br>Home and clinic;<br>Project staff;<br>Every wk for 12 wks	NR;<br>NR

KEY: An=analysed; ASA=American Society of Anaesthesiology physical status classification system; BL=baseline; BMI=body mass index; BP=blood pressure; C=control; CHD=coronary heart disease; CO=Carbon monoxide; CI=confidence interval; COPD=chronic obstructive pulmonary disease; ENT=ear nose and throat; FEV=Forced expiratory volume; g=gram; I=intervention; kg=kilogram; IDDM=insulin dependent diabetes mellitus; NIDDM=non-insulin dependent diabetes mellitus; N or No.=number; NR=not reported; mins=minutes; mth=month; M=male; ppt(s)=participant(s); PO=post-operative; RR=relative risk; Umol/L=micromole/litre; wk(s)=week(s); wkly=weekly; yrs=years

SMOKING/ALCOHOL CESSATION

Table 30. Results

ID	Study	Total number of withdrawals	Clinical outcomes [*n* (%) or mean (SD) unless otherwise stated]	Intervention-specific outcomes [(n or mean (SD) unless otherwise reported)] and economic evaluations
1	Andrews et al., 2005	I=0:C=1	NR	**Outcomes reported: Smoking cessation:**<br>I=18 quitters (6 permanently):C=8 quitters (4 permanently)<br>The RR for intention to quit on a long-term basis was 1.33 (95%CI 0.40–4.46).<br>RR of smoking cessation of 2.21 (95%CI 1.06–4.60)
2	Lee et al., 2013	Lost to FU<br>I=5:C=6	**LoS (days) median (IQR)** 44 ppts undergoing major surgery **I=20:C=24**<br>I=1.75 (1.1-3.1):C= 2.1 (1.4-3.2), P=0.36<br>*LoS days (mean/sd Quantile Estimation (QE) conversion method)*<br>*I=2.47 (2.33):C=2.55 (1.73)*<br>**PO complications (cannot separate major surgery and day surgery ppts)**<br>**Intra-operative**<br>I=5/80:C=6/80, P=1.0<br>**PO**<br>I=2/80:C=5/78, P=0.27<br>**Any time**<br>I=11/84:C=14/84, P=0.67	**Outcomes reported: Smoking cessation, Exhaled CO (ppm), reduced smoking, Cigarettes/day preoperatively, No. of days preoperatively without having smoked**<br>**Smoking cessation**<br>(self-reported continuous abstinence for 7 days preoperatively and biochemical confirmation of point-prevalence cessation with exhaled CO)<br>I=2/84:C=3/84, P=0.027<br>**Inaccurate reported smoking cessation**<br>(exhaled CO >10 ppm despite self-reported 7-day cessation)<br>I=6/84:C=5/84, P=1.00<br>**Exhaled CO on day of surgery**<br>Significantly less in I vs C group (P=0.077)<br>**Reduced smoking**<br>significant reduction in I vs C group (P=0.001)<br>**Cigarettes/day preoperatively as assessed on day of surgery**<br>significantly less in I vs C group (P=0.0002)<br>**No. of days preoperatively without having smoked**<br>I had significantly less days compared to C (P=0.006)

3	Myles et al., 2004	I=13:C=14	**LoS (hours) median (IQR) I=9:C=11**<br>I=34(32-72):C=24(10-50), P=0.14<br>LoS days median (IQR)<br>I=1.4 (1.3-3):C=1 (0.4-2.1)<br>*LoS days (mean/sd Quantile Estimation (QE) conversion method)*<br>*I=2.32 (2.09):C=1.52 (1.62)*<br>**Wound infection**<br>I=1/11:C=1/9, P>0.99<br>**Arterial desaturation** I=0/11:C=1/9, P>0.99<br>**Laryngospasm** I=0/11:C=0/9, NS<br>**Bronchospasm** I=0/11:C=1/9, P>0.99<br>**Cough** I=0/11:C=1/9, P>0.99<br>**Apnoea** I=0/11:C= 1/9, P>0.99<br>**Time spent in PACU**; min<br>I=60 (45–75):C=40 (35–65), P=0.23	**Outcomes reported: quit smoking, cigarettes smoked/day, end - expired carbon monoxide (ppm), Pulse oximetry % 3 wks during intervention, 6 wks during intervention & at admission**<br><br>**FU visit, at 3 wks N: I=18:C=12**<br>**Quit smoking**<br>I=9/18:C=2/12, P= 0.036<br>**Cigarettes smoked/ day**<br>I=0(0–7):C=11(2–15), P=0.019<br>**End-expired CO (ppm)**<br>I=6.8(8.4):C=16.6(8.8), P=0.004<br>**Pulse oximetry** %<br>I=97.4(1.9):C=95.5(1.9), P=0.011<br><br>**FU visit 6 wks N: I=14:C=10**<br>**Quit smoking**<br>I=1/14:C=2/10, P= 0.25<br>**Cigarettes smoked/ day**<br>I=0(0–6):C=12(5–15), P=0.068<br>**End-expired CO (ppm)**<br>I=7.7(7.6):C=26.0(27), P=0.004<br>**Pulse oximetry %**<br>I=97.3(1.2):C=95.4(2.3), P=0.015<br><br>**At hospital admission for surgery N: I=11:C=9**<br>**Quit smoking**<br>I=1/11:C=1/9, P > 0.99<br>**Cigarettes smoked/day median (IQR)**<br>I=6(2–7):C=15(9–20), P=0.046<br>**End-expired carbon monoxidde (ppm)**<br>I=4.5(4.3):C=16.0(7.4), P=0.003<br>**Pulse oximetry %** I=97.6(1.3):C=95.5(1.9), P=0.021
4	Nasell et al., 2010	I=1 (no surgery performed):C=3 (declined participation after inclusion)	**PO complications**<br>**Wound infection:** I=4/49:C=11/55, NS<br>**UTI:** I=2/49:C=3/55, NS<br>**Pneumonia:** I=1/49:C=1/55, NS<br>**Complications related to plaster cast** I=1/49:C=6/55<br>**Pressure ulcers** I=1/49:C=1/55<br>**Fracture re-dislocation** I=1/49:C=1/55<br>**Neurological complication** I=1/49:C=0/55<br>**DVT** I=0/49:C=2/55<br>**Pulmonary embolus** I=0/49:C=1/55, NS<br>**Patients with at least one complication**<br>I=10/49:C=21/55, P=0.048	NR

5	Pei et al., 2014 (Translation)	I=4 excluded (intervention lasted less than 4 wks n=2; cancellation of surgery n=2):C=3 excluded (cancellation of surgery)	**Mortality (In hospital)**<br>I=0/56:C=0/57, NS<br>**LoS days** (Median) IQR NR<br>I=14:C=2, P<0.001<br>**Total PO complications**<br>I=12/56:C=39/57, P<0.001<br>**PO wound complications**<br>I=3/56:C=21/57, P<0.001<br>**Hematoma:**<br>I=1/56:C=4/57, P=0.371<br>**Incision infection:**<br>I=1/56:C=13/57, P=0.001<br>**Deep fascial abscess**<br>I=1/56:C=4/57, P=0.371<br>**Requirement of secondary surgery**:<br>I=1/56:C=9/57, P=0.022<br>• Replacement: I=0/56:C=0/57, NS<br>• Reduction: I=0/56:C=1/57, P=1.000<br>• PO wound debridement: I=1/56:C=8/57, P=0.040<br>**Respiratory insufficiency**<br>I=1/56:C=1/57, P=1.000<br>**Cardiac insufficiency**<br>I=0/56:C=5/57, P=0.070<br>**Renal insufficiency**<br>I=0/56:C=1/57, P=1.000<br>**Confusion**<br>I=1/56:C=5/57, P=0.216<br>**Digestive tract bleeding**<br>I=0/56:C=1/57, P=1.000<br>**UTI**<br>I=5/56:C=6/57, P=0.775<br>**DVT**<br>I=1/56:C=1/57, P=1.000	**Outcomes reported: smoking cessation and reduced smoking**<br>**Prior to surgery:**<br>**Smoking cessation:**<br>I=46/56(82%):C=9/57(16%)<br>**Reduced smoking:**<br>I=10/56(18%):C=0/57(0%)
**Alcohol Cessation**				

**6**	Tønnesen et al., 1999	**Withdrawn:**<br>1=4:C=2<br>**Not operated on:**<br>I=2:C=1<br>**Laparoscopic resection:**<br>I=1:C=1<br>**Prolonged intervention of 3 mths:**<br>I=1:C=0	**Mortality (in hospital)**<br>I=1/16:C=2/19, P=1.00<br>**LoS (days) median (range) I=16:C=19**<br>I=8(3-41):C=10(4-46), NS<br>*LoS (mean/sd) Quantile Estimation (QE) conversion method*<br>*I=14.38 (19.57): C=16.03 (17.88)*<br>**PO complications**<br>**No. of minor complications**<br>I=5/16:C=17/19<br>**Wound infections (requiring surgical intervention)**<br>I=3/16:C=4/19<br>**Wound haematoma (requiring surgical intervention)**<br>I=0/16:C=1/19<br>**Pneumonia (stethoscopy and x ray confirmation)**<br>I=0/16:C=5/19<br>**Thrombophlebitis (venography)**<br>I=1/16:C=0/19<br>**Haematemesis**<br>I=0/16:C=1/19<br>**Subileus (retention >7 days)**<br>I=0/16:C=1/19<br>**Dehydration (readmission for IV rehydration)**<br>I=0/16:C=2/19<br>**Urinary infection (>105 bacteria/ml)**<br>I=1/16:C=2/19<br>**Fistula (external)**<br>I=0/16:C=1/19<br>**No of major complications:**<br>5/16 14/19<br>**Fascial rupture**<br>I=0/16:C=3/19<br>**Intra-abdominal bleeding (requiring surgical intervention and transfusion)**<br>**I=**0/16:C=1/19<br>**Intra-abdominal abscess (drainage)**<br>I=1/16:C=2/19<br>**Anastomotic leakage (surgical intervention)**<br>I=2/16:C=2/19<br>**Ileus (surgical intervention)**<br>I=0/16:C=1/19<br>**Cardiopulmonary insufficiency (intensive care)**<br>I=1/16:C=4/19<br>**Acute myocardial infarction (increased enzymes, ECG changes)**<br>I=0/16:C=0/19<br>**Sepsis (bacteriaemia and fever)**<br>I=1/16:C=1/19<br>**Pulmonary embolism (positive ventilation/perfusion scintigraphy)**<br>I=0/16:C=0/19	**Outcomes reported: alcohol abstinence**<br>All ppts in I group completed the withdrawal programme of total abstinence from alcohol, including the 2 who did not require surgery.<br>The C group continued their drinking habits until surgery.<br>PO to FU (1mth), the alcohol consumption was low in both groups

			**Psychosis (hallucinations requiring pharmacological treatment)**<br>I=0/16:C=0/19<br>**No of ppts with:**<br>**Total complication**<br>I=5/16:C=14/19, P=0.02<br>**Major complications**<br>I=2/16:C=8/19, P=0.07<br>**Minor complications**<br>I=4/16:C=11/19, P=0.09<br>**Ppts who required secondary surgery:**<br>**Intraperitoneal**<br>I=2/16:C=8/19, P=0.07<br>**Superficial**<br>I=3/16:C=4/19, P=1.00<br>The I group developed slightly fewer complications (minor and major), compared with the C group and required slightly less nurse care PO.	
7	Tønnesen et all. 2002	**No withdrawals**	**30-day mortality**<br>I=0/15:C=1/13<br>**LoS days I=15:C=13**<br>I=15(6.1):C=15(5.5)<br>**Total Complications (1 mth PO)**<br>I=4/15:C=7/13	**Outcomes reported: alcohol abstinence**<br>9/10 ppts in I group stopped drinking and 1/10 reduced the alcohol intake from 5 to 1 alcohol units/day.<br>In the C group alcohol consumption was reported to be unchanged.

KEY: CI=confidence interval; C=control; CO=carbon monoxide; CV=cardiovascular; DVT=deep vein thrombosis; ECG=electrocardiograph; FU=follow up; GI=gastrointestinal; I=intervention; LoS=length of stay; No.=Number; NS=non-significant; NR=not reported; ppm = parts per million; PACU=post-anaesthesia care unit. PO=post-operative; ppm=parts per million; ppts=participants; RR=relative risk; UTI=urinary tract infection

Perry R, *et al. BMJ Open* 2022; 11:e050806. doi: 10.1136/bmjopen-2021-050806

SMOKING/ALCOHOL CESSATION

Table 31. Risk of bias

	**Study**	Selection bias		Performance bias	Performance bias	Detection bias						Attrition bias	Reporting bias
		Random sequence generation	Allocation concealment	Blinding of participants (all outcomes)	Blinding of personnel (all outcomes)	Blinding of outcome assessment						Incomplete outcome data	Selective reporting
						Perioperative mortality, hospital readmission	Postoperative complications	Length of stay	Patient reported outcomes (pain, HRQoL)	Intervention related outcomes	Intervention related adverse events		
**Smoking Cessation**													
1	Andrews 2005					**NR**	**NR**	**NR**	**NR**		**NR**		
2	Lee 2013					**NR**			**NR**		**NR**		
3	Myles 2004					**NR**			**NR**		**NR**		
4	Nasell 2010					**NR**		**NR**	**NR**	**NR**	**NR**		
5	Pei 2014					**NR**		**NR**	**NR**		**NR**		
**Alcohol Cessation**													
6	Tonnesen 1999								**NR**		**NR**		
7	Tonnesen 2002								**NR**		**NR**		

NR = not reported

Perry R, *et al. BMJ Open* 2022; 11:e050806. doi: 10.1136/bmjopen-2021-050806

SMOKING CESSATION

Table 32. Summary of findings

Smoking cessation compared to usual care for any major surgery

Patient or population: any major surgery
Setting: hospital
Intervention: smoking cessation
Comparison: usual care

Outcomes	Anticipated absolute effects* (95% CI)		Relative effect (95% CI)	№ of participants (studies)	Certainty of the evidence (GRADE)	Comments
	Risk with usual care	Risk with smoking cessation				
Wound infection follow up: 30 days	275 per 1,000	**77 per 1,000** (33 to 176)	**RR 0.28** (0.12 to 0.64)	236 (3 RCTs)	⨁◯◯◯ VERY LOW [a,b,c]	

***The risk in the intervention group** (and its 95% confidence interval) is based on the assumed risk in the comparison group and the **relative effect** of the intervention (and its 95% CI).

CI: Confidence interval; **RR:** Risk ratio

GRADE Working Group grades of evidence
High certainty: We are very confident that the true effect lies close to that of the estimate of the effect
Moderate certainty: We are moderately confident in the effect estimate: The true effect is likely to be close to the estimate of the effect, but there is a possibility that it is substantially different
Low certainty: Our confidence in the effect estimate is limited: The true effect may be substantially different from the estimate of the effect
Very low certainty: We have very little confidence in the effect estimate: The true effect is likely to be substantially different from the estimate of effect

Explanations

a. Downgrade for risk of bias
b. Downgrade for inconsistency: different sizes of CI
c. Downgrade for imprecision: small sample size

Meta analyses

Figure 75. Random effects meta-analysis of the risk ratio of wound infection between smoking cessation interventions (experimental) and usual care (control)

Study or Subgroup	Experimental Events	Experimental Total	Control Events	Control Total	Weight	Risk Ratio M-H, Random, 95% CI
Myles 2004	1	11	1	8	9.6%	0.73 [0.05, 9.97]
Nssall 2010	4	49	11	55	47.6%	0.41 [0.14, 1.20]
Pei 2014	3	56	21	57	42.7%	0.15 [0.05, 0.46]
**Total (95% CI)**		**116**		**120**	**100.0%**	**0.28 [0.12, 0.64]**
Total events	8		33			

Heterogeneity: Tau² = 0.07; Chi² = 2.28, df = 2 (P = 0.32); I² = 12%
Test for overall effect: Z = 3.03 (P = 0.002)

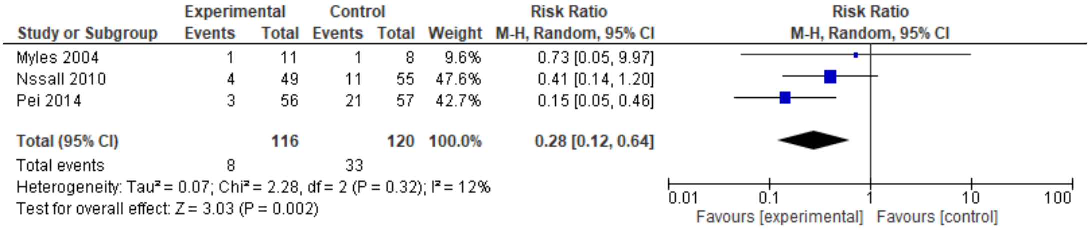

3/5 RCTs (236 participants).

Sensitivity analyses (removing studies at high risk of bias)

No studies were rated as having high risk of bias, so there was no need to conduct these sensitivity analyses.

Sensitivity analyses (removing studies with imputed results)

SMOKING CESSATION

Results were estimated or imputed for LoS in 1 study (Myles et al., 2004). Excluding this study left only one study (Lee et al., 2013) which did not demonstrate an effect of the intervention: difference in LoS between treatments was -0.08 days (95%CI, -1.31, 1.15, P-0.90).

ALCOHOL CESSATION

Results were estimated or imputed for LoS in 1 study (Tønnesen and Kehlet, 1999). Excluding this study left only one study (Tonesen et al, 2002)

Subgroup Analyses

These were not possible becaue there were insuffucinet studies available.

References

Andrews, K., Bale, P., Chu, J., Cramer, A. & Aveyard, P. 2006. A randomized controlled trial to assess the effectiveness of a letter from a consultant surgeon in causing smokers to stop smoking pre-operatively. *Public Health,* 120**,** 356-8.

Lee, S. M., Landry, J., Jones, P. M., Buhrmann, O. & Morley-Forster, P. 2013. The effectiveness of a perioperative smoking cessation program: a randomized clinical trial. *Anesth Analg,* 117**,** 605-13.

Myles, P. S., Leslie, K., Angliss, M., Mezzavia, P. & Lee, L. 2004. Effectiveness of bupropion as an aid to stopping smoking before elective surgery: a randomised controlled trial. *Anaesthesia,* 59**,** 1053-8.

Nåsell, H., Adami, J., Samnegård, E., Tønnesen, H. & Ponzer, S. 2010. Effect of smoking cessation intervention on results of acute fracture surgery: a randomized controlled trial. *J Bone Joint Surg Am,* 92**,** 1335-42.

Oppedal, K., Møller, A. M., Pedersen, B. & Tønnesen, H. 2012. Preoperative alcohol cessation prior to elective surgery. *Cochrane Database Syst Rev***,** Cd008343.

Pei, H., Zhang, L., Zeng, L. & Yu, F. 2014. Effect of preoperative smoking intervention on postoperative complications of total hip replacement [Chinese]. *Chinese Journal of Evidence-Based Medicine,* 14**,** 399-403.

Tønnesen, H. & Kehlet, H. 1999. Preoperative alcoholism and postoperative morbidity. *Br J Surg,* 86**,** 869-74.

PHARMACOLOGICAL INTERVENTIONS

Figure 76. Characteristics of studies

ID	1st Author, year and country	Total *n* randomized Intervention (I):Control (C)<br><br>Number analysed (An)if reported	Patient population, baseline clinical characteristics (mean (SD) or *n (%)* unless otherwise stated	Demographics (mean (SD) or *n (%)* in each group, unless otherwise stated)	Intervention	Comparator	Mode of delivery; place of delivery; training level of individuals who delivered the intervention; the number of contacts	Intervention fidelity; Compliance or adherence to intervention
1	Anceschi et al., 2010 Italy	144 ppts with benign prostatic hyperplasia undergoing transurethral resection of the prostate/ open prostatectomy I=70:C=70	NR	**Overall age yrs [range (median)]** 50-80 (67) **Gender (M%)** 100% due to condition	*Serenoa repens* (Permixon®) at a dose of 320mg daily (oral) for 2 mths before surgery	Usual care	Self-delivery (oral);<br>Self-administered at home;<br>NR;<br>None	NR;<br>NR
2	Chello et al., 2005, Chello et al., 2006 Italy	40 ppts undergoing elective CABG surgery I=20:C=20	**Hypertension**: I=9 (45):C=11(55) **NYHA class, n (%)** I= 2 (10): 2 (10) II=12 (60) 11 (55) III =6 (30) 7 (35)	**Age yrs** I=66.7 (7.7) C=63.7 (7.1) **Gender (M/%)** I=80:C=75 *Age/ gender differed in Chello 2005.*	20mg/d of atorvastatin 3 wks before surgery	Placebo 3 wks before surgery	Self-delivery;<br>Self-administered at home;<br>NR;<br>NR	NR;<br>NR
3	Codina et al., 2020 Spain Abstract	298 ppts undergoing cardiac surgery I=174:C=124	**Diabetes (%)** I=25:C=25 **Hypertension (%)** I=74:C=70 **COPD (%)** I=18:C=14.5 **CKD I=18:C=13**	**Age yrs** I=64.5 (15.9) C=67.3 (31.4) **Gender (M%)** I=66:C=59	Nephrologist management and control of potential risk factors of renal disease for 1 month before surgery (optimizing hydration state, removing or minimizing dose of drugs that potentially deteriorate kidney function and correcting metabolic disorders)	Usual care	NR (assumed by healthcare professionals);<br>NR (assumed in hospital);<br>NR (assumed intervention delivered by highly trained healthcare professionals)	NR;<br>NR
4	Marwick et al., 2009 Australia	400 ppts undergoing major non-cardiac surgery I=197:C=203	**Risk factors% Diabetes M** I=30:C=24 **Hyperlipidemia** I=64:C=69 **Hypertension** I=81:C=84	**Age yrs** I=73 (8) C=72 (8) **Gender (%M)** I=62:C=66 **Smokers %** I=52:C=42	Universal Beta Blockage Algorithm based on nurse-led strategy to optimize dosing and adherence to bisoprolol titration over a week preoperatively	Usual care (beta blockers are continued in those already taking them or prescribed for those with ischemia)	Self-delivery;<br>NR<br>NR:<br>NR	NR;<br>NR

ID	1st Author, year and country	Total *n* randomized Intervention (I):Control (C)<br><br>Number analysed (An)if reported	Patient population, baseline clinical characteristics (mean (SD) or *n (%)* unless otherwise stated	Demographics (mean (SD) or *n (%)* in each group, unless otherwise stated)	Intervention	Comparator	Mode of delivery; place of delivery; training level of individuals who delivered the intervention; the number of contacts	Intervention fidelity; Compliance or adherence to intervention
5	Nouri-Majalan et al., 2009 Iran	60 ppts undergoing CABG surgery I=30:C=30	**Diabetes M N**<br>I=11:C=18<br>**Hypertension N**<br>I=14:C=18	**Age yrs**<br>I=65 (9.5)<br>C=61 (7.9)<br>**Gender (M%)**<br>I=57:C=48<br>**Smoking**<br>I=11:C=4	Allopurinol and Vitamin E supplement (100units Vit E 4x/ day; 100mg allopurinol 2x/day) 3 to 5 days before surgery	Usual care	Oral;<br>NR, assumed at home;<br>In hospital so assumed 4x day for 3-5 days	NR;<br>NR

KEY: An=analysed; CABG=coronary artery bypass graft; C=control; COPD=chronic obstructive pulmonary disease; CKD=chronic kidney disease; I=intervention; M=male; mth(s)=month(s); n = number; NA=not available; NR=not reported; ppt=participants; SD=standard deviation; wk(s)=week(s); yr(s)=year(s);

Perry R, *et al. BMJ Open* 2022; 11:e050806. doi: 10.1136/bmjopen-2021-050806

PHARMACOLOGICAL INTERVENTIONS

Figure 77. Results

ID	Study	Total number of withdrawals	Clinical outcomes (mean (SD) or n unless otherwise stated)	Intervention-specific outcomes [(n or mean (SD) unless otherwise reported)] and economic evaluations
1	Anceschi et al., 2010	Lost to FU<br>I=24/70<br>C=14/70	**LoS (days) (SD NR)**<br>I=5.92: C=9.32<br>**LoS (days (median range)**<br>I=6 (3-9):C=7 (3-19)<br>**PO complications (non-infective)**<br>I=0/46: C=8/56 (5 hemorrhage, 1 anemia, 2 haematuria)	**Outcomes reported: transfusion needs, hemoglobin, hematocrit, RBC, WBC, platelets**<br>Transfusion needs were significantly lower in I vs C P<0.001<br>NS difference in hemoglobin, hematocrit, RBC, WBC, platelets.<br>NS difference in length/amount of drainage between gps<br><br>Paper stated that there were no adverse reactions to *Serenoa repens.*
2	Chello et al., 2005;2006	None reported	**Chello et al, 2005 & 2006:**<br>**MI**<br>I=0/20: C=0/20<br>**Bleeding**<br>I=0/20: C=1/20<br>**Chello et al 2006 only:**<br>**Perioperative mortality**<br>I=0/20: C=0/20, NS<br>**Mean PO stay days, [mean (SD)]**<br>I=6.9 (1.0): C=7.2 (0.9), P=0.4<br>**Mean ICU stay days, [mean (SD)]**<br>I=1.9 (0.6): C=2.1(0.4), P=0.36<br>**Infective complications (sepsis)**<br>I=0/20: C=1/20 P=1.0<br>**Stroke**<br>I=0/20: C=0/20, NS<br>**Renal insufficiency**<br>I=1/20: C=1/20, NS<br>**Atrial fibrillation**<br>I=2/20: C=5/20, P=0.4<br>**Inotropic support**<br>I=5/20: C=6/20, P=1.0	Chello et al 2005<br>In statin vs placebo: no effect on endothelial function (brachial artery diameter, endothelial-dependent flow mediated dilatation and endothelial-dependent nitroglycerin induced dilatation)<br><br>Chello et al 2006<br>In statin vs placebo: significant decrease (p<0.05) in systemic inflammatory response; significant decrease (p<0.05) in interleukin 8 and 6 at 4hrs PO; sig. decrease (p<0.05) in neutrophil expression at 4hrs and 24hrs PO; sig. decrease in adhesion to saphenous vein endothelial cells 4hrs PO; no decrease in TNF alpha. Data reported on graphs.
3	Codina et al., 2020 Spain	NR	**Mortality** (assumed in hospital but not reported)<br>I=1/174:C=3/124<br>No difference in acute kidney injury between groups but numbers not reported.	**Outcomes reported: serum creatinine and albuminuria at 1 yr**<br>Creatinine, mean (SD): I=91.9 (30.8) μmol/L and C=87.1 (23.7) μmol/L (p=0.292)<br>No differences between groups in albuminuria.
4	Marwick et al., 2009	NR	**major CE (cardiac death or myocardial infarction) within 30 days**<br>I=13/197:C=12/203<br>(OR= 1.12, 95% CI 0.52-2.39)	**Outcomes reported: Heart rate control**<br>Those randomized to I group had significantly better heart rate control perioperatively, at the cost of bradycardia and hypotension

5	Nouri-Majalan et al., 2009	None	**Mortality (in hospital)**<br>I=0/30: C=0/30 NS<br>**ICU stay days**<br>I=2.6 (0.75): C=3.9 (1.54), P<0.0001<br>**Adverse Events**<br>Ejection fraction I=44 (10.9): C=45 (10.4), P=0.74<br>Acute renal failure (no.) I=5: C=4, P=0.5<br>Received dopamine infusion (no.) I=8: C=5, P=0.65<br>Hypotension (systolic BP 90 mm Hg) (no.) I=1: C=6, P=0.034<br>Arrhythmia (no.) I=12: C=18, P=0.56	**Outcomes reported: PO Serum levels ((Potassium, Creatine, Creatine clearance):**<br>No significant difference in PO serum levels

KEY: BP=blood pressure; C=control; FU=follow up; I=intervention; ICU=intensive care unit; LoS=length of stay; NS=non-significant; OR= odds ratio; PO=post-operative; RBC = red blood cell; SD=standard deviation; vs=versus; WBC=white blood cell

PHARMACOLOGICAL INTERVENTIONS

Figure 78. Risk of bias

	Study	Selection bias		Performance bias		Detection bias						Attrition bias	Reporting bias
						Blinding of outcome assessment							
		Random sequence generation	Allocation concealment	Blinding of participants (all outcomes)	Blinding of personnel (all outcomes)	Perioperative mortality, hospital readmission	Postoperative complications	Length of stay	Patient reported outcomes (pain, HRQoL)	Intervention related outcomes	Intervention related adverse events	Incomplete outcome data	Selective reporting
1	Anceschi 2010				NR			NR					
2	Chello 2005; 2006						NR	NR	NR	NR			
3	Codina 2020 abstract only							NR	NR		NR		
4	Marwick 2009							NR	NR		NR		
5	Nouri-Maialan 2009						NR	NR	NR				

NR = not reported

Meta analyses

There were not enough studies to conduct pooled analyses for mortality, Length of stay or PO complications

References

Anceschi, R., Bisi, M., Ghidini, N., Ferrari, G. & Ferrari, P. 2010. Serenoa repens (Permixon®) reduces intra- and postoperative complications of surgical treatments of benign prostatic hyperplasia. *Minerva Urol Nefrol,* 62**,** 219-23.

Chello, M., Goffredo, C., Patti, G., Candura, D., Melfi, R., Mastrobuoni, S., . . . Covino, E. 2005. Effects of atorvastatin on arterial endothelial function in coronary bypass surgery. *Eur J Cardiothorac Surg,* 28**,** 805-10.

Chello, M., Patti, G., Candura, D., Mastrobuoni, S., Di Sciascio, G., Agrò, F., . . . Covino, E. 2006. Effects of atorvastatin on systemic inflammatory response after coronary bypass surgery. *Crit Care Med,* 34**,** 660-7.

Codina, S., Coloma, A., Sbraga, F., Boza, E., Vazquez-Reveron, J. M., Ferreiro, E., . . . Montero, N. 2020. SO020NEPHROLOGY INTERVENTION IN PATIENTS AWAITING CARDIAC SURGERY: A RANDOMISED CONTROLLED TRIAL. *Nephrology Dialysis Transplantation,* 35.

Marwick, T. H., Branagan, H., Venkatesh, B. & Stewart, S. 2009. Use of a nurse-led intervention to optimize beta-blockade for reducing cardiac events after major noncardiac surgery. *Am Heart J,* 157**,** 784-90.

Nouri-Majalan, N., Ardakani, E. F., Forouzannia, K. & Moshtaghian, H. 2009. Effects of allopurinol and vitamin E on renal function in patients with cardiac coronary artery bypass grafts. *Vasc Health Risk Manag,* 5**,** 489-94.

.

Funnel plots

Length of stay

Figure 79. Funnel Plot (Immunonutrition studies)

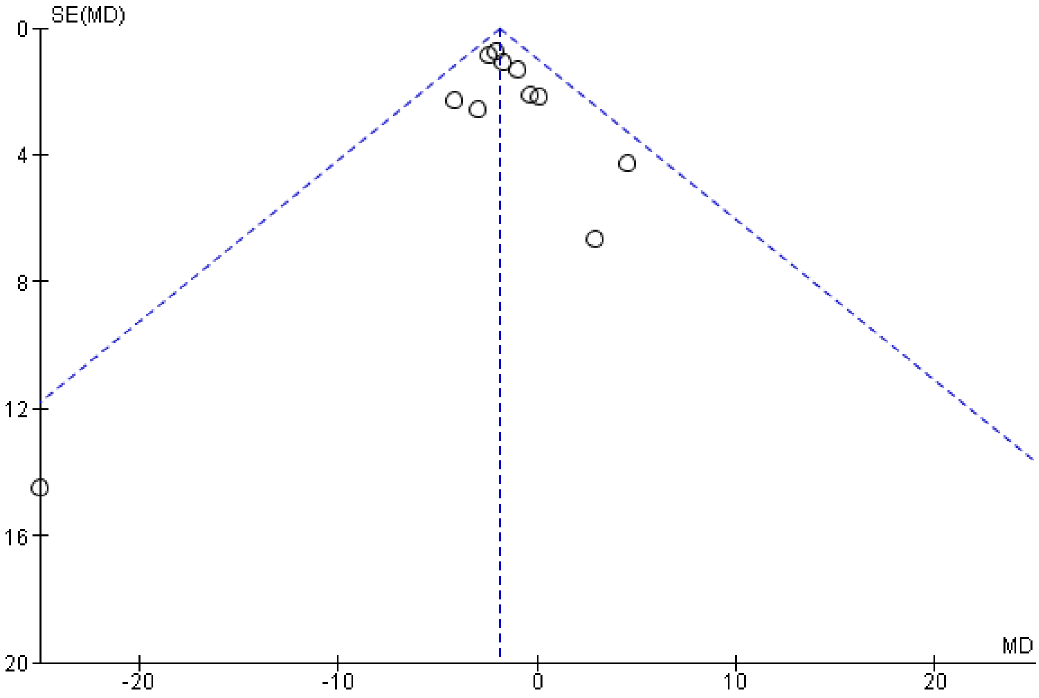

Figure 80. Funnel Plot (Exercise studies)

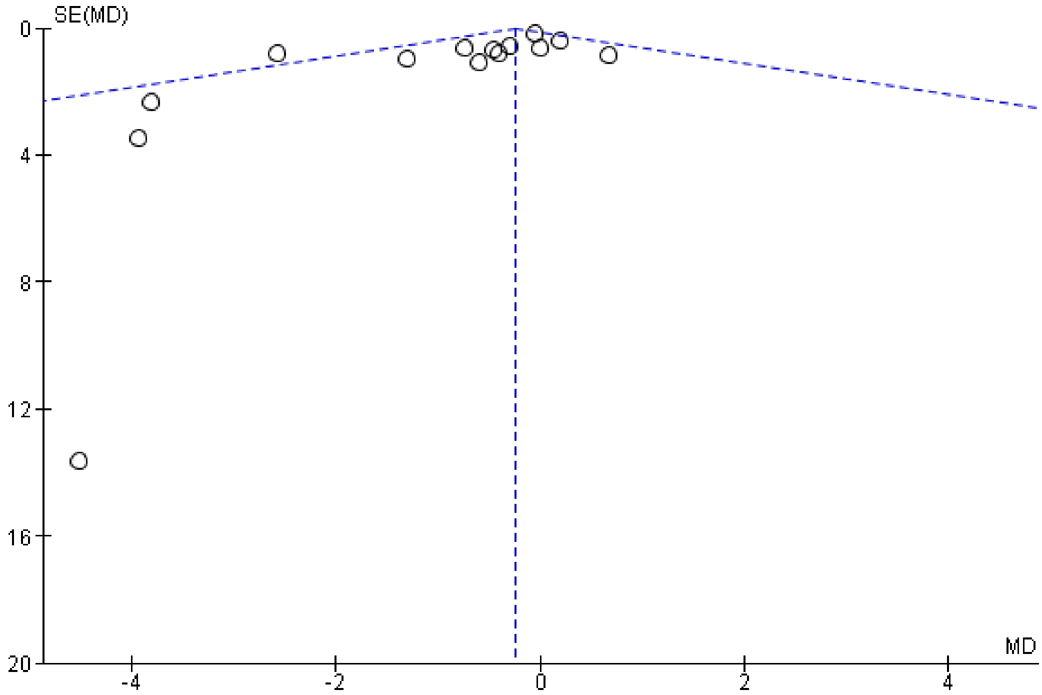

Figure 81. Funnel plot (Multimodal studies)

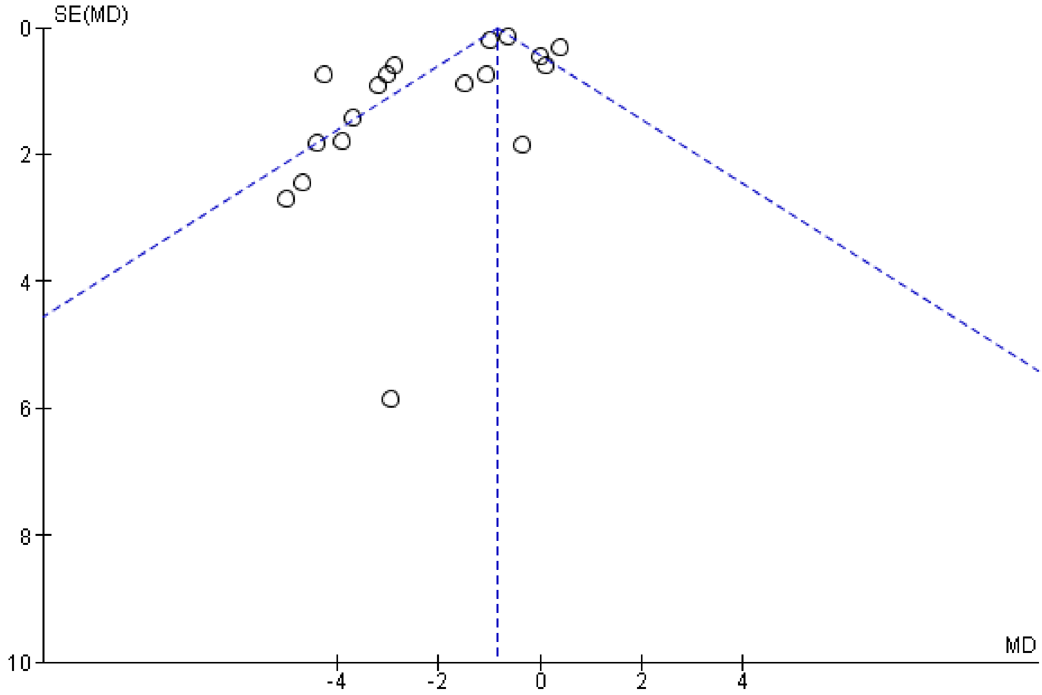

Perry R, *et al. BMJ Open* 2022; 11:e050806. doi: 10.1136/bmjopen-2021-050806

Figure 82. Funnel Plot (Education studies)

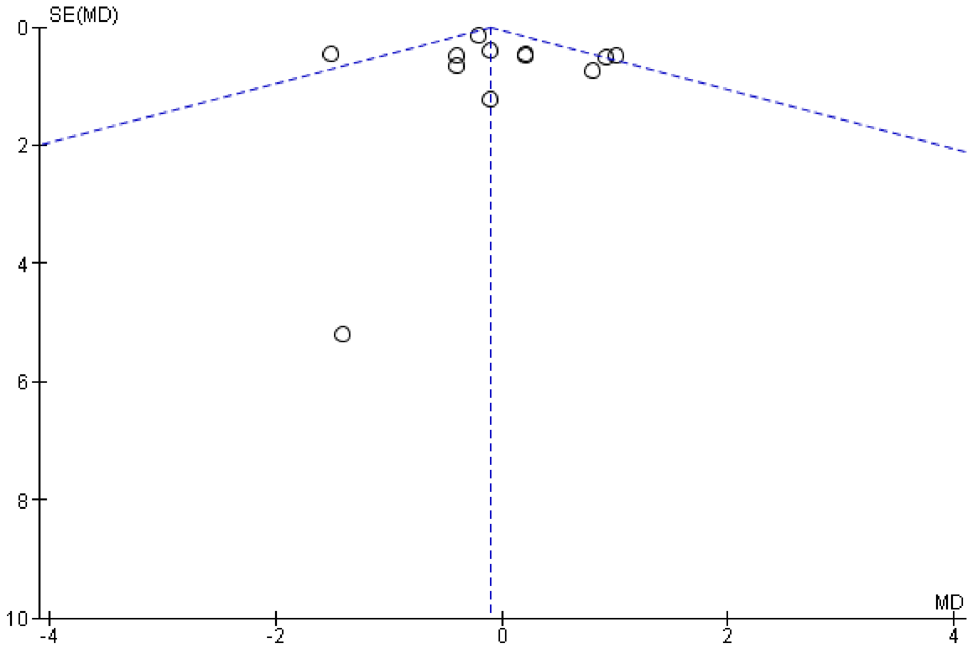

Figure 83. Figure Funnel Plot (IMT studies)

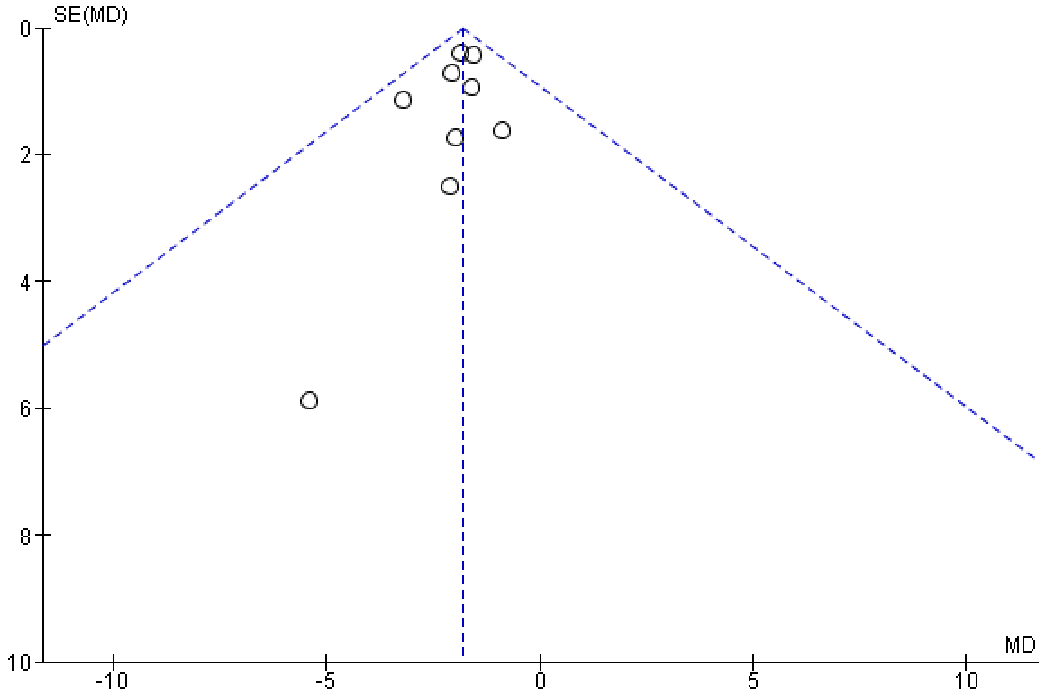

Table 33. Table of excluded studies

	1st Author	Reason for exclusion
1)	Ahn 2015	Intervention was ERAS
2)	Aksu 2018	Intervention administered pre- and postoperatively; relaxation therapy
3)	Almansob 2012	Intervention administered pre- and postoperatively; statin therapy
4)	Aljabari 2018	Comparator not usual care
5)	Amaravati 2013	Intervention administered pre- and postoperatively; intervention not aimed at improving perioperative outcomes; exercise
6)	Anbar 2014	Intervention administered pre- and postoperatively; weight loss intervention
7)	Arango-Gutierrez 2019	Not major surgery
8)	Arvidsson 1982	Intervention administered pre- and postoperatively; respiratory rehabilitation
9)	Ashida 2019	Comparator not usual care
10)	Baillot 2016	No assessment of perioperative outcomes
11)	Bal-bolchenska 2009	Intervention administered pre- and postoperatively (POLISH study)
12)	Banerjee 2015	Surgery specific intervention; no outcomes of interest
13)	Baptiste 2017	No outcomes of interest
14)	Basen-Enqguist 2009	No assessment of perioperative outcomes
15)	Behrend 2019	Intervention administered pre- and postoperatively; nutrition
16)	Bitterli 2009	Surgery specific intervention
17)	Broadbent 2012	Intervention administered pre- and postoperatively; relaxation therapy
18)	Brown 2014	Intervention aimed at improving functional outcomes (specific to TKR) not perioperative outcomes
19)	Burnard 2016	Day surgery only linked to Lahari 2014
20)	Castillo 1985	Intervention administered pre- and postoperatively; respiratory rehabilitation
21)	Carli 2010	Compared 2 prehabilitation interventions
22)	Carli 2020	Compared 2 prehabilitation interventions
23)	Chevillion 2015	Within hospital and < 2 days
24)	Cheung 2003	Intervention administered on day of surgery
25)	Cho 2008	Protocol paper; results published in Cho 2014 but study design changed from RCT to case control
26)	Christensen 2019	Not an RCT

27)	Coats 2013	Not an RCT
28)	Contreras 2018	Compared 2 prehabilitation interventions.
29)	Cordeiro 2019	Intervention administered pre- and postoperatively; IMT
30)	Dao 2011	Intervention administered pre- and postoperatively; psychological
31)	Darnell 2019	No surgical outcomes reported and first timepoint for data collection 2 wks after surgery
32)	das Nair 2018	Intervention not aimed at improving perioperative outcomes
33)	de Heer 2018	Not an RCT
34)	de Wit 1996	Intervention not aimed at improving perioperative outcomes
35)	Dettling 2013	Not an RCT
36)	Dinido 2018	Intervention not aimed at improving perioperative outcomes
37)	Dreyer 2018	Intervention administered pre- and postoperatively; nutrition
38)	Erdem 2018	Compared 2 prehabilitation interventions
39)	Edwards 2009	intravenous iron – in hospital
40)	Farquharson 2011a	Intervention administered pre- and postoperatively; fish oil nutrition
41)	Farquharson 2011b	Intervention administered pre- and postoperatively; fish oil nutrition
42)	Feltrim 2007	Letter connected to Hulzebos study
43)	Finco 2007	Intervention administered pre- and postoperatively; immunonutrition
44)	Foley 2016	Intervention not aimed at improving perioperative outcomes
45)	Furukua 2012	Intervention administered pre- and postoperatively in hospital; immunonutrition
46)	Furukua 2014	Intervention administered pre- and postoperatively in hospital; immunonutrition
47)	Giger 2007	Enteral feed oral
48)	Giger-Pabst 2013	Not usual care for control group
49)	Giles 2019	Editorial not RCT
50)	Gillis 2014	Intervention administered pre- and postoperatively; multimodal
51)	Gianetti 2017	Intervention administered in hospital, but not aimed at improving perioperative outcomes
52)	Gocen 2004	Surgery specific
53)	Grant 2017	Intervention administered < 2 days before surgery; but not aimed at improving perioperative outcomes
54)	Grawe 2010	Intervention administered 24 hrs before surgery
55)	Heidarsdottir 2010	Intervention administered pre- and postoperatively; fish oil nutrition
56)	Hermann 2013	Intervention not aimed at improving perioperative outcomes
57)	Hermann 2014	Intervention not aimed at improving perioperative outcomes
58)	Hogan 2019	Comparator not usual care

59)	Holsgaard-Larsen 2018	Not an RCT
60)	Horchner 1999	Education about the surgery not risk factors for surgery
61)	Iskender 2019	Intervention administered in hospital
62)	Ishikowa 2009	Intervention administered pre- and postoperatively; oral nutrition
63)	Jahic 2018	No outcomes of interest
64)	Jensen 2015	Intervention administered pre- and postoperatively; exercise (linked to Jensen 2016, 2017)
65)	Jensen 2016	Intervention administered pre- and postoperatively; exercise
66)	Jensen 2017	Jensen 2017 is about stoma care, not an intervention designed to improve surgical complications
67)	kakaei 2019	Intervention administered pre- and postoperatively; probiotics
68)	Kale 2018	Intervention not aimed at improving perioperative outcomes
69)	Kalogianni 2016	Intervention administered in hospital
70)	Kara 2020	Intervention given within 48hr of Surgery
71)	Keen 2018	Study protocol (patients not undergoing major surgery)
72)	Keeler 2017	Comparator not usual care
73)	Kim 2014	Not an RCT
74)	Kinoshita 2019	Editorial not an RCT
75)	Kitamura 2018	Not a Prehabilitation intervention
76)	Klaiber 2018	Within hospital <24hrs
77)	Kumar 2016	Compared 2 prehabilitation interventions
78)	Laferton 2015	Intervention not aimed at improving perioperative outcomes
79)	Lahari 2014	See Burnard 2016 (Day surgery only)
80)	Larson 2000	Intervention not aimed at improving perioperative outcomes
81)	Lee CH 2016	Intervention given within 48hrs of surgery ; intervention administered in hospital
82)	Lee CH 2018	Patients not undergoing major surgery
83)	Lee SM 2018	Comparator not usual care
84)	Lee JS 2018	Intervention administered 24 hrs before surgery
85)	Lenz 2000	Not prehabilitation (post-operative intervention)
86)	Leong 2010	Intervention administered pre- and postoperatively; nutrition (seems to be same ppts as Rosenfeldt et al 2011, but different intervention)
87)	Lewis 2018	Comparator not usual care
88)	Liang 2018	Not major surgery
89)	Ligibel 2017	Abstract only -no data. No time points specified.

90)	Liljensøe 2019	Intervention administered pre- and postoperatively
91)	Lim 2018	Review not RCT
92)	Lindstrom 2010	Pre-post smoking intervention
93)	Lindstrom 2008	Pre-post smoking intervention
94)	Liu 2018	Intervention administered pre- and postoperatively; nutrition
95)	Lyon 2016	Not an RCT
96)	Malek 2018	Did not report any outcomes of interest
97)	Mangell 2012	Intervention administered pre- and postoperatively; probiotic nutrition
98)	Marques 2018	Not an RCT
99)	Martorella 2011	Intervention administered pre- and post operatively; web-based intervention-education
100)	Martorella 2012	Intervention administered pre- and postoperatively; web-based intervention-education
101)	Matheus 2012	Not prehabilitation post intervention
102)	Matassi 2014	Intervention not aimed at improving perioperative outcomes
103)	McNaught 2002	Intervention administered pre- and postoperatively; probiotic nutrition
104)	McRee 2003	Intervention administered in hospital
105)	Minschaert 1982	Intervention administered pre- and postoperatively; incentive spirometry
106)	Mirea 2015	Not an RCT
107)	Milios 2018	Intervention administered pre- and postoperatively or Intervention not aimed at assessing perioperative outcomes
108)	Mitchell 2005	Intervention administered pre- and postoperatively; exercise
109)	Moller 2002	Pre-post smoking intervention
110)	Moradian 2019	Intervention administered in hospital
111)	Mudge 2018	Additional feed in control group
112)	Nardi 2019	Intervention administered pre- and postoperatively; mulitmodal
113)	Neilipovitz 2012	Intervention administered pre- and postoperatively; statin therapy
114)	Nielson 2008	Intervention administered pre- and postoperatively; multimodal
115)	Nielson 2010	Intervention administered pre- and postoperatively; multimodal
116)	Nomori 1994	Not an RCT
117)	Okamoto 2009	comparing immunonutrition ONS to standard ONS.
118)	Onerup 2017	Intervention administered pre- and postoperatively; exercise
119)	Owens 2017	Patients did not undergo surgery
120)	Padmanabhan 2019	Control was oral iron – not usual care
121)	Paleiron 2018	Interventon not prehabilitation

122)	Palma-Milla 2018	Although considered usual care in head and neck patients the control group received ONS. NICE guidelines recommend ONS for those who are malnourished but not all patients are malnourished in this paper.
123)	Parker 2018	Patients did not undergo surgery
124)	Patti 2006	Intervention administered pre- and postoperatively; statin therapy
125)	Pehlivan 2018	Patients did not undergo surgery
126)	Pelligrini 2018	Intervention not aimed at improving perioperative outcomes
127)	Pereira 2016	Intervention administered 15 mins before surgery
128)	Petersen 2006	Interventon not prehabilitation
129)	Rajan 2017	Intervention not aimed at improving perioperative outcomes
130)	Reynolds 2015	Intervention not aimed at improving perioperative outcomes
131)	Rezzan 2017	Interventon not prehabilitation
132)	Rodrigues 2018	Not an RCT
133)	Rolving 2014	Intervention not aimed at improving perioperative outcomes
134)	Rolving 2015	Intervention not aimed at improving perioperative outcomes
135)	Roukema 1988	Intervention administered pre- and postoperatively; respiratory rehabilitation
136)	Ruiz-Tovar 2016	Comparator not usual care
137)	Ruiz-Tovar 2018	Comparator not usual care
138)	Ruiz-Tovar 2019	Comparator not usual care
139)	Ryu 2018	Intervention administered in hospital
140)	St Marie 2018	Intervention not aimed at improving perioperative outcomes
141)	Sadahiro 2014	specific to colon resection - not general prehab
142)	Saravanan 2010	Intervention administered pre- and postoperatively; fish oil nutrition
143)	Savluk 2017	Intervention not prehabilitation
144)	Savci 2011	Intervention administered pre- and postoperatively; IMT
145)	Scantling-Birch 2019	Not major surgery
146)	Senguin 2016	Intervention administered pre- and postoperatively;
147)	Setlik 2019	Intervention not aimed at improving perioperative outcomes
148)	Shan 2011	Intervention administered pre- and postoperatively; nutrition
149)	Shao 2019	Day before surgery
150)	Shukry 2016	Intervention not aimed at improving perioperative outcomes
151)	Siddhartha 2018	Patients did not undergo surgery
152)	Sifuentes 2018	Not major surgery (hernia)

Perry R, *et al. BMJ Open* 2022; 11:e050806. doi: 10.1136/bmjopen-2021-050806

153)	Siggirsdottir 2005	Intervention administered pre- and postoperatively; education
154)	Sigurdsson 2008	Intervention administered pre- and postoperatively; education
155)	Skoffer 2016	Intervention not aimed at improving perioperative outcomes
156)	Skrobot 2019	No outcomes of interest
157)	Sommer 2016	Cannot separate groups for analysis
158)	Sorensen 2003	did not undergo surgery of any kind (they had cuts made to skin to test wound healing)
159)	Sorensen 2007	Pre-post smoking intervention
160)	Sorensen 2018	Cannot tell when the outcomes were measured
161)	Steurer 2018	Summary on Boden et al., 2018
162)	Student 2016	Intervention not aimed at assessing perioperative outcomes
163)	Suzuki 2010	Intervention administered pre- and postoperatively; nutrition
164)	Syed 2018	Intervention not prehabilitation
165)	Tepaske 2007	Standard formula given but not usual care
166)	Tjoumakaris 2017	Intervention not prehabilitation
167)	Thomsen 2010	Pre-post smoking intervention;
168)	Turky 2017	Intervention administered pre- and postoperatively; IMT
169)	Tully 2008	Not an RCT
170)	Turnock 2013	Pre and post intervention. While these patients receive feed PO, the intervention patient received intervention feed, not standard feed.
171)	van Rooijen 2019	Not an RCT
172)	Vareai 2014	Intervention not aimed at assessing perioperative outcomes
173)	Vasheghani 2017	Intervention administered pre- and postoperatively; fish oil nutrition
174)	Vranceanu 2016	Patients did not undergo surgery
175)	Waite 2017	Not an RCT
176)	Wall 2017	Intervention not aimed at assessing perioperative outcomes
177)	Wantanakorn 2018	Patients were children
178)	Wijgman 1994	Aim was to optimise the outcome of their specific operation
179)	Wongkietkachorn 2018	Patients did not undergo major surgery therefore no perioperative outcomes reported
180)	Xu 2019	Not a Prehabilitation intervention
181)	Zarei 2018	Patients did not undergo major surgery
182)	Zhang 2013	Intervention administered pre- and postoperatively
	**CANNOT ACCESS**	

183)	Sivaraman 2010	
184)	Walther 2010	
185)	Heynen 2012	
186)	Mirmooji 2002	
187)	McCarter 1998	
188)	Richardson 2014	

Perry R, *et al. BMJ Open* 2022; 11:e050806. doi: 10.1136/bmjopen-2021-050806

Table 34. Table of studies that fit inclusion criteria but have no usable data reported

**Culligan et al 2017**	Ongoing trial: Does a formal interactive patient education program positively impact patient outcomes and satisfaction after thoracic surgery

References for excluded studies

Ahn, S. H., Park, Y. S., Shin, D. J., Park, D. J. & Kim, H. H. 2015. Multimodal eras (early recovery after surgery) program in combination with totally laparoscopic distal gastrectomy is the optimal perioperative care in patients with gastric cancer: a prospective randomized clinical trial. *Surgical endoscopy and other interventional techniques,* 29**,** S352-.

Aksu, N. T., Erdogan, A. & Ozgur, N. 2018. Effects of progressive muscle relaxation training on sleep and quality of life in patients with pulmonary resection. *Sleep Breath,* 22**,** 695-702.

Aljabari, A. 2018. Oral or intravenous iron intake with erythropoietin in patients scheduled for total hip or knee arthroplasty. A prospective randomized study: Cross iron study. *Regional Anesthesia and Pain Medicine,* 43 (7 Supplement 1)**,** e194.

Almansob, M. A., Xu, B., Zhou, L., Hu, X. X., Chen, W., Chang, F. J., . . . Ou, J. S. 2012. Simvastatin reduces myocardial injury undergoing noncoronary artery cardiac surgery: a randomized controlled trial. *Arteriosclerosis, Thrombosis & Vascular Biology,* 32**,** 2304-13.

Amaravati, R. S. & Sekaran, P. 2013. Does preoperative exercise influence the outcome of ACL reconstruction? *Arthroscopy - Journal of Arthroscopic and Related Surgery,* 1)**,** e182-e183.

Anbar, R., Beloosesky, Y., Madar, Z., Theilla, M., Koren-Hakim, T., Weiss, A. & Et Al. 2012. Tight calorie control (TICACOS) in geriatric hip fracture patients. *Clinical Nutrition, Supplement,* 7**,** 18.

Arango-Gutierrez, A. S., Buitrago-Cifuentes, L. J., Medina-Hinestroza, A. M., Molina-Paniagua, S. A., Moreno, E., Rivera-Diaz, J. S., . . . Gonzalez-Obregon, M. P. 2019. Sonotherapy in the reduction of anxiety and postoperative pain in patients with regional anesthesia as a sole technique: Randomized, controlled clinical trial. [Spanish]. *Cirugia y Cirujanos (English Edition),* 87**,** 545-553.

Ardakani, E. F., Nouri-Majalan, N., Forouzannia, S. & Moshtaghian, H. 2010. Effects of allopurinol and vitamin E on renal function in patients with CABG [abstract]. *Internal Medicine Journal,* 40**,** 95.

Arvidsson, L., Hallbook, T. & Lindblad, B. 1982. Is physiotherapeutic respiratory care valuable for prophylactic purpose after an operation?. [Swedish]. *Lakartidningen,* 79**,** 1480-1481.

Ashida, R., Okamura, Y., Wakabayashi-Nakao, K., Mizuno, T., Aoki, S. & Uesaka, K. 2019. The Impact of Preoperative Enteral Nutrition Enriched with Eicosapentaenoic Acid on Postoperative Hypercytokinemia after Pancreatoduodenectomy: The Results of a Double-Blinded Randomized Controlled Trial. *Dig Surg,* 36**,** 348-356.

Baillot, A., Mampuya, W. M., Dionne, I. J., Comeau, E., Méziat-Burdin, A. & Langlois, M. F. 2016. Impacts of Supervised Exercise Training in Addition to Interdisciplinary Lifestyle Management in Subjects Awaiting Bariatric Surgery: a Randomized Controlled Study. *Obes Surg,* 26**,** 2602-2610.

Bal-Bochenska, M., Kadziolka, W. & Kadziolka, J. 2009. Assessment of the effects of preparatory in-home, and hospital physiotherapy, on patients before surgery of lung parenchyma. *Kardiochirurgia I Torakochirurgia Polska,* 6**,** 191-197.

Banerjee, S., Manley, K., Shaw, B., Kumar, V., Ho, E. T. S., Rochester, M., . . . Saxton, J. 2015. 'Prehabilitation' of patients undergoing radical cystectomy to assist recovery: results of a feasibility study. *European urology, supplements,* 14**,** e444-.

Baptiste, C., Buckley De Meritens, A., Burke, W. M., Hou, J. Y., Wright, J. D. & Tergas, A. I. 2017. A randomized controlled trial of a preoperative patient education program to improve satisfaction and reduce resource utilization. *Gynecologic Oncology,* 145**,** 155-156.

Basen-Engquist, K., Perkins, H. Y., Carmack Taylor, C. L., Hughes, D. C., Jovanovic, J. L., Arun, B. K. & Murray, J. L. 2009. Test of weight gain prevention intervention in stage II and III breast cancer patients receiving neoadjuvant chemotherapy. *Journal of Clinical Oncology,* 1)**,** e20523.

Bitterli, R., Sieben, J. M., Hartmann, M. & De Bruin, E. D. 2009. Pre-Operative, Sensory-Motor Training for Patients undergoing Total Hip Replacement: A Randomised Controlled Trial. *Physikalische Medizin Rehabilitationsmedizin Kurortmedizin,* 19**,** 193-201.

Broadbent, E., Kahokehr, A., Booth, R. J., Thomas, J., Windsor, J. A., Buchanan, C. M., . . . Hill, A. G. 2012. A brief relaxation intervention reduces stress and improves surgical wound healing response: a randomised trial. *Brain, Behavior, & Immunity,* 26**,** 212-7.

Brown, K., Loprinzi, P. D., Brosky, J. A. & Topp, R. 2014. Prehabilitation influences exercise-related psychological constructs such as self-efficacy and outcome expectations to exercise. *Journal of Strength & Conditioning Research,* 28**,** 201-9.

Burnand, K. M., Lahiri, R. P., Burr, N., Jansen Van Rensburg, L. & Lewis, M. P. 2016. A randomised, single blinded trial, assessing the effect of a two week preoperative very low calorie diet on laparoscopic cholecystectomy in obese patients. *HPB,* 18**,** 456-61.

Cao, S. L., Ren, Y., Li, Z., Lin, J., Weng, X. S. & Feng, B. 2020. Clinical effectiveness of 3 days preoperative treatment with recombinant human erythropoietin in total knee arthroplasty surgery: A clinical trial. *Qjm,* 113**,** 245-252.

Carli, F., Bousquet-Dion, G., Awasthi, R., Elsherbini, N., Liberman, S., Boutros, M., . . . Fiore, J. F. 2020. Effect of Multimodal Prehabilitation vs Postoperative Rehabilitation on 30-Day Postoperative Complications for Frail Patients Undergoing Resection of Colorectal Cancer: A Randomized Clinical Trial. *JAMA Surgery,* 155**,** 233-242.

Carli, F., Charlebois, P., Stein, B., Feldman, L., Zavorsky, G., Kim, D. J., . . . Mayo, N. E. 2010. Randomized clinical trial of prehabilitation in colorectal surgery. *British Journal of Surgery,* 97**,** 1187-97.

Castillo, R. & Haas, A. 1985. Chest physical therapy: comparative efficacy of preoperative and postoperative in the elderly. *Arch Phys Med Rehabil,* 66**,** 376-9.

Cheung, L. H., Callaghan, P. & Chang, A. M. 2003. A controlled trial of psycho-educational interventions in preparing Chinese women for elective hysterectomy. *International Journal of Nursing Studies,* 40**,** 207-16.

Chevillon, C., Hellyar, M., Madani, C., Kerr, K. & Son Chae, K. 2015. PREOPERATIVE EDUCATION ON POSTOPERATIVE DELIRIUM, ANXIETY, AND KNOWLEDGE IN PULMONARY THROMBOENDARTERECTOMY PATIENTS. *American Journal of Critical Care,* 24**,** 164-171.

Cho, H., Tsuburaya, A., Sakamoto, J., Morita, S., Oba, K., Yoshikawa, T. & Miyajima, N. 2008. A Randomized Phase II Trial of Preoperative Exercise to Reduce Operative Risk in Gastric Cancer Patients with Metabolic Syndrome: Adjuvant Exercise for General Elective Surgery (AEGES) Study Group. *Japanese Journal of Clinical Oncology,* 38**,** 71-73.

Christensen, J. F., Simonsen, C., Banck-Petersen, A., Thorsen-Streit, S., Herrstedt, A., Djurhuus, S. S., . . . De Heer, P. 2019. Safety and feasibility of preoperative exercise training during neoadjuvant treatment before surgery for adenocarcinoma of the gastro-oesophageal junction. *Bjs Open,* 3**,** 74-84.

Coats, V., Maltais, F., Simard, S., Frechette, E., Tremblay, L., Ribeiro, F. & Saey, D. 2013. Feasibility and effectiveness of a home-based exercise training program before lung resection surgery. *Canadian Respiratory Journal,* 20**,** E10-E16.

Cordeiro, A. L., Barbosa, H. M., Landerson, L., Lima, C., Araujo, J., Souza, A., . . . Petto, J. 2019. Inspiratory muscle training based on the anaerobiosis threshold on the functional capacity of patients submitted to coronary arterial bypass grafting: Randomized and controlled clinical

trial. *European Respiratory Journal. Conference: 29th International Congress of the European Respiratory Society, ERS. Spain.,* 54.

Culligan, M., Black, L., Norton, C., Wimbush, S., Wells, C., Jorshari, F., . . . Carr, S. 2017. Winners study: Does a formal interactive patient education program positively impact patient outcomes and satisfaction after thoracic surgery. *Journal of Thoracic Oncology,* 12 (1 Supplement 1)**,** S1096-S1097.

Dao, T. K., Youssef, N. A., Armsworth, M., Wear, E., Papathopoulos, K. N. & Gopaldas, R. 2011. Randomized controlled trial of brief cognitive behavioral intervention for depression and anxiety symptoms preoperatively in patients undergoing coronary artery bypass graft surgery. *Journal of Thoracic & Cardiovascular Surgery,* 142**,** e109-15.

Darnall, B. D., Ziadni, M. S., Krishnamurthy, P., Flood, P., Heathcote, L. C., Mackey, I. G., . . . Wheeler, A. 2019. "My Surgical Success": Effect of a Digital Behavioral Pain Medicine Intervention on Time to Opioid Cessation After Breast Cancer Surgery—A Pilot Randomized Controlled Clinical Trial. *Pain Medicine,* 20**,** 2228-2237.

Das Nair, R., Mhizha-Murira, J. R., Anderson, P., Carpenter, H., Clarke, S., Groves, S., . . . Lincoln, N. B. 2018. Home-based pre-surgical psychological intervention for knee osteoarthritis (HAPPiKNEES): a feasibility randomized controlled trial. *Clin Rehabil,* 32**,** 777-789.

De Heer, P., Christensen, J., Simonsen, C., Banck-Petersen, A., Kristensen, T., Garbyal, R., . . . Kofoed, S. 2018. Exercise training during neo-adjuvant therapy in patients undergoing surgery for cancer of the gastro-esophageal junction. *Diseases of the Esophagus,* 31 (Supplement 1)**,** 127-128.

De Wit, P., Duivenvoorden, H. J. & Van Dixhoorn, J. J. 1996. [More psychological preparation in heart surgery for certain patients is beneficial]. *Nederlands Tijdschrift voor Geneeskunde,* 140**,** 1720-3.

Dettling, D. S., Van Der Schaaf, M., Blom, R. L., Nollet, F., Busch, O. R. & Van Berge Henegouwen, M. I. 2013. Feasibility and effectiveness of pre-operative inspiratory muscle training in patients undergoing oesophagectomy: a pilot study. *Physiotherapy Research International,* 18**,** 16-26.

Dreyer, H. C., Owen, E. C., Strycker, L. A., Smolkowski, K., Muyskens, J. B., Kirkpatrick, T. K., . . . Jewett, B. A. 2018. Essential Amino Acid Supplementation Mitigates Muscle Atrophy After Total Knee Arthroplasty: A Randomized, Double-Blind, Placebo-Controlled Trial. *JB JS Open Access,* 3**,** e0006.

Edwards, T. J., Noble, E. J., Durran, A., Mellor, N. & Hosie, K. B. 2009. Randomized clinical trial of preoperative intravenous iron sucrose to reduce blood transfusion in anaemic patients after colorectal cancer surgery. *British Journal of Surgery,* 96**,** 1122-1128.

Erdem, N. Z., Ozelgun, D., Taskin, H. E., Avsar, F. M. & Taskin, M. 2018. The effects of protein-riched diet, performed before bariatric surgery, on losing weight, clinical results and liver volume reduction. *Obesity Surgery,* 28 (1 Supplement 1)**,** S61.

Farquharson, A. L., Metcalf, R. G., Sanders, P., Stuklis, R., Edwards, J. R., Gibson, R. A., . . . Young, G. D. 2011. Effect of dietary fish oil on atrial fibrillation after cardiac surgery. *American Journal of Cardiology,* 108**,** 851-6.

Farquharson, A. L., Metcalf, R. G., Stuklis, R., Edwards, J. R. M., Cleland, L. G., James, M. J., . . . Young, G. D. 2011. Dietary fish oils delay the development of atrial fibrillation and reduce intensive care unit stay after cardiac surgery. *Heart Rhythm,* 1)**,** S425.

Feltrim, M. I. Z., Jatene, F. B. & Bernardo, W. M. 2007. In high risk patients, who have undergone myocardial revascularization, does the preoperative respiratory physiotherapy prevent pulmonary complications? *Revista da Associacao Medica Brasileira,* 53**,** 8-9.

Finco, C., Magnanini, P., Sarzo, G., Vecchiato, M., Luongo, B., Savastano, S., . . . Merigliano, S. 2007. Prospective randomized study on perioperative enteral immunonutrition in laparoscopic colorectal surgery. *Surgical Endoscopy,* 21**,** 1175-9.

Foley, N. M., O'connell, E. P., Lehane, E. A., Livingstone, V., Maher, B., Kaimkhani, S., . . . Corrigan, M. A. 2016. PATI: Patient accessed tailored information: A pilot study to evaluate the effect on

preoperative breast cancer patients of information delivered via a mobile application. *Breast,* 30**,** 54-58.

Furukawa, K. 2014. Immunonutrition for patients undergoing pancreatoduodenectomy. *Hpb,* 16**,** 31.

Furukawa, K., Aida, T., Suzuki, D., Shimizu, H., Yoshidome, H., Ohtsuka, M., . . . Miyazaki, M. 2012. Preoperative immunonutrition modulates prostaglandin E2 production and T cell differentiation in patients undergoing pancreaticoduodenectomy. *Clinical Nutrition, Supplement,* 7 (1)**,** 151.

Gianotti, L., Biffi, R., Sandini, M., Marrelli, D., Vignali, A., Caccialanza, R., . . . Bernasconi, D. P. 2017. Preoperative Oral Carbohydrate Load Versus Placebo in Major Elective Abdominal Surgery (PROCY): A Randomized, Placebo-controlled, Multicenter, Phase III Trial. *Annals of Surgery,* 02**,** 02.

Giger, U., Buchler, M., Farhadi, J., Berger, D., Husler, J., Schneider, H., . . . Krahenbuhl, L. 2007. Preoperative immunonutrition suppresses perioperative inflammatory response in patients with major abdominal surgery-a randomized controlled pilot study. *Annals of Surgical Oncology,* 14**,** 2798-806.

Giger-Pabst, U., Lange, J., Maurer, C., Bucher, C., Schreiber, V., Schlumpf, R., . . . Krahenbuhl, L. 2013. Short-term preoperative supplementation of an immunoenriched diet does not improve clinical outcome in well-nourished patients undergoing abdominal cancer surgery. *Nutrition,* 29**,** 724-9.

Giles, A. E. & Srinathan, S. K. 2019. Prehabilitation prior to lung cancer surgery: A small step forward. *Journal of Thoracic Disease,* 11**,** 5664-5665.

Gillis, C., Li, C., Lee, L., Awasthi, R., Augustin, B., Gamsa, A., . . . Carli, F. 2014. Prehabilitation versus rehabilitation: a randomized control trial in patients undergoing colorectal resection for cancer. *Anesthesiology,* 121**,** 937-47.

Gils Contreras, A., Bonada Sanjaume, A., Montero Jaime, M., Rabassa Soler, A., Sabench Pereferrer, F., Molina Lopez, A., . . . Salas-Salvado, J. 2018. Effects of Two Preoperatory Weight Loss Diets on Hepatic Volume, Metabolic Parameters, and Surgical Complications in Morbid Obese Bariatric Surgery Candidates: a Randomized Clinical Trial. *Obesity Surgery,* 14**,** 14.

Gocen, Z., Sen, A., Unver, B., Karatosun, V. & Gunal, I. 2004. The effect of preoperative physiotherapy and education on the outcome of total hip replacement: a prospective randomized controlled trial. *Clinical Rehabilitation,* 18**,** 353-8.

Grant, L. F., Cooper, D. J. & Conroy, J. L. 2017. The HAPI 'Hip Arthroscopy Pre-habilitation Intervention' study: does pre-habilitation affect outcomes in patients undergoing hip arthroscopy for femoro-acetabular impingement? *Journal of Hip Preservation Surgery,* 4**,** 85-92.

Grawe, J. S., Mirow, L., Bouchard, R., Lindig, M. & Huppe, M. 2010. [Impact of preoperative patient education on postoperative pain in consideration of the individual coping style]. *Der Schmerz,* 24**,** 575-86.

Heidarsdottir, R., Arnar, D. O., Skuladottir, G. V., Torfason, B., Edvardsson, V., Gottskalksson, G., . . . Indridason, O. S. 2010. Does treatment with n-3 polyunsaturated fatty acids prevent atrial fibrillation after open heart surgery? *Europace,* 12**,** 356-363.

Hermann, A., Holsgaard-Larsen, A., Mejdahl, S., Zerahn, B. & Overgaard, S. 2013. Preoperative resistance training increases muscle function in patients diagnosed with hip osteoarthritis scheduled for total hip arthroplasty A- a randomized explorative trial. *Osteoarthritis and Cartilage,* 21**,** S149-S150.

Hermann, A., Holsgaard-Larsen, A., Zerahn, B., Mejdahl, S. & Overgaard, S. 2014. Preoperative effects of progressive explosive-type resistance training in patients with osteoarthritis scheduled for total hip arthroplasty-a prospective randomized clinical trial. *Osteoarthritis and Cartilage,* 22**,** S454-S455.

Heynen, H., De Jonge, C., Kerkkamp, H., Willms, J. & Sosef, M. 2012. Preconditioning in patients undergoing esophagectomy: A randomized controlled pilot study. *Diseases of the Esophagus,* 25**,** 84A.

Hogan, S., Solomon, M., Rangan, A., Ferrie, S. & Carey, S. 2019. The Impact of Preoperative Immunonutrition and Standard Polymeric Supplements on Patient Outcomes After Pelvic Exenteration Surgery, Taking Compliance Into Consideration: A Randomized Controlled Trial. *Journal of Parenteral and Enteral Nutrition.*

Holsgaard-Larsen, A., Herman, A., Zerahn, B., Mejdahl, S. & Overgaard, S. 2018. POSTOPERATIVE EFFECTS OF PROGRESSIVE RESISTANCE TRAINING PRIOR TO TOTAL HIP ARTHROPLASTY - ONE YEAR OUTCOME OF A RANDOMIZED CONTROLLED TRIAL. *Osteoarthritis and Cartilage,* 26**,** S330-S331.

Horchner, R. & Tuinebreijer, W. 1999. Preoperative preparatory program has no effect on morbidly obese patients undergoing a Lap-Band operation. *Obesity Surgery,* 9**,** 250-257.

Ishikawa, Y., Yoshida, H., Weiner, J. & Tajiri, T. 2009. Prospective randomized controlled study of short-term perioperative enteral nutrition support with branched chain amino acids in liver surgery. *Gastroenterology,* Conference: Digestive Disease Week, DDW 2009 Chicago, IL United States. Conference Start: 20090530 Conference End: 20090604. Conference Publication:**,** A794-A795.

Iskender, M. D., Bektas, O. & Eren, H. 2020. Effect of preoperative in-bed exercises and mobilization training on postoperative anxiety and mobilization level. *Japan Journal of Nursing Science: JJNS***,** e12339.

Jahic, D., Omerovic, D., Tanovic, A. T., Dzankovic, F. & Campara, M. T. 2018. The Effect of Prehabilitation on Postoperative Outcome in Patients Following Primary Total Knee Arthroplasty. *Medical archives (Sarajevo, Bosnia and Herzegovina),* 72**,** 439-443.

Jensen, B. T., Kiesbye, B., Soendergaard, I., Jensen, J. B. & Kristensen, S. A. 2017. Efficacy of preoperative uro-stoma education on self-efficacy after Radical Cystectomy; secondary outcome of a prospective randomized controlled trial. *European Journal of Oncology Nursing,* 28**,** 41-46.

Jensen, B. T., Laustsen, S., Jensen, J. B., Borre, M. & Petersen, A. K. 2016. Exercise-based pre-habilitation is feasible and effective in radical cystectomy pathways-secondary results from a randomized controlled trial. *Supportive Care in Cancer,* 24**,** 3325-31.

Jensen, B. T., Petersen, A. K., Jensen, J. B., Laustsen, S. & Borre, M. 2015. Efficacy of a multiprofessional rehabilitation programme in radical cystectomy pathways: a prospective randomized controlled trial. *Scandinavian Journal of Urology,* 49**,** 133-41.

Kale, P. M., Mohite, V. R., Mohite, R. V., Chendake, M. B. & Gholap, M. C. 2017. The effectiveness of pre-operative deep breathing exercise on post-operative patients of abdominal surgery. *Asian journal of pharmaceutical and clinical research,* 10**,** 157-160.

Kalogianni, A., Almpani, P., Vastardis, L., Baltopoulos, G., Charitos, C. & Brokalaki, H. 2016. Can nurse-led preoperative education reduce anxiety and postoperative complications of patients undergoing cardiac surgery? *European journal of cardiovascular nursing,* 15**,** 447-458.

Kara, H. & Yasim, A. 2020. Effects of high-dose vitamin D supplementation on the occurrence of post-operative atrial fibrillation after coronary artery bypass grafting: randomized controlled trial. *General Thoracic and Cardiovascular Surgery,* 68**,** 477-484.

Keeler 2019. The impact of pre-operative intravenous iron on quality of life after colorectal cancer surgery: outcomes from the intravenous iron in colorectal cancer-associated anaemia (IVICA) trial (vol 74, pg 714, 2019). *Anaesthesia,* 74**,** 1191-1191.

Keen, C., Skilbeck, J., Ross, H., Smith, L., Collins, K., Dixey, J., . . . Mawson, S. 2018. Is it feasible to conduct a randomised controlled trial of pretransplant exercise (prehabilitation) for patients with multiple myeloma awaiting autologous haematopoietic stem cell transplantation? Protocol for the PREeMPT study. 8**,** e021333.

Kim, I. & Lee, H. 2014. Effects of a Progressive Walking Program on Physical Activity, Exercise Tolerance, Recovery, and Post-Operative Complications in Patients with a Lung Resection. *Journal of Korean Academy of Nursing,* 44**,** 381-390.

Kinoshita, K., Beppu, T., Sato, N., Akahoshi, S., Yuki, H. & Yoshida, Y. 2019. Preoperative 1-week diet can markedly decrease blood loss during hepatectomy. *Translational Gastroenterology and Hepatology,* 4 (March) (no pagination).

Kitamura, H., Yamada, S., Adachi, T., Shibata, K., Tamaki, M., Okawa, Y. & Usui, A. 2019. Effect of Perioperative Neuromuscular Electrical Stimulation in Patients Undergoing Cardiovascular Surgery: A Pilot Randomized Controlled Trial. *Seminars in Thoracic and Cardiovascular Surgery,* 31**,** 361-367.

Klaiber, U., Stephan-Paulsen, L. M., Bruckner, T., Müller, G., Auer, S., Farrenkopf, I., . . . Knebel, P. 2018. Impact of preoperative patient education on the prevention of postoperative complications after major visceral surgery: the cluster randomized controlled PEDUCAT trial. *Trials,* 19**,** 288.

Kumar, A. S., Alaparthi, G. K., Augustine, A. J., Pazhyaottayil, Z. C., Ramakrishna, A. & Krishnakumar, S. K. 2016. Comparison of Flow and Volume Incentive Spirometry on Pulmonary Function and Exercise Tolerance in Open Abdominal Surgery: A Randomized Clinical Trial. *Journal of Clinical and Diagnostic Research JCDR,* 10**,** KC01-6.

Laferton, J. A., Auer, C. J., Shedden-Mora, M. C., Moosdorf, R. & Rief, W. 2016. Optimizing preoperative expectations in cardiac surgery patients is moderated by level of disability: the successful development of a brief psychological intervention. *Psychology Health & Medicine,* 21**,** 272-85.

Lahiri, R., Burr, N., Burnand, K., Bennett, J. & Lewis, M. 2014. A randomised, single-blinded trial assessing the effect of a two week preoperative very low calorie diet on laparoscopic cholecystectomy in obese patients. *British Journal of Surgery,* 101**,** 2-2.

Larson, M. R., Duberstein, P. R., Talbot, N. L., Caldwell, C. & Moynihan, J. A. 2000. A presurgical psychosocial intervention for breast cancer patients. psychological distress and the immune response. *J Psychosom Res,* 48**,** 187-94.

Lee, C. H., Liu, J. T., Lin, S. C., Hsu, T. Y., Lin, C. Y. & Lin, L. Y. 2018. Effects of Educational Intervention on State Anxiety and Pain in People Undergoing Spinal Surgery: A Randomized Controlled Trial. *Pain Manag Nurs,* 19**,** 163-171.

Lee, J. S., Song, Y., Kim, J. Y., Park, J. S. & Yoon, D. S. 2018. Effects of Preoperative Oral Carbohydrates on Quality of Recovery in Laparoscopic Cholecystectomy: A Randomized, Double Blind, Placebo-Controlled Trial. *World Journal of Surgery,* 42**,** 3150-3157.

Lee, S. M., Tenney, R., Wallace, A. W. & Arjomandi, M. 2018. E-cigarettes versus nicotine patches for perioperative smoking cessation: a pilot randomized trial. *PeerJ,* 6**,** e5609.

Lenz, E. R. & Perkins, S. 2000. Coronary artery bypass graft surgery patients and their family member caregivers: outcomes of a family-focused staged psychoeducational intervention. *Applied Nursing Research,* 13**,** 142-50.

Leong, J. Y., Van Der Merwe, J., Pepe, S., Bailey, M., Perkins, A., Lymbury, R., . . . Rosenfeldt, F. 2010. Perioperative metabolic therapy improves redox status and outcomes in cardiac surgery patients: a randomised trial. *Heart, Lung & Circulation,* 19**,** 584-91.

Lewis, S., Pugsley, M., Schneider, C., Rakita, S. S. & Moudgill, L. J. 2018. The Effect of Immunonutrition on Veterans Undergoing Major Surgery for Gastrointestinal Cancer. *Federal Practitioner,* 35**,** S49-S56.

Liang, M. K., Bernardi, K., Holihan, J. L., Cherla, D. V., Escamilla, R., Lew, D. F., . . . Kao, L. S. 2018. Modifying Risks in Ventral Hernia Patients With Prehabilitation: A Randomized Controlled Trial. *Ann Surg,* 268**,** 674-680.

Ligibel, J. A., Irwin, M., Dillon, D., Barry, W., Giobbie-Hurder, A., Frank, E., . . . Jeselsohn, R. 2017. Impact of pre-operative exercise on breast cancer gene expression. *Cancer Research.*

Conference: 39th Annual CTRC AACR San Antonio Breast Cancer Symposium. United States, 77.

Liljensoe, A., Laursen, J. O., Bliddal, H., Soballe, K. & Mechlenburg, I. 2019. Weight Loss Intervention Before Total Knee Replacement: A 12-Month Randomized Controlled Trial. *Scandinavian journal of surgery : SJS : official organ for the Finnish Surgical Society and the Scandinavian Surgical Society*, 1457496919883812.

Lim, J., Miles, L. & Litton, E. 2018. Intravenous Iron Therapy in Patients Undergoing Cardiovascular Surgery: A Narrative Review. *Journal of Cardiothoracic and Vascular Anesthesia,* 32**,** 1439-1451.

Lindstrom, D., Sadr Azodi, O., Wladis, A., Tonnesen, H., Linder, S., Nasell, H., . . . Adami, J. 2008. Effects of a perioperative smoking cessation intervention on postoperative complications: a randomized trial. *Annals of Surgery,* 248**,** 739-45.

Lindström, D., Wladis, A. & Pekkari, K. 2010. The thioredoxin and glutaredoxin systems in smoking cessation and the possible relation to postoperative wound complications. *Wounds: A Compendium of Clinical Research & Practice,* 22**,** 88-93.

Lyon, T., Turner, R., Mcbride, D., Gingrich, J., Davies, B., Jacobs, B. & Tarin, T. 2016. A pilot study of high-arginine nutritional supplementation prior to radical cystectomy. *Canadian Urological Association Journal,* 10 (9-10 Supplement 4)**,** S158-S159.

Malek, N. M., Zakerimoghadam, M., Esmaeili, M. & Kazemnejad, A. 2018. Effects of Nurse-Led Intervention on Patients' Anxiety and Sleep Before Coronary Artery Bypass Grafting. *Critical care nursing quarterly,* 41**,** 161-169.

Mangell, P., Thorlacius, H., Syk, I., Ahrne, S., Molin, G., Olsson, C. & Jeppsson, B. 2012. Lactobacillus plantarum 299v does not reduce enteric bacteria or bacterial translocation in patients undergoing colon resection. *Digestive Diseases & Sciences,* 57**,** 1915-24.

Marie, B. S., Hadlandsmyth, K., Embree, J., Tripp, T., Allen, J., Dindo, L. & Rakel, B. 2018. Feasibility of 1-day ACT intervention for at-risk veterans undergoing orthopedic surgery: barriers and facilitators. *The Journal of Pain,* 19**,** S38-S39.

Marques, C. J. 2018. CORR Insights: Preoperative Physical Therapy Education Reduces Time to Meet Functional Milestones after Total Joint Arthroplasty. *Clinical Orthopaedics and Related Research,* 476**,** 49-51.

Martorella, G. 2011. *Developpement et evaluation d'une intervention infirmiere virtuelle sur mesure visant a faciliter l'autogestion de la douleur apres une chirurgie cardiaque.* Ph.D., Universite de Montreal (Canada).

Martorella, G. 2011. Developpement et evaluation d'une intervention infirmiere virtuelle sur mesure visant a faciliter l'autogestion de la douleur apres une chirurgie cardiaque. *Developpement ET evaluation d'une intervention infirmiere virtuelle sur mesure visant a faciliter l'autogestion de la douleur apres une chirurgie cardiaque***,** 400 p.

Martorella, G., Cote, J., Racine, M. & Choiniere, M. 2012. Web-based nursing intervention for self-management of pain after cardiac surgery: pilot randomized controlled trial. *Journal of Medical Internet Research,* 14**,** e177.

Mat Eil Ismail, M. S., Sharifudin, M. A., Shokri, A. A. & Ab Rahman, S. 2016. Preoperative physiotherapy and short-term functional outcomes of primary total knee arthroplasty. *Singapore Med J,* 57**,** 138-43.

Matassi, F., Duerinckx, J., Vandenneucker, H. & Bellemans, J. 2014. Range of motion after total knee arthroplasty: the effect of a preoperative home exercise program. *Knee Surgery, Sports Traumatology, Arthroscopy,* 22**,** 703-9.

Matheus, G. B., Dragosavac, D., Trevisan, P., Costa, C. E., Lopes, M. M. & Ribeiro, G. C. 2012. Inspiratory muscle training improves tidal volume and vital capacity after CABG surgery. *Revista Brasileira de Cirurgia Cardiovascular: Orgao Oficial da Sociedade Brasileira de Cirurgia Cardiovascular,* 27**,** 362-9.

Mccarter, M. D., Gentilini, O. D., Gomez, M. E. & Daly, J. M. 1998. Preoperative oral supplement with immunonutrients in cancer patients. *Jpen: Journal of Parenteral & Enteral Nutrition,* 22**,** 206-11.

Mcnaught, C. E., Woodcock, N. P., Macfie, J. & Mitchell, C. J. 2002. A prospective randomised study of the probiotic Lactobacillus plantarum 299V on indices of gut barrier function in elective surgical patients. *Gut,* 51**,** 827-31.

Mcree, L. D., Noble, S. & Pasvogel, A. 2003. Using massage and music therapy to improve postoperative outcomes. *AORN Journal,* 78**,** 433-42, 445-7.

Milios, J., Ackland, T. & Green, D. 2018. New protocols for a faster return to continence and quality of life following radical prostatectomy. *BJU international. Conference: 19th asia-pacific prostate cancer conference. Australia,* 122**,** 17.

Minschaert, M., Vincent, J. L., Ros, A. M. & Kahn, R. J. 1982. Influence of incentive spirometry on pulmonary volumes after laparotomy. *Acta Anaesthesiologica Belgica,* 33**,** 203-9.

Mirea, L., Pavelescu, D. & Grintescu, I. 2015. Could preoperative and postoperative optimal nutrition support modulate the inflammatory response and clinical outcome of severe malnourished surgical patients with gastrointestinal neoplasia? *Critical Care,* 19**,** S139.

Mirmooji, S. 2002. Impact of the preoperative administration of an oral nutritional supplement on postoperative infections. *Quebec Pharmacie,* 49**,** 233-4,cp11.

Mitchell, C., Walker, J., Walters, S., Morgan, A. B., Binns, T. & Mathers, N. 2005. Costs and effectiveness of pre- and post-operative home physiotherapy for total knee replacement: randomized controlled trial. *Journal of Evaluation in Clinical Practice,* 11**,** 283-92.

Moller, A. M., Villebro, N., Pedersen, T. & Tonnesen, H. 2002. Effect of preoperative smoking intervention on postoperative complications: a randomised clinical trial. *Lancet,* 359**,** 114-7.

Moradian, S. T., Heydari, A. A. & Mahmoudi, H. 2019. What is the role of preoperative breathing exercises in reducing postoperative atelectasis after cabg? *Reviews on Recent Clinical Trials,* 14**,** 275-279.

Mudge, L. A., Watson, D. I., Smithers, B. M., Isenring, E. A., Smith, L. & Jamieson, G. G. 2018. Multicentre factorial randomized clinical trial of perioperative immunonutrition versus standard nutrition for patients undergoing surgical resection of oesophageal cancer. *Br J Surg,* 105**,** 1262-1272.

Nardi, P., Pellegrino, A., Pisano, C., Vacirca, S. R., Anselmi, D., Saulle, S., . . . Ruvolo, G. 2019. The effect of preoperative respiratory physiotherapy and motor exercise in patients undergoing elective cardiac surgery: short-term results. *Kardiochirurgia I Torakochirurgia Polska,* 16**,** 81-87.

Neilipovitz, D. T., Bryson, G. L. & Taljaard, M. 2012. STAR VaS - Short Term Atorvastatin Regime for Vasculopathic Subjects: a randomized placebo-controlled trial evaluating perioperative atorvastatin therapy in noncardiac surgery. *Canadian Journal of Anesthesia-Journal Canadien D Anesthesie,* 59**,** 527-537.

Nielsen, P. R., Andreasen, J., Asmussen, M. & Tønnesen, H. 2008. Costs and quality of life for prehabilitation and early rehabilitation after surgery of the lumbar spine. *BMC Health Serv Res,* 8**,** 209.

Nielsen, P. R., Jørgensen, L. D., Dahl, B., Pedersen, T. & Tønnesen, H. 2010. Prehabilitation and early rehabilitation after spinal surgery: randomized clinical trial. *Clin Rehabil,* 24**,** 137-48.

Nomori, H., Kobayashi, Fuyuno, G., Morinaga, S. & Yashima, H. 1994. Preoperative respiratory muscle training: assessment in thoracic surgery patients with special reference to postoperative pulmonary complications. *Chest,* 105**,** 1782-8.

Okamoto, Y., Okano, K., Izuishi, K., Usuki, H., Wakabayashi, H. & Suzuki, Y. 2009. Attenuation of the systemic inflammatory response and infectious complications after gastrectomy with preoperative oral arginine and omega-3 fatty acids supplemented immunonutrition. *World Journal of Surgery,* 33**,** 1815-21.

Onerup, A., Angenete, E., Bock, D., Borjesson, M., Fagevik Olsen, M., Gryback Gillheimer, E., . . . Nilsson, H. 2017. The effect of pre- and post-operative physical activity on recovery after colorectal cancer surgery (PHYSSURG-C): study protocol for a randomised controlled trial. *Trials [Electronic Resource],* 18**,** 212.

Padmanabhan, H., Siau, K., Nevill, A. M., Morgan, I., Cotton, J., Ng, A., . . . Luckraz, H. 2019. Intravenous iron does not effectively correct preoperative anaemia in cardiac surgery: a pilot randomized controlled trial. *Interact Cardiovasc Thorac Surg,* 28**,** 447-454.

Paleiron, N., Grassin, F., Lancelin, C., Tromeur, C., Margery, J., Berard, H., . . . Couturaud, F. 2018. Late breaking abstract-pre-operative non invasive ventilation (NIV) does not decrease post-operative complication rate after carcinologic lung surgery : The preOVNI GFPC 12-01 randomized controlled study. *European Respiratory Journal. Conference: European Respiratory Society International Congress, ERS,* 52.

Parker, S., Zipursky, J., Ma, H., Baumblatt, G. L. & Siegel, C. A. 2018. A Web-based Multimedia Program Before Colonoscopy Increased Knowledge and Decreased Anxiety, Sedation Requirement, and Procedure Time. *J Clin Gastroenterol,* 52**,** 519-523.

Patti, G., Chello, M., Candura, D., Pasceri, V., D'ambrosio, A., Covino, E. & Di Sciascio, G. 2006. Randomized trial of atorvastatin for reduction of postoperative atrial fibrillation in patients undergoing cardiac surgery - Results of the ARMYDA-3 (Atorvastatin for reduction of MYocardial dysrhythmia after cardiac surgery) study. *Circulation,* 114**,** 1455-1461.

Pehlivan, E., Mutluay, F., Balcı, A. & Kılıç, L. 2018. The effects of inspiratory muscle training on exercise capacity, dyspnea and respiratory functions in lung transplantation candidates: a randomized controlled trial. *Clin Rehabil,* 32**,** 1328-1339.

Pellegrini, C. A., Chang, R. W., Dunlop, D. D., Conroy, D. E., Lee, J., Van Horn, L., . . . Cameron, K. A. 2018. Comparison of a Patient-Centered Weight Loss Program starting before versus after knee replacement: A pilot study. *Obes Res Clin Pract,* 12**,** 472-478.

Pereira, L., Figueiredo-Braga, M. & Carvalho, I. P. 2016. Preoperative anxiety in ambulatory surgery: The impact of an empathic patient-centered approach on psychological and clinical outcomes. *Patient Education & Counseling,* 99**,** 733-8.

Petersen, M. K., Madsen, C., Andersen, N. T. & Soballe, K. 2006. Efficacy of multimodal optimization of mobilization and nutrition in patients undergoing hip replacement: a randomized clinical trial. *Acta Anaesthesiologica Scandinavica,* 50**,** 712-7.

Rajan, R. 2017. Hepatic volume reduction in obese individuals following omega-3 polyunsaturated fatty acid supplements versus very low calorie dietary restriction. pre-operative management. *Obesity Surgery,* 27 (1 Supplement 1)**,** 829.

Reynolds, S. G., Baima, J., Woo, L., Waugh, D., Sooy, J., Larkin, A., . . . Edmiston, K. 2015. Prehabilitation for shoulder dysfunction in breast cancer: A pilot study. *PM and R,* 1)**,** S179-S180.

Rezzan, G., Marinelli, G., Marcato, F., Melegati, G., Tettamanti, A. & Gatti, R. 2015. Efficacy of action observation pre-operative training in functional recovery after hip and knee prosthesis. *Physiotherapy (United Kingdom),* 101**,** eS1502-eS1503.

Richardson, K., Sanders, G., Hayden, P., Marcora, S. & Hopker, J. 2014. The effect of preoperative exercise on postoperative outcome in abdominal aortic aneurysm (AAA) patients: Pilot study. *Intensive care medicine,* 40**,** S136.

Richardson, K., Sanders, G., Hayden, P., Marcora, S. & Hopker, J. 2014. The effect of preoperative exercise on postoperative outcome in abdominal aortic aneurysm (AAA) patients: Pilot study. *Intensive care medicine,* 40**,** S136.

Rodrigues, M. A., Ferreira, L. M., De Carvalho Calvi, E. N. & Nahas, F. X. 2018. Preoperative Respiratory Physiotherapy in Abdominoplasty Patients. *Aesthetic surgery journal,* 38**,** 291-299.

Rolving, N., Nielsen, C. V., Christensen, F. B., Holm, R., Bunger, C. & Ostergaard, L. 2014. Does a preoperative cognitive-behavioural intervention affect postsurgical pain, mobilisation and length of hospitalisation in lumbar spinal fusion patients? *European Spine Journal,* 23**,** S572.

Rolving, N., Nielsen, C. V., Christensen, F. B., Holm, R., Bunger, C. E. & Oestergaard, L. G. 2015. Does a Preoperative Cognitive-Behavioral Intervention Affect Disability, Pain Behavior, Pain, and Return to Work the First Year After Lumbar Spinal Fusion Surgery? *Spine (03622436),* 40**,** 593-600.

Roukema, J. A., Carol, E. J. & Prins, J. G. 1988. The prevention of pulmonary complications after upper abdominal surgery in patients with noncompromised pulmonary status. *Archives of Surgery,* 123**,** 30-4.

Ruiz-Tovar, J., Blanca, M., Garcia, A., Gonzalez, J., Gutierrez, S., Paniagua, A., . . . Duran, M. 2019. Preoperative administration of Omega-3 fatty acids on postoperative pain and acute-phase reactants in patients undergoing Roux-en-Y gastric bypass: A randomized clinical trial. *Clin Nutr,* 38**,** 1588-1593.

Ruiz-Tovar, J., Blanca, M., Garcia, A., Gonzalez, J., Gutierrez, S., Paniagua, A., . . . Duran, M. 2019. Preoperative administration of Omega-3 fatty acids on postoperative pain and acute-phase reactants in patients undergoing Roux-en-Y gastric bypass: A randomized clinical trial. *Clinical Nutrition,* 38**,** 1588-1593.

Ruiz-Tovar, J., Zubiaga, L., Diez, M., Murcia, A., Boix, E., Muñoz, J. L. & Llavero, C. 2016. Preoperative Regular Diet of 900 kcal/day vs Balanced Energy High-Protein Formula vs Immunonutrition Formula: Effect on Preoperative Weight Loss and Postoperative Pain, Complications and Analytical Acute Phase Reactants After Laparoscopic Sleeve Gastrectomy. *Obes Surg,* 26**,** 1221-7.

Ryu, J. H., Park, J. W., Nahm, F. S., Jeon, Y. T., Oh, A. Y., Lee, H. J., . . . Han, S. H. 2018. The Effect of Gamification through a Virtual Reality on Preoperative Anxiety in Pediatric Patients Undergoing General Anesthesia: A Prospective, Randomized, and Controlled Trial. *J Clin Med,* 7.

Sadahiro, S., Suzuki, T., Tanaka, A., Okada, K., Kamata, H., Ozaki, T. & Koga, Y. 2014. Comparison between oral antibiotics and probiotics as bowel preparation for elective colon cancer surgery to prevent infection: prospective randomized trial. *Surgery,* 155**,** 493-503.

Saravanan, P., Bridgewater, B., West, A. L., O'neill, S. C., Calder, P. C. & Davidson, N. C. 2010. Omega-3 fatty acid supplementation does not reduce risk of atrial fibrillation after coronary artery bypass surgery: A randomized, double-blind, placebo-controlled clinical trial. *Circulation: Arrhythmia and Electrophysiology,* 3**,** 46-53.

Saravanan, P., Bridgewater, B., West, A. L., O'neill, S. C., Calder, P. C. & Davidson, N. C. 2010. Omega-3 fatty acid supplementation does not reduce risk of atrial fibrillation after coronary artery bypass surgery: A randomized, double-blind, placebo-controlled clinical trial. *Circulation: Arrhythmia and Electrophysiology,* 3**,** 46-53.

Savci, S., Degirmenci, B., Saglam, M., Arikan, H., Inal-Ince, D., Turan, H. N. & Demircin, M. 2011. Short-term effects of inspiratory muscle training in coronary artery bypass graft surgery: a randomized controlled trial. *Scandinavian Cardiovascular Journal,* 45**,** 286-93.

Savluk, O. F., Kuscu, M. A., Guzelmeric, F., Gurcu, M. E., Erkilinc, A., Cevirme, D., . . . Kocak, T. 2017. Do preoperative oral carbohydrates improve postoperative outcomesin patients undergoing coronary artery bypass grafts? *Turkish Journal of Medical Sciences,* 47**,** 1681-1686.

Scantling-Birch, Y. 2019. RANDOMISED CONTROL TRIAL OF COGNITIVE BEHAVIOURAL THERAPY FOR PREOPERATIVE ANXIETY AND DEPRESSION IN A VASCULAR COHORT. *British Journal of Surgery,* 106**,** 15-15.

Setlik, R. F., Inman, A., Peacock, K., Aden, J., Paat, C., Stoerkel, E., . . . Walter, J. 2019. Pilot project assessing the impact of self-care techniques on post-surgical pain, fatigue, and inflammation. *Cancer research,* Conference: 2018 San Antonio Breast Cancer Symposium. United States. 79.

Shan, Y. S., Chao, Y. J., Kuo, S. E., Huang, C. Y., Tseng, Y. L., Hsu, K. H., . . . Lin, P. W. 2011. Peri-operative usage of enteric immunomodulating nutrients versus standard enteric nutrients for patients receiving major upper gastrointestinal surgery. A prospective randomized clinical trial. *Clinical Nutrition, Supplement,* 6 (1)**,** 224.

Shao, J., Xiao, T., Shi, M., Zhou, X., Wang, Z., Lin, T., . . . Zhang, A. 2019. Effect of multimedia-based nursing visit on perioperative anxiety in esophageal squamous cell carcinoma patients undergoing video-assisted thoracoscopic surgery. *Psychology, health & medicine,* 24**,** 1198-1206.

Siggeirsdottir, K., Olafsson, O., Jonsson, H., Iwarsson, S., Gudnason, V. & Jonsson, B. Y. 2005. Short hospital stay augmented with education and home-based rehabilitation improves function and quality of life after hip replacement: randomized study of 50 patients with 6 months of follow-up. *Acta Orthopaedica,* 76**,** 555-62.

Sigurdsson, E., Siggeirsdottir, K., Jonsson, H., Jr., Gudnason, V., Matthiasson, T. & Jonsson, B. Y. 2008. Early discharge and home intervention reduces unit costs after total hip replacement: results of a cost analysis in a randomized study. *International Journal of Health Care Finance & Economics,* 8**,** 181-92.

Sivaraman, A., Vanithamani & Manoharan, T. S. 2010. Yoga breathing exercise to reduce postoperative pulmonary complications in patients undergoing elective valve replacement for valvular heart disease: A randomized clinical trial. *Heart Surgery Forum,* 13**,** S83.

Sivaraman, A., Vanithamani & Manoharan, T. S. 2010. Yoga breathing exercise to reduce postoperative pulmonary complications in patients undergoing elective valve replacement for valvular heart disease: A randomized clinical trial. *Heart Surgery Forum,* 13**,** S83.

Skoffer, B., Maribo, T., Mechlenburg, I., Hansen, P. M., Soballe, K. & Dalgas, U. 2016. Efficacy of Preoperative Progressive Resistance Training on Postoperative Outcomes in Patients Undergoing Total Knee Arthroplasty. *Arthritis care & research,* 68**,** 1239-51.

Skrobot, W., Liedtke, E., Krasowska, K., Dzik, K. P., Flis, D. J., Samoraj-Dereszkiewicz, A., . . . Kaczor, J. J. 2019. Early rehabilitation program and vitamin D supplementation improves sensitivity of balance and the postural control in patients after posterior lumbar interbody fusion: A randomized trial. *Nutrients,* 11 (9) (no pagination).

Sommer, M. S., Trier, K., Vibe-Petersen, J., Missel, M., Christensen, M., Larsen, K. R., . . . Langberg, H. 2016. Perioperative Rehabilitation in Operable Lung Cancer Patients (PROLUCA): A Feasibility Study. *Integrative Cancer Therapies,* 15**,** 455-466.

Sorensen, L. S., Rasmussen, S. L., Yilmaz, M. N. & Thorlacius-Ussing, O. 2018. Supplementation with omega-3 fatty acids and survival after colorectal cancer surgery - A randomised controlled trial. *Colorectal Disease,* 20 (Supplement 4)**,** 122-123.

Sorensen, L. T., Hemmingsen, U. & Jorgensen, T. 2007. Strategies of smoking cessation intervention before hernia surgery--effect on perioperative smoking behavior. *Hernia,* 11**,** 327-33.

Sorensen, L. T. & Jorgensen, T. 2003. Short-term pre-operative smoking cessation intervention does not affect postoperative complications in colorectal surgery: a randomized clinical trial. *Colorectal Disease,* 5**,** 347-52.

Student, V., Vidlar, A., Bouchal, J., Vrbkova, J., Kolar, Z., Kral, M., . . . Vostalova, J. 2016. Cranberry intervention in patients with prostate cancer prior to radical prostatectomy. Clinical, pathological and laboratory findings. *Biomedical Papers of the Medical Faculty of Palacky University in Olomouc, Czech Republic,* 160**,** 559-565.

Suzuki, D., Furukawa, K., Kimura, F., Shimizu, H., Yoshidome, H., Ohtsuka, M., . . . Miyazaki, M. 2010. Effects of perioperative immunonutrition on cell-mediated immunity, T helper type 1 (Th1)/Th2 differentiation, and Th17 response after pancreaticoduodenectomy. *Surgery,* 148**,** 573-81.

Syed, U. a. M., Aleem, A. W., Wowkanech, C., Weekes, D., Freedman, M., Tjoumakaris, F., . . . Austin, L. S. 2018. Neer Award 2018: the effect of preoperative education on opioid consumption in

patients undergoing arthroscopic rotator cuff repair: a prospective, randomized clinical trial. *Journal of Shoulder and Elbow Surgery,* 27**,** 962-967.

Tepaske, R., Te Velthuis, H., Oudemans-Van Straaten, H. M., Bossuyt, P. M., Schultz, M. J., Eijsman, L. & Vroom, M. 2007. Glycine does not add to the beneficial effects of perioperative oral immune-enhancing nutrition supplements in high-risk cardiac surgery patients. *Jpen: Journal of Parenteral & Enteral Nutrition,* 31**,** 173-80.

Thomsen, T., Tonnesen, H., Okholm, M., Kroman, N., Maibom, A., Sauerberg, M. L. & Moller, A. M. 2010. Brief smoking cessation intervention in relation to breast cancer surgery: a randomized controlled trial. *Nicotine & Tobacco Research,* 12**,** 1118-24.

Tjoumakaris, F. P., Syed, U. a. M., Aleem, A. W., Wowkanech, C. D., Getz, C., Weekes, D., . . . Austin, L. 2017. Does patient education prior to arthroscopic rotator cuff repair decrease narcotic consumption? A randomized prospective study. *Orthopaedic Journal of Sports Medicine. Conference,* 5.

Tully, V., Wolever, T. M., Darling, P., Errett, L. & Keith, M. E. 2008. Pre-operative modification of dietary glycemic index improves pre but not post-operative indices of insulin resistance in patients undergoing coronary artery bypass graft surgery. *Journal of the American College of Nutrition,* 27**,** 168-76.

Turky, K. & Afify, A. M. A. 2017. Effect of Preoperative Inspiratory Muscle Training on Alveolar-Arterial Oxygen Gradients After Coronary Artery Bypass Surgery. *Journal of Cardiopulmonary Rehabilitation & Prevention,* 37**,** 290-294.

Turnock, A., Calder, P. C., West, A. L., Izzard, M., Morton, R. P. & Plank, L. D. 2013. Perioperative immunonutrition in well-nourished patients undergoing surgery for head and neck cancer: evaluation of inflammatory and immunologic outcomes. *Nutrients,* 5**,** 1186-99.

Van Hillegersberg, R., Valkenet, K., Trappenburg, J. & Backx, F. 2016. Preoperative inspiratory muscle training to prevent postoperative pneumonia in esophagectomy patients (prepare trial): a multicenter rct. *Diseases of the esophagus,* 29**,** 16A-.

Van Rooijen, S., Carli, F., Dalton, S., Thomas, G., Bojesen, R., Le Guen, M., . . . Slooter, G. 2019. Multimodal prehabilitation in colorectal cancer patients to improve functional capacity and reduce postoperative complications: The first international randomized controlled trial for multimodal prehabilitation. *BMC Cancer,* 19 (1) (no pagination).

Varaei, S., Shamsizadeh, M., Cheraghi, M. A., Talebi, M., Dehghani, A. & Abbasi, A. 2017. Effects of a peer education on cardiac self-efficacy and readmissions in patients undergoing coronary artery bypass graft surgery: a randomized-controlled trial. *Nurs Crit Care,* 22**,** 19-28.

Vasheghani Farahani, A., Yousefi Azar, A., Goodarzynejad, H. R., Khorrami, E., Hosseinzadeh-Attar, M. J., Oshnouei, S., . . . Ghourban Pour, F. 2017. Fish oil supplementation for primary prevention of atrial fibrillation after coronary artery bypass graft surgery: A randomized clinical trial. *Int J Surg,* 42**,** 41-48.

Vranceanu, M., De Lorenzo, D. & Simion, G. 2016. Effects of a ketogenic diet with MaV ketofast pro supplement in the preoperative fase of bariatric surgery. *Surgery for Obesity and Related Diseases,* 12 (7 Supplement 1)**,** S172-S173.

Waite, I., Deshpande, R., Baghai, M., Massey, T., Wendler, O. & Greenwood, S. 2017. Home-based preoperative rehabilitation (prehab) to improve physical function and reduce hospital length of stay for frail patients undergoing coronary artery bypass graft and valve surgery. *J Cardiothorac Surg,* 12**,** 91.

Walther, C., Fiess, A., Moebius-Winkler, S., Linke, A., Erbs, S., Schuler, G. & Walther, T. 2010. Preoperative exercise training is associated with less peri- and postoperative adverse events but similar long term outcome in patients with stable coronary artery disease. *European Journal of Cardiovascular Prevention and Rehabilitation,* 17**,** S59.

Wantanakorn, P., Harintajinda, S., Chuthapisith, J., Anurathapan, U. & Rattanatamrong, P. 2018. A New Mobile Application to Reduce Anxiety in Pediatric Patients Before Bone Marrow Aspiration Procedures. *Hosp Pediatr,* 8**,** 643-650.

Wijgman, A. J., Dekkers, G. H., Waltjé, E., Krekels, T. & Arens, H. J. 1994. [No positive effect of preoperative exercise therapy and teaching in patients to be subjected to hip arthroplasty]. *Nederlands tijdschrift voor geneeskunde,* 138**,** 949-952.

Wongkietkachorn, A., Wongkietkachorn, N. & Rhunsiri, P. 2018. Preoperative Needs-Based Education to Reduce Anxiety, Increase Satisfaction, and Decrease Time Spent in Day Surgery: A Randomized Controlled Trial. *World J Surg,* 42**,** 666-674.

Xu, Q. W., Xu, P. Y., Cen, Y. Y. & Li, W. M. 2019. Effects of preoperative oral administration of glucose solution combined with postoperative probiotics on inflammation and intestinal barrier function in patients after colorectal cancer surgery. *Oncology Letters,* 18**,** 694-698.

Zarei, B., Valiee, S., Nouri, B., Khosravi, F. & Fathi, M. 2018. The effect of multimedia-based nursing visit on preoperative anxiety and vital signs in patients undergoing lumbar disc herniation surgery: A randomised clinical trial. *J Perioper Pract,* 28**,** 7-15.

Zhang, X. D., Zhao, Q. Y., Fang, Y., Chen, G. X., Zhang, H. F., Zhang, W. X. & Yang, X. P. 2013. Perioperative comprehensive supportive care interventions for chinese patients with esophageal carcinoma: a prospective study. *Asian Pacific Journal of Cancer Prevention: Apjcp,* 14**,** 7359-66.

Zhang, X. D., Zhao, Q. Y., Fang, Y., Chen, G. X., Zhang, H. F., Zhang, W. X. & Yang, X. P. 2013. Perioperative comprehensive supportive care interventions for chinese patients with esophageal carcinoma: a prospective study. *Asian Pac J Cancer Prev,* 14**,** 7359-66.

Table 35. ClinicalTrials.gov: PROTOCOLS (searched 14/11/19)

	Title	Status	Study Results	Conditions	Interventions	Locations
1	Against All Odds -Prehabilitation in Urologic Cancer Surgery	Not yet recruiting	No Results Available	Postoperative Complications, Bladder Cancer	Behavioral: Prehabilitation	NR
2	Multimodal Prehabilitation in Thoracic Surgery	Not yet recruiting	No Results Available	Lung Cancer	Other: Usual care<br>•Other: Multimodal prehabilitation	Spain
3	A Trimodal Prehabilitation Study for Patients Undergoing Major Abdominal Surgery	Recruiting	No Results Available	Pilot Study	Other: Monitoring using a FitBit	UK
4	Prehabilitation in Bariatric Surgery	Not yet recruiting	No Results Available	Obesity,<br>Bariatric Surgery Candidate	Behavioral: Prehabilitation<br>Behavioral: Control	Las Palmas
5	Incentive Spirometry Prehabilitation Study	Recruiting	No Results Available	Pulmonary Disease	Behavioral: Spirometry Group	USA
6	Preoperative Exercise for Patients Undergoing Complex Cancer Surgery	Not yet recruiting	No Results Available	Postoperative Complications,<br>Physical Activity,<br>Surgery--Complications	Other: Control<br>Other: Exercise - HIIT Intervention	Ireland
7	Effect of Prehabilitation Protocol on Quality of Life After Thoracoscopic Surgery	Completed	No Results Available	Thoracoscopy,<br>Prehabilitation	Other: Educational nursing protocol<br>Other: Routine hospital care	Egypt
8	Prehabilitation in Esophageal Surgery (PRESS)	Recruiting	No Results Available	Esophageal Cancer Surgery	Other: Prehabilitation	IRCCS San Raffaele Scientific Institute
9	Prehabilitation Versus Enhanced Recovery Program for Elective Colorectal Cancer Surgery.	Recruiting	No Results Available	Colorectal Cancer,<br>Insulin Resistance	Procedure: Prehabilitation + ERAS<br>Procedure: ERAS	Hungary
10	Better Before - Better After: Prehabilitation Program for Older Patients Awaiting Total Hip Replacement	Recruiting	No Results Available	Arthritis of Hip	Behavioral: Exercise	Norway
11	A Personalized Prehabilitation Intervention In Elective Joint Replacement Surgery	Not yet recruiting	No Results Available	Arthropathy of Knee Joint,<br>Arthropathy of Hip Joint	Behavioral: personalized prehabilitation	USA
12	Prehabilitation Intervention to Maximize Early Recovery (PRIMER) in Liver Transplantation	Active, not recruiting	No Results Available	Liver Diseases,<br>End Stage Liver Disease,<br>Frailty	Device: Nokia GO Wearable StepTracker<br>Other: Medication Reminder<br>Other: Weekly Check-in appointment with study team/provider	USA
13	Pre-habilitation of Patients Scheduled for Cardiac Valve Surgery	Not yet recruiting	No Results Available	Valvular Heart Disease	Behavioral: Tele - Cardiac Pre-Rehabilitation	NR
14	The Wessex Fit-4-Cancer Surgery Trial	Recruiting	No Results Available	Cancer	Behavioral: SRETP<br>Behavioral: Psychological support	UK
15	Prehabilitation to Improve Cancer Surgery Outcomes	Recruiting	No Results Available	Gastrointestinal Cancer,<br>Prehabilitation<br>Surgery<br>Physical Activity	Behavioral: Physical Prehabilitation<br>Behavioral: Psychological Prehabilitation	Canada
16	Efficacy of Preoperative Muscle Training on Postoperative Orthopaedic Surgery Recovery	Recruiting	No Results Available	Arthroplasty,<br>Osteoarthritis	Other: Rehabilitation<br>Other: Standard of Care	Ottawa
17	Improving Outcomes in Cancer Patients With a Nutritional and Physical Conditioning Prehabilitation Program	Recruiting	No Results Available	Pancreatic Cancer,<br>Liver Cancer,<br>Bile Duct Cancer,<br>Hepatobiliary Cancer,<br>Surgery	Other: Exercise<br>Other: Nutrition<br>Behavioral: Relaxation techniques	Canada

18	Implementation of a Trimodal Prehabilitation Program as a Preoperative Optimization Strategy in Cardiac Surgery	Recruiting	No Results Available	Coronary Artery Disease,<br>Valvular Heart Disease	Behavioral: pre-habilitation	Spain
19	Effects of Prehabilitation and Early Mobilization for Patients Undergoing Pancreas Surgery.	Recruiting	No Results Available	Pancreas Cancer	Other: Prehabilitation<br>Other: Routine care<br>Other: Extra early mobilization<br>Other: Standard mobilization	Sweden
20	Effectiveness of Prehabilitation for Patients Undergoing Lumbar Spinal Stenosis Surgery	Recruiting	No Results Available	Lumbar Spinal Stenosis	Prehabilitation<br>Other: 6-week prehabilitation	Hong Kong
21	High-Intensity Interval vs. Moderate Continuous Training in Surgical Prehabilitation	Completed	No Results Available	Colorectal Cancer,<br>Physical Activity	Procedure: HIT<br>Procedure: MCT	Canada
22	Trimodal Prehabilitation for Cystectomy Patients to Enhance Post-operative Care	Unknown status	No Results Available	Bladder Cancer,<br>Nutrition Aspect of Cancer	Combination Product: Trimodal Prehab & ERP<br>Other: No Prehab; ERP Alone	NR
23	Enhancing Fitness With Preoperative Exercise in Colorectal Cancer Surgery	Not yet recruiting	No Results Available	Colorectal Cancer	Behavioral: Exercise intervention	UK
24	A Study of the Efficacy of ONS to Reduce Postoperative Complications Associated With Pancreatic Surgery	Active not recruiting	No Results Available	Pancreatic Cancer,<br>Chronic Pancreatitis	Dietary Supplement: Ensure Surgical<br>Other: Nutrition counseling<br>Other: Low-intensity exercise therapy	USA
25	TSA Exercise Prehabilitation in Older Adults	Recruiting	No Results Available	Postoperative Delirium and Delayed,<br>Functional Recovery	Behavioral: Aerobic Exercise Training (AET)	USA
26	Preoperative Inspiratory Muscle Training Effects on the Perioperative Inflammatory Reaction in Cardiac Surgery.	Unknown status	No Results Available	Thoracic Surgery,<br>Prehabilitation,<br>Respiratory Therapy	Other: Prehabilitation with inspiratory muscle training	NR
27	Prehabilitation in Frail Colon Cancer	Recruiting	No Results Available	Colon Cancer,<br>Fragility	Behavioral: Prehabilitation	Belgium
28	Prehabilitation in Colorectal Cancer	Recruiting	No Results Available	Colon Cancer	Behavioral: Prehabilitation<br>Other: Rehabilitation	Belgium
29	COgnitive and Physical Exercise (COPE) Prehabilitation Pilot Feasibility Study	Completed	No Results Available	Cognitive Impairment,<br>Physical Impairment,<br>Postoperative Complications	Behavioral: Cognitive and physical prehabilitation<br>Other: Active attention control	USA
30	The Impact of Trimodal Prehabilitation Strategy on Patients Undergoing Thoracoscopic Lobectomy	Completed	No Results Available	Perioperative Recovery<br>Prehabilitation<br>Thoracoschisis, Lung Cancer, Lobectomy	Behavioral: Trimodal prehabilitation management	China
31	Piloting Prehabilitation Before Abdominal Surgery	Completed	No Results Available	Frailty	Behavioral: Prehabilitation	USA
32	Cardiovascular Prehabilitation in Patients Awaiting Heart Transplantation (PREHAB HTx Study)	Recruiting	No Results Available	Heart Failure	Behavioral: High-Intensity Interval Training	Canada
33	Prehabilitation for Elective Major Abdominal Surgery	Recruiting	No Results Available	Prehabilitation	Other: Prehabilitation	Switzerland
34	Physiotherapy Prehabilitation in Patients Undergoing Cardiac or Thoracic Surgery	Completed	No Results Available	Coronary Artery Disease,<br>Lung Cancer,<br>Lung Tumor	Other: Walking Programme<br>Device: Incentive Spirometer<br>Other: Deep Breathing Exercises	UK
35	The Prehabilitation Study: Exercise Before Surgery to Improve Patient Function in People	Recruiting	No Results Available	Cancer,<br>Frailty	Behavioral: Prehabilitation Program	Canada

36	The Impact of Prehabilitation Bundle on Perioperative Outcome for Frail Elderly Patients Undergoing Major Abdominal Surgery	Unknown status	No Results Available	Complication, Postoperative Perioperative/Postoperative Complications	Dietary Supplement: Ensure, Resource 2.0 and Glucerna Other: Cognitive Training, Memory Card Game Device: 'Threshold' Inspiratory Muscle Trainer, Respironics of New Jersey Inc	Singapore
37	Developing Prehabilitation Program in Patients With Operable Pancreatic Cancer Perioperatively and Following Surgery	Unknown status	No Results Available	Pancreatic Cancer	Behavioral: Physical and nutrition program	Taiwan
38	Pre-Habilitation Exercise Intervention	Recruiting	No Results Available	Inflammatory Bowel Diseases, Colon Cancer, Rectal Cancer, Diverticular Disease	Behavioral: Exercise for Cancer Patients	USA
39	Effect of Prehabilitation in Gastroesophageal Adenocarcinoma: Study Protocol of a Multicentric, RCT	Unknown status	No Results Available	Oesophageal Cancer, Gastric Cancer	Procedure: prehabilitation Other: nutritional support	France
40	Prehabilitation Using Aquatic Exercise	Completed	No Results Available	Osteoarthritis, Knee	Behavioral: Aquatic Prehab	, USA
41	Prehabilitation - Enhanced Recovery After Colorectal Surgery	Completed	No Results Available	Surgical Operation With Reversal of External Stoma, Colorectal Surgery	Other: Cardiorespiratory and resistance training. Other: Reference	Switzerland
42	Effects of Prehabilitation in Gastric Cancer Patients With Metabolic Syndrome on Perioperative Outcome	Completed	No Results Available	Stomach Neoplasms, Metabolic Syndrome X	Behavioral: pre-operative prehabilitation	China
43	Prehabilitation to Improve Functional and Clinical Outcomes in Patients With Aortic Stenosis	Recruiting	No Results Available	Aortic Stenosis	Other: Prehabilitation Other: Usual Care	USA
44	Prehabilitation in Elective Colorectal Resection: A Pilot Study (Prehab)	Unknown status	No Results Available	Colorectal Cancer	Other: Physiatry Assessment Other: Routine Care	Canada
45	The Feasibility of a Prehabilitation Program in Liver Transplant Population at Barnes-Jewish Hospital	Completed	No Results Available	End Stage Liver Disease	Physical Activity Procedure: Exercise	NR
46	Prehabilitation for Prostate Cancer Surgery	Completed	No Results Available	Prostate Cancer Patients Undergoing Radical Prostatectomy	Behavioral: Prehabilitation (PREHAB)	Canada
47	Effectiveness of Prehabilitation Program for High-risk Patients Underwent Abdominal Surgery	Completed	No Results Available	Abdominal Surgical Patient, High-risk Patient	Behavioral: Prehabilitation	Spain
48	Functional Prehabilitation and Major Elective Surgery	Completed	No Results Available	Surgical Complication, Physical Therapy, Timed Up and Go	Procedure: Functional Assessment Procedure: Prehabilitation	USA
49	Pre-habilitation Program for Elective Coronary Artery Bypass Graft Surgery	Completed	No Results Available	Patients Waiting for Elective CABG Surgery	Behavioral: Prehab	Canada
50	Prehabilitation for Esophageal Resection Surgery	Completed	No Results Available	Esophageal Cancer, Surgery	Behavioral: Exercise	Canada
51	Prehabilitation in Liver Surgery	Completed	No Results Available	Colorectal Cancer, Colorectal Liver Metastasis, Liver Surgery, Exercise Intervention	Behavioral: Exercise Intervention	UK
52	Enhancing Outcomes After Colon Surgery	Completed	No Results Available	Colorectal Surgery	Behavioral: Prehabilitation	Canada
54	Programme to Optimise Risk Factors in Patients Waiting for Coronary Artery Bypass Surgery	Completed	No Results Available	Coronary Artery Disease	Behavioral: a nurse led home-based education and support programme	UK

Table 36. Protocols from searches

First author	Trial registration	Type of prehabilitation	Type of surgery	Status
Sommer 2014	NCT01893580	Perioperative rehabilitation	Lung cancer surgery	Terminated (Due to low recruitment rate, the study design is changed to a feasibility study)
Ciacio et al., 2014	NCT02041871	immunionutrition	Liver resection	Completed recruitment
Dowsey et al 2014	ACTRN12611001184965	Mindfulness training	TKA	Prospectively registered
Le Roy et al., 2016	NCT02780921	multimodal (nutrition/psychological)		Unknown
Loughney et al., 2016	NCT01914068	In hospital exercise training programme		Completed recruitment
Lauridsen et al., 2017	NCT02188446	Stop smoking and alcohol	Radical cystectomy	Completed recruitment
Merki-Kunzli et al., 2017	NCT02746731	cardiorespiratory and resistance training program	Colorectal surgery	Completed not published?
Wong et al., 2017	NCT02531620	Physiatrist directed prehab intervention	Colorectal surgery	Unknown
Snowden et al., 2017	ISRCTN36257982	alcohol cessation	Orthopaedic surgery	Completed but not published
Steffans et al., 2018	ACTRN12617001129370	Physical activity	Major cancer surgery	No results available
Woodfield et al., 2018	ACTRN12617000587303	High intensity interval training	Abdominal surgery	Not published yet
McIssac et al., 2018	NCT02934230	Exercise intervention	Cancer surgery	Still recruiting
Mulder et al., 2018	ID: NTR6113	Antibiotic prophylaxis	Colorectal surgery	Unclear
O'Brien et al., 2018	ACTRN12617000357358	Exercise intervention	hip and knee arthroplasty	Prospectively registered
van Rooijen et al., 2019	NTR5947	Multimodal intervention	Colorectal surgery	Still recruiting

Table 37. Guidance for assessing Risk of Bias in the Prehabilitation systematic review (RCTs)

<table>
<tr><td colspan="3">RANDOM SEQUENCE GENERATION (method of generating a random allocation sequence to assign participants to the intervention of control group)</td></tr>
<tr><td colspan="3">Where do I find information about this?
• Check Methods (generally near the beginning) or Statistical Analysis sections.
• If not obvious, use Ctrl F on keyboard and type in the word ‘random’ in the Find box to identify all words that contain ‘random’ in the PDF of the paper.
• If you cannot identify the root word ‘random’ anywhere in the paper the study is unlikely to be an RCT (mark this as a reason for exclusion in section 2 of the data extraction form and do not extract data).</td></tr>
<tr><td colspan="3">How do I decide on whether the study is at low, high or unclear risk of bias for sequence generation?</td></tr>
<tr><td>Low risk
Paper describes a random component in the sequence generation process, e.g.
• A random number table; a computer random number generator; internet based randomisation programs (e.g. Sealed Envelope) (most common).
• Coin tossing; shuffling cards or envelopes; throwing dice; drawing of lots/slips; minimization (less common).</td><td>High risk
Paper describe a non-random approach of generating the sequence (basically anything that is not in the Low risk box) e.g.
• Odd or even date of birth; date (or day) of admission; hospital or clinic record number; laboratory test results, judgement of clinician, preference of participant, availability of intervention).</td><td>Unclear risk
Not enough information provided to make a judgement of low or high risk, e.g.
• A lot of papers state ‘patients were randomly allocated’ or ‘patients were prospectively randomised’ but do not state how.</td></tr>
<tr><td colspan="3">ALLOCATION CONCEALMENT (concealing the allocation sequence generated above from participants and those assigning participants to intervention groups, i.e. nobody knows which intervention the next participant will receive)</td></tr>
<tr><td colspan="3">Where do I find information about this?</td></tr>
</table>

- It usually comes straight after the description of **RANDOM SEQUENCE GENERATION**. Don't be surprised if you don't find it; this information is often missing.
- Again, you could use Ctrl F on keyboard and type in the word 'conceal' in the Find box, just to double check.

How do I decide on whether the study is at low, high or unclear risk of bias for allocation concealment?

Low risk	*High risk*	*Unclear risk*
Papers states that one of the following was used:<br>• Central allocation (including telephone, web-based, and pharmacy-controlled randomization).<br>• Sequentially numbered drug containers of identical appearance.<br>• Sequentially numbered, opaque, sealed envelopes – all 3 features of the envelopes must be described.	Anything that is not in the *Low risk* box, e.g.<br>• Using an open random allocation schedule (e.g. a list of random numbers).<br>• Assignment envelopes were used without appropriate safeguards (e.g. if envelopes were unsealed or non-opaque or not sequentially numbered).<br>• Alternation or rotation.<br>• Date of birth.<br>• Case record number.<br>• Any other explicitly unconcealed procedure.	• No mention of allocation concealment.<br>• The method is not described in sufficient detail, e.g. if the use of assignment envelopes is described, but it remains unclear whether envelopes were sequentially numbered, opaque and sealed.

BLINDING OF PARTICIPANTS AND PERSONNEL (if doctors and other healthcare workers are not blinded to the intervention group of patients participating in the RCT they may provide better care to one group but not the other, which could influence the study outcomes. If patients are not blinded to their intervention group, they may behave in ways that will influence the study outcomes, e.g. patients who received the intervention being studied get out of bed quicker after the operation so their length of stay will be shorter)

Where do I find information about this?

Perry R, *et al. BMJ Open* 2022; 11:e050806. doi: 10.1136/bmjopen-2021-050806

<table>
<tr><td colspan="3">• This may be anywhere in the paper (most commonly in the Methods and Abstract) but the easiest way to find it is to use Ctrl F on keyboard and type in the word 'blind' in the Find box to identify all words that contain 'blind' in the PDF of the paper.

NOTE: Most studies of exercise interventions, psychosocial interventions and dietary interventions that involve weight loss can't be blinded from participants or their healthcare team.</td></tr>
<tr><td colspan="3">How do I decide on whether the study is at low, high or unclear risk of bias for blinding of participants and healthcare personnel?</td></tr>
<tr><td>Low risk

There is NO BLINDING but:

• The healthcare team looking after the patients after surgery have nothing to do with the research team (e.g. intervention may have been carried out in the pre-operative assessment setting, which is separate from the surgical team.

There is BLINDING and:

• Methods of blinding of participants and personnel described sufficiently and deemed adequate, e.g. in nutritional interventions involving oral supplements or feeds, there is mention that 'feeds or pills looked identical'.

Check each study carefully because although patients may not be blinded, healthcare personnel may be (so above points would only apply to healthcare personnel).

Use above rules for ALL OUTCOMES.</td>
<td>High risk

There is NO BLINDING and:

• The healthcare team looking after the patients after surgery are also involved in the research.

Use above rules for ALL OUTCOMES.

If a study is described as single-blind and mentions blinding of either participant, personnel or outcome assessor, the other two will automatically be rated as high risk of bias</td>
<td>Unclear risk

• Study labelled as blinded but no further detail provided.

• You can check this box if the outcome is not measured (and state not recorded, or NR, in the support for judgement).

If we can't tell if blinding is broken we will put UNCLEAR</td></tr>
</table>

Perry R, *et al. BMJ Open* 2022; 11:e050806. doi: 10.1136/bmjopen-2021-050806

**BLINDING OF OUTCOME ASSESSORS** (the individuals who measure the outcomes. Remember that for patient reported outcomes the patient is the outcome assessor).		
**Where do I find information about this?**<br>• This may be anywhere in the paper (most commonly in the Methods and Abstract) but the easiest way to find it is to use Ctrl F on keyboard and type in the word 'blind' in the Find box to identify all words that contain 'blind' in the PDF of the paper.		
**How do I decide on whether the study is at low, high or unclear risk of bias for blinding of outcome assessors?**		
Low risk<br>• The clinicians (or others) who assessed the outcomes are blinded to the patients' group allocation.<br>• The outcome measurement is objective therefore unlikely to be influenced by lack of blinding (e.g. **Mortality (dead/alive), Hospital readmission (yes/no),**	*High risk*<br>• There is no blinding and the outcome assessment is subjective (e.g. requires clinician or patient judgement). This applies to **ALL Postoperative complications, Patient reported outcomes, and Adverse events.**<br>For outcomes specific to the prehab intervention, you must make an assessment as to whether the outcome is subjective or not (e.g. weight as an outcome measure for weight loss interventions is objective because it is simply recording a number from the weighing scales; Knee Society Clinical Rating Score as an outcome measure following an exercise intervention is subjective because it requires the clinician to decide what the score is).	*Unclear risk*<br>• You can check this box if the outcome is not measured (and state not recorded, or NR, in the support for judgement).
**INCOMPLETE OUTCOME DATA** (are any participants excluded from the trial AFTER randomisation?		

Perry R, et al. BMJ Open 2022; 11:e050806. doi: 10.1136/bmjopen-2021-050806

LOW RISK<br>Relevant with data after randmisation<br>Any one of the following:<br>• No missing outcome data<br>• EXCERISE caution Reasons for missing outcome data unlikely to be related to true outcome (for survival data, censoring unlikely to be introducing bias)<br>• Missing outcome data balanced in numbers across intervention groups, with similar reasons for missing data across groups<br>o Difference in missing data between the groups not greater than 10%<br>o i.e. intervention group of 120 has 6 drop out (5% of trial arm) and control group of 100 has 2 drop out (2% of trial arm): difference in missing data is 3% therefore **LOW RISK**<br>• Dropouts – we need to decide for ourselves if the reasons for dropouts were low risk, and we need to justify this<br><br>• For dichotomous outcome data, the proportion of missing outcomes compared with observed event risk not enough to have a clinically relevant impact on the intervention effect estimate<br>• For continuous outcome data, plausible effect size (difference in means or standardized difference in means) among missing outcomes not enough to have a clinically relevant impact on observed effect size<br>• Missing data have been imputed using appropriate methods - if medians are provided rather than means, or no SDs have been provided) = only low risk as this data can't be used in our meta-analyses
HIGH RISK<br>Any one of the following:<br>• Reason for missing outcome data likely to be related to true outcome, with either imbalance in numbers or reasons for missing data across intervention groups<br>o Difference in missing data between the groups greater than 10%<br>o i.e. intervention group of 120 has 6 drop out (5% of trial arm) and control group of 100 has 26 drop out (26% of trial arm): difference in missing data is 11% therefore **HIGH RISK**<br>• Overall missing data greater than 10% of the total randomised population<br>• Stated as 'intention-to-treat analysis' but doesn't use this<br>• For dichotomous outcome data, the proportion of missing outcomes compared with observed event risk enough to induce clinically relevant bias in intervention effect estimate<br>• For continuous outcome data, plausible effect size (difference in means or standardized difference in means) among missing outcomes enough to induce clinically relevant bias in observed effect size<br>• 'As-treated' analysis done with substantial departure of the intervention received from that assigned at randomization

• Potentially inappropriate application of simple imputation
UNCLEAR RISK<br>Any one of the following:<br>• Insufficient reporting of attrition/exclusions to permit judgement of 'Yes' or 'No' (e.g. number randomized not stated, no reasons for missing data provided)<br>• Dropouts not mentioned
**SELECTIVE OUTCOME REPORTING**
LOW RISK<br>Any of the following: expected,<br>Unclear if no protocol,<br>High risk<br>• The study protocol is available and all of the study's pre-specified (primary and secondary) outcomes that are of interest in the review have been reported in the pre-specified way – use trial registration number if available to locate protocol<br>• The study protocol is not available but it is clear that the published reports include all expected outcomes, including those that were pre-specified (convincing text of this nature may be uncommon).
HIGH RISK<br>Any one of the following:<br>• Not all of the study's pre-specified primary and secondary outcomes have been reported<br>○ Pre-specified in methods section<br>○ Or pre-specified in protocol<br>• Results for primary and secondary outcomes presented, but outcomes not stated in Methods<br>• One or more primary or secondary outcomes is reported using measurements, analysis methods or subsets of the data (e.g. subscales) that were not pre-specified<br>• One or more reported primary or secondary outcomes were not pre-specified (unless clear justification for their reporting is provided, such as an unexpected adverse effect)<br>• One or more outcomes of interest in the review are reported incompletely so that they cannot be entered in a meta-analysis – any data excluded from the analysis despite the data being available (i.e. so the reviewers decided not to include it in the meta-analysis)<br>• The study report fails to include results for a key outcome that would be expected to have been reported for such a study
UNCLEAR RISK<br>Insufficient information to permit judgement of 'Yes' or 'No'.

Perry R, *et al. BMJ Open* 2022; 11:e050806. doi: 10.1136/bmjopen-2021-050806

• No protocol is available<br>**Most studies will fall in this category**
**OTHER BIAS**
LOW RISK<br>The study appears to be free of other sources of bias.
HIGH RISK<br>There is at least one important risk of bias. For example, the study:<br>• Had a potential source of bias related to the specific study design used<br>• Stopped early due to some data-dependent process (including a formal-stopping rule)<br>• Had extreme baseline imbalance<br>• Has been claimed to have been fraudulent<br>• Had some other problem
UNCLEAR RISK<br>There may be a risk of bias, but there is either:<br>• Insufficient information to assess whether an important risk of bias exists<br>• Insufficient rationale or evidence that an identified problem will introduce bias.

Perry R, *et al. BMJ Open* 2022; 11:e050806. doi: 10.1136/bmjopen-2021-050806

Table 38. Sensitivity analyses: fixed versus random effects for all outcomes

	Mortality		Length of stay		Total PO complications		Total PO infective complications		Pneumonia		PPCs		Wound infection	
	**Random Effects**	**Fixed Effects**	**Random Effects**	**Fixed Effects**	**Random Effects**	**Fixed Effects**	**Random Effects**	**Fixed Effects**	**Random Effects**	**Fixed Effects**	**Random Effects**	**Fixed Effects**	**Random Effects**	**Fixed Effects**
**Immuno nutrition**	RR=0.55 (95% CI (0.21to 1.42), P=0.22	RR=0.56 (95% CI 0.23 to 1.35), P=0.20	MD=-2.11 (95% CI -3.07 to -1.15), P<0.00001	MD=-2.55 (95% CI -3.16 to -1.93), P<0.00001	RR=0.70 (95% CI 0.49 to 0.99), P=0.04	RR=0.78 (95% CI 0.65 to 0.94), P=0.009	RR=0.64 (95% CI 0.40 to 1.01), P=0.05	RR=0.56 (95% CI 0.39 to 0.82), P=0.002	RR=0.52 (95% CI 0.18 to 1.44), P=0.21	RR=0.56 (95% CI 0.18 to 1.44), P=0.21			RR=0.71 (95% CI, 0.51 to 0.99), P=0.05	RR=0.70 (95% CI, 0.52 to 0.96, P=0.03
**ONS**	RR=1.18 (95% CI 0.23 to 6.11), P=0.85	RR=1.12 (95% CI 0.40 to 3.16), P=0.82	MD=-0.54 (95% CI -1.38 to 0.29), P=0.20	MD=-0.54 (95% CI -1.38 to 0.29), P=0.20										
**Pre/Pro-biotics**	RR=0.76 (95% CI 0.17 to 3.42), P=0.23	RR=0.94 [95% CI 0.40, 2.22], P=0.89					RR 0.48 (95% CI 0.14 to 1.62), P=0.23	RR 0.72 (95% CI 0.46 to 1.11), P=0.14						
**Weight Loss**			MD=0.22 (95% CI -0.46 to 0.91), P=0.53	MD=0.40 (95% CI -0.14.to 0.66), P=0.002										
**Exercise**	RR=0.74 (95% CI 0.23 to 2.35), P=0.61	RR=0.72 (95% CI 0.24 to 2.21), P=0.57	MD=-0.34 (95% CI -0.79 to 0.11), P=0.14	MD=-0.22 (95% CI (-0.53, 0.09), P=0.16	RR=0.83 (95% CI 0.61 to 1.12), P=0.22	RR=0.81 (95% CI 0.60 to 1.08), P=0.18			RR=0.72 (95% CI 0.35 to 1.44), P=0.35	RR=0.73 (95% CI 0.37 to 1.45), P=0.37	RR=0.54 (95% CI 0.39 to 0.75), P=0.0003	RR=0.53 (95% CI 0.38 to 0.75), P=0.0003		
**IMT**	RR=1.49 (95% CI 0.60 to 3.69), P=0.39	RR=1.43 (0.61, 3.32), P=0.41	MD=-1.81 (-2.31, -1.32), P<0.00001	MD=-1.81 (95% CI (-2.31, -1.32), P<0.00001					RR=0.69 (95% CI 0.46, 1.05), P=0.08	RR=0.77 (95% CI 0.59, 1.01), P=0.06	RR=0.55 (95% CI 0.38 to 0.80), P=0.002	RR=0.53 (95% CI 0.39 to 0.73), P<0.0001		
**IS**			MD=-2.39 (95% CI -5.50 to 0.72), P=0.13	MD=-0.29 (95% CI -0.52 to -0.06), P=0.01							RR=0.68 (95% CI 0.25 to 1.81), P=0.44	RR=0.84 (95% CI 0.66 to 1.08), P=0.18		
**Multimo dal**	RR=0.67 (95% CI, 0.23 to 1.97), P=0.46	RR=0.71 (95% CI, 0.27 to 1.83), P=0.47	MD=-1.67 (95% CI -2.31 to -1.03), P<0.00001	MD=-0.86 (95% CI -1.06 to -0.65), P<0.00001	RR=0.84 (95% CI 0.72 to 0.97), P=0.02	RR=0.83 (95% CI 0.70 to 0.98), P=0.03			RR=0.56 (95% CI 0.28 to 1.12), P=0.10	RR=0.58 (95% CI 0.31 to 1.12), P=0.11				
**Educatio n**	RR=0.83 (95% CI 0.24 to 2.95), P=0.78	RR=0.81 (95% CI, 0.23 to 2.80), P=0.74	MD=0.00 (95% CI -0.40 to 0.40), P=1.00	MD=-0.10 (95% CI -0.32 to 0.12), P=0.36										
**Psycholo gical**			MD=-0.79 (95% CI -1.87 to 0.29), P=0.15	MD=-0.79 (95% CI -1.87 to 0.29), P=0.15										
**Smoking Cessatio n**	RR=0.44 (95% CI 0.15 to 0.15 to 1.33), P=0.15	RR=0.43 (95% CI 0.15 to 1.26), P=0.12	MD=-1.86 (95% CI -2.72 to -1.00), P<0.0001	MD=-1.86 (95% CI -2.72 to -1.00), P<0.0001	RR=0.65 (95% CI 0.43 to 0.99), P=0.05	RR=0.74 (95% CI 0.59 to 0.93), P=0.009	RR=0.63 (95% CI 0.44 to 0.90), P=0.01	RR=0.66 (95% CI 0.52 to 0.85), P=0.001	RR=0.87 (95% CI 0.21 to 3.56), P=0.85	RR=0.92 (95% CI 0.41 to 2.09), P=0.84			RR=0.36 (95%CI 0.16 to 0.79, P=0.01	RR=0.31 (95%CI 0.16 to 0.58, P=0.0003
